# Selective Coupling of 1,2‐Bis‐Boronic Esters at the more Substituted Site through Visible‐Light Activation of Electron Donor–Acceptor Complexes

**DOI:** 10.1002/anie.202202061

**Published:** 2022-03-07

**Authors:** Hui Wang, Jingjing Wu, Adam Noble, Varinder K. Aggarwal

**Affiliations:** ^1^ School of Chemistry University of Bristol, Cantock's Close Bristol BS8 1TS UK; ^2^ Current address: Frontiers Science Center for Transformative Molecules, School of Chemistry and Chemical Engineering Shanghai Jiaotong University No. 800, Dongchuan Road Shanghai 200240 China

**Keywords:** 1,2-Boron Shift, Alkyl Radicals, Boronate Complexes, Electron Donor–Acceptor Complexes, Photoactivation

## Abstract

1,2‐Bis‐boronic esters are useful synthetic intermediates particularly as the two boronic esters can be selectively functionalized. Usually, the less hindered primary boronic ester reacts, but herein, we report a coupling reaction that enables the reversal of this selectivity. This is achieved through the formation of a boronate complex with an electron‐rich aryllithium which, in the presence of an electron‐deficient aryl nitrile, leads to the formation of an electron donor–acceptor complex. Following visible‐light photoinduced electron transfer, a primary radical is generated which isomerizes to the more stable secondary radical before radical‐radical coupling with the arene radical‐anion, giving β‐aryl primary boronic ester products. The reactions proceed under catalyst‐free conditions. This method also allows stereodivergent coupling of cyclic *cis*‐1,2‐bis‐boronic esters to provide *trans*‐substituted products, complementing the selectivity observed in the Suzuki–Miyaura reaction.

## Graphical abstract

The site‐selective monofunctionalization of 1,2‐bis‐boronic esters was achieved by using a catalyst‐free photoinduced coupling with (hetero)aryl nitriles. The reaction proceeds through electron donor–acceptor (EDA) complex‐driven deboronation and radical 1,2‐boron shift, giving β‐aryl primary boronic ester products. The reaction also works with primary, secondary, and tertiary boronic esters.

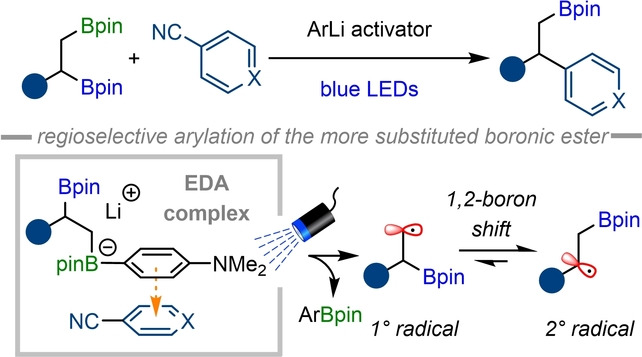

Organoboron compounds are valuable building blocks in modern synthesis, with applications that span pharmaceuticals, natural products, and functional materials.^[1]^ In recent years, 1,2‐bis‐boronic esters have received significant attention since they are readily accessible from alkenes and because the two boronic esters can be selectively functionalized.[^2^, ^3^] Regioselective functionalization has been established in reactions of 1,2‐bis‐boronic esters derived from terminal alkenes, where the less hindered primary boronic ester reacts preferentially over secondary or tertiary positions, including in Suzuki–Miyaura cross‐couplings (Figure 1a, i)^[4]^ and homologations with lithiated carbenoids.^[5]^

**Figure 1:**
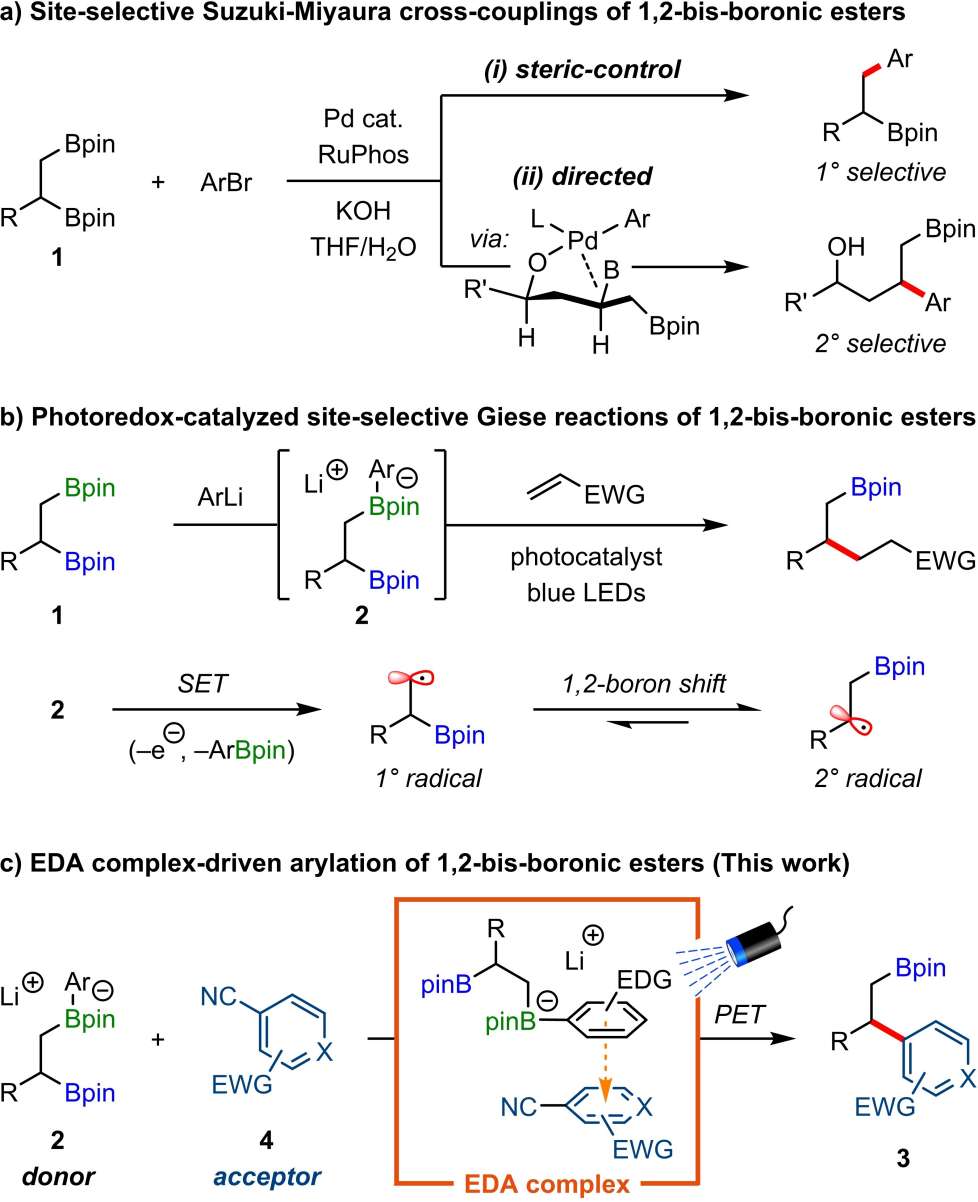
Site‐selective reactions of 1,2‐bis‐boronic esters.

Reactions that are selective for the more hindered position represent a greater synthetic challenge with only a few reports. Morken demonstrated that amine‐*N*‐oxides selectively oxidize secondary boronic esters because of the greater migratory aptitude of secondary over primary alkyl groups.^[6]^ In addition, the same group found *β*‐hydroxy directing groups could reverse the regioselectivity of palladium‐catalyzed cross‐couplings in favor of secondary boronic esters (Figure 1a, ii).^[7]^ However, the reliance on directing groups limits substrate generality, and the sensitivity of palladium‐catalyzed cross‐couplings to sterics hinders their application to tertiary boronic esters. Therefore, there remains a need for more general approaches to these regioselective couplings of 1,2‐bis‐boronic esters.

Recently, we disclosed a photoredox‐catalyzed mono‐deboronative Giese‐type reaction between 1,2‐bis‐boronic esters **1** and electron‐deficient alkenes (Figure 1b).^[8]^ Interestingly, despite initial activation of the primary boronic ester with an aryllithium to form arylboronate complex **2**, single‐electron transfer (SET)‐induced deboronative radical addition led exclusively to substitution of the secondary boronic ester. This selectivity arises from a rapid 1,2‐boron shift of the primary alkyl radical to the thermodynamically favored secondary alkyl radical.^[9]^ We reasoned that if this 1,2‐boron shift occurred during a radical‐mediated deboronative arylation, high regioselectivity for secondary‐coupled product **3** should be observed (Figure 1c), which is complementary to that of palladium‐catalyzed Suzuki–Miyaura cross‐couplings. In our design plan, we recognized that an electron‐rich aromatic ring of boronate complex **2** could interact with a suitable electron‐deficient aromatic coupling partner (**4**) to form an electron donor–acceptor (EDA) complex.^[10]^ Subsequent photoinduced electron transfer (PET) would initiate a reaction cascade that ultimately led to the coupled product **3**. The advantage of exploiting EDA complexes is their ability to undergo PET in the absence of photocatalysts, therefore our proposed deboronative arylation should proceed under catalyst‐free conditions.^[11]^ Herein, we demonstrate the successful realization of this strategy in highly regioselective couplings of (hetero)aryl nitriles with the more hindered position of a broad range of 1,2‐bis‐boronic esters. Furthermore, we show that this process is general and can also be applied to primary, secondary, and tertiary mono‐boronic esters.

We initiated our studies by investigating the arylation of 1,2‐bis‐boronic ester **1** **a** with 1,4‐dicyanobenzene (**4** **a**) (Table 1). We selected (hetero)aryl nitriles as coupling partners because of their established reactivity with alkyl radicals,^[12]^ including in deboronative processes[^11^, ^13^] and EDA complex‐mediated reactions.^[14]^ To promote EDA complex formation, we chose (4‐(dimethylamino)phenyl)lithium (**A**) to activate the primary boronic ester of **1** **a**, with the expectation that the dimethylamino group would enhance the electron donor properties of the resulting arylboronate complex **2** **a**. Pleasingly, blue‐light irradiation of an acetonitrile solution of **2** **a** and **4** **a** at room temperature provided the secondary‐coupled product **3** **aa** in 60 % yield and with excellent regioselectivity (entry 1). The reaction was successful in a range of solvents (entries 2–7), but all gave lower regioselectivities compared to acetonitrile. Increasing the stoichiometry of **2** **a** significantly improved the yield of **3** **aa** (entry 8), and an optimum yield of 90 % was obtained upon performing the reaction at a higher concentration (entry 9). Alternative aryllithium activators **B**–**E** were also tested, but all were less effective than **A** (entries 10–13). Finally, a control reaction showed that light was essential for reactivity (entry 14).^[15]^

**Table 1:** Optimization table.

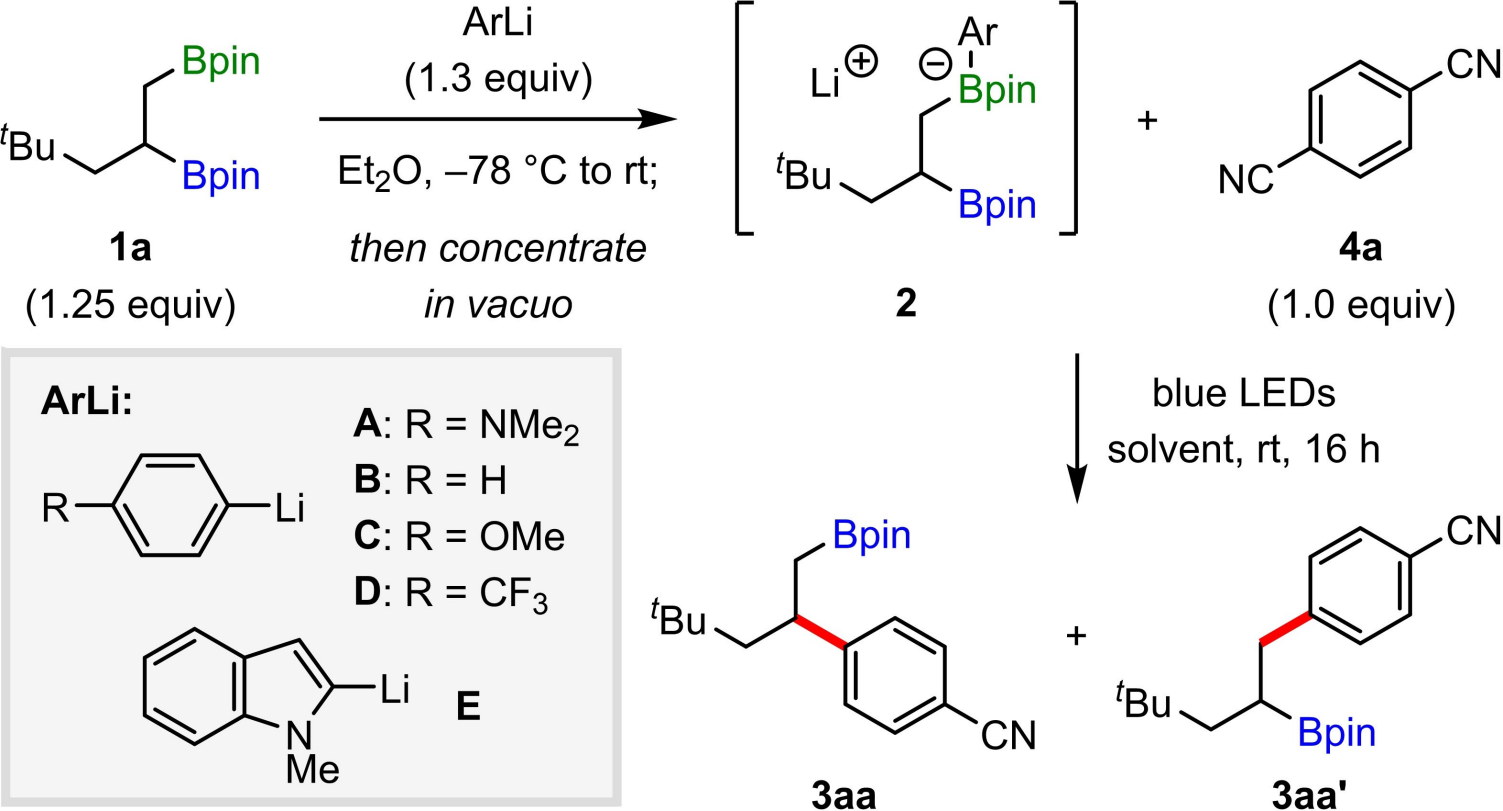				
Entry^[a]^	ArLi	Solvent	**3aa** [%]	**3aa**/**3aa′**
1	**A**	CH_3_CN	60	97/3
2	**A**	THF	64	95/5
3	**A**	DMSO	71	88/12
4	**A**	DMF	74	90/10
5	**A**	DCE	14	68/32
6	**A**	acetone	45	95/5
7	**A**	1,4‐dioxane	30	54/46
8^[b]^	**A**	CH_3_CN	83	98/2
**9** ^[b,c]^	**A**	**CH_3_CN**	**90** (**84**)	**97**/**3**
10^[b,c]^	**B**	CH_3_CN	21	98/2
11^[b,c]^	**C**	CH_3_CN	16	95/5
12^[b,c]^	**D**	CH_3_CN	23	>98/2
13^[b,c]^	**E**	CH_3_CN	74	95/5
14^[b,c,d]^	**A**	CH_3_CN	0	–

[a] Reactions performed using 0.2 mmol of **4** **a** in 2.0 mL of solvent. Yields and regiomeric ratios (r.r.) were determined by GC analysis using 1,3,5‐trimethoxybenzene as the internal standard. Yield of the isolated product is shown in parentheses. [b] Using 1.5 equivalents of **1** **a** and 1.6 equivalents of aryllithium. [c] Using 1.0 mL of CH_3_CN. [d] Reaction performed in the dark.

With the optimized conditions in hand, the scope of this regioselective coupling was evaluated (Scheme 1a). A range of alkyl‐substituted 1,2‐bis‐boronic esters was coupled with **4** **a** in good yields and excellent regioselectivity (**3** **aa**–**3** **ea**). Interestingly, the regioselectivity was relatively insensitive to steric hindrance, with a substrate containing an α‐^*t*^Bu group still reacting with high secondary selectivity (**3** **ea**, 93 : 7). Various functional groups were tolerated, including trimethylsilyl, halide, ester, and aromatic rings (**3** **fa**–**3** **ka**). Coupling of benzylic boronic esters was also possible with complete regioselectivity (**3** **la**–**3** **ma**). It is notable that substrates containing a tertiary boronic ester displayed high reactivity to provide tertiary functionalized products **3** **na**–**3** **pa** with complete regioselectivity.

**Scheme 1:**
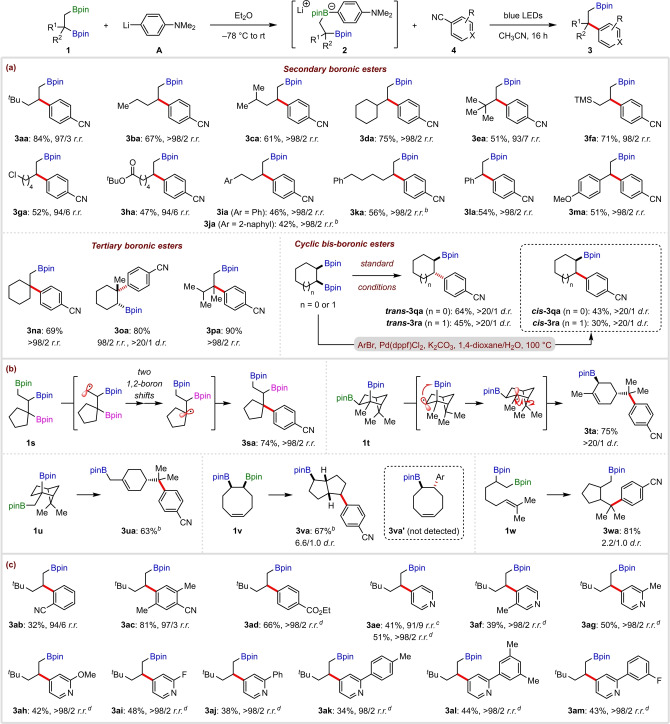
1,2‐Bis‐boronic ester scope.^[a]^ [a] Reactions performed using **1** (0.30 mmol), **A** (0.32 mmol), **4** (0.20 mmol), and CH_3_CN (1.0 mL). Yields are of isolated products. Regioselectivities were determined by GC analysis. [b] Isolated as the corresponding alcohol after oxidation. [c] DMF as solvent. [d] With 4CzIPN (5.0 mol%) and PhLi in place of **A**.

This protocol could be extended to cyclic *cis*‐1,2‐bis‐boronic esters, giving *trans* products **3** **qa** and **3** **ra** with >20 : 1 diastereoselectivity (Scheme 1a). We also examined the reactivity of these cyclic substrates under Pd catalysis, with the expectation that stereoretentive transmetalation would lead to *cis* coupled products. The only reports of such reactions of cyclic bis‐boronic esters are by Morken and Tortosa, who used Pd‐RuPhos‐based catalysts for hydroxy‐directed couplings of cyclopentane and cyclohexane substrates, and non‐directed couplings of strained cyclobutane substrates.[^7a^, ^16^] However, these conditions were ineffective for our substrates, reflecting the known challenges associated with coupling cyclic secondary boronic esters,^[17]^ the low reactivity of which often requires initial conversion to the more reactive trifluoroborates.[^18^, ^19^] Ultimately, we found that using Pd(dppf)_2_Cl_2_ with K_2_CO_3_ in 1,4‐dioxane/H_2_O was successful, giving *cis*‐**3** **qa** and *cis*‐**3** **ra** stereoselectively. These latter examples further highlight the complementarity of the current photocatalyzed process to the Suzuki–Miyaura reaction, providing the option for stereodivergent coupling reactions to access *trans* and *cis* isomers of cyclic *β*‐aryl boronic esters in high selectivity from the same *cis*‐1,2‐bis‐boronic ester. Further extension of the boronic ester scope was demonstrated in a series of radical cascade reactions (Scheme 1b). Coupling of 1,2,3‐tris‐boronic ester **1** **s** provided the tertiary functionalized product **3** **sa** in high regioselectivity after two sequential 1,2‐boron shifts. Arylation of (−)‐*α*‐pinene and (−)‐*β*‐pinene derived substrates **1** **t** and **1** **u** involved 1,2‐boron shifts followed by ring‐opening of the cyclobutane moiety, providing **3** **ta** and **3** **ua** in good yields. The high diastereoselectivity observed for **3** **ta** is indicative of a stereospecific 1,2‐boron shift. Diborated cyclooctadiene **1** **v** underwent transannular cyclization and arylation to give bicyclo[3.3.0]octane **3** **va**, with no non‐cyclized product **3** **va′** observed. Similarly, alkene substrate **1** **w** exclusively gave cyclopentane **3** **wa** in 81 % yield.

The scope of the (hetero)aryl nitrile substrates was subsequently studied (Scheme 1c). 1,2‐Dicyanobenzene gave **3** **ab** in low yield, but the more sterically hindered 2,5‐dimethylbenzene‐1,4‐dicarbonitrile provided **3** **ac** in 81 % yield. Unfortunately, when this catalyst‐free coupling was applied to ethyl 4‐cyanobenzoate (**3** **ad**) and 4‐cyanopyridine (**3** **ae**), low conversions were observed. We found that changing the solvent to DMF dramatically improved the coupling of 4‐cyanopyridine, providing **3** **ae** in 41 % yield, but this modified procedure was unsuccessful for other cyanopyridines. We believe that after PET of the EDA complex generates a radical ion pair, rapid back electron transfer (BET) outcompetes primary alkyl radical formation via C−B bond cleavage.^[20]^ We reasoned that BET could be avoided by using a photoredox catalyst to spatially separate the oxidation of the boronate complex and reduction of the cyanopyridine, thus preventing the formation of the radical ion pair. Pleasingly, using 4CzIPN as the catalyst in combination with phenyllithium to activate the boronic ester,^[21]^ this strategy allowed a range of 4‐cyanopyridines to be successfully coupled in moderate yields and with excellent regioselectivity (**3** **ae**–**3** **am**).

To further explore the generality of the photoinduced catalyst‐free coupling, we applied it to a range of mono‐boronic esters **5** (Scheme 2). Moderate to good yields were obtained for a broad range of primary (**6** **aa**–**6** **ga**) and secondary boronic esters, including carbocyclic (**6** **ha**–**6** **ia**), heterocyclic (**6** **ja**–**6** **ka**), and acyclic substrates (**6** **la**–**6** **oa**). Diastereoselective couplings were possible with boronic esters derived from (−)‐*α*‐pinene (**6** **pa**), norbornene (**6** **qa**), and cholesteryl chloride (**6** **ra**). Despite the increase in steric hindrance, which might have hindered EDA complex formation, tertiary boronic esters were also coupled efficiently (**6** **sa**–**6** **va**). In addition, other (hetero)aryl nitrile substrates reacted in moderate to good yields (**6** **sb**–**6** **hc**). Notably, the coupling of 4‐cyanopyridine with a tertiary boronic ester gave **6** **sc** in excellent yield, which suggests that unproductive BET is not an issue when C−B bond cleavage generates more stabilized tertiary alkyl radicals.

**Scheme 2:**
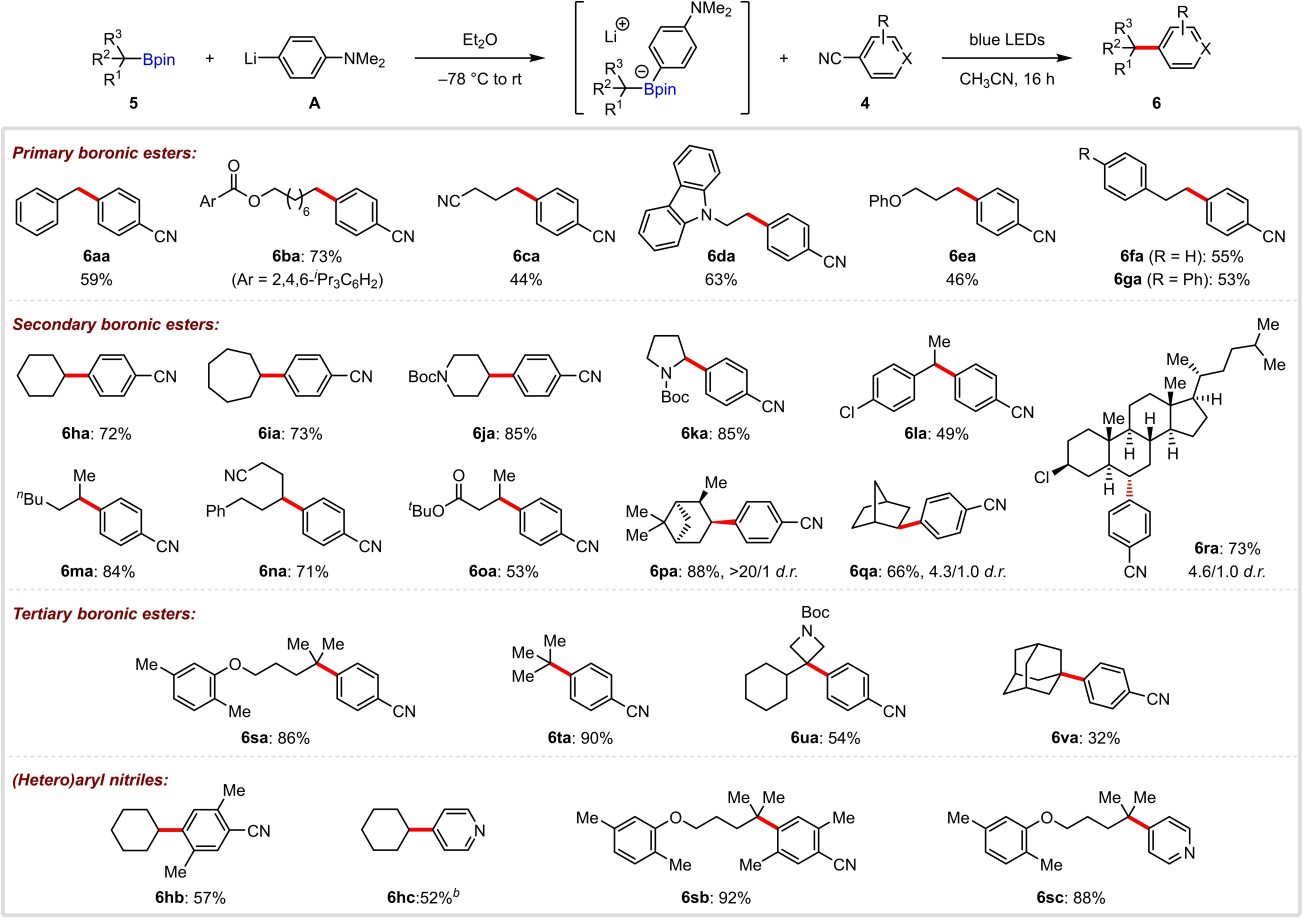
Mono‐boronic ester scope.^[a]^ [a] Reactions performed using **5** (0.30 mmol), **A** (0.32 mmol), **4** (0.20 mmol), and CH_3_CN (1.0 mL). Yields are of isolated products. [b] DMF as solvent.

Finally, we investigated how the EDA complex‐mediated arylation was impacted by the location of the electron donor moiety in the boronate complex; specifically, whether the donor could be incorporated into the diol ligand instead of the aryl substituent. Ohmiya recently demonstrated that boronate complexes bearing highly conjugated polyaromatic diol ligands can undergo direct visible‐light photoexcitation to generate alkyl radicals.^[11]^ We postulated that an EDA complex strategy would obviate the need for such elaborate ligands, therefore we explored the use of catechol boronic esters, which have recently found application as electron donors in EDA complex‐mediated reactions.[^10b^, ^10d^] Pleasingly, boronate complex **8**, formed from catechol boronic ester **7** and *t*‐butyllithium, underwent efficient catalyst‐free couplings with 1,4‐dicyanobenzene and 4‐cyanopyridine to afford **6** **ta** and **6** **tc**, respectively (Scheme 3). The success of these arylation reactions, regardless of the location of the electron donor group on the boronate complex, highlights the flexibility of this EDA complex strategy for photoinduced deboronative alkyl radical formation.

**Scheme 3:**
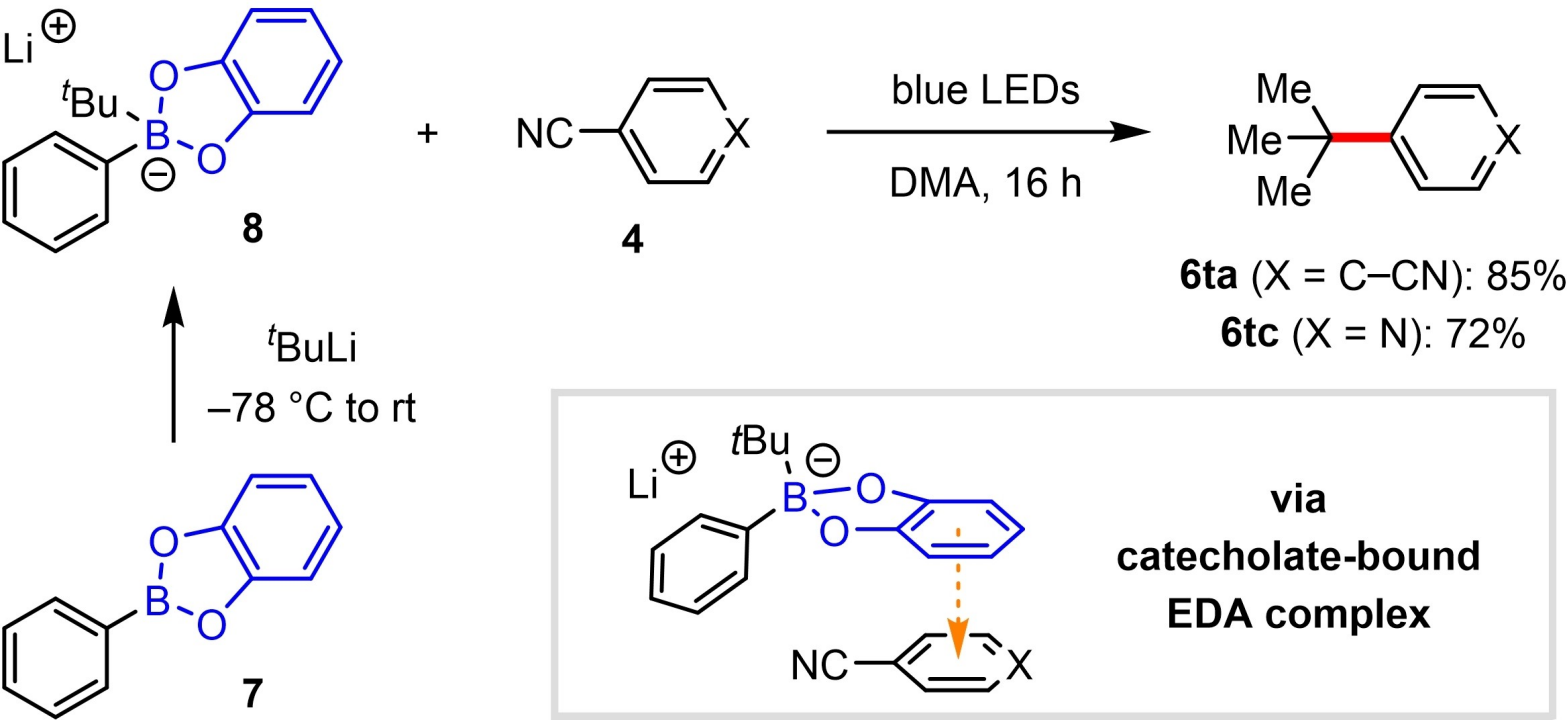
Reactions of catechol boronic esters.

To provide insight into the mechanism of the coupling of 1,2‐bis‐boronic esters, we investigated the regioselectivity of boronate complex formation (Figure 2a). Reaction of **1** **a** with **A** followed by acid hydrolysis gave primary borinic acid **9** in 61 % yield, confirming that boronate complex formation occurs predominantly at the primary boronic ester, although this is of no consequence in the subsequent fragmentation/isomerization of the radical. Evidence for the formation of an EDA complex between arylboronate **2** **a** and 1,4‐dicyanobenzene (**4** **a**) was obtained by UV/Vis absorption spectroscopy, where a bathochromic shift was observed for a 1 : 1 mixture of **2** **a** and **4** **a** (Figure 2b). The formation of a visible‐light absorbing complex was also apparent from the dramatic change in color observed upon mixing solutions of **2** **a** and **4** **a**.^[22]^

**Figure 2:**
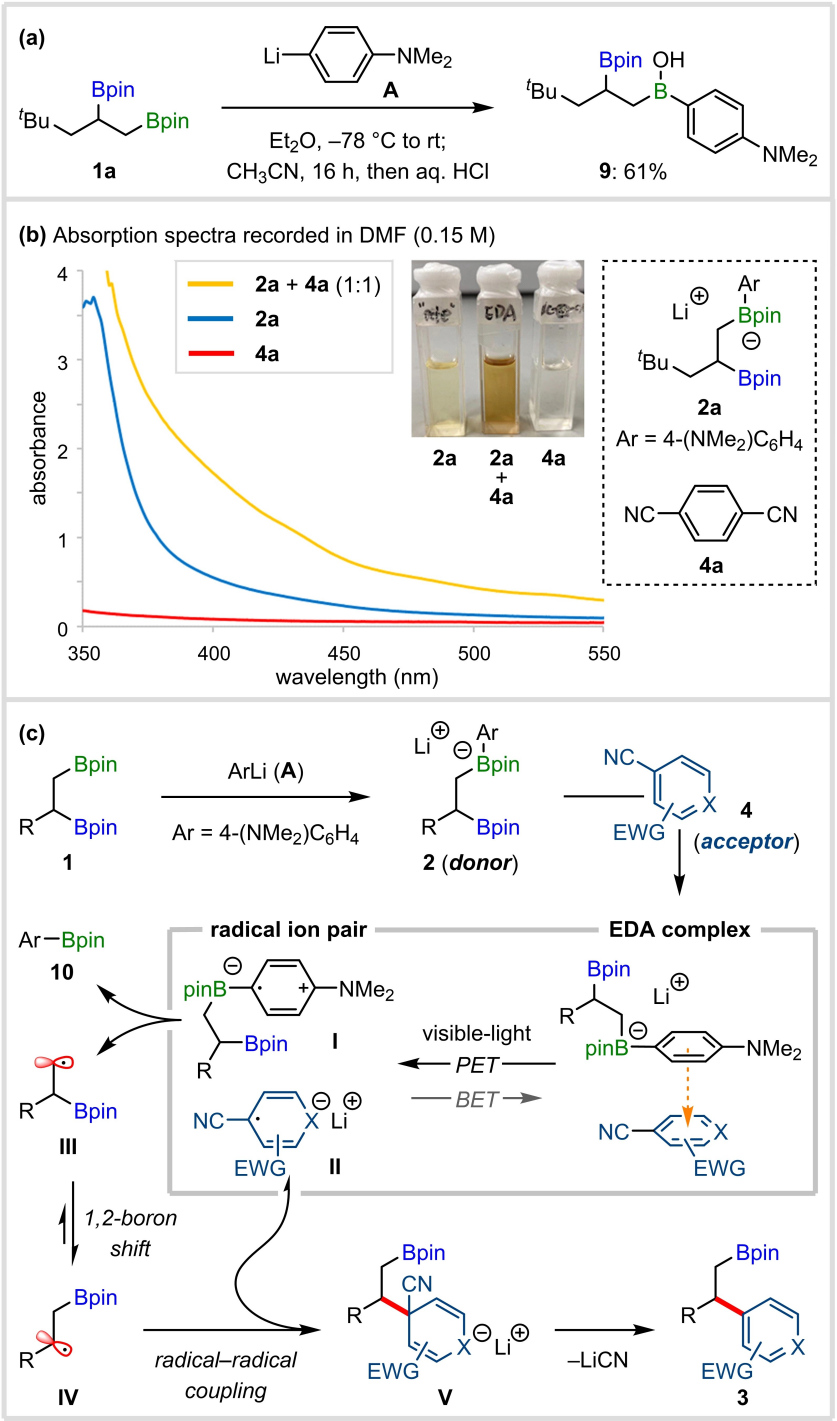
Mechanistic investigations and proposal.

Based on these findings, we propose the mechanism shown in Figure 2c. Regioselective activation of the sterically less hindered primary boronic ester of **1** with **A** generates boronate complex **2**. The presence of the electron‐rich aromatic ring in **2** results in the formation of an EDA complex through interaction with the electron‐deficient aromatic ring of (hetero)aryl nitrile **4**. Visible‐light photoexcitation initiates SET to generate a radical ion pair comprised of boronate complex radical cation **I** and (hetero)aryl radical anion **II**. Homolytic cleavage of the primary C−B bond of **I** forms arylboronic ester **10** and primary *β*‐boryl radical **III**, which undergoes rapid 1,2‐boron shift to the more stable secondary radical **IV**.^[8]^ After coupling of the persistent radical anion **II** and transient radical **IV**,^[23]^ giving anion **V**, elimination of cyanide leads to product **3**.

In conclusion, we have developed a catalyst‐free photoinduced coupling of primary, secondary, and tertiary boronic esters with (hetero)aryl nitriles. This was achieved by initial activation of the boronic ester as an electron‐rich arylboronate complex, which enabled EDA complex formation with electron‐deficient aryl nitriles. Subsequent visible‐light irradiation triggered deboronative alkyl radical formation and radical‐radical coupling to give arylated products. Application to mono‐deboronative couplings of 1,2‐bis‐boronic esters highlighted its complementary selectivity to that of palladium‐catalyzed Suzuki–Miyaura cross‐couplings, including reversal of regioselectivity through arylation of the more substituted boronic ester, and reversal of stereoselectivity through stereoinvertive couplings of cyclic *cis*‐1,2‐bis‐boronic esters.

## Conflict of interest

The authors declare no conflict of interest.

## Supporting information

As a service to our authors and readers, this journal provides supporting information supplied by the authors. Such materials are peer reviewed and may be re‐organized for online delivery, but are not copy‐edited or typeset. Technical support issues arising from supporting information (other than missing files) should be addressed to the authors.

Supporting Information

## Data Availability

The data that support the findings of this study are available in the supplementary material of this article.
