# Supplementary material for: Selective Coupling of 1,2‐Bis‐Boronic Esters at the more Substituted Site through Visible‐Light Activation of Electron Donor–Acceptor Complexes
Source: Angew Chem Int Ed Engl. 2022 Mar 7;61(18):e202202061. doi: 10.1002/anie.202202061 (PMC9314813; doi:10.1002/anie.202202061)
Supplement: Supplementary file 1 — Supporting Information [file ANIE-61-0-s001.pdf]

Angewandte Chemie
Eine Zeitschrift der Gesellschaft Deutscher Chemiker

Supporting Information

Selective Coupling of 1,2-Bis-Boronic Esters at the more Substituted Site through Visible-Light Activation of Electron Donor–Acceptor Complexes

*H. Wang, J. Wu, A. Noble, V. K. Aggarwal**

TABLE OF CONTENTS

LIST OF SUPPLEMENTARY SCHEMES, FIGURES AND TABLES S2

LIST OF CHARACTERISED PRODUCTS S2

MATERIALS AND GENERAL METHODS S1

1.1. Glassware, Solvents and Reagents S1

1.2. Chromatography and Instrumentation S1

1.3. Naming of Compounds S1

2. EXPERIMENTAL DATA S2

2.1 Photochemical Equipment and Setup S2

2.2. Reaction Optimisation S3

2.2.1. Optimisation Studies and Control Reactions S3

2.2.2. Screening of Organolithium Reagents S4

2.2.3. Optimisation Studies for Photoredox-catalysed Coupling S5

2.3. General Procedures S6

2.3.1. General Procedure A: Diboration of Terminal or 1,2-Disubstituted Alkenes S6

2.3.2. General Procedure B: Diboration of 1,1-Disubstituted or Trisubstituted Alkenes S6

2.3.3. General Procedure C: Synthesis of 2-Aryl-4-cyanopyridines from 4-Cyanopyridine S7

2.3.4. General Procedure D: For Reactions of Boronic Esters 1a-1w and **5a-5v** with **4a-4d** S7

2.3.5. General Procedure E: For Reactions of Bis-boronic Ester 1a with 4-Cyanopyridines **4e-4m** S8

2.4. Synthesis and Purchasing of Starting Materials S9

2.5. Product Characterisation S32

2.6. Mechanistic Studies S73

2.6.1. Regioselectivity of Boronate Complex Formation S73

2.6.2. UV/Vis Absorption Spectroscopy S75

2.6.3. Quantum Yield Measurement S80

2.6.4. EDA Complex-Mediated Reactions of Catechol Boronic Esters S84

2.6.5. Proposed Mechanism for the Photoredox-Catalysed Reaction S86

2.6.6. Further Support for Photoexcitation of EDA Complexes S87

2.7. Structure Elucidation of **3ua** S88

3. SPECTROSCOPIC DATA S94

4. REFERENCES S226

UOB Confidential & Sensitive

LIST OF SUPPLEMENTARY SCHEMES, FIGURES AND TABLES

Figure S1. Photochemical Reaction Setup. S2
Scheme S1: Synthesis of [4-(Dimethylamino)phenyl]lithium Reagent. S3
Table S1: Optimisation Studies for the Selective Cross-Coupling Reaction. S3
Scheme S2: Screening of Organolithium Reagents. S4
Table S2: Optimisation Studies for Photoredox-catalysed Coupling. S5
Figure S2. Initial Reaction Setup. S8
Scheme S3. Commercially Available Starting Materials. S9
Scheme S4. Previously Reported Boronic Ester Substrates. S10
Scheme S5. Synthesis of Primary Borinic Acid **9**. S73
Scheme S6. Synthesis of Boronate Complex **2a**. S75
Figure S3. UV/Vis Absorption Spectra of **2a** and **4a** at (a) 0.05 M and (b) 0.15 M. S76
Scheme S7. Synthesis of Boronate Complex **2h**. S77
Figure S4. UV/Vis Absorption Spectra of **2h** and **4a**. S78
Scheme S8. Synthesis of Boronate Complex **8**. S78
Figure S5. UV/Vis Absorption Spectra of **8** and **4a**. S79
Figure S6: Actinometry: UV/Vis Spectra of Ferrioxalate/1,10-Phenanthroline Solutions. S80
Figure S7: Actinometry: Moles of Fe^{2+} Formed vs. Irradiation Time. S81
Figure S8: Moles of 3aa Formed vs. Irradiation Time. S83
Scheme S9. Synthesis of PhenylBcat **7**. S84
Scheme S10. Photoinduced Coupling Reaction with PhenylBcat. S85
Scheme S11. Proposed Mechanism for the Photoredox-Catalysed Reaction. S86
Scheme S12. Attempted Photoinduced Giese Reactions with Benzyl Acrylate. S87
Scheme S13. Photoinduced Giese Reactions with Dimethyl Fumarate. S87
Table S3: Comparison Between Calculated NOE Distances and Experiment for Configuration A. S90
Table S4: Comparison Between Calculated NOE Distances and Experiment for Configuration B. S91
Table S5: Comparison Between Calculated NOE Distances and Experiment for Configuration C. S92
Table S6: Comparison Between Calculated NOE Distances and Experiment for Configuration D. S93

LIST OF CHARACTERISED PRODUCTS

2,2'-(4,4-Dimethylpentane-1,2-diyl)bis(4,4,5,5-tetramethyl-1,3,2-dioxaborolane) (**1a**) S11
2,2'-(Pentane-1,2-diyl)bis(4,4,5,5-tetramethyl-1,3,2-dioxaborolane) (**1b**) S11
2,2'-(4-Methylpentane-1,2-diyl)bis(4,4,5,5-tetramethyl-1,3,2-dioxaborolane) (**1c**) S12
2,2'-(1-Cyclohexylethane-1,2-diyl)bis(4,4,5,5-tetramethyl-1,3,2-dioxaborolane) (**1d**) S12
2,2'-(3,3-Dimethylbutane-1,2-diyl)bis(4,4,5,5-tetramethyl-1,3,2-dioxaborolane) (**1e**) S13
[2,3-Bis(4,4,5,5-tetramethyl-1,3,2-dioxaborolan-2-yl)propyl]trimethylsilane (**1f**) S13
2,2'-(6-Chlorohexane-1,2-diyl)bis(4,4,5,5-tetramethyl-1,3,2-dioxaborolane) (**1g**) S14
tert-Butyl 6,7-bis(4,4,5,5-tetramethyl-1,3,2-dioxaborolan-2-yl)heptanoate (**1h**) S15
2,2'-(4-Phenylbutane-1,2-diyl)bis(4,4,5,5-tetramethyl-1,3,2-dioxaborolane) (**1i**) S15
2,2'-[4-(Naphthalen-2-yl)butane-1,2-diyl]bis(4,4,5,5-tetramethyl-1,3,2-dioxaborolane) (**1j**) S16
2,2'-(6-Phenylhexane-1,2-diyl)bis(4,4,5,5-tetramethyl-1,3,2-dioxaborolane) (**1k**) S16
2,2'-(1-Phenylethane-1,2-diyl)bis(4,4,5,5-tetramethyl-1,3,2-dioxaborolane) (**1l**) S17
2,2'-[1-(4-Methoxyphenyl)ethane-1,2-diyl]bis(4,4,5,5-tetramethyl-1,3,2-dioxaborolane) (**1m**) S18

4,4,5,5-Tetramethyl-2-{[1-(4,4,5,5-tetramethyl-1,3,2-dioxaborolan-2-yl)cyclohexyl]methyl}-1,3,2-dioxaborolane (**1n**) S18
2,2'-[(*cis*)-1-Methylcyclohexane-1,2-diyl]bis(4,4,5,5-tetramethyl-1,3,2-dioxaborolane) (**1o**) S19
2,2'-(2,3-Dimethylbutane-1,2-diyl)bis(4,4,5,5-tetramethyl-1,3,2-dioxaborolane) (**1p**) S20
(*cis*)-1,2-Bis(4,4,5,5-tetramethyl-1,3,2-dioxaborolan-2-yl)cyclopentane (**1q**) S20
(*cis*)-1,2-Bis(4,4,5,5-tetramethyl-1,3,2-dioxaborolan-2-yl)cyclohexane (**1r**) S21
2,2'-[(2*R*)-2,6,6-Trimethylbicyclo[3.1.1]heptane-2,3-diyl]bis(4,4,5,5-tetramethyl-1,3,2-dioxaborolane) (**1t**) ... S21
2-{(2*R*)-6,6-Dimethyl-2-[(4,4,5,5-tetramethyl-1,3,2-dioxaborolan-2-yl)methyl]bicyclo[3.1.1]heptan-2-yl}-4,4,5,5-tetramethyl-1,3,2-dioxaborolane (**1u**) S22
(*cis,Z*)-5,6-Bis(4,4,5,5-tetramethyl-1,3,2-dioxaborolan-2-yl)cyclooct-1-ene (**1v**) S23
2,2'-(7-Methyloct-6-ene-1,2-diyl)bis(4,4,5,5-tetramethyl-1,3,2-dioxaborolane) (**1w**) S23
2,5-Dimethylterephthalonitrile (**4c**) S24
2-Methylisonicotinonitrile (**4g**) S24
2-Methoxyisonicotinonitrile (**4h**) S25
2-Phenylisonicotinonitrile (**4j**) S26
2-(*p*-Tolyl)isonicotinonitrile (**4k**) S26
2-(3,5-Dimethylphenyl)isonicotinonitrile (**4l**) S27
2-(3-Fluorophenyl)isonicotinonitrile (**4m**) S27
8-(4,4,5,5-Tetramethyl-1,3,2-dioxaborolan-2-yl)octyl 2,4,6-triisopropylbenzoate (**5b**) S28
4,4,5,5-Tetramethyl-2-(3-phenoxypropyl)-1,3,2-dioxaborolane (**5e**) S29
tert-Butyl 3-(4,4,5,5-tetramethyl-1,3,2-dioxaborolan-2-yl)butanoate (**5o**) S29
exo-2-(Bicyclo[2.2.1]heptan-2-yl)-4,4,5,5-tetramethyl-1,3,2-dioxaborolane (**5q**) S30
2-{(3*S*,5*R*,6*R*,8*R*,9*S*,10*S*,13*R*,14*S*,17*R*)-3-Chloro-10,13-dimethyl-17-[(*R*)-5-methylhexan-2-yl]hexadecahydro-1*H*-cyclopenta[a]phenanthren-6-yl}-4,4,5,5-tetramethyl-1,3,2-dioxaborolane (**5r**) S31
4-[4,4-Dimethyl-1-(4,4,5,5-tetramethyl-1,3,2-dioxaborolan-2-yl)pentan-2-yl]benzonitrile (**3aa**) S32
4-[1-(4,4,5,5-Tetramethyl-1,3,2-dioxaborolan-2-yl)pentan-2-yl]benzonitrile (**3ba**) S32
4-[4-Methyl-1-(4,4,5,5-tetramethyl-1,3,2-dioxaborolan-2-yl)pentan-2-yl]benzonitrile (**3ca**) S33
4-[1-Cyclohexyl-2-(4,4,5,5-tetramethyl-1,3,2-dioxaborolan-2-yl)ethyl]benzonitrile (**3da**) S34
4-[3,3-Dimethyl-1-(4,4,5,5-tetramethyl-1,3,2-dioxaborolan-2-yl)butan-2-yl]benzonitrile (**3ea**) S34
4-[1-(4,4,5,5-Tetramethyl-1,3,2-dioxaborolan-2-yl)-3-(trimethylsilyl)propan-2-yl]benzonitrile (**3fa**) S35
4-[6-Chloro-1-(4,4,5,5-tetramethyl-1,3,2-dioxaborolan-2-yl)hexan-2-yl]benzonitrile (**3ga**) S35
tert-Butyl 6-(4-cyanophenyl)-7-(4,4,5,5-tetramethyl-1,3,2-dioxaborolan-2-yl)heptanoate (**3ha**) S36
4-[4-Phenyl-1-(4,4,5,5-tetramethyl-1,3,2-dioxaborolan-2-yl)butan-2-yl]benzonitrile (**3ia**) S37
4-[1-Hydroxy-4-(naphthalen-2-yl)butan-2-yl]benzonitrile (**3ja**) S37
4-(1-Hydroxy-6-phenylhexan-2-yl)benzonitrile (**3ka**) S38
4-[1-Phenyl-2-(4,4,5,5-tetramethyl-1,3,2-dioxaborolan-2-yl)ethyl]benzonitrile (**3la**) S39
4-[1-(4-Methoxyphenyl)-2-(4,4,5,5-tetramethyl-1,3,2-dioxaborolan-2-yl)ethyl]benzonitrile (**3ma**) S39
4-{1-[(4,4,5,5-Tetramethyl-1,3,2-dioxaborolan-2-yl)methyl]cyclohexyl}benzonitrile (**3na**) S40
4-[1-Methyl-2-(4,4,5,5-tetramethyl-1,3,2-dioxaborolan-2-yl)cyclohexyl]benzonitrile (**3oa**) S41
4-[2,3-Dimethyl-1-(4,4,5,5-tetramethyl-1,3,2-dioxaborolan-2-yl)butan-2-yl]benzonitrile (**3pa**) S41
4-[(*trans*)-2-(4,4,5,5-Tetramethyl-1,3,2-dioxaborolan-2-yl)cyclopentyl]benzonitrile (**(*trans*)-3qa**) S42
4-[(*cis*)-2-(4,4,5,5-Tetramethyl-1,3,2-dioxaborolan-2-yl)cyclopentyl]benzonitrile (**(*cis*)-3qa**) S43
4-[(*trans*)-2-(4,4,5,5-Tetramethyl-1,3,2-dioxaborolan-2-yl)cyclohexyl]benzonitrile (**(*trans*)-3ra**) S43
4-[(*cis*)-2-(4,4,5,5-Tetramethyl-1,3,2-dioxaborolan-2-yl)cyclohexyl]benzonitrile (**(*cis*)-3ra**) S44
4-{1-[1,2-Bis(4,4,5,5-tetramethyl-1,3,2-dioxaborolan-2-yl)ethyl]cyclopentyl}benzonitrile (**3sa**) S45
4-{2-[(1*S*,5*S*)-4-Methyl-5-(4,4,5,5-tetramethyl-1,3,2-dioxaborolan-2-yl)cyclohex-3-en-1-yl]propan-2-

yl}benzonitrile (**3ta**) S45
(*R*)-4-{2-[4-(Hydroxymethyl)cyclohex-3-en-1-yl]propan-2-yl}benzonitrile (**3ua**) S46
4-[(1*R**,3a*R**,4*R**,6a*S**)-4-Hydroxyoctahydropentalen-1-yl]benzonitrile (**3va**) S47
4-{2-{2-[(4,4,5,5-Tetramethyl-1,3,2-dioxaborolan-2-yl)methyl]cyclopentyl}propan-2-yl}benzonitrile (**3wa**) S47
2-[4,4-Dimethyl-1-(4,4,5,5-tetramethyl-1,3,2-dioxaborolan-2-yl)pentan-2-yl]benzonitrile (**3ab**) S48
4-[4,4-Dimethyl-1-(4,4,5,5-tetramethyl-1,3,2-dioxaborolan-2-yl)pentan-2-yl]-2,5-dimethylbenzonitrile (**3ac**) S49
Ethyl 4-[4,4-dimethyl-1-(4,4,5,5-tetramethyl-1,3,2-dioxaborolan-2-yl)pentan-2-yl]benzoate (**3ad**) S49
4-[4,4-Dimethyl-1-(4,4,5,5-tetramethyl-1,3,2-dioxaborolan-2-yl)pentan-2-yl]pyridine (**3ae**) S50
4-[4,4-Dimethyl-1-(4,4,5,5-tetramethyl-1,3,2-dioxaborolan-2-yl)pentan-2-yl]-3-methylpyridine (**3af**) S51
4-[4,4-Dimethyl-1-(4,4,5,5-tetramethyl-1,3,2-dioxaborolan-2-yl)pentan-2-yl]-2-methylpyridine (**3ag**) S52
4-[4,4-Dimethyl-1-(4,4,5,5-tetramethyl-1,3,2-dioxaborolan-2-yl)pentan-2-yl]-2-methoxypyridine (**3ah**) S52
4-[4,4-Dimethyl-1-(4,4,5,5-tetramethyl-1,3,2-dioxaborolan-2-yl)pentan-2-yl]-2-fluoropyridine (**3ai**) S53
4-[4,4-Dimethyl-1-(4,4,5,5-tetramethyl-1,3,2-dioxaborolan-2-yl)pentan-2-yl]-2-phenylpyridine (**3aj**) S54
4-[4,4-Dimethyl-1-(4,4,5,5-tetramethyl-1,3,2-dioxaborolan-2-yl)pentan-2-yl]-2-(*p*-tolyl)pyridine (**3ak**) S54
4-[4,4-Dimethyl-1-(4,4,5,5-tetramethyl-1,3,2-dioxaborolan-2-yl)pentan-2-yl]-2-(3,5-dimethylphenyl)pyridine (**3al**) S55
4-[4,4-Dimethyl-1-(4,4,5,5-tetramethyl-1,3,2-dioxaborolan-2-yl)pentan-2-yl]-2-(3-fluorophenyl)pyridine (**3am**) S56
4-Benzylbenzonitrile (**6aa**) S57
8-(4-Cyanophenyl)octyl 2,4,6-triisopropylbenzoate (**6ba**) S57
4-(3-Cyanopropyl)benzonitrile (**6ca**) S58
4-[2-(9*H*-Carbazol-9-yl)ethyl]benzonitrile (**6da**) S58
4-(3-Phenoxypropyl)benzonitrile (**6ea**) S59
4-Phenethylbenzonitrile (**6fa**) S59
4-{2-([1,1'-Biphenyl]-4-yl)ethyl}benzonitrile (**6ga**) S60
4-Cyclohexylbenzonitrile (**6ha**) S61
4-Cycloheptylbenzonitrile (**6ia**) S61
tert-Butyl 4-(4-cyanophenyl)piperidine-1-carboxylate (**6ja**) S62
tert-Butyl 2-(4-cyanophenyl)pyrrolidine-1-carboxylate (**6ka**) S62
4-[1-(4-Chlorophenyl)ethyl]benzonitrile (**6la**) S63
4-(Heptan-2-yl)benzonitrile (**6ma**) S63
4-(1-Cyano-5-phenylpentan-3-yl)benzonitrile (**6na**) S64
tert-Butyl 3-(4-cyanophenyl)butanoate (**6oa**) S65
4-[(1*R*,2*R*,3*R*,5*R*)-2,6,6-Trimethylbicyclo[3.1.1]heptan-3-yl]benzonitrile (**6pa**) S65
4-[(1*R**,2*R**,4*S**)-Bicyclo[2.2.1]heptan-2-yl]benzonitrile (**6qa**) S66
4-{(3*S*,5*R*,6*S*,8*S*,10*R*,13*R*,14*S*,17*R*)-3-Chloro-10,13-dimethyl-17-[(*S*)-6-methylheptan-2-yl]hexadecahydro-1*H*-cyclopenta[a]phenanthren-6-yl}benzonitrile (**6ra**) S67
4-[5-(2,5-Dimethylphenoxy)-2-methylpentan-2-yl]benzonitrile (**6sa**) S67
4-(*tert*-Butyl)benzonitrile (**6ta**) S68
tert-Butyl 3-(4-cyanophenyl)-3-cyclohexylazetidine-1-carboxylate (**6ua**) S69
4-[(3*r*,5*r*,7*r*)-Adamantan-1-yl]benzonitrile (**6va**) S69
4-Cyclohexyl-2,5-dimethylbenzonitrile (**6hb**) S70
4-Cyclohexylpyridine (**6hc**) S70
4-[5-(2,5-Dimethylphenoxy)-2-methylpentan-2-yl]-2,5-dimethylbenzonitrile (**6sb**) S71
4-[5-(2,5-Dimethylphenoxy)-2-methylpentan-2-yl]pyridine (**6sc**) S72
4-{[4,4-Dimethyl-2-(4,4,5,5-tetramethyl-1,3,2-dioxaborolan-2-yl)pentyl](hydroxy)boraneyl}-*N*,*N*-dimethylaniline (**9**) S74

2-Phenylbenzo[*d*][1,3,2]dioxaborole (**7**) S84
4-(*tert*-Butyl)pyridine (**6tc**) S85

UOB Confidential & Sensitive

MATERIALS AND GENERAL METHODS

1.1. Glassware, Solvents and Reagents

All manipulations were performed with oven-dried (130 °C for a minimum of 12 h) or flame-dried glassware using standard Schlenk techniques under an atmosphere of nitrogen, unless otherwise stated.

All anhydrous solvents were commercially supplied or dried using an Anhydrous Engineering alumina column drying system (THF, toluene, Et_2O, CH_2Cl_2, and CH_3CN). Reagents were purchased from commercial sources and used as received. All organolithium reagents were titrated against *N*-benzylbenzamide.[1]

1.2. Chromatography and Instrumentation

Thin layer chromatography (TLC) was performed using Merck Kieselgel 60 F254 fluorescent treated silica, which was visualised under UV light, or by staining with aqueous basic potassium permanganate followed by heating, *p*-anisaldehyde solution followed by heating, Hanessian's stain (CAM stain) followed by heating, or an ethanolic solution of phosphomolybdic acid followed by heating, as stated.

Flash column chromatography (FCC) was carried out using Sigma-Aldrich silica gel (60 Å, 230-400 mesh, 40-63 µm) or a Biotage Isolera™ flash purification system. In cases where automated column chromatography was employed the solvent gradient and flow rate are indicated.

NMR spectra were recorded at various field strengths, as indicated, using Bruker 400 MHz, Varian VNMR 400 MHz, Varian VNMR 500 MHz, or Bruker Cryo 500 MHz for ^{1}H, ^{11}B, ^{13}C and ^{19}F acquisitions. All NMR spectra were recorder at 25 °C unless otherwise stated. Chemical shifts (δ) are reported in parts per million (ppm) and referenced to $CDCl_3$ (^{1}H: 7.26 ppm; ^{13}C: 77.16 ppm). Coupling constants (*J*) are given in Hertz (Hz) and refer to apparent multiplicities (s = singlet, d = doublet, t = triplet, q = quartet, quin = quintet, hex = hextet, h = heptet, m = multiplet, br s = broad signal, dd = doublet of doublets, etc.). The ^{1}H NMR spectra are reported as follows: chemical shift (multiplicity, coupling constants, number of protons).

High resolution mass spectra (HRMS) were recorded on a Bruker Daltonics MicrOTOF II by Electrospray Ionisation (ESI); a Thermo Scientific QExactive by Electron Ionisation (EI); a Thermo Scientific Orbitrap Elite by ESI or Atmospheric Pressure Chemical Ionisation (APCI); or a Bruker UltrafleXtreme by Matrix-assisted Laser Desorption/Ionisation (MALDI).

IR spectra were recorded neat as a thin film on a Perkin Elmer Spectrum One FT-IR. Selected absorption maxima (ν_{max}) are reported in wavenumbers (cm^{-1}).

Gas chromatography–mass spectrometry (**GC-MS**) was recorded on an Agilent 6890 Series GC and 5973 detector using a HP-5MS UI column (15 m x 0.25 mm x 0.25 µm).

1.3. Naming of Compounds

Compound names are those generated by ChemDraw Professional 20.0 software (PerkinElmer), following the IUPAC nomenclature.

UOB Confidential & Sensitive

2. EXPERIMENTAL DATA

2.1 Photochemical Equipment and Setup

The photochemical reactions were carried out in the photochemical reactor (PhotoCube^{TM}, manufactured by ThalesNano) which the LED panels (blue light, λ_{max} = 457 nm) are on the 4 sides (the intensity knob is set to 70% and the current selector is set to Hi mode) (Figure S1).

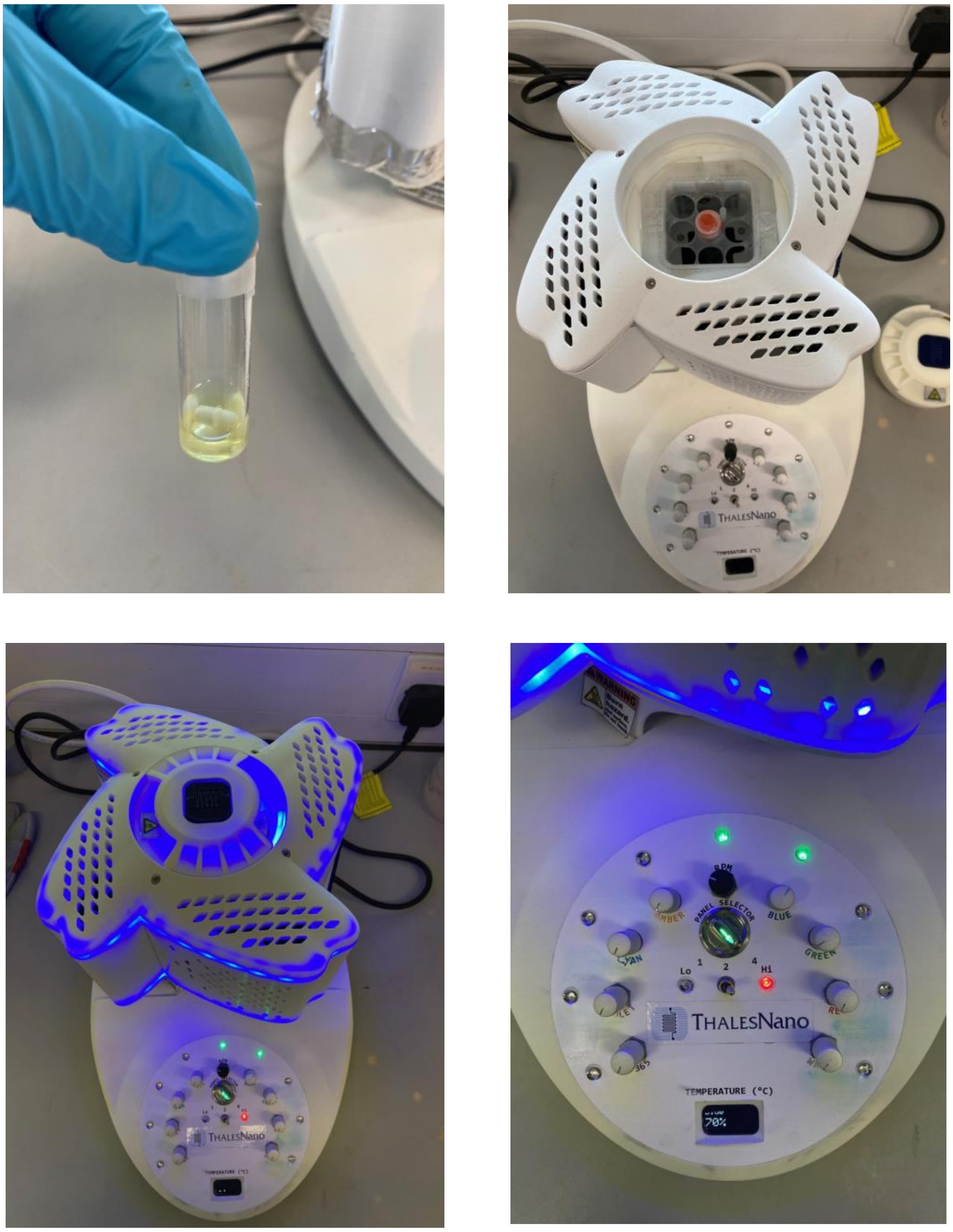

Figure S1. Photochemical Reaction Setup.

2.2. Reaction Optimisation

2.2.1. Optimisation Studies and Control Reactions

[4-(Dimethylamino)phenyl]lithium is made from 4-bromo-*N*,*N*-dimethylaniline (1.0 equiv.) with *t*-BuLi (2.0 equiv., 1.7 M in pentane) (**Scheme S1**)

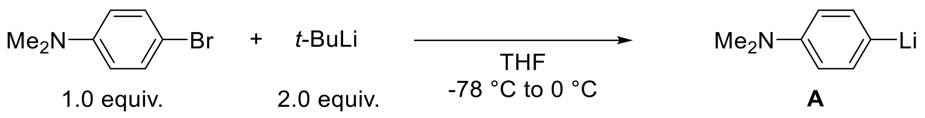

Scheme S1: Synthesis of [4-(Dimethylamino)phenyl]lithium Reagent.

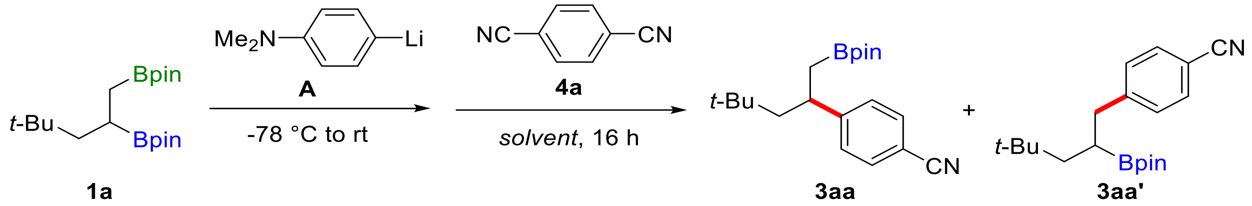

Entry	Equiv. (1a)	Solvent	Conc. (M) /4a	Yield (3aa)[a]	*r.r.* (3aa/3aa')[b]
1	1.25	THF	0.1	64%	95/5
2	1.25	CH_3CN	0.1	60%	97/3
3	1.25	DMSO	0.1	71%	88/12
4	1.25	Acetone	0.1	45%	95/5
5	1.25	DCE	0.1	14%	68/32
6	1.25	NMP	0.1	28%	78/22
7	1.25	DMF	0.1	74%	90/10
8	1.25	1,4-dioxane	0.1	30%	54/46
9	1.25	EtOAc	0.1	56%	89/11
10	1.5	CH_3CN	0.1	83%	98/2
11	2.0	CH_3CN	0.1	86%	98/2
**12**	**1.5**	**CH_3CN**	**0.2**	**90% (84%)[c]**	**97/3**
13	1.5	CH_3CN	0.2	0%[d]	--
14	1.5	THF	0.1	82%	95/5
15	1.5	DME(glyme)	0.2	69%	92/8
16	1.5	diglyme	0.2	47%	85/15
17	1.5	1,4-dioxane	0.2	29%	49/51

Table S1: Optimisation Studies for the Selective Cross-Coupling Reaction.

All reactions were performed on a 0.2 mmol scale of **4a** under N_2 in photochemical reactor (PhotoCube™) for 16 hours; [a] The yields were determined by GC analysis using 1,3,5-trimethoxybenzene as the internal standard; [b] The regioselectivity were determined by GC-Fid analysis; [c] Isolated yield; [d] Reaction performed in the dark.

2.2.2. Screening of Organolithium Reagents

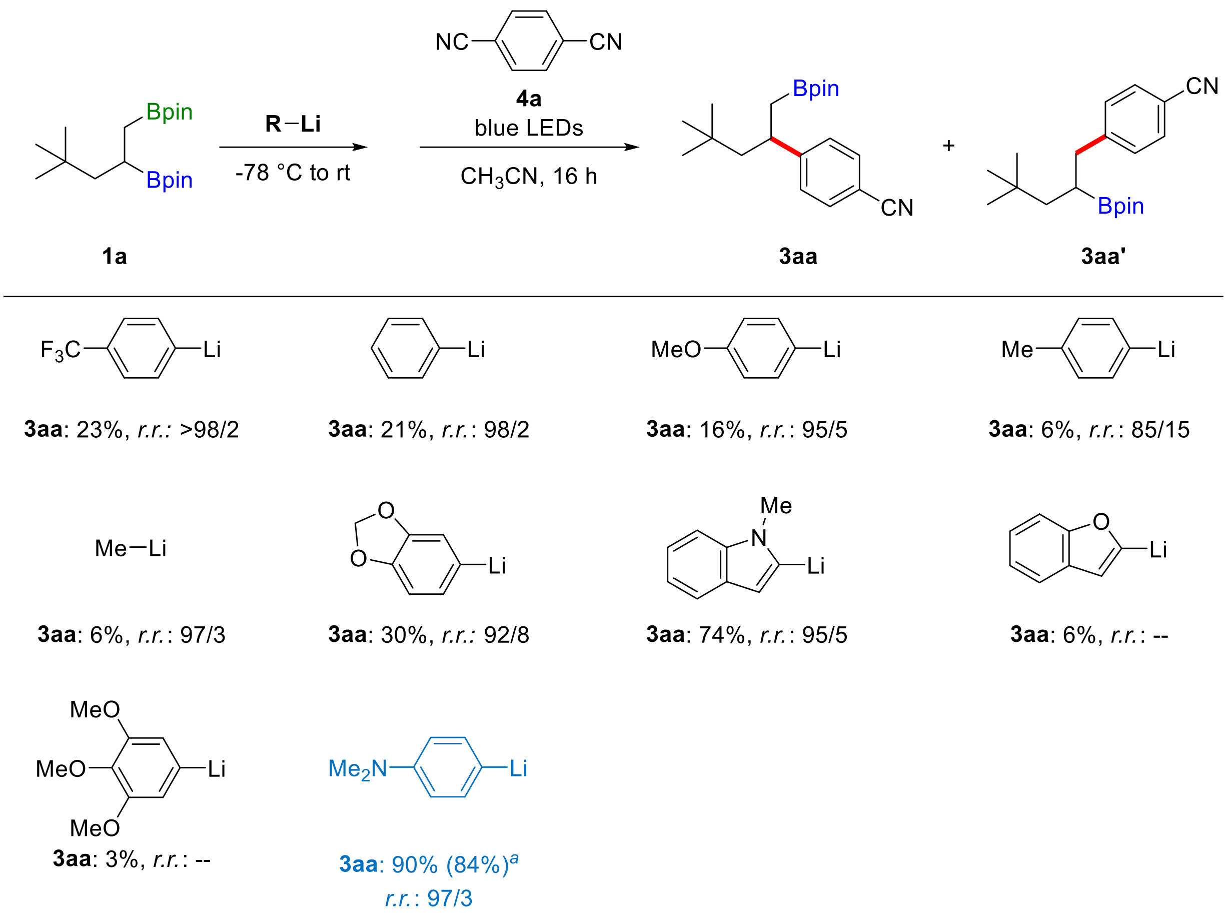

Scheme S2: Screening of Organolithium Reagents.

Reaction conditions: **1a** (0.3 mmol, 1.5 equiv.), **4a** (0.2 mmol, 1.0 equiv.), blue LEDs (PhotoCube™), CH_3CN (1.0 mL), N_2, 16 h. Yields and regioselectivity were determined by GC analysis using 1,3,5-trimethoxybenzene as the internal standard. [a] Isolated yield.

2.2.3. Optimisation Studies for Photoredox-catalysed Coupling

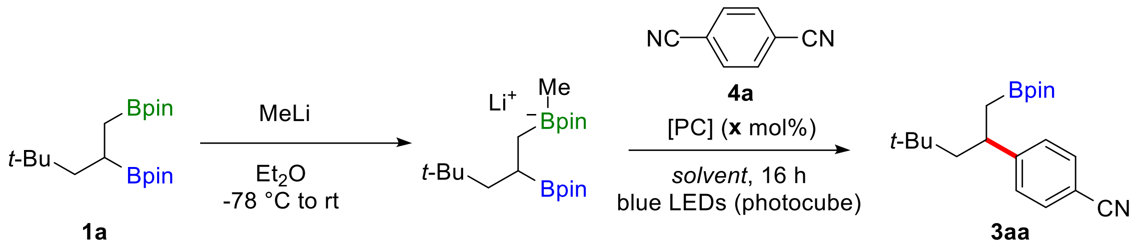

Entry	Catalyst / x mol%	Solvent	Yield[a]	*r.r.* (3aa/3aa')[b]
1	$Ir(ppy)_3$/2.5	CH_3CN	56%	7/1
2	4CzIPN/1.0	CH_3CN	13%	> 20/1
3	4CzIPN/5.0	CH_3CN	56%	> 20/1
4	4-Cl-CzIPN/5.0	CH_3CN	41%	> 20/1
5	$[Ru(bpy)_3](SbF_6)_2$/5.0	CH_3CN	--	--
6	4CzIPN/10.0	CH_3CN	72%	> 20/1
**7**	**4CzIPN/5.0**	**CH_3CN**	**89% (82%)[c]**	**> 20/1**
8	4CzIPN/5.0	DMA	~81%	~ 4.4/1
9	4CzIPN/5.0	THF	80%	> 20/1

Table S2: Optimisation Studies for Photoredox-catalysed Coupling.

Reaction conditions: **1a** (0.3 mmol, 1.5 equiv.), **4a** (0.2 mmol, 1.0 equiv.), MeLi (0.32 mmol, 1.6 equiv., 1.6 M in diethyl ether), blue LEDs (PhotoCube™), N_2, 16 h. [a, b] Yields and regioselectivity were determined by crude ^1H NMR using 1,3,5-trimethoxybenzene as the internal standard; [c] Isolated yield.

2.3. General Procedures

2.3.1. General Procedure A: Diboration of Terminal or 1,2-Disubstituted Alkenes

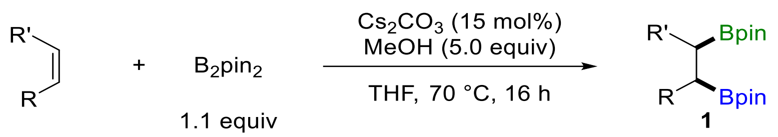

Following a modified literature procedure:[2]

To a flame-dried Schlenk flask were added B_2pin_2 (1.1 equiv.) and Cs_2CO_3 (15.0 mol%), after which the flask was evacuated and back-filled with nitrogen three times. Subsequently, anhydrous THF (0.25 M with respect to the alkene substrate), anhydrous methanol (5.0 equiv.) and the corresponding alkene (1.0 equiv.) were added sequentially *via* syringe. The resulting suspension was heated to 70 °C for 16 h, after which the reaction mixture was filtered through a plug of silica (eluting with diethyl ether). The filtrate was concentrated under reduced pressure and the resulting crude material was purified by flash column chromatography (pentane/diethyl ether) to give the bis-boronic ester product **1**.

2.3.2. General Procedure B: Diboration of 1,1-Disubstituted or Trisubstituted Alkenes

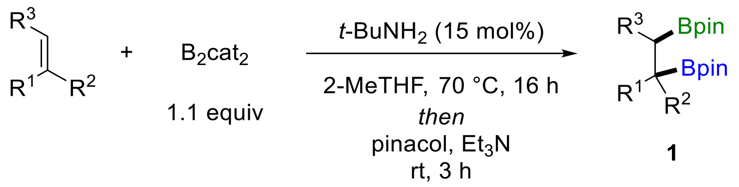

Following a modified literature procedure:[3]

To a flame-dried Schlenk flask was added B_2cat_2 (1.1 equiv.), after which the flask was evacuated and back-filled with nitrogen three times. Subsequently, anhydrous 2-MeTHF (0.25 M with respect to the alkene substrate), distilled *t*-butylamine (10 mol%) and the corresponding alkene (1.0 equiv.) were added sequentially *via* syringe. The resulting solution was heated to 70 °C for 16 hours and subsequently allowed to cool to ambient temperature. Then, a preformed solution of pinacol (6.0 equiv.) in triethylamine (0.4 M with respect to the alkene substrate) was added to the reaction mixture and the resulting solution was vigorously stirred at ambient temperature for another 3 hours. After the reaction, the mixture was directly concentrated under reduced pressure and the resulting crude material was purified by flash column chromatography (pentane/diethyl ether) to afford the bis-boronic ester product **1**.

UOB Confidential & Sensitive

2.3.3. General Procedure C: Synthesis of 2-Aryl-4-cyanopyridines from 4-Cyanopyridine

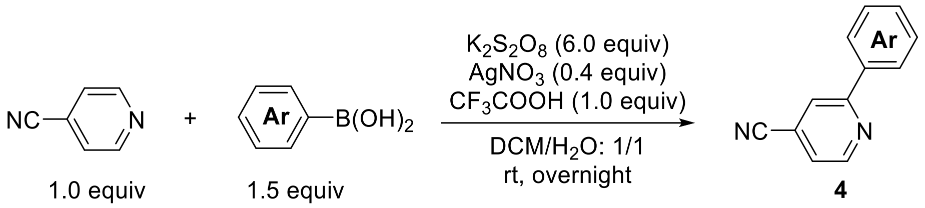

Following a modified literature procedure:[4]

To a solution of 4-cyanopyridine (1.0 equiv.) in DCM (0.25 M with respect to 4-cyanopyridine) was added trifluoroacetic acid (1.0 equiv.) followed by arylboronic acid (1.5 equiv.). Water (0.25 M with respect to 4-cyanopyridine) was then added, followed by silver(I) nitrate (0.2 equiv.) and potassium persulfate (3.0 equiv.). Then the solution was stirred vigorously at room temperature for 3 hours, a second addition of silver(I) nitrate (0.2 equiv.) and potassium persulfate (3.0 equiv.) was added. The reaction mixture was allowed to stir for another 12 h. After that, the mixture was extracted with DCM. The organic layer was combined and washed with brine, dried over $MgSO_4$, filtered, and concentrated under reduced pressure. The crude material was purified by flash column chromatography (pentane /EtOAc) to give the product **4**.

2.3.4. General Procedure D: For Reactions of Boronic Esters 1a-1w and 5a-5v with 4a-4d

A flame-dried nitrogen-flushed Schlenk tube (10 mL) was charged with 4-bromo-*N,N*-dimethylaniline (0.32 mmol, 1.6 equiv.) and THF (0.8 mL). The solution was cooled to –78 °C (dry ice/acetone) then *t*-BuLi (0.64 mmol, 3.2 equiv., 1.7 M in pentane) was added slowly dropwise and the mixture was stirred for 5 min before removing the cooling bath, then the solution was allowed to stir at 0 °C for 30 min (**Figure S2a and b**).

In glovebox, to a 7 mL vial equipped with a magnetic stir bar was added the boronic ester (**1**) (0.30 mmol, 1.5 equiv.). The vial was sealed with a septum and removed from the glovebox, and then anhydrous diethyl ether (2.0 mL) was added under N_2. The solution was cooled to −78 °C (dry ice/acetone) and the pre-prepared [4-(dimethylamino)phenyl]lithium solution (from above) was added dropwise (**Figure S2c and d**). The mixture was allowed to stir for 30 min at −78 °C before removing the cooling bath and warming to ambient temperature. After 30 min, the solvent was removed under high vacuum, and the vial with boronate complex was quickly put into the glovebox, and then the cyanopyridine (0.20 mmol, 1.0 equiv.) and anhydrous CH_3CN (1.0 mL) were added. The vial was tightly sealed, then removed from the glovebox and stirred under blue LED irradiation for 16 h (see **Figure S1** for experimental setup). After irradiation, the reaction mixture was diluted with Et_2O (5.0 mL) and washed with 2.0 M aqueous HCl solution (5.0 mL) and brine (5.0 mL), dried over $MgSO_4$, filtered, and concentrated under reduced pressure. The crude material was purified by flash column chromatography (pentane/diethyl ether or pentane/EtOAc) to afford the coupled product (or oxidized to the corresponding alcohol according to the procedure reported below).

Oxidation of Boronic Esters: The crude material was dissolved in THF (3.0 mL) and cooled to 0 °C. An aqueous solution of sodium hydroxide (2.0 M, 1.0 mL) and aqueous hydrogen peroxide (30%, 1.0 mL) were added dropwise. After 10 min, the vigorously stirred biphasic reaction mixture was allowed to reach ambient

temperature and stirred for another 2 h, at which point TLC analysis showed the disappearance of the boronic ester. The mixture was diluted with water (5 mL) and ethyl acetate (10 mL) and the phases were separated. The aqueous phase was washed with ethyl acetate (3 × 10 mL), and the combined organic phases were dried over Na_2SO_4, filtered, and concentrated under reduced pressure. The resulting crude material was purified by flash column chromatography to afford the alcohol product.

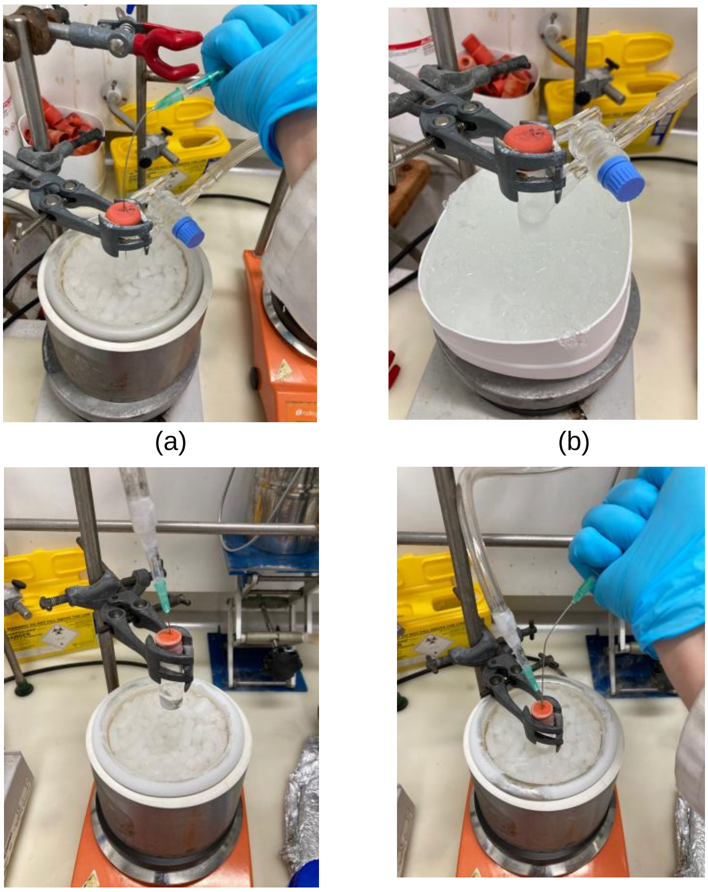

(a) (b) (c) (d)

Figure S2. Initial Reaction Setup.

2.3.5. General Procedure E: For Reactions of Bis-boronic Ester 1a with 4-Cyanopyridines 4e-4m

In glovebox, to a 7 mL vial equipped with a magnetic stir bar was added the 2,2'-(4,4-dimethylpentane-1,2-diyl)bis(4,4,5,5-tetramethyl-1,3,2-dioxaborolane) (**1a**) (106 mg, 0.300 mmol, 1.50 equiv.). The vial was sealed with a septum and removed from the glovebox, and then anhydrous diethyl ether (2.0 mL) was added under N_2. The solution was cooled to −78 °C (dry ice/acetone) and PhLi (0.32 mmol, 1.6 equiv., 1.9 M in dibutyl ether) was added dropwise. The mixture was allowed to stir for 30 min at −78 °C before removing the cooling bath and warming to ambient temperature. After 30 min, the solvent was removed under high vacuum, and the vial with boronate complex was quickly put into the glovebox, and then the 4-cyanopyridine (0.20 mmol, 1.0 equiv.), 4CzIPN (7.9 mg, 0.010 mmol, 5.0 mol%) and anhydrous CH_3CN (1.5 mL) were added. The vial was tightly sealed, then removed from the glovebox and stirred under blue LED irradiation for 16 h (see **Figure S1** for experimental setup). After irradiation, the reaction mixture was transferred to a 50 mL round flask with Et_2O (10 mL) and concentrated under reduced pressure. The crude material was purified by flash column

UOB Confidential & Sensitive

chromatography (pentane/diethyl ether or pentane/EtOAc) to afford the coupled product.

2.4. Synthesis and Purchasing of Starting Materials

The following starting materials were purchased from commercial suppliers:

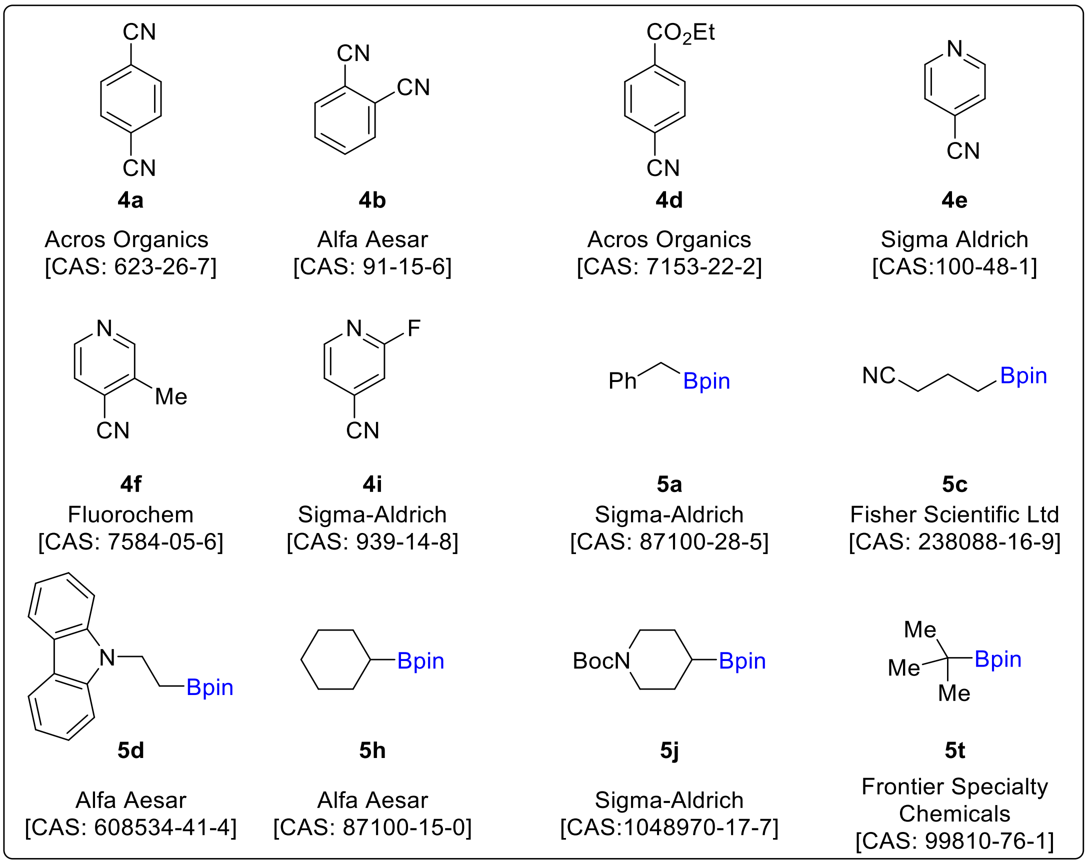

Scheme S3. Commercially Available Starting Materials.

UOB Confidential & Sensitive

The following boronic esters were synthesised according to the reports by our group:[5-14]

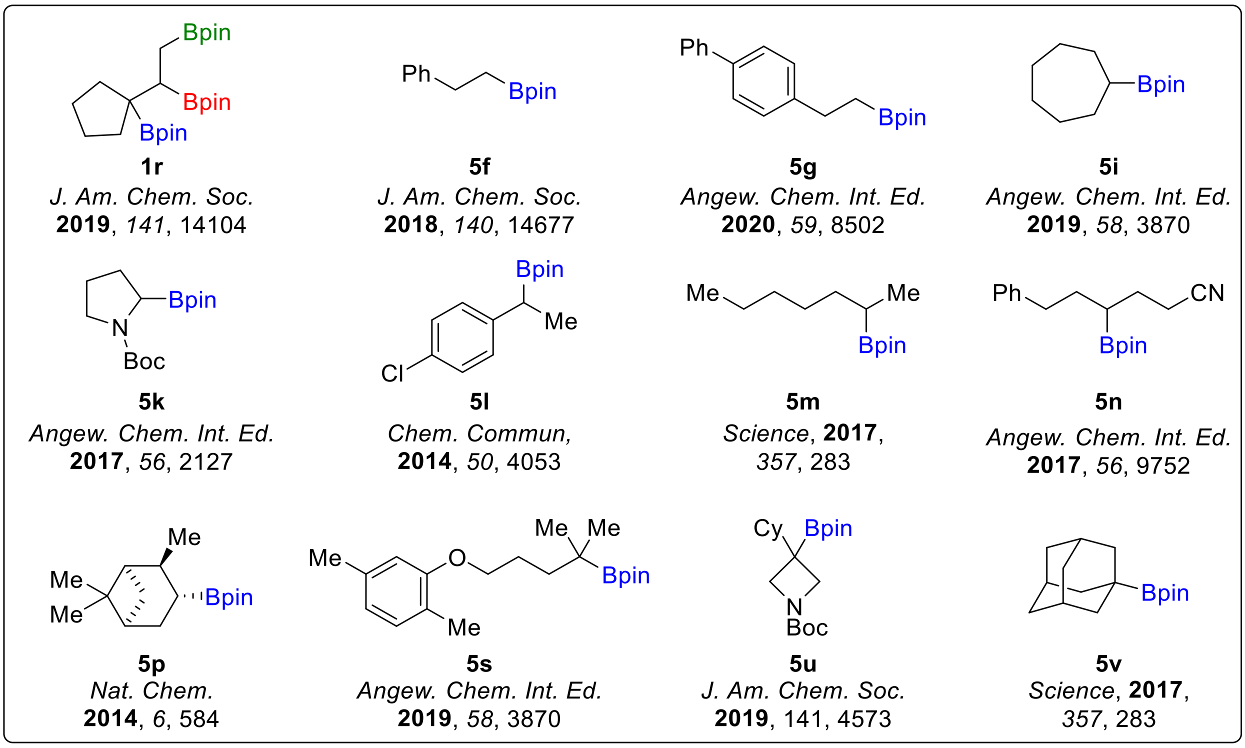

Scheme S4. Previously Reported Boronic Ester Substrates.

2,2'-(4,4-Dimethylpentane-1,2-diyl)bis(4,4,5,5-tetramethyl-1,3,2-dioxaborolane) (1a)

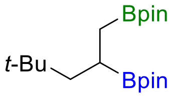

1a

Prepared following **General Procedure A** using 4,4-dimethylpent-1-ene (1.44 mL, 10.0 mmol, 1.00 equiv.), B_2pin_2 (2.79 g, 11.0 mmol, 1.10 equiv.), Cs_2CO_3 (0.53 g, 1.5 mmol, 15 mol%), anhydrous methanol (2.02 mL, 50.0 mmol, 5.00 equiv.) and anhydrous THF (50 mL). Purification by flash column chromatography (95:5 pentane/diethyl ether) gave the title compound (2.78 g, 79%) as a white solid.

TLC: R_f = 0.24 (95:5 *n*-hexane/EtOAc, CAM stain).

NMR Spectroscopy (*see spectra*):

> **^{1}H NMR** (400 MHz, $CDCl_3$): δ_H 1.58 (dd, *J* = 13.0, 9.8 Hz, 1H), 1.22 (s, 12H), 1.22 (s, 6H), 1.21 (s, 6H), 1.17 – 1.04 (m, 2H), 0.91 – 0.80 (m, 10H), 0.73 (dd, *J* = 15.5, 7.8 Hz, 1H) ppm;
>
> **^{13}C NMR** (101 MHz, $CDCl_3$): δ_C 83.0, 82.8, 48.5, 31.3, 29.9, 25.1, 25.0, 24.9 ppm. The carbon attached to boron was not observed due to quadrupolar relaxation;
>
> **^{11}B NMR** (128 MHz, $CDCl_3$): δ_B 35.0 ppm.

IR (film): ν_{max} 2977, 2950, 1468, 1370, 1311, 1269, 1213, 1141, 968, 865, 846, 671 cm^{-1}.

All recorded spectroscopic data matched those previously reported in the literature.[12]

2,2'-(Pentane-1,2-diyl)bis(4,4,5,5-tetramethyl-1,3,2-dioxaborolane) (1b)

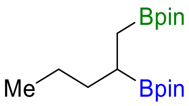

1b

Prepared following **General Procedure A** using pent-1-ene (1.09 mL, 10.0 mmol, 1.00 equiv.), B_2pin_2 (2.79 g, 11.0 mmol, 1.10 equiv.), Cs_2CO_3 (0.53 g, 1.5 mmol, 15 mol%), anhydrous methanol (2.02 mL, 50.0 mmol, 5.00 equiv.) and anhydrous THF (50 mL). Purification by flash column chromatography (95:5 pentane/diethyl ether) gave the title compound (2.3 g, 71%) as a colorless oil.

TLC: R_f = 0.24 (95:5 *n*-hexane/EtOAc, CAM stain).

NMR Spectroscopy (*see spectra*):

> **^{1}H NMR** (400 MHz, $CDCl_3$): δ_H 1.47 – 1.39 (m, 1H), 1.35 – 1.26 (m, 3H), 1.22 (s, 12H), 1.22 (s, 12H), 1.16 – 1.07 (m, 1H), 0.91 – 0.75 (m, 5H) ppm;
>
> **^{13}C NMR** (101 MHz, $CDCl_3$): δ_C 82.9, 82.9, 36.3, 25.0, 25.0, 24.9, 24.9, 22.1, 14.5 ppm. The carbon attached to boron was not observed due to quadrupolar relaxation;
>
> **^{11}B NMR** (128 MHz, $CDCl_3$): δ_B 33.3 ppm.

UOB Confidential & Sensitive

IR (film): ν_{max} 2977, 2927, 1369, 1350, 1310, 1212, 1140, 968, 846, 671, 578 cm^{-1}.

All recorded spectroscopic data matched those previously reported in the literature.[12]

2,2'-(4-Methylpentane-1,2-diyl)bis(4,4,5,5-tetramethyl-1,3,2-dioxaborolane) (1c)

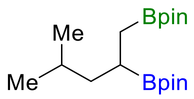

1c

Prepared following **General Procedure A** using 4-methylpent-1-ene (1.27 mL, 10.0 mmol, 1.00 equiv.), B_2pin_2 (2.79 g, 11.0 mmol, 1.10 equiv.), Cs_2CO_3 (0.53 g, 1.5 mmol, 15 mol%), anhydrous methanol (2.02 mL, 50.0 mmol, 5.00 equiv.) and anhydrous THF (50 mL). Purification by flash column chromatography (95:5 pentane/diethyl ether) gave the title compound (2.5 g, 74%) as a colorless oil.

TLC: R_f = 0.24 (95:5 *n*-hexane/EtOAc, CAM stain).

NMR Spectroscopy (*see spectra*):

^{1}**H NMR** (400 MHz, $CDCl_3$): δ_H 1.65 – 1.53 (m, 1H), 1.41 – 1.30 (m, 1H), 1.23 (s, 12H), 1.22 (s, 12H), 1.21 – 1.11 (m, 2H), 0.86 (d, *J* = 6.6 Hz, 3H), 0.84 (d, *J* = 6.6 Hz, 3H), 0.82 – 0.79 (m, 2H) ppm;

^{13}**C NMR** (101 MHz, $CDCl_3$): δ_C 83.0, 82.9, 43.1, 26.9, 25.1, 25.0, 24.9, 24.9, 23.0, 22.8 ppm. The carbon attached to boron was not observed due to quadrupolar relaxation;

^{11}**B NMR** (128 MHz, $CDCl_3$): δ_B 34.0 ppm.

IR (film): ν_{max} 2977, 2953, 1467, 1369, 1311, 1247, 1212, 1141, 968, 848, 672 cm^{-1}.

HRMS (APCI$^+$): m/z calculated for $C_{18}H_{37}B_2O_4$ $[M+H]^+$, 339.2872; found, 339.2879.

2,2'-(1-Cyclohexylethane-1,2-diyl)bis(4,4,5,5-tetramethyl-1,3,2-dioxaborolane) (1d)

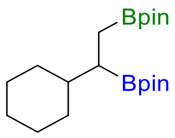

1d

Prepared following **General Procedure A** using vinylcyclohexane (1.37 mL, 10.0 mmol, 1.00 equiv.), B_2pin_2 (2.79 g, 11.0 mmol, 1.10 equiv.), Cs_2CO_3 (0.53 g, 1.5 mmol, 15 mol%), anhydrous methanol (2.02 mL, 50.0 mmol, 5.00 equiv.) and anhydrous THF (50 mL). Purification by flash column chromatography (95:5 pentane/diethyl ether) gave the title compound (1.93 g, 53%) as a colorless oil.

TLC: R_f = 0.24 (95:5 *n*-hexane/EtOAc, CAM stain).

NMR Spectroscopy (*see spectra*):

^{1}**H NMR** (400 MHz, $CDCl_3$): δ_H 1.73 – 1.56 (m, 5H), 1.41 – 1.28 (m, 1H), 1.23 (s, 12H), 1.22 (s, 12H), 1.17

– 0.93 (m, 6H), 0.86 (dd, J = 15.7, 11.0 Hz, 1H), 0.76 (dd, J = 15.7, 5.0 Hz, 1H) ppm;

^{13}C NMR (101 MHz, $CDCl_3$): δ_C 82.9, 82.9, 41.6, 32.3, 32.1, 27.0, 27.0, 26.9, 25.1, 25.1, 25.0, 24.9 ppm. The carbon attached to boron was not observed due to quadrupolar relaxation;

^{11}B NMR (128 MHz, $CDCl_3$): δ_B 34.3 ppm.

IR (film): ν_{max} 2978, 2923, 2851, 1740, 1370, 1311, 1216, 1144, 970, 846 cm^{-1}.

All recorded spectroscopic data matched those previously reported in the literature.[12]

2,2'-(3,3-Dimethylbutane-1,2-diyl)bis(4,4,5,5-tetramethyl-1,3,2-dioxaborolane) (1e)

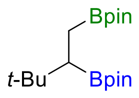

1e

Prepared following **General Procedure A** using 3,3-dimethylbut-1-ene (1.29 mL, 10.0 mmol, 1.00 equiv.), B_2pin_2 (2.79 g, 11.0 mmol, 1.10 equiv.), Cs_2CO_3 (0.53 g, 1.5 mmol, 15 mol%), anhydrous methanol (2.02 mL, 50.0 mmol, 5.00 equiv.) and anhydrous THF (50 mL). Purification by flash column chromatography (95:5 pentane/diethyl ether) gave the title compound (1.86 g, 55%) as a white solid.

TLC: R_f = 0.24 (95:5 *n*-hexane/EtOAc, CAM stain).

NMR Spectroscopy (*see spectra*):

^{1}H NMR (400 MHz, $CDCl_3$): δ_H 1.25 (s, 12H), 1.22 (s, 6H), 1.21 (s, 6H), 0.96 (dd, J = 11.6, 4.6 Hz, 1H), 0.91 (s, 9H), 0.88 – 0.75 (m, 2H) ppm;

^{13}C NMR (101 MHz, $CDCl_3$): δ_C 83.0, 82.8, 32.5, 29.2, 25.4, 25.2, 25.0, 24.8 ppm. The carbon attached to boron was not observed due to quadrupolar relaxation;

^{11}B NMR (128 MHz, $CDCl_3$): δ_B 33.6 ppm.

IR (film): ν_{max} 2977, 1740, 1467, 1370, 1312, 1216, 1144, 969, 848 cm^{-1}.

All recorded spectroscopic data matched those previously reported in the literature.[12]

[2,3-Bis(4,4,5,5-tetramethyl-1,3,2-dioxaborolan-2-yl)propyl]trimethylsilane (1f)

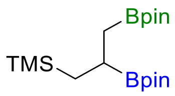

1f

Prepared following **General Procedure A** using allyltrimethylsilane (1.59 mL, 10.0 mmol, 1.00 equiv.), B_2pin_2 (2.79 g, 11.0 mmol, 1.10 equiv.), Cs_2CO_3 (0.53 g, 1.5 mmol, 15 mol%), anhydrous methanol (2.02 mL, 50.0 mmol, 5.00 equiv.) and anhydrous THF (50 mL). Purification by flash column chromatography (95:5 pentane/diethyl ether) gave the title compound (2.84 g, 77%) as a colorless oil.

TLC: R_f = 0.28 (95:5 *n*-hexane/EtOAc, CAM stain).

NMR Spectroscopy (*see spectra*):

^{1}**H NMR** (400 MHz, $CDCl_3$): δ_H 1.23 (s, 12H), 1.22 (s, 12H), 1.21 - 1.14 (m, 1H), 0.96 – 0.76 (m, 3H), 0.49 (dd, *J* = 14.6, 6.6 Hz, 1H), -0.02 (s, 9H) ppm;

^{13}**C NMR** (101 MHz, $CDCl_3$): δ_C 82.9, 82.9, 25.1, 25.0, 24.9, 24.9, 20.5, -0.6 ppm. The carbon attached to boron was not observed due to quadrupolar relaxation;

^{11}**B NMR** (128 MHz, $CDCl_3$): δ_B 33.0 ppm.

IR (film): ν_{max} 2978, 1740, 1369, 1311, 1245, 1215, 968, 835, 690 cm^{-1}.

All recorded spectroscopic data matched those previously reported in the literature.[15]

2,2'-(6-Chlorohexane-1,2-diyl)bis(4,4,5,5-tetramethyl-1,3,2-dioxaborolane) (1g)

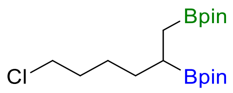

1g

Prepared following **General Procedure A** using 6-chlorohex-1-ene (0.66 mL, 5.0 mmol, 1.0 equiv.), B_2pin_2 (1.4 g, 5.5 mmol, 1.1 equiv.), Cs_2CO_3 (0.25 g, 1.5 mmol, 15 mol%), anhydrous methanol (1.01 mL, 25.0 mmol, 5.00 equiv.) and anhydrous THF (25 mL). Purification by flash column chromatography (91:9 pentane/diethyl ether) gave the title compound (1.23 g, 66%) as a colorless oil.

TLC: R_f = 0.36 (91:9 *n*-hexane/EtOAc, CAM stain).

NMR Spectroscopy (*see spectra*):

^{1}**H NMR** (400 MHz, $CDCl_3$): δ_H 3.51 (t, *J* = 6.8 Hz, 1H), 3.47 (q, *J* = 7.0 Hz, 1H), 1.75 (dt, *J* = 8.5, 6.6 Hz, 2H), 1.51 – 1.30 (m, 4H), 1.23 (s, 12H), 1.22 (s, 12H), 1.16 – 1.06 (m, 1H), 0.88 (dd, *J* = 15.8, 9.4 Hz, 1H), 0.79 (dd, *J* = 15.8, 6.0 Hz, 1H) ppm;

^{13}**C NMR** (101 MHz, $CDCl_3$): δ_C 83.0, 45.3, 33.0, 33.0, 26.3, 25.1, 25.0, 24.9, 24.9 ppm. The carbon attached to boron was not observed due to quadrupolar relaxation;

^{11}**B NMR** (128 MHz, $CDCl_3$): δ_B 34.6 ppm.

IR (film): ν_{max} 2977, 2930, 1740, 1370, 1312, 1215, 1141, 968, 846 cm^{-1}.

All recorded spectroscopic data matched those previously reported in the literature.[16]

UOB Confidential & Sensitive

tert-Butyl 6,7-bis(4,4,5,5-tetramethyl-1,3,2-dioxaborolan-2-yl)heptanoate (1h)

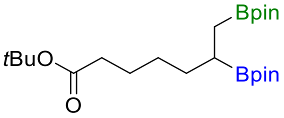

1h

Prepared following **General Procedure A** using *tert*-butyl hept-6-enoate (0.92 g, 5.0 mmol, 1.0 equiv.), B_2pin_2 (1.4 g, 5.5 mmol, 1.1 equiv.), Cs_2CO_3 (0.25 g, 1.5 mmol, 15 mol%), anhydrous methanol (1.01 mL, 25.0 mmol, 5.00 equiv.) and anhydrous THF (25 mL). Purification by flash column chromatography (90:10 pentane/diethyl ether) gave the title compound (0.99 g, 45%) as a colorless oil.

TLC: R_f = 0.24 (91:9 *n*-hexane/EtOAc, CAM stain).

NMR Spectroscopy (*see spectra*):

^{1}**H NMR** (400 MHz, $CDCl_3$): δ_H 2.18 (t, *J* = 7.6 Hz, 2H), 1.60 – 1.51 (m, 2H), 1.49 – 1.44 (m, 1H), 1.43 (s, 9H), 1.37 – 1.26 (m, 3H), 1.22 (s, 12H), 1.22 (s, 12H), 1.15 – 1.05 (m, 1H), 0.86 (dd, *J* = 15.8, 9.4 Hz, 1H), 0.78 (dd, *J* = 15.8, 5.9 Hz, 1H) ppm;

^{13}**C NMR** (101 MHz, $CDCl_3$): δ_C 173.5, 83.0, 82.9, 79.9, 35.8, 33.5, 28.5, 28.3, 25.5, 25.1, 25.0, 24.9, 24.9 ppm. The carbon attached to boron was not observed due to quadrupolar relaxation;

^{11}**B NMR** (128 MHz, $CDCl_3$): δ_B 34.3 ppm.

IR (film): ν_{max} 2976, 2931, 1732, 1368, 1313, 1216, 1144, 968, 847 cm^{-1}.

All recorded spectroscopic data matched those previously reported in the literature.[17]

2,2'-(4-Phenylbutane-1,2-diyl)bis(4,4,5,5-tetramethyl-1,3,2-dioxaborolane) (1i)

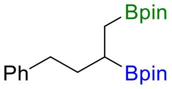

1i

Prepared following **General Procedure A** using but-3-en-1-ylbenzene (0.75 mL, 5.0 mmol, 1.0 equiv.), B_2pin_2 (1.4 g, 5.5 mmol, 1.1 equiv.), Cs_2CO_3 (0.25 g, 1.5 mmol, 15 mol%), anhydrous methanol (1.01 mL, 25.0 mmol, 5.00 equiv.) and anhydrous THF (25 mL). Purification by flash column chromatography (91:9 pentane/diethyl ether) gave the title compound (1.24 g, 64%) as a colorless oil.

TLC: R_f = 0.36 (91:9 *n*-hexane/EtOAc, CAM stain).

NMR Spectroscopy (*see spectra*):

^{1}**H NMR** (400 MHz, $CDCl_3$): δ_H 7.30 – 7.21 (m, 2H), 7.20 – 7.12 (m, 3H), 2.62 (t, *J* = 8.3 Hz, 2H), 1.86 – 1.73 (m, 1H), 1.71 – 1.58 (m, 1H), 1.25 (s, 12H), 1.24 (s, 12H), 1.20 – 1.16 (m, 1H), 0.95 (dd, *J* = 15.8, 9.4 Hz, 1H), 0.87 (dd, *J* = 15.8, 5.9 Hz, 1H) ppm;

^{13}**C NMR** (101 MHz, $CDCl_3$): δ_C 143.5, 128.6, 128.3, 125.6, 83.0, 36.1, 35.5, 25.1, 25.0, 24.9, 24.9 ppm.

UOB Confidential & Sensitive

The carbon attached to boron was not observed due to quadrupolar relaxation;

^{11}B NMR (128 MHz, $CDCl_3$): δ_B 34.5 ppm.

IR (film): ν_{max} 2977, 2927, 1740, 1454, 1369, 1312, 1215, 1141, 968, 845, 698 cm^{-1}.

All recorded spectroscopic data matched those previously reported in the literature.[12]

2,2'-[4-(Naphthalen-2-yl)butane-1,2-diyl]bis(4,4,5,5-tetramethyl-1,3,2-dioxaborolane) (1j)

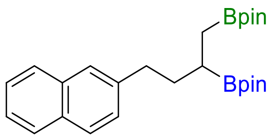

1j

Prepared following **General Procedure A** using 2-(but-3-en-1-yl)naphthalene (0.91 g, 5.0 mmol, 1.0 equiv.), B_2pin_2 (1.40 g, 5.50 mmol, 1.10 equiv.), Cs_2CO_3 (0.25 g, 1.5 mmol, 15 mol%), anhydrous methanol (1.01 mL, 25.0 mmol, 5.00 equiv.) and anhydrous THF (25.0 mL). Purification by flash column chromatography (91:9 pentane/diethyl ether) gave the title compound (1.31 g, 60%) as a white solid.

TLC: R_f = 0.33 (91:9 *n*-hexane/EtOAc, CAM stain).

NMR Spectroscopy (*see spectra*):

^{1}H NMR (400 MHz, $CDCl_3$): δ_H 7.82 – 7.71 (m, 3H), 7.62 (s, 1H), 7.47 – 7.35 (m, 2H), 7.35 (dd, *J* = 8.4, 1.7 Hz, 1H), 2.80 (t, *J* = 8.2 Hz, 2H), 1.95 – 1.83 (m, 1H), 1.79 – 1.68 (m, 1H), 1.27 (s, 12H), 1.25 (s, 12H), 1.00 (dd, *J* = 15.8, 9.3 Hz, 1H), 0.92 (dd, *J* = 15.8, 6.0 Hz, 1H) ppm;

^{13}C NMR (101 MHz, $CDCl_3$): δ_C 141.0, 133.8, 132.0, 127.8, 127.8, 127.7, 127.5, 126.4, 125.8, 125.0, 83.0, 83.0, 35.9, 35.6, 25.1, 25.0, 24.9, 24.9 ppm. The carbon attached to boron was not observed due to quadrupolar relaxation;

^{11}B NMR (128 MHz, $CDCl_3$): δ_B 34.9 ppm.

IR (film): ν_{max} 2976, 2928, 1740, 1369, 1314, 1216, 1142, 968, 845, 476 cm^{-1}.

HRMS (APCI$^+$): m/z calculated for $C_{26}H_{39}B_2O_4$ [M+H]$^+$, 437.3029; found, 437.3036.

2,2'-(6-Phenylhexane-1,2-diyl)bis(4,4,5,5-tetramethyl-1,3,2-dioxaborolane) (1k)

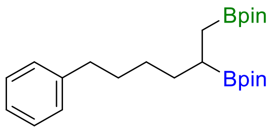

1k

Prepared following **General Procedure A** using hex-5-en-1-ylbenzene (1.60 g, 10.0 mmol, 1.00 equiv.), B_2pin_2 (2.79 g, 11.0 mmol, 1.10 equiv.), Cs_2CO_3 (0.53 g, 1.5 mmol, 15 mol%), anhydrous methanol (2.02 mL, 50.0

mmol, 5.00 equiv.) and anhydrous THF (50 mL). Purification by flash column chromatography (95:5 pentane/diethyl ether) gave the title compound (2.74 g, 66%) as a white solid.

TLC: R_f = 0.36 (91:9 *n*-hexane/EtOAc, CAM stain).

NMR Spectroscopy (*see spectra*):

> **^{1}H NMR** (400 MHz, $CDCl_3$): δ_H 7.28 – 7.21 (m, 2H), 7.19 – 7.11 (m, 3H), 2.59 (dd, *J* = 8.6, 6.8 Hz, 2H), 1.65 – 1.55 (m, 2H), 1.52 – 1.44 (m, 1H), 1.40 – 1.29 (m, 3H), 1.22 (s, 12H), 1.21 (s, 12H), 1.15 – 1.04 (m, 1H), 0.87 (dd, *J* = 15.8, 9.5 Hz, 1H), 0.79 (dd, *J* = 15.8, 6.0 Hz, 1H) ppm;

> **^{13}C NMR** (101 MHz, $CDCl_3$): δ_C 143.1, 128.6, 128.3, 125.6, 83.0, 82.9, 36.0, 33.8, 31.8, 28.7, 25.1, 25.0, 24.9, 24.9 ppm. The carbon attached to boron was not observed due to quadrupolar relaxation;

IR (film): ν_{max} 2977, 2927, 2856, 1369, 1310, 1269, 1214, 1140, 968, 846, 746, 698 cm^{-1}.

HRMS (APCI$^+$): m/z calculated for $C_{24}H_{41}B_2O_4$ $[M+H]^+$, 415.3185; found, 415.3183.

All recorded spectroscopic data matched those previously reported in the literature.[18]

2,2'-(1-Phenylethane-1,2-diyl)bis(4,4,5,5-tetramethyl-1,3,2-dioxaborolane) (1l)

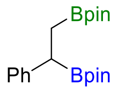

1l

Prepared following **General Procedure A** using styrene (1.15 mL, 10.0 mmol, 1.00 equiv.), B_2pin_2 (2.79 g, 11.0 mmol, 1.10 equiv.), Cs_2CO_3 (0.53 g, 1.5 mmol, 15 mol%), anhydrous methanol (2.02 mL, 50.0 mmol, 5.00 equiv.) and anhydrous THF (50 mL). Purification by flash column chromatography (95:5 pentane/diethyl ether) gave the title compound (2.83 g, 79%) as a white solid.

TLC: R_f = 0.30 (91:9 *n*-hexane/EtOAc, CAM stain).

NMR Spectroscopy (*see spectra*):

> **^{1}H NMR** (400 MHz, $CDCl_3$): δ_H 7.24 – 7.18 (m, 4H), 7.12 – 7.06 (m, 1H), 2.52 (dd, *J* = 11.0, 5.7 Hz, 1H), 1.38 (dd, *J* = 15.9, 11.0 Hz, 1H), 1.20 (s, 12H), 1.19 (s, 6H), 1.17 (s, 6H), 1.12 (dd, *J* = 15.9, 5.7 Hz, 1H) ppm;

> **^{13}C NMR** (101 MHz, $CDCl_3$): δ_C 145.5, 128.3, 128.0, 125.0, 83.3, 83.2, 25.1, 24.8, 24.8, 24.7, 24.6 ppm. The carbon attached to boron was not observed due to quadrupolar relaxation;

IR (film): ν_{max} 2978, 2932, 1740, 1368, 1317, 1216, 1143, 968, 845, 700 cm^{-1}.

All recorded spectroscopic data matched those previously reported in the literature.[16]

2,2'-[1-(4-Methoxyphenyl)ethane-1,2-diyl]bis(4,4,5,5-tetramethyl-1,3,2-dioxaborolane) (1m)

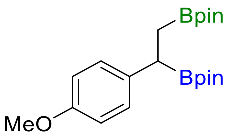

1m

Prepared following **General Procedure A** using 1-methoxy-4-vinylbenzene (1.33 mL, 10.0 mmol, 1.00 equiv.), B_2pin_2 (2.79 g, 11.0 mmol, 1.10 equiv.), Cs_2CO_3 (0.53 g, 1.5 mmol, 15 mol%), anhydrous methanol (2.02 mL, 50.0 mmol, 5.00 equiv.) and anhydrous THF (50 mL). Purification by flash column chromatography (75:25 pentane/diethyl ether) gave the title compound (2.37 g, 61%) as a white solid.

TLC: R_f = 0.25 (80:20 *n*-hexane/EtOAc, CAM stain).

NMR Spectroscopy (*see spectra*):

^{1}**H NMR** (400 MHz, $CDCl_3$): δ_H 7.14 (d, *J* = 8.6 Hz, 2H), 6.78 (d, *J* = 8.6 Hz, 2H), 3.76 (s, 3H), 2.46 (dd, *J* = 10.9, 5.8 Hz, 1H), 1.33 (dd, *J* = 16.0, 10.9 Hz, 1H), 1.20 (s, 12H), 1.19 (s, 6H), 1.17 (s, 6H), 1.08 (dd, *J* = 16.0, 5.8 Hz, 1H) ppm;

^{13}**C NMR** (101 MHz, $CDCl_3$): δ_C 157.3, 137.6, 128.9, 113.8, 83.3, 83.1, 55.3, 25.1, 24.9, 24.8, 24.7 ppm. The carbon attached to boron was not observed due to quadrupolar relaxation;

^{11}**B NMR** (128 MHz, $CDCl_3$): δ_B 33.6 ppm.

IR (film): ν_{max} 2977, 1740, 1512, 1476, 1453, 1371, 1217, 1145, 982, 851, 674 cm^{-1}.

All recorded spectroscopic data matched those previously reported in the literature.[17]

4,4,5,5-Tetramethyl-2-{[1-(4,4,5,5-tetramethyl-1,3,2-dioxaborolan-2-yl)cyclohexyl]methyl}-1,3,2-dioxaborolane (1n)

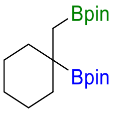

1n

Prepared following **General Procedure B** using methylenecyclohexane (0.48 mL, 4.0 mmol, 1.0 equiv.), B_2cat_2 (1.05 g, 4.40 mmol, 1.10 equiv.), *t*-butylamine (63 µL, 0.60 mmol, 15 mol%) and anhydrous 2-MeTHF (20 mL). Transesterification was performed using pinacol (2.83 g, 24.0 mmol, 6.00 equiv.) and triethylamine (10 mL). Purification by flash column chromatography (95:5 pentane/diethyl ether) gave the title compound (0.77 g, 55%) as a white solid.

TLC: R_f = 0.28 (95:5 *n*-hexane/EtOAc, CAM stain).

NMR Spectroscopy (*see spectra*):

^{1}**H NMR** (400 MHz, $CDCl_3$): δ_H 1.89 – 1.80 (m, 2H), 1.63 – 1.49 (m, 3H), 1.38 – 1.28 (m, 2H), 1.24 (s, 12H), 1.21 (s, 12H), 1.17 – 1.07 (m, 1H), 1.06 – 0.96 (m, 2H), 0.79 (s, 2H) ppm;

^{13}C NMR (101 MHz, $CDCl_3$): δ_C 82.9, 82.9, 37.4, 26.7, 25.2, 25.0, 25.0 ppm. The carbon attached to boron was not observed due to quadrupolar relaxation;

^{11}B NMR (128 MHz, $CDCl_3$): δ_B 34.1 ppm.

IR (film): ν_{max} 2977, 2923, 2851, 1740, 1449, 1370, 1304, 1233, 1144, 972, 850 cm^{-1}.

All recorded spectroscopic data matched those previously reported in the literature.[12]

2,2'-[(*cis*)-1-Methylcyclohexane-1,2-diyl]bis(4,4,5,5-tetramethyl-1,3,2-dioxaborolane) (1o)

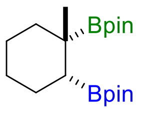

1o

Prepared following **General Procedure B** using 1-methylcyclohex-1-ene (0.47 mL, 4.0 mmol, 1.0 equiv.), B_2cat_2 (1.05 g, 4.40 mmol, 1.10 equiv.), *t*-butylamine (63 µL, 0.60 mmol, 15 mol%) and anhydrous 2-MeTHF (20 mL). Transesterification was performed using pinacol (2.83 g, 24.0 mmol, 6.00 equiv.) and triethylamine (10 mL). Purification by flash column chromatography (95:5 pentane/diethyl ether) gave the title compound (0.52 g, 37%) as a white solid.

TLC: R_f = 0.38 (95:5 *n*-hexane/EtOAc, CAM stain).

NMR Spectroscopy (*see spectra*):

^{1}H NMR (400 MHz, $CDCl_3$): δ_H 1.78 (dtd, *J* = 12.9, 3.6, 1.4 Hz, 1H), 1.71 – 1.51 (m, 3H), 1.50 – 1.42 (m, 1H), 1.39 – 1.29 (m, 1H), 1.22 (s, 12H), 1.21 (s, 12H), 1.13 (tt, *J* = 12.2, 3.6 Hz, 1H), 1.01 (s, 3H), 0.93 (td, *J* = 12.9, 3.4 Hz, 1H), 0.55 (dd, *J* = 12.2, 3.4 Hz, 1H) ppm;

^{13}C NMR (101 MHz, $CDCl_3$): δ_C 82.8, 82.6, 39.4, 27.5, 26.8, 26.3, 25.3, 25.0, 25.0, 24.9, 24.9 ppm. The carbon attached to boron was not observed due to quadrupolar relaxation;

^{11}B NMR (128 MHz, $CDCl_3$): δ_B 34.4 ppm.

IR (film): ν_{max} 2974, 2925, 1740, 1446, 1368, 1302, 1230, 1217, 1145, 528 cm^{-1}.

All recorded spectroscopic data matched those previously reported in the literature.[12]

UOB Confidential & Sensitive

2,2'-(2,3-Dimethylbutane-1,2-diyl)bis(4,4,5,5-tetramethyl-1,3,2-dioxaborolane) (1p)

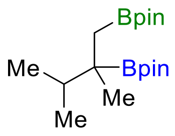

1p

Prepared following **General Procedure B** using 2,3-dimethylbut-1-ene (0.62 mL, 5.0 mmol, 1.0 equiv.), B_2cat_2 (1.31 g, 5.50 mmol, 1.10 equiv.), *t*-butylamine (79 µL, 0.75 mmol, 15 mol%) and anhydrous 2-MeTHF (20 mL). Transesterification was performed using pinacol (3.55 g, 30.0 mmol, 6.00 equiv.) and triethylamine (10 mL). Purification by flash column chromatography (95:5 pentane/diethyl ether) gave the title compound (1.10 g, 65%) as a colorless oil.

TLC: R_f = 0.31 (95:5 *n*-hexane/EtOAc, CAM stain).

NMR Spectroscopy (*see spectra*):

> ^{1}**H NMR** (400 MHz, $CDCl_3$): δ_H 1.62 – 1.53 (m, 1H), 1.25 (s, 12H), 1.22 (s, 6H), 1.22 (s, 6H), 0.96 (d, *J* = 15.4 Hz, 1H), 0.92 (d, *J* = 0.9 Hz, 3H), 0.87 (d, *J* = 6.8 Hz, 3H), 0.84 (d, *J* = 6.8 Hz, 3H), 0.66 (d, *J* = 15.4 Hz, 1H) ppm;
>
> ^{13}**C NMR** (101 MHz, $CDCl_3$): δ_C 83.0, 82.8, 36.3, 25.3, 25.3, 25.0, 24.8, 20.4, 19.1, 18.3 ppm. The carbon attached to boron was not observed due to quadrupolar relaxation;
>
> ^{11}**B NMR** (128 MHz, $CDCl_3$): δ_B 34.3 ppm.

IR (film): ν_{max} 2970, 1740, 1368, 1229, 1217, 1144, 528 cm^{-1}.

All recorded spectroscopic data matched those previously reported in the literature.[12]

(*cis*)-1,2-Bis(4,4,5,5-tetramethyl-1,3,2-dioxaborolan-2-yl)cyclopentane (1q)

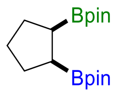

1q

Prepared following **General Procedure A** using cyclopentene (0.88 mL, 10 mmol, 1.0 equiv.), B_2pin_2 (2.79 g, 11.0 mmol, 1.10 equiv.), Cs_2CO_3 (0.53 g, 1.5 mmol, 15 mol%), anhydrous methanol (2.02 mL, 50.0 mmol, 5.00 equiv.) and anhydrous THF (50 mL). Purification by flash column chromatography (95:5 pentane/diethyl ether) gave the title compound (2.0 g, 62%) as a colorless oil.

TLC: R_f = 0.28 (95:5 *n*-hexane/EtOAc, CAM stain).

NMR Spectroscopy (*see spectra*):

> ^{1}**H NMR** (400 MHz, $CDCl_3$): δ_H 1.77 – 1.65 (m, 2H), 1.66 – 1.56 (m, 3H), 1.55 – 1.47 (m, 1H), 1.39 (td, *J* = 6.1, 2.5 Hz, 2H), 1.23 (s, 12H), 1.23 (s, 12H) ppm;
>
> ^{13}**C NMR** (101 MHz, $CDCl_3$): δ_C 82.9, 28.8, 25.8, 25.0, 24.9 ppm. The carbon attached to boron was not

observed due to quadrupolar relaxation;

^{11}B NMR (128 MHz, $CDCl_3$): δ_B 34.6 ppm.

IR (film): ν_{max} 2976, 2941, 1740, 1377, 1308, 1219, 1141, 970, 858, 727, 670 cm^{-1}.

All recorded spectroscopic data matched those previously reported in the literature.[19]

(*cis*)-1,2-Bis(4,4,5,5-tetramethyl-1,3,2-dioxaborolan-2-yl)cyclohexane (1r)

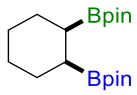

1r

Prepared following **General Procedure A** using cyclohexene (1.01 mL, 10.0 mmol, 1.00 equiv.), B_2pin_2 (2.79 g, 11.0 mmol, 1.10 equiv.), Cs_2CO_3 (0.53 g, 1.5 mmol, 15 mol%), anhydrous methanol (2.02 mL, 50.0 mmol, 5.00 equiv.) and anhydrous THF (50 mL). Purification by flash column chromatography (95:5 pentane/diethyl ether) gave the title compound (2.16 g, 67%) as a colorless oil.

TLC: R_f = 0.28 (95:5 *n*-hexane/EtOAc, CAM stain).

NMR Spectroscopy (*see spectra*):

^{1}H NMR (400 MHz, $CDCl_3$): δ_H 1.66 – 1.48 (m, 4H), 1.48 – 1.31 (m, 4H), 1.29 – 1.14 (m, 26H) ppm;

^{13}C NMR (101 MHz, $CDCl_3$): δ_C 82.9, 28.2, 27.0, 25.0, 25.0 ppm. The carbon attached to boron was not observed due to quadrupolar relaxation;

^{11}B NMR (128 MHz, $CDCl_3$): δ_B 34.4 ppm.

IR (film): ν_{max} 2976, 2924, 2850, 1740, 1412, 1378, 1306, 1237, 1216, 1144, 970, 859 cm^{-1}.

All recorded spectroscopic data matched those previously reported in the literature.[16]

2,2'-[(2*R*)-2,6,6-Trimethylbicyclo[3.1.1]heptane-2,3-diyl]bis(4,4,5,5-tetramethyl-1,3,2-dioxaborolane) (1t)

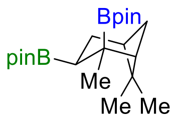

1t

Prepared following **General Procedure B** using (–)-*α*-pinene (87% *ee*) (0.66 mL, 4.0 mmol, 1.0 equiv.), B_2cat_2 (1.05 g, 4.40 mmol, 1.10 equiv.), *t*-butylamine (63 µL, 0.60 mmol, 15 mol%) and anhydrous 2-MeTHF (20 mL). Transesterification was performed using pinacol (2.83 g, 24.0 mmol, 6.00 equiv.) and triethylamine (10 mL). Purification by flash column chromatography (95:5 pentane/diethyl ether) gave the title compound (0.44 g, 28%) as a white solid.

TLC: R_f = 0.42 (95:5 *n*-hexane/EtOAc, CAM stain).

UOB Confidential & Sensitive

NMR Spectroscopy (*see spectra*):

^{1}H NMR (400 MHz, $CDCl_3$): δ_H 2.23 (dtd, *J* = 9.5, 6.2, 1.9 Hz, 1H), 2.05 – 1.90 (m, 2H), 1.87 – 1.80 (m, 2H), 1.43 (t, *J* = 9.8 Hz, 1H), 1.26 (s, 12H), 1.20 (s, 6H), 1.19 (s, 9H), 1.17 (s, 3H), 1.16 (s, 3H), 0.98 (d, *J* = 9.5 Hz, 1H) ppm;

^{13}C NMR (101 MHz, $CDCl_3$): δ_C 83.1, 82.6, 50.2, 41.2, 41.0, 34.0, 30.2, 29.6, 25.6, 25.3, 25.2, 24.8, 24.4, 23.5 ppm. The carbon attached to boron was not observed due to quadrupolar relaxation;

^{11}B NMR (128 MHz, $CDCl_3$): δ_B 32.4 ppm.

IR (film): ν_{max} 2976, 2923, 1740, 1457, 1369, 1354, 1300, 1217, 1147, 1102, 861, 528 cm^{-1}.

All recorded spectroscopic data matched those previously reported in the literature.[12]

2-{(2*R*)-6,6-Dimethyl-2-[(4,4,5,5-tetramethyl-1,3,2-dioxaborolan-2-yl)methyl]bicyclo[3.1.1]heptan-2-yl}-4,4,5,5-tetramethyl-1,3,2-dioxaborolane (1u)

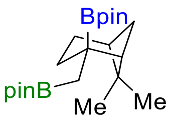

1u

Prepared following **General Procedure B** using (–)-*β*-pinene (0.64 mL, 4.0 mmol, 1.0 equiv.), B_2cat_2 (1.05 g, 4.40 mmol, 1.10 equiv.), *t*-butylamine (63 µL, 0.60 mmol, 15 mol%) and anhydrous 2-MeTHF (20 mL). Transesterification was performed using pinacol (2.83 g, 24.0 mmol, 6.00 equiv.) and triethylamine (10 mL). Purification by flash column chromatography (95:5 pentane/diethyl ether) gave the title compound (0.69 g, 44%) as a white solid.

TLC: R_f = 0.32 (95:5 *n*-hexane/EtOAc, CAM stain).

NMR Spectroscopy (*see spectra*):

^{1}H NMR (400 MHz, $CDCl_3$): δ_H 2.28 – 2.17 (m, 2H), 1.97 – 1.75 (m, 4H), 1.44 (ddd, *J* = 14.9, 10.8, 6.3 Hz, 1H), 1.27 -1.25 (m, 1H),1.24 (s, 6H), 1.23 (s, 6H), 1.21 (s, 6H), 1.20 (s, 6H), 1.17 (s, 3H), 1.10 (s, 3H), 0.99 (d, *J* = 9.6 Hz, 1H), 0.93 (d, *J* = 15.3 Hz, 1H) ppm;

^{13}C NMR (101 MHz, $CDCl_3$): δ_C 83.1, 82.9, 50.0, 41.1, 40.7, 32.4, 28.7, 27.2, 25.7, 25.3, 25.2, 25.1, 25.0, 24.7, 24.0 ppm. The carbon attached to boron was not observed due to quadrupolar relaxation;

^{11}B NMR (128 MHz, $CDCl_3$): δ_B 34.1 ppm.

IR (film): ν_{max} 2977, 2924, 1740, 1470, 1362, 1307, 1217, 1143, 1108, 969, 847 cm^{-1}.

HRMS (ESI^+) m/z calculated for $C_{22}H_{41}O_4B_2$ $[M+H]^+$ 391.3185, found 391.3175.

All recorded spectroscopic data matched those previously reported in the literature.[12]

(*cis,Z*)-5,6-Bis(4,4,5,5-tetramethyl-1,3,2-dioxaborolan-2-yl)cyclooct-1-ene (1v)

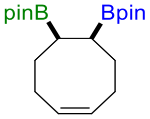

1v

Prepared following a modified **General Procedure A** using 1,5-cyclooctadiene (2.94 mL, 24.0 mmol, 4.00 equiv.), B_2pin_2 (1.53 g, 6.00 mmol, 1.00 equiv.), Cs_2CO_3 (0.30 g, 0.90 mmol, 15 mol%), anhydrous methanol (12.0 mL, 30.0 mmol, 5.00 equiv.) and anhydrous THF (12 mL). Instead of heating to 70 °C, the reaction mixture was heated to 180 °C in the microwave for 20 h. Purification by flash column chromatography (95:5 pentane/diethyl ether) gave the title compound (0.42 g, 29%) as a pale yellow oil.

TLC: R_f = 0.32 (95:5 *n*-hexane/EtOAc, CAM stain).

NMR Spectroscopy (*see spectra*):

^{1}**H NMR** (400 MHz, $CDCl_3$): δ_H 5.67 – 5.52 (m, 2H), 2.33 – 2.19 (m, 2H), 2.11 – 1.92 (m, 4H), 1.61 – 1.40 (m, 4H), 1.22 (s, 24H) ppm;

^{13}**C NMR** (101 MHz, $CDCl_3$): δ_C 130.5, 82.8, 30.2, 26.5, 24.9 ppm. The carbon attached to boron was not observed due to quadrupolar relaxation;

^{11}**B NMR** (128 MHz, $CDCl_3$): δ_B 33.6 ppm.

IR (film): ν_{max} 2976, 2927, 1740, 1370, 1351, 1305, 1217, 1143, 970, 835, 726 cm^{-1}.

HRMS (ESI$^+$) calculated for $C_{20}H_{37}O_4B_2$ $[M+H]^+$ 363.2872, found 363.2870.

All recorded spectroscopic data matched those previously reported in the literature.[12]

2,2'-(7-Methyloct-6-ene-1,2-diyl)bis(4,4,5,5-tetramethyl-1,3,2-dioxaborolane) (1w)

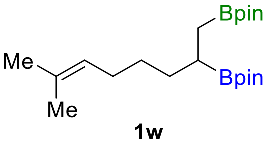

1w

Prepared following **General Procedure A** using 7-methylocta-1,6-diene (1.32 mL, 8.00 mmol, 2.00 equiv.), B_2pin_2 (1.02 g, 4.00 mmol, 1.00 equiv.), Cs_2CO_3 (0.20 g, 0.60 mmol, 15 mol%), anhydrous methanol (0.81 mL, 20 mmol, 5.0 equiv.) and anhydrous THF (20 mL). Purification by flash column chromatography (95:5 pentane/diethyl ether) gave the title compound (0.64 g, 32%) as a colorless oil.

TLC: R_f = 0.24 (95:5 *n*-hexane/EtOAc, CAM stain).

NMR Spectroscopy (*see spectra*):

^{1}**H NMR** (400 MHz, $CDCl_3$): δ_H 5.11 (dddd, *J* = 7.1, 5.7, 2.8, 1.4 Hz, 1H), 1.94 (dd, *J* = 14.0, 7.1 Hz, 2H), 1.66 (d, *J* = 1.3 Hz, 3H), 1.58 (d, *J* = 1.4 Hz, 3H), 1.50 – 1.41 (m, 1H), 1.38 – 1.28 (m, 3H), 1.23 (s, 12H), 1.22 (s, 12H), 1.15 – 1.04 (m, 1H), 0.87 (dd, *J* = 15.8, 9.4 Hz, 1H), 0.79 (dd, *J* = 15.8, 6.0 Hz, 1H) ppm;

UOB Confidential & Sensitive

^{13}C NMR (101 MHz, $CDCl_3$): δ_C 131.1, 125.2, 82.9, 82.9, 33.7, 29.3, 28.5, 25.8, 25.1, 25.0, 24.9, 24.9, 17.8 ppm. The carbon attached to boron was not observed due to quadrupolar relaxation;

^{11}B NMR (128 MHz, $CDCl_3$): δ_B 33.9 ppm.

IR (film): ν_{max} 2977, 2924, 1741, 1370, 1312, 1216, 1142, 968, 846, 672 cm^{-1}.

HRMS (APCI$^+$): m/z calculated for $C_{21}H_{41}B_2O_4$ $[M+H]^+$, 379.3185; found, 379.3197.

2,5-Dimethylterephthalonitrile (4c)

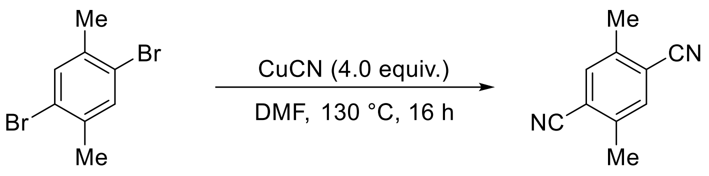

4c

2,5-Dimethylterephthalonitrile (**4c**) was prepared following a modified literature procedure.[20] To a solution of 2,5-dibromo-*p*-xylene (2.64 g, 10.0 mmol) in DMF (40 mL) was added CuCN (3.59 g, 40.0 mmol) and the solution was stirred at 130 °C for 16 hours. After reaction, the mixture was cooled to room temperature, and slowly poured into water. The resulting precipitate was collected by filtration before being dissolved in DCM, washed with water, and dried over $MgSO_4$. Concentration under reduced pressure afforded the title compound (1.30 g, 83%) as a white solid.

TLC: R_f = 0.27 (91:9 *n*-hexane/EtOAc, $KMnO_4$ stain).

NMR Spectroscopy (*see spectra*):

^{1}H NMR (400 MHz, $CDCl_3$): δ_H 7.56 (s, 2H), 2.55 (s, 6H) ppm;

^{13}C NMR (101 MHz, $CDCl_3$): δ_C 140.1, 134.0, 117.1, 116.7, 20.0 ppm;

IR (film): ν_{max} 3039, 2970, 2228, 1740, 1442, 1368, 1229, 1217, 906, 528, 464 cm^{-1}.

All recorded spectroscopic data matched those previously reported in the literature.[20]

2-Methylisonicotinonitrile (4g)

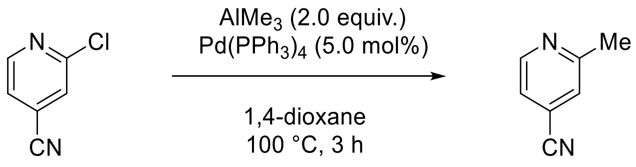

4g

2-Methylisonicotinonitrile (**4g**) was prepared following a modified literature procedure:[21] To a solution of 2-chloroisonicotinonitrile (0.69 g, 5.0 mmol, 1.0 equiv.) and tetrakis(triphenylphosphine)palladium(0) (0.29 g, 0.25 mmol, 5.0 mol%) in 1,4-dioxane (15 mL) was added trimethyl aluminum (5.0 mL, 2.0 M in toluene, 10 mmol, 2.0

equiv.) at room temperature under N_2. The resulting suspension was allowed to stir at 100 °C for 3 hours. The mixture was cooled to 0 °C, and saturated aq. Na_2SO_4 was added dropwise until no more reaction was observed. Then the reaction mixture was filtered over Celite, eluting with diethyl ether. The filtrate was concentrated under reduced pressure and the resulting crude material was purified by flash column chromatography (75:25 pentane/ethyl acetate) to afford the title compound (0.36 g, 61%) as a white solid.

TLC: R_f = 0.24 (80:20 *n*-hexane/EtOAc, $KMnO_4$ stain).

NMR Spectroscopy (*see spectra*):

> **^{1}H NMR** (400 MHz, $CDCl_3$): δ_H 8.68 (dd, *J* = 5.0, 0.9 Hz, 1H), 7.39 (s, 1H), 7.33 (dd, *J* = 5.1, 0.8 Hz, 1H), 2.63 (s, 3H) ppm;
>
> **^{13}C NMR** (101 MHz, $CDCl_3$): δ_C 160.3, 150.3, 124.9, 122.3, 120.7, 116.8, 24.6 ppm.

All recorded spectroscopic data matched those previously reported in the literature.[21]

2-Methoxyisonicotinonitrile (4h)

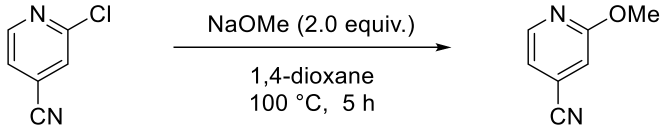

4h

2-Methoxyisonicotinonitrile (**4h**) was prepared following a modified literature procedure:[22] To a solution of 2-chloroisonicotinonitrile (0.69 g, 5.0 mmol, 1.0 equiv.) in 1,4-dioxane (8.0 mL) was added a solution of sodium methoxide in methanol (25 wt. %, 1.84 g, 8.5 mmol). The reaction mixture was heated at 100 °C for 5 hours. The solution was then cooled to room temperature and left overnight in refrigerator. The precipitate was filtered and washed with methanol. The filtrate was concentrated down to about 10 mL, and water (100 mL) was added. The precipitate was collected by filtration and washed with water to afford the title compound (0.36 g, 54%) as a white solid.

TLC: R_f = 0.28 (91:9 *n*-hexane/EtOAc, $KMnO_4$ stain).

NMR Spectroscopy (*see spectra*):

> **^{1}H NMR** (400 MHz, $CDCl_3$): δ_H 8.31 (dd, *J* = 5.2, 0.8 Hz, 1H), 7.06 (dd, *J* = 5.2, 1.3 Hz, 1H), 6.99 (dd, *J* = 1.3, 0.8 Hz, 1H), 3.97 (s, 3H) ppm;
>
> **^{13}C NMR** (101 MHz, $CDCl_3$): δ_C 164.4, 148.6, 122.5, 117.7, 116.6, 114.3, 54.2 ppm;

IR (film): ν_{max} 3028, 2970, 2238, 1740, 1607, 1548, 1368, 1229, 1217, 1042, 986, 886, 831 cm^{-1}.

All recorded spectroscopic data matched those previously reported in the literature.[22]

2-Phenylisonicotinonitrile (4j)

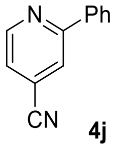

4j

Prepared following **General Procedure C** using 4-cyanopyridine (0.52 g, 5.0 mmol, 1.0 equiv.), phenylboronic acid (0.92 g, 7.5 mmol, 1.5 equiv.), trifluoroacetic acid (0.39 mL, 5.0 mmol, 1.0 equiv.), silver(I)nitrate (0.34 g, 2.0 mmol, 0.40 equiv.), potassium persulfate (7.14 g, 30.0 mmol, 6.00 equiv.), DCM (20 mL) and H_2O (20 mL). Purification by flash column chromatography (90:10 pentane/diethyl ether) gave the title compound (0.44 g, 49%) as a pale-yellow solid.

TLC: R_f = 0.22 (91:9 *n*-hexane/EtOAc, $KMnO_4$ stain).

NMR Spectroscopy (*see spectra*):

^{1}H NMR (400 MHz, $CDCl_3$): δ_H 8.86 (dd, *J* = 5.0, 1.0 Hz, 1H), 8.03 – 7.97 (m, 2H), 7.95 (t, *J* = 1.2 Hz, 1H), 7.55 – 7.48 (m, 3H), 7.45 (dd, *J* = 5.0, 1.4 Hz, 1H) ppm;

^{13}C NMR (101 MHz, $CDCl_3$): δ_C 158.9, 150.8, 137.5, 130.4, 129.2, 127.1, 123.3, 122.2, 121.4, 116.9 ppm;

IR (film): ν_{max} 3073, 3029, 3025, 2236, 1740, 1593, 1544, 1475, 1446, 1389, 1231, 1208, 899, 842, 775 cm^{-1}.

All recorded spectroscopic data matched those previously reported in the literature.[4]

2-(*p*-Tolyl)isonicotinonitrile (4k)

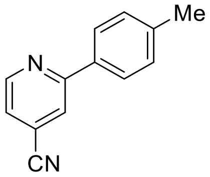

4k

Prepared following **General Procedure C** using 4-cyanopyridine (0.52 g, 5.0 mmol, 1.0 equiv.), *p*-tolylboronic acid (1.02 g, 7.50 mmol, 1.50 equiv.), trifluoroacetic acid (0.39 mL, 5.0 mmol, 1.0 equiv.), silver(I)nitrate (0.34 g, 2.0 mmol, 0.40 equiv.), potassium persulfate (7.14 g, 30.0 mmol, 6.00 equiv.), DCM (20 mL) and H_2O (20 mL). Purification by flash column chromatography (90:10 pentane/diethyl ether) gave the title compound (0.18 g, 19%) as a brown solid.

TLC: R_f = 0.22 (91:9 *n*-hexane/EtOAc, $KMnO_4$ stain).

NMR Spectroscopy (*see spectra*):

^{1}H NMR (400 MHz, $CDCl_3$): δ_H 8.83 (dd, *J* = 5.0, 1.0 Hz, 1H), 7.93 – 7.87 (m, 3H), 7.41 (dd, *J* = 5.0, 1.4 Hz, 1H), 7.32 (dt, *J* = 7.9, 0.7 Hz, 2H), 2.43 (s, 3H) ppm;

^{13}C NMR (101 MHz, $CDCl_3$): δ_C 158.9, 150.7, 140.7, 134.7, 130.0, 127.0, 122.9, 121.9, 121.3, 117.0, 21.5 ppm;

UOB Confidential & Sensitive

IR (film): ν_{max} 3028, 2926, 2235, 1741, 1594, 1543, 1456, 1384, 1229, 1217, 852, 820, 628 cm^{-1}.

All recorded spectroscopic data matched those previously reported in the literature.[23]

2-(3,5-Dimethylphenyl)isonicotinonitrile (4l)

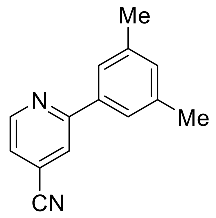

4l

Prepared following **General Procedure C** using 4-cyanopyridine (0.52 g, 5.0 mmol, 1.0 equiv.), (3,5-dimethylphenyl)boronic acid (1.13 g, 7.5 mmol, 1.50 equiv.), trifluoroacetic acid (0.39 mL, 5.0 mmol, 1.0 equiv.), silver(I)nitrate (0.34 g, 2.0 mmol, 0.40 equiv.), potassium persulfate (7.14 g, 30.0 mmol, 6.00 equiv.), DCM (20 mL) and H_2O (20 mL). Purification by flash column chromatography (90:10 pentane/diethyl ether) gave the title compound (0.33 g, 32%) as a brown solid.

TLC: R_f = 0.25 (91:9 *n*-hexane/EtOAc, $KMnO_4$ stain).

NMR Spectroscopy (*see spectra*):

> **^{1}H NMR** (400 MHz, $CDCl_3$): δ_H 8.84 (dd, *J* = 4.9, 1.0 Hz, 1H), 7.92 (t, *J* = 1.2 Hz, 1H), 7.60 (dd, *J* = 1.6, 0.8 Hz, 2H), 7.42 (dd, *J* = 5.0, 1.4 Hz, 1H), 7.13 (s, 1H), 2.41 (d, *J* = 0.8 Hz, 6H) ppm;
>
> **^{13}C NMR** (101 MHz, $CDCl_3$): δ_C 159.3, 150.6, 138.9, 137.4, 132.0, 125.0, 123.1, 122.3, 121.3, 116.9, 21.5 ppm;

IR (film): ν_{max} 2970, 2919, 2238, 1740, 1590, 1546, 1474, 1440, 1378, 1387, 841, 780, 696 cm^{-1}.

HRMS (APCI$^+$): m/z calculated for $C_{14}H_{13}N_2$ [M+H]$^+$, 209.1073; found, 209.1076.

2-(3-Fluorophenyl)isonicotinonitrile (4m)

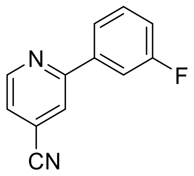

4m

Prepared following **General Procedure C** using 4-cyanopyridine (0.52 g, 5.0 mmol, 1.0 equiv.), (3-fluorophenyl)boronic acid (1.05 g, 7.50 mmol, 1.50 equiv.), trifluoroacetic acid (0.39 mL, 5.0 mmol, 1.0 equiv.), silver(I)nitrate (0.34 g, 2.0 mmol, 0.40 equiv.), potassium persulfate (7.14 g, 30.0 mmol, 6.00 equiv.), DCM (20 mL) and H_2O (20 mL). Purification by flash column chromatography (90:10 pentane/diethyl ether) gave the title compound (0.51 g, 51%) as a white solid.

TLC: R_f = 0.22 (91:9 *n*-hexane/EtOAc, $KMnO_4$ stain).

NMR Spectroscopy (*see spectra*):

^{1}H NMR (400 MHz, $CDCl_3$): δ_H 8.86 (dd, *J* = 4.9, 1.0 Hz, 1H), 7.93 (t, *J* = 1.2 Hz, 1H), 7.80 – 7.73 (m, 2H), 7.52 – 7.43 (m, 2H), 7.18 (tdd, *J* = 8.3, 2.3, 1.4 Hz, 1H) ppm;

^{13}C NMR (101 MHz, $CDCl_3$): δ_C 163.53 (d, $^1J_{C\text{-}F}$ = 246.7 Hz), 157.49 (d, $^4J_{C\text{-}F}$ = 2.8 Hz), 150.87, 139.67 (d, $^3J_{C\text{-}F}$ = 7.7 Hz), 130.77 (d, $^3J_{C\text{-}F}$ = 8.0 Hz), 123.88, 122.60 (d, $^4J_{C\text{-}F}$ = 3.1 Hz), 122.19, 121.57, 117.29 (d, $^2J_{C\text{-}F}$ = 21.2 Hz), 116.66, 114.21 (d, $^2J_{C\text{-}F}$ = 23.3 Hz) ppm;

^{19}F NMR (377 MHz, $CDCl_3$): δ_F -111.94 ppm.

IR (film): ν_{max} 3066, 2238, 1740, 1587, 1547, 1476, 1451, 1395, 1381, 1202, 1158, 918, 880, 840, 787 cm^{-1}.

All recorded spectroscopic data matched those previously reported in the literature.[23]

8-(4,4,5,5-Tetramethyl-1,3,2-dioxaborolan-2-yl)octyl 2,4,6-triisopropylbenzoate (5b)

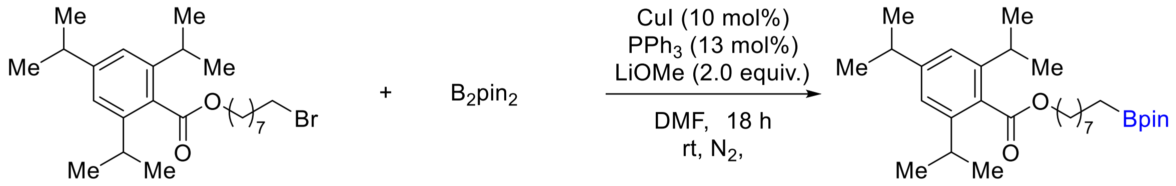

1.0 equiv. 1.5 equiv. **5b**

Compound **5b** was prepared followed a modified literature procedure:[24] To a flame-dried Schlenk flask was added 8-bromooctyl 2,4,6-triisopropylbenzoate (0.88 g, 2.0 mmol, 1.0 equiv.), bis(pinacolato)diboron (0.76 g, 3.0 mmol, 1.5 equiv.), CuI (38 mg, 0.20 mmol, 10 mol%), triphenylphosphine (68 mg, 0.26 mmol, 13 mol%), LiOMe (152 mg, 4.00 mmol, 2.00 equiv.), after which the flask was evacuated and back-filled with nitrogen three times. Subsequently, anhydrous DMF (10 mL) was added *via* syringe and the mixture was stirred for 18 hours at room temperature. After that, water (10 mL) was added to the reaction and the mixture was extracted with EtOAc (3 × 10 mL). The organic layer was dried over Na_2SO_4, filtered, and concentrated under reduced pressure. The resulting crude material was purified by column chromatography (95:5 pentane/diethyl ether) to give the title compound (0.60 g, 62%) as a colorless oil.

TLC: R_f = 0.32 (*n*-hexane/EtOAc 95:5, CAM stain).

NMR Spectroscopy (*see spectra*):

^{1}H NMR (400 MHz, $CDCl_3$): δ_H 7.00 (s, 2H), 4.29 (t, *J* = 6.7 Hz, 2H), 2.86 (dq, *J* = 13.6, 6.9 Hz, 3H), 1.71 (dq, *J* = 8.1, 6.6 Hz, 2H), 1.40 (dd, *J* = 8.9, 4.9 Hz, 4H), 1.34 – 1.18 (m, 36H), 0.76 (t, *J* = 7.8 Hz, 2H) ppm;

^{13}C NMR (101 MHz, $CDCl_3$): δ_C 171.2, 150.1, 144.9, 130.9, 121.0, 83.0, 65.2, 34.6, 32.5, 31.6, 29.5, 29.3, 28.8, 26.2, 25.0, 24.3, 24.1 ppm. The carbon attached to boron was not observed due to quadrupolar relaxation;

^{11}B NMR (128 MHz, $CDCl_3$): δ_B 35.0 ppm.

IR (film): ν_{max} 2969, 2928, 1728, 1462, 1371, 1230, 1217, 1143, 1105, 1075, 968, 876 cm^{-1}.

HRMS (EI$^+$): m/z calculated for $C_{30}H_{52}BO_4$ [M+H]$^+$, 487.3953; found, 487.3974.

4,4,5,5-Tetramethyl-2-(3-phenoxypropyl)-1,3,2-dioxaborolane (5e)

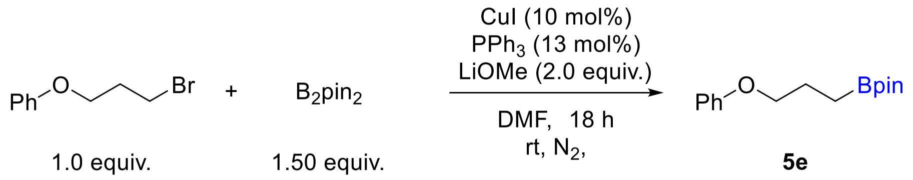

4,4,5,5-Tetramethyl-2-(3-phenoxypropyl)-1,3,2-dioxaborolane (**5e**) was prepared following a modified literature procedure:[24] To a solution of bis(pinacolato)diboron (1.9 g, 0.75 mmol, 1.5 equiv.), CuI (96 mg, 0.50 mmol, 10 mol %), PPh_3 (0.17 g, 0.65 mmol, 13 mol%), and LiOMe (0.38 g, 10 mmol, 2.0 equiv.) in DMF (50 mL) was added (3-bromopropoxy)benzene (1.08 g, 5.00 mmol, 1.00 equiv.) at room temperature under N_2. The resulting suspension was allowed to stir vigorously at this temperature for 18 hours. After that, water (50 mL) was added to the reaction and the mixture was extracted with EtOAc (3 × 50 mL). The organic layer was dried over Na_2SO_4, filtered, and concentrated under reduced pressure. The resulting crude material was purified by column chromatography (95:5 pentane/diethyl ether) to give the title compound (0.89 g, 68%) as a colorless oil.

TLC: R_f = 0.47 (*n*-hexane/EtOAc 91:9, CAM stain).

NMR Spectroscopy (*see spectra*):

^{1}**H NMR** (400 MHz, $CDCl_3$): δ_H 7.30 – 7.21 (m, 2H), 6.95 – 6.86 (m, 3H), 3.93 (t, *J* = 6.7 Hz, 2H), 1.95 – 1.83 (m, 2H), 1.24 (s, 12H), 0.92 (t, *J* = 7.8 Hz, 2H) ppm;

^{13}**C NMR** (101 MHz, $CDCl_3$): δ_C 159.3, 129.5, 120.5, 114.8, 83.2, 69.7, 25.0, 23.9 ppm. The carbon attached to boron was not observed due to quadrupolar relaxation;

^{11}**B NMR** (128 MHz, $CDCl_3$): δ_B 34.6 ppm.

All recorded spectroscopic data matched those previously reported in the literature.[25]

tert-Butyl 3-(4,4,5,5-tetramethyl-1,3,2-dioxaborolan-2-yl)butanoate (5o)

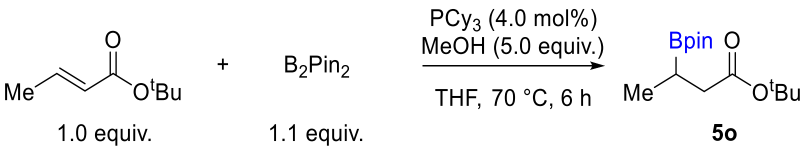

tert-Butyl 3-(4,4,5,5-tetramethyl-1,3,2-dioxaborolan-2-yl)butanoate (**5o**) was prepared following a literature procedure:[26] To a flame-dried Schlenk flask was added bis(pinacolato)diboron (1.4 g, 5.5 mmol, 1.1 equiv.), and tricyclohexylphosphine (56 mg, 0.20 mmol, 4.0 mol%), after which the flask was evacuated and back-filled with nitrogen three times. Subsequently, anhydrous THF (20 mL) was added via syringe and the mixture was

UOB Confidential & Sensitive

stirred for 10 minutes at room temperature to dissolve the phosphine and boron reagents completely. Then *tert*-butyl (*E*)-but-2-enoate (0.71 g, 5.0 mmol, 1.0 equiv.) and MeOH (1.3 mL, 25 mmol, 5.0 equiv.) were added, and the reaction mixture was stirred at 70 °C for 6 hours. After that, the reaction was cooled to room temperature, and all volatiles were removed under reduced pressure. The resulting crude material was purified by column chromatography (95:5 pentane/diethyl ether) to give the title compound (1.08 g, 80%) as a colorless oil.

TLC: R_f = 0.45 (91:9 *n*-hexane/EtOAc, CAM stain).

NMR Spectroscopy (*see spectra*):

^{1}H NMR (400 MHz, $CDCl_3$): δ_H 2.34 (dd, *J* = 16.5, 7.7 Hz, 1H), 2.25 (dd, *J* = 16.5, 7.0 Hz, 1H), 1.43 (s, 9H), 1.37 – 1.28 (m, 1H), 1.23 (s, 6H), 1.23 (s, 6H), 0.98 (d, *J* = 7.5 Hz, 3H) ppm;

^{13}C NMR (101 MHz, $CDCl_3$): δ_C 173.4, 83.2, 80.0, 39.0, 28.3, 24.9, 24.8, 15.1 ppm. The carbon attached to boron was not observed due to quadrupolar relaxation;

^{11}B NMR (128 MHz, $CDCl_3$): δ_B 34.0 ppm.

IR (film): ν_{max} 2976, 2933, 1726, 1462, 1367, 1316, 1215, 1138, 970, 1009, 861, 670 cm^{-1}.

All recorded spectroscopic data matched those previously reported in the literature.[26]

exo-2-(Bicyclo[2.2.1]heptan-2-yl)-4,4,5,5-tetramethyl-1,3,2-dioxaborolane (5q)

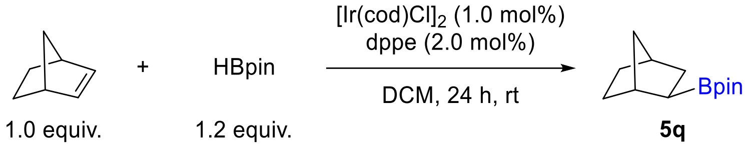

exo-2-(Bicyclo[2.2.1]heptan-2-yl)-4,4,5,5-tetramethyl-1,3,2-dioxaborolane (**5q**) was prepared following a modified literature procedure:[27] To a solution of norbornene (0.94 g, 10 mmol, 1.0 equiv.), bis(1,5-cyclooctadiene)diiridium(I) dichloride (67 mg, 0.10 mmol, 1.0 mol%) and 1,2-bis(diphenylphosphino)ethane (80 mg, 0.20 mmol, 2.0 mol%) in DCM (15 mL) was added pinacolborane (1.5 g, 12 mmol, 1.7 ml, 1.2 equiv.) at ambient temperature. After stirring for 24 h, the reaction mixture was quenched with methanol (2.0 mL) and water (10 mL) was added. The organic layer was separated, and the aqueous layer was extracted with DCM (3 × 20 mL). The organic layers were combined, washed with brine (20 mL), dried over Na_2SO_4, and concentrated under reduced pressure. The resulting crude material was purified by column chromatography (97:3 pentane/diethyl ether) to give the title compound (1.84 g, 83%) as a colorless oil.

TLC: R_f = 0.50 (*n*-hexane/EtOAc 95:5, CAM stain).

NMR Spectroscopy (*see spectra*):

^{1}H NMR (400 MHz, $CDCl_3$): δ_H 2.29 – 2.25 (m, 1H), 2.25 – 2.18 (m, 1H), 1.58 – 1.42 (m, 3H), 1.34 (ddd, *J* = 11.8, 9.8, 2.3 Hz, 1H), 1.29 – 1.11 (m, 17H), 0.87 (ddd, *J* = 10.3, 6.2, 1.7 Hz, 1H) ppm;

^{13}C NMR (101 MHz, $CDCl_3$): δ_C 82.9, 38.9, 38.3, 36.8, 32.4, 32.3, 29.4, 24.9 ppm. The carbon attached to boron was not observed due to quadrupolar relaxation;

^{11}B NMR (128 MHz, $CDCl_3$): δ_B 35.4 ppm.

IR (film): ν_{max} 2973, 2946, 2868, 1740, 1451, 1407, 1370, 1309, 1225, 1145, 1108, 980, 860 cm^{-1}.

All recorded spectroscopic data matched those previously reported in the literature.[27]

2-{(3*S*,5*R*,6*R*,8*R*,9*S*,10*S*,13*R*,14*S*,17*R*)-3-Chloro-10,13-dimethyl-17-[(*R*)-5-methylhexan-2-yl]hexadecahydro-1*H*-cyclopenta[a]phenanthren-6-yl}-4,4,5,5-tetramethyl-1,3,2-dioxaborolane (5r)

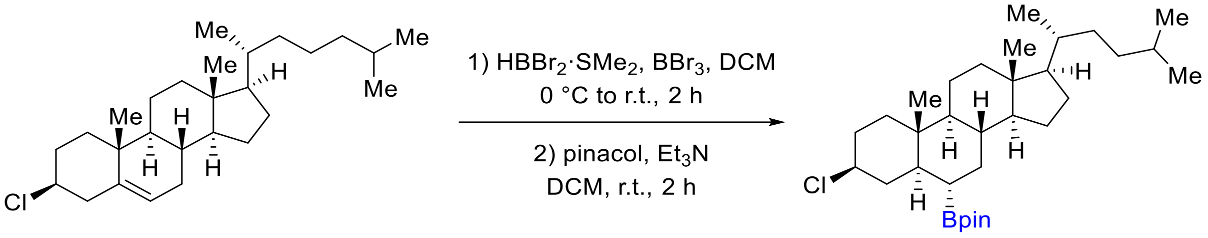

5r

Compound **5r** was prepared according to a reported literature:[28] To a solution of cholesteryl chloride (4.05 g, 10.0 mmol, 1.00 equiv.) in DCM was added dropwise $HBBr_2{\cdot}SMe_2$ (1.0 M in DCM, 10.0 mL, 10.0 mmol, 1.00 equiv.) followed by BBr_3 (1.0 M in DCM, 10.0 mL, 10.0 mmol, 1.00 equiv.) at 0 °C. The reaction mixture was removed from the ice bath and stirred at room temperature for 2 hours. Then, a preformed solution of pinacol (3.55 g, 30.0 mmol, 3.00 equiv.) in triethylamine (11.2 mL) was added to the reaction and the resulting solution was vigorously stirred at room temperature for another 2 hours. After that, the reaction mixture was quenched with aqueous $NaHSO_4$ (20 mL, 1.0 M) and water (10 mL) was added. The organic layer was separated, and the aqueous layer was extracted with DCM (3 × 40 mL). The organic layers were combined, washed with brine (50 mL), dried over Na_2SO_4, and concentrated under reduced pressure. The resulting crude material was recrystallization from *i*-PrOH to give the title compound (3.74 g, 72%, >99:1 *d.r.*) as a white solid.

TLC: R_f = 0.45 (98:2 *n*-hexane/EtOAc, CAM stain).

NMR Spectroscopy (*see spectra*):

^{1}H NMR (400 MHz, $CDCl_3$): δ_H 3.90 (tt, *J* = 11.9, 4.8 Hz, 1H), 2.05 – 1.90 (m, 2H), 1.87 – 1.69 (m, 4H), 1.66 – 1.42 (m, 6H), 1.40 – 1.19 (m, 19H), 1.17 – 0.93 (m, 9H), 0.89 (d, *J* = 6.5 Hz, 3H), 0.87 (d, *J* = 1.9 Hz, 3H), 0.85 (d, *J* = 1.9 Hz, 3H), 0.83 (s, 3H), 0.74 – 0.62 (m, 1H), 0.63 (s, 3H) ppm;

^{13}C NMR (100 MHz, $CDCl_3$): δ_C 83.2, 60.5, 56.4, 56.4, 53.9, 47.4, 42.8, 40.1, 39.7, 39.2, 38.6, 36.3, 35.9, 35.7, 35.3, 33.6, 33.2, 28.4, 28.2, 25.0, 24.8, 24.3, 24.0, 23.0, 22.7, 21.3, 18.8, 12.6, 12.2 ppm. The carbon attached to boron was not observed due to quadrupolar relaxation;

IR (film): ν_{max} 2941, 2868, 1740, 1467, 1375, 1321, 1229, 1217, 1145, 969, 850, 527 cm^{-1}.

All recorded spectroscopic data matched those previously reported in the literature.[29]

2.5. Product Characterisation

4-[4,4-Dimethyl-1-(4,4,5,5-tetramethyl-1,3,2-dioxaborolan-2-yl)pentan-2-yl]benzonitrile (3aa)

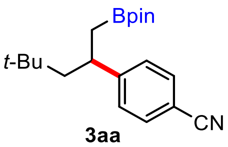

3aa

Prepared following **General Procedure D**, using 2,2'-(4,4-dimethylpentane-1,2-diyl)bis(4,4,5,5-tetramethyl-1,3,2-dioxaborolane) (**1a**) (106 mg, 0.300 mmol, 1.50 equiv.), 4-bromo-*N,N*-dimethylaniline (64 mg, 0.32 mmol, 1.6 equiv.), *tert*-butyllithium (1.7 M in pentane, 376 µL, 0.640 mmol, 3.20 equiv.) and terephthalonitrile (**4a**) (25.6 mg, 0.200 mmol, 1.00 equiv.). Purification by flash column chromatography (91:9 pentane/diethyl ether) gave boronic ester **3aa** (54.9 mg, 84%) as a colorless oil.

R*f* = 0.34 (91:9 *n*-hexane/EtOAc, CAM stain)

NMR Spectroscopy (*see spectra*):

> **^{1}H NMR** (400 MHz, $CDCl_3$): δ_H 7.52 (d, J = 8.4 Hz, 2H), 7.34 (d, J = 8.4 Hz, 2H), 3.03 (tdd, J = 9.2, 7.0, 3.4 Hz, 1H), 1.72 (dd, J = 14.2, 9.5 Hz, 1H), 1.55 (dd, J = 14.2, 3.4 Hz, 1H), 1.15 (dd, J = 15.4, 7.0 Hz, 1H), 1.11 – 1.00 (m, 13H), 0.74 (s, 9H) ppm;
>
> **^{13}C NMR** (101 MHz, $CDCl_3$): δ_C 155.2, 132.1, 128.7, 119.4, 109.5, 83.3, 52.9, 38.8, 31.6, 30.3, 24.9, 24.8 ppm. The carbon attached to boron was not observed due to quadrupolar relaxation;
>
> **^{11}B NMR** (128 MHz, $CDCl_3$): δ_B 33.6 ppm.

IR (film): ν_{max} 2953, 2227,1740,1607,1371, 1366, 1325, 1217, 1144, 967, 649 cm^{-1}.

HRMS (ESI^+): m/z calculated for $C_{20}H_{30}BNNaO_2$ $[M+Na]^+$, 350.2265; found, 350.2282.

4-[1-(4,4,5,5-Tetramethyl-1,3,2-dioxaborolan-2-yl)pentan-2-yl]benzonitrile (3ba)

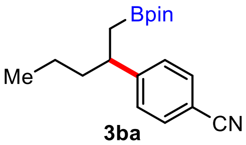

3ba

Prepared following **General Procedure D**, using 2,2'-(pentane-1,2-diyl)bis(4,4,5,5-tetramethyl-1,3,2-dioxaborolane) (**1b**) (97 mg, 0.30 mmol, 1.5 equiv.), 4-bromo-*N,N*-dimethylaniline (64 mg, 0.32 mmol, 1.6 equiv.), *tert*-butyllithium (1.7 M in pentane, 376 µL, 0.640 mmol, 3.20 equiv.) and terephthalonitrile (**4a**) (25.6 mg, 0.200 mmol, 1.00 equiv.). Purification by flash column chromatography (91:9 pentane/diethyl ether) gave boronic ester **3ba** (40.1 mg, 67%) as a colorless oil.

R*f* = 0.30 (91:9 *n*-hexane/EtOAc, CAM stain)

NMR Spectroscopy (*see spectra*):

^{1}H NMR (400 MHz, $CDCl_3$): δ_H 7.54 (d, J = 8.3 Hz, 2H), 7.29 (d, J = 8.3 Hz, 2H), 2.89 (tt, J = 8.9, 6.4 Hz, 1H), 1.64 – 1.49 (m, 2H), 1.28 – 1.16 (m, 2H), 1.15 – 1.01 (m, 14H), 0.84 (t, J = 7.3 Hz, 3H) ppm;

^{13}C NMR (101 MHz, $CDCl_3$): δ_C 153.6, 132.1, 128.5, 119.4, 109.6, 83.3, 41.6, 41.5, 24.8, 24.8, 20.8, 14.1 ppm. The carbon attached to boron was not observed due to quadrupolar relaxation;

^{11}B NMR (128 MHz, $CDCl_3$): δ_B 33.0 ppm.

IR (film): ν_{max} 2973, 2929, 2227, 1740, 1607, 1370, 1326, 1216, 1144, 968, 847, 598 cm^{-1}.

HRMS (EI^+): m/z calculated for $C_{17}H_{23}BNO_2$ $[M-CH_3]^+$, 284.1816; found, 284.1816.

4-[4-Methyl-1-(4,4,5,5-tetramethyl-1,3,2-dioxaborolan-2-yl)pentan-2-yl]benzonitrile (3ca)

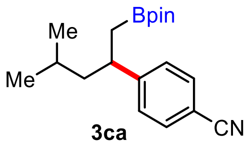

3ca

Prepared following **General Procedure D**, using 2,2'-(4-methylpentane-1,2-diyl)bis(4,4,5,5-tetramethyl-1,3,2-dioxaborolane) (**1c**) (116 mg, 0.300 mmol, 1.50 equiv.), 4-bromo-*N,N*-dimethylaniline (64 mg, 0.32 mmol, 1.6 equiv.), *tert*-butyllithium (1.7 M in pentane, 376 µL, 0.640 mmol, 3.20 equiv.) and terephthalonitrile (**4a**) (25.6 mg, 0.200 mmol, 1.00 equiv.). Purification by flash column chromatography (91:9 pentane/diethyl ether) gave boronic ester **3ca** (38.2 mg, 61%) as a colorless oil.

R_f = 0.30 (91:9 *n*-hexane/EtOAc, CAM stain)

NMR Spectroscopy (*see spectra*):

^{1}H NMR (400 MHz, $CDCl_3$): δ_H 7.54 (d, J = 8.4 Hz, 2H), 7.30 (d, J = 8.4 Hz, 2H), 2.97 (tt, J = 9.3, 6.1 Hz, 1H), 1.53 (ddd, J = 13.4, 9.5, 5.2 Hz, 1H), 1.41 (ddd, J = 13.5, 8.6, 5.5 Hz, 1H), 1.31 – 1.21 (m, 1H), 1.16 (dd, J = 15.4, 6.7 Hz, 1H), 1.09 (s, 6H), 1.08 (s, 6H), 1.07 – 1.01 (m, 1H), 0.86 (d, J = 6.5 Hz, 3H), 0.80 (d, J = 6.5 Hz, 3H) ppm;

^{13}C NMR (101 MHz, $CDCl_3$): δ_C 153.6, 132.2, 128.5, 119.4, 109.6, 83.3, 48.7, 39.6, 25.7, 24.8, 24.8, 23.4, 22.1 ppm. The carbon attached to boron was not observed due to quadrupolar relaxation;

^{11}B NMR (128 MHz, $CDCl_3$) δ_B 32.5 ppm.

IR (film): ν_{max} 2956, 2227, 1740, 1607, 1504, 1467, 1369, 1324, 1143, 967, 912, 849, 732, 568 cm^{-1}.

HRMS (ESI^+): m/z calculated for $C_{19}H_{28}BNNaO_2$ $[M+Na]^+$, 336.2109; found, 336.2116.

UOB Confidential & Sensitive

4-[1-Cyclohexyl-2-(4,4,5,5-tetramethyl-1,3,2-dioxaborolan-2-yl)ethyl]benzonitrile (3da)

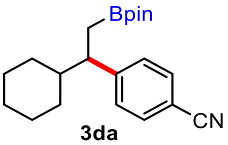

3da

Prepared following **General Procedure D**, using 2,2'-(1-cyclohexylethane-1,2-diyl)bis(4,4,5,5-tetramethyl-1,3,2-dioxaborolane) (**1d**) (109 mg, 0.300 mmol, 1.50 equiv.), 4-bromo-*N,N*-dimethylaniline (64 mg, 0.32 mmol, 1.6 equiv.), *tert*-butyllithium (1.7 M in pentane, 376 µL, 0.640 mmol, 3.20 equiv.) and terephthalonitrile (**4a**) (25.6 mg, 0.200 mmol, 1.00 equiv.). Purification by flash column chromatography (91:9 pentane/diethyl ether) gave boronic ester **3da** (50.9 mg, 75%) as a colorless oil.

R_f = 0.29 (91:9 *n*-hexane/EtOAc, CAM stain)

NMR Spectroscopy (*see spectra*):

^{1}H NMR (400 MHz, $CDCl_3$): δ_H 7.53 (d, *J* = 8.3 Hz, 2H), 7.25 (d, *J* = 8.3 Hz, 2H), 2.66 (ddd, *J* = 10.8, 7.7, 5.5 Hz, 1H), 1.84 (dt, *J* = 12.1, 3.3 Hz, 1H), 1.78 – 1.68 (m, 1H), 1.64 – 1.53 (m, 2H), 1.41 – 1.29 (m, 3H), 1.21 – 1.15 (m, 1H), 1.11 – 1.03 (m, 3H), 1.03 (s, 6H), 0.99 (s, 6H), 0.89 (ddd, *J* = 25.3, 11.9, 3.6 Hz, 1H), 0.80 – 0.66 (m, 1H) ppm;

^{13}C NMR (101 MHz, $CDCl_3$): δ_C 152.5, 131.8, 129.3, 119.4, 109.5, 83.2, 47.9, 45.0, 31.1, 30.9, 26.6, 26.5, 24.8, 24.6 ppm. The carbon attached to boron was not observed due to quadrupolar relaxation;

^{11}B NMR (128 MHz, $CDCl_3$) δ_B 34.0 ppm.

IR (film): ν_{max} 2977, 2925, 2852, 2227, 1740, 1607, 1448, 1365, 1324, 1215, 1143, 967, 846, 732, 578 cm^{-1}.

HRMS (ESI$^+$): m/z calculated for $C_{21}H_{30}BNNaO_2$ [M+Na]$^+$, 362.2266; found, 362.2281.

4-[3,3-Dimethyl-1-(4,4,5,5-tetramethyl-1,3,2-dioxaborolan-2-yl)butan-2-yl]benzonitrile (3ea)

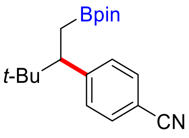

3ea

Prepared following **General Procedure D**, using 2,2'-(3,3-dimethylbutane-1,2-diyl)bis(4,4,5,5-tetramethyl-1,3,2-dioxaborolane) (**1e**) (116 mg, 0.300 mmol, 1.50 equiv.), 4-bromo-*N,N*-dimethylaniline (64 mg, 0.32 mmol, 1.6 equiv.), *tert*-butyllithium (1.7 M in pentane, 376 µL, 0.640 mmol, 3.20 equiv.) and terephthalonitrile (**4a**) (25.6 mg, 0.200 mmol, 1.00 equiv.). Purification by flash column chromatography (91:9 pentane/diethyl ether) gave boronic ester **3ea** (32.0 mg, 51%) as a colorless oil.

R_f = 0.32 (91:9 *n*-hexane/EtOAc, CAM stain)

NMR Spectroscopy (*see spectra*):

^{1}**H NMR** (400 MHz, $CDCl_3$): δ_H 7.52 (d, J = 8.4 Hz, 2H), 7.27 (d, J = 8.4 Hz, 2H), 2.76 (dd, J = 10.0, 7.1 Hz, 1H), 1.22 (d, J = 3.0 Hz, 2H), 0.98 (s, 6H), 0.89 (s, 6H), 0.84 (s, 9H) ppm;

^{13}**C NMR** (101 MHz, $CDCl_3$): δ_C 150.7, 131.2, 130.5, 119.3, 109.6, 83.1, 52.0, 34.3, 27.7, 24.7, 24.3 ppm. The carbon attached to boron was not observed due to quadrupolar relaxation;

^{11}**B NMR** (128 MHz, $CDCl_3$) δ_B 32.1 ppm.

IR (film): ν_{max} 2971, 2871, 2227, 1740, 1607, 1469, 1370, 1328, 1232, 1145, 968, 847, 569 cm^{-1}.

HRMS (ESI$^+$): m/z calculated for $C_{19}H_{28}BNNaO_2$ [M+Na]$^+$, 336.2109; found, 336.2124.

4-[1-(4,4,5,5-Tetramethyl-1,3,2-dioxaborolan-2-yl)-3-(trimethylsilyl)propan-2-yl]benzonitrile (3fa)

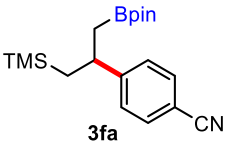

3fa

Prepared following **General Procedure D**, using [2,3-bis(4,4,5,5-tetramethyl-1,3,2-dioxaborolan-2-yl)propyl]trimethylsilane (**1f**) (116 mg, 0.300 mmol, 1.50 equiv.), 4-bromo-*N,N*-dimethylaniline (64 mg, 0.32 mmol, 1.6 equiv.), *tert*-butyllithium (1.7 M in pentane, 376 µL, 0.640 mmol, 3.20 equiv.) and terephthalonitrile (**4a**) (25.6 mg, 0.200 mmol, 1.00 equiv.). Purification by flash column chromatography (92:8 pentane/diethyl ether) gave boronic ester **3fa** (48.8 mg, 71%) as a colorless oil.

R*f* = 0.47 (10:1 *n*-hexane/EtOAc, CAM stain)

NMR Spectroscopy (*see spectra*):

^{1}**H NMR** (400 MHz, $CDCl_3$): δ_H 7.52 (d, J = 8.4 Hz, 2H), 7.32 (d, J = 8.4 Hz, 2H), 3.05 (tt, J = 8.4, 6.8 Hz, 1H), 1.18 (qd, J = 15.4, 7.9 Hz, 2H), 1.07 (s, 6H), 1.07 (s, 6H), 1.00 – 0.95 (m, 2H), -0.19 (s, 9H) ppm;

^{13}**C NMR** (101 MHz, $CDCl_3$): δ_C 155.1, 132.1, 128.2, 119.4, 109.6, 83.2, 38.4, 28.1, 24.8, -0.9 ppm. The carbon attached to boron was not observed due to quadrupolar relaxation;

^{11}**B NMR** (128 MHz, $CDCl_3$) δ_B 31.2 ppm.

IR (film): ν_{max} 2978, 2227, 1740, 1607, 1366, 1325, 1248, 1143, 967, 888, 863, 834, 733, 692 cm^{-1}.

HRMS (ESI$^+$): m/z calculated for $C_{19}H_{30}BNNaO_2Si$ [M+Na]$^+$, 366.2035; found, 366.2053.

4-[6-Chloro-1-(4,4,5,5-tetramethyl-1,3,2-dioxaborolan-2-yl)hexan-2-yl]benzonitrile (3ga)

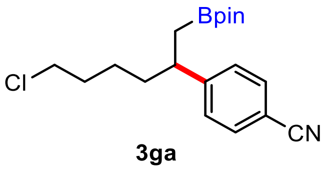

3ga

Prepared following **General Procedure D**, using 2,2'-(6-chlorohexane-1,2-diyl)bis(4,4,5,5-tetramethyl-1,3,2-dioxaborolane) (**1g**) (112 mg, 0.300 mmol, 1.50 equiv.), 4-bromo-*N,N*-dimethylaniline (64 mg, 0.32 mmol, 1.6 equiv.), *tert*-butyllithium (1.7 M in pentane, 376 µL, 0.640 mmol, 3.20 equiv.) and terephthalonitrile (**4a**) (25.6 mg, 0.200 mmol, 1.00 equiv.). Purification by flash column chromatography (92:8 pentane/diethyl ether) gave boronic ester **3ga** (36.2 mg, 52%) as a colorless oil.

R*f* = 0.41 (10:1 EtOAc/*n*-hexane, CAM stain)

NMR Spectroscopy (*see spectra*):

^{1}**H NMR** (400 MHz, $CDCl_3$): δ_H 7.55 (d, *J* = 8.3 Hz, 2H), 7.29 (d, *J* = 8.3 Hz, 2H), 3.46 (td, *J* = 6.7, 2.0 Hz, 2H), 2.88 (tt, *J* = 8.8, 6.3 Hz, 1H), 1.81 – 1.52 (m, 4H), 1.40 – 1.27 (m, 1H), 1.27 – 1.04 (m, 15H) ppm;

^{13}**C NMR** (101 MHz, $CDCl_3$): δ_C 153.0, 132.2, 128.4, 119.3, 109.9, 83.4, 44.8, 41.7, 38.2, 32.5, 24.9, 24.8, 24.8 ppm. The carbon attached to boron was not observed due to quadrupolar relaxation;

^{11}**B NMR** (128 MHz, $CDCl_3$) δ_B 33.1 ppm.

IR (film): ν_{max} 2932, 2978, 2227, 1740, 1607, 1505, 1370, 1324, 1215, 1142, 967, 912, 846, 731, 648 cm^{-1}.

HRMS (ESI$^+$): m/z calculated for $C_{19}H_{27}BClNNaO_2$ [M+Na]$^+$, 370.1719; found, 370.1720.

***tert*-Butyl 6-(4-cyanophenyl)-7-(4,4,5,5-tetramethyl-1,3,2-dioxaborolan-2-yl)heptanoate (3ha)**

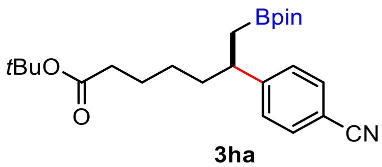

3ha

Prepared following **General Procedure D**, using *tert*-butyl 6,7-bis(4,4,5,5-tetramethyl-1,3,2-dioxaborolan-2-yl)heptanoate (**1h**) (132 mg, 0.300 mmol, 1.50 equiv.), 4-bromo-*N,N*-dimethylaniline (64 mg, 0.32 mmol, 1.6 equiv.), *tert*-butyllithium (1.7 M in pentane, 376 µL, 0.640 mmol, 3.20 equiv.) and terephthalonitrile (**4a**) (25.6 mg, 0.200 mmol, 1.00 equiv.). Purification by flash column chromatography (90:10 pentane/diethyl ether) gave boronic ester **3ha** (38.9 mg, 47%) as a colorless oil.

R*f* = 0.29 (91:9 EtOAc/pentane, CAM stain)

NMR Spectroscopy (*see spectra*):

^{1}**H NMR** (400 MHz, $CDCl_3$): δ_H 7.54 (d, *J* = 8.2 Hz, 2H), 7.28 (d, *J* = 8.2 Hz, 2H), 2.87 (tt, *J* = 8.9, 6.2 Hz, 1H), 2.12 (td, *J* = 7.4, 1.1 Hz, 2H), 1.69 – 1.44 (m, 4H), 1.39 (s, 9H), 1.28 – 1.00 (m, 16H) ppm;

^{13}**C NMR** (101 MHz, $CDCl_3$): δ_C 173.1, 153.3, 132.2, 128.4, 119.3, 109.7, 83.3, 80.1, 41.7, 38.8, 35.5, 28.2, 27.1, 25.1, 24.8, 24.8 ppm. The carbon attached to boron was not observed due to quadrupolar relaxation;

^{11}**B NMR** (128 MHz, $CDCl_3$): δ_B 34.2 ppm.

IR (film): ν_{max} 3478, 2951, 1740, 1604, 1368, 1313, 1217, 1139, 884, 798, 710, 523 cm^{-1}.

HRMS (ESI$^+$): m/z calculated for $C_{24}H_{36}BNNaO_4$ [M+Na]$^+$, 436.2634; found, 436.2647.

4-[4-Phenyl-1-(4,4,5,5-tetramethyl-1,3,2-dioxaborolan-2-yl)butan-2-yl]benzonitrile (3ia)

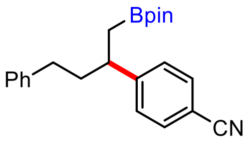

3ia

Prepared following **General Procedure D**, using 2,2'-(4-phenylbutane-1,2-diyl)bis(4,4,5,5-tetramethyl-1,3,2-dioxaborolane) (**1i**) (116 mg, 0.300 mmol, 1.50 equiv.), 4-bromo-*N,N*-dimethylaniline (64 mg, 0.32 mmol, 1.6 equiv.), *tert*-butyllithium (1.7 M in pentane, 376 µL, 0.640 mmol, 3.20 equiv.) and terephthalonitrile (**4a**) (25.6 mg, 0.200 mmol, 1.00 equiv.). Purification by flash column chromatography (86:14 *n*-hexane/EtOAc) gave boronic ester **3ia** (33.3 mg, 46%) as a colorless oil.

R$_f$ = 0.46 (83:17 *n*-hexane/EtOAc, CAM stain)

NMR Spectroscopy (*see spectra*):

^{1}H NMR (400 MHz, $CDCl_3$): δ_H 7.58 (d, *J* = 8.3 Hz, 2H), 7.33 (d, *J* = 8.3 Hz, 2H), 7.28 – 7.21 (m, 2H), 7.19 – 7.13 (m, 1H), 7.10 – 7.05 (m, 2H), 2.94 (tdd, *J* = 9.0, 6.6, 5.4 Hz, 1H), 2.44 (ddd, *J* = 9.7, 6.6, 3.2 Hz, 2H), 2.05 – 1.85 (m, 2H), 1.25 (dd, *J* = 15.5, 6.6 Hz, 1H), 1.18 – 1.12 (m, 1H), 1.10 (s, 6H), 1.09 (s, 6H) ppm;

^{13}C NMR (101 MHz, $CDCl_3$): δ_C 153.0, 142.0, 132.3, 128.6, 128.5, 128.4, 126.0, 119.3, 109.9, 83.3, 41.5, 40.7, 33.9, 24.8, 24.8 ppm. The carbon attached to boron was not observed due to quadrupolar relaxation;

^{11}B NMR (128 MHz, $CDCl_3$) δ_B 34.5 ppm.

IR (film): ν_{max} 2976, 2930, 2227, 1740, 1606, 1370, 1325, 1216, 1143, 967, 846, 700, 565 cm^{-1}.

HRMS (EI$^+$): m/z calculated for $C_{22}H_{25}BNO_2$ [M-CH_3]$^+$, 346.1973; found, 346.1969.

4-[1-Hydroxy-4-(naphthalen-2-yl)butan-2-yl]benzonitrile (3ja)

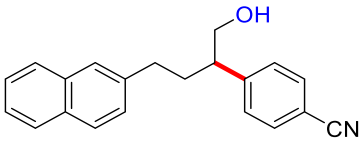

3ja

Prepared following **General Procedure D**, using 2,2'-(4-(naphthalen-2-yl)butane-1,2-diyl)bis(4,4,5,5-tetramethyl-1,3,2-dioxaborolane) (**1j**) (131 mg, 0.300 mmol, 1.50 equiv.), 4-bromo-*N,N*-dimethylaniline (64 mg, 0.32 mmol, 1.6 equiv.), *tert*-butyllithium (1.7 M in pentane, 376 µL, 0.640 mmol, 3.20 equiv.) and terephthalonitrile (**4a**) (25.6 mg, 0.200 mmol, 1.00 equiv.). The crude material was oxidized following the

procedure mentioned above. Purification by flash column chromatography (75:25 *n*-hexane/EtOAc) gave the title compound **3ja** (25.3 mg, 42%) as a colorless oil.

R*f* = 0.48 (67:33 *n*-hexane/EtOAc, $KMnO_4$ stain)

NMR Spectroscopy (*see spectra*):

^{1}H NMR (400 MHz, $CDCl_3$): δ_H 7.80 (dd, *J* = 7.9, 1.6 Hz, 1H), 7.77 – 7.73 (m, 2H), 7.65 (d, *J* = 8.3 Hz, 2H), 7.51 (s, 1H), 7.49 – 7.40 (m, 2H), 7.36 (d, *J* = 8.3 Hz, 2H), 7.24 (dd, *J* = 8.5, 1.8 Hz, 1H), 3.89 – 3.72 (m, 2H), 2.91 (ddt, *J* = 10.6, 6.9, 5.4 Hz, 1H), 2.76 – 2.59 (m, 2H), 2.29 – 2.15 (m, 1H), 2.08 – 1.96 (m, 1H), 1.43 (brs, 1H) ppm;

^{13}C NMR (101 MHz, $CDCl_3$): δ_C 148.3, 138.9, 133.7, 132.6, 132.2, 129.2, 128.2, 127.8, 127.5, 127.1, 126.6, 126.2, 125.5, 119.0, 110.9, 67.1, 48.2, 33.6, 33.2 ppm;

IR (film): ν_{max} 3440, 3051, 2933, 2864, 2227, 1740, 1607, 1505, 1416, 1367, 1217, 1038, 836 819, 749 cm^{-1}.

HRMS (ESI$^+$): m/z calculated for $C_{21}H_{19}NNaO$ [M+Na]$^+$, 324.1359; found, 324.1375.

4-(1-Hydroxy-6-phenylhexan-2-yl)benzonitrile (3ka)

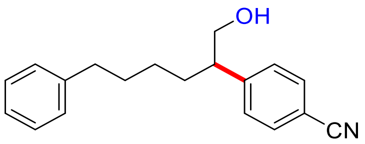

3ka

Prepared following **General Procedure D**, using 2,2'-(6-phenylhexane-1,2-diyl)bis(4,4,5,5-tetramethyl-1,3,2-dioxaborolane) (**1k**) (124 mg, 0.300 mmol, 1.50 equiv.), 4-bromo-*N,N*-dimethylaniline (64 mg, 0.32 mmol, 1.6 equiv.), *tert*-butyllithium (1.7 M in pentane, 376 µL, 0.640 mmol, 3.20 equiv.) and terephthalonitrile (**4a**) (25.6 mg, 0.200 mmol, 1.00 equiv.). The crude material was oxidized following the procedure mentioned above. Purification by flash column chromatography (90:10 DCM/Et_2O) gave the title compound **3ka** (29.1 mg, 52%) as a colorless oil.

R*f* = 0.58 (90:10 DCM/Et_2O, $KMnO_4$ stain)

NMR Spectroscopy (*see spectra*):

^{1}H NMR (400 MHz, $CDCl_3$): δ_H 7.61 (d, *J* = 8.3 Hz, 2H), 7.31 (d, *J* = 8.3 Hz, 2H), 7.28 – 7.23 (m, 2H), 7.20 – 7.15 (m, 1H), 7.13 – 7.08 (m, 2H), 3.86 – 3.66 (m, 2H), 2.83 (ddt, *J* = 9.4, 7.4, 5.5 Hz, 1H), 2.63 – 2.46 (m, 2H), 1.83 – 1.71 (m, 1H), 1.69 – 1.53 (m, 3H), 1.35 (t, *J* = 5.3 Hz, 1H), 1.30 – 1.14 (m, 2H) ppm;

^{13}C NMR (101 MHz, $CDCl_3$): δ_C 148.8, 142.4, 132.5, 129.0, 128.5, 128.4, 125.9, 119.0, 110.7, 67.1, 48.8, 35.8, 31.7, 31.4, 26.9 ppm.

IR (film): ν_{max} 3431, 2929, 2857, 2227, 1606, 1496, 1453, 1039, 836, 747, 699, 568 cm^{-1}.

HRMS (APCI$^+$): m/z calculated for $C_{19}H_{22}NO$ [M+H]$^+$, 280.1696; found, 280.1693.

4-[1-Phenyl-2-(4,4,5,5-tetramethyl-1,3,2-dioxaborolan-2-yl)ethyl]benzonitrile (3la)

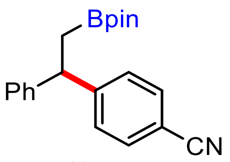

3la

Prepared following **General Procedure D**, using 2,2'-(1-phenylethane-1,2-diyl)bis(4,4,5,5-tetramethyl-1,3,2-dioxaborolane) (**1l**) (107 mg, 0.300 mmol, 1.50 equiv.), 4-bromo-*N,N*-dimethylaniline (64 mg, 0.32 mmol, 1.6 equiv.), *tert*-butyllithium (1.7 M in pentane, 376 µL, 0.640 mmol, 3.20 equiv.) and terephthalonitrile (**4a**) (25.6 mg, 0.200 mmol, 1.00 equiv.). Purification by flash column chromatography (90:10 pentane/diethyl ether) gave boronic ester **3la** (36.0 mg, 54%) as a colorless oil.

R*f* = 0.26 (91:9 *n*-hexane/EtOAc, CAM stain)

NMR Spectroscopy (*see spectra*):

> **^{1}H NMR** (400 MHz, $CDCl_3$): δ_H 7.54 (d, *J* = 8.4 Hz, 2H), 7.36 (d, *J* = 8.4 Hz, 2H), 7.31 – 7.13 (m, 5H), 4.32 (t, *J* = 8.4 Hz, 1H), 1.58 (dd, *J* = 8.4, 3.3 Hz, 2H), 1.06 (s, 12H) ppm;
>
> **^{13}C NMR** (101 MHz, $CDCl_3$): δ_C 152.4, 145.1, 132.3, 128.7, 128.7, 127.8, 126.7, 119.2, 109.9, 83.5, 46.8, 24.7, 24.7 ppm. The carbon attached to boron was not observed due to quadrupolar relaxation;
>
> **^{11}B NMR** (128 MHz, $CDCl_3$): δ_B 33.5 ppm.

IR (film): ν_{max} 2977, 2929, 2227, 1739, 1605, 1452, 1366, 1325, 1142, 997, 889, 846, 699, 675 cm^{-1}.

HRMS (EI$^+$): m/z calculated for $C_{20}H_{21}BNO_2$ [M-CH_3]$^+$, 318.1660; found, 318.1656.

All recorded spectroscopic data matched those previously reported in the literature.[30]

4-[1-(4-Methoxyphenyl)-2-(4,4,5,5-tetramethyl-1,3,2-dioxaborolan-2-yl)ethyl]benzonitrile (3ma)

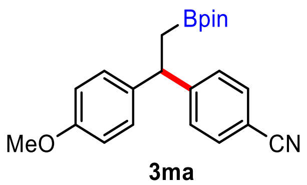

3ma

Prepared following **General Procedure D**, using 2,2'-[1-(*p*-tolyl)ethane-1,2-diyl]bis(4,4,5,5-tetramethyl-1,3,2-dioxaborolane) (**1m**) (117 mg, 0.300 mmol, 1.50 equiv.), 4-bromo-*N,N*-dimethylaniline (64 mg, 0.32 mmol, 1.6 equiv.), *tert*-butyllithium (1.7 M in pentane, 376 µL, 0.640 mmol, 3.20 equiv.) and terephthalonitrile (**4a**) (25.6 mg, 0.200 mmol, 1.00 equiv.). Purification by flash column chromatography (80:20 pentane/diethyl ether) gave boronic ester **3ma** (39.2 mg, 54%) as a colorless oil.

UOB Confidential & Sensitive

R$_f$ = 0.30 (80:20 *n*-hexane/EtOAc, CAM stain)

NMR Spectroscopy (*see spectra*):

^{1}H NMR (400 MHz, $CDCl_3$): δ_H 7.53 (d, *J* = 8.5 Hz, 2H), 7.34 (d, *J* = 8.2 Hz, 2H), 7.13 (d, *J* = 8.6 Hz, 2H), 6.81 (d, *J* = 8.7 Hz, 2H), 4.27 (t, *J* = 8.4 Hz, 1H), 3.76 (s, 3H), 1.55 (dd, *J* = 8.4, 2.9 Hz, 2H), 1.07 (s, 12H) ppm;

^{13}C NMR (101 MHz, $CDCl_3$): δ_C 158.3, 152.8, 137.3, 132.3, 128.7, 128.5, 119.3, 114.0, 109.8, 83.5, 55.4, 46.0, 24.7 ppm. The carbon attached to boron was not observed due to quadrupolar relaxation;

^{11}B NMR (128 MHz, $CDCl_3$): δ_B 33.4 ppm.

IR (film): ν_{max} 2971, 2939, 2227, 1740, 1609, 1511, 1368, 1229, 1217, 1144, 1037, 847, 576 cm^{-1}.

HRMS (EI$^+$): m/z calculated for $C_{22}H_{26}BNO_2$ [M]$^+$, 363.2000; found, 363.1998.

All recorded spectroscopic data matched those previously reported in the literature.[31]

4-{1-[(4,4,5,5-Tetramethyl-1,3,2-dioxaborolan-2-yl)methyl]cyclohexyl}benzonitrile (3na)

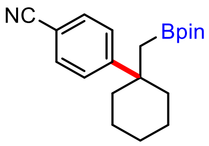

3na

Prepared following **General Procedure D**, using 4,4,5,5-tetramethyl-2-{[1-(4,4,5,5-tetramethyl-1,3,2-dioxaborolan-2-yl)cyclohexyl]methyl}-1,3,2-dioxaborolane (**1n**) (105 mg, 0.300 mmol, 1.50 equiv.), 4-bromo-*N,N*-dimethylaniline (64 mg, 0.32 mmol, 1.6 equiv.), *tert*-butyllithium (1.7 M in pentane, 376 µL, 0.640 mmol, 3.20 equiv.) and terephthalonitrile (**4a**) (25.6 mg, 0.200 mmol, 1.00 equiv.). Purification by flash column chromatography (91:9 pentane/diethyl ether) gave boronic ester **3na** (44.9 mg, 69%) as a colorless oil.

R$_f$ = 0.28 (91:9 *n*-hexane/EtOAc, CAM stain)

NMR Spectroscopy (*see spectra*):

^{1}H NMR (400 MHz, $CDCl_3$): δ_H 7.57 (d, *J* = 8.7 Hz, 2H), 7.48 (d, *J* = 8.7 Hz, 2H), 2.18 – 2.05 (m, 2H), 1.72 (ddd, *J* = 13.3, 9.5, 3.5 Hz, 2H), 1.63 – 1.52 (m, 2H), 1.47 –1.27 (m, 4H), 1.14 (s, 2H), 1.03 (s, 12H) ppm;

^{13}C NMR (101 MHz, $CDCl_3$): δ_C 155.1, 131.9, 127.7, 119.5, 109.0, 82.9, 40.4, 38.1, 26.3, 24.8, 22.7 ppm. The carbon attached to boron was not observed due to quadrupolar relaxation;

^{11}B NMR (128 MHz, $CDCl_3$): δ_B 33.3 ppm.

IR (film): ν_{max} 2977, 2928, 2858, 2226, 1740, 1606, 1505, 1454, 1362, 1321, 1230, 1215, 1142, 971, 883, 851, 834, 565 cm^{-1}.

HRMS (ESI$^+$): m/z calculated for $C_{20}H_{28}BNNaO_2$ [M+Na]$^+$, 348.2109; found, 348.2118.

UOB Confidential & Sensitive

4-[1-Methyl-2-(4,4,5,5-tetramethyl-1,3,2-dioxaborolan-2-yl)cyclohexyl]benzonitrile (3oa)

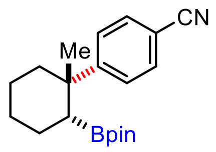

3oa

Prepared following **General Procedure D**, using 2,2'-(1-methylcyclohexane-1,2-diyl)bis(4,4,5,5-tetramethyl-1,3,2-dioxaborolane) (**1o**) (105 mg, 0.300 mmol, 1.50 equiv.), 4-bromo-*N,N*-dimethylaniline (64 mg, 0.32 mmol, 1.6 equiv.), *tert*-butyllithium (1.7 M in pentane, 376 µL, 0.640 mmol, 3.20 equiv.) and terephthalonitrile (**4a**) (25.6 mg, 0.200 mmol, 1.00 equiv.). Purification by flash column chromatography (91:9 pentane/diethyl ether) gave boronic ester **3oa** (52.1 mg, 80%) as a colorless oil.

R*f* = 0.26 (91:9 *n*-hexane/EtOAc, CAM stain)

NMR Spectroscopy (*see spectra*):

> ^{1}**H NMR** (400 MHz, $CDCl_3$): δ_H 7.57 (d, *J* = 8.6 Hz, 2H), 7.53 (d, *J* = 8.7 Hz, 2H), 1.78 – 1.50 (m, 8H), 1.39 (s, 3H), 1.35 – 1.24 (m, 1H), 1.06 (s, 12H) ppm;
>
> ^{13}**C NMR** (101 MHz, $CDCl_3$): δ_C 157.4, 131.9, 127.0, 119.5, 109.1, 82.9, 40.2, 39.9, 26.2, 24.7, 24.6, 24.2, 22.5, 22.4 ppm. The carbon attached to boron was not observed due to quadrupolar relaxation;
>
> ^{11}**B NMR** (128 MHz, $CDCl_3$): δ_B 31.7 ppm.

IR (film): ν_{max} 2977, 2926, 2861, 2227, 1606, 1368, 1320, 1143, 991, 848, 566 cm^{-1}.

HRMS (ESI$^+$): m/z calculated for $C_{20}H_{29}BNO_2$ [M+H]$^+$, 326.2286; found, 326.2284.

4-[2,3-Dimethyl-1-(4,4,5,5-tetramethyl-1,3,2-dioxaborolan-2-yl)butan-2-yl]benzonitrile (3pa)

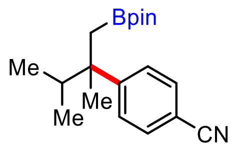

3pa

Prepared following **General Procedure D**, using 2,2'-(2,3-dimethylbutane-1,2-diyl)bis(4,4,5,5-tetramethyl-1,3,2-dioxaborolane) (**1p**) (101 mg, 0.300 mmol, 1.50 equiv.), 4-bromo-*N,N*-dimethylaniline (64 mg, 0.32 mmol, 1.6 equiv.), *tert*-butyllithium (1.7 M in pentane, 376 µL, 0.640 mmol, 3.20 equiv.) and terephthalonitrile (**4a**) (25.6 mg, 0.200 mmol, 1.00 equiv.). Purification by flash column chromatography (91:9 pentane/diethyl ether) gave boronic ester **3pa** (56.4 mg, 90%) as a colorless oil.

R*f* = 0.25 (91:9 *n*-hexane/EtOAc, CAM stain)

NMR Spectroscopy (*see spectra*):

^{1}H NMR (400 MHz, $CDCl_3$): δ_H 7.54 (d, *J* = 8.6 Hz, 2H), 7.42 (d, *J* = 8.6 Hz, 2H), 1.84 (p, *J* = 6.8 Hz, 1H), 1.35 (s, 3H), 1.33 (d, *J* = 14.7 Hz, 1H), 1.14 (d, *J* = 14.7 Hz, 1H), 0.98 (s, 6H), 0.92 (s, 6H), 0.86 (d, *J* = 6.8 Hz, 3H), 0.58 (d, *J* = 6.8 Hz, 3H) ppm;

^{13}C NMR (101 MHz, $CDCl_3$): δ_C 155.9, 131.5, 127.7, 119.5, 109.0, 82.9, 43.0, 40.5, 24.8, 24.5, 20.2, 17.9, 17.6 ppm. The carbon attached to boron was not observed due to quadrupolar relaxation;

^{11}B NMR (128 MHz, $CDCl_3$): δ_B 32.1 ppm.

IR (film): v_{max} 2975, 2879, 2227, 1606, 1468, 1370, 1354, 1324, 1219, 1145, 969, 846, 569 cm^{-1}.

HRMS (ESI$^+$): m/z calculated for $C_{19}H_{29}BNO_2$ [M+H]$^+$, 314.2286; found, 314.2284.

4-[(*trans*)-2-(4,4,5,5-Tetramethyl-1,3,2-dioxaborolan-2-yl)cyclopentyl]benzonitrile ((*trans*)-3qa)

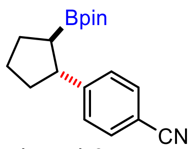

(*trans*)-3qa

Prepared following **General Procedure D**, using (*cis*)-1,2-bis(4,4,5,5-tetramethyl-1,3,2-dioxaborolan-2-yl)cyclopentane (**1q**) (97 mg, 0.30 mmol, 1.5 equiv.), 4-bromo-*N,N*-dimethylaniline (64 mg, 0.32 mmol, 1.6 equiv.), *tert*-butyllithium (1.7 M in pentane, 376 µL, 0.640 mmol, 3.20 equiv.) and terephthalonitrile (**4a**) (25.6 mg, 0.200 mmol, 1.00 equiv.). Purification by flash column chromatography (91:9 pentane/diethyl ether) gave boronic ester **(*trans*)-3qa** (38.0 mg, 64%) as a colorless oil.

R_f = 0.26 (91:9 *n*-hexane/EtOAc, CAM stain)

NMR Spectroscopy (*see spectra*):

^{1}H NMR (400 MHz, $CDCl_3$): δ_H 7.54 (d, *J* = 8.4 Hz, 2H), 7.34 (d, *J* = 8.4 Hz, 2H), 3.14 – 3.04 (m, 1H), 2.14 -2.05 (m, 1H), 1.99 (dddd, *J* = 12.3, 8.9, 7.4, 4.8 Hz, 1H), 1.90 – 1.56 (m, 4H), 1.32 (dd, *J* = 19.4, 10.2 Hz, 1H), 1.16 (s, 6H), 1.15 (s, 6H) ppm;

^{13}C NMR (101 MHz, $CDCl_3$): δ_C 152.2, 132.1, 128.2, 119.4, 109.6, 83.3, 49.4, 35.9, 29.2, 26.6, 24.8, 24.7 ppm. The carbon attached to boron was not observed due to quadrupolar relaxation;

^{11}B NMR (128 MHz, $CDCl_3$): δ_B 34.1 ppm.

IR (film): v_{max} 2974, 2870, 2227, 1740, 1607, 1415, 1370, 1319, 1216, 1143, 851, 830, 561 cm^{-1}.

HRMS (ESI$^+$): m/z calculated for $C_{18}H_{25}BNO_2$ [M+H]$^+$, 298.1973; found, 298.1974.

UOB Confidential & Sensitive

4-[(*cis*)-2-(4,4,5,5-Tetramethyl-1,3,2-dioxaborolan-2-yl)cyclopentyl]benzonitrile ((*cis*)-3qa)

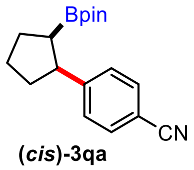

(*cis*)-3qa

According to a modified literature procedure:[32] To a Schlenk flask were added 1,2-bis(4,4,5,5-tetramethyl-1,3,2-dioxaborolan-2-yl)cyclopentane (**1p**) (64 mg, 0.20 mmol, 1.0 equiv.), 4-bromobenzonitrile (55 mg, 0.30 mmol, 1.5 equiv.), Pd(dppf)Cl_2 (7.4 mg, 0.010 mmol, 5.0 mol%), and K_2CO_3 (41 mg, 0.30 mmol, 1.5 equiv.), after which the flask was evacuated and back-filled with nitrogen three times. Subsequently, 1,4-dioxane (1.0 mL) and H_2O (0.1 mL) were added via syringe and the resulting suspension was heated to 100 °C for 24 h, after which the reaction mixture was filtered over Celite, eluting with diethyl ether. The filtrate was concentrated under reduced pressure and the resulting crude material was purified by flash column chromatography (91:9 pentane/diethyl ether) to afford **(*cis*)-3pa** (25.6 mg, 43%) as a colorless oil.

R_f = 0.26 (91:9 *n*-hexane/EtOAc, CAM stain)

NMR Spectroscopy (*see spectra*):

^{1}H NMR (400 MHz, $CDCl_3$): δ_H 7.54 (d, *J* = 8.4 Hz, 2H), 7.34 (d, *J* = 8.3 Hz, 2H), 3.11 – 2.99 (m, 1H), 2.20 (dt, *J* = 12.8, 6.9 Hz, 1H), 2.11 (dtdd, *J* = 12.7, 7.9, 4.8, 1.1 Hz, 1H), 1.92 (ddt, *J* = 12.7, 9.3, 7.6 Hz, 1H), 1.86 – 1.75 (m, 1H), 1.69 – 1.38 (m, 3H), 1.25 (s, 12H) ppm;

^{13}C NMR (101 MHz, $CDCl_3$): δ_C 152.0, 132.2, 128.2, 119.4, 109.5, 83.2, 47.7, 37.5, 34.7, 27.7, 24.9 ppm. The carbon attached to boron was not observed due to quadrupolar relaxation;

^{11}B NMR (128 MHz, $CDCl_3$): δ_B 34.5 ppm.

IR (film): ν_{max} 2974, 2870, 2227, 1740, 1607, 1415, 1370, 1319, 1216, 1143, 851, 830 cm^{-1}.

HRMS (ESI$^+$): m/z calculated for $C_{18}H_{25}BNO_2$ $[M+H]^+$, 298.1973; found, 298.1974.

4-[(*trans*)-2-(4,4,5,5-Tetramethyl-1,3,2-dioxaborolan-2-yl)cyclohexyl]benzonitrile ((*trans*)-3ra)

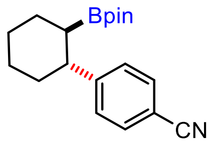

(*trans*)-3ra

Prepared following **General Procedure D**, using (*cis*)-1,2-bis(4,4,5,5-tetramethyl-1,3,2-dioxaborolan-2-yl)cyclohexane (**1r**) (101 mg, 0.300 mmol, 1.50 equiv.), 4-bromo-*N,N*-dimethylaniline (64 mg, 0.32 mmol, 1.6 equiv.), *tert*-butyllithium (1.7 M in pentane, 376 µL, 0.640 mmol, 3.20 equiv.) and terephthalonitrile (**4a**) (25.6 mg, 0.200 mmol, 1.00 equiv.). Purification by flash column chromatography (91:9 pentane/diethyl ether) gave

UOB Confidential & Sensitive

boronic ester **(*trans*)-3ra** (28.0 mg, 45%) as a colorless oil.

R*f* = 0.27 (91:9 *n*-hexane/EtOAc, CAM stain)

NMR Spectroscopy (*see spectra*):

^{1}H NMR (400 MHz, $CDCl_3$): δ_H 7.53 (d, *J* = 8.3 Hz, 2H), 7.31 (d, *J* = 8.3 Hz, 2H), 2.63 (td, *J* = 11.7, 3.2 Hz, 1H), 1.89 – 1.75 (m, 4H), 1.51 – 1.18 (m, 5H), 0.96 (d, *J* = 3.6 Hz, 12H) ppm;

^{13}C NMR (101 MHz, $CDCl_3$): δ_C 153.5, 132.1, 128.5, 119.4, 109.6, 83.0, 46.4, 35.8, 28.1, 26.8, 26.7, 24.6, 24.4 ppm. The carbon attached to boron was not observed due to quadrupolar relaxation;

^{11}B NMR (128 MHz, $CDCl_3$): δ_B 33.3 ppm.

IR (film): ν_{max} 2977, 2923, 2851, 2226, 1607, 1446, 1377, 1323, 1260, 1143, 848, 832 cm^{-1}.

HRMS (ESI$^+$): m/z calculated for $C_{19}H_{27}BNO_2$ [M+H]$^+$, 312.2129; found, 312.2131.

4-[(*cis*)-2-(4,4,5,5-Tetramethyl-1,3,2-dioxaborolan-2-yl)cyclohexyl]benzonitrile ((*cis*)-3ra)

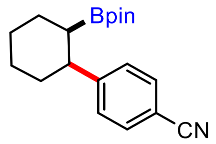

(*cis*)-3ra

According to a modified literature procedure:[32] To a Schlenk flask was added (*cis*)-1,2-bis(4,4,5,5-tetramethyl-1,3,2-dioxaborolan-2-yl)cyclohexane (**1r**) (67 mg, 0.20 mmol, 1.0 equiv.), 4-bromobenzonitrile (55 mg, 0.30 mmol, 1.5 equiv.), Pd(dppf)Cl_2 (7.4 mg, 0.010 mmol, 5.0 mol%), and K_2CO_3 (41 mg, 0.30 mmol, 1.5 equiv.), after which the flask was evacuated and back-filled with nitrogen three times. Subsequently, 1,4-dioxane (1.0 mL) and H_2O (0.10 mL) were added via syringe and the resulting suspension was heated to 100 °C for 24 h, after which the reaction mixture was filtered over Celite, eluting with diethyl ether. The filtrate was concentrated under reduced pressure and the resulting crude material was purified by flash column chromatography (91:9 pentane/diethyl ether) to afford **(*cis*)-3ra** (18.7 mg, 30%) as a colorless oil.

R*f* = 0.27 (91:9 *n*-hexane/EtOAc, CAM stain)

NMR Spectroscopy (*see spectra*):

^{1}H NMR (400 MHz, $CDCl_3$): δ_H 7.55 (d, *J* = 8.3 Hz, 2H), 7.29 (d, *J* = 8.1 Hz, 2H), 2.55 (ddd, *J* = 11.8, 8.2, 3.4 Hz, 1H), 1.96 – 1.79 (m, 4H), 1.50 – 1.33 (m, 3H), 1.32 – 1.24 (m, 1H), 1.24 (s, 12H), 1.10 (tt, *J* = 12.1, 2.8 Hz, 1H) ppm;

^{13}C NMR (101 MHz, $CDCl_3$): δ_C 153.7, 132.3, 127.9, 119.4, 109.7, 83.2, 45.7, 35.5, 33.6, 30.5, 27.5, 27.3, 24.9, 24.9 ppm. The carbon attached to boron was not observed due to quadrupolar relaxation;

^{11}B NMR (128 MHz, $CDCl_3$): δ_B 34.8 ppm.

IR (film): ν_{max} 2977, 2923, 2851, 2226, 1607, 1446, 1377, 1323, 1260, 1143, 848, 832 cm^{-1}.

UOB Confidential & Sensitive

HRMS (ESI$^+$): m/z calculated for $C_{19}H_{27}BNO_2$ [M+H]$^+$, 312.2129; found, 312.2131.

4-{1-[1,2-Bis(4,4,5,5-tetramethyl-1,3,2-dioxaborolan-2-yl)ethyl]cyclopentyl}benzonitrile (3sa)

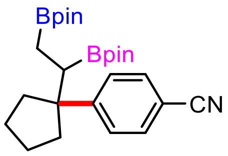

3sa

Prepared following **General Procedure D**, using 2,2'-{1-[1-(4,4,5,5-tetramethyl-1,3,2-dioxaborolan-2-yl)cyclopentyl]ethane-1,2-diyl}bis(4,4,5,5-tetramethyl-1,3,2-dioxaborolane) (**1s**) (143 mg, 0.300 mmol, 1.50 equiv.), 4-bromo-*N,N*-dimethylaniline (64 mg, 0.32 mmol, 1.6 equiv.), *tert*-butyllithium (1.7 M in pentane, 376 µL, 0.640 mmol, 3.20 equiv.) and terephthalonitrile (**4a**) (25.6 mg, 0.200 mmol, 1.00 equiv.). Purification by flash column chromatography (91:9 pentane/diethyl ether) gave boronic ester **3sa** (66.8 mg, 74%) as a colorless oil.

R*f* = 0.21 (91:9 *n*-hexane/EtOAc, CAM stain)

NMR Spectroscopy (*see spectra*):

^{1}**H NMR** (400 MHz, $CDCl_3$): δ_H 7.51 (d, J = 8.6 Hz, 2H), 7.43 (d, J = 8.6 Hz, 2H), 2.14 – 2.04 (m, 1H), 2.02 – 1.86 (m, 3H), 1.77 – 1.57 (m, 2H), 1.53 (dd, J = 11.9, 4.6 Hz, 1H), 1.50 – 1.30 (m, 2H), 1.18 (s, 12H), 1.17 (s, 6H), 1.16 (s, 6H), 0.71 – 0.56 (m, 2H) ppm;

^{13}**C NMR** (101 MHz, $CDCl_3$): δ_C 154.2, 131.5, 128.5, 119.5, 109.1, 83.2, 83.1, 54.3, 37.4, 35.9, 25.4, 25.2, 24.9, 24.7, 23.2, 23.1 ppm. The carbon attached to boron was not observed due to quadrupolar relaxation;

^{11}**B NMR** (128 MHz, $CDCl_3$): δ_B 33.7 ppm.

IR (film): ν_{max} 2979, 2874, 2225, 1740, 1606, 1458, 1361, 1313, 1212, 1143, 967, 908, 849, 835, 734 cm^{-1}.

HRMS (ESI$^+$): m/z calculated for $C_{26}H_{40}B_2NO_4$ [M+H]$^+$, 452.3147; found, 452.3136.

4-{2-[(1*S*,5*S*)-4-Methyl-5-(4,4,5,5-tetramethyl-1,3,2-dioxaborolan-2-yl)cyclohex-3-en-1-yl]propan-2-yl}benzonitrile (3ta)

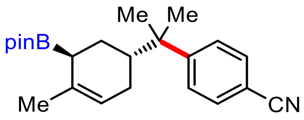

3ta

Prepared following **General Procedure D**, using 2,2'-[(1*S*,2*R*,3*S*,5*S*)-2,6,6-Trimethylbicyclo[3.1.1]heptane-2,3-diyl]bis(4,4,5,5-tetramethyl-1,3,2-dioxaborolane) (**1t**) (117 mg, 0.300 mmol, 1.50 equiv.), 4-bromo-*N,N*-dimethylaniline (64 mg, 0.32 mmol, 1.6 equiv.), *tert*-butyllithium (1.7 M in pentane, 376 µL, 0.640 mmol, 3.20 equiv.) and terephthalonitrile (**4a**) (25.6 mg, 0.200 mmol, 1.00 equiv.). Purification by flash column

chromatography (91:9 pentane/diethyl ether) gave boronic ester **3ta** (54.8 mg, 75%) as a colorless oil.

R$_f$ = 0.29 (91:9 *n*-hexane/EtOAc, CAM stain)

NMR Spectroscopy (*see spectra*):

^{1}**H NMR** (400 MHz, $CDCl_3$): δ_H 7.56 (d, J = 8.5 Hz, 2H), 7.43 (d, J = 8.5 Hz, 2H), 5.31 (td, J = 2.6, 1.1 Hz, 1H), 1.89 – 1.59 (m, 8H), 1.35 (dd, J = 12.2, 6.2 Hz, 1H), 1.28 (s, 3H), 1.25 (s, 3H), 1.21 (s, 6H), 1.17 (s, 6H) ppm;

^{13}**C NMR** (101 MHz, $CDCl_3$): δ_C 156.0, 135.0, 131.7, 127.3, 119.8, 119.3, 109.2, 83.3, 43.1, 40.8, 27.2, 26.4, 25.2, 25.0, 24.6, 24.3, 23.8 ppm. The carbon attached to boron was not observed due to quadrupolar relaxation;

^{11}**B NMR** (128 MHz, $CDCl_3$): δ_B 33.7 ppm.

IR (film): ν_{max} 2973, 2926, 2227, 1740, 1607, 1471, 1450, 1370, 1357, 1318, 1259, 1216, 1141, 968, 839 cm^{-1}.

HRMS (ESI$^+$): m/z calculated for $C_{23}H_{32}BNNaO_2$ [M+Na]$^+$, 388.2422; found, 388.2435.

(*R*)-4-{2-[4-(Hydroxymethyl)cyclohex-3-en-1-yl]propan-2-yl}benzonitrile (3ua)

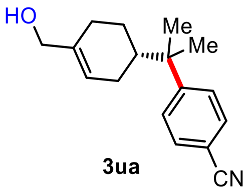

3ua

Prepared following **General Procedure D**, using 2-{(*1S,2R,5S*)-6,6-Dimethyl-2-[(4,4,5,5-tetramethyl-1,3,2-dioxaborolan-2-yl)methyl]bicyclo[3.1.1]heptan-2-yl}-4,4,5,5-tetramethyl-1,3,2-dioxaborolane (**1u**) (117 mg, 0.300 mmol, 1.50 equiv.), 4-bromo-*N,N*-dimethylaniline (64 mg, 0.32 mmol, 1.6 equiv.), *tert*-butyllithium (1.7 M in pentane, 376 µL, 0.640 mmol, 3.20 equiv.) and terephthalonitrile (**4a**) (25.6 mg, 0.200 mmol, 1.00 equiv.). The crude material was oxidized following the procedure mentioned above. Purification by flash column chromatography (67:33 *n*-hexane/EtOAc) gave the title compound **3ua** (32.2 mg, 63%) as a colorless oil.

R$_f$ = 0.27 (67:33 *n*-hexane/EtOAc, $KMnO_4$ stain)

NMR Spectroscopy (*see spectra*):

^{1}**H NMR** (400 MHz, $CDCl_3$): δ_H 7.59 (d, J = 8.6 Hz, 2H), 7.43 (d, J = 8.7 Hz, 2H), 5.59 (s, 1H), 3.96 (d, J = 4.7 Hz, 2H), 2.08 (dd, J = 17.2, 5.4 Hz, 1H), 2.03 – 1.89 (m, 1H), 1.83 – 1.71 (m, 3H), 1.70 – 1.61 (m, 1H), 1.41 (brs, 1H), 1.29 (d, J = 15.8 Hz, 6H), 1.17 (tdd, J = 15.8, 12.7, 6.7 Hz, 1H) ppm;

^{13}**C NMR** (101 MHz, $CDCl_3$): δ_C 155.6, 137.5, 131.9, 127.1, 122.7, 119.2, 109.5, 67.1, 45.0, 40.8, 27.0, 26.9, 25.0, 24.7, 24.2 ppm.

IR (film): ν_{max} 3409, 2968, 2922, 2227, 1606, 1505, 1403, 1367, 1053, 1005, 840, 577 cm^{-1}.

HRMS (ESI$^+$): m/z calculated for $C_{17}H_{21}NNaO$ [M+Na]$^+$, 278.1515; found, 278.1523.

UOB Confidential & Sensitive

4-[(1*R,3a*R**,4*R**,6a*S**)-4-Hydroxyoctahydropentalen-1-yl]benzonitrile (3va)**

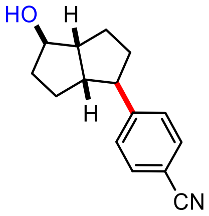

3va

Prepared following **General Procedure D**, using (*5R*,6S*,Z*)-5,6-bis(4,4,5,5-tetramethyl-1,3,2-dioxaborolan-2-yl)cyclooct-1-ene (**1v**) (109 mg, 0.300 mmol, 1.50 equiv.), 4-bromo-*N,N*-dimethylaniline (64 mg, 0.32 mmol, 1.6 equiv.), *tert*-butyllithium (1.7 M in pentane, 376 µL, 0.640 mmol, 3.20 equiv.) and terephthalonitrile (**4a**) (25.6 mg, 0.200 mmol, 1.00 equiv.). The crude material was oxidized following the procedure mentioned above. Purification by flash column chromatography (67:33 *n*-hexane/EtOAc) gave the title compound **3va** (30.5 mg, 67%, 6.6:1 d.r.) as a colorless oil.

R_f = 0.24 (67:33 *n*-hexane/EtOAc, $KMnO_4$ stain)

NMR Spectroscopy (*see spectra*):

^{1}H NMR (400 MHz, $CDCl_3$): δ_H 7.62 – 7.55 (m, 2H), 7.36 – 7.28 (m, 2H), 4.06 (dt, *J* = 3.9, 1.9 Hz, 0.87H), 3.88 (dt, *J* = 6.4, 4.7 Hz, 0.13H), 3.21 (dt, *J* = 12.5, 6.7 Hz, 0.13H), 3.01 (p, *J* = 8.0 Hz, 0.13H), 2.68 – 2.41 (m, 2.74H), 2.19 – 2.09 (m, 1H), 2.06 – 1.79 (m, 3H), 1.75 – 1.58 (m, 2H), 1.52 – 1.41 (m, 2H), 1.27 – 1.15 (m, 0.87H), 0.88 – 0.77 (m, 0.13H) ppm;

^{13}C NMR (101 MHz, $CDCl_3$): δ_C (*Major isomer*) 151.1, 132.3, 128.2, 119.2, 109.9, 79.3, 53.6, 53.5, 50.7, 36.1, 33.4, 31.2, 28.8; δ_C (*Minor isomer*) 151.1, 131.9, 128.8, 119.2, 109.9, 81.3, 51.7, 48.9, 46.2, 35.4, 29.9, 27.5, 25.4 ppm.

IR (film): ν_{max} 3381, 2942, 2864, 2226, 1606, 1504, 1448, 1343, 1177, 1066, 1032, 992, 973, 827, 731 cm^{-1}.

HRMS (ESI$^+$): m/z calculated for $C_{15}H_{17}NNaO$ [M+Na]$^+$, 250.1202; found, 250.1198.

4-{2-{2-[(4,4,5,5-Tetramethyl-1,3,2-dioxaborolan-2-yl)methyl]cyclopentyl}propan-2-yl}benzonitrile (3wa)

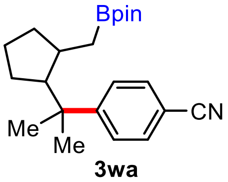

3wa

Prepared following **General Procedure D**, using 2,2'-(6-methylhept-5-ene-1,2-diyl)bis(4,4,5,5-tetramethyl-1,3,2-dioxaborolane) (**1w**) (109 mg, 0.300 mmol, 1.50 equiv.), 4-bromo-*N,N*-dimethylaniline (64 mg, 0.32 mmol, 1.6 equiv.), *tert*-butyllithium (1.7 M in pentane, 376 µL, 0.640 mmol, 3.20 equiv.) and terephthalonitrile (**4a**) (25.6

mg, 0.200 mmol, 1.00 equiv.). Purification by flash column chromatography (91:9 pentane/diethyl ether) gave boronic ester **3wa** (57.3 mg, 81%, 2.2:1 d.r.) as a colorless oil.

R_f = 0.28 (91:9 *n*-hexane/EtOAc, CAM stain)

NMR Spectroscopy (*see spectra*):

^{1}**H NMR** (400 MHz, $CDCl_3$): δ_H 7.58 – 7.53 (m, 2H), 7.48 – 7.42 (m, 2H), 2.09 – 1.97 (m, 1.4H), 1.93 – 1.82 (m, 0.6H), 1.76 – 1.59 (m, 2H), 1.58 – 1.06 (m, 22H), 0.64 – 0.46 (m, 2H) ppm;

^{13}**C NMR** (101 MHz, $CDCl_3$): δ_C (*Major isomer*)156.6, 131.8, 127.1, 119.4, 109.1, 83.0, 55.3, 39.9, 36.7, 33.6, 29.5, 25.7, 25.0, 24.7, 24.1, 21.7 ppm; δ_C (*Minor isomer*) 156.4, 131.8, 127.2, 119.4, 109.2, 82.9, 58.5, 41.1, 36.3, 35.8, 29.4, 25.7, 25.4, 25.1, 25.0, 24.8 ppm. The carbon attached to boron was not observed due to quadrupolar relaxation;

^{11}**B NMR** (128 MHz, $CDCl_3$): δ_B 33.7 ppm.

IR (film): ν_{max} 2971, 2948, 2227, 1740, 1606, 1367, 1320, 1229, 1217, 1144, 968, 841, 579 cm^{-1}.

HRMS (ESI$^+$): m/z calculated for $C_{22}H_{32}BNNaO_2$ [M+Na]$^+$, 376.2422; found, 376.2430.

2-[4,4-Dimethyl-1-(4,4,5,5-tetramethyl-1,3,2-dioxaborolan-2-yl)pentan-2-yl]benzonitrile (3ab)

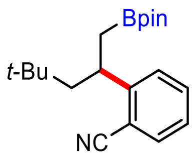

3ab

Prepared following **General Procedure D**, using 2,2'-(4,4-dimethylpentane-1,2-diyl)bis(4,4,5,5-tetramethyl-1,3,2-dioxaborolane) (**1a**) (106 mg, 0.300 mmol, 1.50 equiv.), 4-bromo-*N,N*-dimethylaniline (64 mg, 0.32 mmol, 1.6 equiv.), *tert*-butyllithium (1.7 M in pentane, 376 µL, 0.640 mmol, 3.20 equiv.) and phthalonitrile (**2b**) (25.6 mg, 0.200 mmol, 1.00 equiv.). Purification by flash column chromatography (91:9 pentane/diethyl ether) gave boronic ester **3ab** (21.0 mg, 32%) as a colorless oil.

R_f = 0.30 (91:9 *n*-hexane/EtOAc, CAM stain)

NMR Spectroscopy (*see spectra*):

^{1}**H NMR** (400 MHz, $CDCl_3$): δ_H 7.54 (ddd, *J* = 7.7, 1.5, 0.6 Hz, 1H), 7.52 – 7.44 (m, 1H), 7.40 (dd, *J* = 8.1, 1.2 Hz, 1H), 7.21 (td, *J* = 7.5, 1.2 Hz, 1H), 3.47 (tdd, *J* = 9.4, 6.7, 3.5 Hz, 1H), 1.81 (dd, *J* = 14.2, 9.3 Hz, 1H), 1.63 (dd, *J* = 14.2, 3.5 Hz, 1H), 1.23 – 1.20 (m, 1H), 1.18 – 1.13 (m, 1H), 1.09 (s, 6H), 1.08 (s, 6H), 0.79 (s, 9H) ppm.

^{13}**C NMR** (101 MHz, $CDCl_3$): δ_C 153.3, 132.8, 132.7, 127.9, 126.2, 118.8, 112.8, 83.2, 53.2, 36.4, 31.5, 30.3, 24.8, 24.8 ppm. The carbon attached to boron was not observed due to quadrupolar relaxation;

^{11}**B NMR** (128 MHz, $CDCl_3$): δ_B 33.3 ppm.

IR (film): v_{max} 2952, 2224, 1740, 1468, 1366, 1325, 1216, 1143, 967, 847, 762, 733, 550 cm^{-1}.

HRMS (ESI$^+$): m/z calculated for $C_{20}H_{30}BNNaO_2$ [M+Na]$^+$, 350.2265; found, 350.2282.

4-[4,4-Dimethyl-1-(4,4,5,5-tetramethyl-1,3,2-dioxaborolan-2-yl)pentan-2-yl]-2,5-dimethylbenzonitrile (3ac)

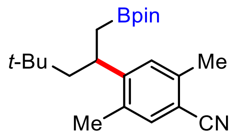

3ac

Prepared following **General Procedure D**, using 2,2'-(4,4-dimethylpentane-1,2-diyl)bis(4,4,5,5-tetramethyl-1,3,2-dioxaborolane) (**1a**) (106 mg, 0.300 mmol, 1.50 equiv.), 4-bromo-*N,N*-dimethylaniline (64 mg, 0.32 mmol, 1.6 equiv.), *tert*-butyllithium (1.7 M in pentane, 376 µL, 0.640 mmol, 3.20 equiv.) and 2,5-dimethylterephthalonitrile (**2c**) (31.3 mg, 0.200 mmol, 1.00 equiv.). Purification by flash column chromatography (91:9 pentane/diethyl ether) gave boronic ester **3ac** (59.3 mg, 81%) as a white solid.

R$_f$ = 0.32 (91:9 *n*-hexane/EtOAc, CAM stain)

NMR Spectroscopy (*see spectra*):

> **^{1}H NMR** (400 MHz, CDCl$_3$): δ_H 7.29 (s, 1H), 7.14 (s, 1H), 3.22 (tdd, J = 9.1, 6.6, 4.3 Hz, 1H), 2.46 (s, 3H), 2.39 (s, 3H), 1.71 (ddd, J = 14.2, 8.4, 1.4 Hz, 1H), 1.53 (ddd, J = 14.2, 4.3, 1.4 Hz, 1H), 1.26 – 0.97 (m, 14H), 0.77 (s, 9H) ppm;
>
> **^{13}C NMR** (101 MHz, CDCl$_3$): δ_C 153.6, 139.2, 133.9, 133.6, 128.4, 118.9, 109.3, 83.2, 53.1, 31.6, 30.4, 24.9, 24.7, 20.3, 19.6 ppm. The carbon attached to boron was not observed due to quadrupolar relaxation;
>
> **^{11}B NMR** (128 MHz, CDCl$_3$): δ_B 32.8 ppm.

IR (film): v_{max} 2955, 2221, 1740, 1466, 1366, 1321, 1215, 1143, 967, 879, 847 cm^{-1}.

HRMS (ESI$^+$): m/z calculated for $C_{22}H_{34}BNNaO_2$ [M+Na]$^+$, 378.2579; found, 378.2592.

Ethyl 4-[4,4-dimethyl-1-(4,4,5,5-tetramethyl-1,3,2-dioxaborolan-2-yl)pentan-2-yl]benzoate (3ad)

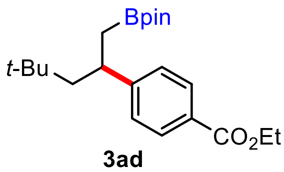

3ad

Prepared following **General Procedure E**, using 2,2'-(4,4-dimethylpentane-1,2-diyl)bis(4,4,5,5-tetramethyl-1,3,2-dioxaborolane) (**1a**) (106 mg, 0.300 mmol, 1.50 equiv.), 4CzIPN (7.9 mg, 5.0 mol%), phenyllithium (1.9 M

in dibutyl ether, 168 µL, 0.320 mmol, 1.60 equiv.) and ethyl 4-cyanobenzoate (**2d**) (35.0 mg, 0.200 mmol, 1.00 equiv.). Purification by flash column chromatography (93:7 pentane/diethyl ether) gave boronic ester **3ad** (49.4 mg, 66%) as a colorless oil.

R_f = 0.39 (91:9 *n*-hexane/EtOAc, CAM stain)

NMR Spectroscopy (*see spectra*):

^{1}H NMR (400 MHz, CDCl$_3$): δ_H 7.91 (d, *J* = 8.3 Hz, 2H), 7.30 (d, *J* = 8.3 Hz, 2H), 4.34 (td, *J* = 7.2, 6.6 Hz, 2H), 3.04 (ddt, *J* = 12.6, 7.4, 3.3 Hz, 1H), 1.76 (dd, *J* = 14.1, 9.7 Hz, 1H), 1.54 (dd, *J* = 14.1, 3.3 Hz, 1H), 1.37 (t, *J* = 7.1 Hz, 3H), 1.15 (dd, *J* = 15.3, 7.3 Hz, 1H), 1.11 – 1.06 (m, 1H), 1.10 (s, 6H), 1.08 (s, 6H), 0.73 (s, 9H) ppm;

^{13}C NMR (101 MHz, CDCl$_3$): δ_C 167.0, 154.9, 129.6, 128.0, 127.8, 83.2, 60.8, 53.0, 38.7, 31.6, 30.4, 24.9, 24.8, 14.5 ppm. The carbon attached to boron was not observed due to quadrupolar relaxation;

^{11}B NMR (128 MHz, CDCl$_3$): δ_B 33.6 ppm.

IR (film): ν_{max} 2971, 2951, 1738, 1719, 1609, 1366, 1323, 1274, 1217, 1144, 1101, 1021, 968, 846, 773, 710, 528 cm^{-1}.

HRMS (ESI$^+$): m/z calculated for $C_{22}H_{36}BO_4$ [M+H]$^+$, 375.2710; found, 375.2695.

4-[4,4-Dimethyl-1-(4,4,5,5-tetramethyl-1,3,2-dioxaborolan-2-yl)pentan-2-yl]pyridine (3ae)

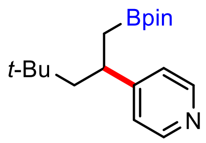

3ae

Prepared followinga modified **General Procedure D**, using 2,2'-(4,4-dimethylpentane-1,2-diyl)bis(4,4,5,5-tetramethyl-1,3,2-dioxaborolane) (**1a**) (106 mg, 0.300 mmol, 1.50 equiv.), 4-bromo-*N,N*-dimethylaniline (64 mg, 0.32 mmol, 1.6 equiv.), *tert*-butyllithium (1.7 M in pentane, 376 µL, 0.640 mmol, 3.20 equiv.), and isonicotinonitrile (**2e**) (20.8 mg, 0.200 mmol, 1.00 equiv.) in DMF (1.0 mL). The reaction mixture was not washed with 2 M HCl solution but transferred to a 50 mL round flask with Et_2O (20 mL) and concentrated under reduced pressure. Purification by flash column chromatography (86:14 *n*-hexane/EtOAc) gave boronic ester **3ae** (24.9, 41%) as a colorless oil.

Prepared following **General Procedure E**, using 2,2'-(4,4-dimethylpentane-1,2-diyl)bis(4,4,5,5-tetramethyl-1,3,2-dioxaborolane) (**1a**) (106 mg, 0.300 mmol, 1.50 equiv.), 4CzIPN (7.9 mg, 5.0 mol%), phenyllithium (1.9 M in dibutyl ether, 168 µL, 0.320 mmol, 1.60 equiv.) and isonicotinonitrile (**2e**) (20.8 mg, 0.200 mmol, 1.00 equiv.). Purification by flash column chromatography (75:25 pentane/EtOAc) gave boronic ester **3ae** (30.9 mg, 51%) as a colorless oil.

R_f = 0.38 (80:20 *n*-hexane/EtOAc, CAM stain)

UOB Confidential & Sensitive

NMR Spectroscopy (<u>*see spectra*</u>):

[1]**H NMR** (400 MHz, $CDCl_3$): δ_H 8.44 (dt, J = 4.6, 1.5 Hz, 2H), 7.18 (dt, J = 4.6, 2.5 Hz, 2H), 3.06 – 2.87 (m, 1H), 1.84 – 1.69 (m, 1H), 1.59 – 1.48 (m, 1H), 1.19 – 0.99 (m, 14H), 0.76 (s, 9H) ppm;

[13]**C NMR** (101 MHz, $CDCl_3$): δ_C 158.9, 149.3, 123.4, 83.3, 52.4, 38.1, 31.6, 30.3, 24.9, 24.8 ppm. The carbon attached to boron was not observed due to quadrupolar relaxation;

[11]**B NMR** (128 MHz, $CDCl_3$): δ_B 33.3 ppm.

IR (film): ν_{max} 2971, 2952, 1740, 1598, 1468, 1415, 1366, 1320, 1217, 1143, 967, 848, 821, 537 cm^{-1}.

HRMS (EI^+): m/z calculated for $C_{17}H_{27}BNO_2$ $[M-CH_3]^+$, 288.2129; found, 288.2126.

4-[4,4-Dimethyl-1-(4,4,5,5-tetramethyl-1,3,2-dioxaborolan-2-yl)pentan-2-yl]-3-methylpyridine (3af)

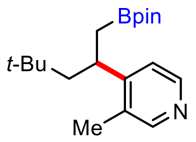

3af

Prepared following **General Procedure E**, using 2,2'-(4,4-dimethylpentane-1,2-diyl)bis(4,4,5,5-tetramethyl-1,3,2-dioxaborolane) (**1a**) (106 mg, 0.300 mmol, 1.50 equiv.), 4CzIPN (7.9 mg, 5.0 mol%), phenyllithium (1.9 M in dibutyl ether, 168 µL, 0.320 mmol, 1.60 equiv.) and 3-methylisonicotinonitrile (**2f**) (23.6 mg, 0.20 mmol, 1.0 equiv.). Purification by flash column chromatography (75:25 pentane/EtOAc) gave boronic ester **3af** (24.7 mg, 39%) as a colorless oil.

$\mathbf{R_f}$ = 0.38 (67:33 *n*-hexane/EtOAc, CAM stain)

NMR Spectroscopy (<u>*see spectra*</u>):

[1]**H NMR** (400 MHz, $CDCl_3$): δ_H 8.32 (d, *J* = 5.2 Hz, 1H), 8.27 (s, 1H), 7.14 (d, *J* = 5.2 Hz, 1H), 3.19 (tdd, *J* = 8.8, 6.8, 3.9 Hz, 1H), 2.40 (s, 3H), 1.74 (dd, *J* = 14.2, 8.7 Hz, 1H), 1.53 (dd, *J* = 14.2, 3.9 Hz, 1H), 1.14 (dd, *J* = 15.5, 6.9 Hz, 1H), 1.06 (s, 6H), 1.06 (s, 6H), 1.06 – 0.99 (m, 1H), 0.77 (s, 9H) ppm;

[13]**C NMR** (101 MHz, $CDCl_3$): δ_C 157.3, 150.5, 147.3, 131.0, 121.5, 83.2, 52.6, 32.4, 31.6, 30.4, 24.9, 24.7, 17.0 ppm. The carbon attached to boron was not observed due to quadrupolar relaxation;

[11]**B NMR** (128 MHz, $CDCl_3$): δ_B 35.2 ppm.

IR (film): ν_{max} 2970, 2954, 1740, 1593, 1468, 1366, 1322, 1216, 1143, 967, 848 cm^{-1}.

HRMS (EI^+): m/z calculated for $C_{19}H_{33}BNO_2$ $[M+H]^+$, 318.2602; found, 318.2618.

UOB Confidential & Sensitive

4-[4,4-Dimethyl-1-(4,4,5,5-tetramethyl-1,3,2-dioxaborolan-2-yl)pentan-2-yl]-2-methylpyridine (3ag)

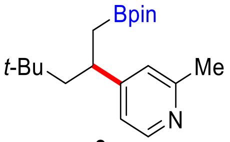

3ag

Prepared following **General Procedure E**, using 2,2'-(4,4-dimethylpentane-1,2-diyl)bis(4,4,5,5-tetramethyl-1,3,2-dioxaborolane) (**1a**) (105.7 mg, 0.30 mmol, 1.5 equiv.), 4CzIPN (7.89 mg, 5.0 mol%), phenyllithium (1.9 M in dibutyl ether, 168 µL, 0.32 mmol, 1.6 equiv.) and 2-methylisonicotinonitrile (**2g**) (23.6 mg, 0.200 mmol, 1.00 equiv.). Purification by flash column chromatography (75:25 pentane/EtOAc) gave boronic ester **3ag** (31.7 mg, 50%) as a colorless oil.

R_f = 0.37 (80:20 *n*-hexane/EtOAc, CAM stain)

NMR Spectroscopy (*see spectra*):

> **^{1}H NMR** (400 MHz, $CDCl_3$): δ_H 8.32 (d, *J* = 5.3 Hz, 1H), 7.04 (s, 1H), 7.00 (dd, *J* = 5.3, 1.7 Hz, 1H), 2.92 (tdd, *J* = 9.0, 7.1, 3.4 Hz, 1H), 2.51 (s, 3H), 1.74 (dd, *J* = 14.1, 9.3 Hz, 1H), 1.52 (dd, *J* = 14.1, 3.4 Hz, 1H), 1.13 (dd, *J* = 15.4, 8.3 Hz, 1H), 1.10 (s, 6H), 1.10 (s, 6H), 1.04 (dd, *J* = 15.4, 8.7 Hz, 1H), 0.76 (s, 9H) ppm;
>
> **^{13}C NMR** (101 MHz, $CDCl_3$): δ_C 159.4, 157.7, 148.5, 123.1, 120.5, 83.3, 52.3, 38.1, 31.6, 30.3, 24.9, 24.8, 24.2 ppm. The carbon attached to boron was not observed due to quadrupolar relaxation;
>
> **^{11}B NMR** (128 MHz, $CDCl_3$): δ_B 32.6 ppm.

IR (film): ν_{max} 2970, 2951, 1740, 1423, 1367, 1229, 1217, 1144, 849, 528 cm^{-1}.

HRMS (EI^+): m/z calculated for $C_{19}H_{32}BNO_2$ $[M]^+$, 317.2521; found, 317.2522.

4-[4,4-Dimethyl-1-(4,4,5,5-tetramethyl-1,3,2-dioxaborolan-2-yl)pentan-2-yl]-2-methoxypyridine (3ah)

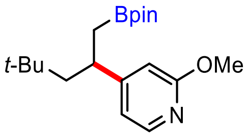

3ah

Prepared following **General Procedure E**, using 2,2'-(4,4-dimethylpentane-1,2-diyl)bis(4,4,5,5-tetramethyl-1,3,2-dioxaborolane) (**1a**) (106 mg, 0.300 mmol, 1.50 equiv.), 4CzIPN (7.9 mg, 5.0 mol%), phenyllithium (1.9 M in dibutyl ether, 168 µL, 0.320 mmol, 1.60 equiv.) and 2-methylisonicotinonitrile (**2h**) (26.8 mg, 0.200 mmol, 1.00 equiv.). Purification by flash column chromatography (91:9 pentane/EtOAc) gave boronic ester **3ah** (28.0 mg, 42%) as a colorless oil.

R_f = 0.34 (91:9 *n*-hexane/EtOAc, CAM stain)

NMR Spectroscopy (*see spectra*):

^{1}**H NMR** (400 MHz, $CDCl_3$): δ_H 8.01 (dd, J = 5.4, 0.6 Hz, 1H), 6.78 (dd, J = 5.4, 1.4 Hz, 1H), 6.62 (d, J = 1.4 Hz, 1H), 3.90 (s, 3H), 2.91 (tdd, J = 9.3, 7.8, 3.2 Hz, 1H), 1.74 (dd, J = 14.1, 9.5 Hz, 1H), 1.49 (dd, J = 14.1, 3.2 Hz, 1H), 1.13 (s, 6H), 1.12 (s, 6H), 1.08 (dd, J = 15.6, 8.2 Hz, 1H), 1.02 (dd, J = 15.4, 8.2 Hz, 1H), 0.77 (s, 9H) ppm;

^{13}**C NMR** (101 MHz, $CDCl_3$): δ_C 164.4, 161.7, 146.4, 116.8, 109.8, 83.3, 53.5, 52.0, 38.0, 31.6, 30.3, 24.9, 24.8 ppm. The carbon attached to boron was not observed due to quadrupolar relaxation;

^{11}**B NMR** (128 MHz, $CDCl_3$): δ_B 33.0 ppm.

IR (film): ν_{max} 2971, 2950, 1740, 1607, 1560, 1449, 1400, 1366, 1318, 1216, 1144, 1044, 847 cm^{-1}.

HRMS (EI$^+$): m/z calculated for $C_{19}H_{32}BNO_3$ [M]$^+$, 333.2470; found, 333.2462.

4-[4,4-Dimethyl-1-(4,4,5,5-tetramethyl-1,3,2-dioxaborolan-2-yl)pentan-2-yl]-2-fluoropyridine (3ai)

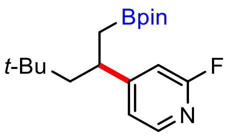

3ai

Prepared following **General Procedure E**, using 2,2'-(4,4-dimethylpentane-1,2-diyl)bis(4,4,5,5-tetramethyl-1,3,2-dioxaborolane) (**1a**) (106 mg, 0.300 mmol, 1.50 equiv.), 4CzIPN (7.9 mg, 5.0 mol%), phenyllithium (1.9 M in dibutyl ether, 168 µL, 0.320 mmol, 1.60 equiv.) and 2-fluoroisonicotinonitrile (**2i**) (24.4 mg, 0.200 mmol, 1.00 equiv.). Purification by flash column chromatography (91:9 pentane/EtOAc) gave boronic ester **3ai** (30.8 mg, 48%) as a colorless oil.

R*f* = 0.21 (91:9 *n*-hexane/EtOAc, CAM stain)

NMR Spectroscopy (*see spectra*):

^{1}**H NMR** (400 MHz, $CDCl_3$): δ_H 8.05 (d, J = 5.2 Hz, 1H), 7.06 (ddd, J = 5.2, 2.2, 1.4 Hz, 1H), 6.80 (s, 1H), 3.00 (tdd, J = 12.1, 3.4, 3.0 Hz, 1H), 1.74 (dd, J = 14.2, 9.3 Hz, 1H), 1.54 (dd, J = 14.2, 3.4 Hz, 1H), 1.20 – 0.98 (m, 14H), 0.77 (s, 9H) ppm;

^{13}**C NMR** (101 MHz, $CDCl_3$): δ_C 165.2 (d, ^3J = 27.5 Hz), 163.9 (d, $^1J_{C-F}$ = 203.2 Hz), 147.3 (d, $^3J_{C-F}$ = 15.3 Hz), 121.0 (d, $^4J_{C-F}$ = 3.7 Hz), 108.6 (d, $^2J_{C-F}$ = 36.5 Hz), 83.4, 52.3, 38.1 (d, $^4J_{C-F}$ = 2.8 Hz), 31.6, 30.3, 24.9, 24.8 ppm. The carbon attached to boron was not observed due to quadrupolar relaxation;

^{19}**F NMR** (377 MHz, $CDCl_3$): δ_F -69.49 ppm;

^{11}**B NMR** (128 MHz, $CDCl_3$): δ_B 33.0 ppm.

IR (film): ν_{max} 2970, 2953, 1740, 1609, 1568, 1413, 1368, 1324, 1216, 1144, 968, 848 cm^{-1}.

HRMS (EI$^+$): m/z calculated for $C_{18}H_{29}BFNO_2$ [M]$^+$, 321.2270; found, 321.2269.

UOB Confidential & Sensitive

4-[4,4-Dimethyl-1-(4,4,5,5-tetramethyl-1,3,2-dioxaborolan-2-yl)pentan-2-yl]-2-phenylpyridine (3aj)

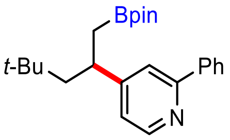

3aj

Prepared following **General Procedure E**, using 2,2'-(4,4-dimethylpentane-1,2-diyl)bis(4,4,5,5-tetramethyl-1,3,2-dioxaborolane) (**1a**) (106 mg, 0.300 mmol, 1.50 equiv.), 4CzIPN (7.9 mg, 5.0 mol%), phenyllithium (1.9 M in dibutyl ether, 168 µL, 0.320 mmol, 1.60 equiv.) and 2-phenylisonicotinonitrile (**2j**) (36.1 mg, 0.200 mmol, 1.00 equiv.). Purification by flash column chromatography (89:11 pentane/EtOAc) gave boronic ester **3aj** (29.0 mg, 38%) as a colorless oil.

R*f* = 0.26 (91:9 *n*-hexane/EtOAc, CAM stain)

NMR Spectroscopy (*see spectra*):

^{1}H NMR (400 MHz, $CDCl_3$): δ_H 8.55 (dd, *J* = 5.1, 0.9 Hz, 1H), 8.03 – 7.97 (m, 2H), 7.64 (d, *J* = 0.9 Hz, 1H), 7.51 – 7.42 (m, 2H), 7.43 – 7.38 (m, 1H), 7.15 (dd, *J* = 5.2, 1.6 Hz, 1H), 3.05 (tdd, *J* = 9.0, 7.1, 3.4 Hz, 1H), 1.82 (dd, *J* = 14.1, 9.3 Hz, 1H), 1.58 (dd, *J* = 14.1, 3.4 Hz, 1H), 1.27 – 1.04 (m, 2H), 1.09 (s, 12H), 0.80 (s, 9H) ppm;

^{13}C NMR (101 MHz, $CDCl_3$): δ_C 159.6, 157.3, 149.5, 139.8, 129.0, 128.9, 127.2, 121.9, 120.6, 83.4, 52.6, 38.4, 31.7, 30.5, 25.0, 24.9 ppm. The carbon attached to boron was not observed due to quadrupolar relaxation;

^{11}B NMR (128 MHz, $CDCl_3$): δ_B 33.2 ppm.

IR (film): ν_{max} 2953, 1598, 1554, 1474, 1446, 1407, 1364, 1322, 1143, 967, 845, 776, 732, 695 cm^{-1}.

HRMS (EI$^+$): m/z calculated for $C_{24}H_{34}BNO_2$ [M]$^+$, 379.2677; found, 379.2674.

4-[4,4-Dimethyl-1-(4,4,5,5-tetramethyl-1,3,2-dioxaborolan-2-yl)pentan-2-yl]-2-(*p*-tolyl)pyridine (3ak)

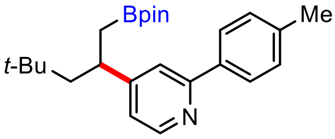

3ak

Prepared following **General Procedure E**, using 2,2'-(4,4-dimethylpentane-1,2-diyl)bis(4,4,5,5-tetramethyl-1,3,2-dioxaborolane) (**1a**) (106 mg, 0.300 mmol, 1.50 equiv.), 4CzIPN (7.9 mg, 5.0 mol%), phenyllithium (1.9 M in dibutyl ether, 168 µL, 0.320 mmol, 1.60 equiv.) and 2-(*p*-tolyl)isonicotinonitrile (**2k**) (38.9 mg, 0.200 mmol, 1.00 equiv.). Purification by flash column chromatography (89:11 pentane/EtOAc) gave boronic ester **3ak** (26.8 mg, 38%) as a colorless oil.

R*f* = 0.29 (91:9 *n*-hexane/EtOAc, CAM stain)

UOB Confidential & Sensitive

NMR Spectroscopy (see spectra):

^{1}H NMR (400 MHz, $CDCl_3$): δ_H 8.53 (dd, *J* = 5.2, 0.7 Hz, 1H), 7.90 (d, *J* = 8.2 Hz, 2H), 7.62 (d, *J* = 0.9 Hz, 1H), 7.28 (d, *J* = 7.7 Hz, 2H), 7.13 (dd, *J* = 5.2, 1.6 Hz, 1H), 3.04 (tdd, *J* = 8.9, 7.1, 3.4 Hz, 1H), 2.41 (s, 3H), 1.81 (dd, *J* = 14.1, 9.3 Hz, 1H), 1.58 (dd, *J* = 14.1, 3.4 Hz, 1H), 1.20 (dd, *J* = 15.5, 7.2 Hz, 1H), 1.13 (dd, *J* = 15.6, 8.7 Hz, 1H), 1.09 (s, 12H), 0.80 (s, 9H) ppm；

^{13}C NMR (101 MHz, $CDCl_3$): δ_C 160.0, 156.9, 148.9, 139.1, 129.6, 127.0, 125.7, 121.5, 120.4, 83.3, 52.5, 38.4, 31.7, 30.4, 24.9, 24.8, 21.4 ppm. The carbon attached to boron was not observed due to quadrupolar relaxation;

^{11}B NMR (128 MHz, $CDCl_3$): δ_B 32.9 ppm.

IR (film): ν_{max} 2970, 2951, 1740, 1599, 1470, 1366, 1322, 1217, 1143, 967, 846, 819 cm^{-1}.

HRMS (EI^+): m/z calculated for $C_{25}H_{36}BNO_2$ $[M]^+$, 393.2834; found, 393.2828.

4-[4,4-Dimethyl-1-(4,4,5,5-tetramethyl-1,3,2-dioxaborolan-2-yl)pentan-2-yl]-2-(3,5-dimethylphenyl)pyridine (3al)

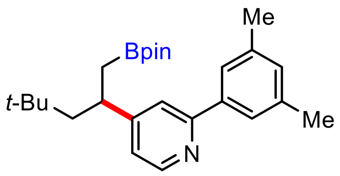

3al

Prepared following **General Procedure E**, using 2,2'-(4,4-dimethylpentane-1,2-diyl)bis(4,4,5,5-tetramethyl-1,3,2-dioxaborolane) (**1a**) (106 mg, 0.300 mmol, 1.50 equiv.), 4CzIPN (7.9 mg, 5.0 mol%), phenyllithium (1.9 M in dibutyl ether, 168 µL, 0.320 mmol, 1.60 equiv.) and 2-(3,5-dimethylphenyl)isonicotinonitrile (**2l**) (41.7 mg, 0.200 mmol, 1.00 equiv.). Purification by flash column chromatography (90:10 pentane/EtOAc) gave boronic ester **3al** (36.0 mg, 44%) as a colorless oil.

R_f = 0.28 (91:9 *n*-hexane/EtOAc, CAM stain)

NMR Spectroscopy (see spectra):

^{1}H NMR (400 MHz, $CDCl_3$): δ_H 8.53 (dd, *J* = 5.1, 0.8 Hz, 1H), 7.69 – 7.58 (m, 3H), 7.13 (dd, *J* = 5.2, 1.7 Hz, 1H), 7.05 (t, *J* = 0.8 Hz, 1H), 3.04 (tdd, *J* = 9.0, 7.2, 3.3 Hz, 1H), 2.40 (s, 6H), 1.82 (dd, *J* = 14.2, 9.3 Hz, 1H), 1.58 (dd, *J* = 14.1, 3.4 Hz, 1H), 1.20 (dd, *J* = 15.5, 7.3 Hz, 1H), 1.13 (d, *J* = 15.5, 8.7 Hz, 1H), 1.10 (s, 6H), 1.10 (s, 6H), 0.80 (s, 9H) ppm;

^{13}C NMR (101 MHz, $CDCl_3$): δ_C 159.6, 157.3, 149.1, 139.3, 138.4, 130.7, 124.9, 121.6, 120.6, 83.3, 52.5, 38.4, 31.7, 30.4, 24.9, 24.8, 21.5 ppm. The carbon attached to boron was not observed due to quadrupolar relaxation;

^{11}B NMR (128 MHz, $CDCl_3$): δ_B 33.0 ppm.

IR (film): ν_{max} 2970, 2951, 1740, 1595, 1554, 1366, 1217, 1144, 967, 849, 703, 528 cm^{-1}.

HRMS (EI$^+$): m/z calculated for $C_{26}H_{38}BNO_2$ [M]$^+$, 407.2990; found, 407.2985.

4-[4,4-Dimethyl-1-(4,4,5,5-tetramethyl-1,3,2-dioxaborolan-2-yl)pentan-2-yl]-2-(3-fluorophenyl)pyridine (3am)

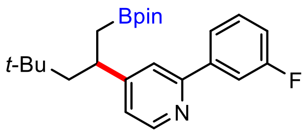

3am

Prepared following **General Procedure E**, using 2,2'-(4,4-dimethylpentane-1,2-diyl)bis(4,4,5,5-tetramethyl-1,3,2-dioxaborolane) (**1a**) (106 mg, 0.300 mmol, 1.50 equiv.), 4CzIPN (7.9 mg, 5.0 mol%), phenyllithium (1.9 M in dibutyl ether, 168 µL, 0.320 mmol, 1.60 equiv.) and 2-(3,5-dimethylphenyl)isonicotinonitrile (**2m**) (39.6 mg, 0.200 mmol, 1.00 equiv.). Purification by flash column chromatography (89:11 pentane/EtOAc) gave boronic ester **3am** (34.2 mg, 43%) as a colorless oil.

R*f* = 0.29 (91:9 *n*-hexane/EtOAc, CAM stain)

NMR Spectroscopy (*see spectra*):

^{1}**H NMR** (400 MHz, $CDCl_3$): δ_H 8.54 (dd, J = 5.1, 0.8 Hz, 1H), 7.77 (dt, J = 7.8, 1.3 Hz, 1H), 7.72 (ddd, J = 10.4, 2.6, 1.6 Hz, 1H), 7.62 (s, 1H), 7.43 (td, J = 8.0, 5.9 Hz, 1H), 7.17 (dd, J = 5.1, 1.6 Hz, 1H), 7.09 (tdd, J = 8.3, 2.6, 0.9 Hz, 1H), 3.05 (tdd, J = 8.9, 7.0, 3.4 Hz, 1H), 1.81 (dd, J = 14.1, 9.2 Hz, 1H), 1.59 (dd, J = 14.2, 3.4 Hz, 1H), 1.20 (dd, J = 15.6, 7.1 Hz, 1H), 1.12 (dd, J = 15.5, 8.8 Hz, 1H), 1.09 (s, 12H), 0.80 (s, 9H) ppm;

^{13}**C NMR** (101 MHz, $CDCl_3$): δ_C 164.7, 162.3, 161.3 (d, $^{1}J_{C\text{-}F}$ = 271.7 Hz), 155.8, 149.4, 130.3 (d, $^{3}J_{C\text{-}F}$ = 8.2 Hz), 122.6 (d, $^{3}J_{C\text{-}F}$ = 3.0 Hz), 122.3, 120.6, 115.8 (d, $^{2}J_{C\text{-}F}$ = 21.3 Hz), 114.1 (d, $^{2}J_{C\text{-}F}$ = 22.7 Hz), 83.3, 52.5, 38.3, 31.7, 30.4, 24.9, 24.8 ppm. The carbon attached to boron was not observed due to quadrupolar relaxation;

^{19}**F NMR** (377 MHz, $CDCl_3$): δ_F -113.06 ppm;

^{11}**B NMR** (128 MHz, $CDCl_3$): δ_B 33.3 ppm.

IR (film): ν_{max} 2970, 2951, 1740, 1587, 1449, 1367, 1264, 1217, 1143, 788, 733, 699, 527 cm^{-1}.

HRMS (EI$^+$): m/z calculated for $C_{24}H_{33}BFNO_2$ [M]$^+$, 397.2853; found, 397.2577.

4-Benzylbenzonitrile (6aa)

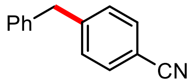

6aa

Prepared following **General Procedure A**, using 2-benzyl-4,4,5,5-tetramethyl-1,3,2-dioxaborolane (**5a**) (65 mg, 0.30 mmol, 1.5 equiv.), 4-bromo-*N,N*-dimethylaniline (64 mg, 0.32 mmol, 1.6 equiv.), *tert*-butyllithium (1.7 M in pentane, 376 µL, 0.640 mmol, 3.20 equiv.) and terephthalonitrile (**4a**) (25.6 mg, 0.200 mmol, 1.00 equiv.). Purification by flash column chromatography (91:9 pentane/diethyl ether) gave the title compound **6aa** (22.8 mg, 59%) as a colorless oil.

R_f = 0.32 (91:9 *n*-hexane/EtOAc, $KMnO_4$ stain)

NMR Spectroscopy (*see spectra*):

^{1}**H NMR** (400 MHz, $CDCl_3$): δ_H 7.59 (d, *J* = 8.1 Hz, 2H), 7.38 – 7.23 (m, 5H), 7.18 (d, *J* = 7.1 Hz, 2H), 4.05 (s, 2H) ppm;

^{13}**C NMR** (101 MHz, $CDCl_3$): δ_C 146.9, 139.5, 132.4, 129.8, 129.1, 128.9, 126.8, 119.1, 110.2, 42.1 ppm.

IR (film): ν_{max} 3027, 2970, 2227, 1740, 1603, 1495, 1453, 1366, 1217, 1029, 911, 797, 760, 725, 697 cm^{-1}.

All recorded spectroscopic data matched those previously reported in the literature.[33]

8-(4-Cyanophenyl)octyl 2,4,6-triisopropylbenzoate (6ba)

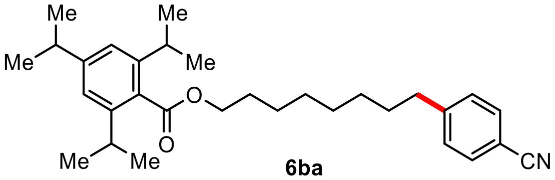

6ba

Prepared following **General Procedure D**, using 8-(4,4,5,5-tetramethyl-1,3,2-dioxaborolan-2-yl)octyl 2,4,6-triisopropylbenzoate (**5b**) (142 mg, 0.300 mmol, 1.50 equiv.), 4-bromo-*N,N*-dimethylaniline (64 mg, 0.32 mmol, 1.6 equiv.), *tert*-butyllithium (1.7 M in pentane, 376 µL, 0.640 mmol, 3.20 equiv.) and terephthalonitrile (**4a**) (25.6 mg, 0.200 mmol, 1.00 equiv.). Purification by flash column chromatography (91:9 pentane/diethyl ether) gave the title compound **6ba** (67.4 mg, 73%) as a colorless oil.

R_f = 0.30 (91:9 *n*-hexane/EtOAc, $KMnO_4$ stain)

NMR Spectroscopy (*see spectra*):

^{1}**H NMR** (400 MHz, $CDCl_3$): δ_H 7.56 (d, *J* = 8.2 Hz, 2H), 7.26 (d, *J* = 8.2 Hz, 2H), 7.01 (s, 2H), 4.30 (t, *J* = 6.6 Hz, 2H), 2.95 – 2.81 (m, 3H), 2.65 (dd, *J* = 8.7, 6.8 Hz, 2H), 1.72 (dd, *J* = 8.1, 6.4 Hz, 2H), 1.66 – 1.55 (m, 2H), 1.46 – 1.29 (m, 8H), 1.25 (d, *J* = 6.9 Hz, 18H) ppm;

^{13}**C NMR** (101 MHz, $CDCl_3$): δ_C 171.1, 150.2, 148.6, 144.8, 132.3, 130.8, 129.3, 121.0, 119.3, 109.7, 65.1,

36.2, 34.6, 31.6, 31.0, 29.4, 29.2, 29.2, 28.8, 26.2, 24.3, 24.1 ppm.

IR (film): ν_{max} 2970, 2930, 2227, 1737, 1726, 1461, 1367, 1217, 1229, 1076, 527 cm^{-1}.

HRMS (ESI$^+$): m/z calculated for $C_{31}H_{43}NNaO_2$ [M+Na]$^+$, 484.3186; found, 484.3179.

4-(3-Cyanopropyl)benzonitrile (6ca)

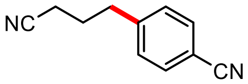

6ca

Prepared following **General Procedure D**, using 4-(4,4,5,5-tetramethyl-1,3,2-dioxaborolan-2-yl)butanenitrile (**5c**) (59 mg, 0.30 mmol, 1.5 equiv.), 4-bromo-*N,N*-dimethylaniline (64 mg, 0.32 mmol, 1.6 equiv.), *tert*-butyllithium (1.7 M in pentane, 376 µL, 0.640 mmol, 3.20 equiv.) and terephthalonitrile (**4a**) (25.6 mg, 0.200 mmol, 1.00 equiv.). Purification by flash column chromatography (67:33 pentane/EtOAc) gave the title compound **6ca** (15.0 mg, 44%) as a colorless oil.

R*f* = 0.36 (67:33 *n*-hexane/EtOAc, $KMnO_4$ stain)

NMR Spectroscopy (*see spectra*):

^{1}**H NMR** (400 MHz, $CDCl_3$): δ_H 7.61 (d, *J* = 8.3 Hz, 2H), 7.31 (d, *J* = 8.3 Hz, 2H), 2.85 (t, *J* = 7.6 Hz, 2H), 2.35 (t, *J* = 7.0 Hz, 2H), 2.06 – 1.94 (m, 2H) ppm;

^{13}**C NMR** (101 MHz, $CDCl_3$): δ_C 145.4, 132.7, 129.4, 119.1, 118.9, 110.8, 34.6, 26.5, 16.7 ppm.

IR (film): ν_{max} 2970, 2945, 2228, 1740, 1608, 1506, 1425, 1368, 1229, 1217, 843, 814, 553 cm^{-1}.

All recorded spectroscopic data matched those previously reported in the literature.[34]

4-[2-(9*H*-Carbazol-9-yl)ethyl]benzonitrile (6da)

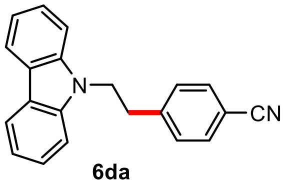

6da

Prepared following **General Procedure D**, using 9-[2-(4,4,5,5-tetramethyl-1,3,2-dioxaborolan-2-yl)ethyl]-9*H*-carbazole (**5d**) (96 mg, 0.30 mmol, 1.5 equiv.), 4-bromo-*N,N*-dimethylaniline (64 mg, 0.32 mmol, 1.6 equiv.), *tert*-butyllithium (1.7 M in pentane, 376 µL, 0.640 mmol, 3.20 equiv.) and terephthalonitrile (**4a**) (25.6 mg, 0.200 mmol, 1.00 equiv.). Purification by flash column chromatography (91:9 pentane/diethyl ether) gave the title compound **6da** (37.4 mg, 63%) as a colorless oil.

R*f* = 0.21 (91:9 *n*-hexane/EtOAc, $KMnO_4$ stain)

NMR Spectroscopy (see spectra):

^{1}H NMR (400 MHz, $CDCl_3$): δ_H 8.09 (d, J = 7.7 Hz, 2H), 7.47 (d, J = 8.2 Hz, 2H), 7.41 (ddd, J = 8.2, 7.2, 1.2 Hz, 2H), 7.27 – 7.18 (m, 4H), 7.15 (d, J = 8.2 Hz, 2H), 4.54 (t, J = 7.1 Hz, 2H), 3.20 (t, J = 7.1 Hz, 2H) ppm;

^{13}C NMR (101 MHz, $CDCl_3$): δ_C 144.4, 140.2, 132.5, 129.8, 125.9, 123.1, 120.6, 119.3, 118.9, 110.8, 108.5, 44.3, 35.5 ppm.

IR (film): ν_{max} 2970, 2228, 1740, 1454, 1366, 1352, 1229, 1217, 751, 723, 550 cm^{-1}.

All recorded spectroscopic data matched those previously reported in the literature.[35]

4-(3-Phenoxypropyl)benzonitrile (6ea)

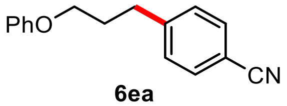

6ea

Prepared following **General Procedure D**, using 4,4,5,5-tetramethyl-2-(3-phenoxypropyl)-1,3,2-dioxaborolane (**5e**) (79 mg, 0.30 mmol, 1.5 equiv.), 4-bromo-*N,N*-dimethylaniline (64 mg, 0.32 mmol, 1.6 equiv.), *tert*-butyllithium (1.7 M in pentane, 376 µL, 0.640 mmol, 3.20 equiv.) and terephthalonitrile (**4a**) (25.6 mg, 0.200 mmol, 1.00 equiv.). Purification by flash column chromatography (91:9 pentane/diethyl ether) gave the title compound **6ea** (21.8 mg, 46%) as a colorless oil.

R_f = 0.24 (91:9 *n*-hexane/EtOAc, $KMnO_4$ stain)

NMR Spectroscopy (see spectra):

^{1}H NMR (400 MHz, $CDCl_3$): δ_H 7.57 (d, J = 8.3 Hz, 2H), 7.33 – 7.22 (m, 4H), 6.94 (tt, J = 7.3, 1.1 Hz, 1H), 6.87 (dd, J = 8.8, 1.1 Hz, 2H), 3.95 (t, J = 6.1 Hz, 2H), 2.88 (dd, J = 8.5, 6.8 Hz, 2H), 2.15 – 2.04 (m, 2H) ppm;

^{13}C NMR (101 MHz, $CDCl_3$): δ_C 158.9, 147.4, 132.4, 129.6, 129.5, 121.0, 119.2, 114.6, 110.1, 66.4, 32.6, 30.5 ppm.

IR (film): ν_{max} 2942, 2871, 2227, 1601, 1497, 1245, 1173, 1044, 816, 755, 692 cm^{-1}.

All recorded spectroscopic data matched those previously reported in the literature.[36]

4-Phenethylbenzonitrile (6fa)

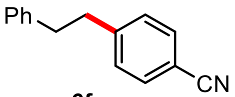

6fa

Prepared following **General Procedure D**, using 4,4,5,5-tetramethyl-2-phenethyl-1,3,2-dioxaborolane (**5f**) (70 mg, 0.30 mmol, 1.5 equiv.), 4-bromo-*N,N*-dimethylaniline (64 mg, 0.32 mmol, 1.6 equiv.), *tert*-butyllithium (1.7 M in pentane, 376 µL, 0.640 mmol, 3.20 equiv.) and terephthalonitrile (**4a**) (25.6 mg, 0.200 mmol, 1.00 equiv.). Purification by flash column chromatography (91:9 pentane/diethyl ether) gave the title compound **6fa** (22.8 mg, 55%) as a colorless oil.

R*f* = 0.34 (91:9 *n*-hexane/EtOAc, $KMnO_4$ stain)

NMR Spectroscopy (*see spectra*):

^{1}H NMR (400 MHz, $CDCl_3$): δ_H 7.55 (d, *J* = 8.3 Hz, 2H), 7.33 – 7.17 (m, 5H), 7.15 – 7.11 (m, 2H), 3.02 – 2.90 (m, 4H) ppm;

^{13}C NMR (101 MHz, $CDCl_3$): δ_C 147.4, 140.7, 132.3, 129.5, 128.6, 128.6, 126.4, 119.2, 110.0, 38.0, 37.3 ppm.

IR (film): v_{max} 3027, 2970, 2227, 1740, 1605, 1454, 1366, 1217, 824, 750, 700, 551 cm^{-1}.

All recorded spectroscopic data matched those previously reported in the literature.[37]

4-{2-([1,1'-Biphenyl]-4-yl)ethyl}benzonitrile (6ga)

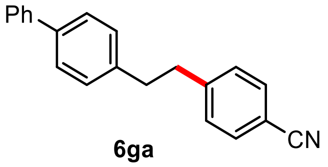

6ga

Prepared following **General Procedure D**, using 2-(2-([1,1'-biphenyl]-4-yl)ethyl)-4,4,5,5-tetramethyl-1,3,2-dioxaborolane (**5g**) (93 mg, 0.30 mmol, 1.5 equiv.), 4-bromo-*N,N*-dimethylaniline (64 mg, 0.32 mmol, 1.6 equiv.), *tert*-butyllithium (1.7 M in pentane, 376 µL, 0.640 mmol, 3.20 equiv.) and terephthalonitrile (**4a**) (25.6 mg, 0.200 mmol, 1.00 equiv.). Purification by flash column chromatography (91:9 pentane/diethyl ether) gave the title compound **6ga** (30.1 mg, 53%) as a colorless oil.

R*f* = 0.28 (91:9 *n*-hexane/EtOAc, $KMnO_4$ stain)

NMR Spectroscopy (*see spectra*):

^{1}H NMR (400 MHz, $CDCl_3$): δ_H 7.63 – 7.56 (m, 4H), 7.56 – 7.52 (m, 2H), 7.48 – 7.42 (m, 2H), 7.38 – 7.33 (m, 1H), 7.30 – 7.27 (m, 2H), 7.20 (d, *J* = 8.2 Hz, 2H), 3.07 – 2.94 (m, 4H) ppm;

^{13}C NMR (101 MHz, $CDCl_3$): δ_C 147.3, 141.0, 139.8, 139.4, 132.3, 129.5, 129.0, 128.9, 127.3, 127.1, 119.2, 110.1, 38.0, 37.0 ppm.

IR (film): v_{max} 2970, 2224, 1740, 1487, 1366, 1229, 1217, 838, 770, 699, 548 cm^{-1}.

HRMS (ACPI$^+$): m/z calculated for $C_{21}H_{18}N$ $[M+H]^+$, 284.1434; found, 284.1433.

4-Cyclohexylbenzonitrile (6ha)

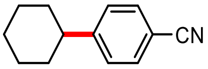

6ha

Prepared following **General Procedure D**, using 2-cyclohexyl-4,4,5,5-tetramethyl-1,3,2-dioxaborolane (**5h**) (63 mg, 0.30 mmol, 1.5 equiv.), 4-bromo-*N,N*-dimethylaniline (64 mg, 0.32 mmol, 1.6 equiv.), *tert*-butyllithium (1.7 M in pentane, 376 µL, 0.640 mmol, 3.20 equiv.) and terephthalonitrile (**4a**) (25.6 mg, 0.200 mmol, 1.00 equiv.). Purification by flash column chromatography (95:5 pentane/diethyl ether) gave the title compound **6ha** (26.7 mg, 72%) as a colorless oil.

R_f = 0.49 (91:9 *n*-hexane/EtOAc, $KMnO_4$ stain)

NMR Spectroscopy (*see spectra*):

^{1}H NMR (400 MHz, $CDCl_3$): δ_H 7.57 (d, J = 8.3 Hz, 2H), 7.29 (d, J = 8.3 Hz, 2H), 2.60 – 2.51 (m, 1H), 1.90 – 1.82 (m, 4H), 1.77 (ddd, J = 12.2, 2.9, 1.4 Hz, 1H), 1.48 – 1.33 (m, 4H), 1.32 – 1.19 (m, 1H) ppm;

^{13}C NMR (101 MHz, $CDCl_3$): δ_C 153.6, 132.3, 127.8, 119.3, 109.7, 44.9, 34.1, 26.7, 26.1 ppm.

IR (film): ν_{max} 2925, 2852, 2226, 1607, 1504, 1448, 1176, 999, 829, 562 cm^{-1}.

All recorded spectroscopic data matched those previously reported in the literature.[38]

4-Cycloheptylbenzonitrile (6ia)

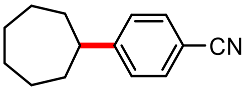

6ia

Prepared following **General Procedure D**, using 2-cycloheptyl-4,4,5,5-tetramethyl-1,3,2-dioxaborolane (**5i**) (67 mg, 0.30 mmol, 1.5 equiv.), 4-bromo-*N,N*-dimethylaniline (64 mg, 0.32 mmol, 1.6 equiv.), *tert*-butyllithium (1.7 M in pentane, 376 µL, 0.640 mmol, 3.20 equiv.) and terephthalonitrile (**4a**) (25.6 mg, 0.200 mmol, 1.00 equiv.). Purification by flash column chromatography (95:5 pentane/diethyl ether) gave the title compound **6ia** (29.1 mg, 73%) as a colorless oil.

R_f = 0.49 (91:9 *n*-hexane/EtOAc, CAM stain)

NMR Spectroscopy (*see spectra*):

^{1}H NMR (400 MHz, $CDCl_3$): δ_H 7.55 (d, J = 8.4 Hz, 2H), 7.27 (d, J = 8.3 Hz, 2H), 2.71 (tt, J = 10.3, 3.5 Hz, 1H), 1.94 – 1.75 (m, 4H), 1.75 – 1.48 (m, 8H) ppm;

^{13}C NMR (101 MHz, $CDCl_3$): δ_C 155.5, 132.4, 127.7, 119.4, 109.5, 47.3, 36.5, 27.9, 27.3 ppm.

IR (film): ν_{max} 2922, 2854, 2226, 1739, 1607, 1503, 1445, 1368, 1217, 910, 817, 732, 562 cm^{-1}.

All recorded spectroscopic data matched those previously reported in the literature.[38]

UOB Confidential & Sensitive

tert-Butyl 4-(4-cyanophenyl)piperidine-1-carboxylate (6ja)

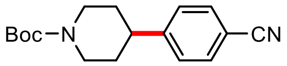

6ja

Prepared following **General Procedure D**, using *tert*-butyl 4-(4,4,5,5-tetramethyl-1,3,2-dioxaborolan-2-yl)piperidine-1-carboxylate (**5j**) (93 mg, 0.30 mmol, 1.5 equiv.), 4-bromo-*N,N*-dimethylaniline (64 mg, 0.32 mmol, 1.60 equiv.), *tert*-butyllithium (1.7 M in pentane, 376 µL, 0.640 mmol, 3.20 equiv.) and terephthalonitrile (**4a**) (25.6 mg, 0.200 mmol, 1.00 equiv.). Purification by flash column chromatography (85:15 pentane/diethyl ether) gave the title compound **6ja** (48.7 mg, 85%) as a colorless oil.

R_f = 0.33 (86:14 *n*-hexane/EtOAc, $KMnO_4$ stain)

NMR Spectroscopy (*see spectra*):

^{1}H NMR (400 MHz, $CDCl_3$): δ_H 7.59 (d, J = 8.4 Hz, 2H), 7.30 (d, J = 8.4 Hz, 2H), 4.26 (dt, J = 13.4, 2.6 Hz, 2H), 2.80 (ddd, J = 13.4, 12.2, 2.6 Hz, 2H), 2.70 (tt, J = 12.2, 3.6 Hz, 1H), 1.87 – 1.77 (m, 2H), 1.60 (qd, J = 12.2, 4.3 Hz, 2H), 1.47 (s, 9H) ppm;

^{13}C NMR (101 MHz, $CDCl_3$): δ_C 154.9, 151.3, 132.5, 127.8, 119.0, 110.4, 79.8, 44.2, 43.0, 32.9, 28.6 ppm.

IR (film): ν_{max} 2970, 2941, 2227, 1740, 1689, 1423, 1366, 1231, 1217, 1167, 1127, 1013, 838 cm^{-1}.

All recorded spectroscopic data matched those previously reported in the literature.[39]

tert-Butyl 2-(4-cyanophenyl)pyrrolidine-1-carboxylate (6ka)

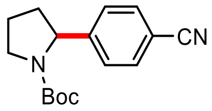

6ka

Prepared following **General Procedure D**, using *tert*-butyl 2-(4,4,5,5-tetramethyl-1,3,2-dioxaborolan-2-yl)pyrrolidine-1-carboxylate (**5k**) (89 mg, 0.30 mmol, 1.5 equiv.), 4-bromo-*N,N*-dimethylaniline (64 mg, 0.32 mmol, 1.6 equiv.), *tert*-butyllithium (1.7 M in pentane, 376 µL, 0.640 mmol, 3.20 equiv.) and terephthalonitrile (**4a**) (25.6 mg, 0.200 mmol, 1.00 equiv.). Purification by flash column chromatography (85:15 pentane/diethyl ether) gave the title compound **6ka** (46.3 mg, 85%) as a colorless oil.

R_f = 0.30 (86:14 *n*-hexane/EtOAc, $KMnO_4$ stain)

NMR Spectroscopy (*see spectra*):

^{1}H NMR (400 MHz, $CDCl_3$): δ_H 7.58 (d, J = 8.4 Hz, 2H), 7.27 (d, J = 8.2 Hz, 2H), 5.00 – 4.66 (m, 1H), 3.61 (s, 2H), 2.34 (s, 1H), 1.87 (dt, J = 13.5, 6.6 Hz, 2H), 1.76 (dtd, J = 12.4, 6.2, 4.4 Hz, 1H), 11.56 – 1.00 (m,

9) ppm;

^{13}C NMR (101 MHz, $CDCl_3$): δ_C 154.4, 150.9, 150.0 (rotamer), 132.3, 126.3, 119.0, 110.5, 79.8, 61.2, 60.9 (rotamer), 47.4, 36.0, 34.9 (rotamer), 28.3, 23.5 ppm.

IR (film): ν_{max} 2971, 2878, 2227, 1738, 1689, 1387, 1365, 1217, 1157, 1111, 832, 775, 550 cm^{-1}.

All recorded spectroscopic data matched those previously reported in the literature.[33]

4-[1-(4-Chlorophenyl)ethyl]benzonitrile (6la)

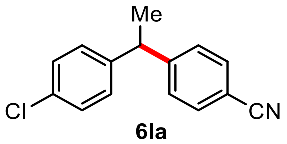

6la

Prepared following **General Procedure D**, using 2-[1-(4-chlorophenyl)ethyl]-4,4,5,5-tetramethyl-1,3,2-dioxaborolane (**5l**) (80 mg, 0.30 mmol, 1.5 equiv.), 4-bromo-*N,N*-dimethylaniline (64 mg, 0.32 mmol, 1.6 equiv.), *tert*-butyllithium (1.7 M in pentane, 376 µL, 0.640 mmol, 3.20 equiv.) and terephthalonitrile (**4a**) (25.6 mg, 0.200 mmol, 1.00 equiv.). Purification by flash column chromatography (95:5 pentane/diethyl ether) gave the title compound **6la** (23.7 mg, 49%) as a colorless oil.

R_f = 0.38 (91:9 *n*-hexane/EtOAc, $KMnO_4$ stain)

NMR Spectroscopy (*see spectra*):

^{1}H NMR (400 MHz, $CDCl_3$): δ_H 7.58 (d, *J* = 8.4 Hz, 2H), 7.28 (dd, *J* = 8.4, 5.2 Hz, 4H), 7.11 (d, *J* = 8.4 Hz, 2H), 4.17 (q, *J* = 7.2 Hz, 1H), 1.63 (d, *J* = 7.2 Hz, 3H) ppm;

^{13}C NMR (101 MHz, $CDCl_3$): δ_C 151.4, 143.3, 132.6, 132.5, 129.1, 128.9, 128.5, 119.0, 110.4, 44.4, 21.5 ppm.

IR (film): ν_{max} 2970, 2931, 2228, 1739, 1607, 1491, 1366, 1229, 1217, 1092, 1014, 832, 528 cm^{-1}.

HRMS (APCI$^+$): m/z calculated for $C_{15}H_{13}NCl$ $[M+H]^+$, 242.0731; found, 242.0720.

4-(Heptan-2-yl)benzonitrile (6ma)

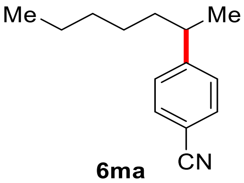

6ma

Prepared following **General Procedure D**, using 2-(heptan-2-yl)-4,4,5,5-tetramethyl-1,3,2-dioxaborolane (**5m**) (82 mg, 0.30 mmol, 1.5 equiv.), 4-bromo-*N,N*-dimethylaniline (64 mg, 0.32 mmol, 1.6 equiv.), *tert*-butyllithium

UOB Confidential & Sensitive

(1.7 M in pentane, 376 µL, 0.640 mmol, 3.20 equiv.) and terephthalonitrile (**4a**) (25.6 mg, 0.200 mmol, 1.00 equiv.). Purification by flash column chromatography (95:5 pentane/diethyl ether) gave the title compound **6ma** (33.8 mg, 84%) as a colorless oil.

R*f* = 0.49 (91:9 *n*-hexane/EtOAc, $KMnO_4$ stain)

NMR Spectroscopy (*see spectra*):

^{1}H NMR (400 MHz, $CDCl_3$): δ_H 7.55 (d, *J* = 8.3 Hz, 2H), 7.25 (d, *J* = 8.4 Hz, 2H), 2.71 (q, *J* = 7.1 Hz, 1H), 1.54 (q, *J* = 7.2 Hz, 2H), 1.30 – 1.00 (m, 9H), 0.82 (t, *J* = 6.8 Hz, 3H) ppm;

^{13}C NMR (101 MHz, $CDCl_3$): δ_C 153.7, 132.3, 128.0, 119.3, 109.8, 40.4, 38.1, 31.9, 27.3, 22.6, 22.0, 14.1 ppm.

IR (film): ν_{max} 2957, 2927, 2227, 1740, 1608, 1504, 1455, 1217, 1206, 834, 733, 568 cm^{-1}.

HRMS ($APCI^+$): m/z calculated for $C_{14}H_{20}N$ $[M+H]^+$, 202.1590; found, 202.1589.

4-(1-Cyano-5-phenylpentan-3-yl)benzonitrile (6na)

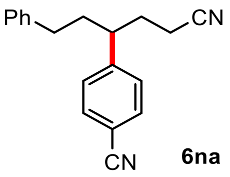

6na

Prepared following **General Procedure D**, using 6-phenyl-4-(4,4,5,5-tetramethyl-1,3,2-dioxaborolan-2-yl)hexanenitrile (**5n**) (90mg, 0.30 mmol, 1.5 equiv.), 4-bromo-*N,N*-dimethylaniline (64 mg, 0.32 mmol, 1.6 equiv.), *tert*-butyllithium (1.7 M in pentane, 376 µL, 0.640 mmol, 3.20 equiv.) and terephthalonitrile (**4a**) (25.6 mg, 0.200 mmol, 1.00 equiv.). Purification by flash column chromatography (80:20 pentane/diethyl ether) gave the title compound **6na** (39.0 mg, 71%) as a colorless oil.

R*f* = 0.23 (80:20 *n*-hexane/EtOAc, $KMnO_4$ stain)

NMR Spectroscopy (*see spectra*):

^{1}H NMR (400 MHz, $CDCl_3$): δ_H 7.64 (d, *J* = 8.3 Hz, 2H), 7.29 (d, *J* = 8.3 Hz, 2H), 7.23 (d, *J* = 7.7 Hz, 2H), 7.19 – 7.13 (m, 1H), 7.06 – 7.01 (m, 2H), 2.78 (tt, *J* = 9.8, 4.8 Hz, 1H), 2.46 – 2.39 (m, 2H), 2.23 – 2.14 (m, 1H), 2.12 – 1.80 (m, 5H) ppm;

^{13}C NMR (101 MHz, $CDCl_3$): δ_C 148.4, 141.1, 132.9, 128.7, 128.6, 128.3, 126.3, 119.0, 118.7, 111.3, 44.7, 37.8, 33.5, 32.0, 15.5 ppm.

IR (film): ν_{max} 2970, 2938, 2227, 1740, 1607, 1496, 1454, 1366, 1217, 838, 751, 701, 564 cm^{-1}.

HRMS ($APCI^+$): m/z calculated for $C_{19}H_{19}N$ $[M+H]^+$, 275.1543; found, 275.1537.

UOB Confidential & Sensitive

tert-Butyl 3-(4-cyanophenyl)butanoate (6oa)

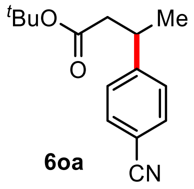

6oa

Prepared following **General Procedure D**, using *tert*-butyl 3-(4,4,5,5-tetramethyl-1,3,2-dioxaborolan-2-yl)butanoate (**5o**) (81 mg, 0.30 mmol, 1.5 equiv.), 4-bromo-*N,N*-dimethylaniline (64 mg, 0.32 mmol, 1.6 equiv.), *tert*-butyllithium (1.7 M in pentane, 376 µL, 0.640 mmol, 3.20 equiv.) and terephthalonitrile (**4a**) (25.6 mg, 0.200 mmol, 1.00 equiv.). Purification by flash column chromatography (87:13 pentane/diethyl ether) gave the title compound **6oa** (26.0 mg, 53%) as a colorless oil.

R_f = 0.51 (80:20 *n*-hexane/EtOAc, $KMnO_4$ stain)

NMR Spectroscopy (*see spectra*):

^{1}H NMR (400 MHz, $CDCl_3$): δ_H 7.58 (d, *J* = 8.3 Hz, 2H), 7.32 (d, *J* = 8.3 Hz, 2H), 3.28 (q, *J* = 7.3 Hz, 1H), 2.50 (d, *J* = 7.6 Hz, 2H), 1.33 (s, 9H), 1.28 (d, *J* = 7.0 Hz, 3H) ppm;

^{13}C NMR (101 MHz, $CDCl_3$): δ_C 171.1, 151.6, 132.4, 127.9, 119.1, 110.3, 80.8, 43.6, 37.0, 28.1, 21.8 ppm.

IR (film): ν_{max} 2970, 2934, 2228, 1728, 1609, 1505, 1455, 1367, 1229, 1217, 1149, 839, 566 cm^{-1}.

All recorded spectroscopic data matched those previously reported in the literature.[40]

4-[(1*R*,2*R*,3*R*,5*R*)-2,6,6-Trimethylbicyclo[3.1.1]heptan-3-yl]benzonitrile (6pa)

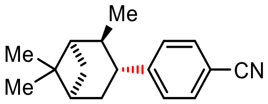

6pa

Prepared following **General Procedure D**, using 4,4,5,5-tetramethyl-2-[(*1R,2S,3R,5R*)-2,6,6-trimethylbicyclo[3.1.1]heptan-3-yl]-1,3,2-dioxaborolane (**5p**) (79 mg, 0.30 mmol, 1.5 equiv.), 4-bromo-*N,N*-dimethylaniline (64 mg, 0.32 mmol, 1.6 equiv.), *tert*-butyllithium (1.7 M in pentane, 376 µL, 0.640 mmol, 3.20 equiv.) and terephthalonitrile (**4a**) (25.6 mg, 0.200 mmol, 1.00 equiv.). Purification by flash column chromatography (95:5 pentane/diethyl ether) gave the title compound **6pa** (41.1 mg, 88%, >20:1 d.r.) as a colorless oil.

R_f = 0.51 (91:9 *n*-hexane/EtOAc, $KMnO_4$ stain)

NMR Spectroscopy (*see spectra*):

^{1}H NMR (400 MHz, $CDCl_3$): δ_H 7.59 (d, *J* = 7.8 Hz, 2H), 7.39 (d, *J* = 8.3 Hz, 2H), 3.05 (dt, *J* = 10.3, 7.8 Hz, 1H), 2.52 (dtd, *J* = 9.9, 6.2, 2.2 Hz, 1H), 2.42 (dddd, *J* = 13.9, 10.3, 3.7, 2.1 Hz, 1H), 2.08 – 1.99 (m, 2H),

1.90 (ddd, *J* = 6.8, 5.4, 1.8 Hz, 1H), 1.84 (ddd, *J* = 13.9, 7.6, 2.3 Hz, 1H), 1.28 (s, 3H), 1.15 (s, 3H), 1.10 (d, *J* = 9.9 Hz, 1H), 0.98 (d, *J* = 7.1 Hz, 3H) ppm;

^{13}C NMR (101 MHz, $CDCl_3$): δ_C 155.1, 132.3, 129.3, 119.3, 109.6, 48.0, 45.8, 45.3, 41.9, 39.3, 37.3, 35.1, 28.6, 23.1, 21.0 ppm.

IR (film): ν_{max} 2970, 2941, 2228, 1740, 1606, 1454, 1372, 1217, 909, 839, 730, 561 cm^{-1}.

HRMS (APCI$^+$): m/z calculated for $C_{17}H_{22}N$ [M+H]$^+$, 240.1747; found, 240.1741.

4-[(1*R,2*R**,4*S**)-Bicyclo[2.2.1]heptan-2-yl]benzonitrile (6qa)**

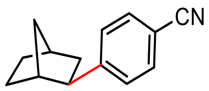

6qa

Prepared following **General Procedure D**, using 2-{(1*R**,2*R**,4*R**)-bicyclo[2.2.1]heptan-2-yl}-4,4,5,5-tetramethyl-1,3,2-dioxaborolane (**5q**) (67 mg, 0.30 mmol, 1.5 equiv.), 4-bromo-*N,N*-dimethylaniline (64 mg, 0.32 mmol, 1.6 equiv.), *tert*-butyllithium (1.7 M in pentane, 376 µL, 0.640 mmol, 3.20 equiv.) and terephthalonitrile (**4a**) (25.6 mg, 0.200 mmol, 1.00 equiv.). Purification by flash column chromatography (95:5 pentane/diethyl ether) gave the title compound **6qa** (26.1 mg, 66%, 4.3:1 d.r.) as a colorless oil.

R_f = 0.49 (91:9 *n*-hexane/EtOAc, $KMnO_4$ stain)

NMR Spectroscopy (*see spectra*):

^{1}H NMR (400 MHz, $CDCl_3$): δ_H 7.62 – 7.53 (m, 2H), 7.30 (d, *J* = 8.1 Hz, 2H), 3.25 (dd, *J* = 10.5, 4.7 Hz, 0.19H), 2.78 (dd, *J* = 9.2, 5.6 Hz, 0.81H), 2.48 – 2.31 (m, 2H), 2.01 (dddd, *J* = 12.6, 11.6, 4.7, 3.2 Hz, 0.2H), 1.81 (ddd, *J* = 11.8, 9.2, 2.4 Hz, 0.8H), 1.69 – 1.53 (m, 3H), 1.51 – 1.11 (m, 4H) ppm;

^{13}C NMR (101 MHz, $CDCl_3$): δ_C (*Major isomer*) 153.3, 132.2, 128.0, 119.3, 109.3, 47.6, 42.7, 39.3, 37.0, 36.3, 30.6, 28.8. (*Minor isomer*) 149.8, 131.9, 129.1, 125.6, 109.4, 46.4, 42.6, 40.7, 37.6, 34.3, 30.1, 23.0 ppm.

IR (film): ν_{max} 2950, 2870, 2226, 1739, 1606, 1504, 1455, 1311, 849, 826, 557 cm^{-1}.

All recorded spectroscopic data matched those previously reported in the literature.[38]

4-{(3*S*,5*R*,6*S*,8*S*,10*R*,13*R*,14*S*,17*R*)-3-Chloro-10,13-dimethyl-17-[(*S*)-6-methylheptan-2-yl]hexadecahydro-1*H*-cyclopenta[a]phenanthren-6-yl}benzonitrile (6ra)

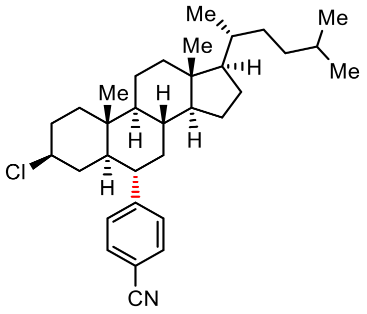

6ra

Prepared following **General Procedure D**, using 2-{(*3S,5R,6S,8R,10S,13R,14S,17R*)-3-chloro-10,13-dimethyl-17-[(*S*)-6-methylheptan-2-yl]hexadecahydro-1*H*-cyclopenta[a]phenanthren-6-yl}-4,4,5,5-tetramethyl-1,3,2-dioxaborolane (**5r**) (160 mg, 0.300 mmol, 1.50 equiv.), 4-bromo-*N,N*-dimethylaniline (64 mg, 0.32 mmol, 1.6 equiv.), *tert*-butyllithium (1.7 M in pentane, 376 µL, 0.640 mmol, 3.20 equiv.) and terephthalonitrile (**4a**) (25.6 mg, 0.200 mmol, 1.00 equiv.). Purification by flash column chromatography (95:5 pentane/diethyl ether) gave the title compound **6ra** (74.2 mg, 73%, 4.6:1 d.r.) as a colorless oil.

$\mathbf{R}_f$ = 0.43 (91:9 *n*-hexane/EtOAc, $KMnO_4$ stain)

NMR Spectroscopy (*see spectra*):

> ^{1}**H NMR** (400 MHz, $CDCl_3$): δ_H 7.67 – 7.43 (m, 2H), 7.29 – 7.07 (m, 2H), 3.97 – 3.81 (m, 0.15H), 3.77 – 3.58 (m, 0.85H), 3.05 – 2.97 (m, 0.15H), 2.66 – 2.44 (m, 0.85H), 2.25 – 2.12 (m, 0.3H), 2.09 -1.93 (m, 1.70H), 1.91 – 1.67 (m, 4H), 1.58 – 1.44 (m, 4H), 1.42 – 0.75 (m, 31H), 0.73 – 0.43 (m, 3H) ppm;

> ^{13}**C NMR** (101 MHz, $CDCl_3$): δ_C (*Major isomer*) 151.2, 132.6, 131.5, 119.1, 110.2, 60.0, 56.4, 56.3, 54.1, 51.0, 45.1, 42.8, 41.1, 40.0, 39.6, 38.9, 36.3, 36.0, 35.9, 35.3, 33.0, 28.3, 28.1, 24.2, 24.0, 22.9, 22.7, 21.3, 18.8, 13.3, 12.2 ppm.

IR (film): ν_{max} 2970, 2227, 1740, 1607, 1508, 1456, 1369, 1264, 1217, 1129, 1043, 909, 837, 804, 731 cm^{-1}.

HRMS ($APCI^+$): m/z calculated for $C_{34}H_{51}NCl$ $[M+H]^+$, 508.3705; found, 508.3706.

4-[5-(2,5-Dimethylphenoxy)-2-methylpentan-2-yl]benzonitrile (6sa)

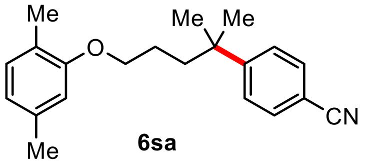

6sa

Prepared following **General Procedure D**, using 2-(5-(2,5-dimethylphenoxy)-2-methylpentan-2-yl)-4,4,5,5-tetramethyl-1,3,2-dioxaborolane (**5s**) (100 mg, 0.300 mmol, 1.50 equiv.), 4-bromo-*N,N*-dimethylaniline (64 mg, 0.32 mmol, 1.6 equiv.), *tert*-butyllithium (1.7 M in pentane, 376 µL, 0.640 mmol, 3.20 equiv.) and

terephthalonitrile (**4a**) (25.6 mg, 0.200 mmol, 1.00 equiv.). Purification by flash column chromatography (95:5 pentane/diethyl ether) gave the title compound **6sa** (52.9 mg, 86%) as a colorless oil.

R*f* = 0.52 (91:9 *n*-hexane/EtOAc, $KMnO_4$ stain)

NMR Spectroscopy (*see spectra*):

^{1}**H NMR** (400 MHz, $CDCl_3$): δ_H 7.60 (d, *J* = 8.5 Hz, 2H), 7.48 (d, *J* = 8.5 Hz, 2H), 7.01 (d, *J* = 7.5 Hz, 1H), 6.67 (d, *J* = 7.5 Hz, 1H), 6.55 (s, 1H), 3.85 (t, *J* = 6.2 Hz, 2H), 2.30 (s, 3H), 2.18 (s, 3H), 1.90 – 1.81 (m, 2H), 1.62 – 1.49 (m, 2H), 1.37 (s, 6H) ppm;

^{13}**C NMR** (101 MHz, $CDCl_3$): δ_C 157.0, 155.0, 136.6, 132.1, 130.4, 126.9, 123.5, 120.9, 119.2, 112.0, 109.6, 67.9, 40.4, 38.3, 28.9, 25.0, 21.5, 15.9 ppm.

IR (film): ν_{max} 2970, 2950, 2227, 1740, 1607, 1508, 1456, 1369, 1264, 1229, 1217, 1129, 1043, 909, 837 cm^{-1}.

HRMS ($APCI^+$): m/z calculated for $C_{21}H_{26}NO$ $[M+H]^+$, 308.2009; found, 308.2005.

4-(*tert*-Butyl)benzonitrile (6ta)

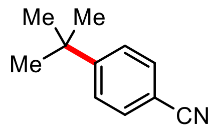

6ta

Prepared following **General Procedure D**, using 2-(*tert*-butyl)-4,4,5,5-tetramethyl-1,3,2-dioxaborolane (**5t**) (55 mg, 0.30 mmol, 1.5 equiv.), 4-bromo-*N,N*-dimethylaniline (64 mg, 0.32 mmol, 1.6 equiv.), *tert*-butyllithium (1.7 M in pentane, 376 µL, 0.640 mmol, 3.20 equiv.) and terephthalonitrile (**4a**) (25.6 mg, 0.200 mmol, 1.00 equiv.). Purification by flash column chromatography (95:5 pentane/diethyl ether) gave the title compound **6ta** (29.7 mg, 90%) as a colorless oil.

R*f* = 0.41 (95:5 *n*-hexane/EtOAc, $KMnO_4$ stain)

NMR Spectroscopy (*see spectra*):

^{1}**H NMR** (400 MHz, $CDCl_3$): δ_H 8.50 (dd, *J* = 4.6, 1.7 Hz, 2H), 7.27 (dd, *J* = 4.6, 1.7 Hz, 2H), 1.31 (s, 9H) ppm;

^{13}**C NMR** (101 MHz, $CDCl_3$): δ_C 156.8, 132.1, 126.3, 119.3, 109.4, 35.4, 31.1 ppm.

IR (film): ν_{max} 2969, 2871, 2227, 1740, 1606, 1505, 1464, 1366, 1229, 1217, 1106, 837, 569 cm^{-1}.

All recorded spectroscopic data matched those previously reported in the literature.[41]

tert-Butyl 3-(4-cyanophenyl)-3-cyclohexylazetidine-1-carboxylate (6ua)

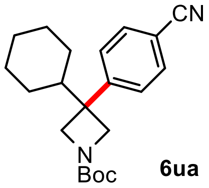

6ua

Prepared following **General Procedure D**, using *tert*-butyl 3-cyclohexyl-3-(4,4,5,5-tetramethyl-1,3,2-dioxaborolan-2-yl)azetidine-1-carboxylate (**5u**) (110 mg, 0.300 mmol, 1.50 equiv.), 4-bromo-*N,N*-dimethylaniline (64 mg, 0.32 mmol, 1.6 equiv.), *tert*-butyllithium (1.7 M in pentane, 376 µL, 0.640 mmol, 3.20 equiv.) and terephthalonitrile (**4a**) (25.6 mg, 0.200 mmol, 1.00 equiv.). Purification by flash column chromatography (89:11 pentane/EtOAc) gave the title compound **6ua** (36.8 mg, 54%) as a colorless oil.

R_f = 0.44 (86:14 *n*-hexane/EtOAc, $KMnO_4$ stain)

NMR Spectroscopy (*see spectra*):

^{1}H NMR (400 MHz, $CDCl_3$): δ_H 7.61 (d, J = 8.4 Hz, 2H), 7.11 (d, J = 8.4 Hz, 2H), 4.10 (s, 4H), 1.87 – 1.56 (m, 6H), 1.43 (s, 9H), 1.31 – 1.11 (m, 2H), 1.01 – 0.83 (m, 1H), 0.70 – 0.55 (m, 2H) ppm;

^{13}C NMR (101 MHz, $CDCl_3$): δ_C 156.3, 149.0, 131.7, 128.4, 118.9, 110.5, 79.9, 58.9, 46.8, 46.4, 28.5, 27.5, 26.4, 26.2 ppm.

IR (film): ν_{max} 2927, 2854, 2228, 1738, 1698, 1451, 1392, 1366, 1217, 1153, 914, 843, 732, 598 cm^{-1}.

HRMS (ESI$^+$): m/z calculated for $C_{21}H_{28}N_2NaO_2$ [M+Na]$^+$, 363.2043; found, 363.2047.

4-[(3*r*,5*r*,7*r*)-Adamantan-1-yl]benzonitrile (6va)

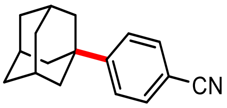

6va

Prepared following **General Procedure D**, using 2-[(3*r*,5*r*,7*r*)-adamantan-1-yl]-4,4,5,5-tetramethyl-1,3,2-dioxaborolane (**5v**) (79 mg, 0.30 mmol, 1.5 equiv.), 4-bromo-*N,N*-dimethylaniline (64 mg, 0.32 mmol, 1.6 equiv.), *tert*-butyllithium (1.7 M in pentane, 376 µL, 0.640 mmol, 3.20 equiv.) and terephthalonitrile (**4a**) (25.6 mg, 0.200 mmol, 1.00 equiv.). Purification by flash column chromatography (95:5 pentane/diethyl ether) gave the title compound **6va** (15.2 mg, 32%) as a colorless oil.

R_f = 0.44 (91:9 *n*-hexane/EtOAc, $KMnO_4$ stain)

NMR Spectroscopy (*see spectra*):

^{1}H NMR (400 MHz, $CDCl_3$): δ_H 7.60 (d, J = 8.6 Hz, 2H), 7.45 (d, J = 8.5 Hz, 2H), 2.16 – 2.07 (m, 3H), 1.90 (d, J = 2.9 Hz, 6H), 1.85 – 1.67 (m, 6H) ppm;

^{13}**C NMR** (101 MHz, $CDCl_3$): δ_C 156.8, 132.2, 126.0, 119.4, 109.4, 42.9, 37.0, 36.7, 28.9 ppm.

IR (film): ν_{max} 2917, 2849, 2234, 1739, 1448, 1366, 1229, 1217, 806, 563, 527 cm^{-1}.

HRMS (APCI$^+$): m/z calculated for $C_{17}H_{20}N$ $[M+H]^+$, 238.1590; found, 238.1591.

4-Cyclohexyl-2,5-dimethylbenzonitrile (6hb)

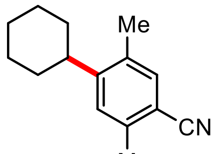

6hb

Prepared following **General Procedure D**, using 2-cyclohexyl-4,4,5,5-tetramethyl-1,3,2-dioxaborolane (**5h**) (63 mg, 0.30 mmol, 1.5 equiv.), 4-bromo-*N,N*-dimethylaniline (64 mg, 0.32 mmol, 1.6 equiv.), *tert*-butyllithium (1.7 M in pentane, 376 µL, 0.640 mmol, 3.20 equiv.) and 2,5-dimethylterephthalonitrile (**4b**) (31.2 mg, 0.200 mmol, 1.00 equiv.). Purification by flash column chromatography (96:4 pentane/diethyl ether) gave the title compound **6hb** (24.3 mg, 57%) as a colorless oil.

R$_f$ = 0.66 (91:9 *n*-hexane/EtOAc, $KMnO_4$ stain)

NMR Spectroscopy (*see spectra*):

^{1}**H NMR** (400 MHz, $CDCl_3$): δ_H 7.34 (s, 1H), 7.12 (s, 1H), 2.76 – 2.63 (m, 1H), 2.48 (s, 3H), 2.29 (s, 3H), 1.91 – 1.84 (m, 2H), 1.82 – 1.71 (m, 3H), 1.48 – 1.21 (m, 5H) ppm;

^{13}**C NMR** (101 MHz, $CDCl_3$): δ_C 151.4, 139.6, 133.9, 133.6, 127.5, 118.8, 109.6, 40.5, 33.4, 27.0, 26.3, 20.3, 18.8 ppm.

IR (film): ν_{max} 2926, 2852, 2220, 1613, 1497, 1448, 1257, 1029, 857, 800 cm^{-1}.

HRMS (APCI$^+$): m/z calculated for $C_{15}H_{20}N$ $[M+Na]^+$, 214.1590; found, 214.1591.

4-Cyclohexylpyridine (6hc)

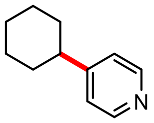

6hc

Prepared following a modified **General Procedure D**, using 2-cyclohexyl-4,4,5,5-tetramethyl-1,3,2-dioxaborolane (**5h**) (63 mg, 0.30 mmol, 1.5 equiv.), 4-bromo-*N,N*-dimethylaniline (64 mg, 0.32 mmol, 1.6 equiv.), *tert*-butyllithium (1.7 M in pentane, 376 µL, 0.640 mmol, 3.20 equiv.), and isonicotinonitrile (**4c**) (20.8 mg, 0.200 mmol, 1.00 equiv.) in DMF (1.0 mL). The reaction mixture was not washed with 2 M HCl solution but transferred to a 50 mL round flask with Et_2O (20 mL) and concentrated under reduced pressure. Purification by flash column

UOB Confidential & Sensitive

chromatography (95:5 pentane/diethyl ether) gave the title compound **6hc** (16.8 mg, 52%) as a colorless oil.

R*f* = 0.49 (91:9 *n*-hexane/EtOAc, $KMnO_4$ stain)

NMR Spectroscopy (*see spectra*):

^{1}H NMR (400 MHz, $CDCl_3$): δ_H 8.48 (d, *J* = 6.2 Hz, 2H), 7.12 (d, *J* = 6.2 Hz, 2H), 2.49 (tq, *J* = 9.0, 3.0 Hz, 1H), 1.86 (dq, *J* = 7.1, 4.7, 4.1 Hz, 4H), 1.76 (dtt, *J* = 12.7, 3.0, 1.6 Hz, 1H), 1.49 – 1.33 (m, 4H), 1.32 – 1.17 (m, 1H) ppm;

^{13}C NMR (101 MHz, $CDCl_3$): δ_C 156.9, 149.7, 122.5, 44.0, 33.6, 26.7, 26.1 ppm.

IR (film): ν_{max} 2925, 2852, 1740, 1597, 1448, 1366, 1229, 1217, 1031, 814, 624, 547 cm^{-1}.

All recorded spectroscopic data matched those previously reported in the literature.[42]

4-[5-(2,5-Dimethylphenoxy)-2-methylpentan-2-yl]-2,5-dimethylbenzonitrile (6sb)

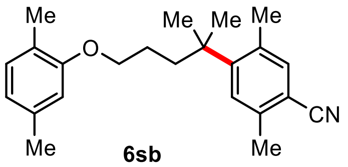

6sb

Prepared following **General Procedure D**, using 2-(5-(2,5-dimethylphenoxy)-2-methylpentan-2-yl)-4,4,5,5-tetramethyl-1,3,2-dioxaborolane (**5s**) (100 mg, 0.300 mmol, 1.50 equiv.), 4-bromo-*N,N*-dimethylaniline (64 mg, 0.32 mmol, 1.6 equiv.), *tert*-butyllithium (1.7 M in pentane, 376 µL, 0.640 mmol, 3.20 equiv.) and 2,5-dimethylterephthalonitrile (**4b**) (31.2 mg, 0.200 mmol, 1.00 equiv.). Purification by flash column chromatography (95:5 pentane/diethyl ether) gave the title compound **6sb** (61.7 mg, 92%) as a white solid.

R*f* = 0.51 (91:9 *n*-hexane/EtOAc, $KMnO_4$ stain)

NMR Spectroscopy (*see spectra*):

^{1}H NMR (400 MHz, $CDCl_3$): δ_H 7.33 (s, 1H), 7.26 (s, 1H), 7.01 (d, *J* = 7.5Hz, 1H), 6.66 (d, *J* = 7.5 Hz, 1H), 6.56 (s, 1H), 3.87 (t, *J* = 6.0 Hz, 2H), 2.53 (s, 3H), 2.50 (s, 3H), 2.30 (s, 3H), 2.17 (s, 3H), 2.04 – 1.95 (m, 2H), 1.57 – 1.45 (m, 2H), 1.44 (s, 6H) ppm;

^{13}C NMR (101 MHz, $CDCl_3$): δ_C 157.0, 151.7, 139.1, 136.6, 136.3, 134.8, 130.5, 129.5, 123.5, 120.8, 118.5, 111.9, 110.3, 68.0, 39.6, 38.1, 29.5, 25.5, 22.7, 21.5, 20.4, 16.0 ppm.

IR (film): ν_{max} 2953, 2870, 2222, 1611, 1585, 1508, 1461, 1388, 1263, 1157, 1129, 1042, 887, 803, 587 cm^{-1}.

HRMS (ESI$^+$): m/z calculated for $C_{23}H_{30}NO$ $[M+H]^+$, 336.2322; found, 336.2330.

4-[5-(2,5-Dimethylphenoxy)-2-methylpentan-2-yl]pyridine (6sc)

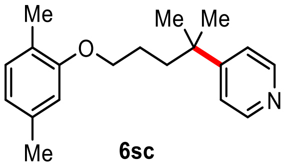

6sc

Prepared following a modified **General Procedure D**, using 2-(5-(2,5-dimethylphenoxy)-2-methylpentan-2-yl)-4,4,5,5-tetramethyl-1,3,2-dioxaborolane (**5s**) (100 mg, 0.300 mmol, 1.50 equiv.), 4-bromo-*N,N*-dimethylaniline (64 mg, 0.32 mmol, 1.6 equiv.), *tert*-butyllithium (1.7 M in pentane, 376 µL, 0.640 mmol, 3.20 equiv.) and isonicotinonitrile (**4c**) (20.8 mg, 0.200 mmol, 1.00 equiv.). The reaction mixture was not washed with 2 M HCl solution but transferred to a 50 mL round flask with Et_2O (20 mL) and concentrated under reduced pressure. Purification by flash column chromatography (80:20 pentane/EtOAc) gave the title compound **6sc** (49.9 mg, 88%) as a colorless oil.

R_f = 0.24 (80:20 *n*-hexane/EtOAc, $KMnO_4$ stain)

NMR Spectroscopy (*see spectra*):

^{1}H NMR (400 MHz, $CDCl_3$): δ_H 8.53 (d, *J* = 6.4 Hz, 2H), 7.29 – 7.26 (m, 2H), 7.00 (d, *J* = 7.4 Hz, 1H), 6.65 (d, *J* = 7.5 Hz, 1H), 6.55 (s, 1H), 3.85 (t, *J* = 6.2 Hz, 2H), 2.29 (d, *J* = 0.8 Hz, 3H), 2.17 (s, 3H), 1.88 – 1.79 (m, 2H), 1.61 – 1.50 (m, 2H), 1.34 (s, 6H) ppm;

^{13}C NMR (101 MHz, $CDCl_3$): 158.6, 157.0, 149.7, 136.6, 130.4, 123.6, 121.5, 120.9, 112.0, 68.0, 40.1, 37.8, 28.4, 25.0, 21.5, 15.9 ppm.

IR (film): ν_{max} 2956, 2868, 1596, 1509, 1459, 1411, 1265, 1158, 1130, 1043, 996, 821, 584 cm^{-1}.

HRMS (ESI$^+$): m/z calculated for $C_{19}H_{26}NO$ $[M+H]^+$, 284.2009; found, 284.2013.

2.6. Mechanistic Studies

2.6.1. Regioselectivity of Boronate Complex Formation

The formation of primary borinic ester **9** upon activation of **1a** with **A** indicates that boronate complex formation occurs regioselectively at the sterically less hindered primary boronic ester. This means that the high secondary selectivity observed in the photoinduced coupling reactions with (hetero)aryl nitriles must arise from a 1,2-boron shift of the secondary boronic ester to the initially generated primary alkyl radical.

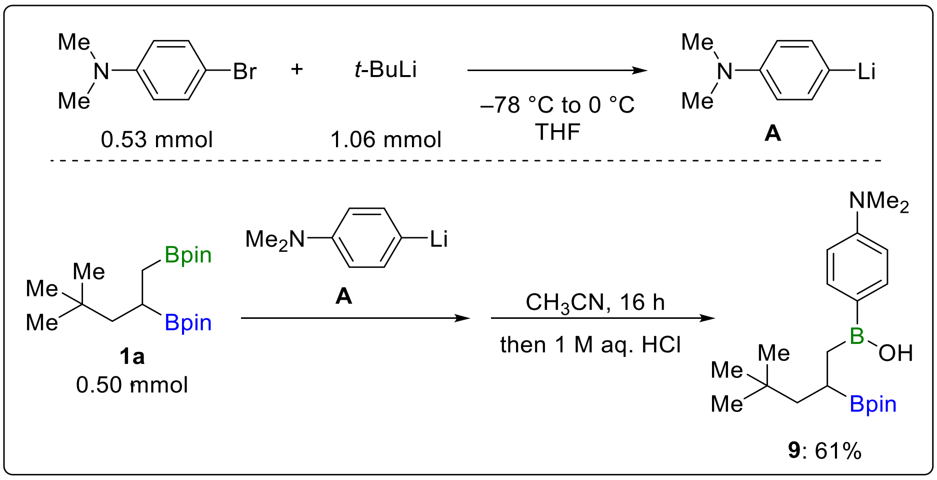

Scheme S5. Synthesis of Primary Borinic Acid 9.

A flame-dried nitrogen-flushed 10 mL Schlenk tube was charged with 4-bromo-*N,N*-dimethylaniline (106 mg, 0.530 mmol, 1.06 equiv.) and THF (1.5 mL). The solution was cooled to –78 °C (dry ice/acetone) then *t*-BuLi (624 μL, 1.06 mmol, 1.7 M in pentane) was added slowly dropwise and the mixture was stirred for 5 min before removing the cooling bath, then the solution was allowed to stir at 0 °C for 30 min. In glovebox, to a 7 mL vial equipped with a magnetic stir bar was added 2,2'-(4,4-dimethylpentane-1,2-diyl)bis(4,4,5,5-tetramethyl-1,3,2-dioxaborolane) (**1a**) (176 mg, 0.500 mmol, 1.00 equiv.). The vial was sealed with a septum and removed from the glovebox, and then anhydrous diethyl ether (3.5 mL) was added under N_2. The solution was cooled to −78 °C (dry ice/acetone) and the pre-prepared [4-(dimethylamino)phenyl]lithium solution was added dropwise. The mixture was allowed to stir for 30 min at −78 °C before removing the cooling bath and warming to ambient temperature. After 30 min, the solvent was removed under high vacuum, then anhydrous CH_3CN (2.0 mL) was added to the vial, and the solution was allowed to stir for 16 hours at room temperature. After that, the reaction mixture was added a solution of 2.0 M aqueous HCl (1.5 mL) and allowed to stir for 5 min. Then the reaction mixture was extracted with Et_2O (3 × 5 mL). The organic layers were combined, dried over Na_2SO_4, and concentrated under reduced pressure. The resulting crude material was purified by flash column chromatography (91:9 pentane/diethyl ether) to give the title compound **9** (113.8 mg, 61%) as a white solid.

UOB Confidential & Sensitive

4-{[4,4-Dimethyl-2-(4,4,5,5-tetramethyl-1,3,2-dioxaborolan-2-yl)pentyl](hydroxy)boraneyl}-*N,N*-dimethylaniline (9)

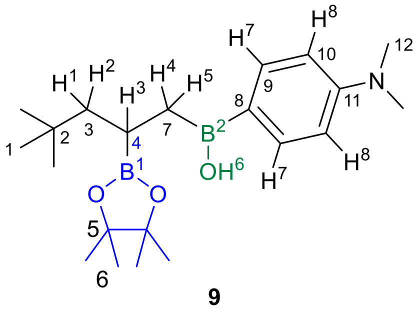

9

R*f* = 0.29 (91:9 *n*-hexane/EtOAc, CAM stain)

NMR Spectroscopy (*see spectra*):

^{1}H NMR (400 MHz, $CDCl_3$): δ_H 7.77 (d, *J* = 8.7 Hz, 2H^7), 7.44 (s, 1H^6), 6.71 (d, *J* = 8.7 Hz, 2H^8), 3.00 (s, 6H, NMe_2), 1.76 (dd, *J* = 13.4, 10.8 Hz, 1H^1), 1.61 (dd, *J* = 17.0, 9.5 Hz, 1H^4), 1.39 (dd, *J* = 17.0, 4.1 Hz, 1H^5), 1.32 – 1.21 (m, 13H, Bpin + H^3), 1.12 (dd, *J* = 13.4, 2.8 Hz, 1H^2), 0.88 (s, 9H, *t*-Bu) ppm;

^{13}C NMR (101 MHz, $CDCl_3$): δ_C 152.6 (C^{11}), 135.5 (C^9), 111.2 (C^{10}), 83.7 (C^5), 48.8 (C^{12}), 40.3 (C^3), 31.3 (C^2), 29.8 (C^1), 24.8 (C^6) ppm. The carbon (C^4, C^7, C^8) attached to boron was not observed due to quadrupolar relaxation;

^{11}B NMR (128 MHz, $CDCl_3$): δ_B 48.7 (B^2), 35.2 (B^2) ppm.

Note: The *NOESY* spectra shows that H^7 has *NOE* with H^4 and H^5, but has no *NOE* with H^1 and H^2, confirming the formation of borinic acid regioisomer at primary position**.**

IR (film): ν_{max} 3478, 2951, 1740, 1604, 1368, 1313, 1217, 1204, 1139, 884, 798, 523 cm^{-1}.

HRMS (ESI$^+$): m/z calculated for $C_{21}H_{38}B_2NO_3$ [M+H]$^+$, 374.3032; found, 374.3030.

2.6.2. UV/Vis Absorption Spectroscopy

Synthesis of Boronate Complex 2a:

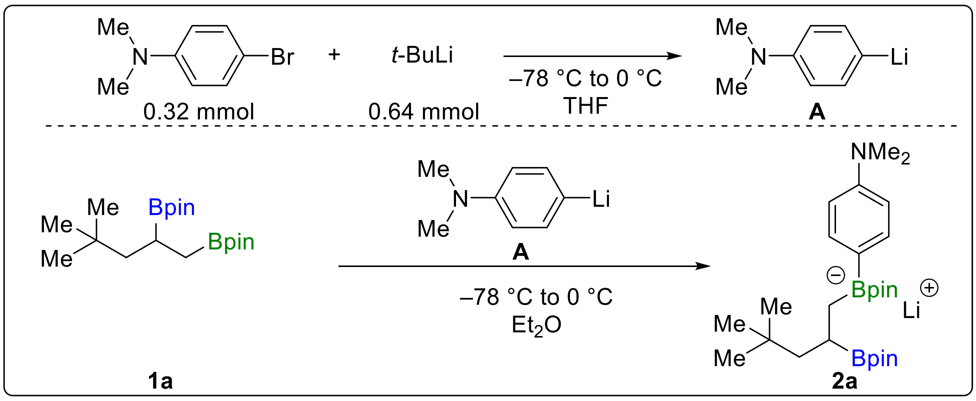

Scheme S6. Synthesis of Boronate Complex 2a.

A flame-dried nitrogen-flushed 10 mL Schlenk tube was charged with 4-bromo-*N,N*-dimethylaniline (64.2 mg, 0.321 mmol, 1.07 equiv.) and THF (0.8 mL). The solution was cooled to –78 °C (dry ice/acetone) then *t*-BuLi (378 μL, 0.642 mmol, 2.14 equiv., 1.7 M in pentane) was added slowly dropwise and the mixture was stirred for 5 min before removing the cooling bath, then the solution was allowed to stir at 0 °C for 30 min (**Figure S2**). In a glovebox, to a 7 mL vial equipped with a magnetic stir bar was added 2,2'-(4,4-dimethylpentane-1,2-diyl)bis(4,4,5,5-tetramethyl-1,3,2-dioxaborolane) (**1a**) (105 mg, 0.300 mmol, 1.00 equiv.). The vial was sealed with a septum and removed from the glovebox, and then anhydrous diethyl ether (2.0 mL) was added under N_2. The solution was cooled to −78 °C (dry ice/acetone) and the pre-prepared [4-(dimethylamino)phenyl]lithium solution was added dropwise. The mixture was allowed to stir for 30 min at −78 °C before removing the cooling bath and warming to ambient temperature. After 30 min, the solvent was removed under high vacuum, and boronate complex **5** was obtained as a white solid and quickly put into the glovebox for further use (**Scheme S6**).

The reaction of boronate complex **2a** and terephthalonitrile (**4a**) was investigated by UV/Vis spectroscopy to determine whether EDA complex formation occurred under the reaction conditions. These studies were performed using DMF as the solvent due to the poor solubility of **4a** in CH_3CN. The absorption spectra of DMF solutions of **2a** (0.05 M), **4a** (0.05 M), and a 1:1 mixture of **2a** (0.05 M) and **4a** (0.05 M) [spectrum recorded 10 min after mixing] are shown in **Figure S3(a)**. The absorption spectrum of the 1:1 mixture shows a clear bathochromic shift, which provides evidence for the formation of an EDA complex between boronate complex **2a** and terephthalonitrile (**4a**). **Figure S3(b)** shows the absorption spectra at higher concentration (0.15 M in DMF), which further enhanced the bathochromic shift.

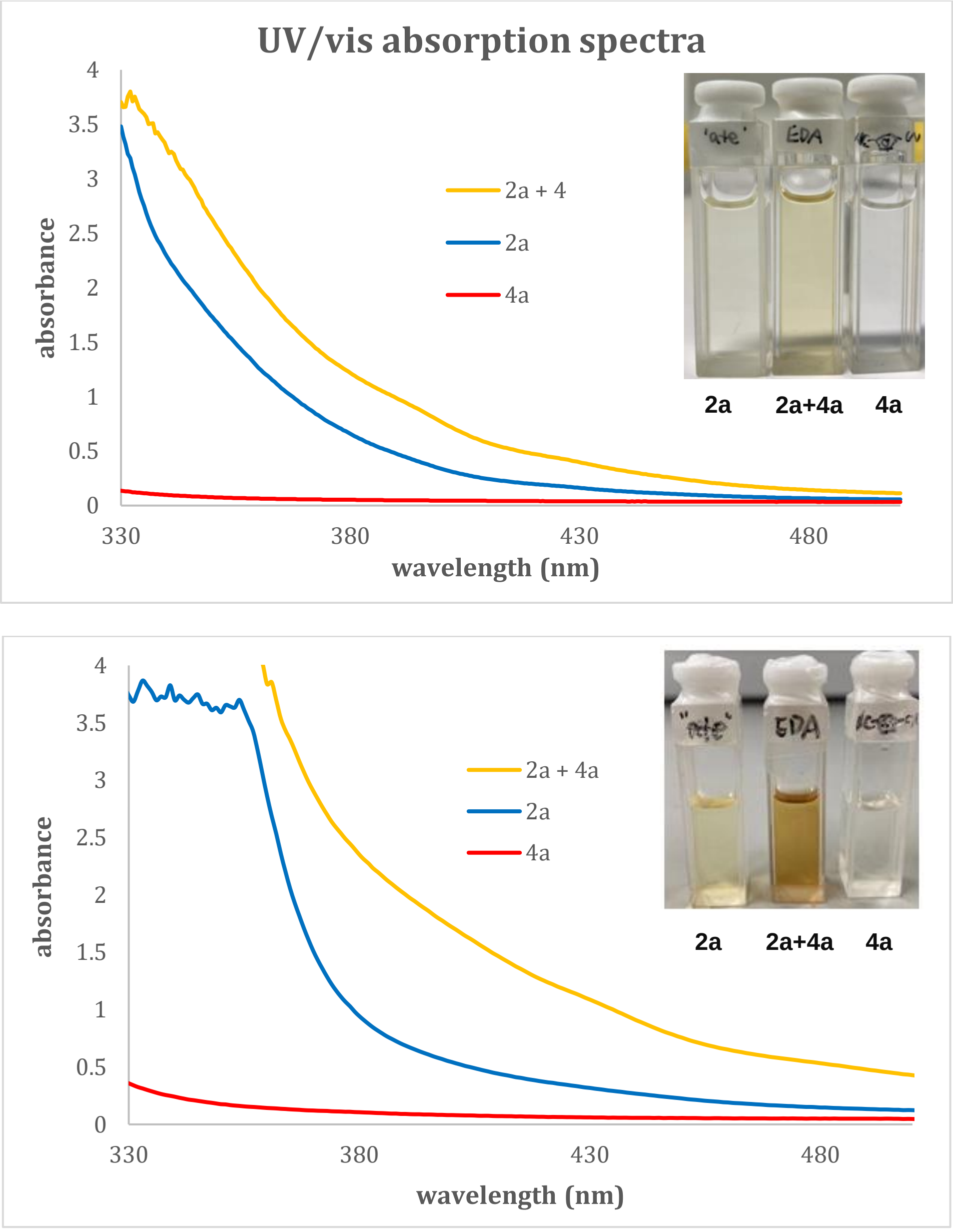

Figure S3. UV/Vis Absorption Spectra of 2a and 4a at (a) 0.05 M and (b) 0.15 M.

UOB Confidential & Sensitive

Synthesis of Boronate Complex 2h:

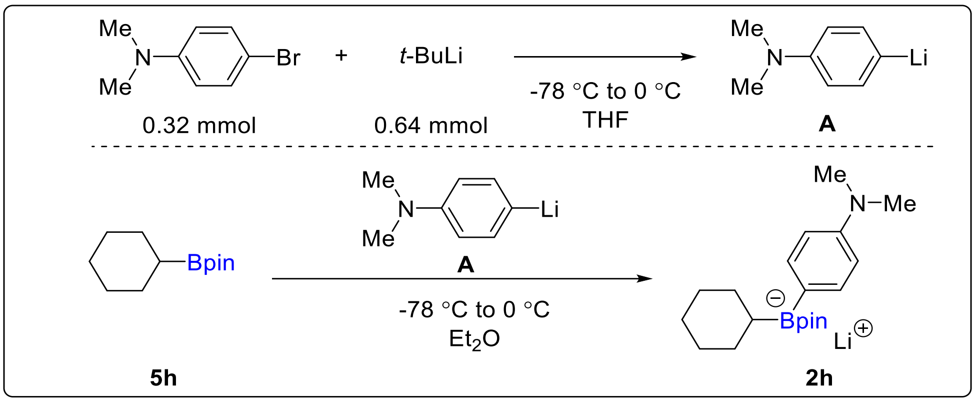

Scheme S7. Synthesis of Boronate Complex 2h.

A flame-dried nitrogen-flushed 10 mL Schlenk tube was charged with 4-bromo-*N,N*-dimethylaniline (64.2 mg, 0.321 mmol, 1.07 equiv.) and THF (0.8 mL). The solution was cooled to –78 °C (dry ice/acetone) then *t*-BuLi (378 µL, 0.642 mmol, 2.14 equiv., 1.7 M in pentane) was added slowly dropwise and the mixture was stirred for 5 min before removing the cooling bath, then the solution was allowed to stir at 0 °C for 30 min (**Figure S2**). In a glovebox, to a 7 mL vial equipped with a magnetic stir bar was added 2-cyclohexyl-4,4,5,5-tetramethyl-1,3,2-dioxaborolane (**5h**) (63.0 mg, 0.300 mmol, 1.00 equiv.). The vial was sealed with a septum and removed from the glovebox, and then anhydrous diethyl ether (2.0 mL) was added under N_2. The solution was cooled to −78 °C (dry ice/acetone) and the pre-prepared [4-(dimethylamino)phenyl]lithium solution was added dropwise. The mixture was allowed to stir for 30 min at −78 °C before removing the cooling bath and warming to ambient temperature. After 30 min, the solvent was removed under high vacuum, and boronate complex **2h** was obtained as a white solid and quickly put into the glovebox for further use (**Scheme S7**).

The reaction of boronate complex **2h** and terephthalonitrile (**4a**) was investigated by UV/Vis spectroscopy to determine whether EDA complex formation occurred under the reaction conditions. These studies were performed using DMF as the solvent due to the poor solubility of **4a** in CH_3CN. The absorption spectra of DMF solutions of **2h** (0.05 M), **4a** (0.05 M), and a 1:1 mixture of **2h** (0.05 M) and **4a** (0.05 M) [spectrum recorded 10 min after mixing] are shown in **Figure S4**. The absorption spectrum of the 1:1 mixture shows a clear bathochromic shift, which provides evidence for the formation of an EDA complex between boronate complex **2h** and terephthalonitrile (**4a**).

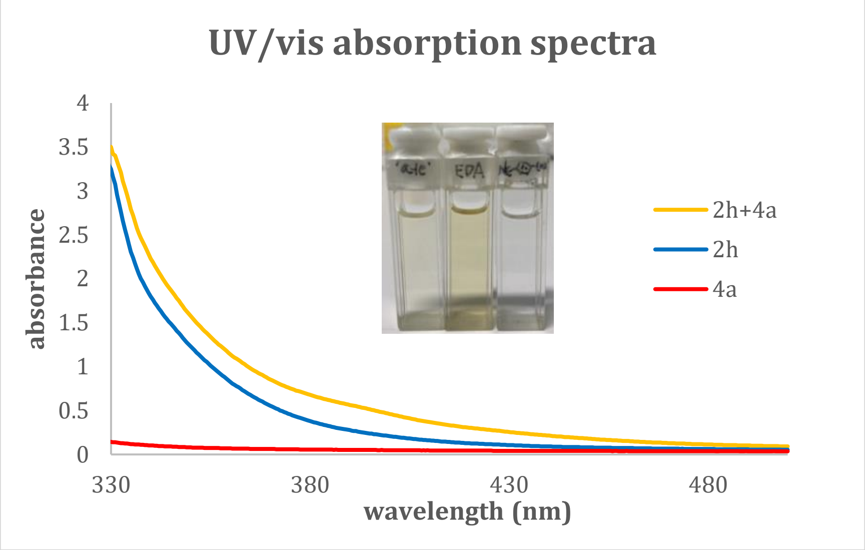

Figure S4. UV/Vis Absorption Spectra of 2h and 4a.

Synthesis of Boronate Complex 8:

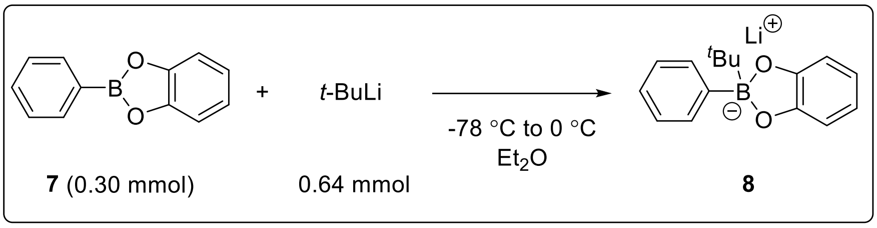

Scheme S8. Synthesis of Boronate Complex 8.

In a glovebox, to a 7 mL vial equipped with a magnetic stir bar was added 2-phenylbenzo[d][1,3,2]dioxaborole (**7**) (58.8 mg, 0.30 mmol, 1.0 equiv.). The vial was sealed with a septum and removed from the glovebox, and then anhydrous diethyl ether (2.0 mL) was added under N_2. The solution was cooled to −78 °C (dry ice/acetone) and *t*-BuLi (188 μL, 0.32 mmol, 1.7 M in pentane) was added slowly dropwise. The mixture was allowed to stir for 30 min at −78 °C before removing the cooling bath and warming to ambient temperature. After 30 min, the solvent was removed under high vacuum, and boronate complex **2h** was obtained as a white solid and quickly put into the glovebox for further use (**Scheme S8**).

The reaction of boronate complex **8** and terephthalonitrile (**4a**) was investigated by UV/Vis spectroscopy to determine whether EDA complex formation occurred under the reaction conditions. The absorption spectra of DMA solutions of **8** (0.15 M), **4a** (0.15 M), and a 1:1 mixture of **8** (0.15 M) and **4a** (0.15 M) [spectrum recorded 10 min after mixing] are shown in **Figure S5**. The absorption spectrum of the 1:1 mixture shows a clear bathochromic shift, which provides evidence for the formation of an EDA complex between boronate complex **8** and terephthalonitrile (**4a**).

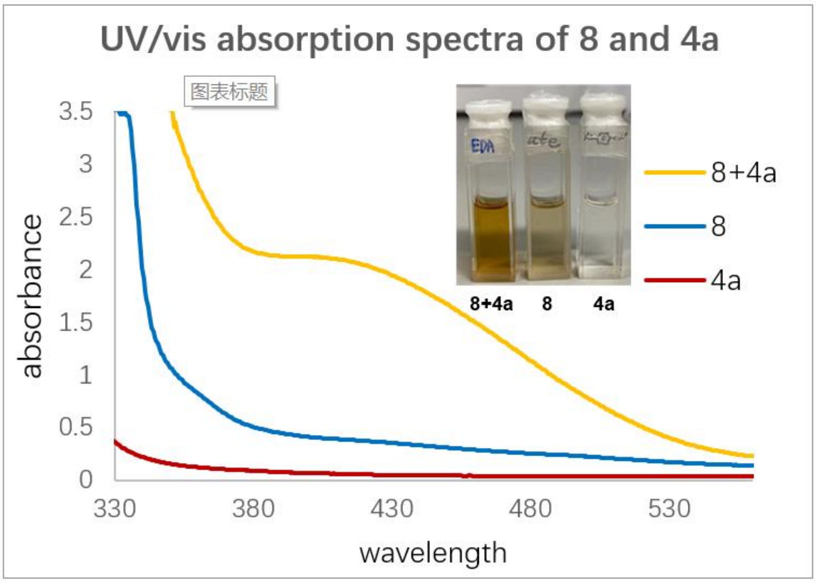

Figure S5. UV/Vis Absorption Spectra of 8 and 4a.

UOB Confidential & Sensitive

2.6.3. Quantum Yield Measurement

Determination of the Photon Flux: The photon flux of the LED setup was determined using standard ferrioxalate actinometry[43] following a modified literature procedure:[44]

A 0.018 M ferrioxalate solution was prepared by dissolving 178 mg of potassium ferrioxalate trihydrate and 84 µL of H_2SO_4 (95–98%) in 20 mL of water. This solution was stored in an amber bottle in the dark. A buffer solution was prepared by dissolving 2.5 g of sodium acetate and 0.50 mL of H_2SO_4 (95–98%) in 50 mL of water.

While being careful to minimize exposure to background light, 1.0 mL of the 0.018 M ferrioxalate solution was added to a 7 mL vial. The vial was put into the PhotoCube (λ = 457 nm) and irradiated for between 6 s and 66 s. Immediately after irradiation, 100 µL of the solution was transferred to a foil-covered 10 mL volumetric flask containing 15 mg of 1,10-phenanthroline dissolved in 3.0 mL of the buffer solution. Water was then added to the flask to make a total volume of 10 mL. The flask was shaken to ensure efficient mixing and the solution was stored in the dark for approximately 20 min. 2.0 mL of the solution was transferred to a quartz cuvette (1.0 mL path length) and the absorbance at λ = 510 nm was measured by UV/Vis spectroscopy (**Figure S6**). This process was repeated for 6 s, 6+15 s, 6+30 s, 6+45 s, 6+60 s, and the absorbance of a non-irradiated sample was also measured.

NOTE: After switching on the LEDs in the PhotoCube, the irradiation intensity gradually increases to a maximum after approximately 6 s. To account for this, only measurements taken after this initial 6 s were used to determine the photon flux.

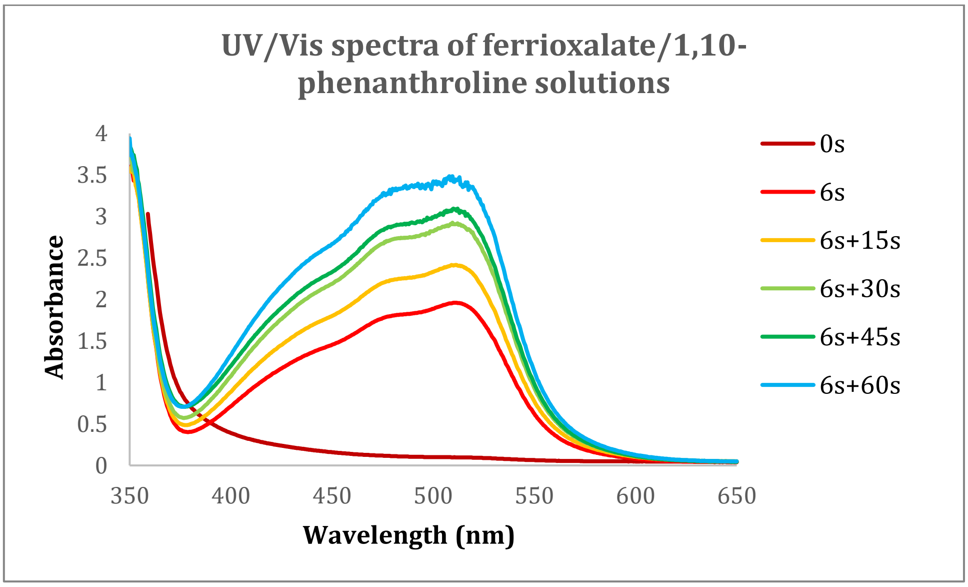

Figure S6: Actinometry: UV/Vis Spectra of Ferrioxalate/1,10-Phenanthroline Solutions.

t (s)	0	6	6+15	6+30	6+45	6+60
Absorbance(A)	0.098	1.960	2.408	2.906	3.093	3.417
ΔA	--	0	0.448	0.946	1.133	1.457
mol Fe^{2+}	--	0	4.04×10^{-6}	8.52×10^{-6}	1.02×10^{-5}	1.31×10^{-5}

The number of moles of Fe^{2+} formed was calculated using:

$$mol\ Fe^{2+} = \frac{V_1 V_3 \Delta A(510\ \mathrm{nm})}{V_2 l \varepsilon(510\ \mathrm{nm})}$$

Where V_1 is the volume of ferrioxalate solution irradiated (1.0×10^{-3} L), V_2 is the volume of the aliquot taken for measurement of the concentration of Fe^{2+} ions (1.0×10^{-4} L), V_3 is the final volume after complexation with 1,10-phenanthroline (1.0×10^{-2} L), ΔA(510 nm) is the difference in absorbance at λ = 510 nm between the irradiated (6+15 s to 6+60 s) and non-irradiated (after 6 s) ferrioxalate/1,10-phenanthroline solutions, l is the optical path length of the irradiation cell (1.0 cm), and ε (510 nm) is the molar absorptivity of the $Fe(phen)_3^{2+}$ complex at λ = 510 nm (11,100 L mol^{-1} cm^{-1}).

The moles of Fe^{2+} were plotted as a function of time (**Figure S7**):

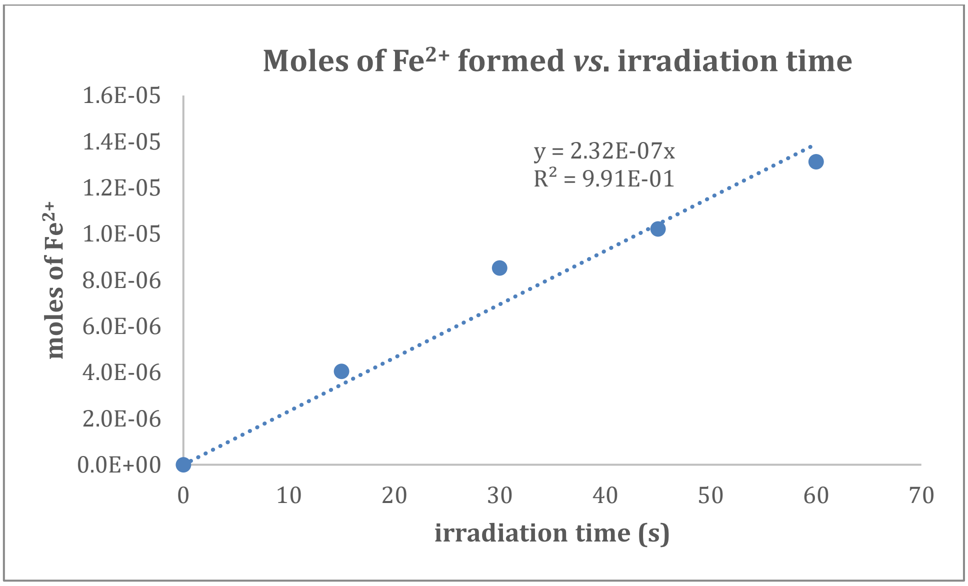

Figure S7: Actinometry: Moles of Fe^{2+} Formed vs. Irradiation Time.

The photon flux was then calculated using:

$$photon\ flux = \frac{mol\ Fe^{2+}}{\Phi t f}$$

Where Φ is the quantum yield of the ferrioxalate actinometer (1.10 at λ = 460 nm),[43] t is the time, and f is the fraction of absorbed light at λ = 457 nm, where $f = 1\text{–}10^{-A}$. The absorbance (A) of the 0.018 M ferrioxalate solution at λ = 457 nm was measured in a quartz cuvette (path length 1.0 cm) by UV/Vis spectroscopy to be 0.398, therefore f = 0.60.

$$photon\ flux = \frac{2.32 \times 10^{-7}}{1.01 \times 0.60} = 3.52 \times 10^{-7}\ \ \mathrm{einstein\ s^{-1}}$$

Determination of the Quantum Yield:

The quantum yield was measured for the coupling reaction of 1,2-bis-boronic ester **1a** with **4a**:

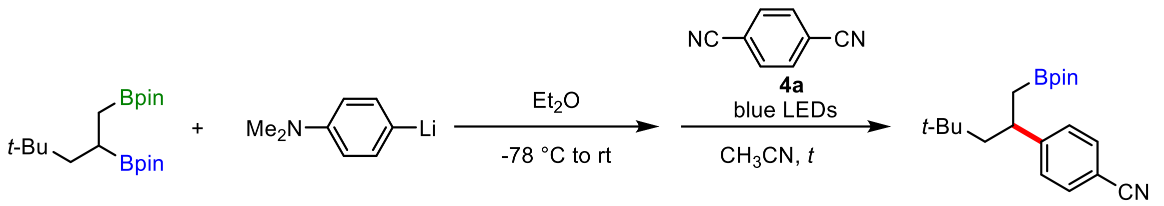

1a **A** **3aa**

A flame-dried nitrogen-flushed 10 mL Schlenk tube was charged with 4-bromo-*N,N*-dimethylaniline (64 mg, 0.32 mmol, 1.6 equiv.) and THF (0.8 mL). The solution was cooled to –78 °C (dry ice/acetone) then *t*-BuLi (376 μL, 0.64 mmol, 3.2 equiv., 1.7 M in pentane) was added slowly dropwise and the mixture was stirred for 5 min before removing the cooling bath, then the solution was allowed to stir at 0 °C for 30 min. In a glovebox, to a 7 mL vial equipped with a magnetic stir bar was added 2,2'-(4,4-dimethylpentane-1,2-diyl)bis(4,4,5,5-tetramethyl-1,3,2-dioxaborolane) (**1a**) (105 mg, 0.300 mmol, 1.00 equiv.). The vial was sealed with a septum and removed from the glovebox, and then anhydrous diethyl ether (2.0 mL) was added under N_2. The solution was cooled to −78 °C (dry ice/acetone) and the pre-prepared [4-(dimethylamino)phenyl]lithium solution was added dropwise. The mixture was allowed to stir for 30 min at −78 °C before removing the cooling bath and warming to ambient temperature. After 30 min, the solvent was removed under high vacuum, and the vial with boronate complex was quickly put into the glovebox, and then terephthalonitrile (**4a**) (26.0 mg, 0.20 mmol, 1.0 equiv.) and anhydrous CH_3CN (1.0 mL) were added. The vial was tightly sealed, then removed from the glovebox and stirred under blue LED irradiation for 30 min. After irradiation, the reaction mixture was diluted with Et_2O (5.0 mL) and the yield of **3aa** was determined to be 4% (0.8 × 10^{-5} mmol) by GC-Fid analysis by using 1,3,5-trimethoxylbenzene as an internal standard.

The reaction was repeated with irradiation times of 1 h, 2 h and 3 h. The yield of **3aa** was found to have a linear relationship with irradiation time (**Figure S8**).

Time	*t* (s)	Yield (%)	Moles of product
-	0	0	0
30 min	1800	4	0.80 × 10^{-5}
1 h	3600	10	2.0 × 10^{-5}
2 h	7200	21	4.2 × 10^{-5}
3 h	10800	37	7.4 × 10^{-5}

UOB Confidential & Sensitive

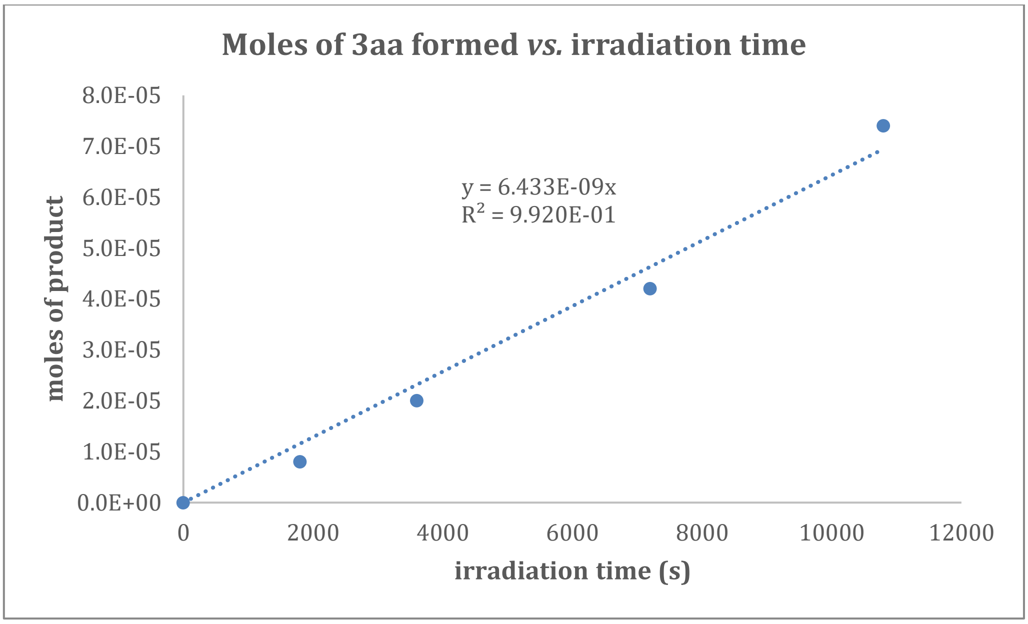

Figure S8: Moles of 3aa Formed vs. Irradiation Time.

The quantum yield (*Φ*) was then calculated using:

$$\Phi = \frac{mol\ product}{photon\ flux\ \times\ t \times f}$$

Where *t* is the time and *f* is the fraction of light absorbed by the reaction mixture at *λ* = 457 nm, where *f* = 1 – 10^{-A} (the absorbance of the reaction mixture (A) at *λ* = 457 nm was measured in a quartz cuvette (path length 1 cm) by UV/Vis spectroscopy be 0.219, thus *f* = 0.396):

$$\Phi = \frac{6.43 \times 10^{-9}}{3.52 \times 10^{-7} \times 0.396} = \ 0.046$$

UOB Confidential & Sensitive

2.6.4. EDA Complex-Mediated Reactions of Catechol Boronic Esters

Synthesis of 2-phenylbenzo[*d*][1,3,2]dioxaborole (7):

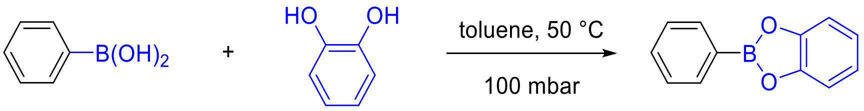

1.0 equiv. 1.0 equiv. **7**

Scheme S9. Synthesis of PhenylBcat 7.

2-Phenylbenzo[*d*][1,3,2]dioxaborole (**7**) was prepared following a literature procedure:[45] To a flame-dried flask were added phenyl boronic acid (1.22 g, 10.0 mmol, 1.00 equiv.) and catechol (1.10 g, 10.0 mmol, 1.00 equiv.) in toluene (50 mL) and the resulting mixture was stirred at room temperature for 1 hour. Then the reaction flask was placed on a rotary evaporator and the solvent was removed with the heating bath set to 50 °C. The resulting solid was dissolved in 100 mL of pentane, filtered, and stored at –20 °C for recrystallization to give the title compound (1.04 g, 53%) as white crystals.

2-Phenylbenzo[*d*][1,3,2]dioxaborole (7)

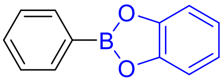

7

R*f* = 0.34 (91:9 *n*-hexane/EtOAc, CAM stain)

NMR Spectroscopy (*see spectra*):

¹H NMR (400 MHz, $CDCl_3$): δ_H 8.14 – 8.06 (m, 2H), 7.62 – 7.55 (m, 1H), 7.53 – 7.47 (m, 2H), 7.33 (dd, *J* = 5.9, 3.3 Hz, 2H), 7.14 (dd, *J* = 5.9, 3.3 Hz, 2H) ppm;

¹³C NMR (101 MHz, $CDCl_3$): δ_C 148.7, 135.1, 132.5, 128.4, 122.9, 112.7 ppm. The carbon attached to boron was not observed due to quadrupolar relaxation.

¹¹B NMR (128 MHz, $CDCl_3$): δ_B 30.7 ppm.

IR (film): ν_{max} 2972, 1740, 1603, 1351, 1315, 1271, 1198, 1138, 1089, 960, 858, 654 cm^{-1}.

All recorded spectroscopic data matched those previously reported in the literature.[45]

UOB Confidential & Sensitive

Photoindued Coupling Reaction with 2-Phenylbenzo[*d*][1,3,2]dioxaborole

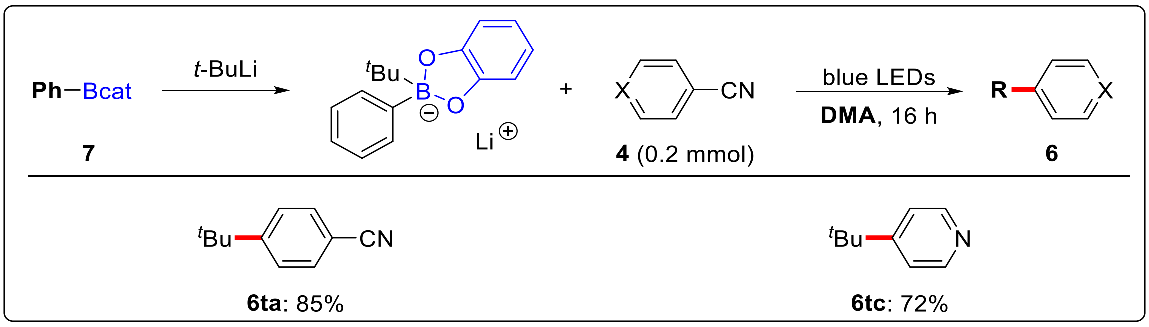

Scheme S10. Photoinduced Coupling Reaction with PhenylBcat.

4-(*tert*-Butyl)benzonitrile (6ta)

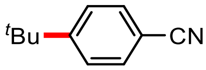

6ta

Prepared following **General Procedure E**, using 2-phenylbenzo[*d*][1,3,2]dioxaborole (**9**) (59 mg, 0.30 mmol, 1.5 equiv.), *tert*-butyllithium (1.7 M in pentane, 176 µL, 0.300 mmol, 1.50 equiv.), and terephthalonitrile (**4a**) (25.6 mg, 0.200 mmol, 1.00 equiv.) in DMA (1.0 mL) without 4CzIPN photocatalyst. Purification by flash column chromatography (95:5 pentane/diethyl ether) gave the title compound **6ua** (27.1 mg, 85%) as a colorless oil. All data (*see above*).

4-(*tert*-Butyl)pyridine (6tc)

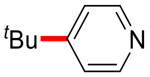

6tc

Prepared following **General Procedure E**, using 2-phenylbenzo[*d*][1,3,2]dioxaborole (**9**) (59 mg, 0.30 mmol, 1.5 equiv.), *tert*-butyllithium (1.7 M in pentane, 176 µL, 0.300 mmol, 1.50 equiv.), and isonicotinonitrile (**4c**) (20.8 mg, 0.200 mmol, 1.00 equiv.) in DMA (1.0 mL) without 4CzIPN photocatalyst. Purification by flash column chromatography (85:15 pentane/diethyl ether) gave the title compound **6tc** (19.5 mg, 72%) as a colorless oil.

R_f = 0.30 (86:14 *n*-hexane/EtOAc, $KMnO_4$ stain)

NMR Spectroscopy (*see spectra*):

^{1}H NMR (400 MHz, $CDCl_3$): δ_H 8.49 (dd, J = 4.3, 2.0 Hz, 2H), 7.25 (dd, J = 4.2, 1.9 Hz, 2H), 1.30 (s, 9H) ppm;

^{13}C NMR (101 MHz, $CDCl_3$): δ_C 159.9, 149.7, 120.8, 34.7, 30.6 ppm.

IR (film): ν_{max} 2964, 2872, 1597, 1547, 1495, 1409, 1366, 1274, 1074, 996, 821, 713, 569 cm^{-1}.

All recorded spectroscopic data matched those previously reported in the literature.[46]

UOB Confidential & Sensitive

2.6.5. Proposed Mechanism for the Photoredox-Catalysed Reaction

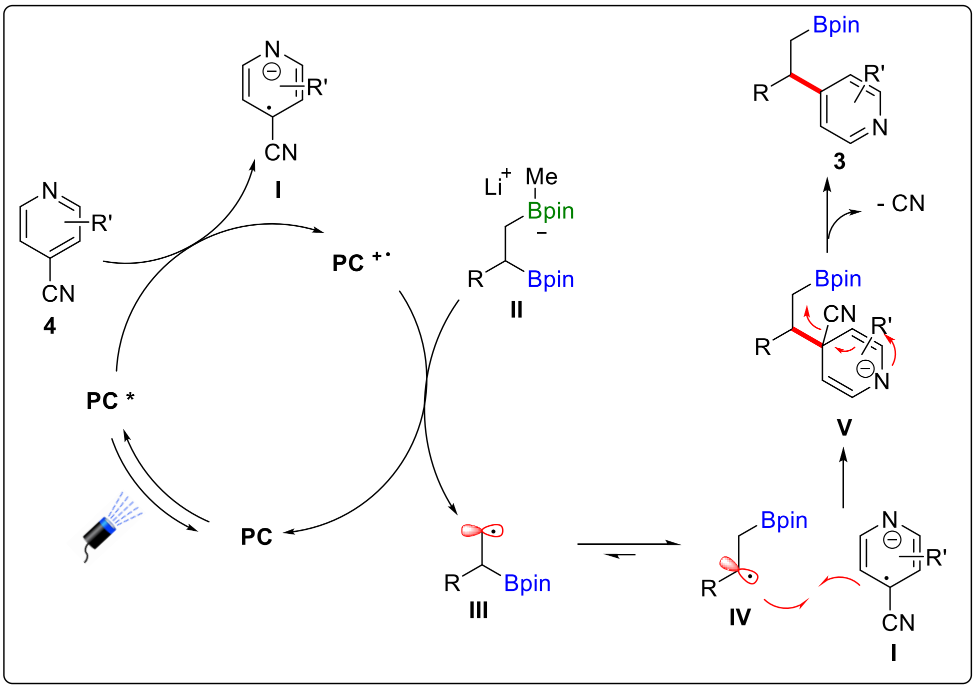

Scheme S11. Proposed Mechanism for the Photoredox-Catalysed Reaction.

Under visible light irradiation, excitation of the photocatalyst (PC) generates the excited state photocatalyst (PC*). Subsequently, PC* can be oxidatively quenched by 4-cyanopyridine **4** to form pyridine radical anion **I** and the radical cation of the photocatalyst ($PC^{+\bullet}$). Single electron transfer between boronate complex **II** and $PC^{+\bullet}$ completes the photoredox catalytic cycle and generates the primary *β*-boryl radical intermediate **III**, which could rapidly undergo 1,2-boron shift to form thermodynamically favoured secondary *β*-boryl radical **IV**. Then radical-radical coupling between the transient radical **IV** and the persistent pyridyl radical anion **I** would form dihydropyridine anion **V**, and subsequent elimination of cyanide gives the coupled product **3**.

2.6.6. Further Support for Photoexcitation of EDA Complexes

To provide further support for the key role of EDA complexes in promoting photoinduced deboronative alkyl radical formation, we investigated the reaction of boronate complex **2t** in Giese reactions with various electron-deficient alkenes. When a mixture of **2t** and benzyl acrylate was irradiated, no Giese product **S1** was formed, whereas when terephthalonitrile (**4a**) was added, **S1** was observed in 1% along with 24% arylation product **6ta** (**Scheme S12**). When the reactions were performed with dimethyl fumarate, 14% Giese product **S2** was formed in the absence of **4a**, whereas 30% Giese product **S2** and 17% arylation product **6ta** were formed in the presence of **4a**. These results provide clear evidence that the addition of terephthalonitrile (**4a**) enhances photoinduced deboronative alkyl radical generation, therefore supporting the key role of EDA complexes in our reactions.

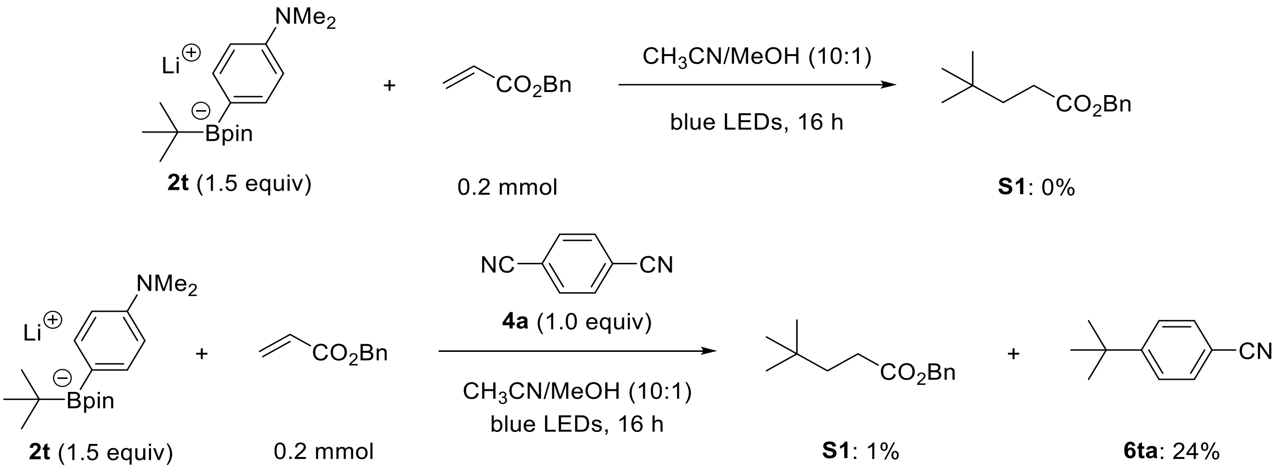

Scheme S12. Attempted Photoinduced Giese Reactions with Benzyl Acrylate.
Yields were determined by GC-FID analysis using 1,3,5-trimethoxybenzene as an internal standard.

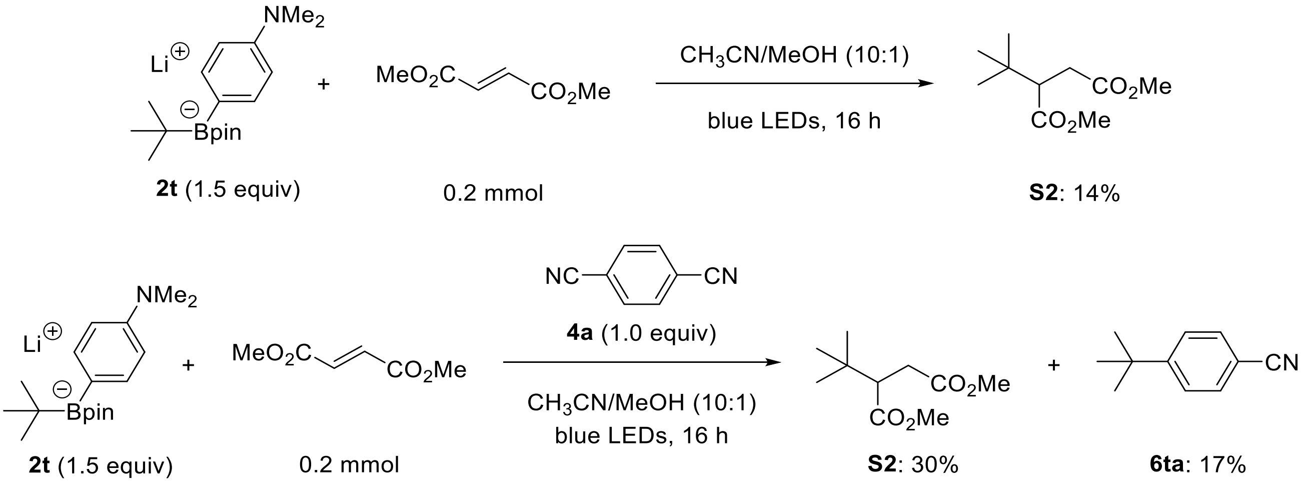

Scheme S13. Photoinduced Giese Reactions with Dimethyl Fumarate.
Yields were determined by GC-FID analysis using 1,3,5-trimethoxybenzene as an internal standard.

UOB Confidential & Sensitive

2.7. Structure Elucidation of 3ua

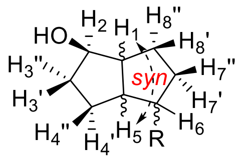

R = 4-CN-C_6H_4
4 possible configurations

NOESY Experiment and Distance Restraints Extraction

A 2D-NOESY experiment for the sample in $CDCl_3$ was run on a 500 MHz Bruker AVANCE III HD Spectrometer with a 5 mm DCH ^{13}C-^{1}H/D Cryoprobe. (Number of scans: 4, mixing time: 0.5 s, spectral size: 2048 (f2) & 512 (f1), spectral width: 10 ppm)

All NOE intensities (η) were integrated in f2 slices and normalised to the corresponding diagonal peaks for quantitative distance measurements, according to the PANIC approach described by Macura, Hu and Butts.[47-49] The geminal Interproton distance between rigid 8' and 8'' from DFT computations (described later) was used as the reference (r_{ref} = 1.76 Å) to afford other NOE distance restraints, given by the equation:

$$\frac{\eta_{ref}}{\eta_i} = \frac{(r_{ref})^{-6}}{(r_i)^{-6}}$$

Where η_{ref} is normalised intensity of NOE for 8' – 8'', of which the ratio to any other normalised NOE intensity (η_i) that is extractable from the 2D spectrum is equal to the ratio of their interproton distances under r^{-6} scaling.

General Procedure for Computational Modelling

The computational modelling started with conformational search using Monte Carlo Multiple Minimum (MCMM) method based on Optimized Potentials for Liquid Simulations (OPLS) all-atom force field and Truncated Newton Gradients (TNCG) minimisation with 500 iterations (0.05 convergence threshold). GB/SA continuum solvation model of chloroform was applied in the search. All different conformers (maximum atom deviation > 0.5 Å) within 21 kJ/mol threshold over the global minima were stored. Around 20-30 conformers were generated for each configuration. This was performed on MacroModel module in Maestro software package powered by Schrodinger. The running environment is the Grendel high performance computing (HPC) cluster in the School of Chemistry, University of Bristol.

For each configuration, all conformers from the MCMM search were subjected to batch DFT optimisation with B3LYP(D3BJ) functional and 6-311+G** level of basis set along with IEFPCM solvation model of chloroform. Tight convergence criteria with ultrafine integration grid were applied in the minimisation. Thermal corrected Gibbs free energy for each conformer was obtained after frequency analysis. These calculations were performed using GAUSSIAN 16 program on Unix system of the BluePebble HPC in the Advanced Computing Research Centre (ACRC), University of Bristol.

All different conformers (maximum atom deviation > 0.1 Å) within 10 kJ/mol above the global minimum after DFT optimization were adopted as computational conformational ensembles in chloroform, which contain at

UOB Confidential & Sensitive

least 5 minimized geometries for each configuration. The calculated NOE distances were extracted from the ensembles under Boltzmann population average. The r^{-6} scaling was used for further comparison with experimental NOE restraints. This was given by:

$$\mathrm{r}_{H-H,calc} = \left(\sum_{i=1}^{n} \left(r_{H-H,i} \right)^{-6} * p_i \right)^{-1/6}$$

Where $\mathrm{r}_{H-H,calc}$ is the calculated NOE distance from the conformational ensembles, in which the interproton distance of each conformer, $r_{H-H,i}$, was scaled by r^{-6} and population in ensembles (p_i) successively. The sum of calculated NOEs was also used for comparison if two cross-peaks are integrated together in the spectrum (due to NOE overlap). Further details are provided in the literature.[50],[51]

Proton Assignment (Pure Shift in Aliphatic Region)

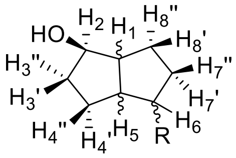

R = 4-CN-C_6H_4

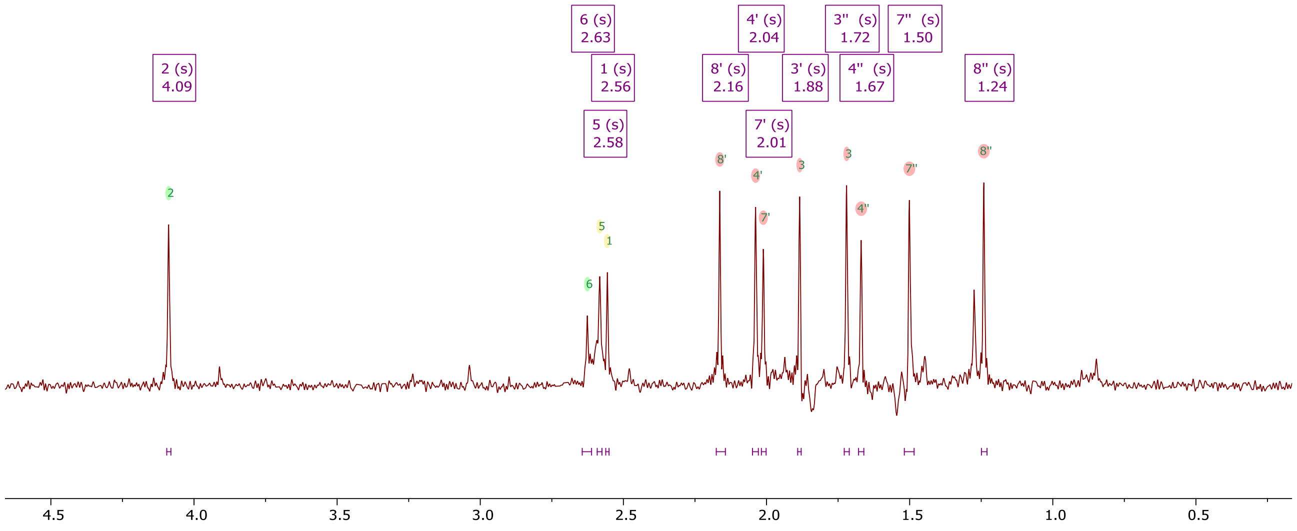

Configuration A

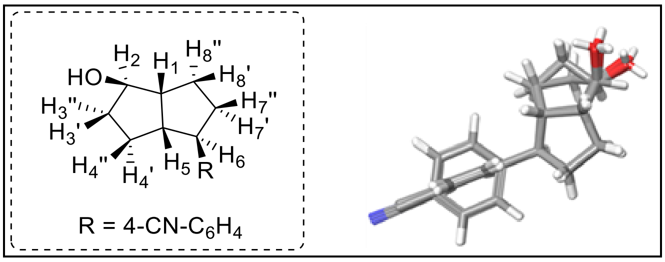

MCMM-DFT conformational ensembles [B3LYP(D3)/6-311+G**/IEFPCM: $CHCl_3$]

NOE restraints	DFT cal. (Å)	NMR exp. (Å)	Violation to exp.
8 '- 8" (ref)	1.76	1.76	
2 - 1/5 sum	2.73	2.64	3.71%
2 - 4'/7' sum	3.49	2.92	19.59%
2 - 3'	2.71	2.46	9.95%
2 - 8"	2.42	2.45	1.39%
8' - 2	3.06	3.00	2.09%
8' - 1/5 sum	2.37	2.52	5.85%
7" - 6	3.05	3.10	1.64%
7" - 1/5 sum	2.62	2.75	4.71%
8" - 4'/7' sum	2.44	2.31	5.65%
8" - 3"	2.65	2.50	6.28%
**MAE**			**6.08%**

Table S3: Comparison Between Calculated NOE Distances and Experiment for Configuration A.

Configuration B

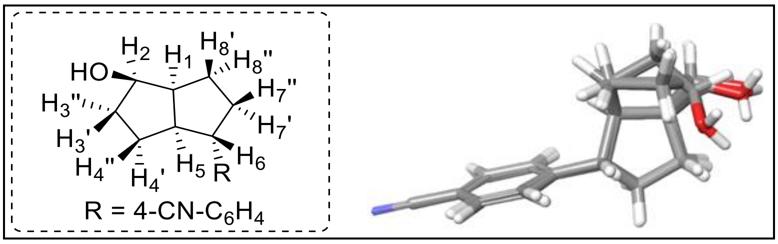

MCMM-DFT conformational ensembles [B3LYP(D3)/6-311+G**/IEFPCM: $CHCl_3$]

NOE restraints	DFT cal. (Å)	NMR exp. (Å)	Violation to exp.
8 '- 8" (ref)	1.76	1.76	
2 - 1/5 sum	2.32	2.64	12.09%
2 - 4'/7' sum	2.82	2.92	3.56%
2 - 3'	2.87	2.46	16.24%
2 - 8"	3.76	2.45	53.28%
8' - 2	3.44	3.00	14.57%
8' - 1/5 sum	2.92	2.52	16.01%
7" - 6	2.45	3.10	21.10%
7" - 1/5 sum	3.54	2.75	28.84%
8" - 4'/7' sum	2.45	2.31	6.23%
8" - 3"	5.02	2.50	101.07%
**MAE**			**27.30%**

Table S4: Comparison Between Calculated NOE Distances and Experiment for Configuration B.

Configuration C

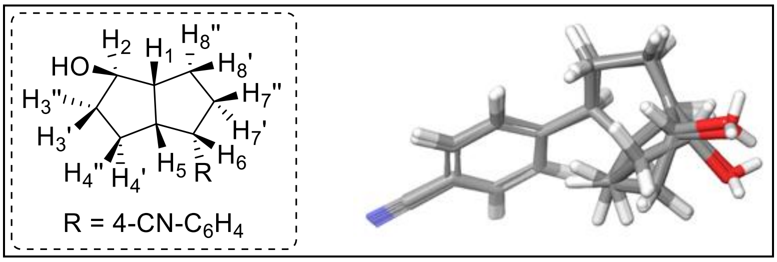

MCMM-DFT conformational ensembles [B3LYP(D3)/6-311+G**/IEFPCM: $CHCl_3$]

NOE restraints	DFT cal. (Å)	NMR exp. (Å)	Violation to exp.
8 '- 8" (ref)	1.76	1.76	
2 - 1/5 sum	2.84	2.64	7.69%
2 - 4'/7' sum	2.41	2.92	17.36%
2 - 3'	2.94	2.46	19.28%
2 - 8"	2.43	2.45	1.01%
8' - 2	3.61	3.00	20.42%
8' - 1/5 sum	2.33	2.52	7.60%
7" - 6	2.46	3.10	20.67%
7" - 1/5 sum	3.73	2.75	35.78%
8" - 4'/7' sum	2.44	2.31	5.70%
8" - 3"	3.52	2.50	41.05%
**MAE**			**17.66%**

Table S5: Comparison Between Calculated NOE Distances and Experiment for Configuration C.

Configuration D

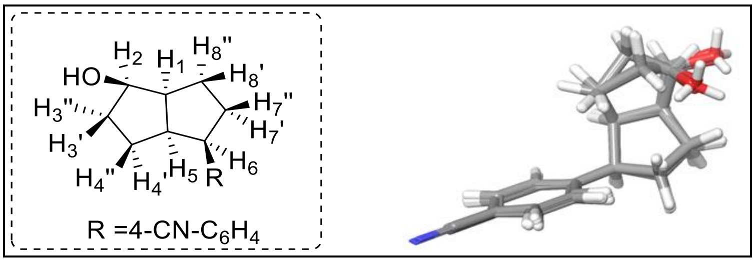

MCMM-DFT conformational ensembles [B3LYP(D3)/6-311+G**/IEFPCM: $CHCl_3$]

NOE restraints	DFT cal. (Å)	NMR exp. (Å)	Violation to exp.
8 '- 8" (ref)	1.76	1.76	
2 - 1/5 sum	2.31	2.64	12.48%
2 - 4'/7' sum	3.23	2.92	10.63%
2 - 3'	2.76	2.46	11.97%
2 - 8"	3.99	2.45	62.47%
8' - 2	3.26	3.00	8.66%
8' - 1/5 sum	2.77	2.52	10.09%
7" - 6	3.05	3.10	1.64%
7" - 1/5 sum	3.43	2.75	24.61%
8" - 4'/7' sum	2.44	2.31	5.66%
8" - 3"	5.02	2.50	100.90%
**MAE**			**24.91%**

Table S6: Comparison Between Calculated NOE Distances and Experiment for Configuration D.

3. SPECTROSCOPIC DATA

^{1}H NMR (400 MHz, $CDCl_3$) of **1a** (*see procedure*)

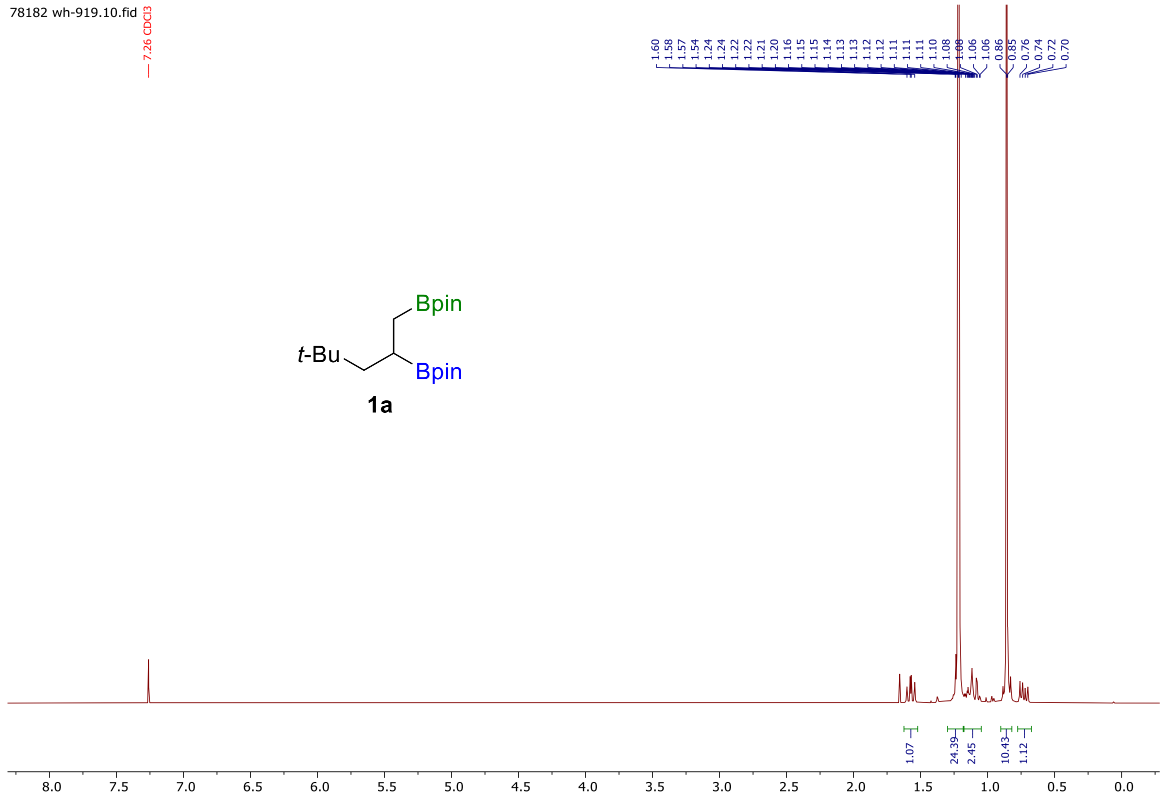

^{13}C NMR (101 MHz, $CDCl_3$) of **1a**

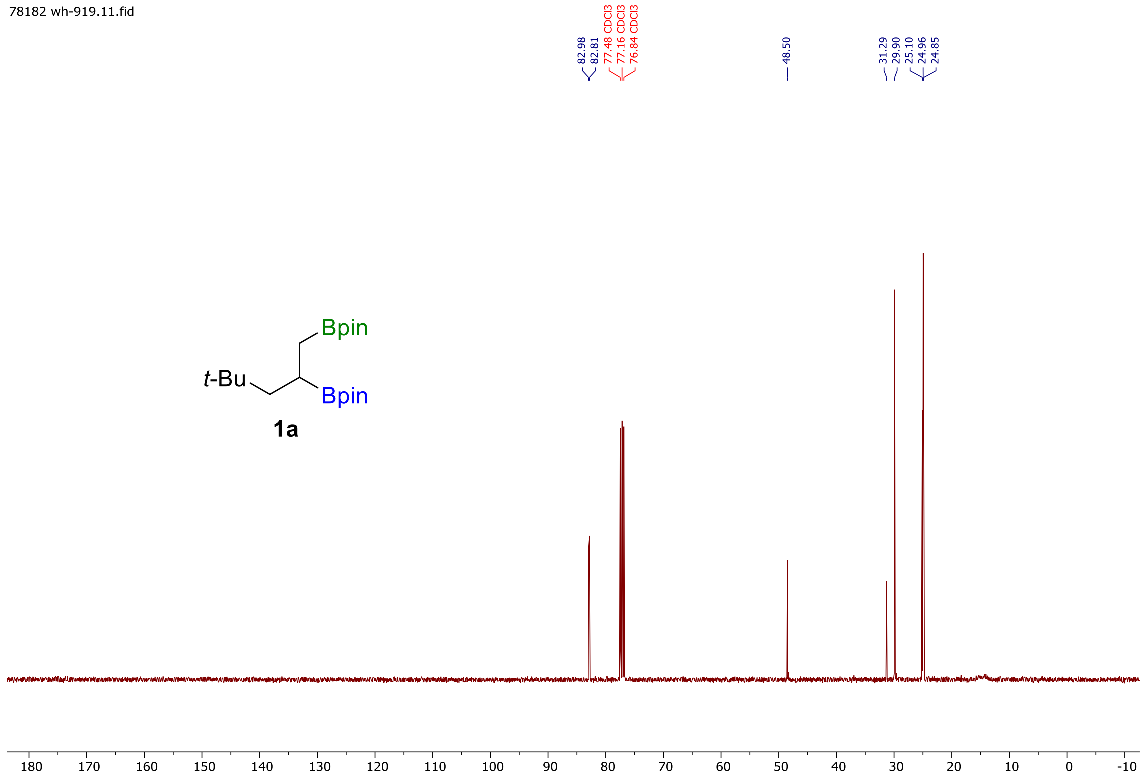

^{11}B NMR (128 MHz, $CDCl_3$) of **1a**

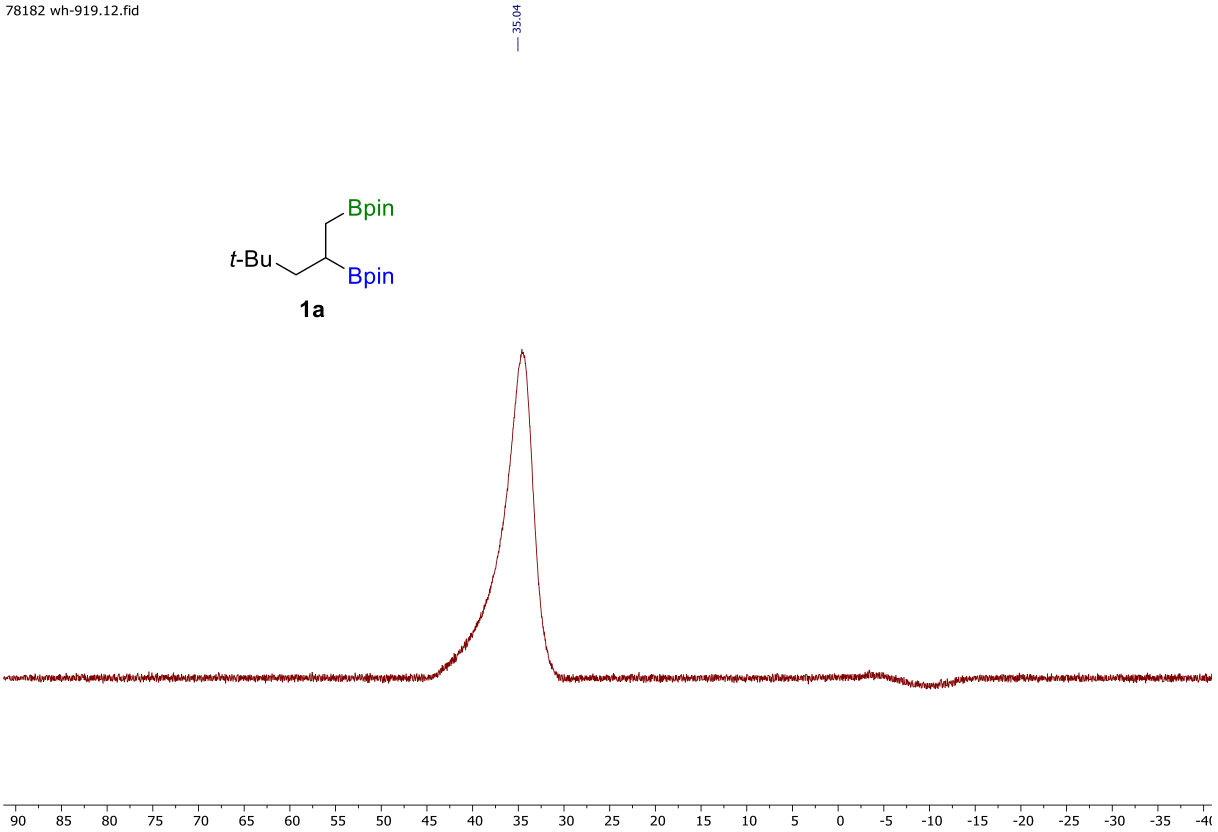

^{1}H NMR (400 MHz, $CDCl_3$) of **1b** (see procedure)

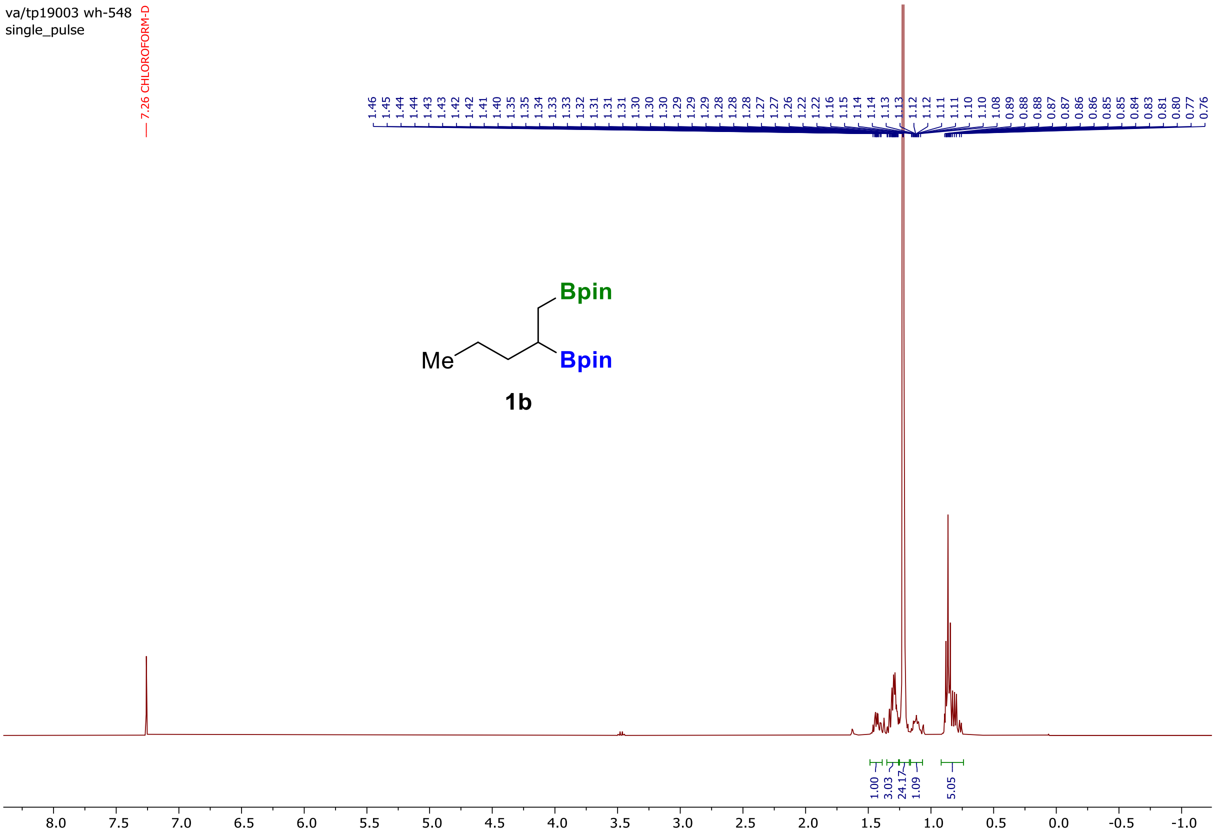

^{13}C NMR (101 MHz, $CDCl_3$) of **1b**

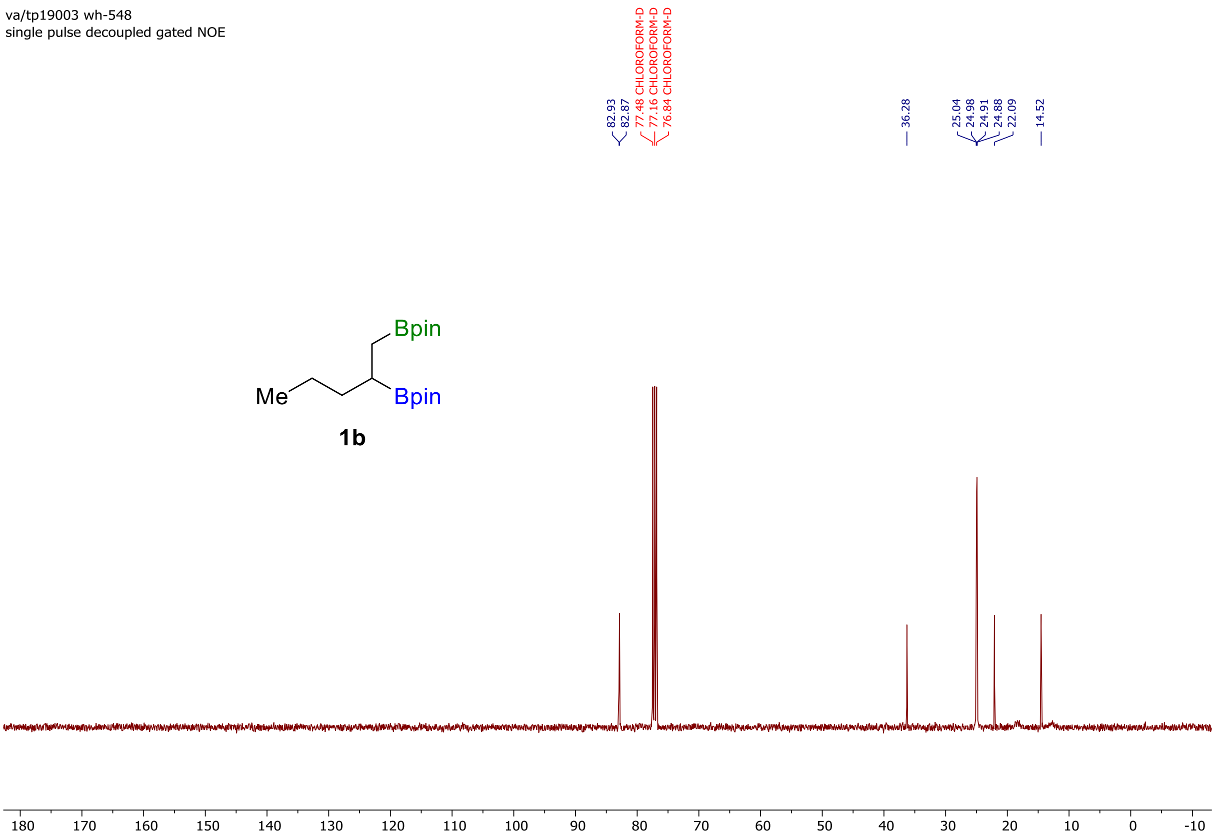

^{11}B NMR (128 MHz, $CDCl_3$) of **1b**

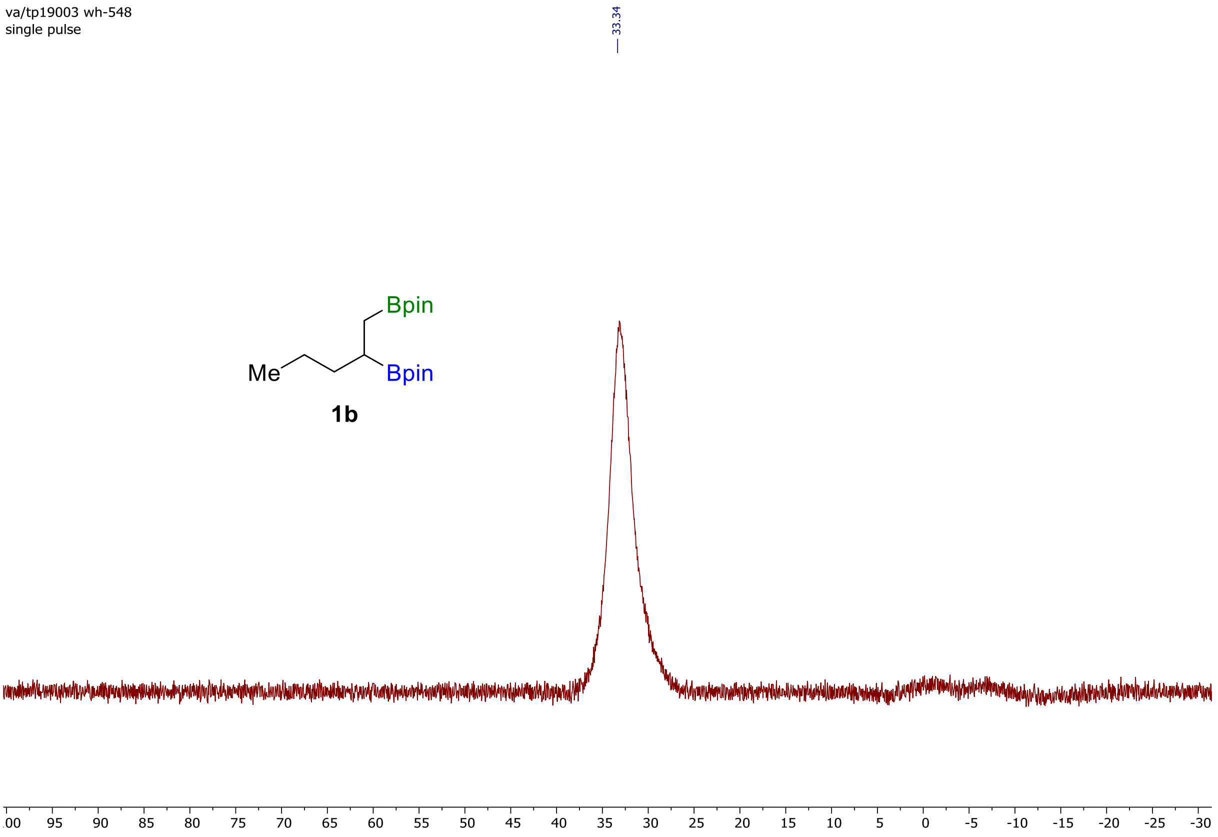

^{1}H NMR (400 MHz, $CDCl_3$) of **1c** (*see procedure*)

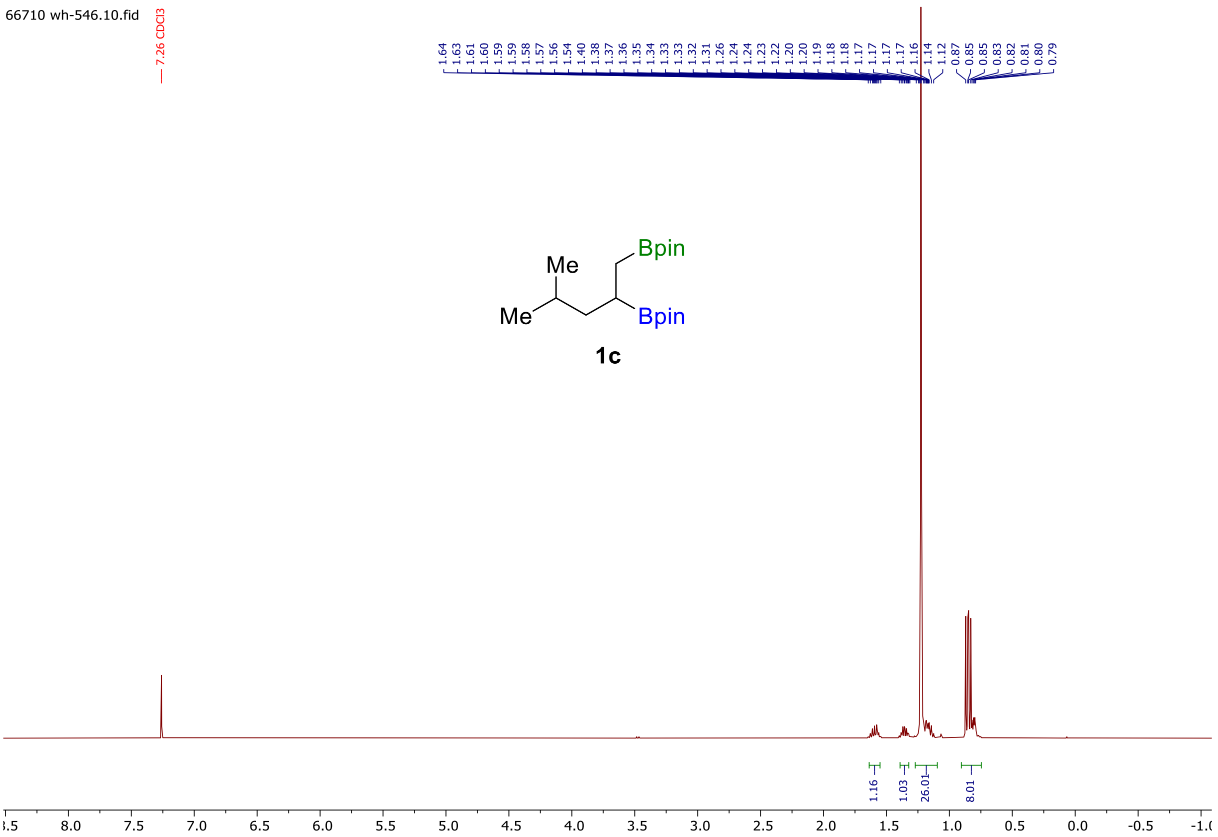

^{13}C NMR (101 MHz, $CDCl_3$) of **1c**

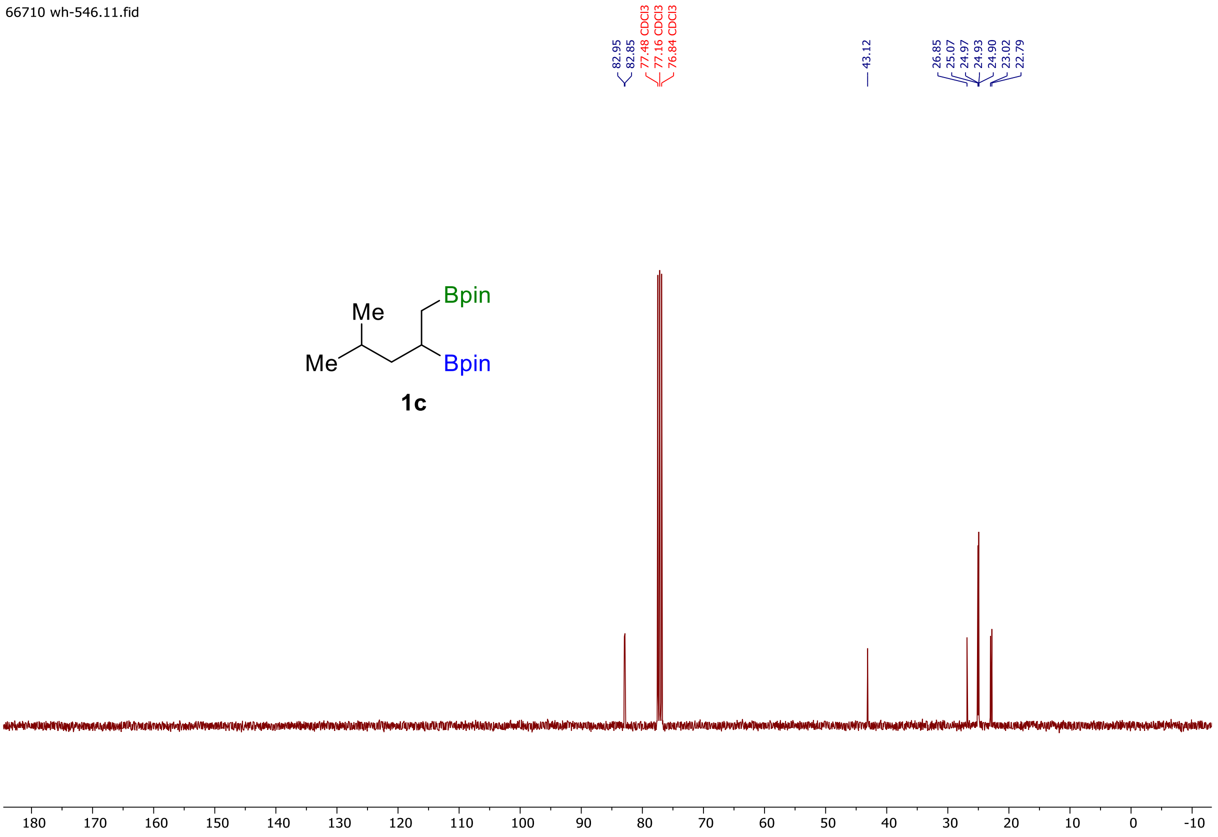

^{11}B NMR (128 MHz, $CDCl_3$) of **1c**

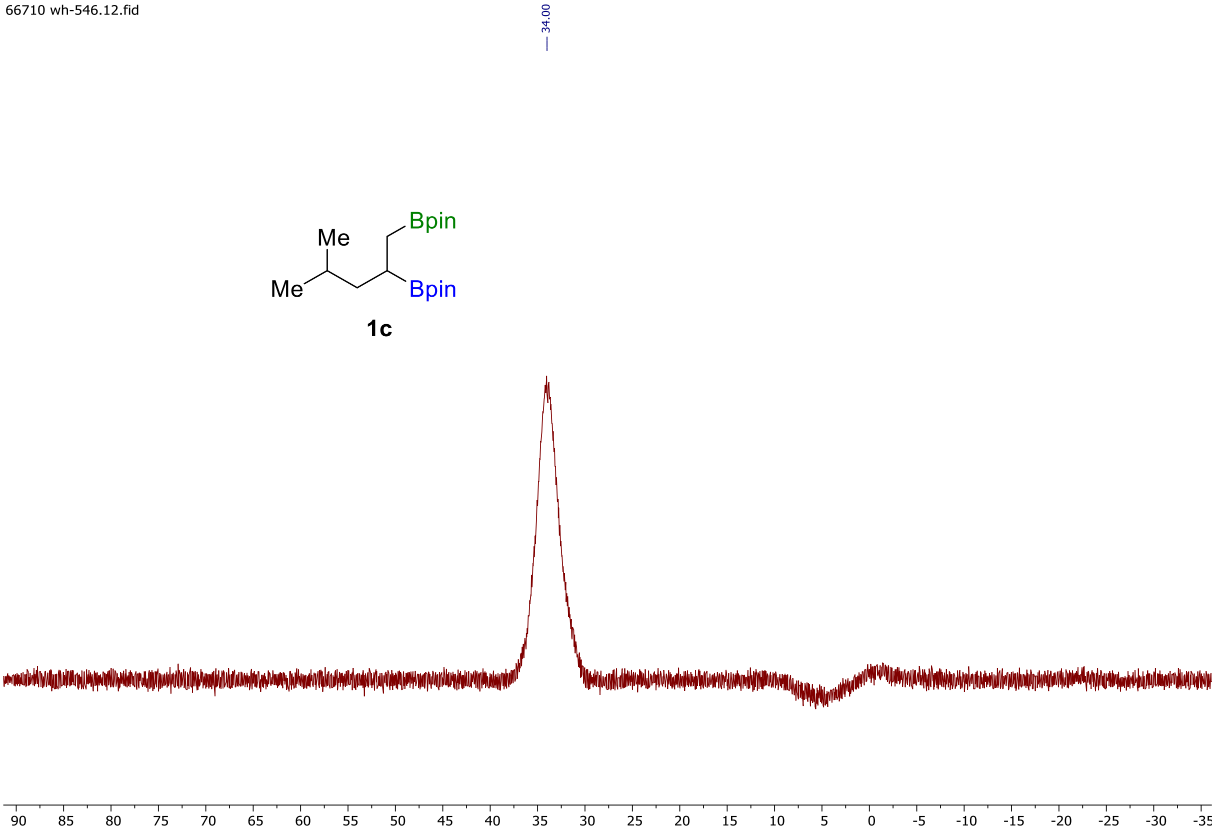

^{1}H NMR (400 MHz, $CDCl_3$) of **1d** (*see procedure*)

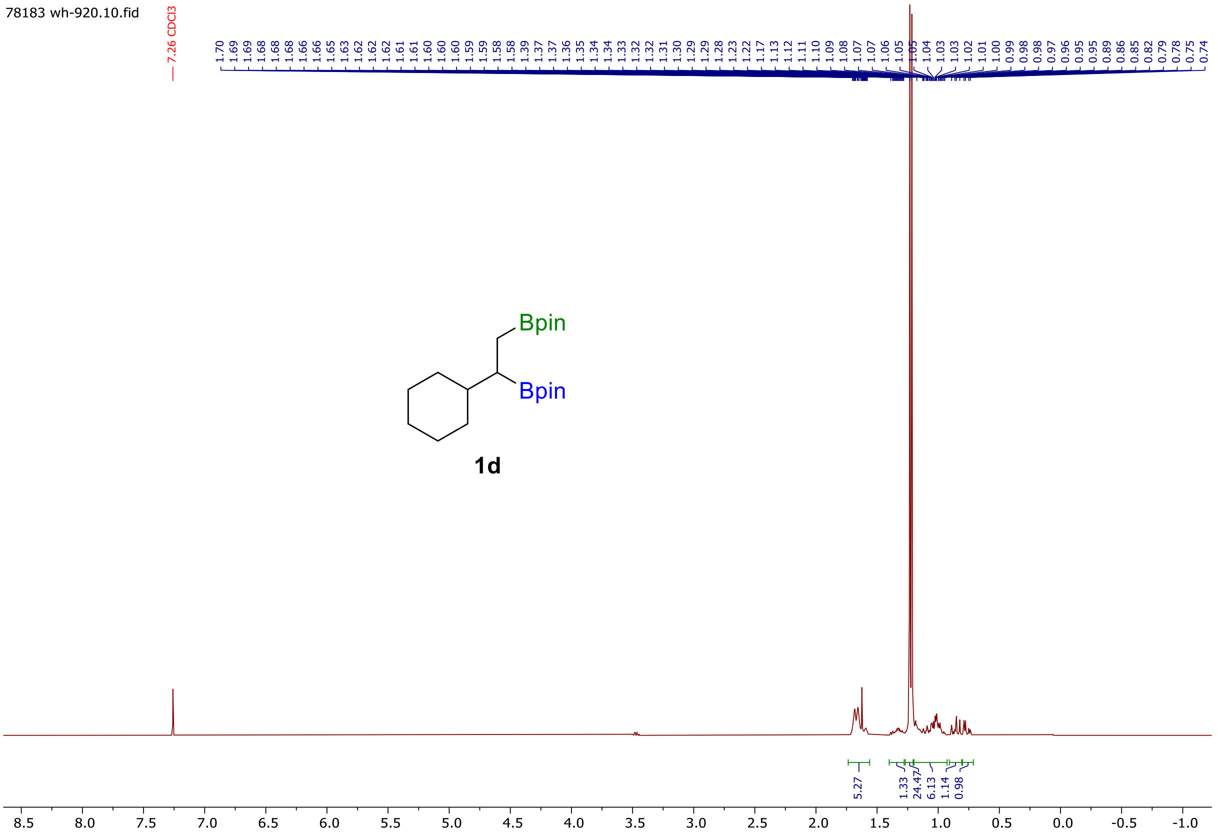

^{13}C NMR (101 MHz, $CDCl_3$) of **1d**

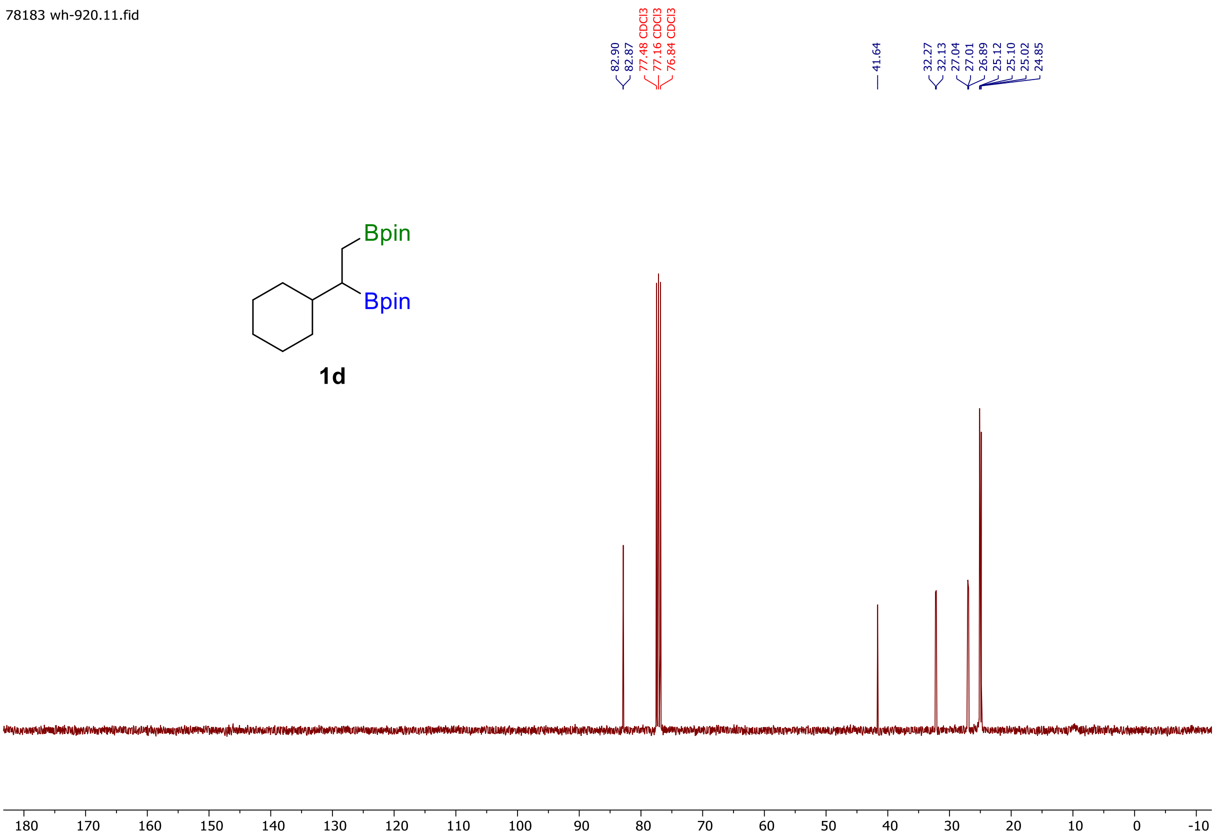

^{11}B NMR (128 MHz, $CDCl_3$) of **1d**

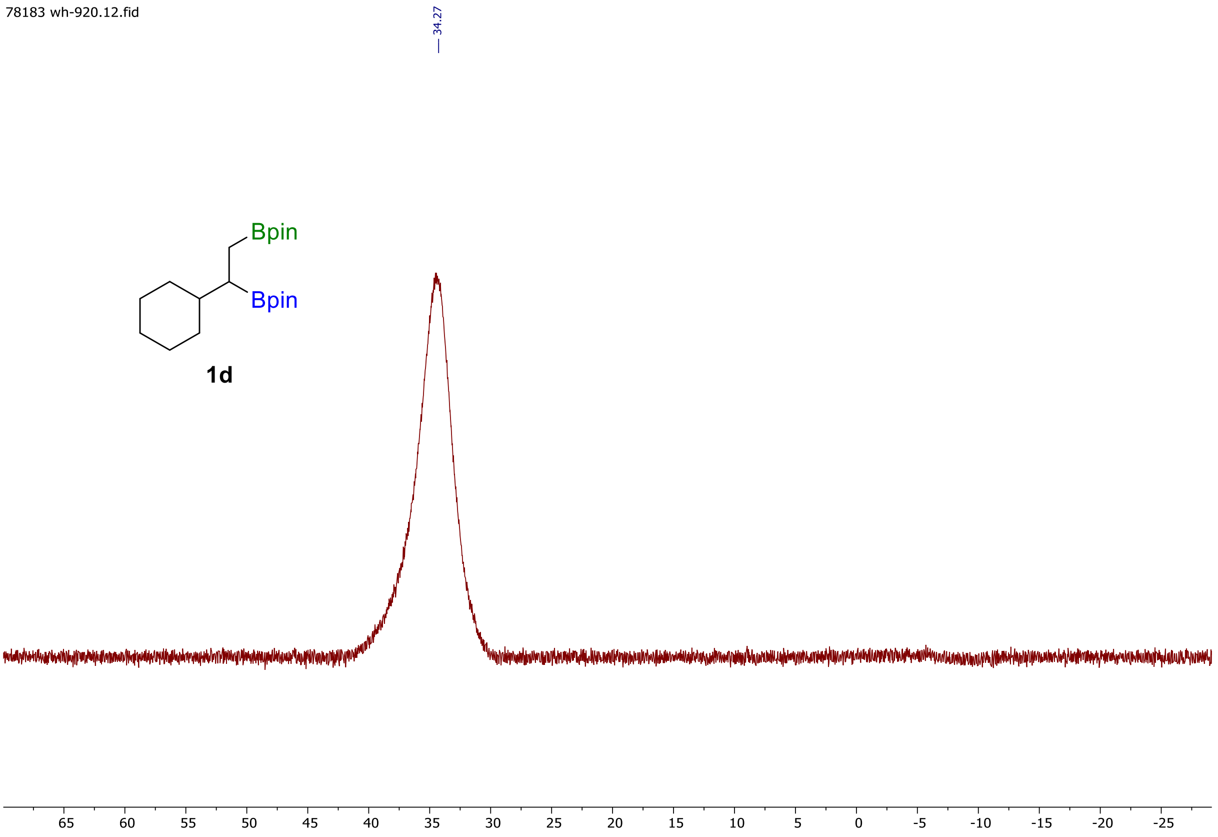

^{1}H NMR (400 MHz, $CDCl_3$) of **1e** (*see procedure*)

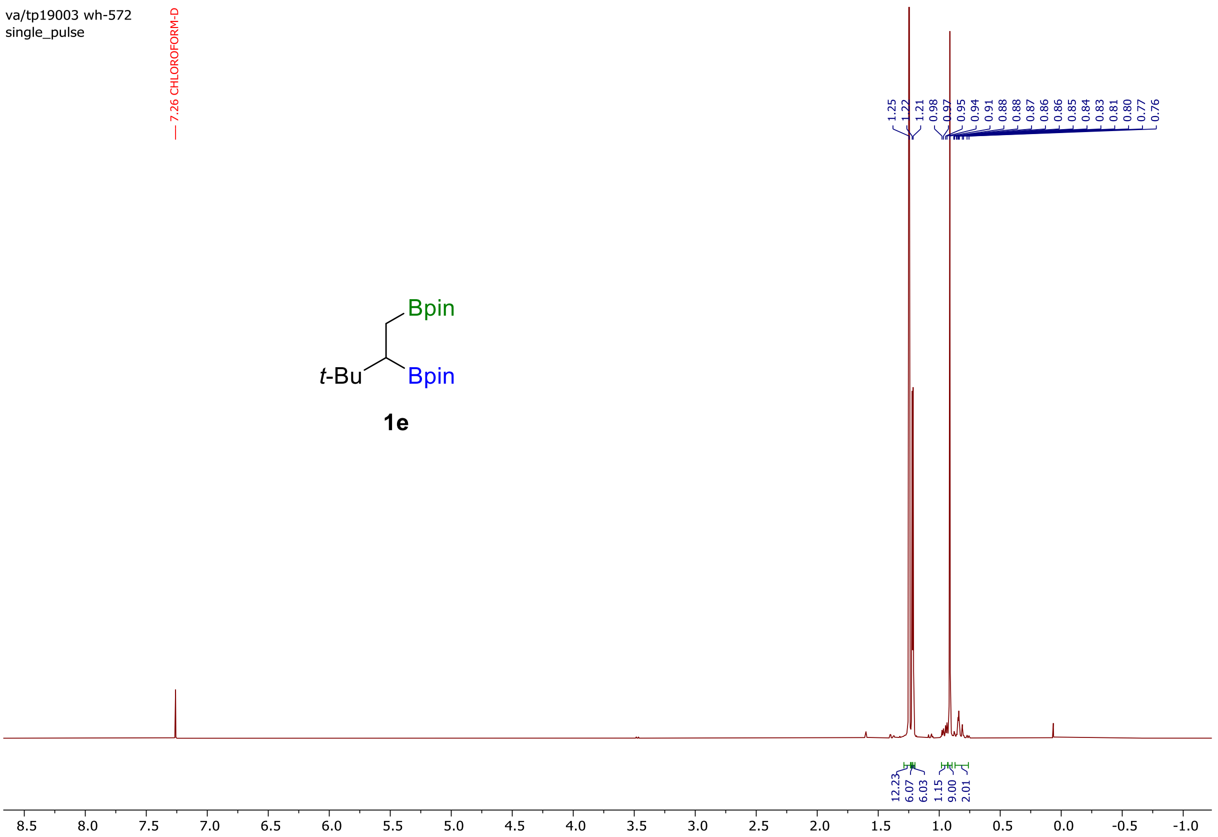

^{13}C NMR (101 MHz, $CDCl_3$) of **1e**

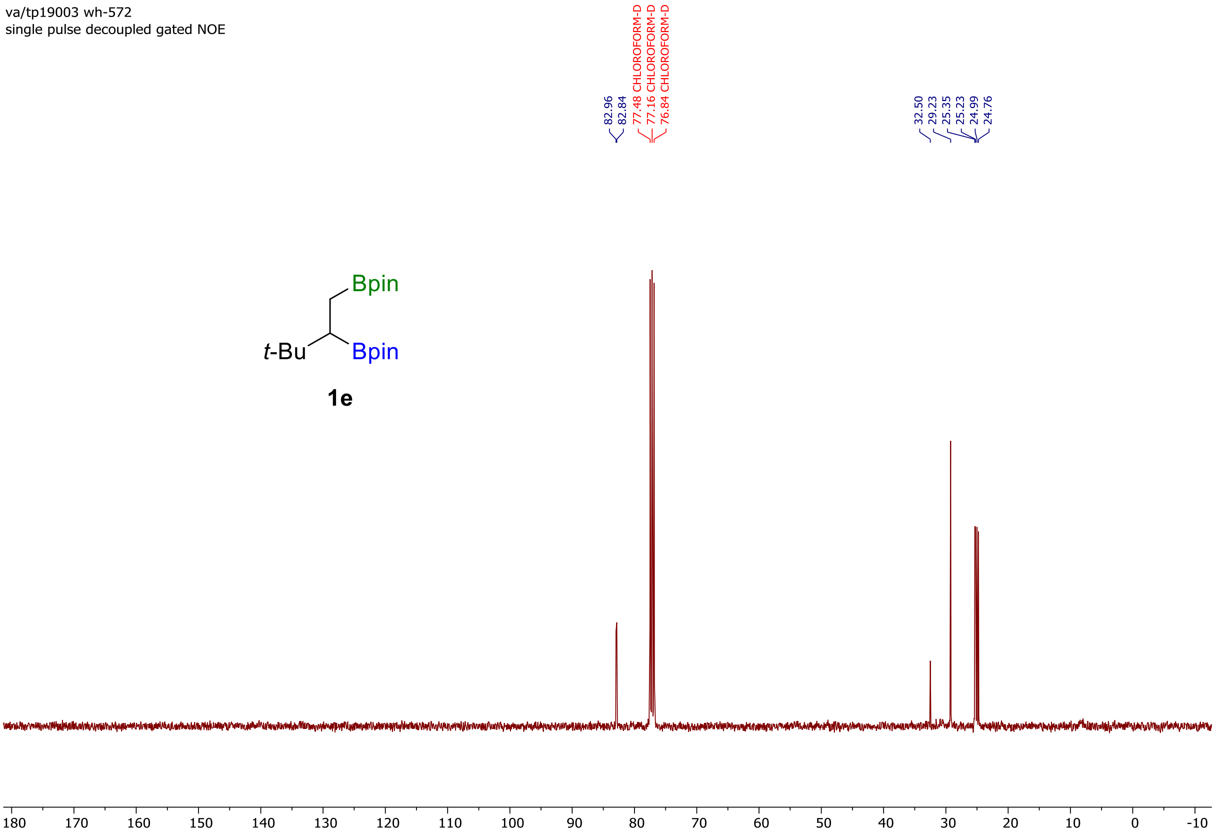

^{11}B NMR (128 MHz, $CDCl_3$) of **1e**

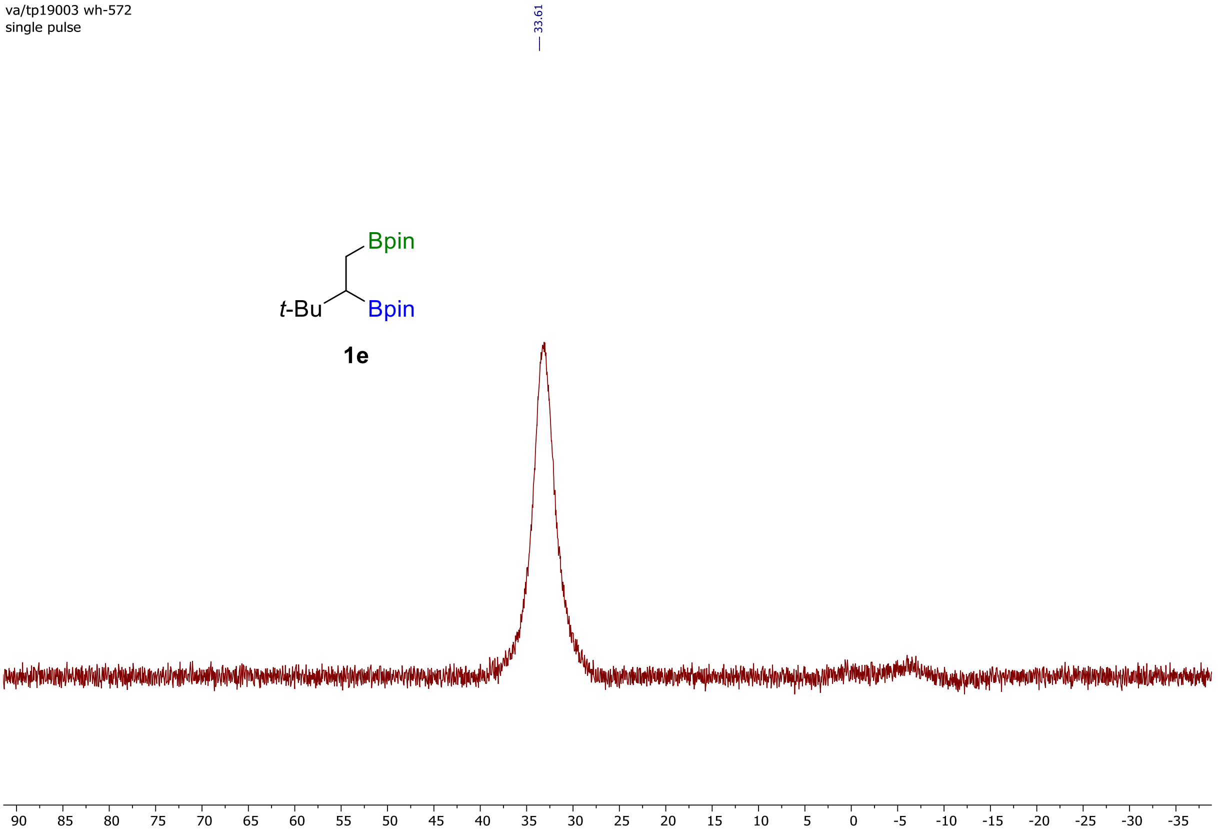

^{1}H NMR (400 MHz, $CDCl_3$) of **1f** (*see procedure*)

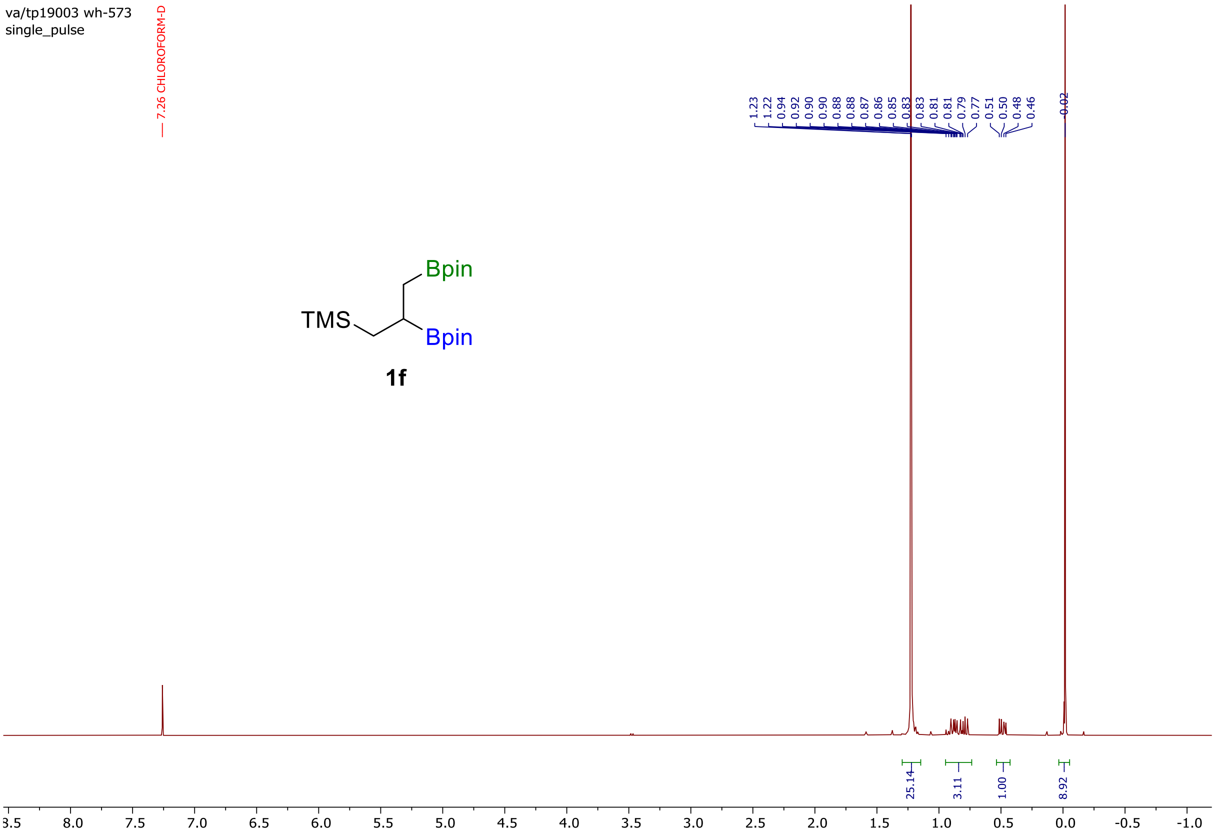

UOB Confidential & Sensitive

^{13}C NMR (101 MHz, CDCl$_3$) of **1f**

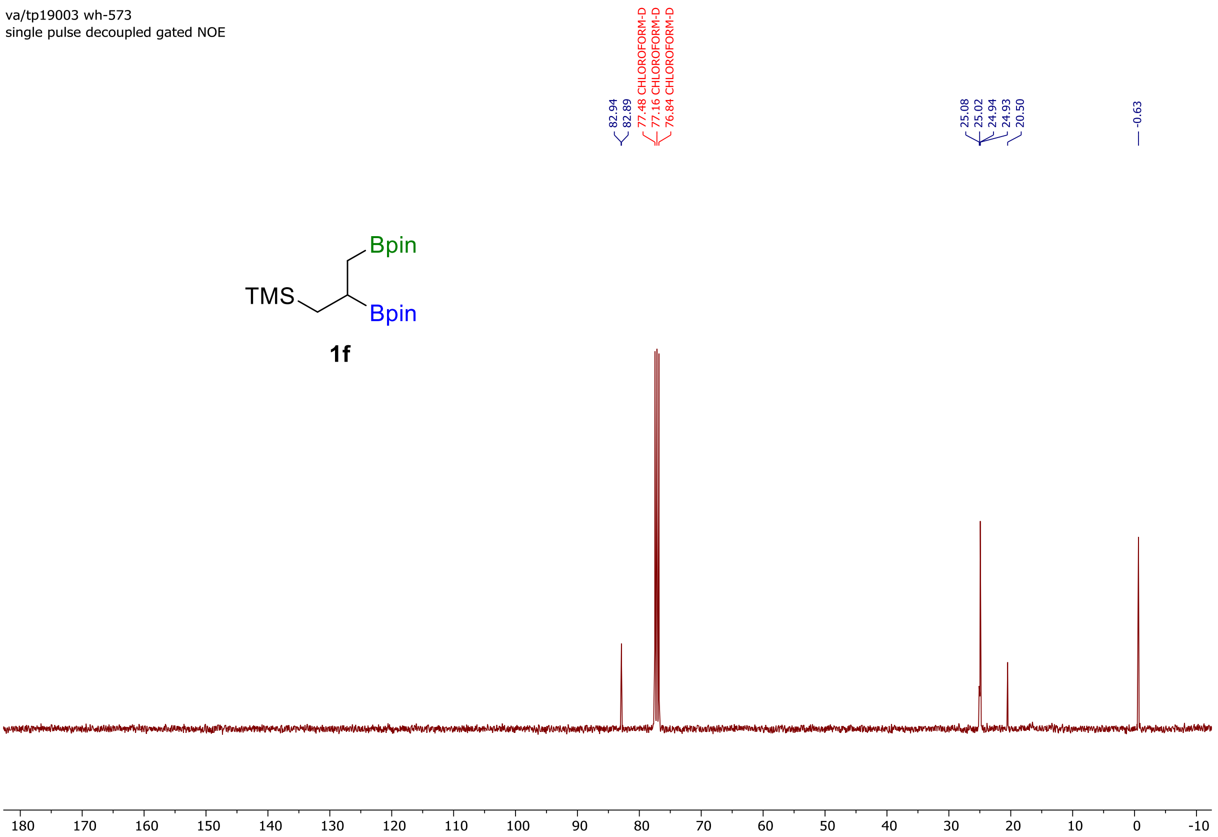

^{11}B NMR (128 MHz, CDCl$_3$) of **1f**

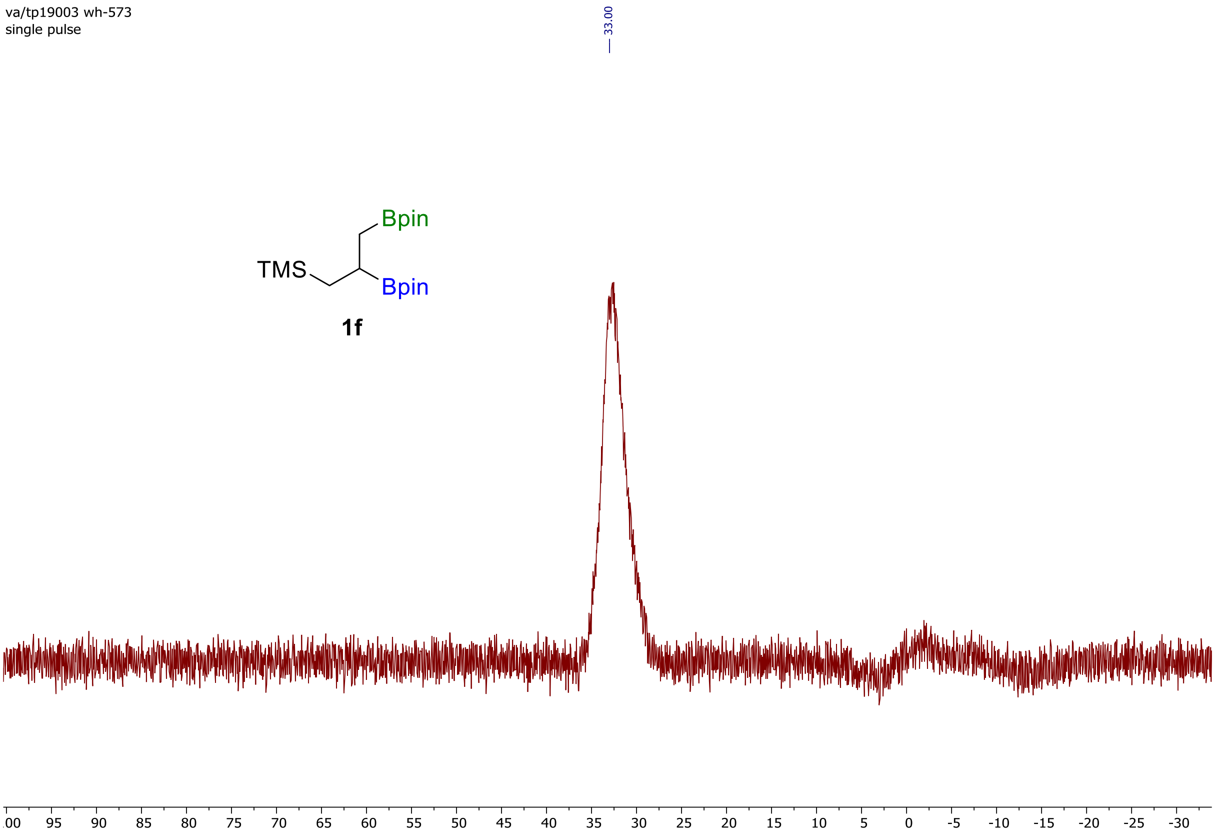

UOB Confidential & Sensitive

^{1}H NMR (400 MHz, $CDCl_3$) of **1g** (*see procedure*)

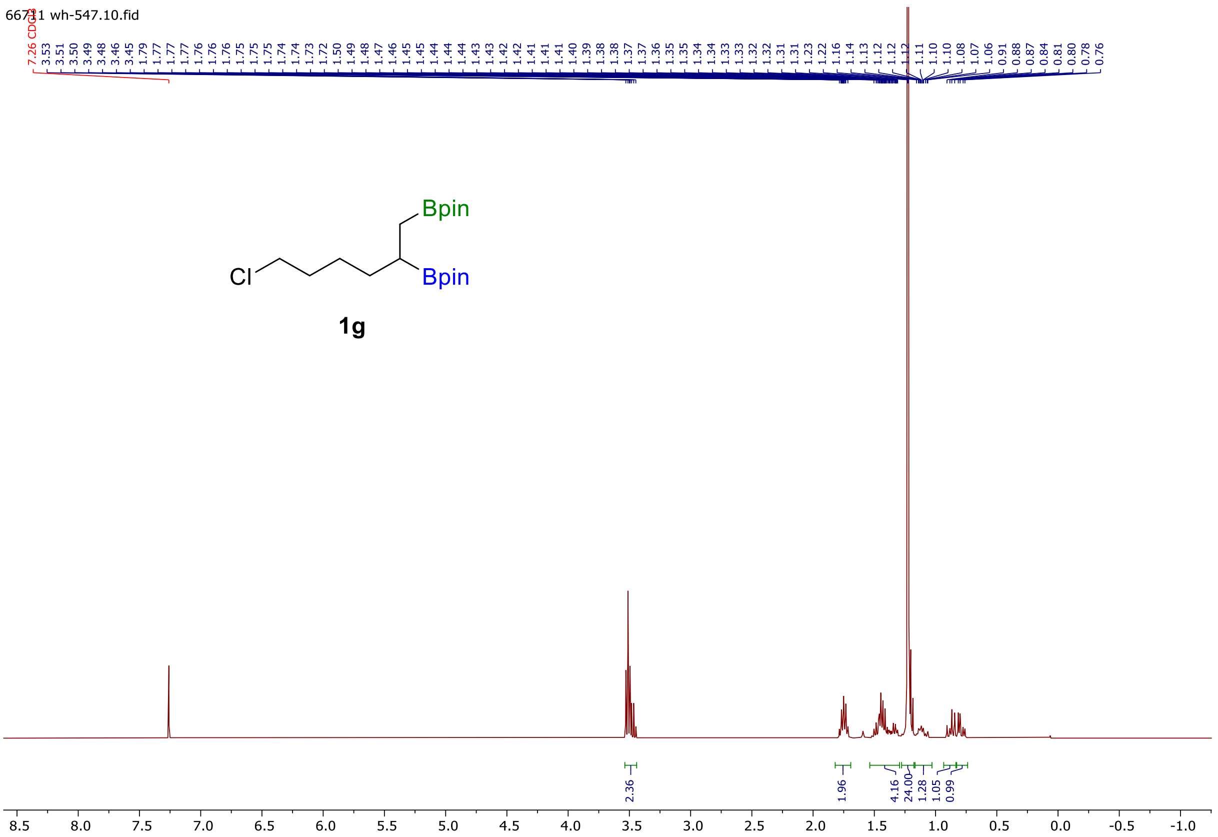

^{13}C NMR (101 MHz, $CDCl_3$) of **1g**

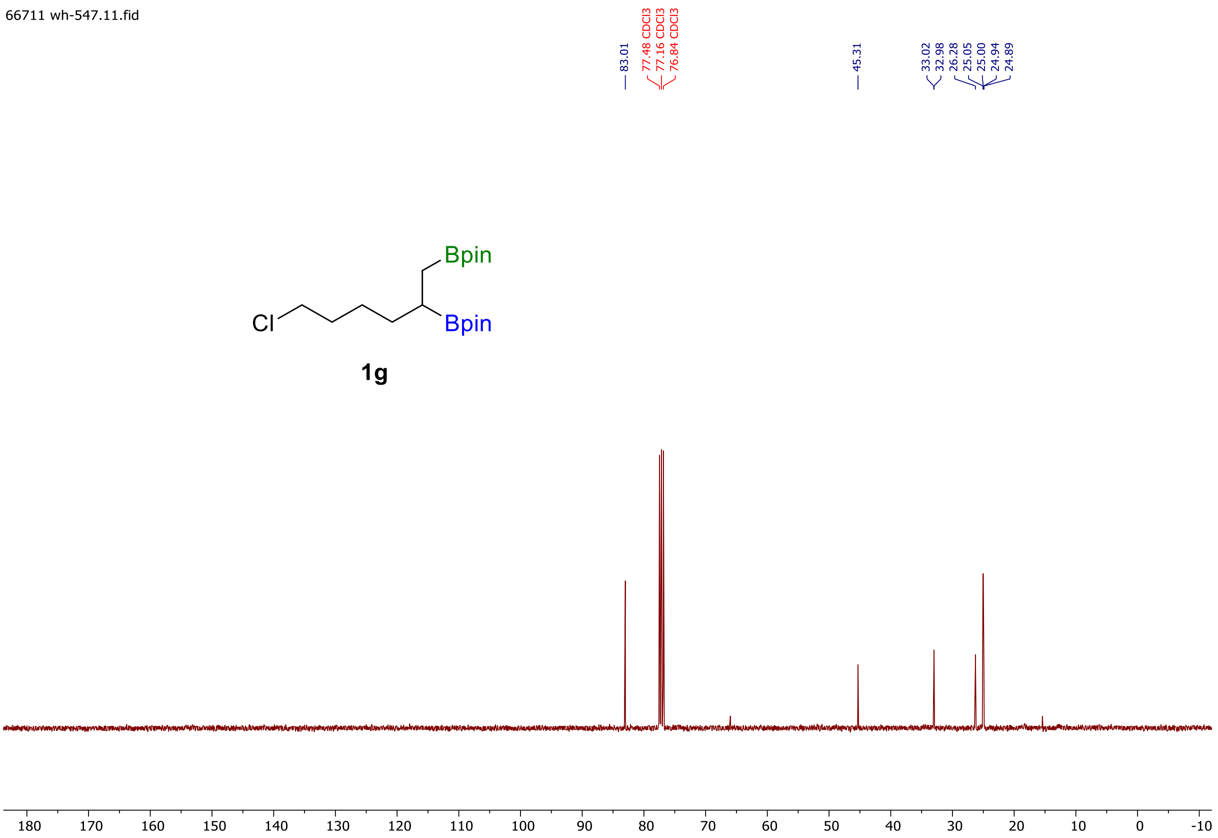

^{11}B NMR (128 MHz, $CDCl_3$) of **1g**

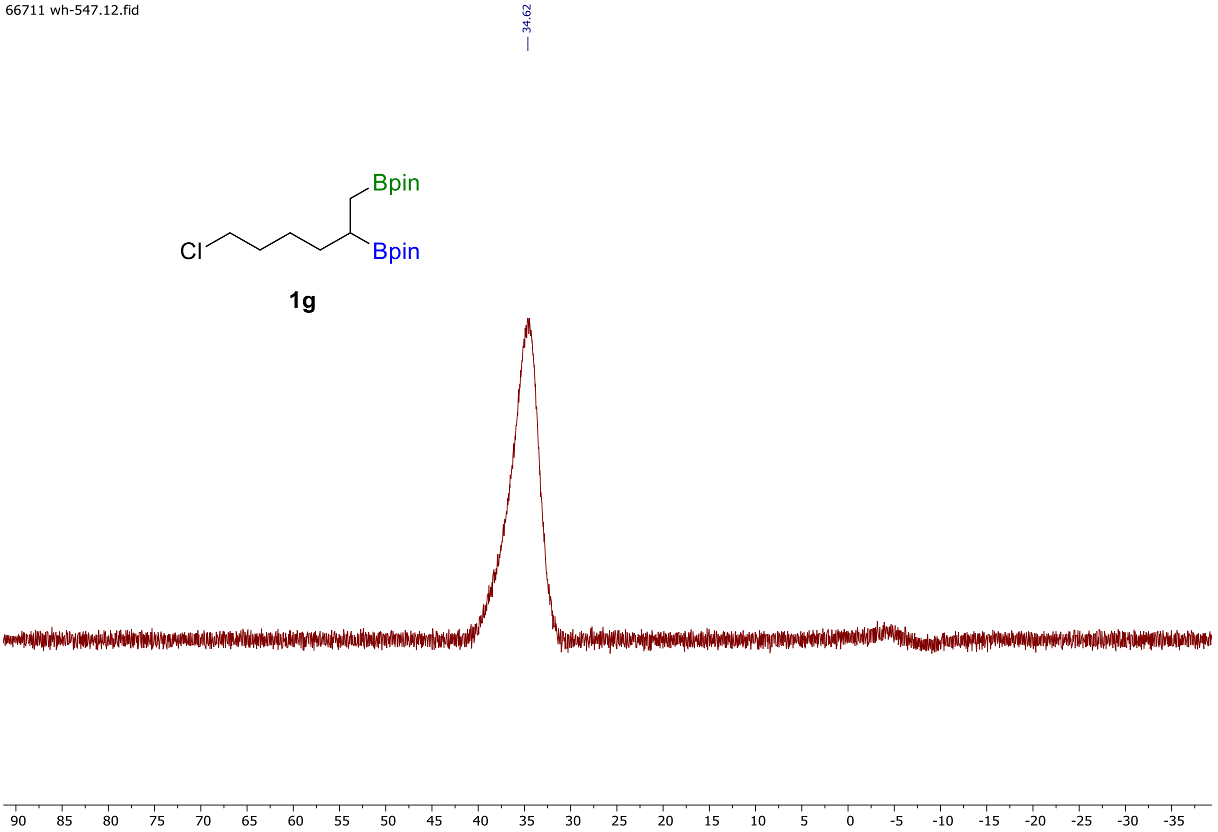

^{1}H NMR (400 MHz, $CDCl_3$) of **1h** (*see procedure*)

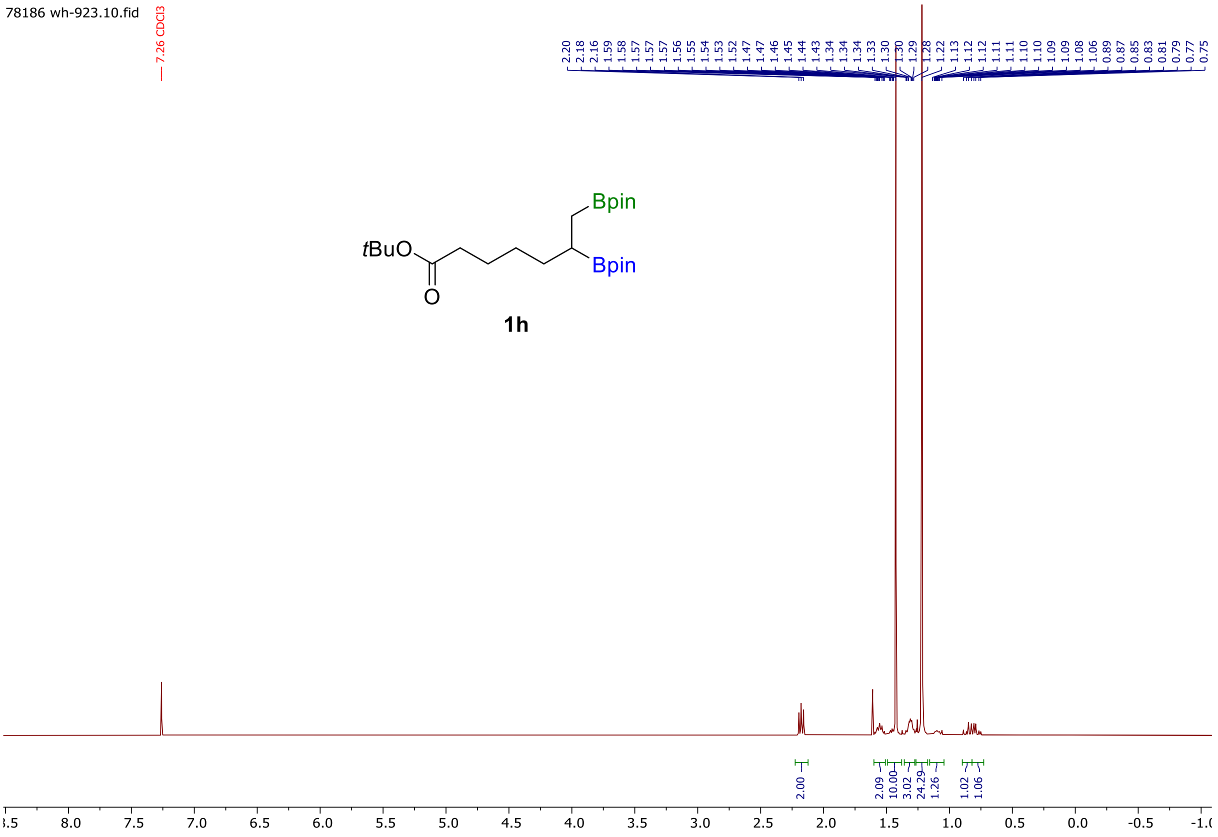

^{13}C NMR (101 MHz, $CDCl_3$) of **1h**

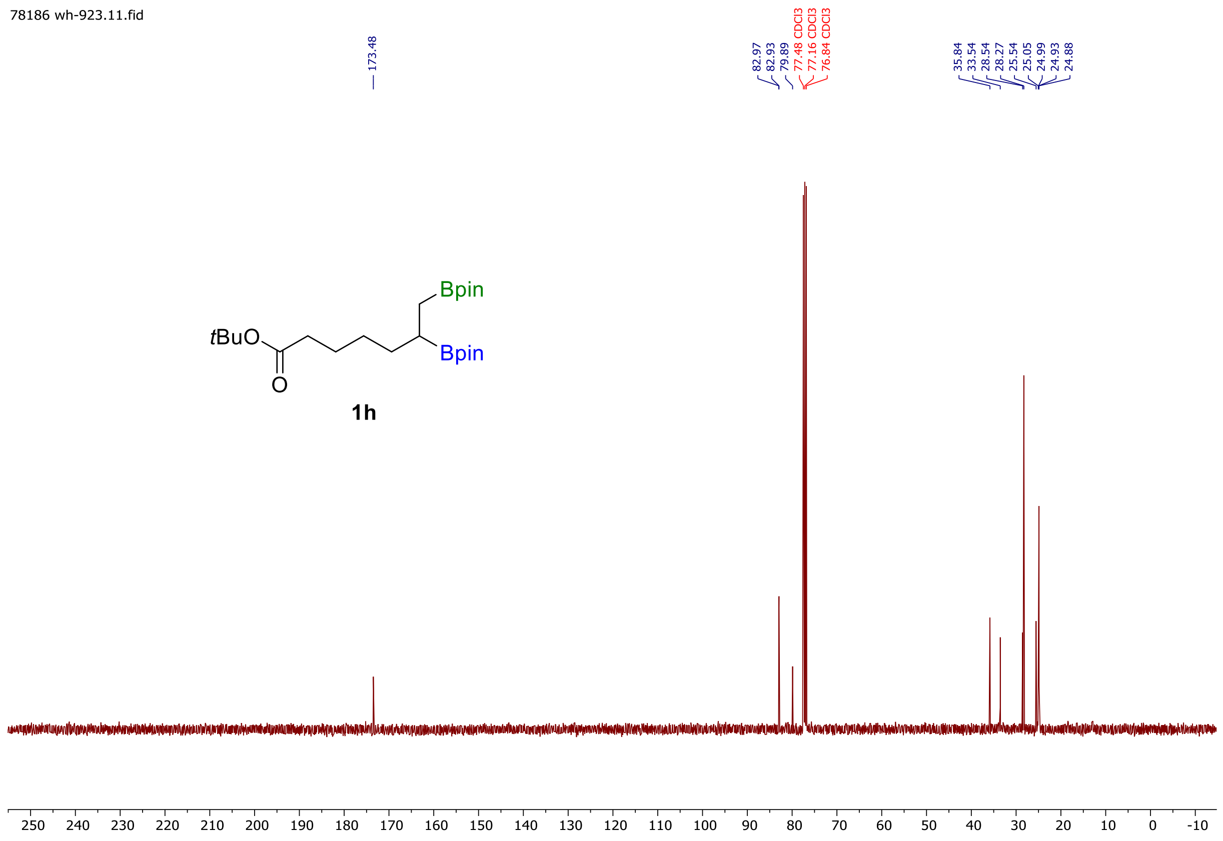

^{11}B NMR (128 MHz, $CDCl_3$) of **1h**

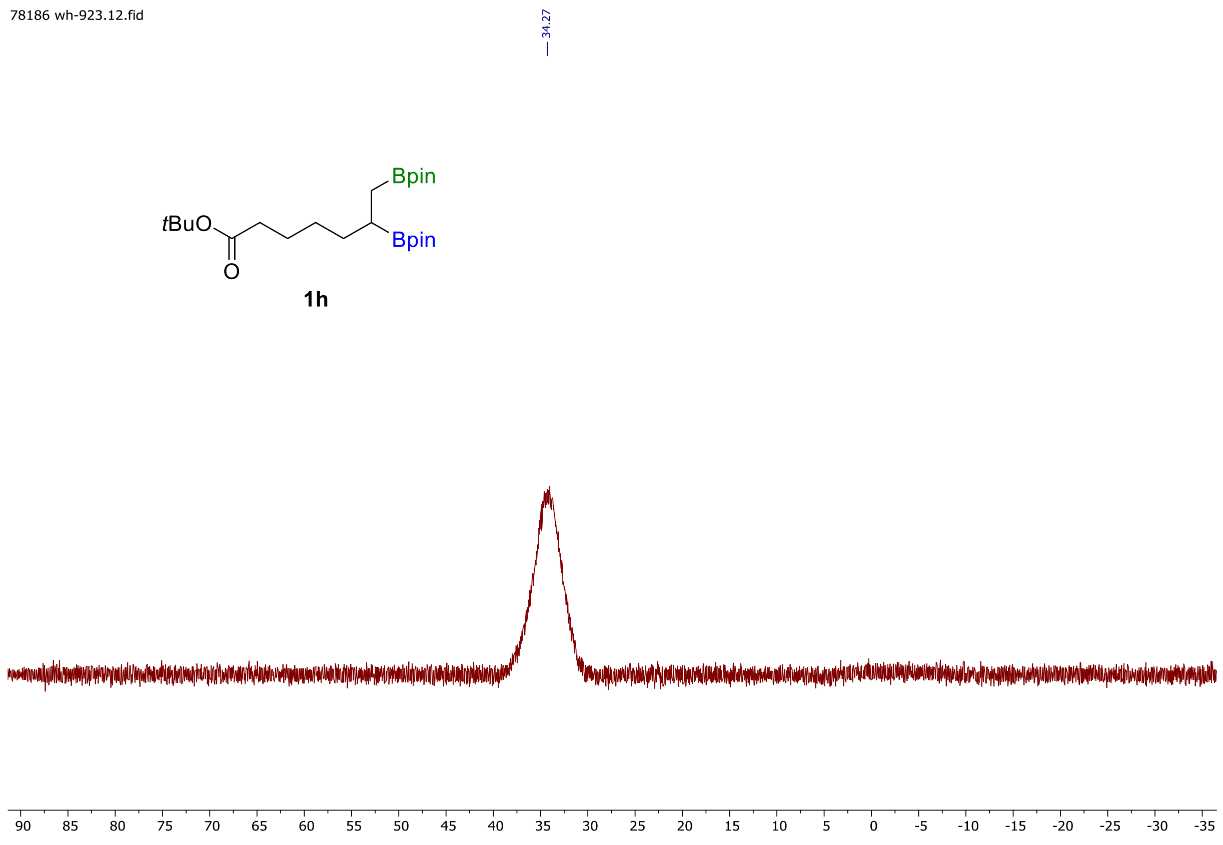

^{1}H NMR (400 MHz, $CDCl_3$) of **1i** (*see procedure*)

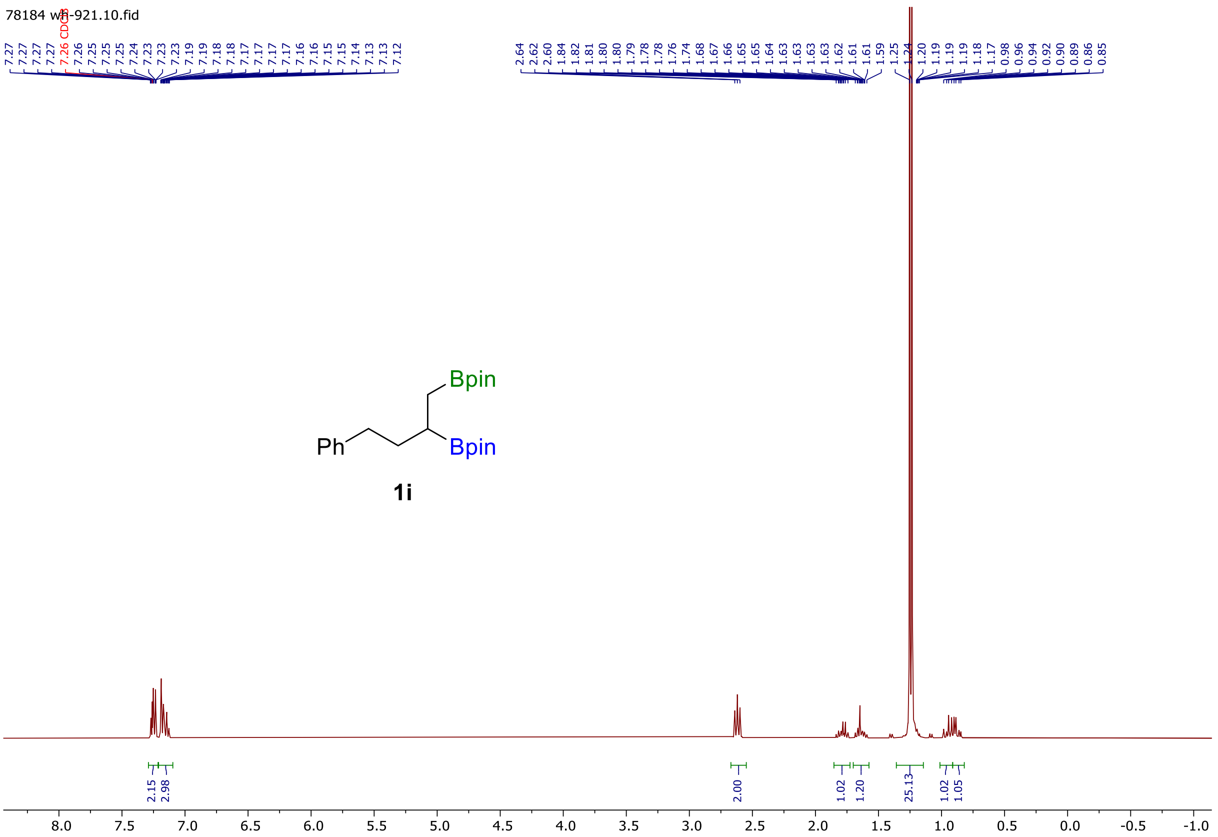

^{13}C NMR (101 MHz, $CDCl_3$) of **1i**

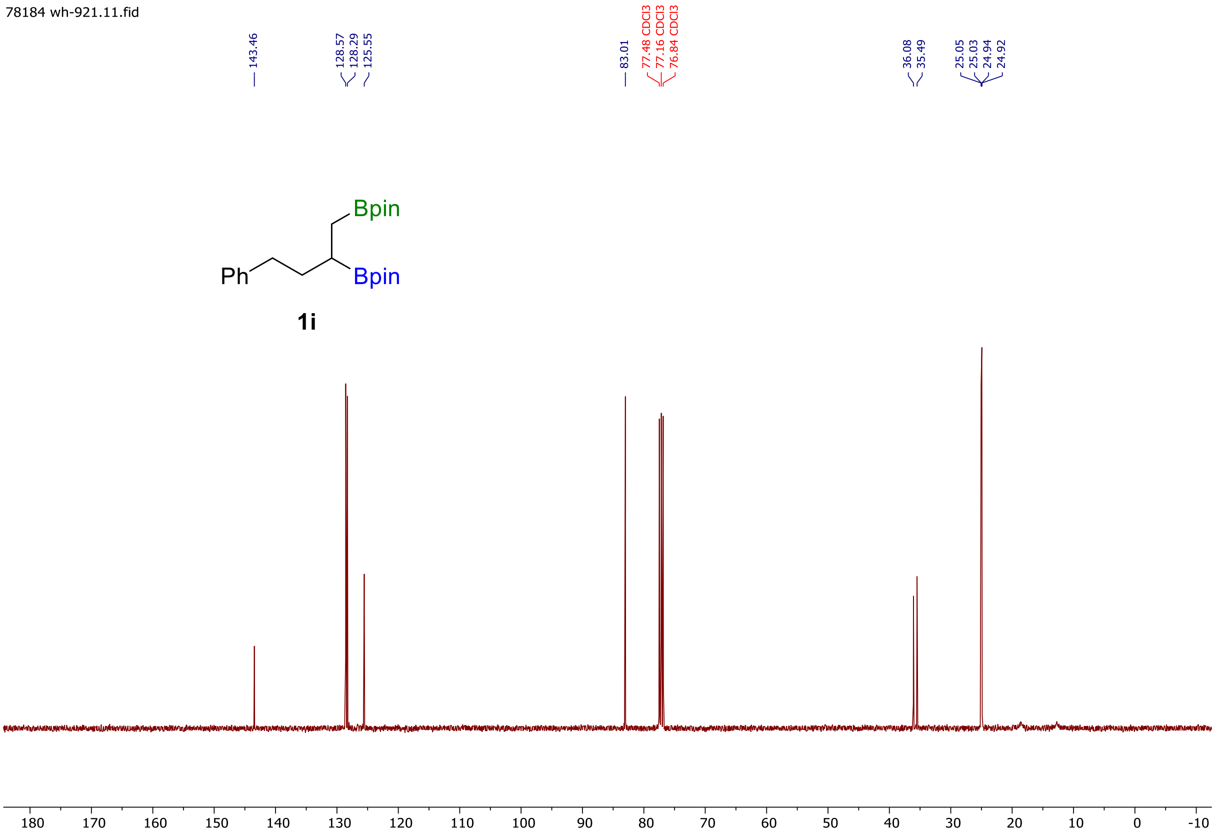

^{11}B NMR (128 MHz, $CDCl_3$) of **1i**

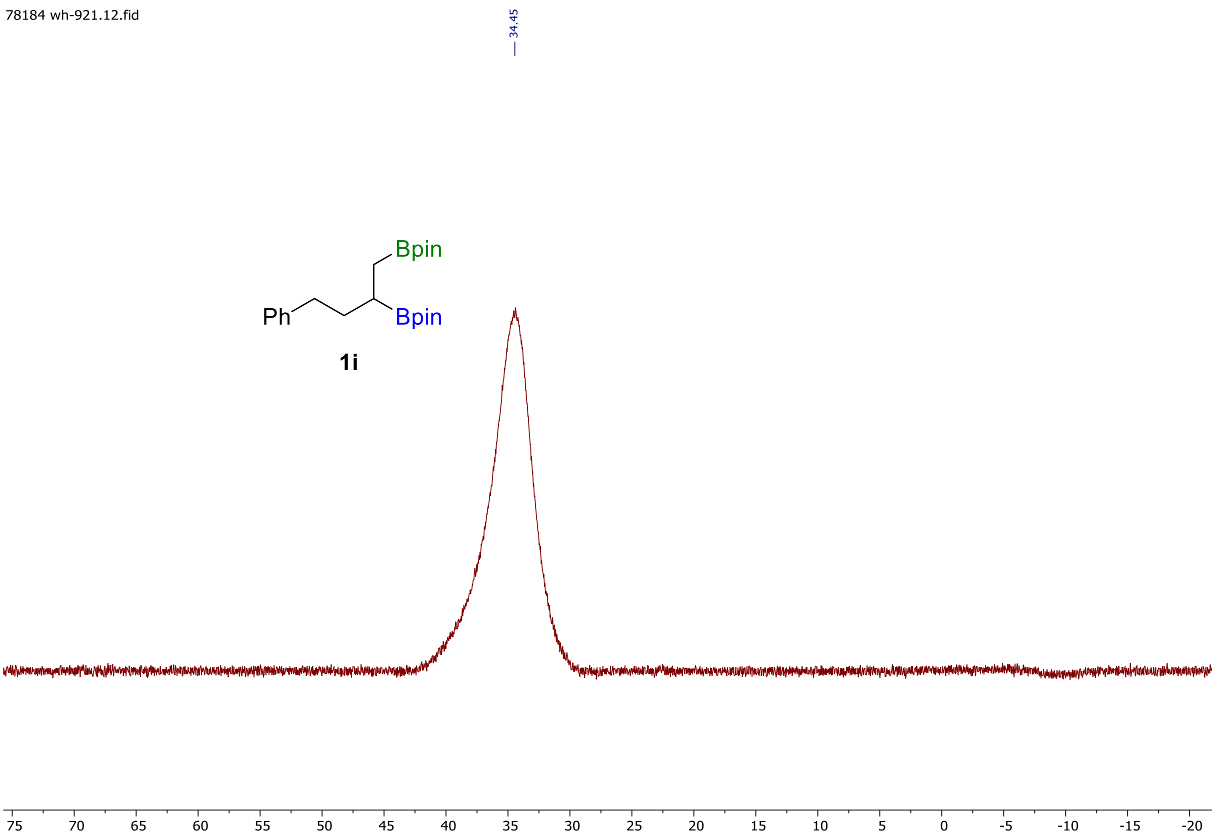

^{1}H NMR (400 MHz, $CDCl_3$) of **1j** (*see procedure*)

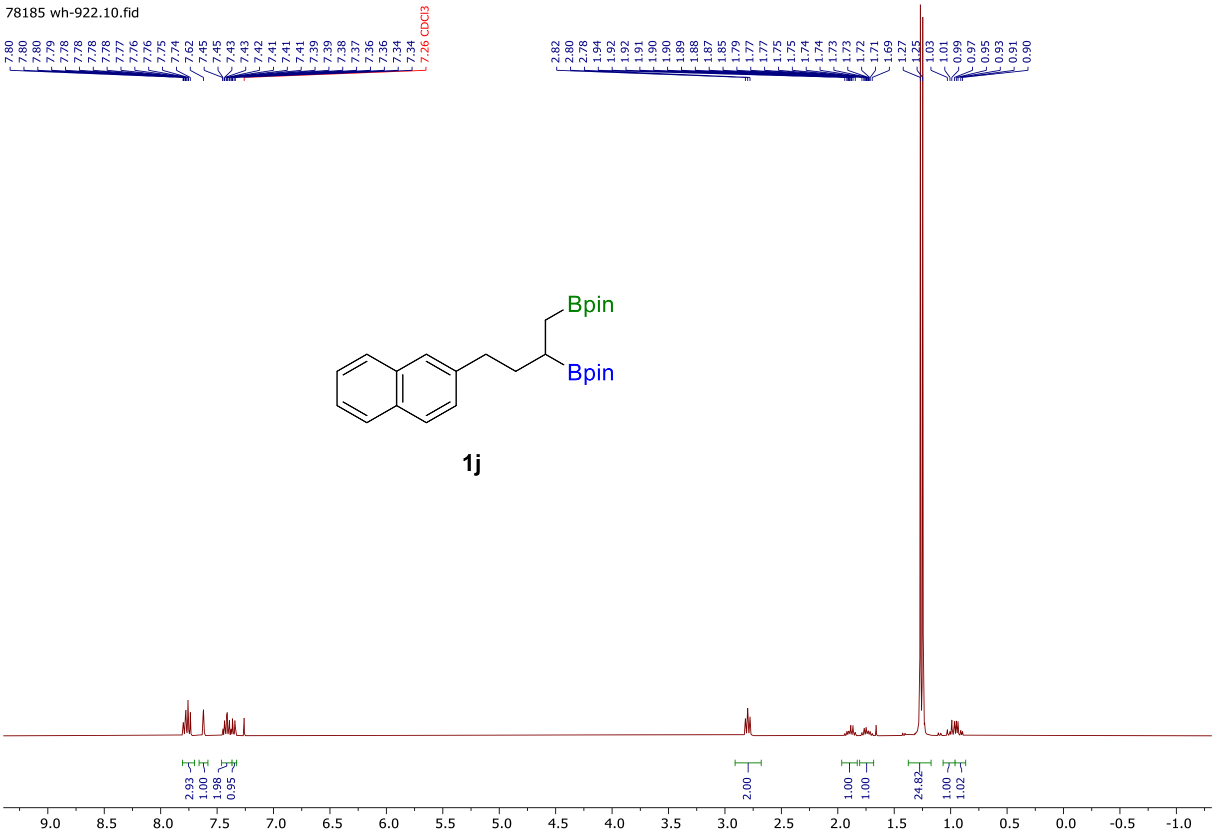

UOB Confidential & Sensitive

^{13}C NMR (101 MHz, $CDCl_3$) of **1j**

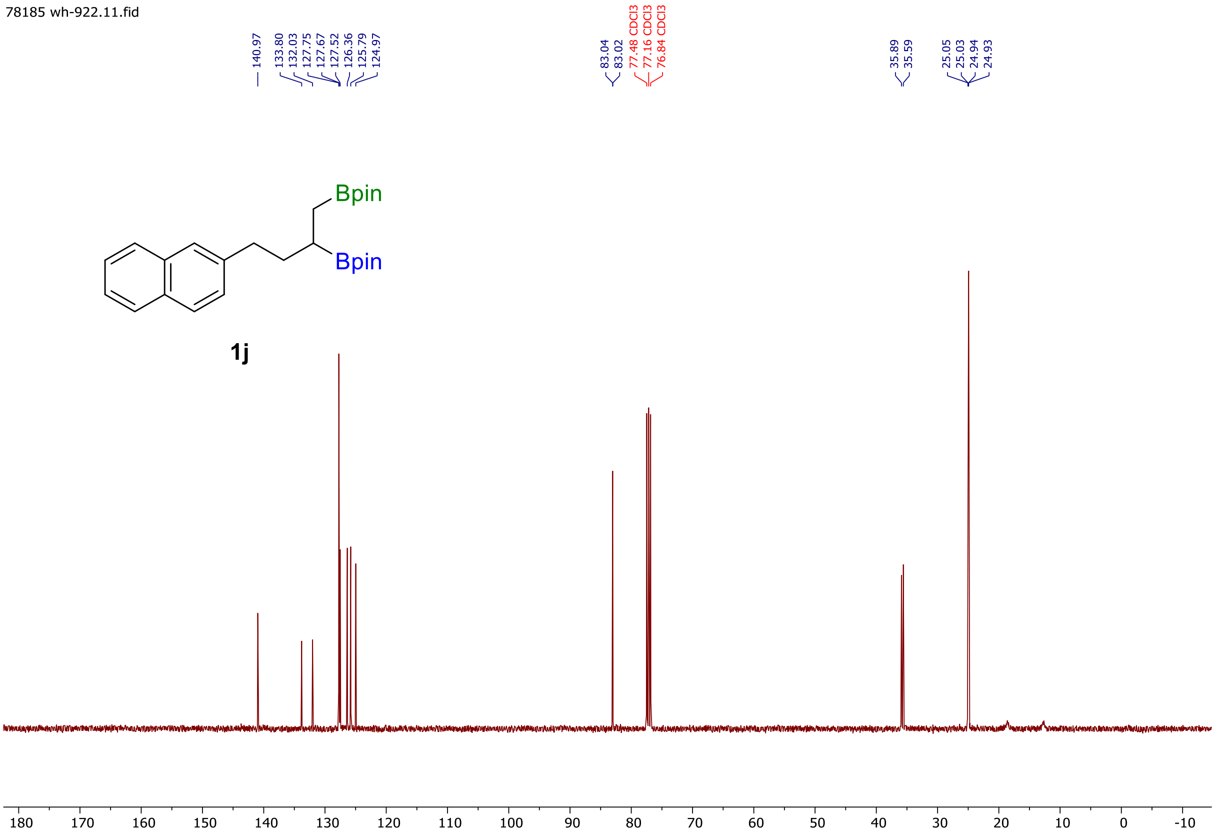

^{11}B NMR (128 MHz, $CDCl_3$) of **1j**

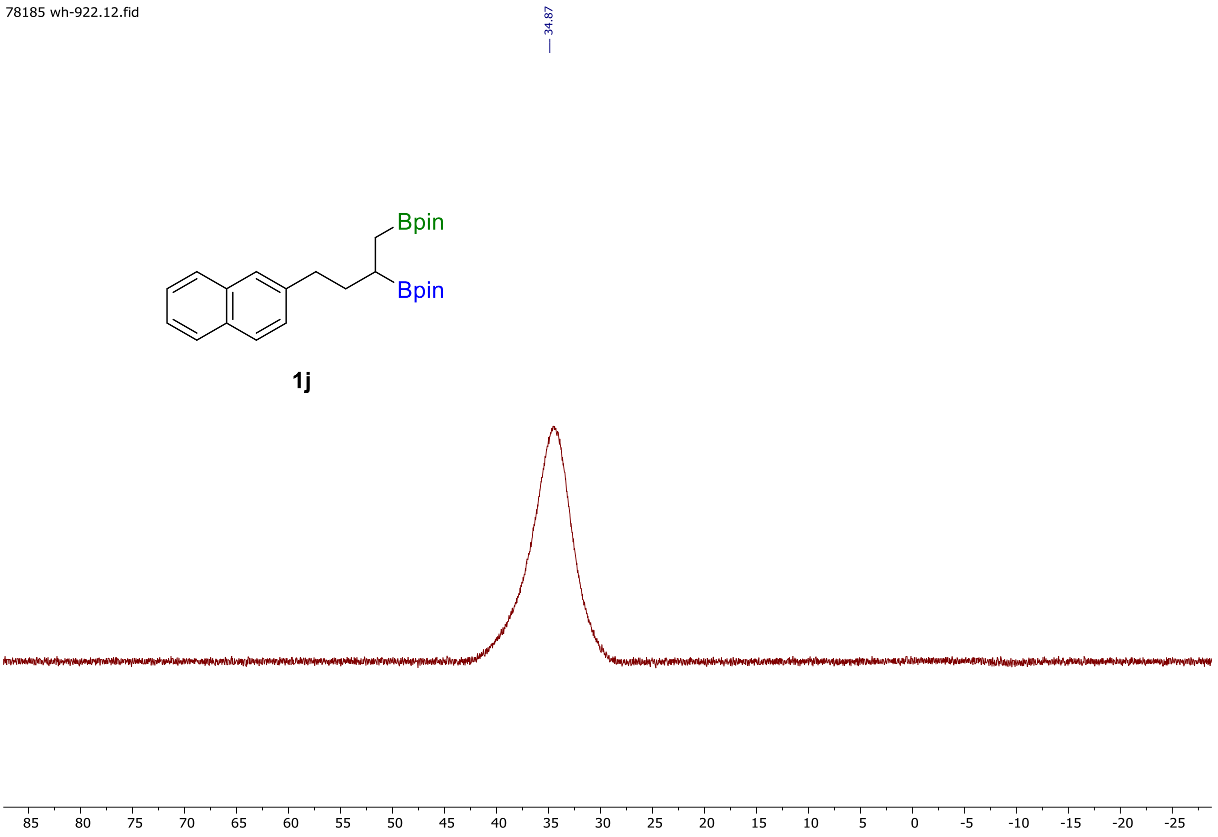

^{1}H NMR (400 MHz, $CDCl_3$) of **1k** (*see procedure*)

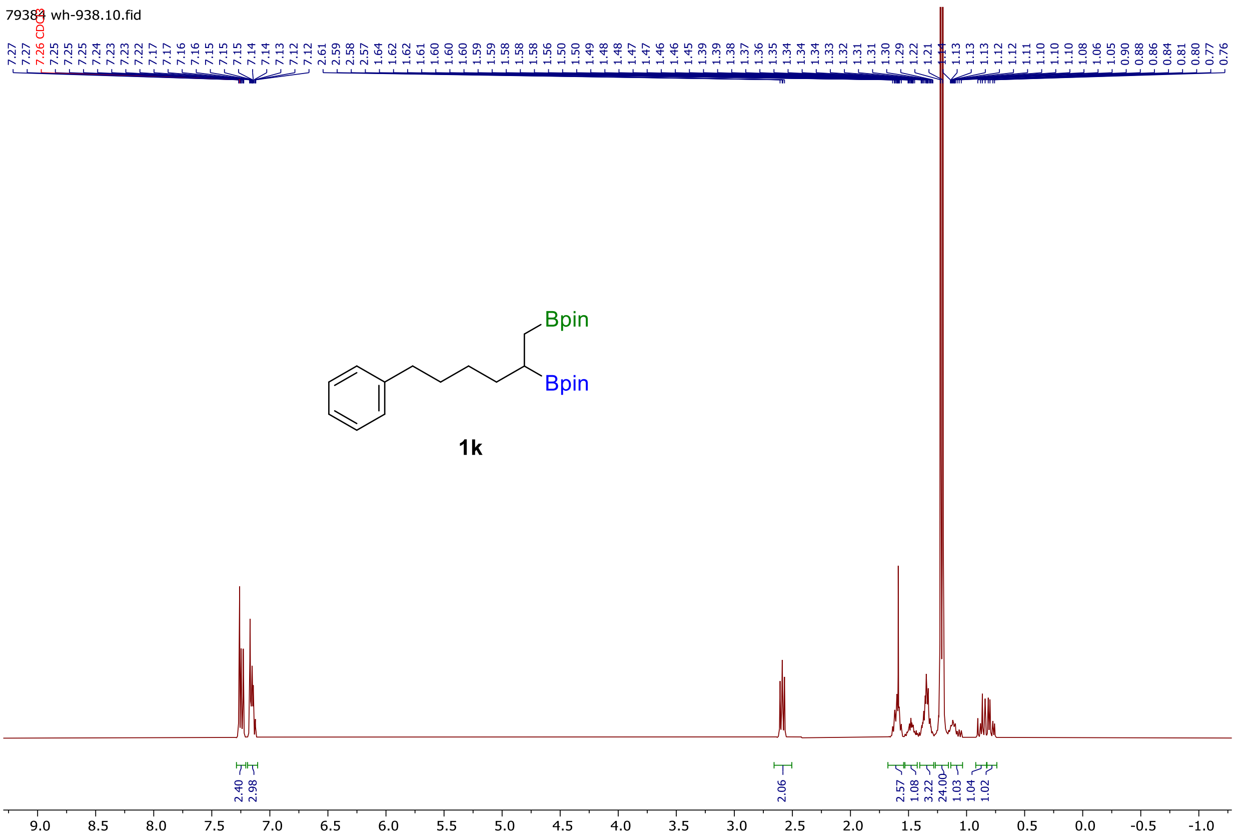

^{13}C NMR (101 MHz, $CDCl_3$) of **1k**

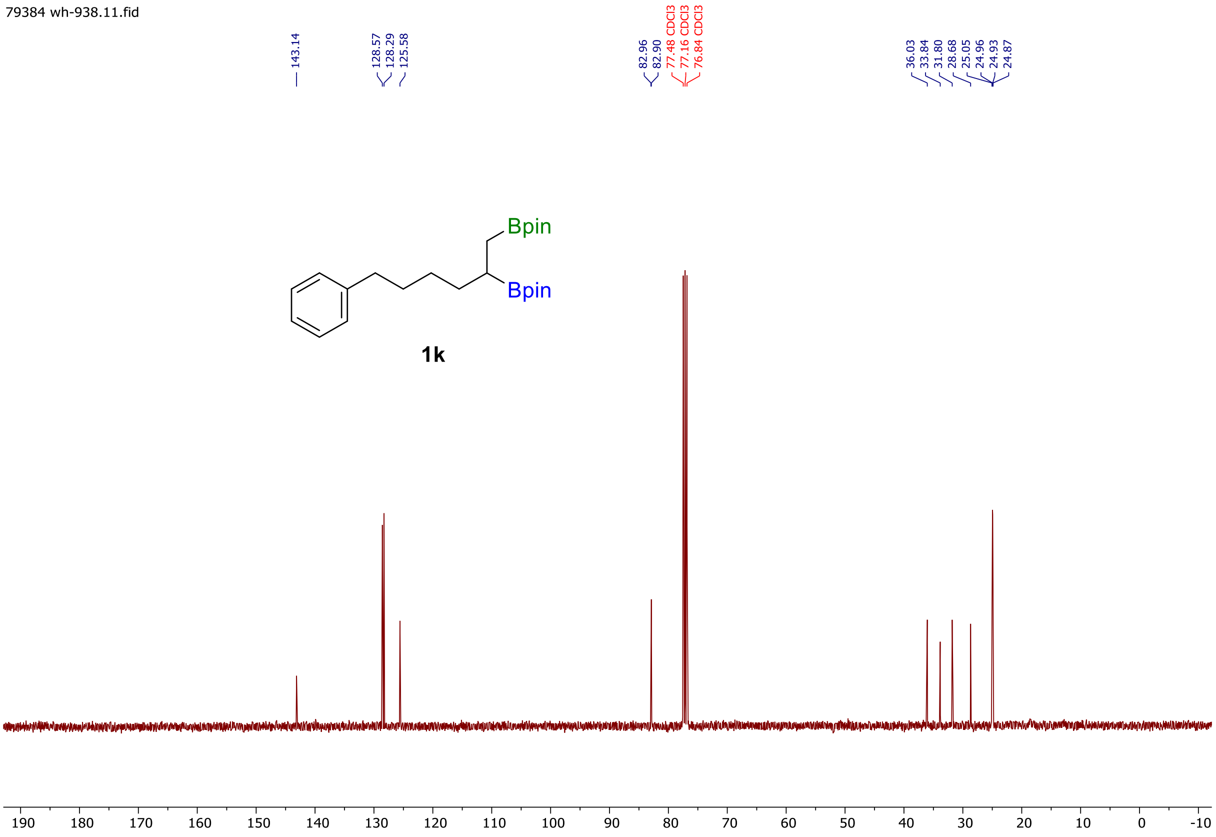

UOB Confidential & Sensitive

^{11}B NMR (128 MHz, $CDCl_3$) of **1k**

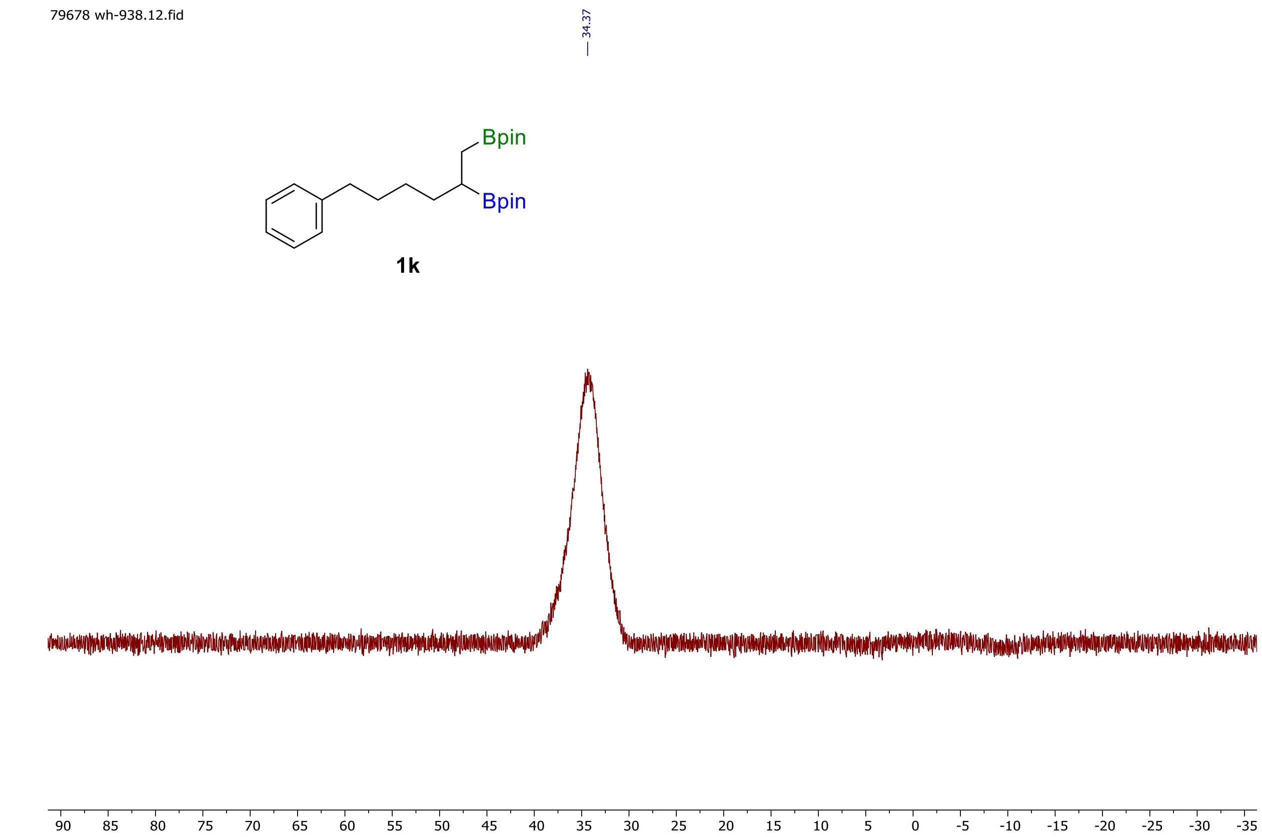

^{1}H NMR (400 MHz, $CDCl_3$) of **1l** (*see procedure*)

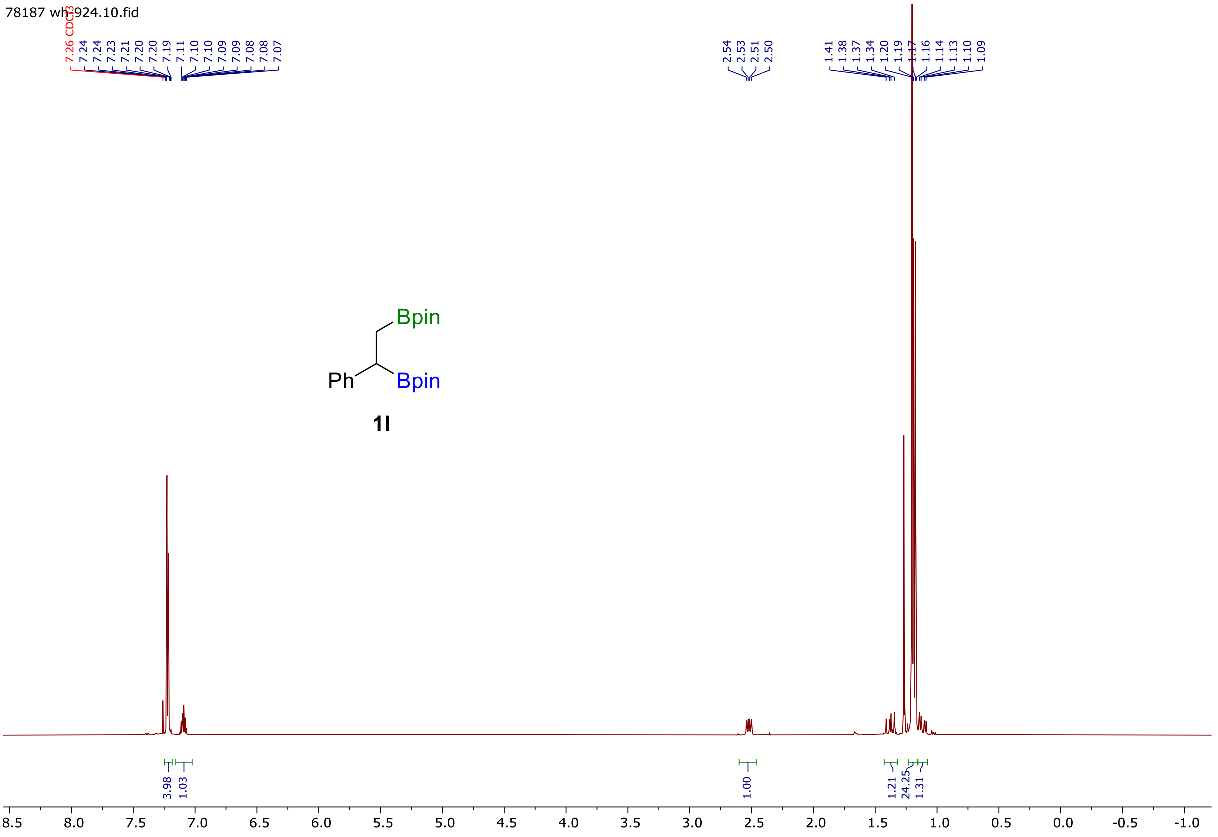

^{13}C NMR (101 MHz, $CDCl_3$) of **1l**

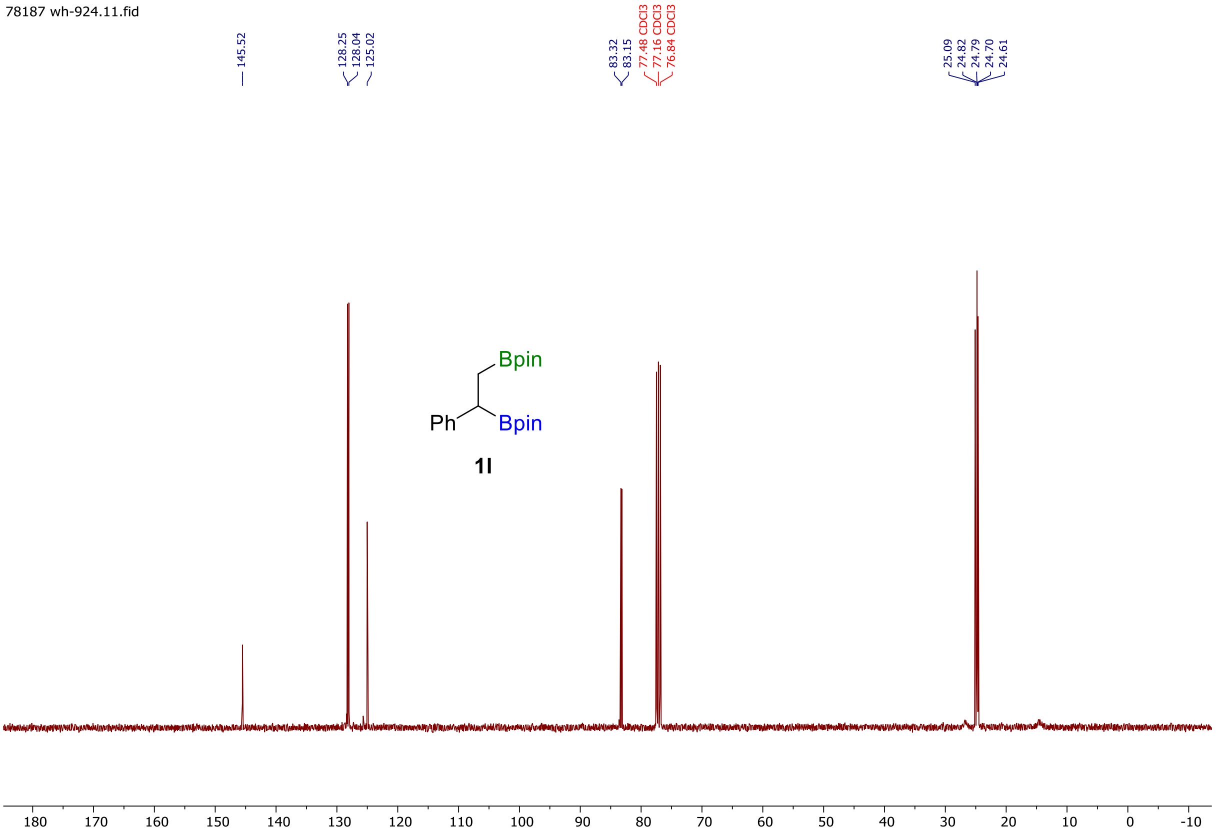

^{1}H NMR (400 MHz, $CDCl_3$) of **1m** (*see procedure*)

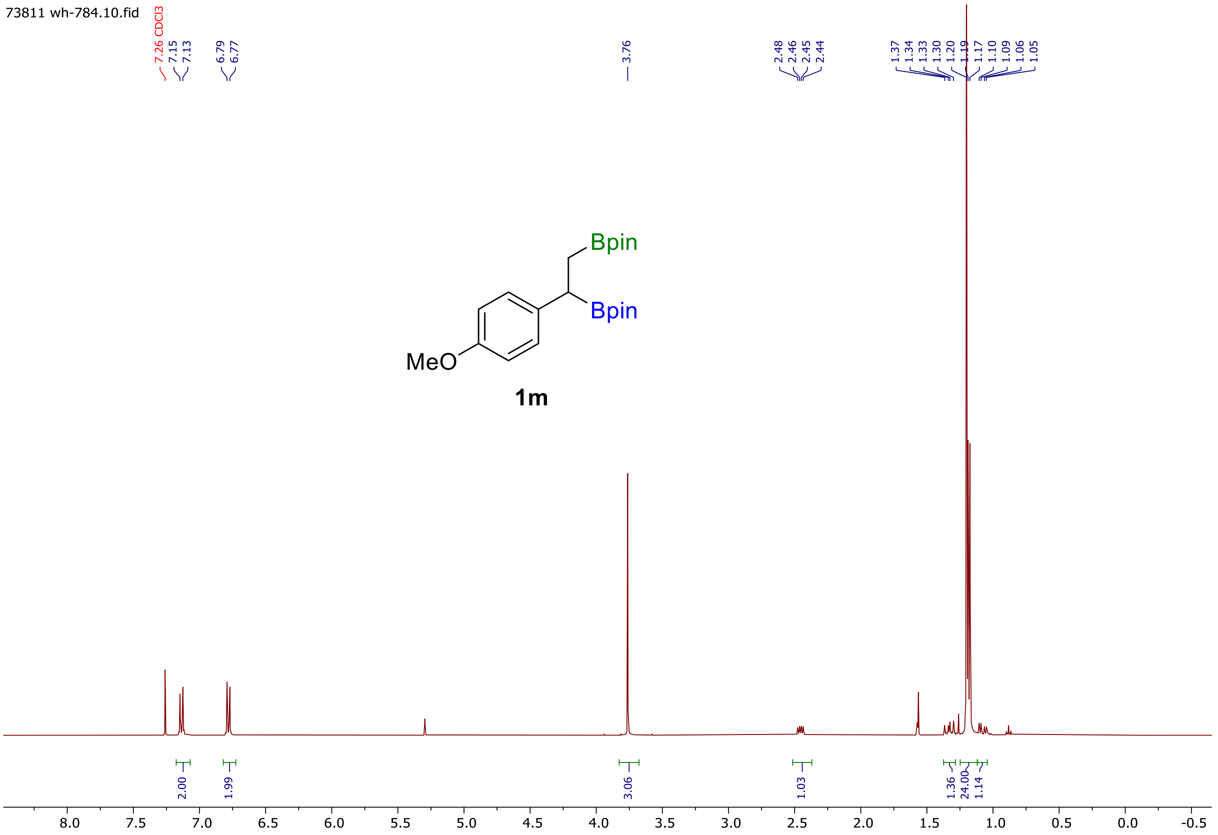

UOB Confidential & Sensitive

^{13}C NMR (101 MHz, $CDCl_3$) of **1m**

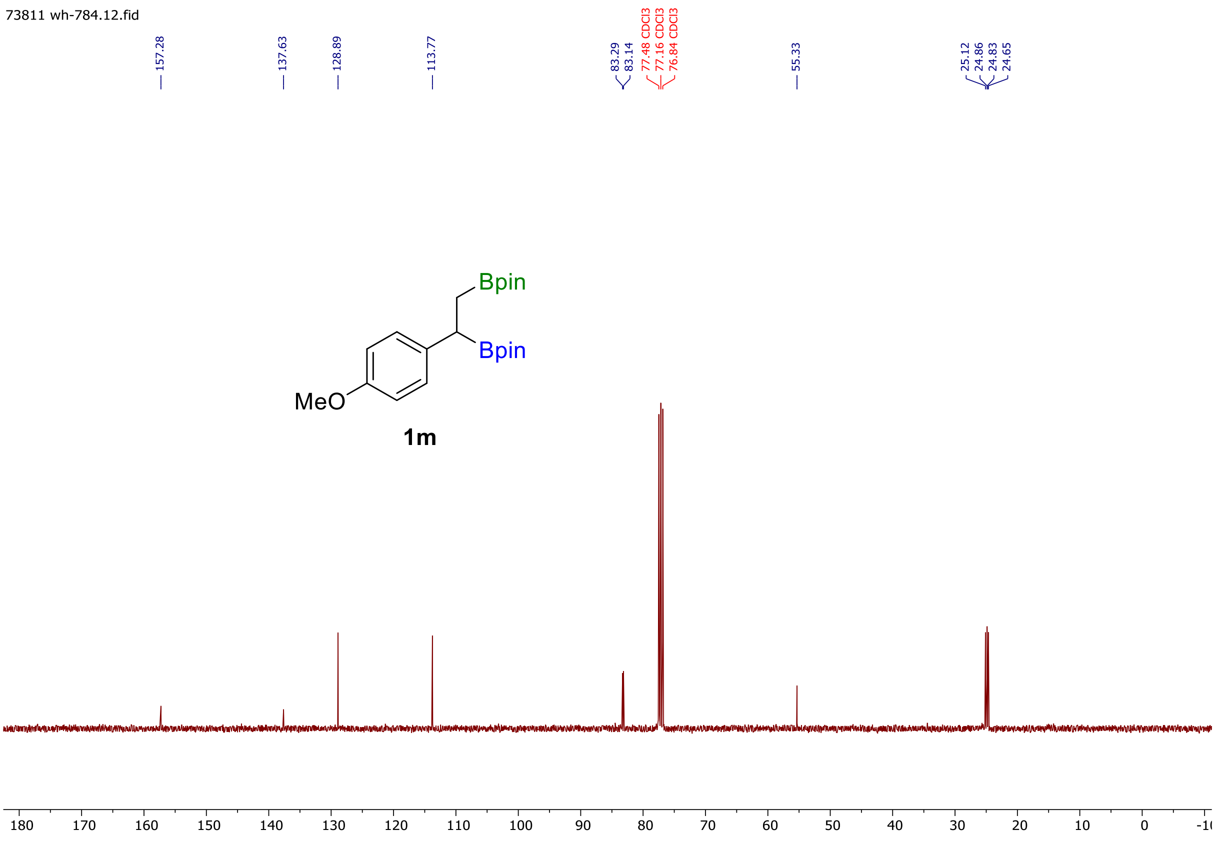

^{11}B NMR (128 MHz, $CDCl_3$) of **1m**

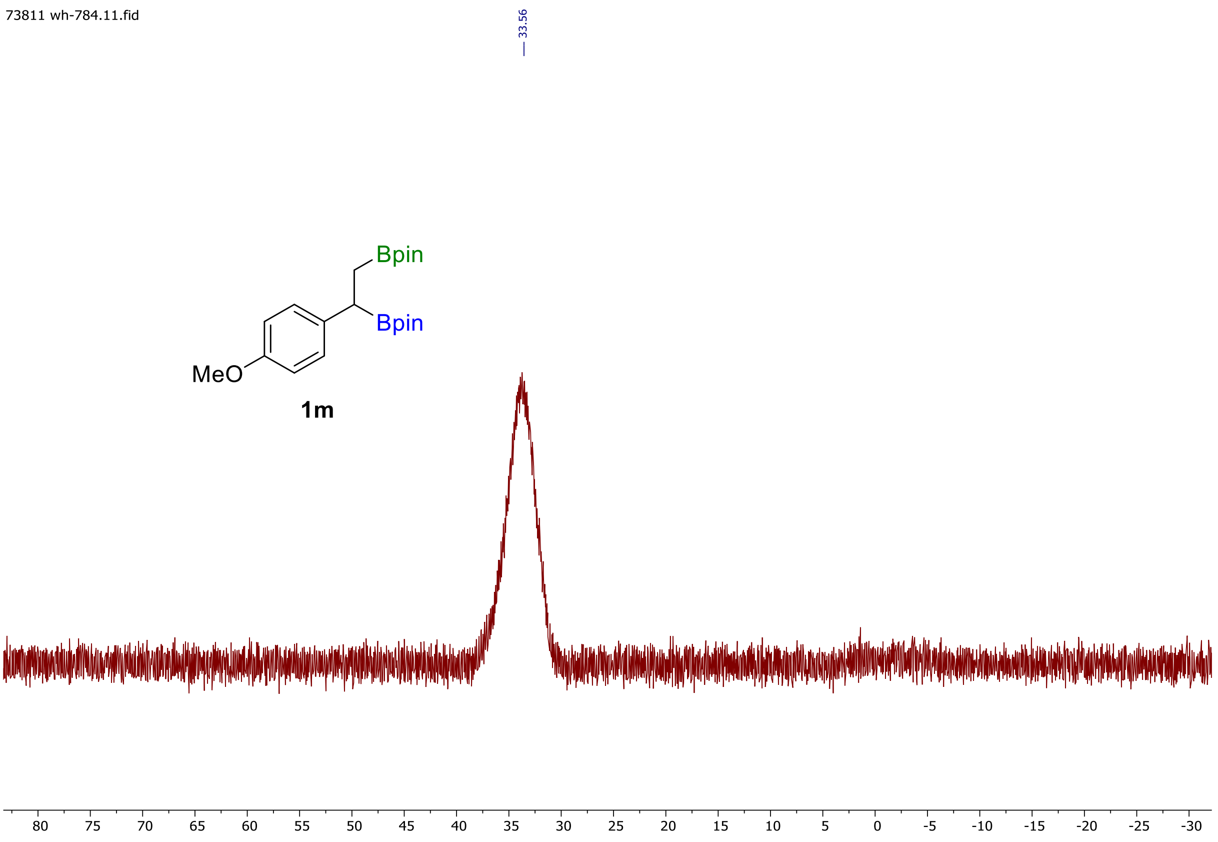

^{1}H NMR (400 MHz, $CDCl_3$) of **1n** (*see procedure*)

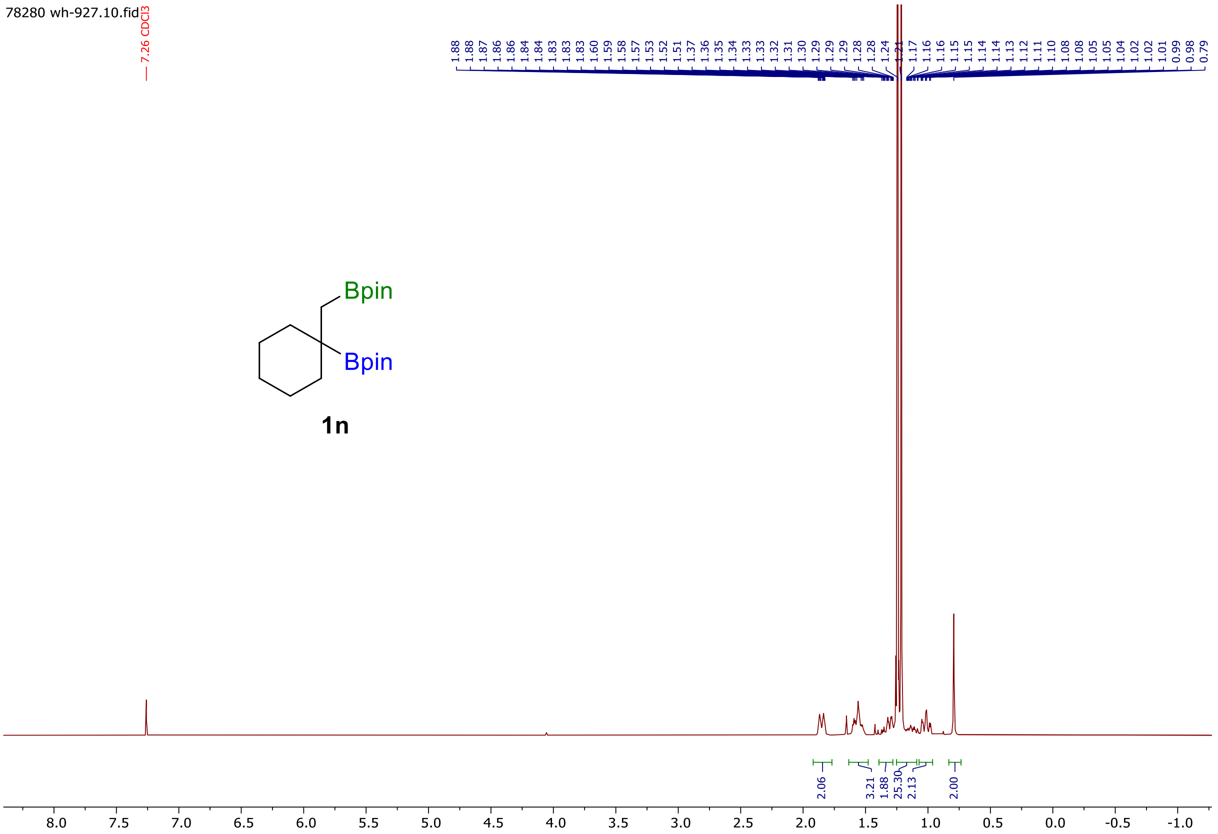

^{13}C NMR (101 MHz, $CDCl_3$) of **1n**

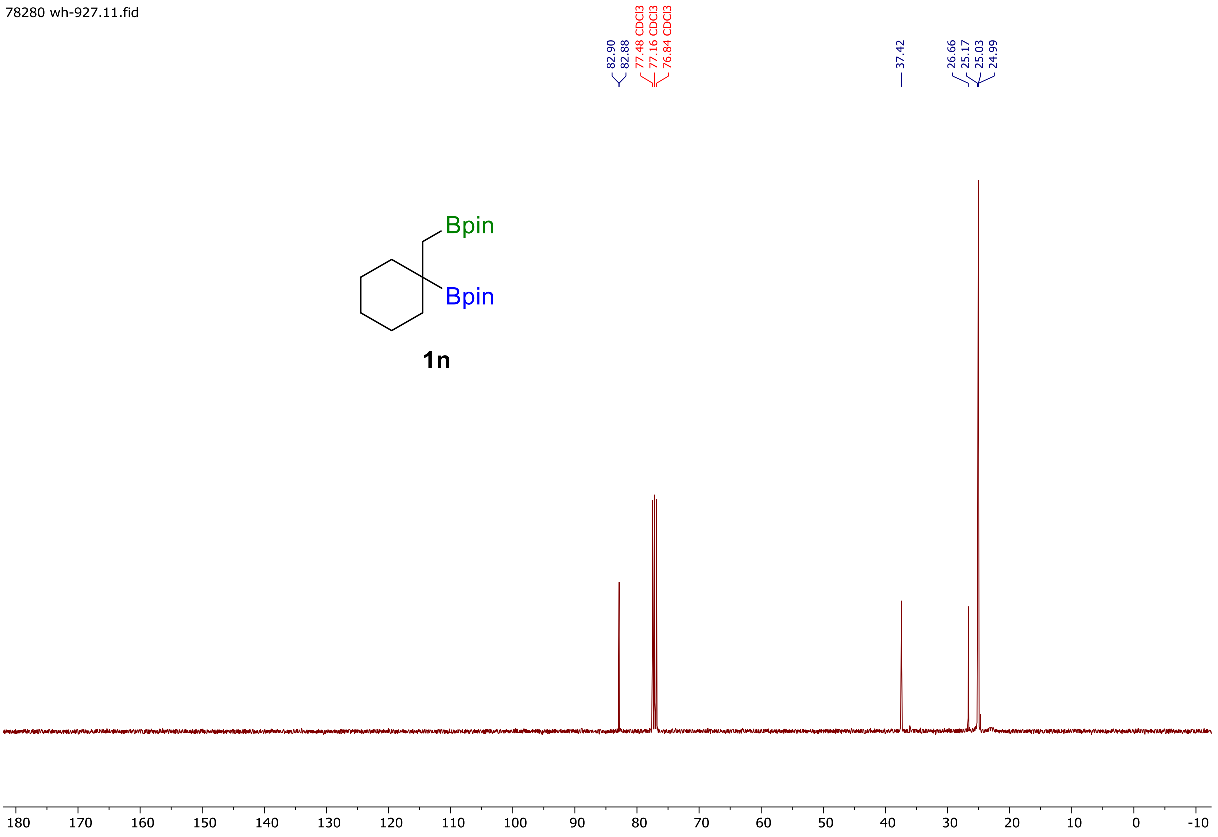

^{11}B NMR (128 MHz, $CDCl_3$) of **1n**

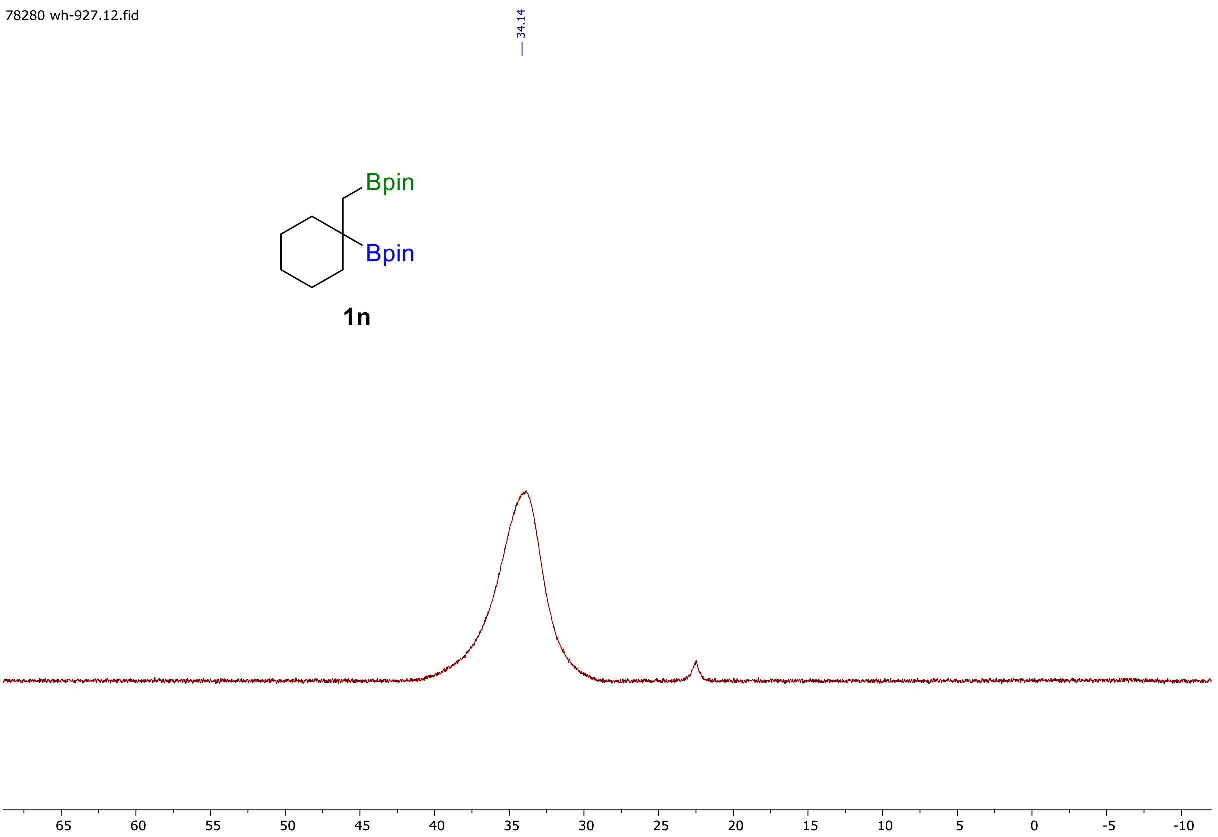

^{1}H NMR (400 MHz, $CDCl_3$) of **1o** (*see procedure*)

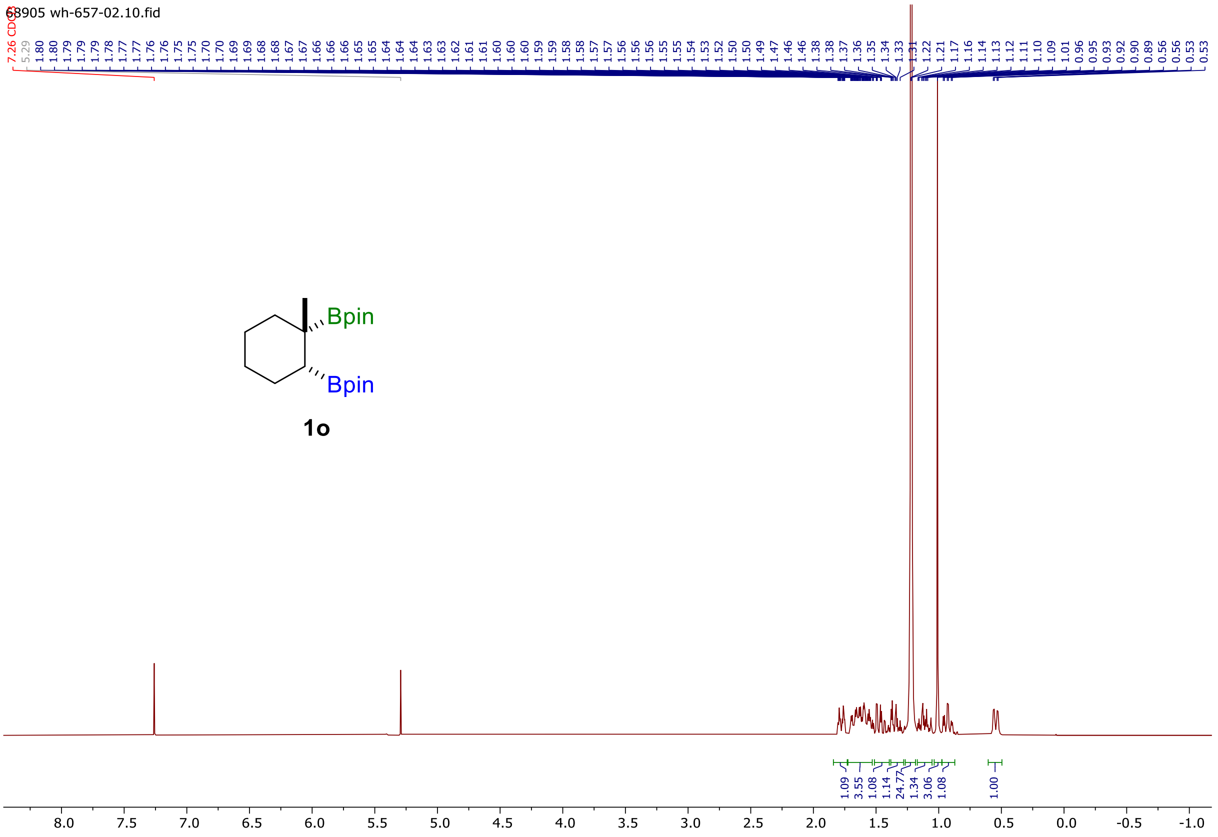

^{13}C NMR (101 MHz, $CDCl_3$) of **1o**

68905 wh-657-02.11.fid

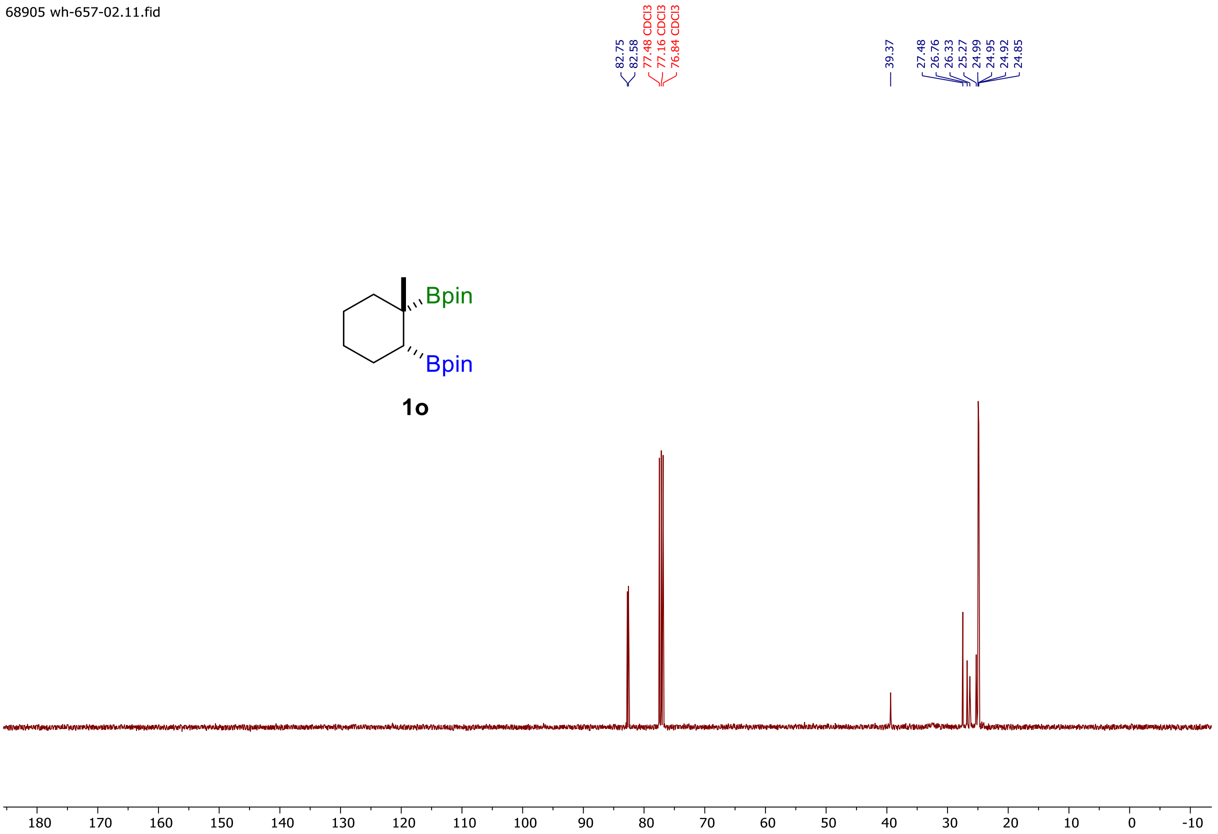

^{11}B NMR (128 MHz, $CDCl_3$) of **1o**

68905 wh-657-02.12.fid

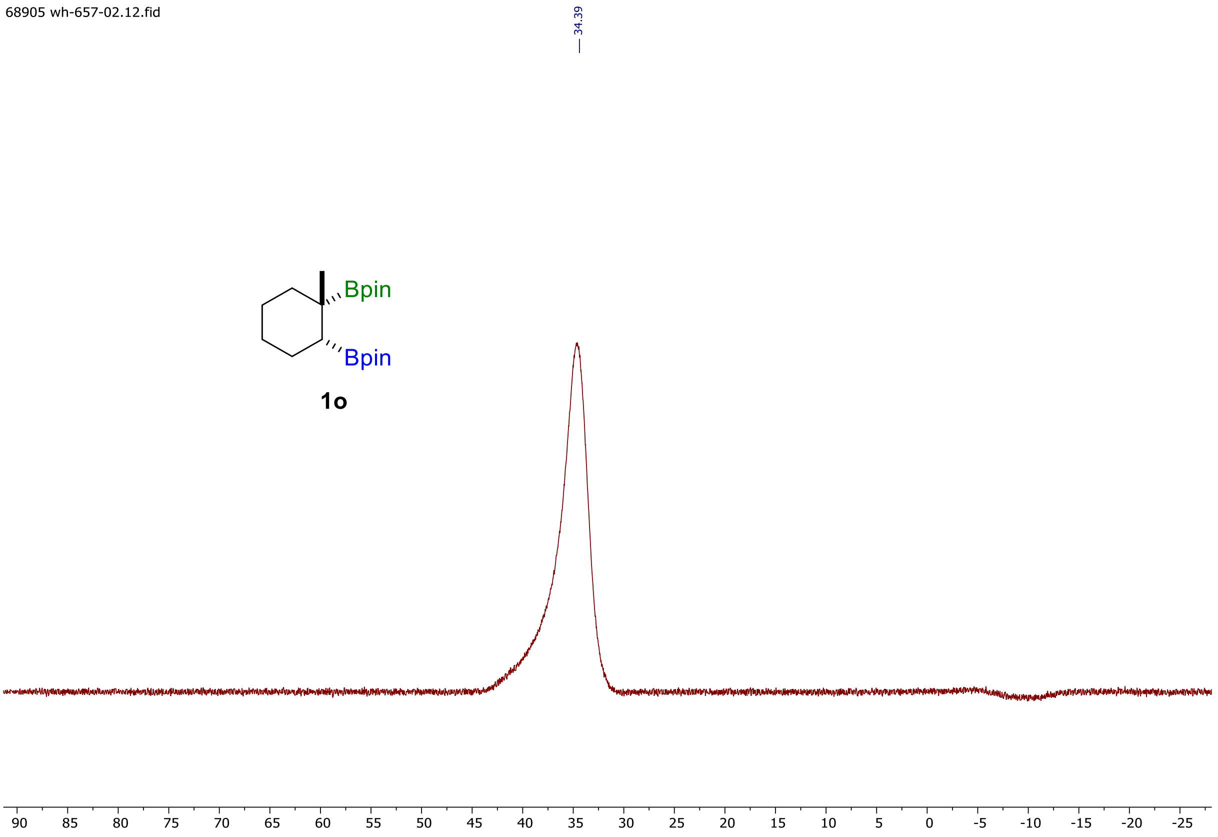

^{1}H NMR (400 MHz, $CDCl_3$) of **1p** (*see procedure*)

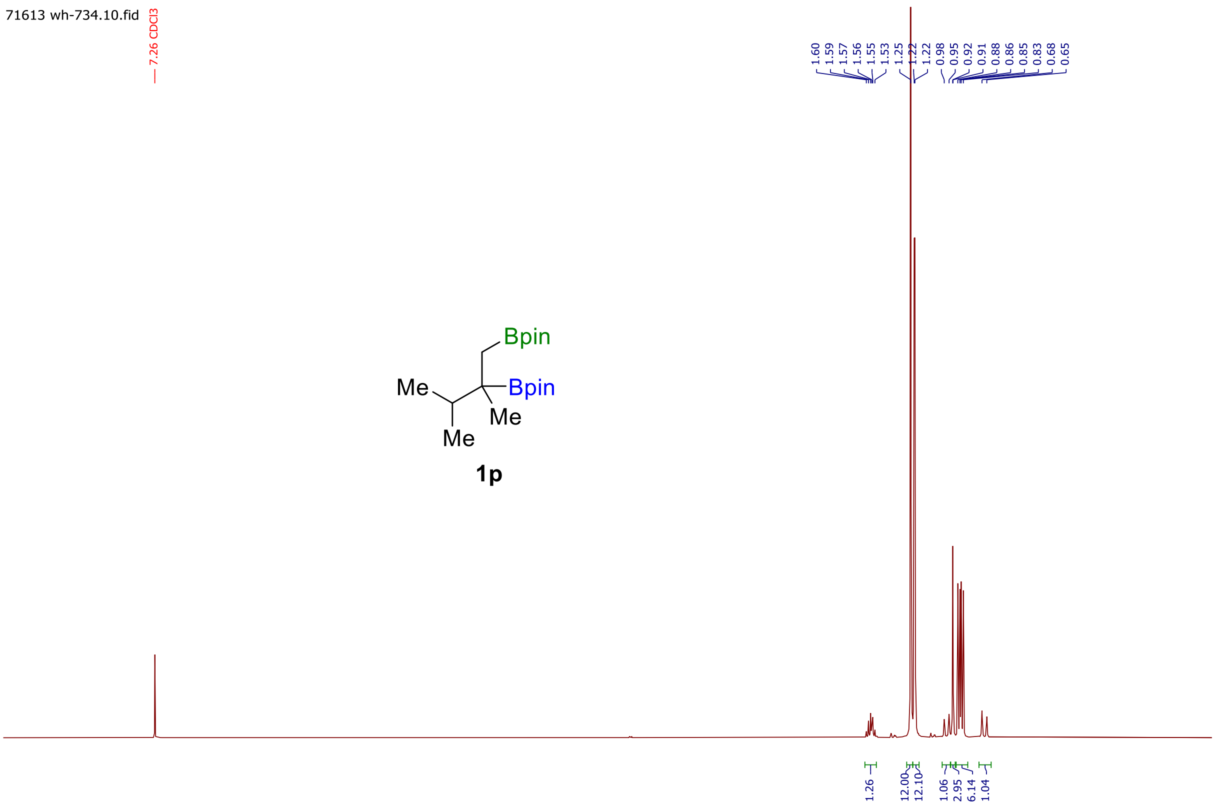

^{13}C NMR (101 MHz, $CDCl_3$) of **1p**

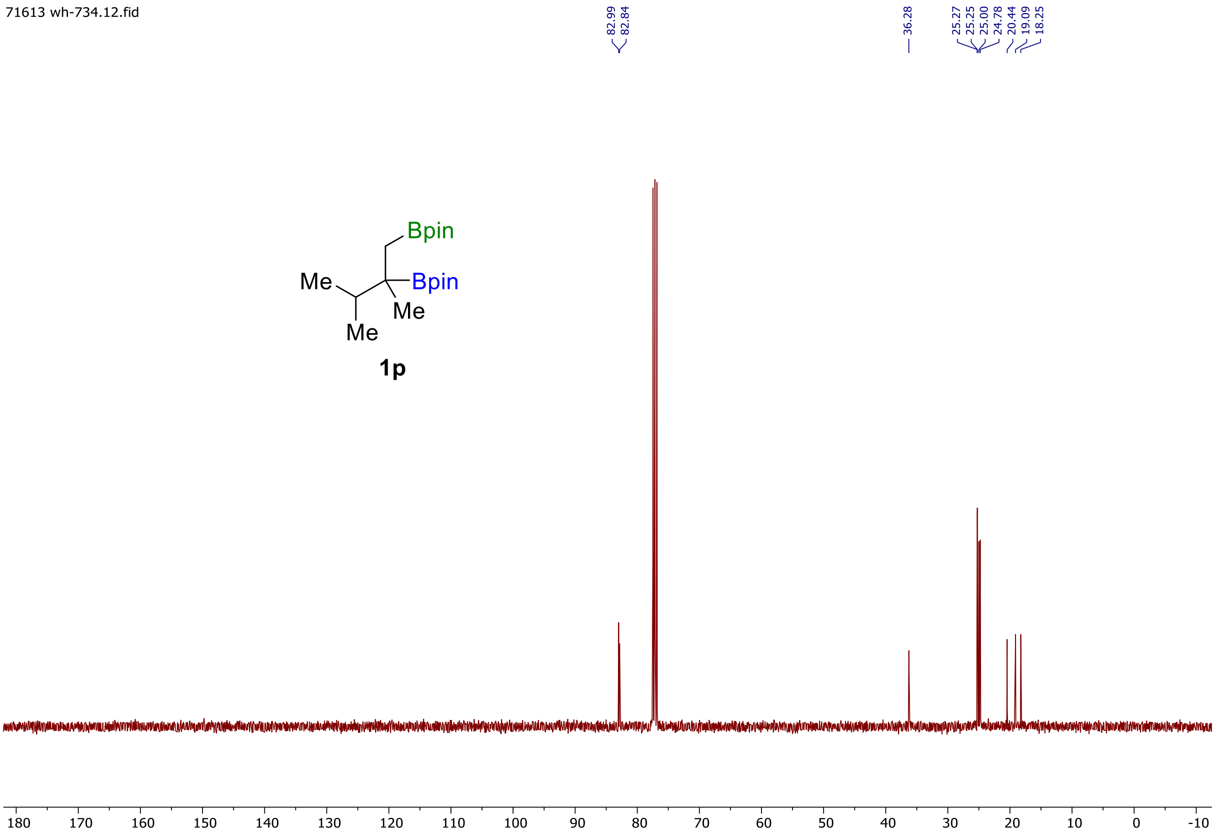

^{11}B NMR (128 MHz, $CDCl_3$) of **1p**

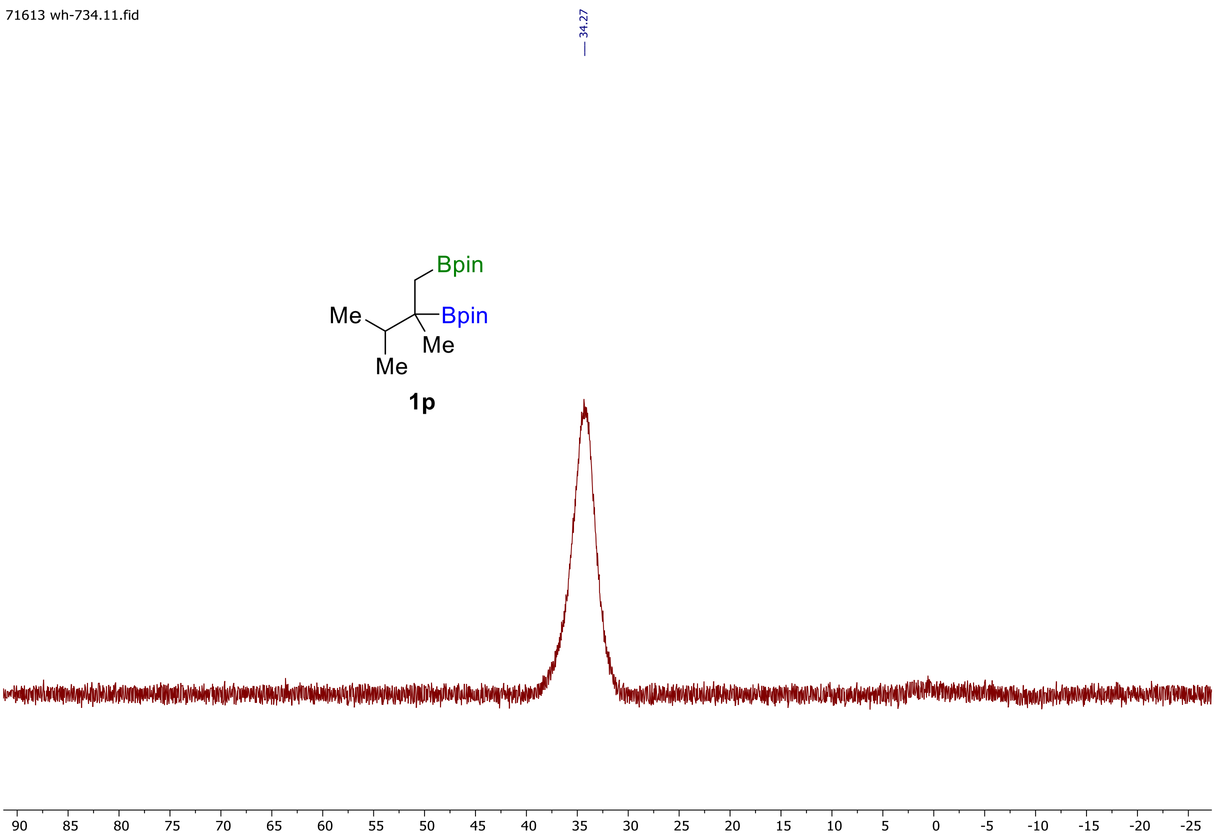

^{1}H NMR (400 MHz, $CDCl_3$) of **1q** (*see procedure*)

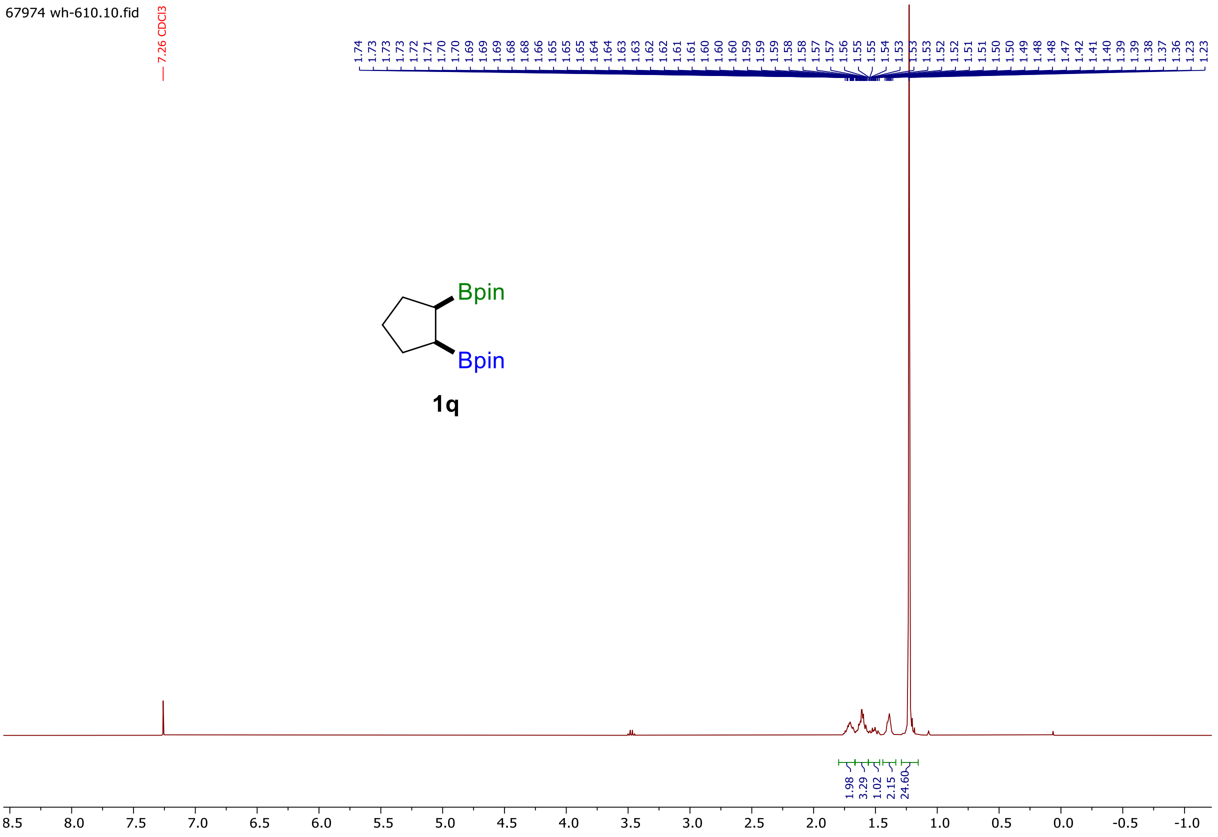

^{13}C NMR (101 MHz, $CDCl_3$) of **1q**

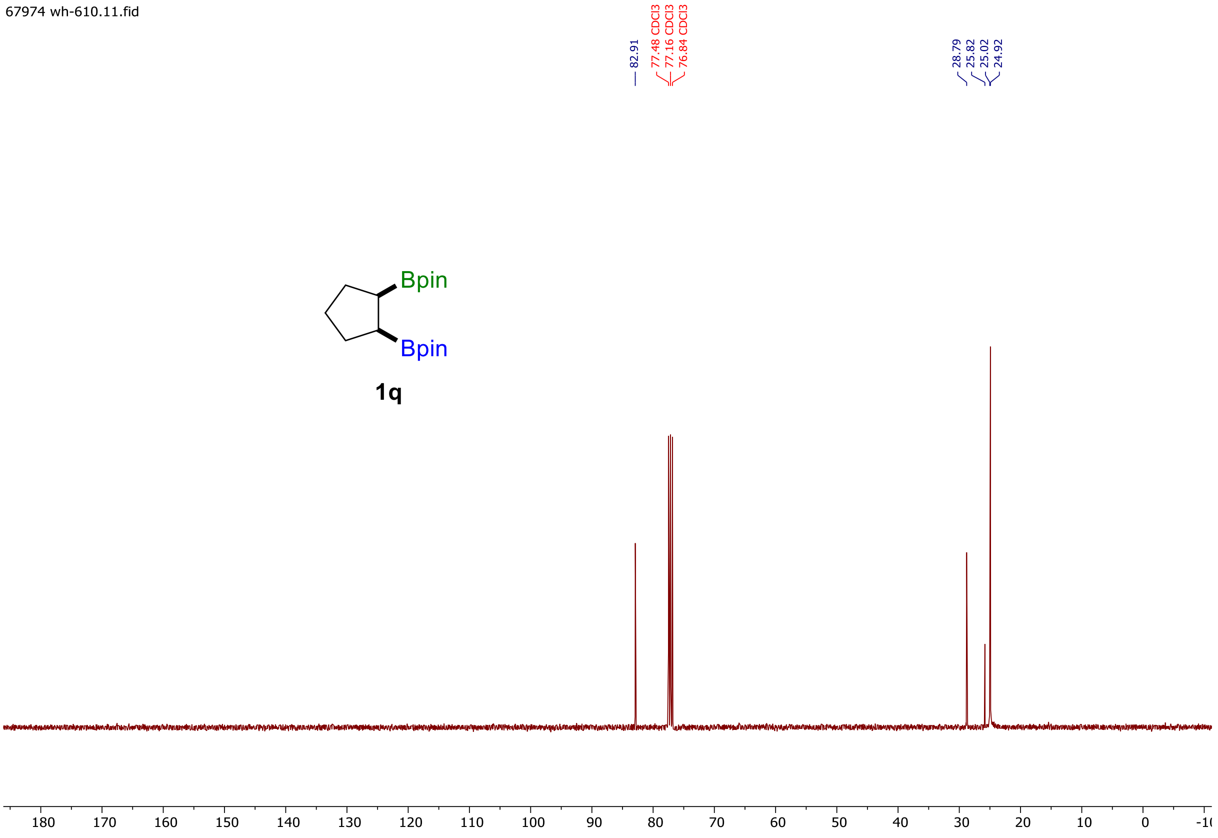

^{11}B NMR (128 MHz, $CDCl_3$) of **1q**

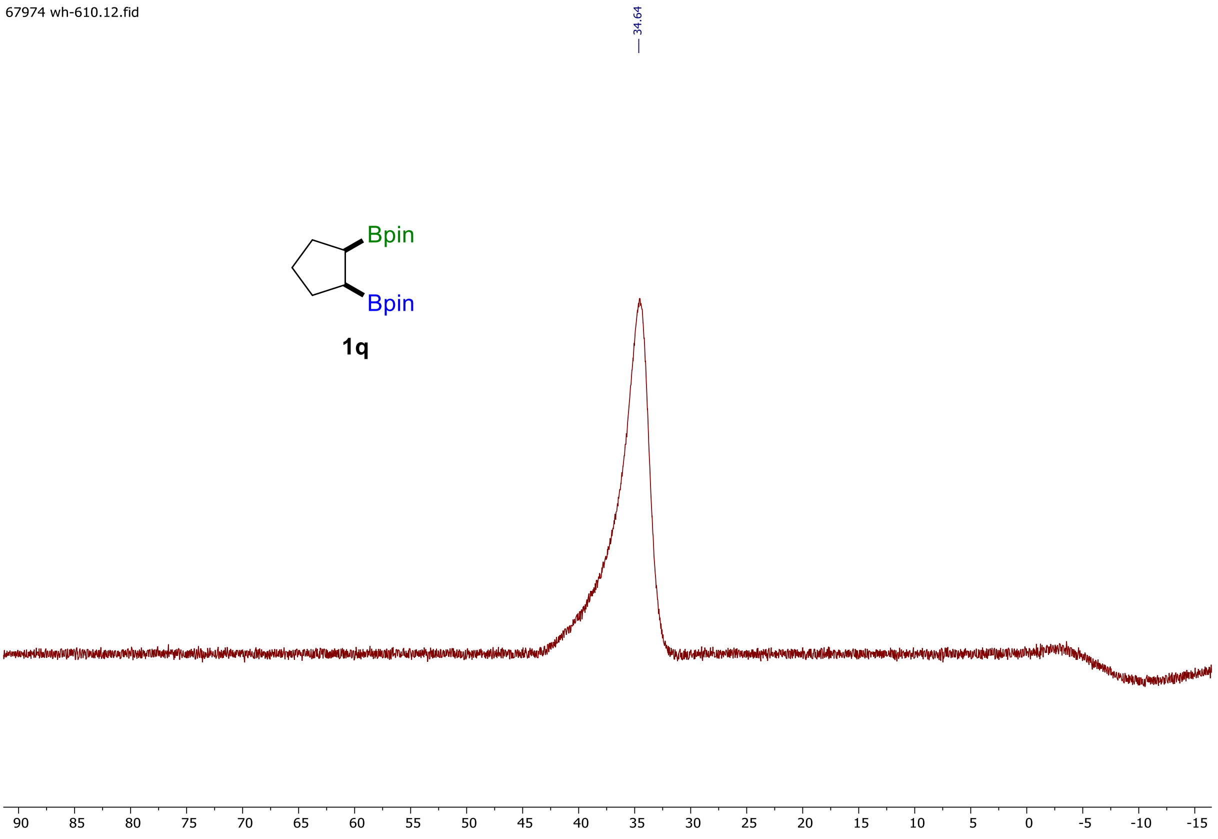

^{1}H NMR (400 MHz, $CDCl_3$) of **1r** (*see procedure*)

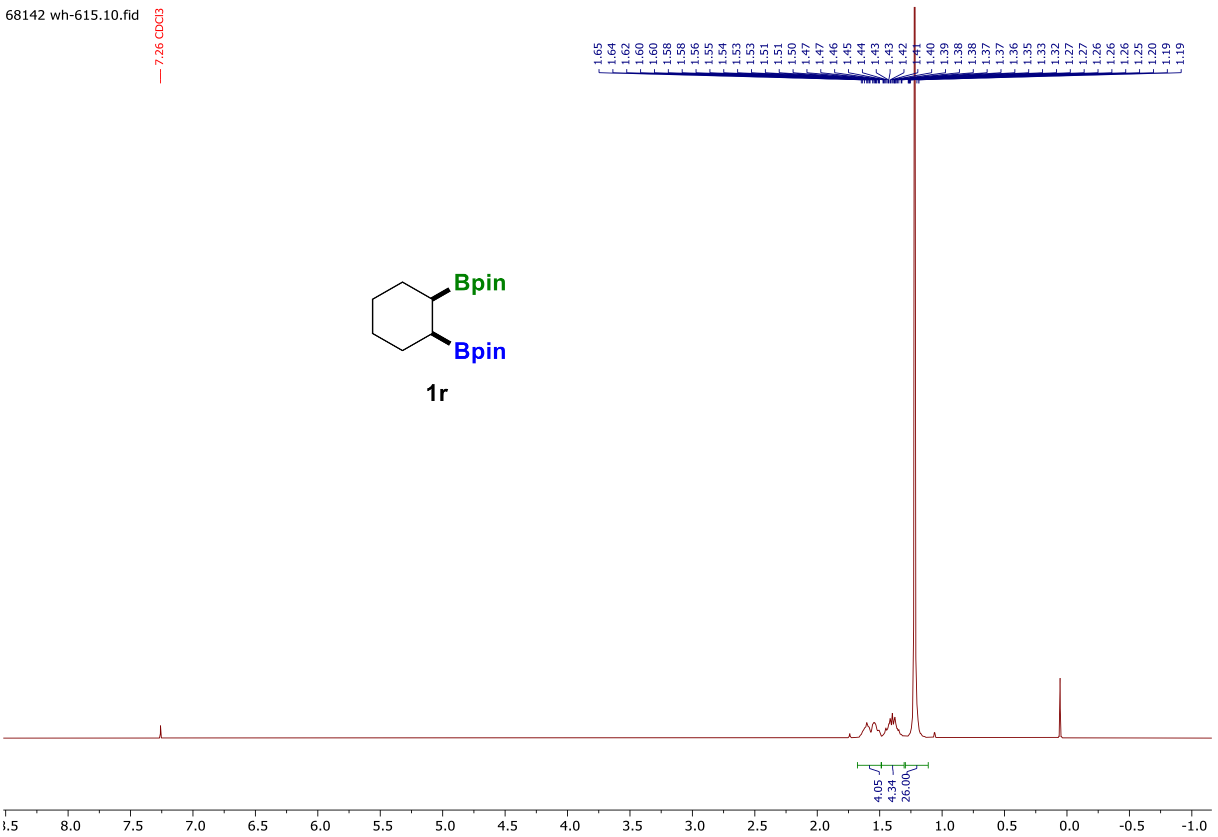

^{13}C NMR (101 MHz, $CDCl_3$) of **1r**

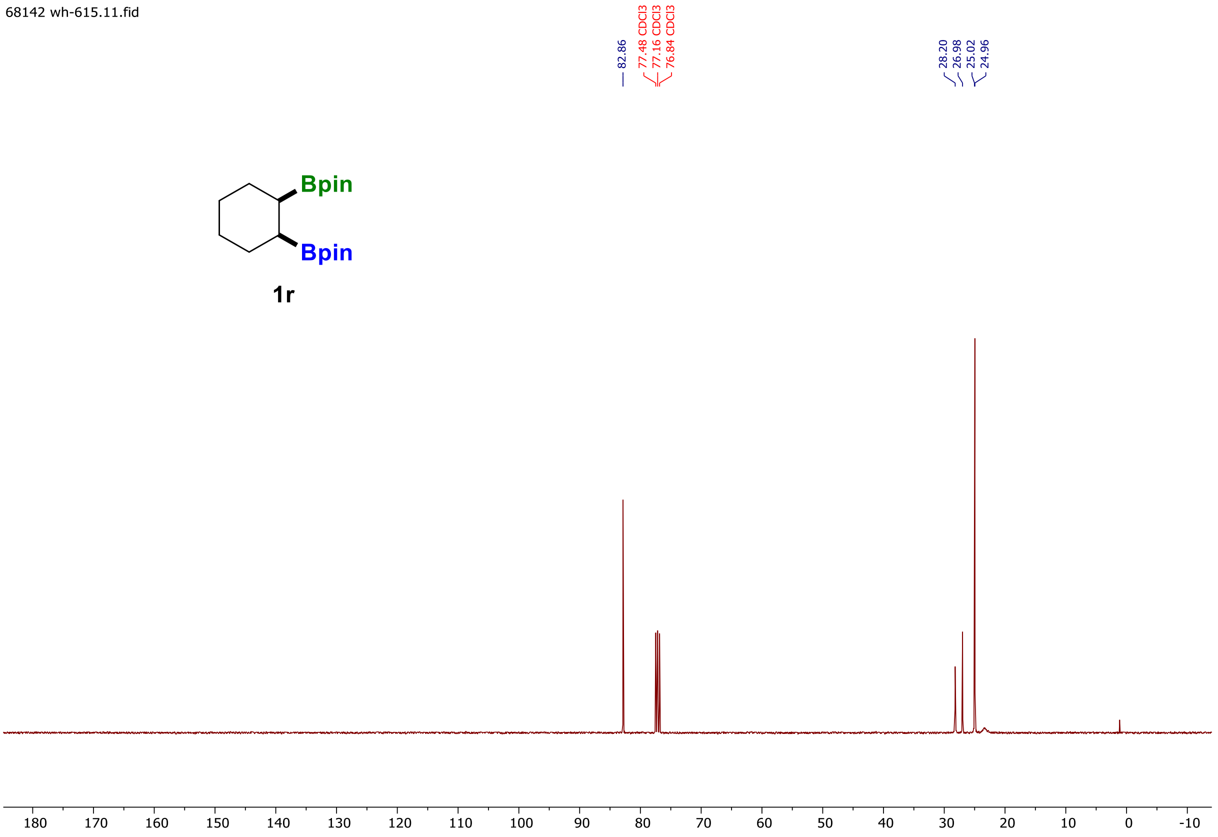

^{11}B NMR (128 MHz, $CDCl_3$) of **1r**

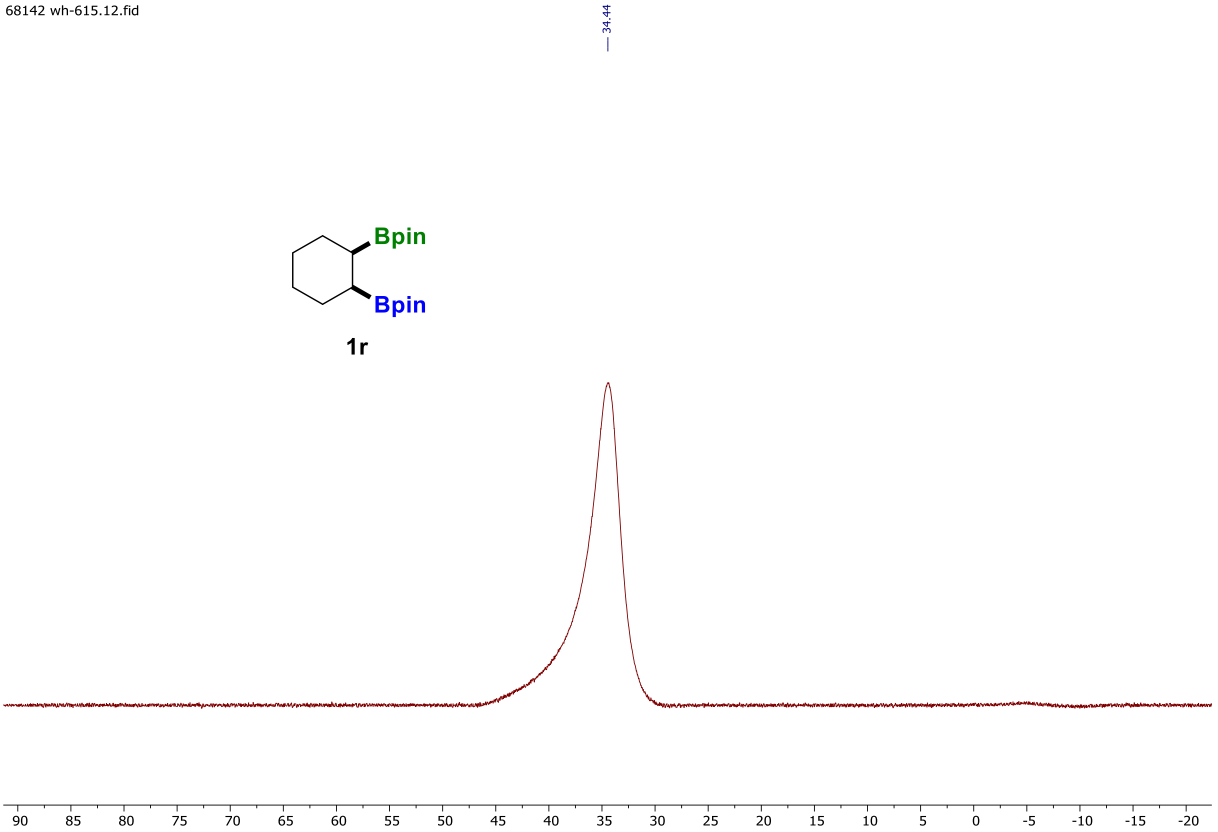

^{1}H NMR (400 MHz, $CDCl_3$) of **1t** (*see procedure*)

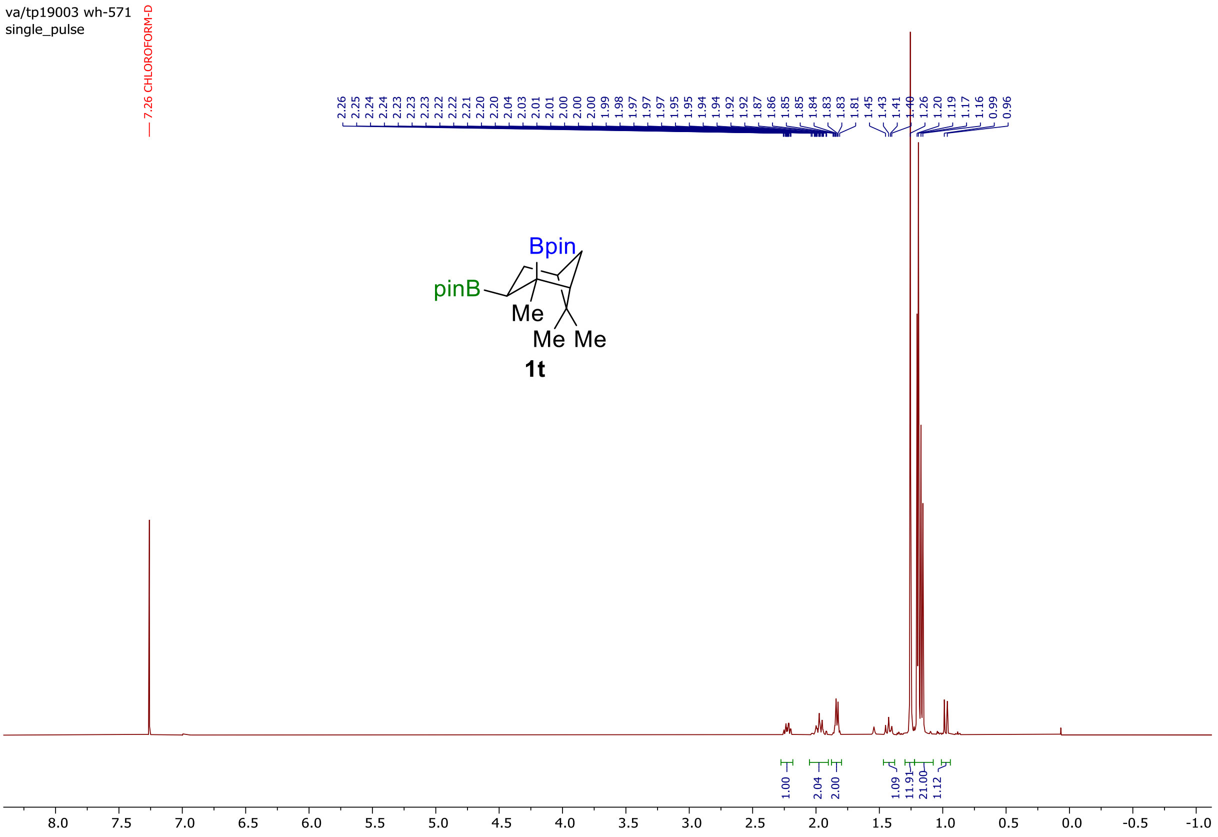

^{13}C NMR (101 MHz, $CDCl_3$) of **1t**

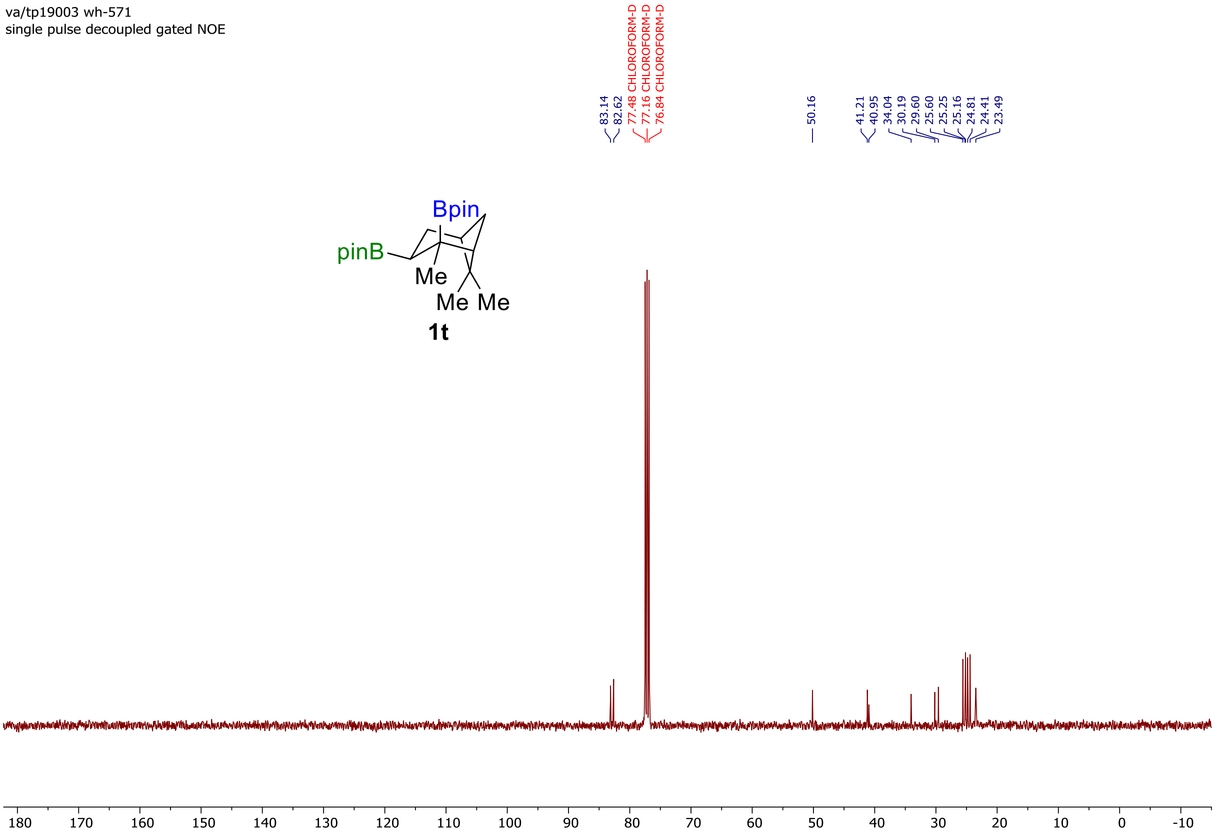

^{11}B NMR (128 MHz, $CDCl_3$) of **1t**

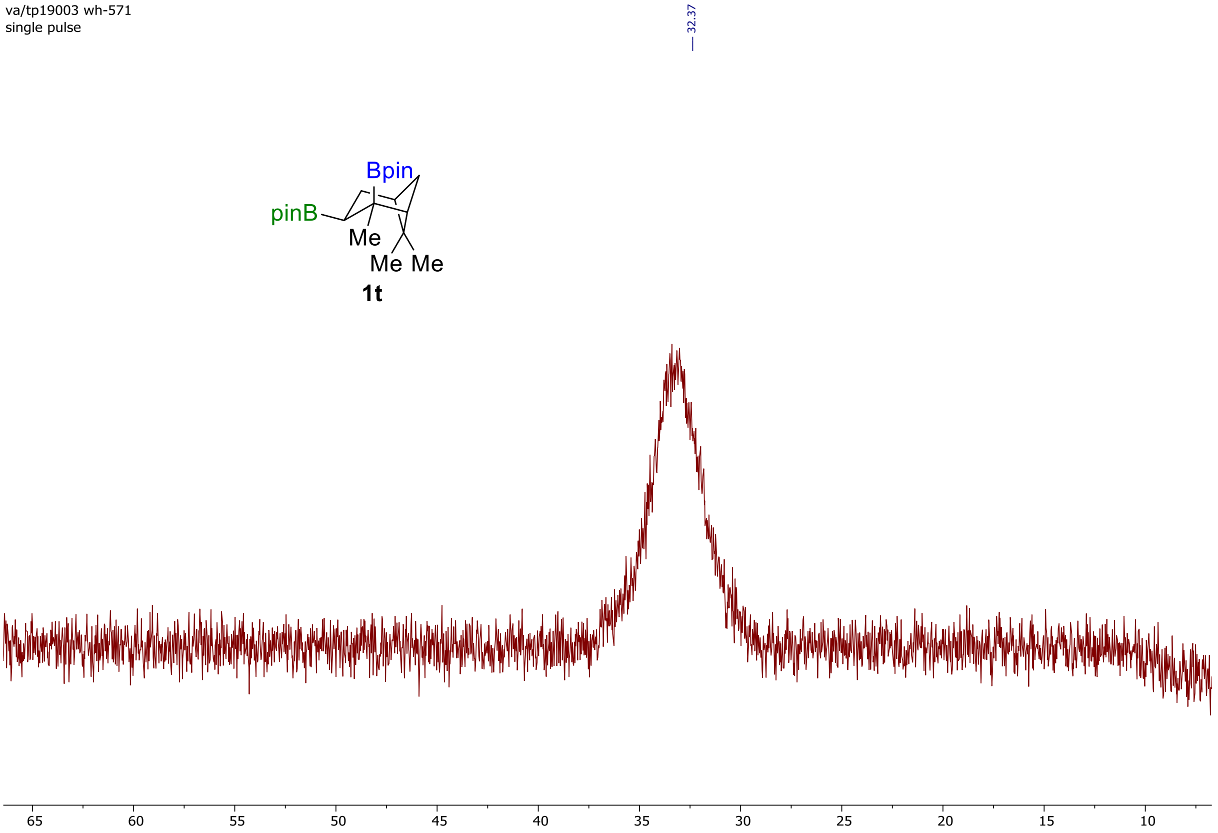

^{1}H NMR (400 MHz, $CDCl_3$) of **1u** (*see procedure*)

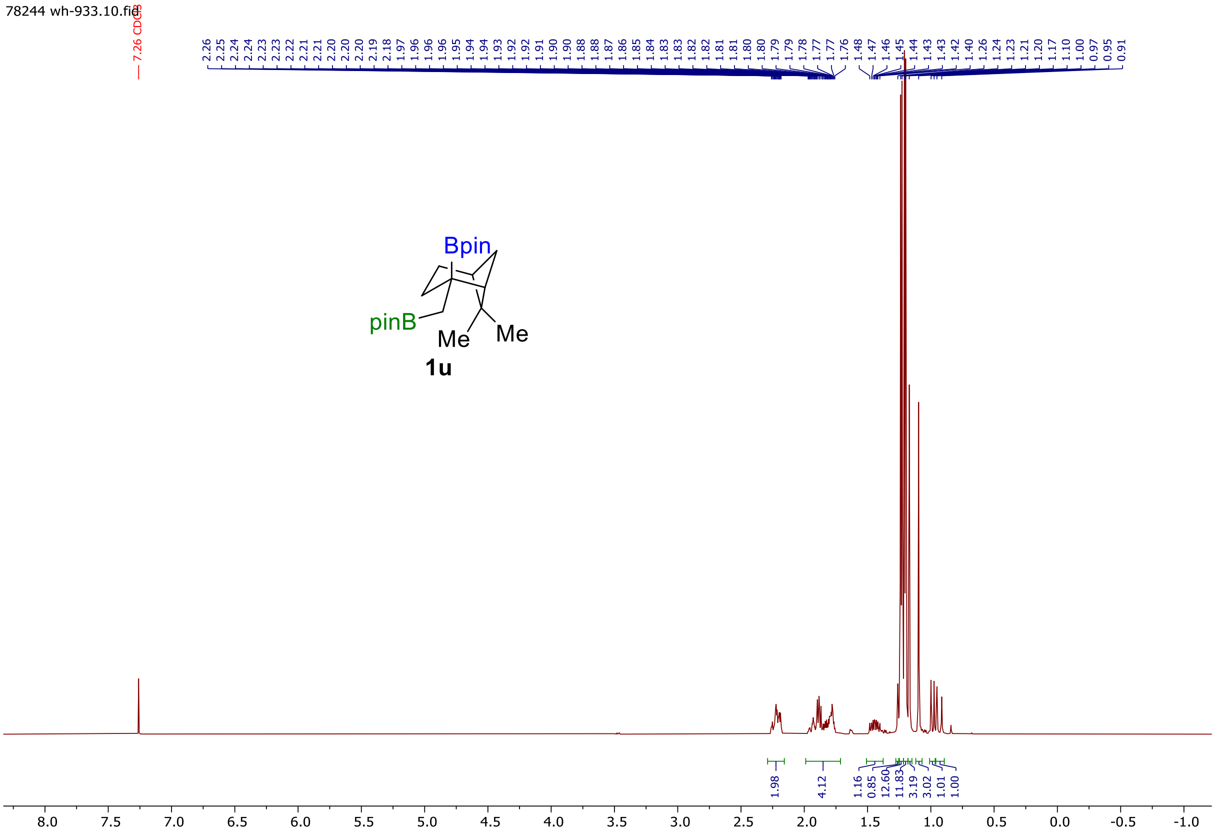

^{13}C NMR (101 MHz, $CDCl_3$) of **1u**

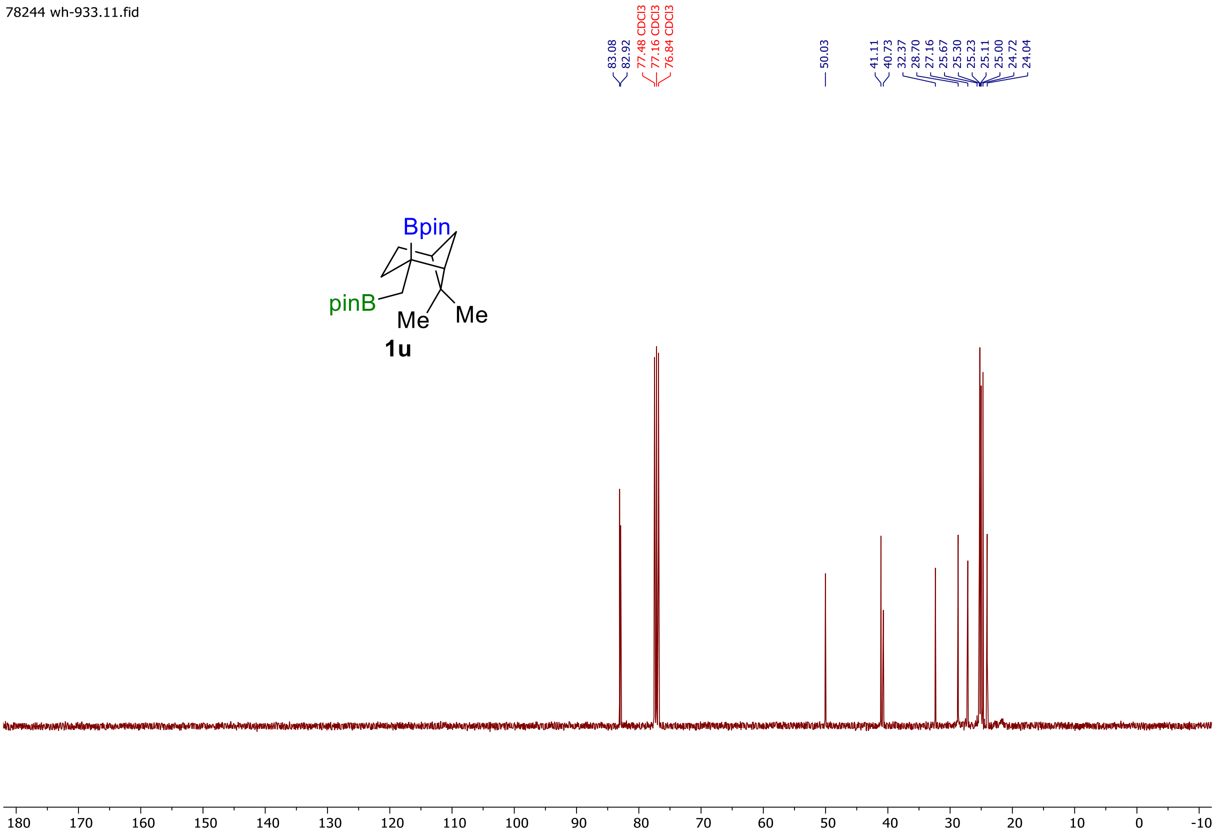

^{11}B NMR (128 MHz, $CDCl_3$) of **1u**

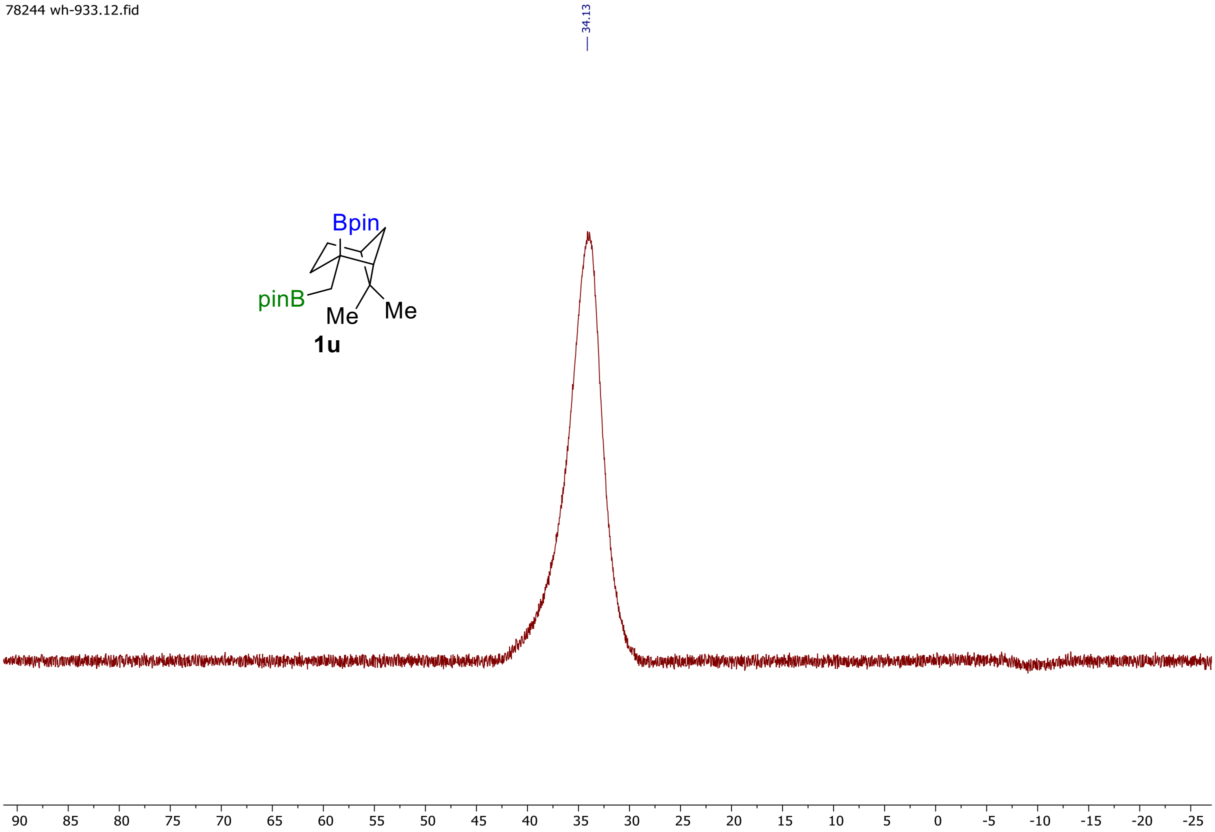

^{1}H NMR (400 MHz, $CDCl_3$) of **1v** (*see procedure*)

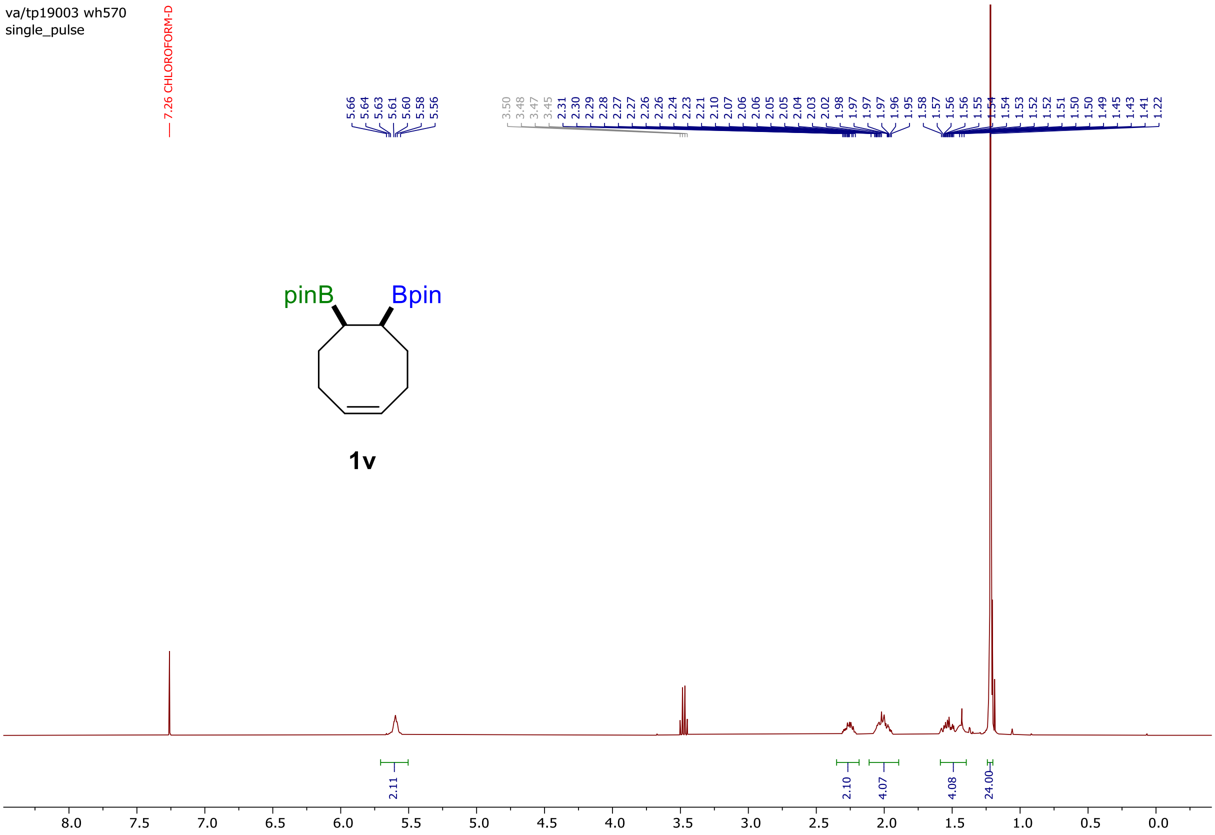

^{13}C NMR (101 MHz, $CDCl_3$) of **1v**

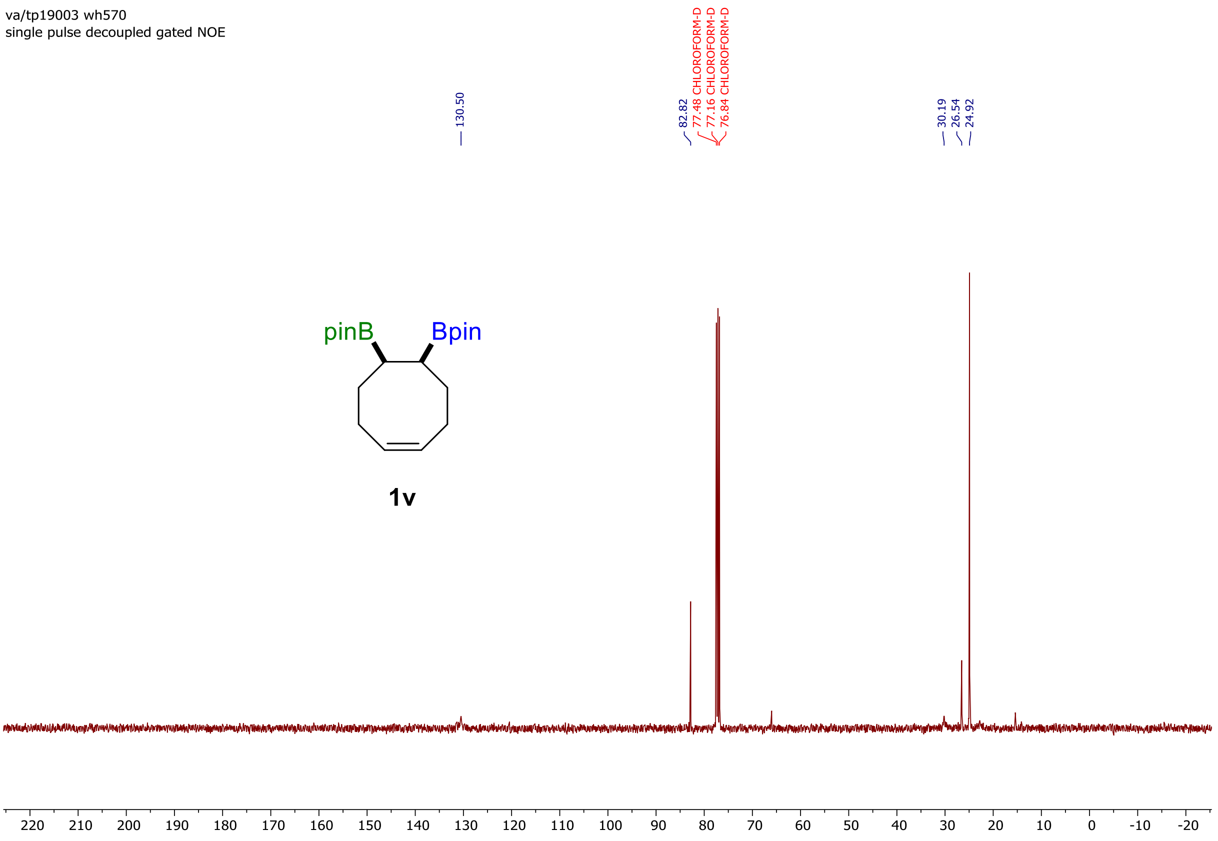

^{11}B NMR (128 MHz, $CDCl_3$) of **1v**

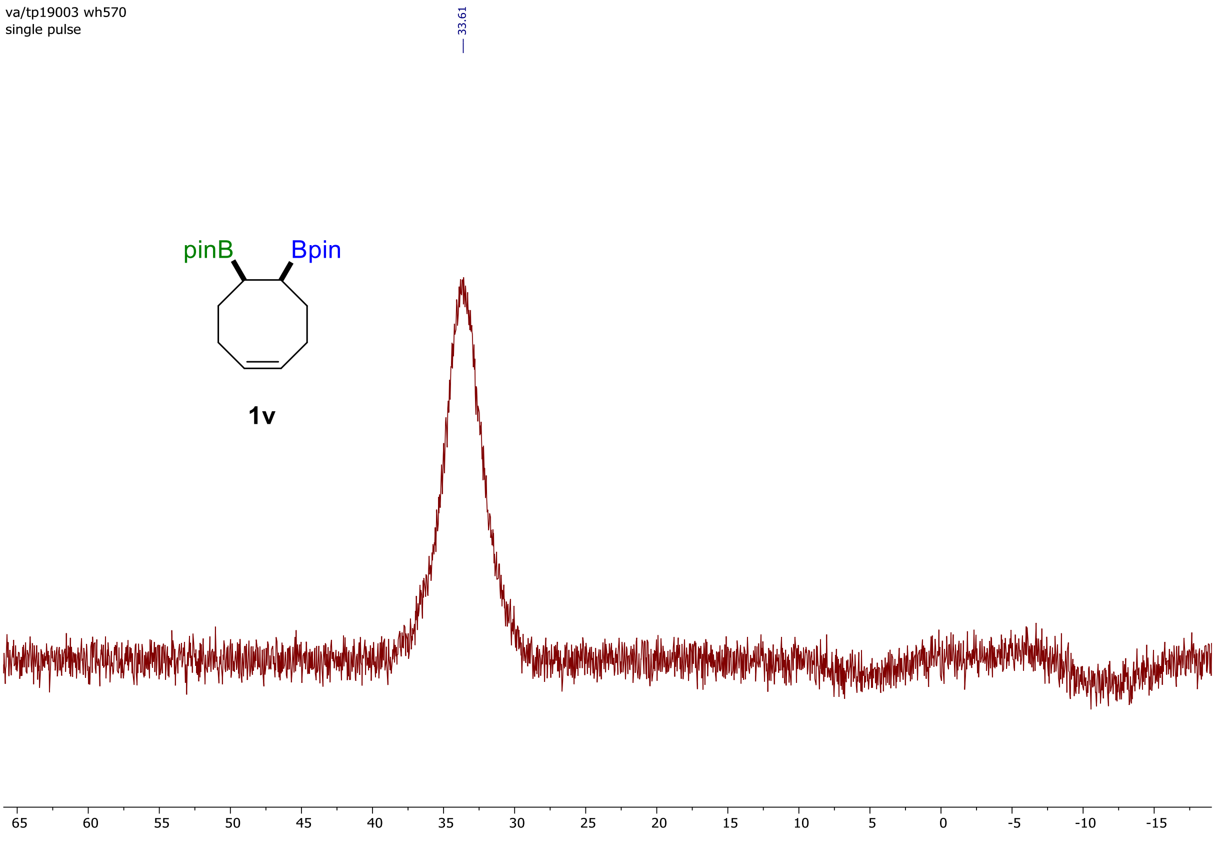

^{1}H NMR (400 MHz, $CDCl_3$) of **1w** (*see procedure*)

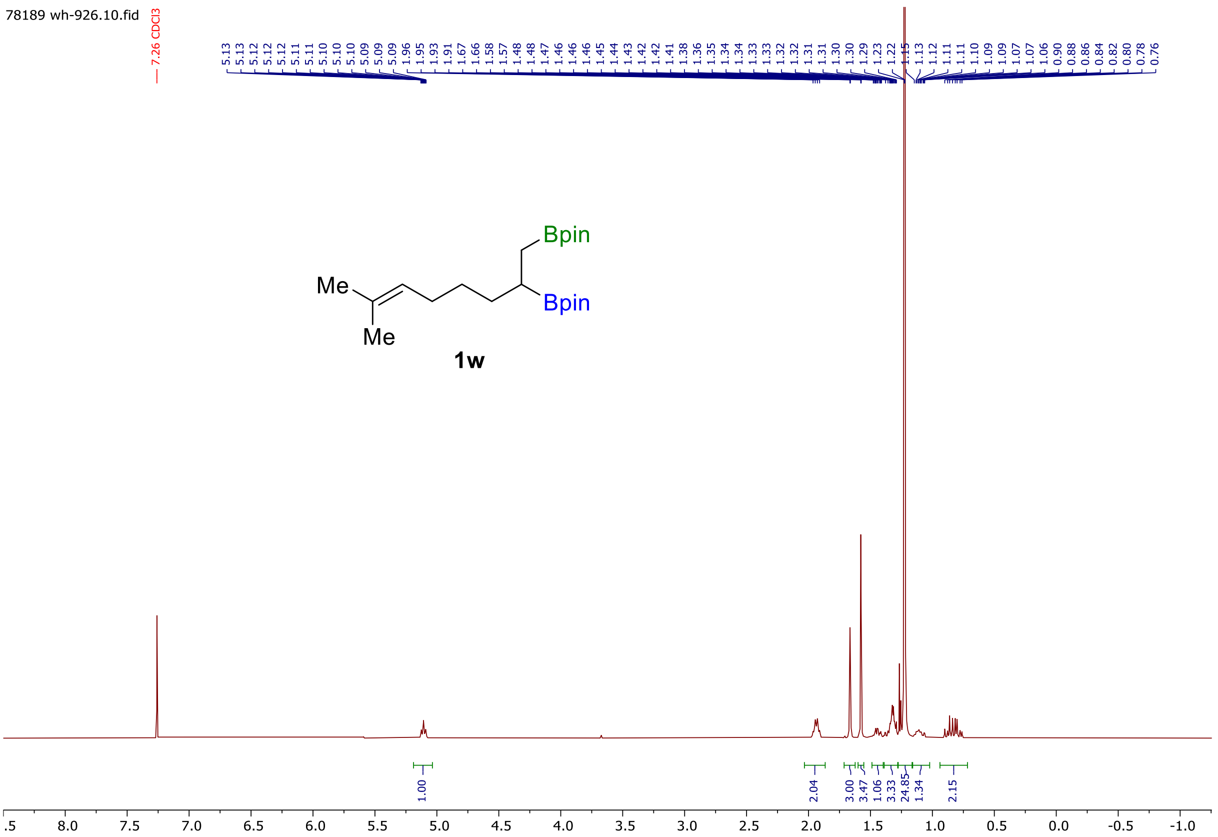

^{13}C NMR (101 MHz, $CDCl_3$) of **1w**

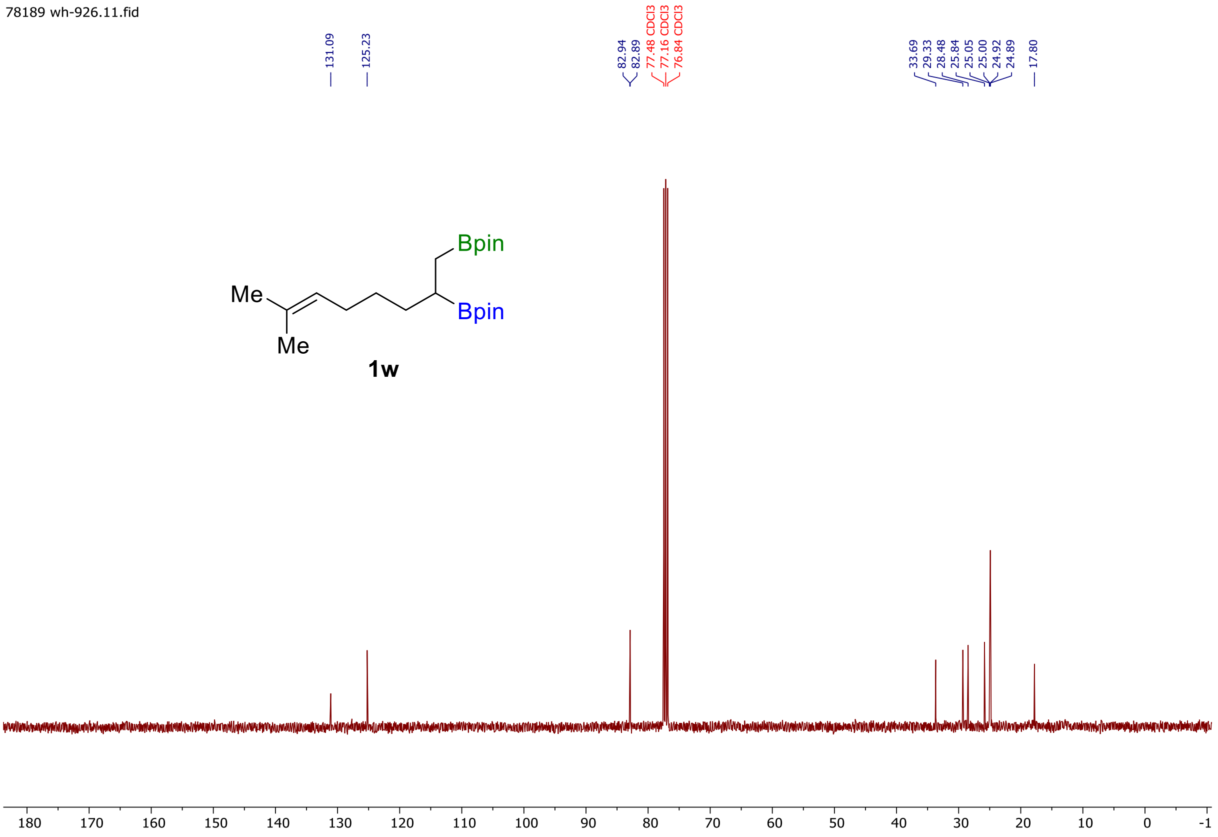

^{11}B NMR (128 MHz, $CDCl_3$) of **1w**

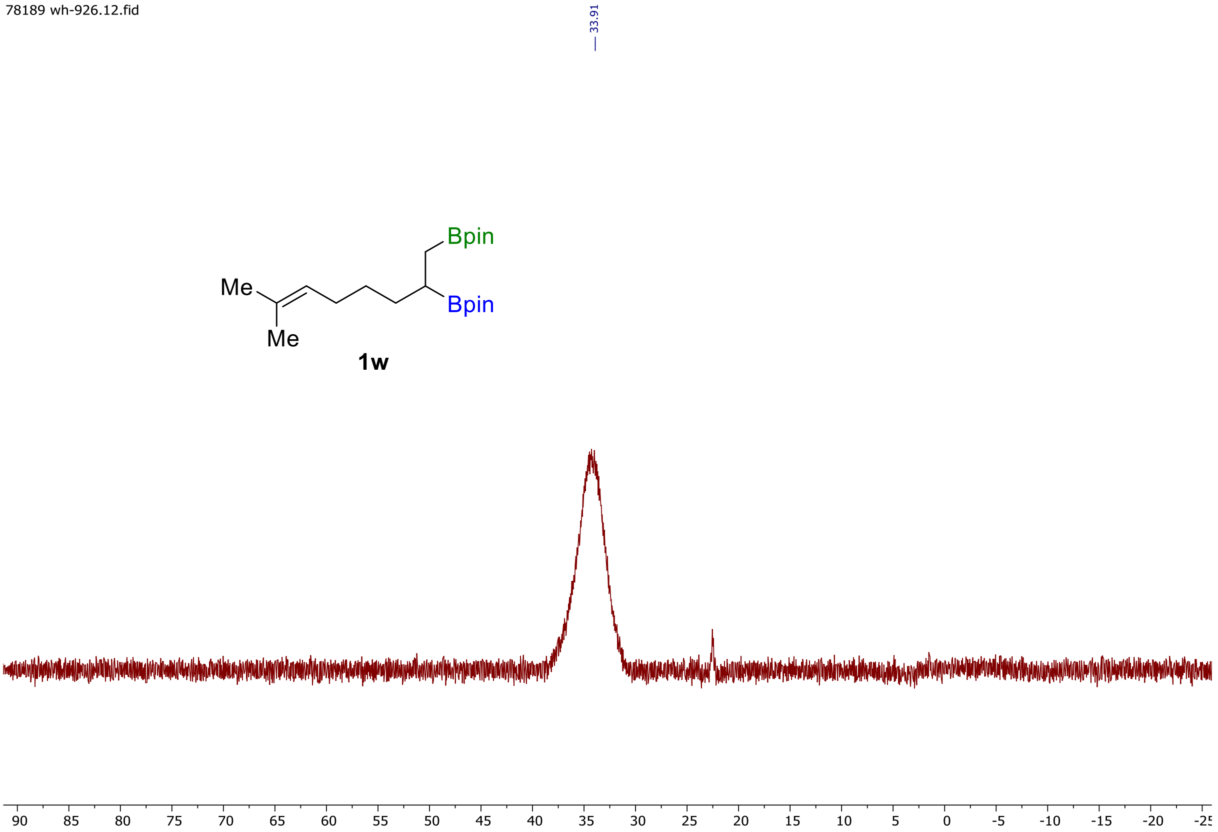

^{1}H NMR (400 MHz, $CDCl_3$) of **4c** (*see procedure*)

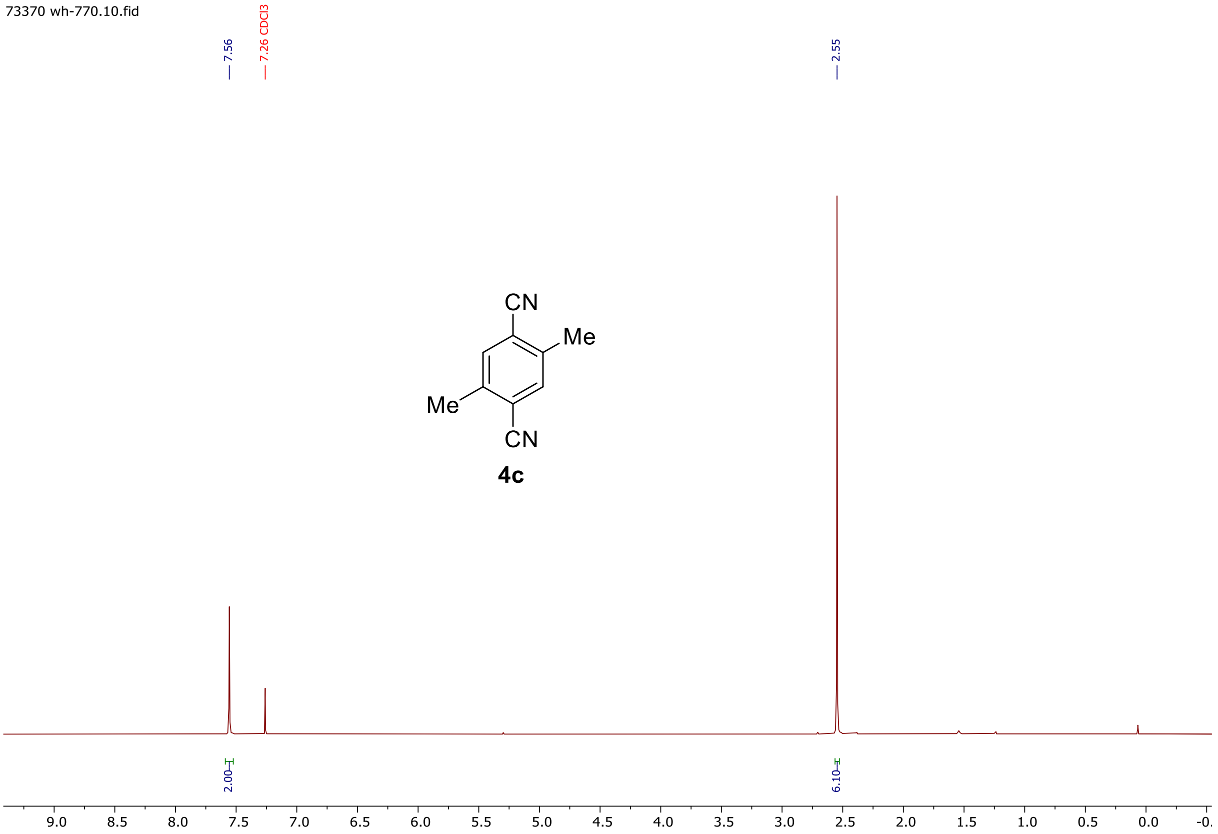

^{13}C NMR (101 MHz, $CDCl_3$) of **4c**

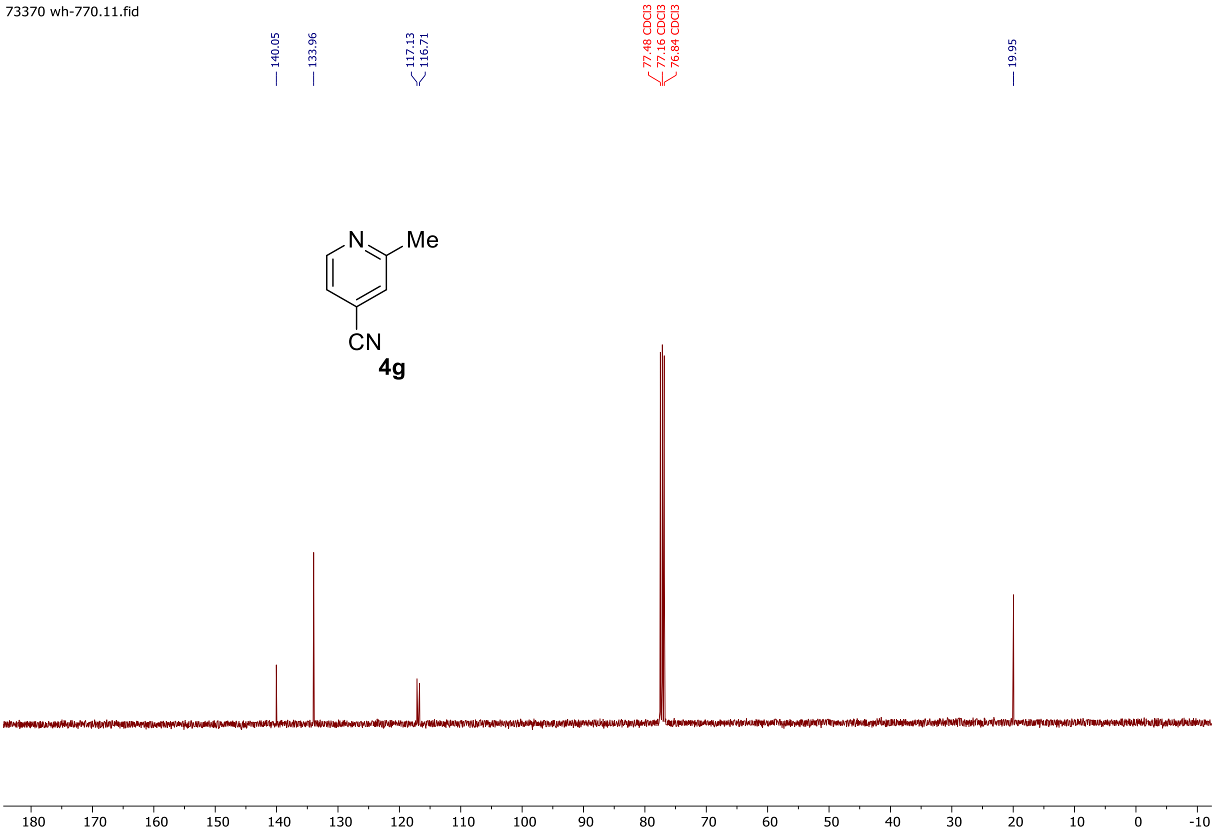

^{1}H NMR (400 MHz, $CDCl_3$) of **4g** (*see procedure*)

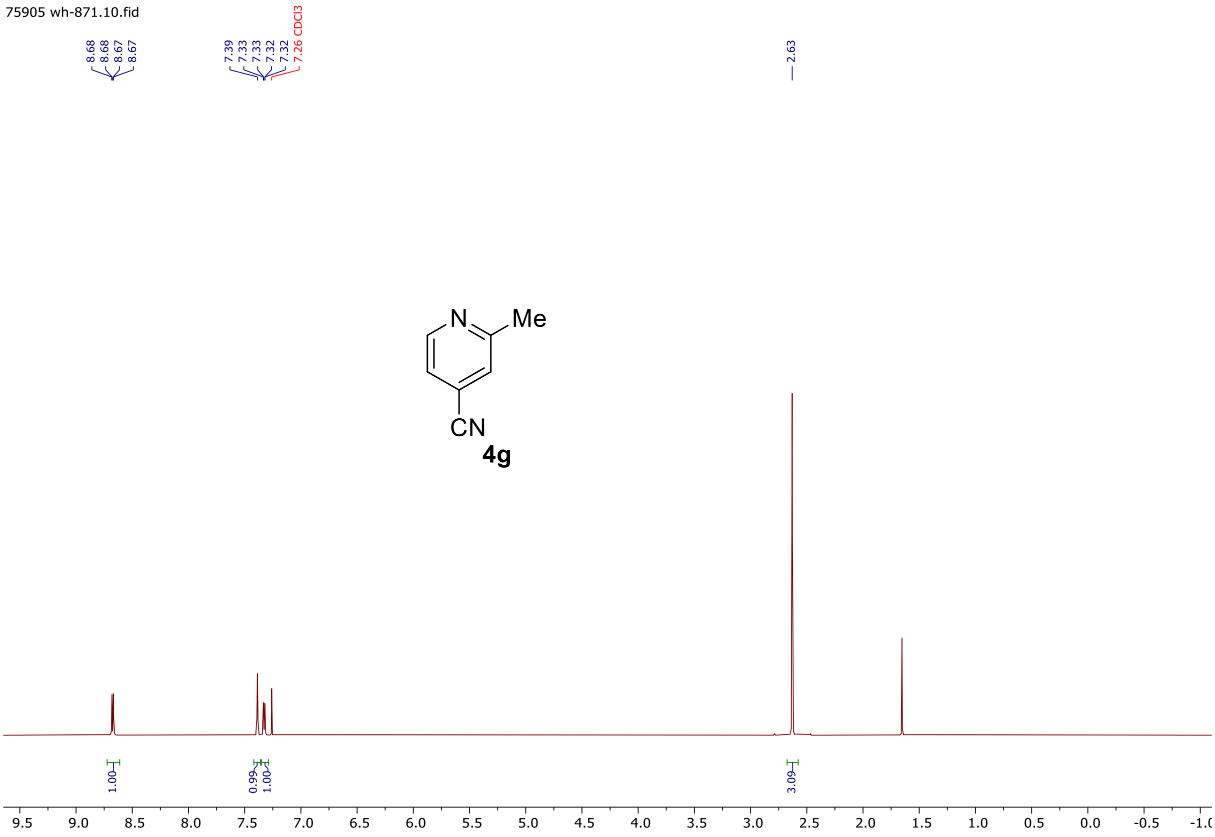

^{13}C NMR (101 MHz, $CDCl_3$) of **4g**

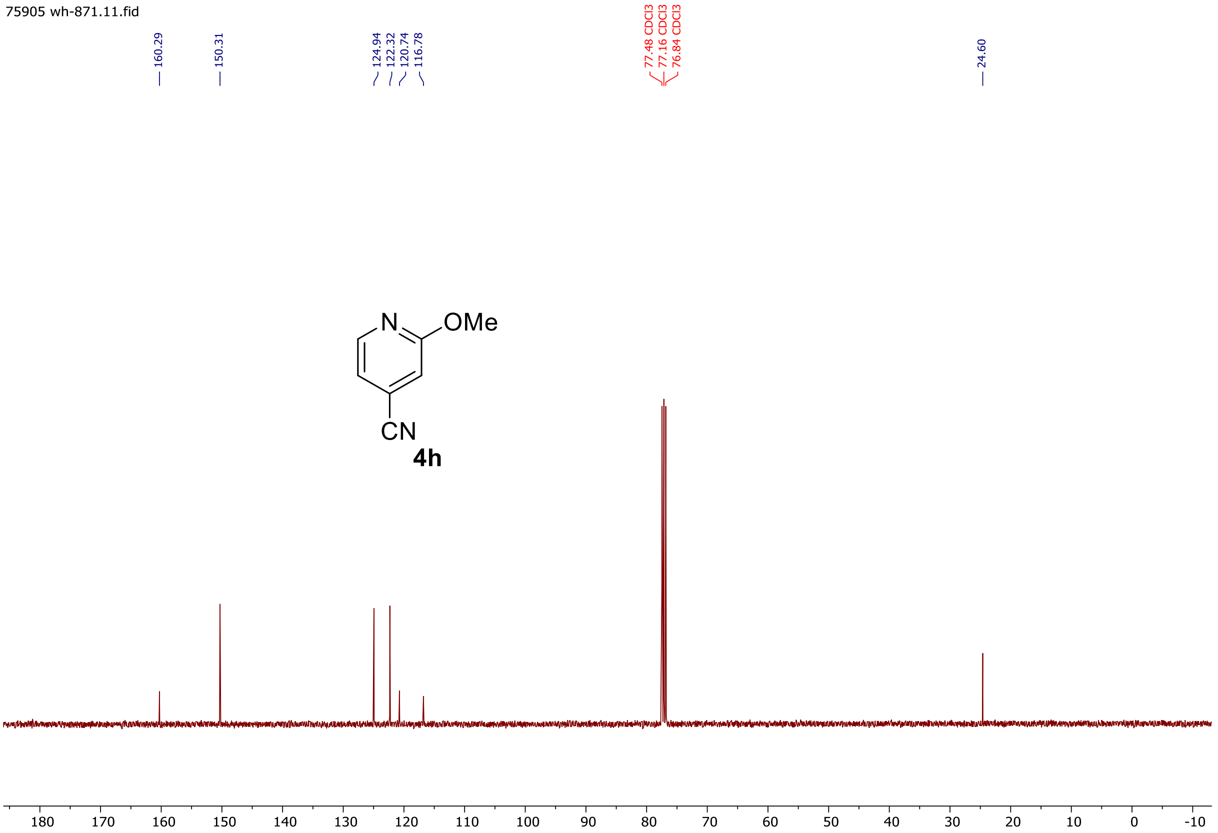

^{1}H NMR (400 MHz, $CDCl_3$) of **4h** (see procedure)

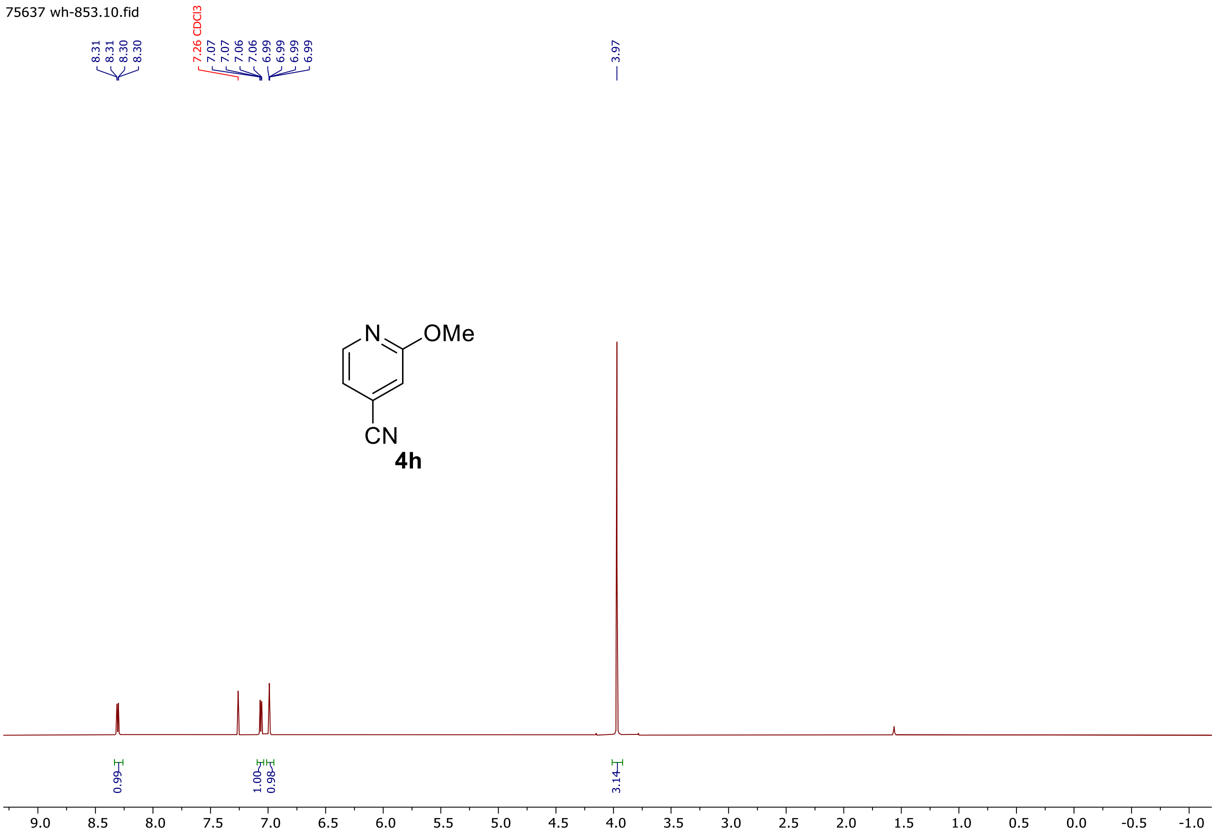

^{13}C NMR (101 MHz, $CDCl_3$) of **4h**

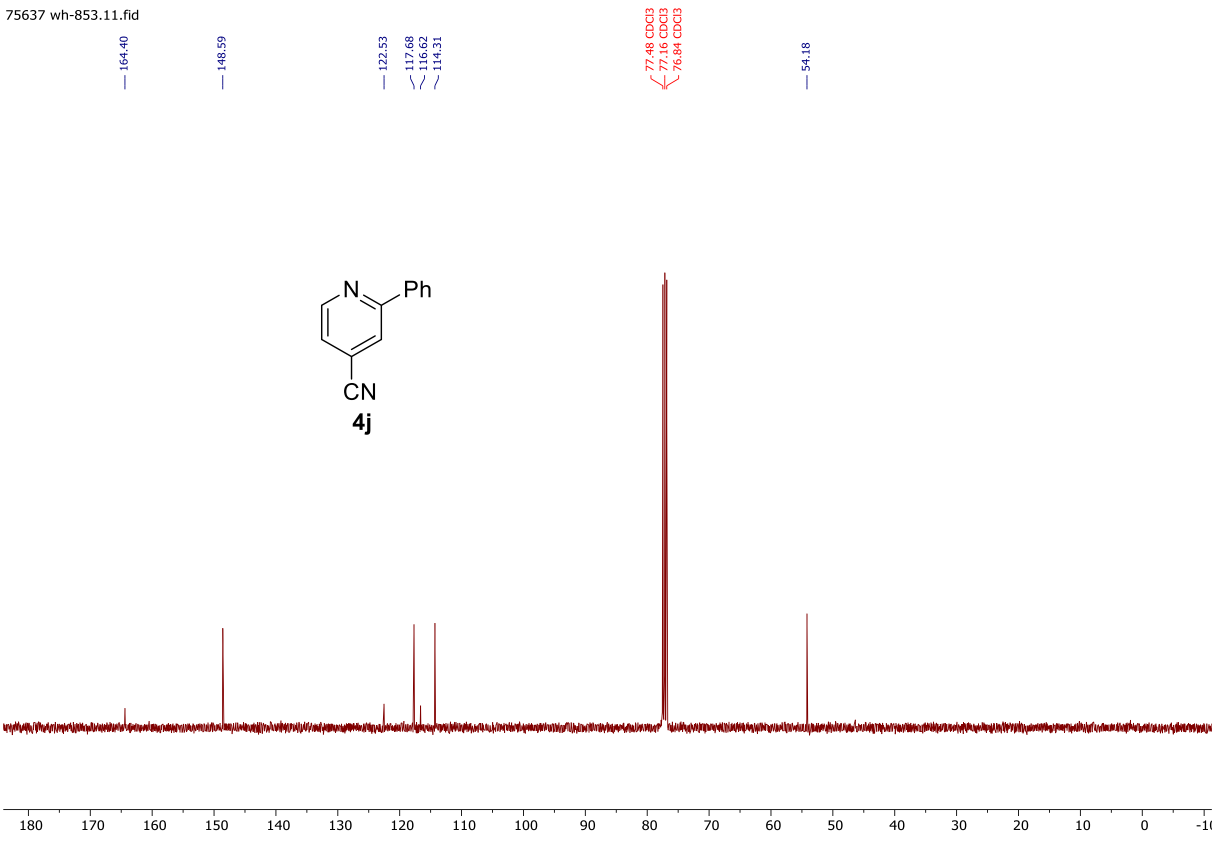

^{1}H NMR (400 MHz, $CDCl_3$) of **4j** (*see procedure*)

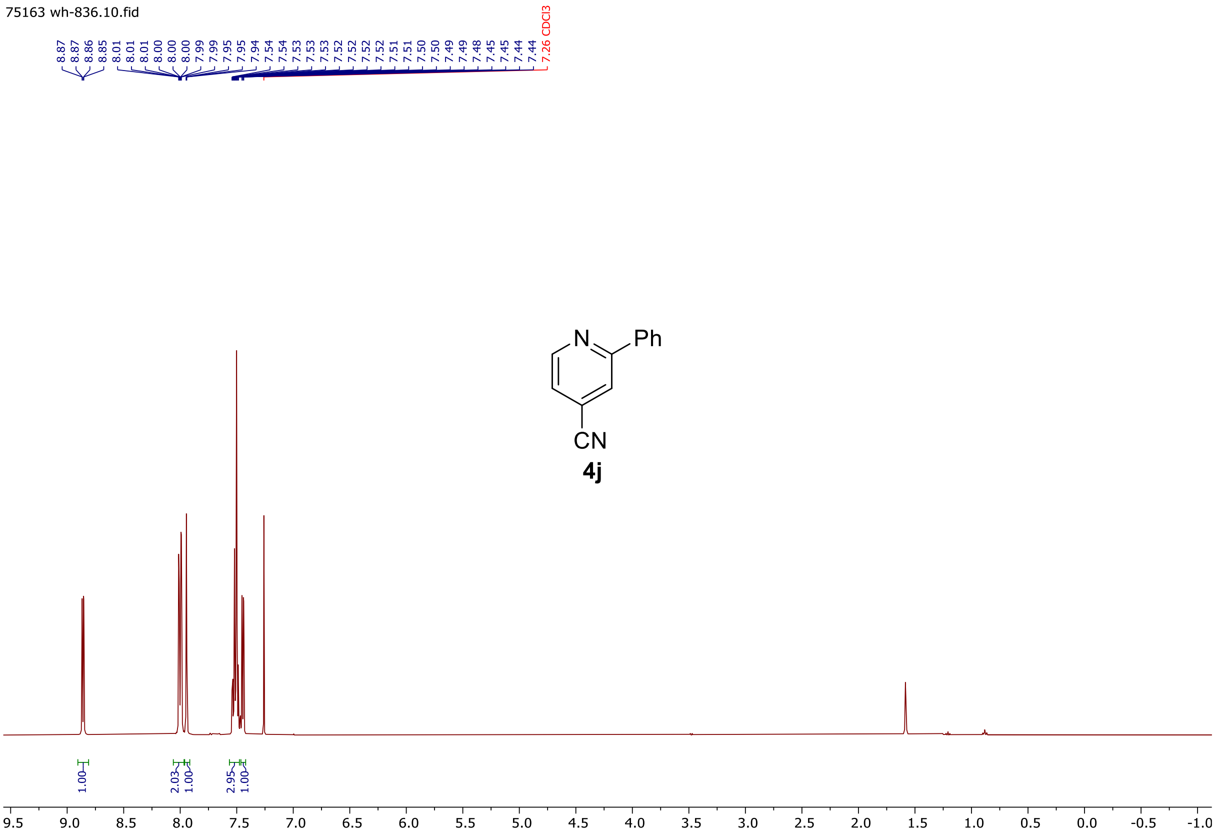

UOB Confidential & Sensitive

^{13}C NMR (101 MHz, $CDCl_3$) of **4j**

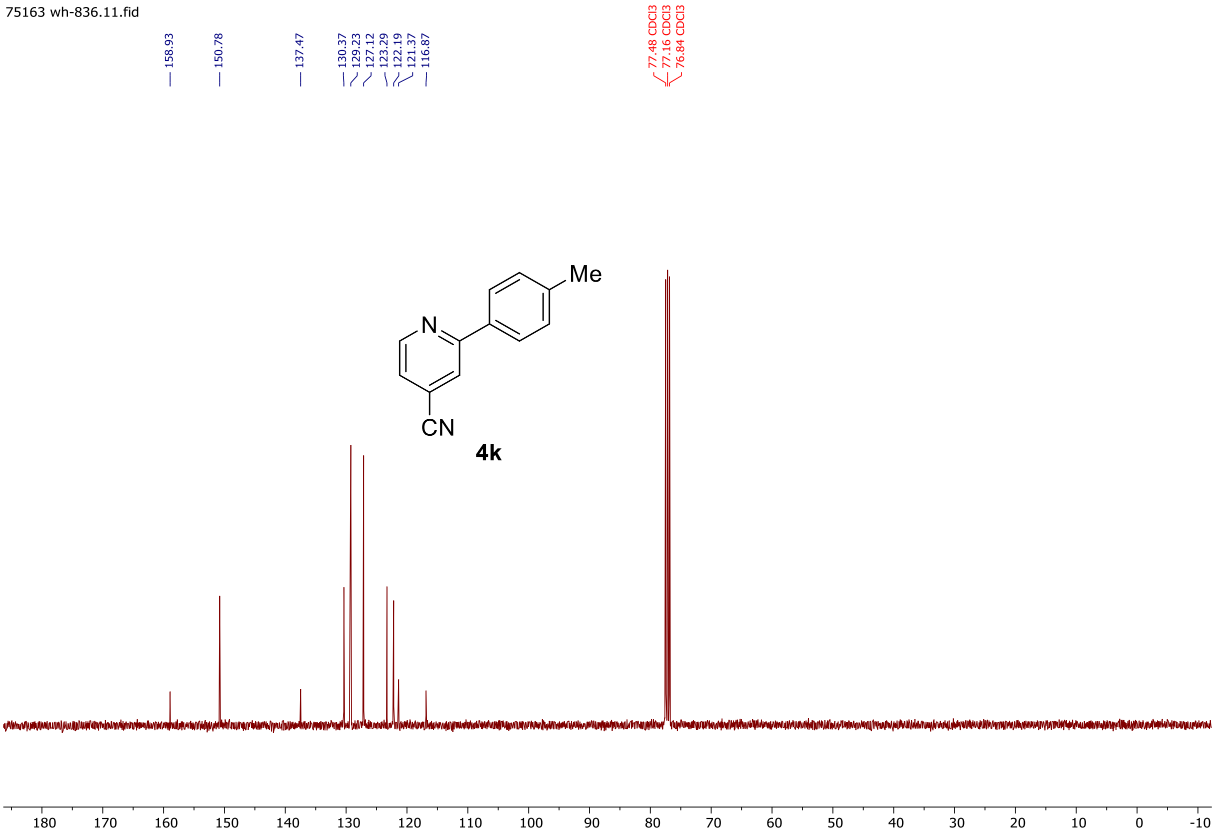

^{1}H NMR (400 MHz, $CDCl_3$) of **4k** (*see procedure*)

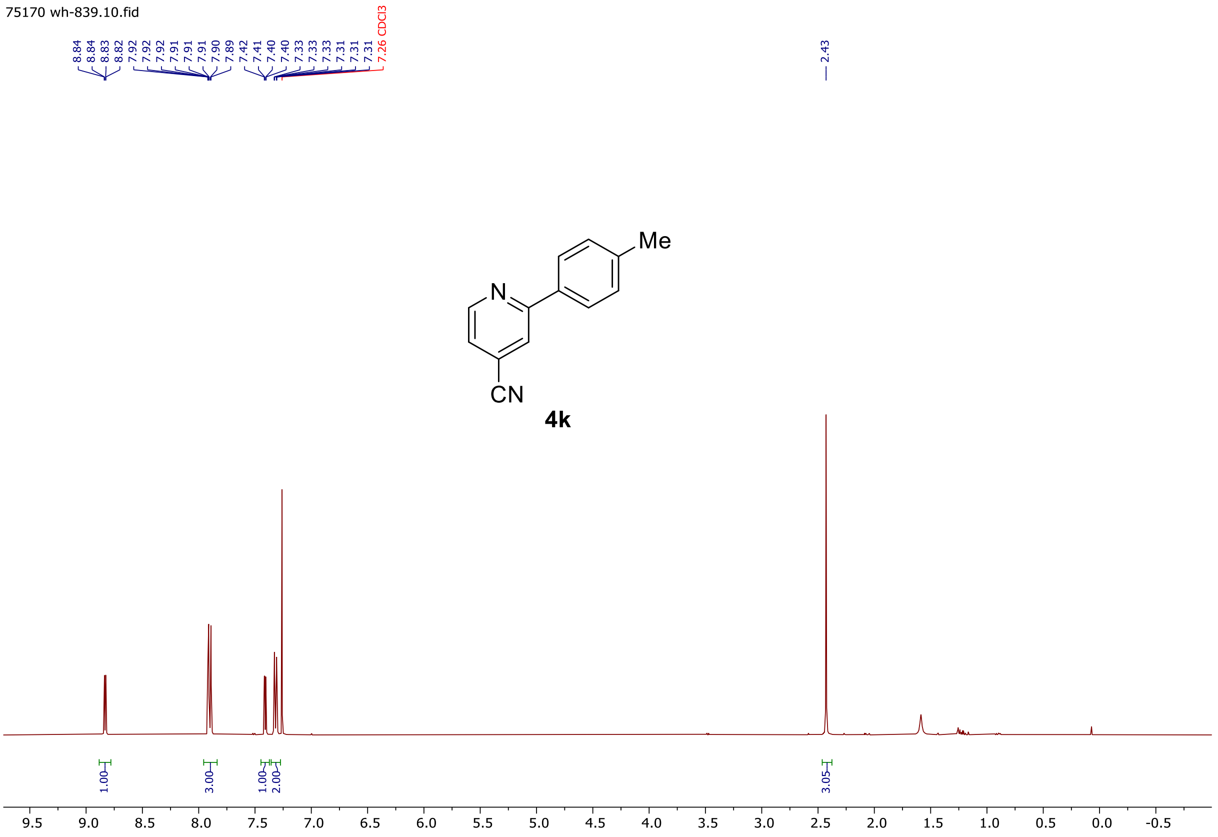

^{13}C NMR (101 MHz, $CDCl_3$) of **4k**

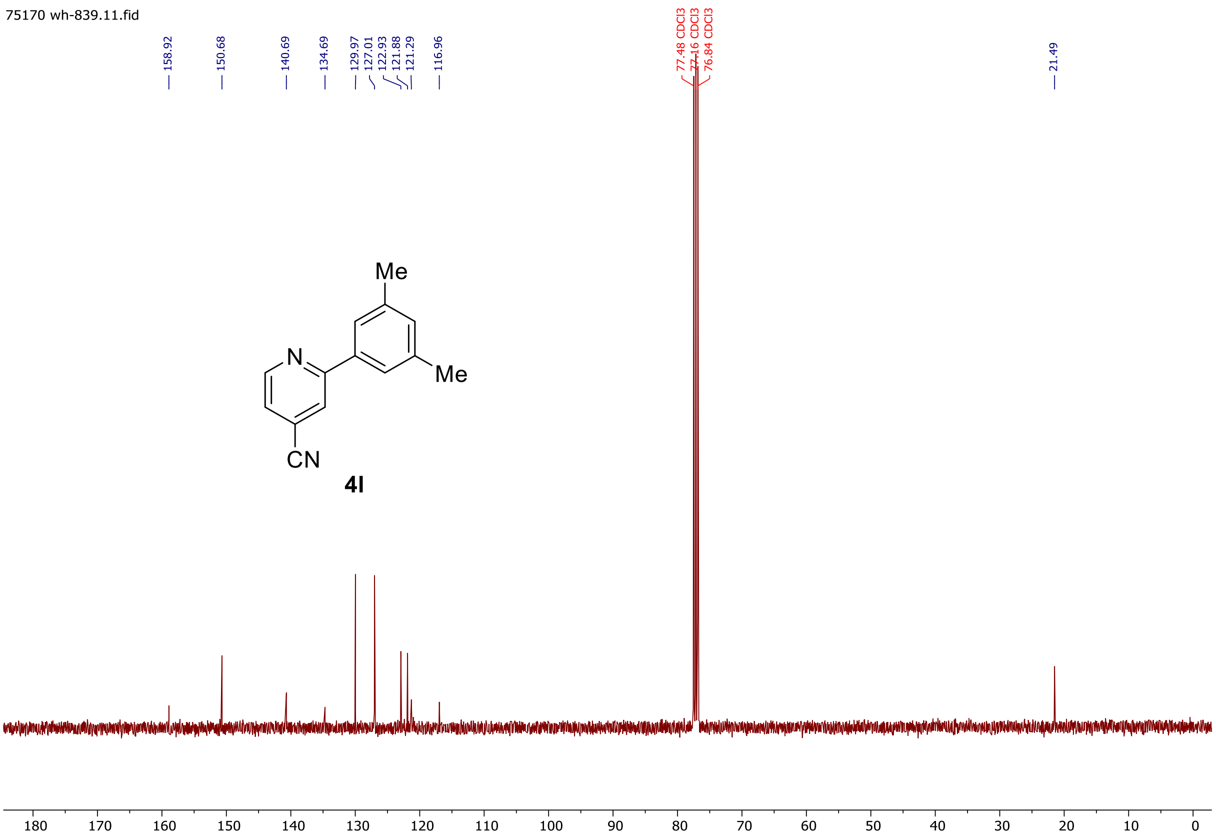

^{1}H NMR (400 MHz, $CDCl_3$) of **4l** (*see procedure*)

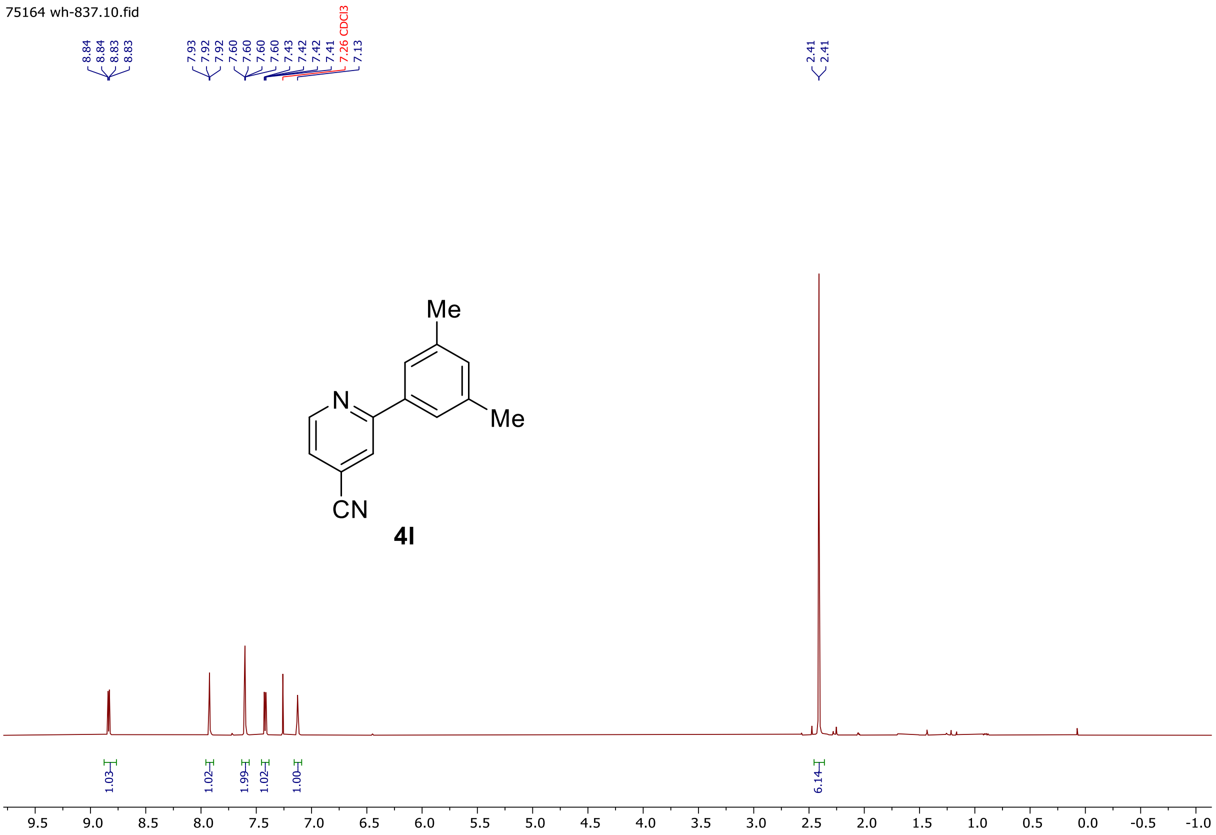

^{13}C NMR (101 MHz, $CDCl_3$) of **4l**

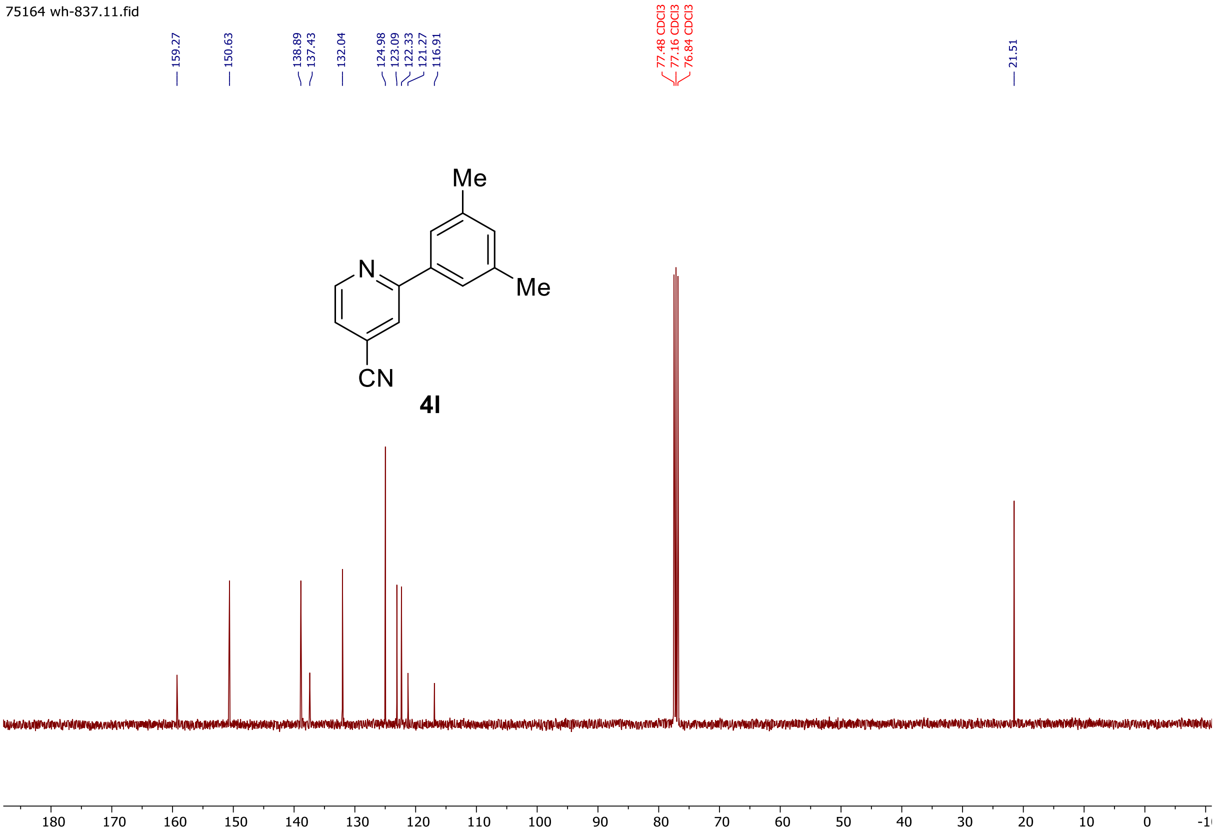

^{1}H NMR (400 MHz, $CDCl_3$) of **4m** (*see procedure*)

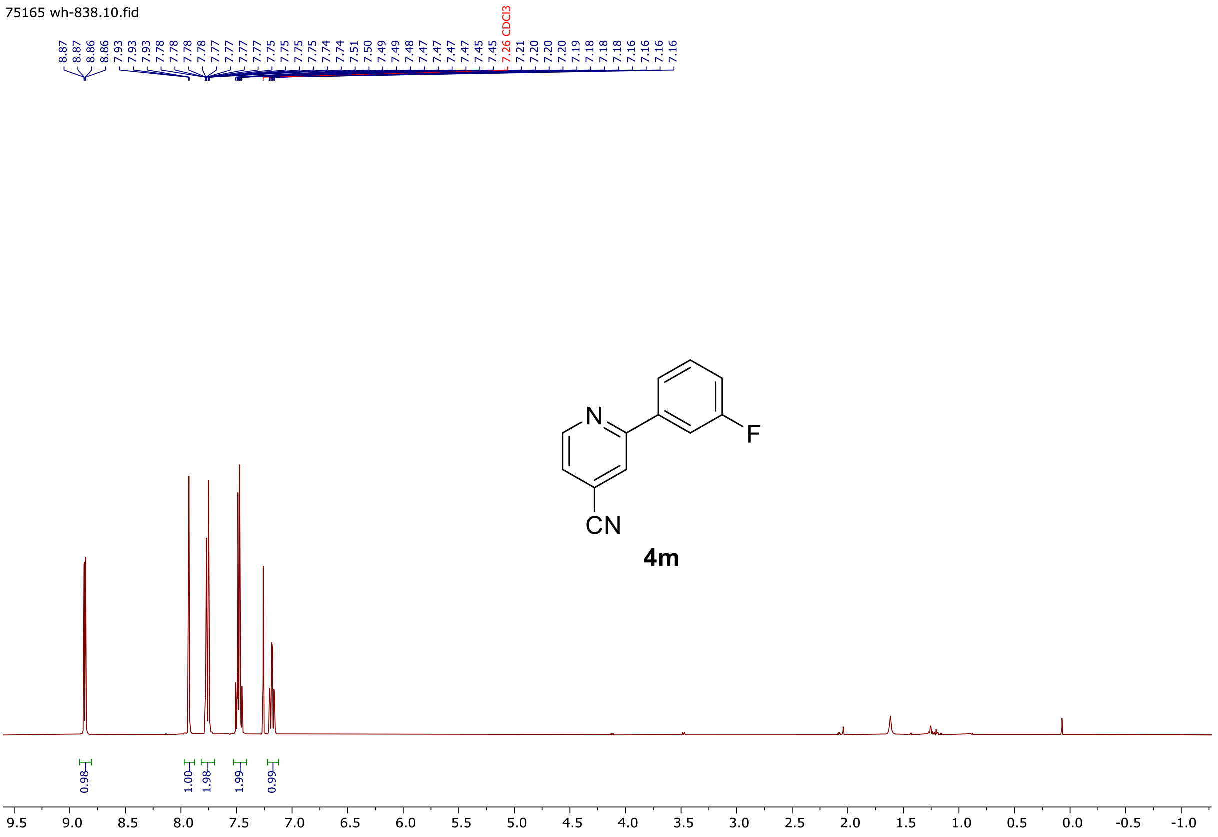

^{13}C NMR (101 MHz, $CDCl_3$) of **4m**

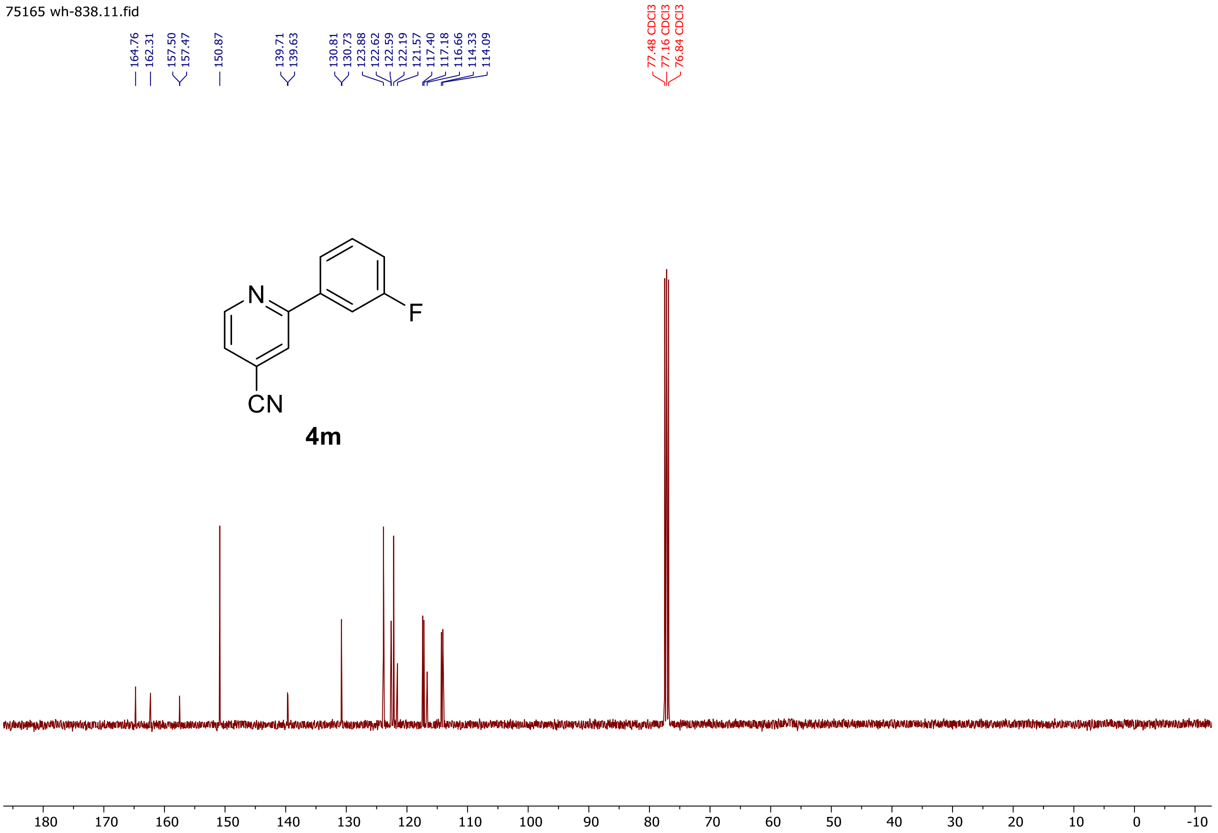

^{19}F NMR (377 MHz, $CDCl_3$) of **4m**

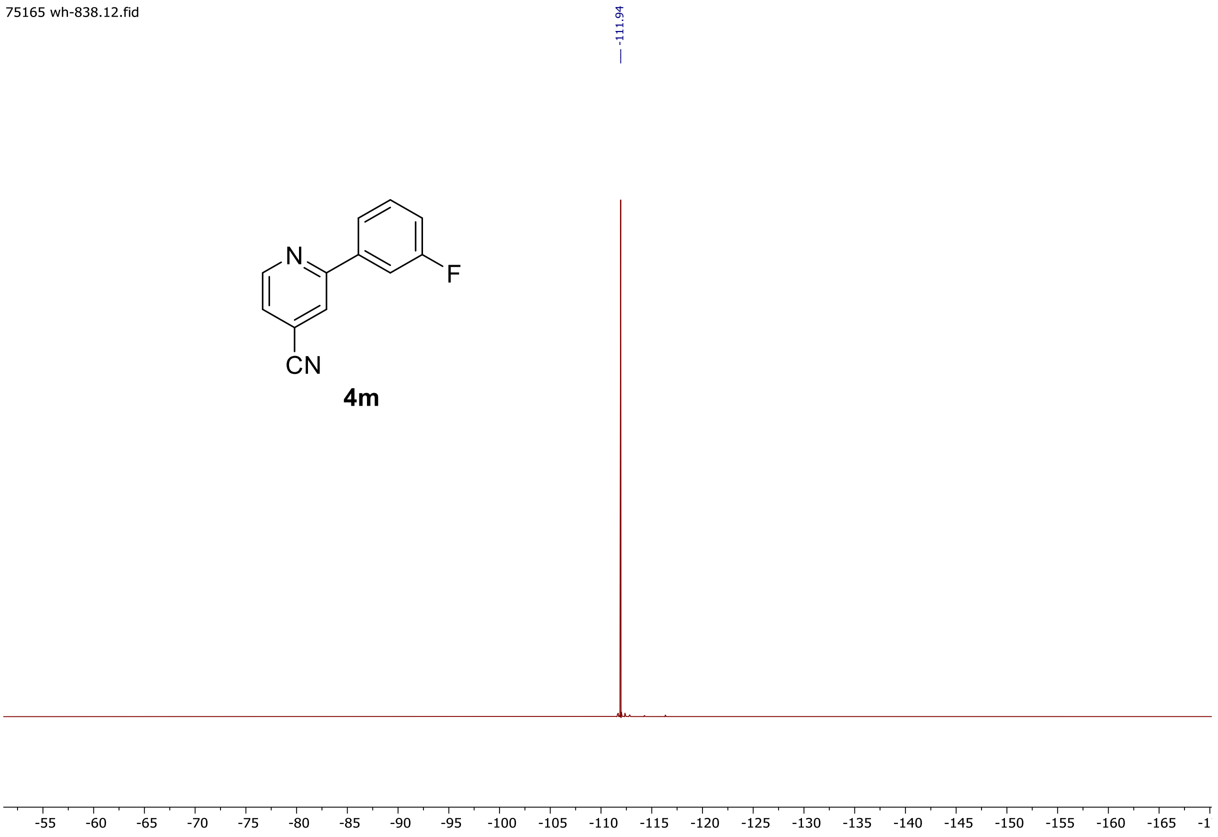

^{1}H NMR (400 MHz, $CDCl_3$) of **5b** (*see procedure*)

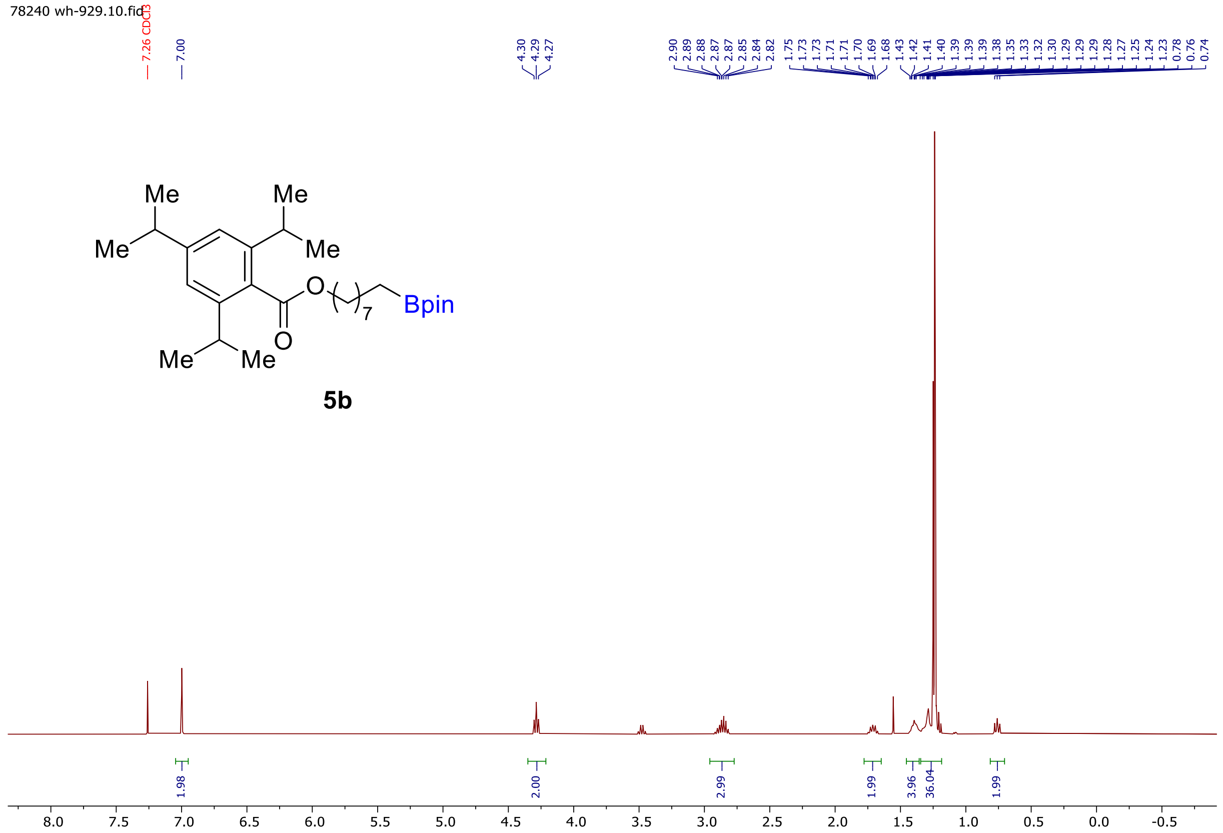

^{13}C NMR (101 MHz, $CDCl_3$) of **5b**

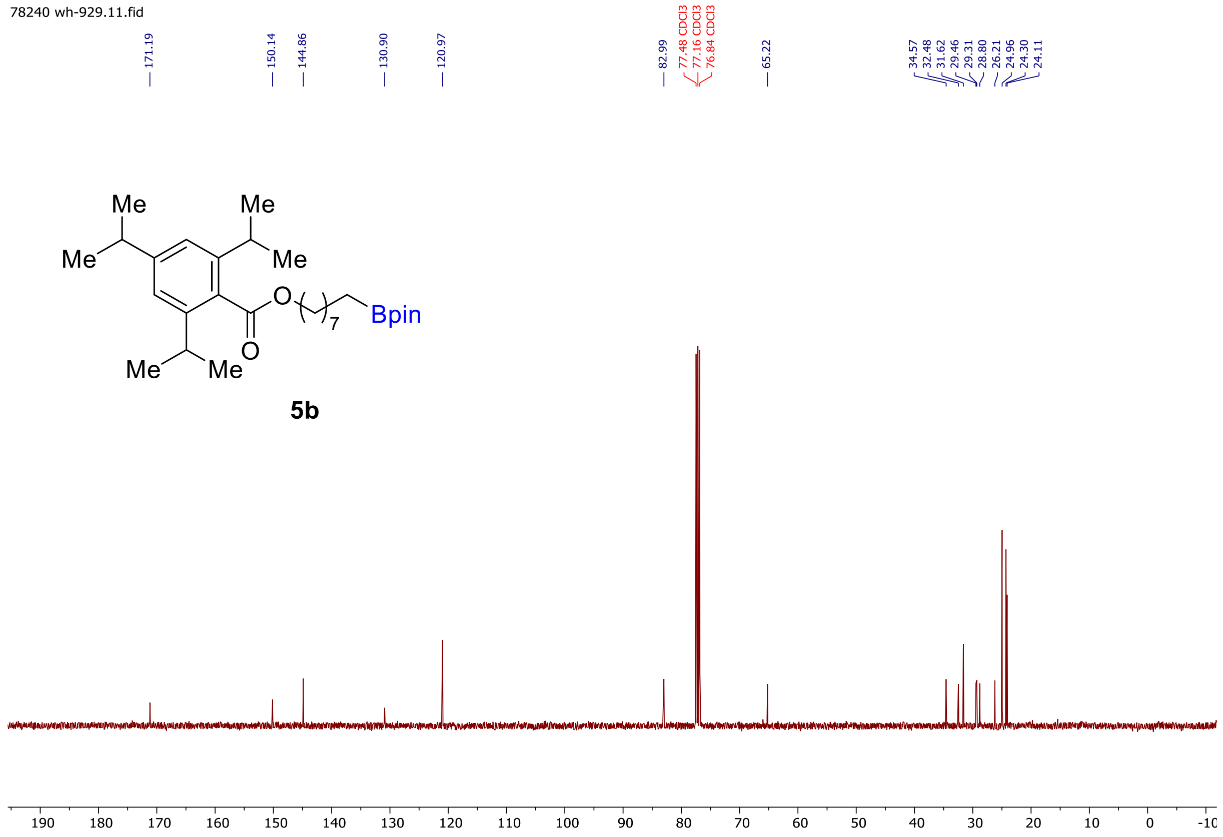

^{11}B NMR (128 MHz, $CDCl_3$) of **5b**

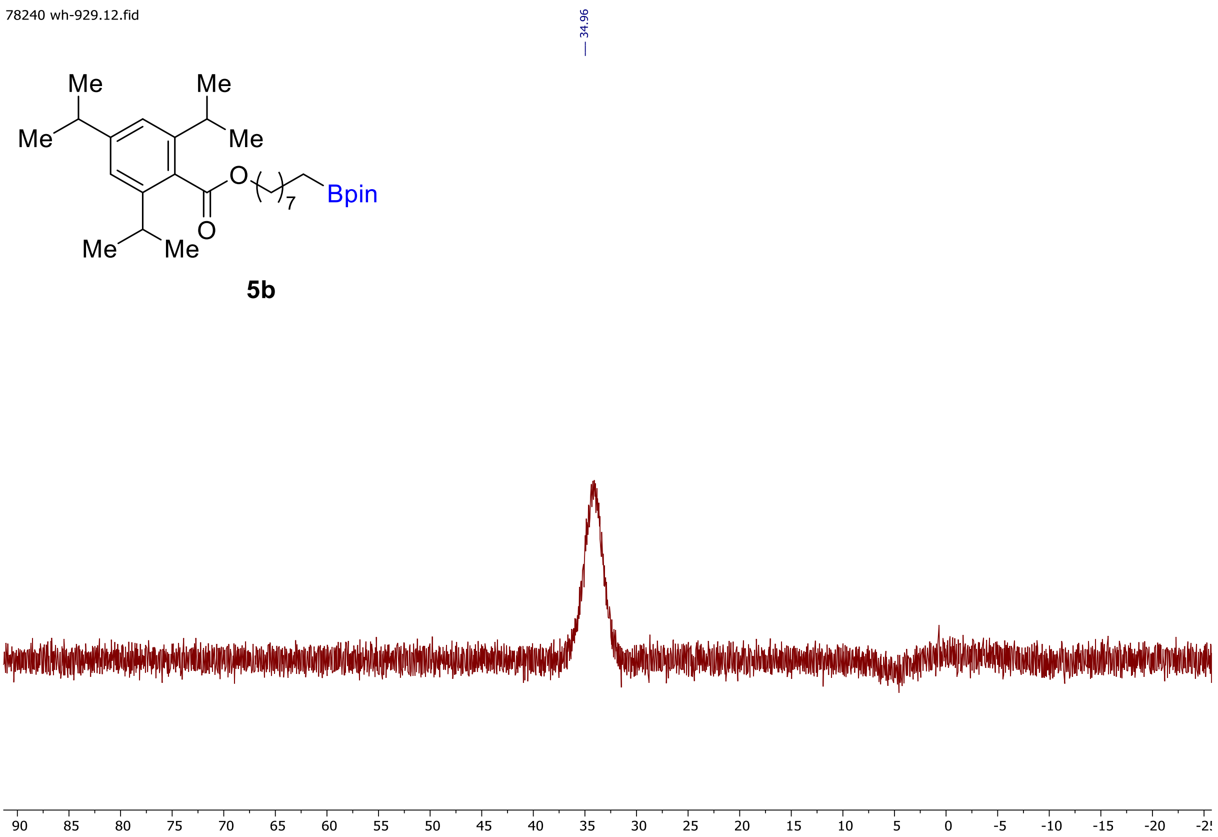

^{1}H NMR (400 MHz, $CDCl_3$) of **5e** (*see procedure*)

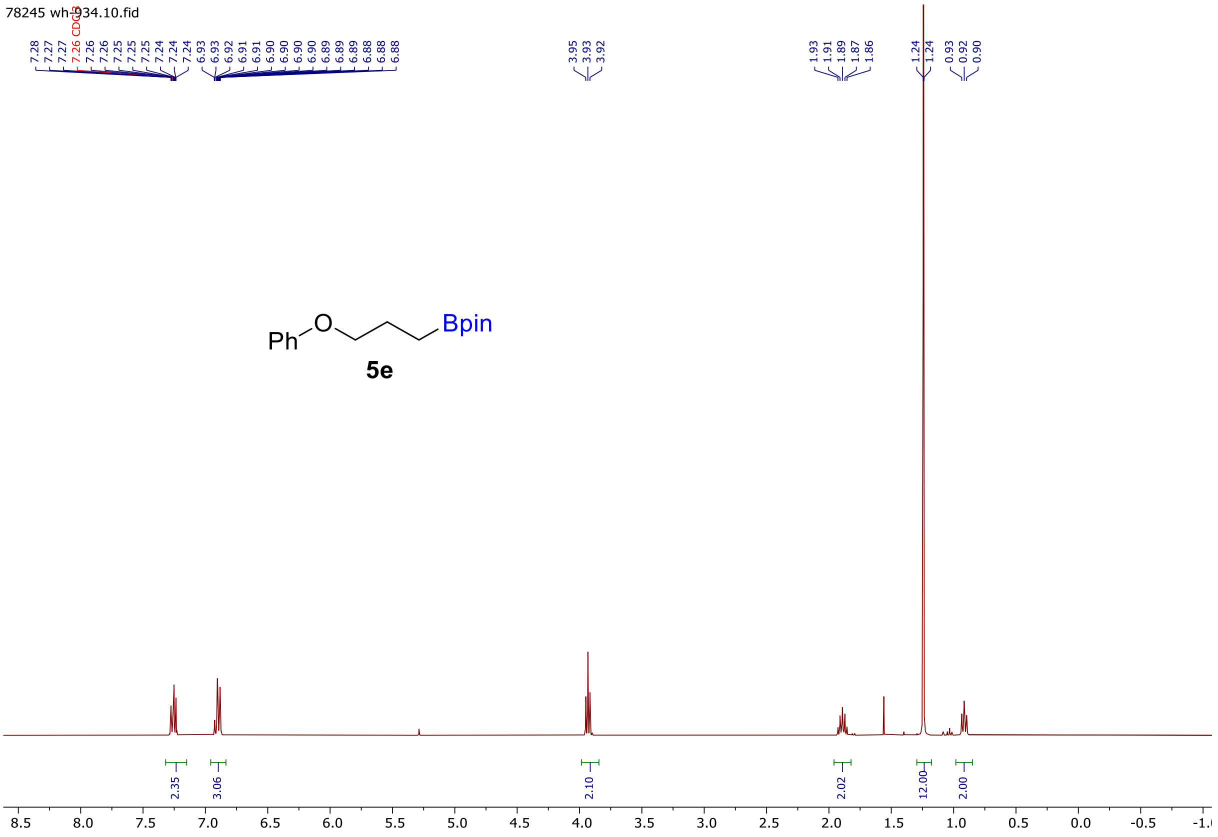

^{13}C NMR (101 MHz, $CDCl_3$) of **5e**

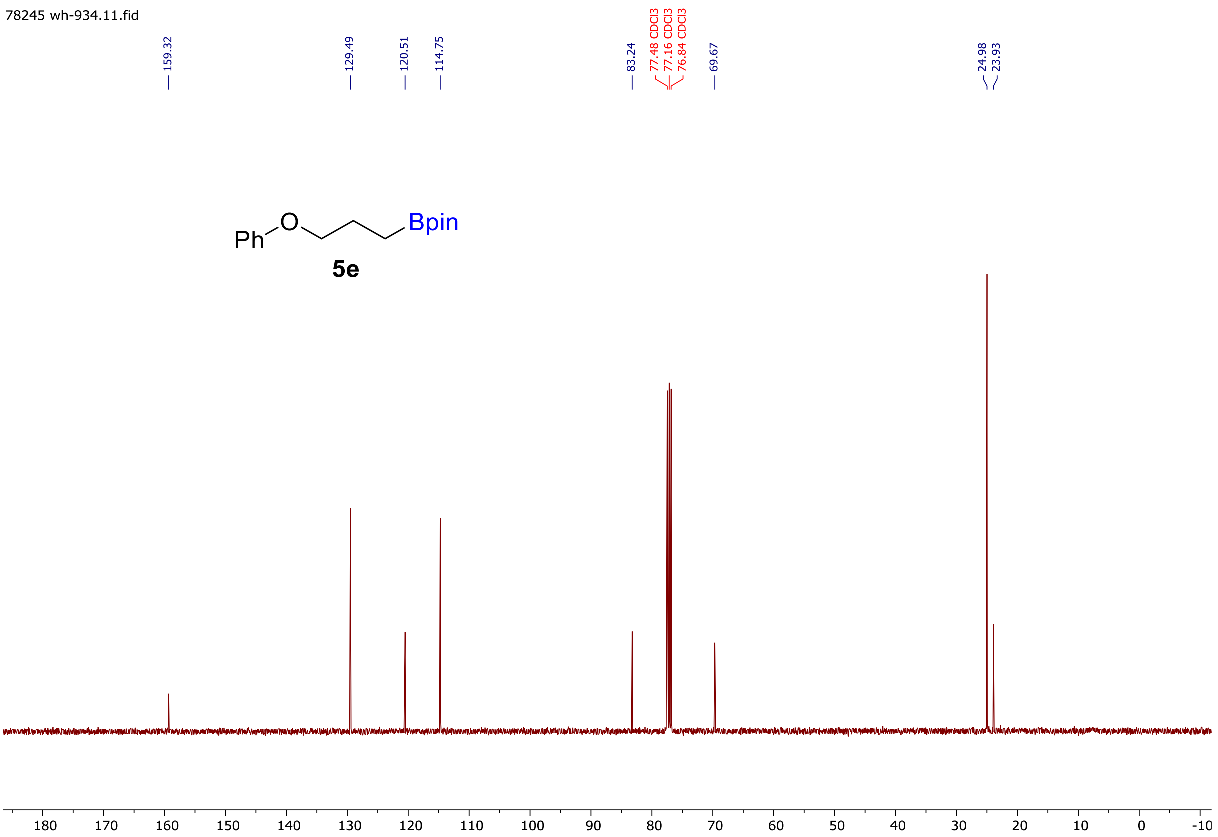

^{11}B NMR (128 MHz, $CDCl_3$) of **5e**

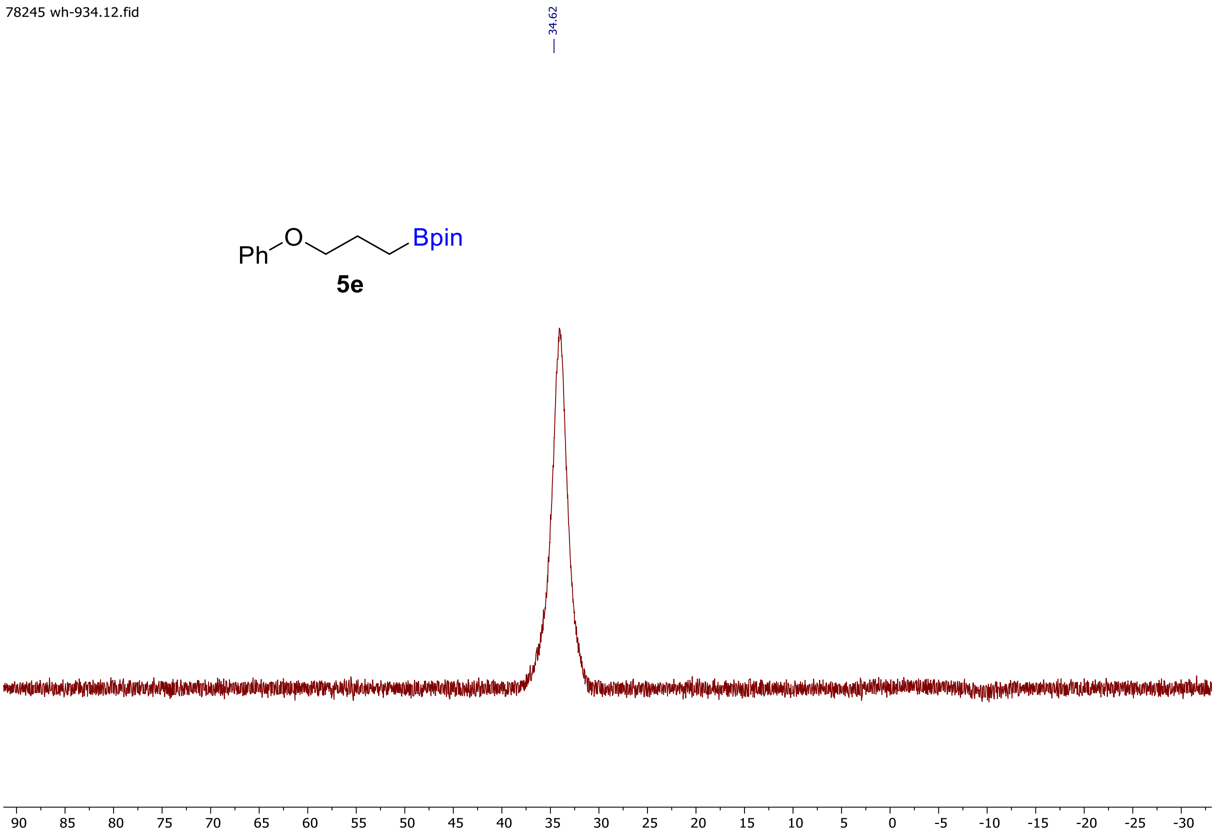

^{1}H NMR (400 MHz, $CDCl_3$) of **5q** (*see procedure*)

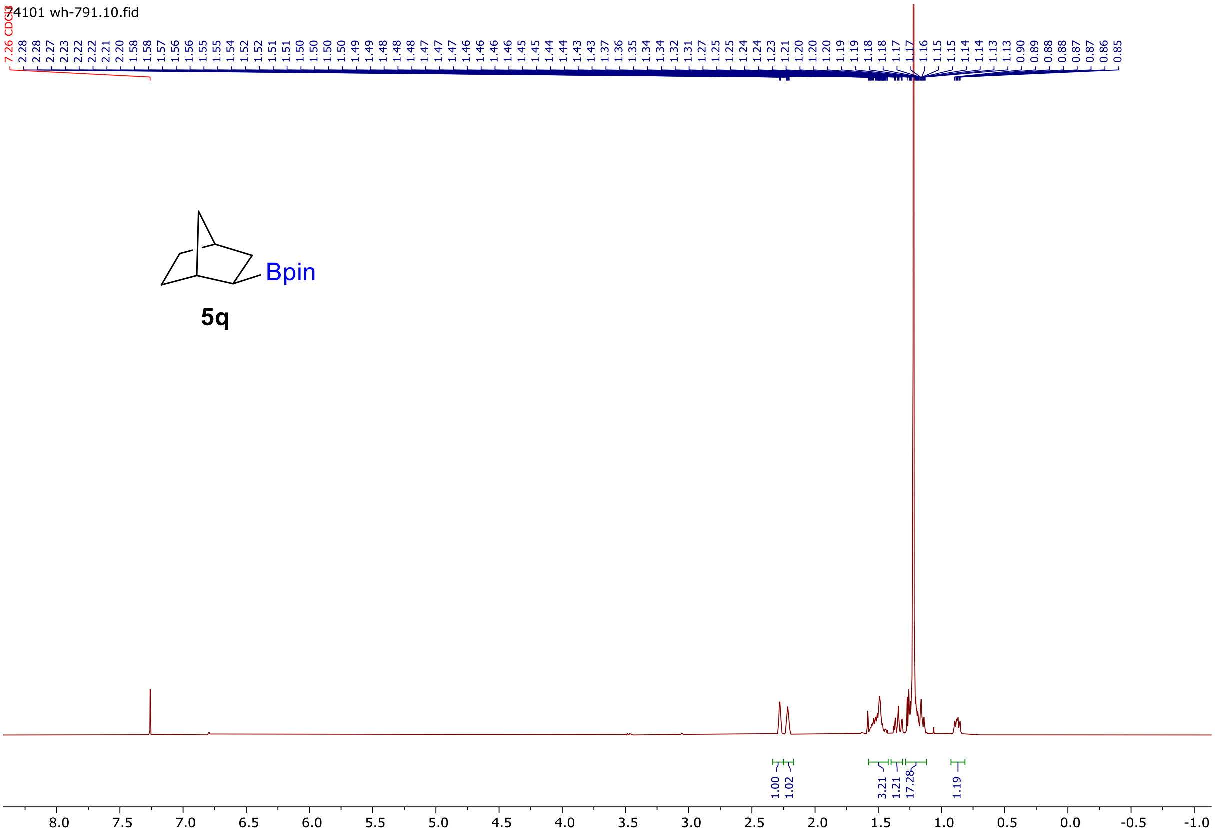

^{13}C NMR (101 MHz, $CDCl_3$) of **5q**

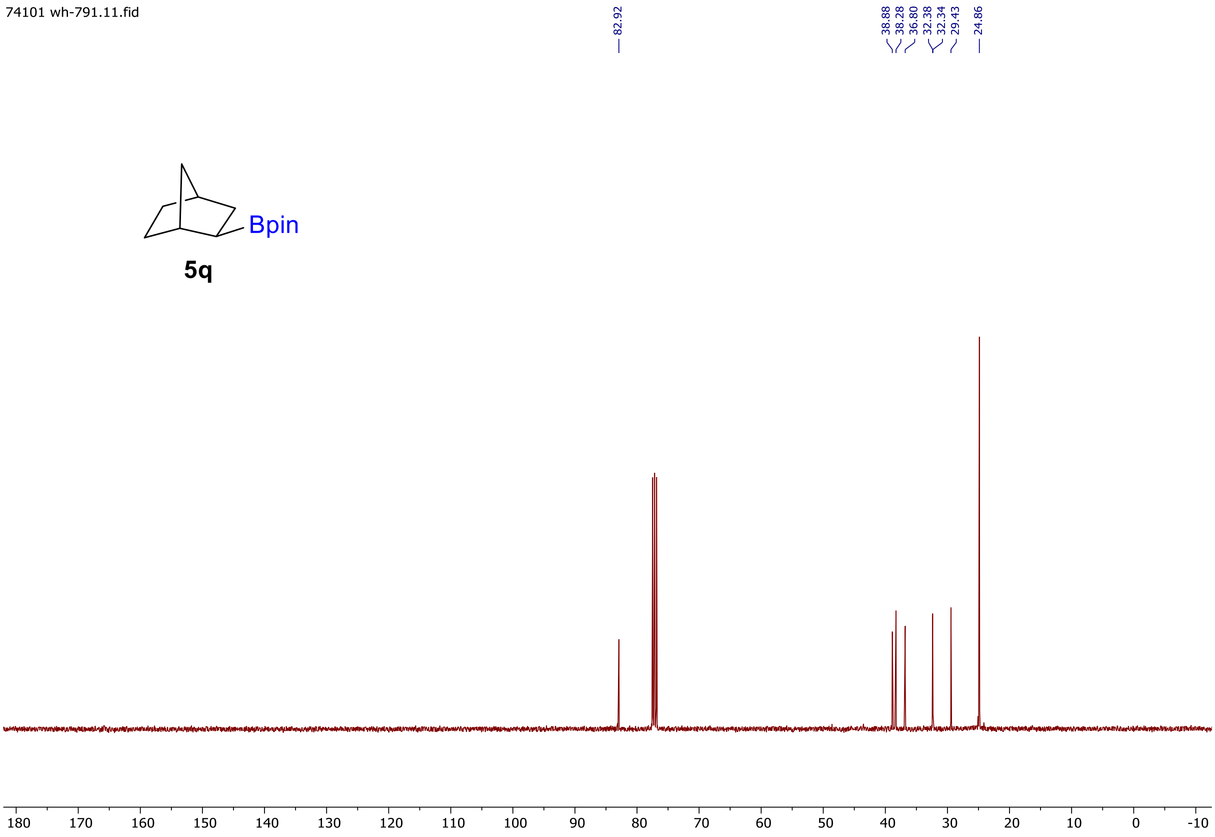

UOB Confidential & Sensitive

^{11}B NMR (128 MHz, $CDCl_3$) of **5q**

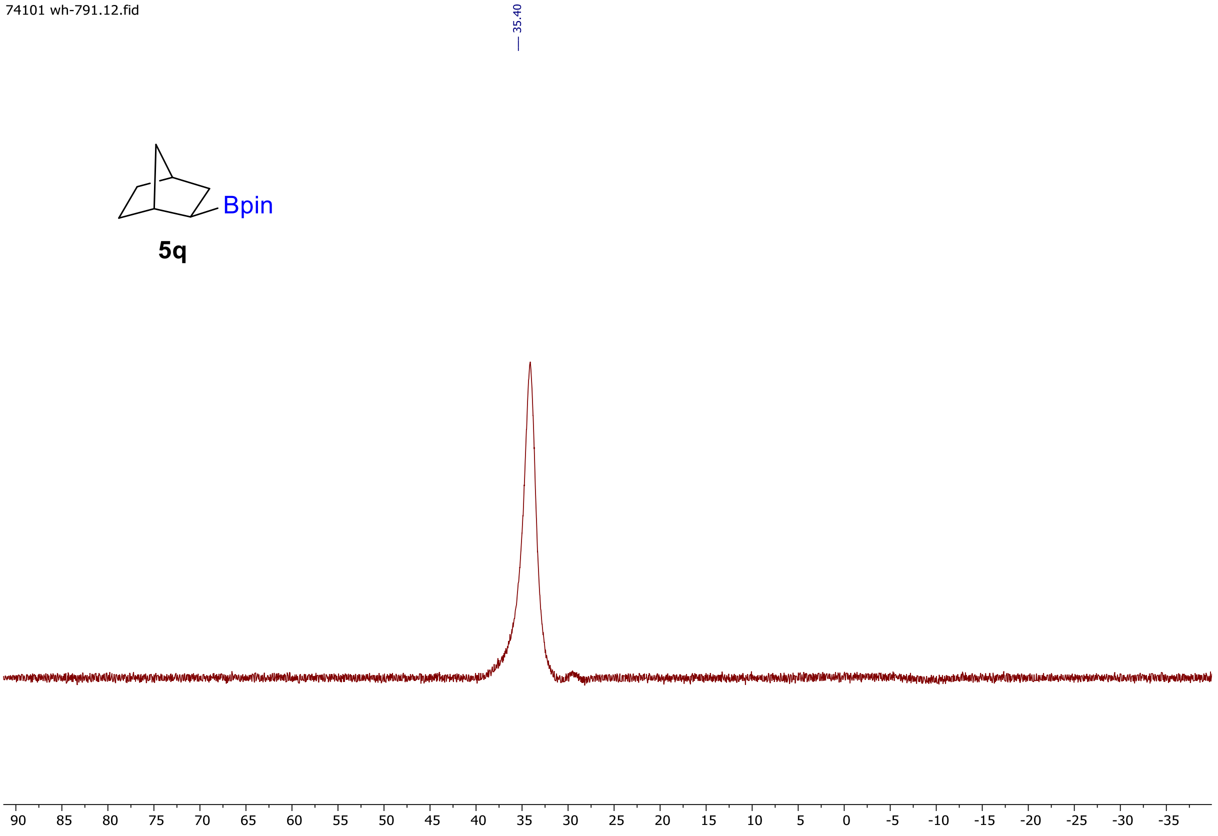

^{1}H NMR (400 MHz, $CDCl_3$) of **5o** (*see procedure*)

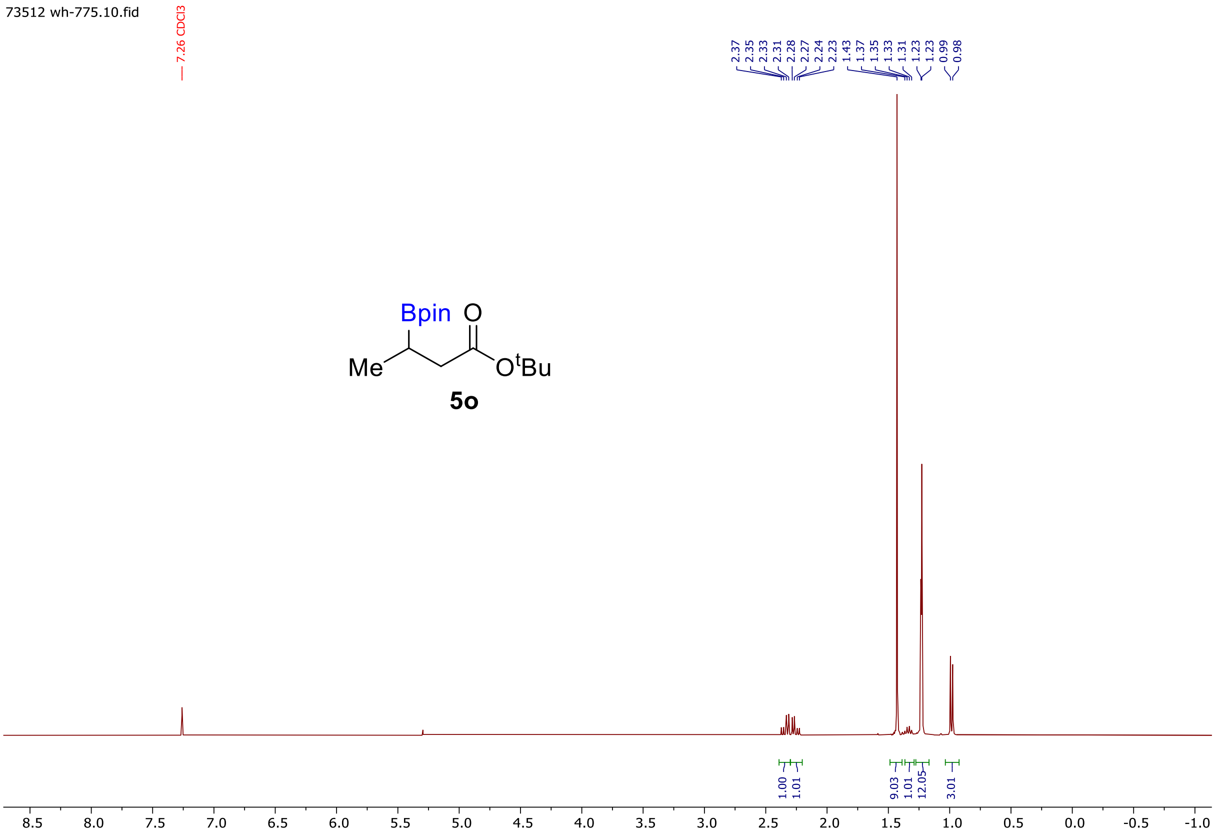

UOB Confidential & Sensitive

^{13}C NMR (101 MHz, CDCl$_3$) of **5o**

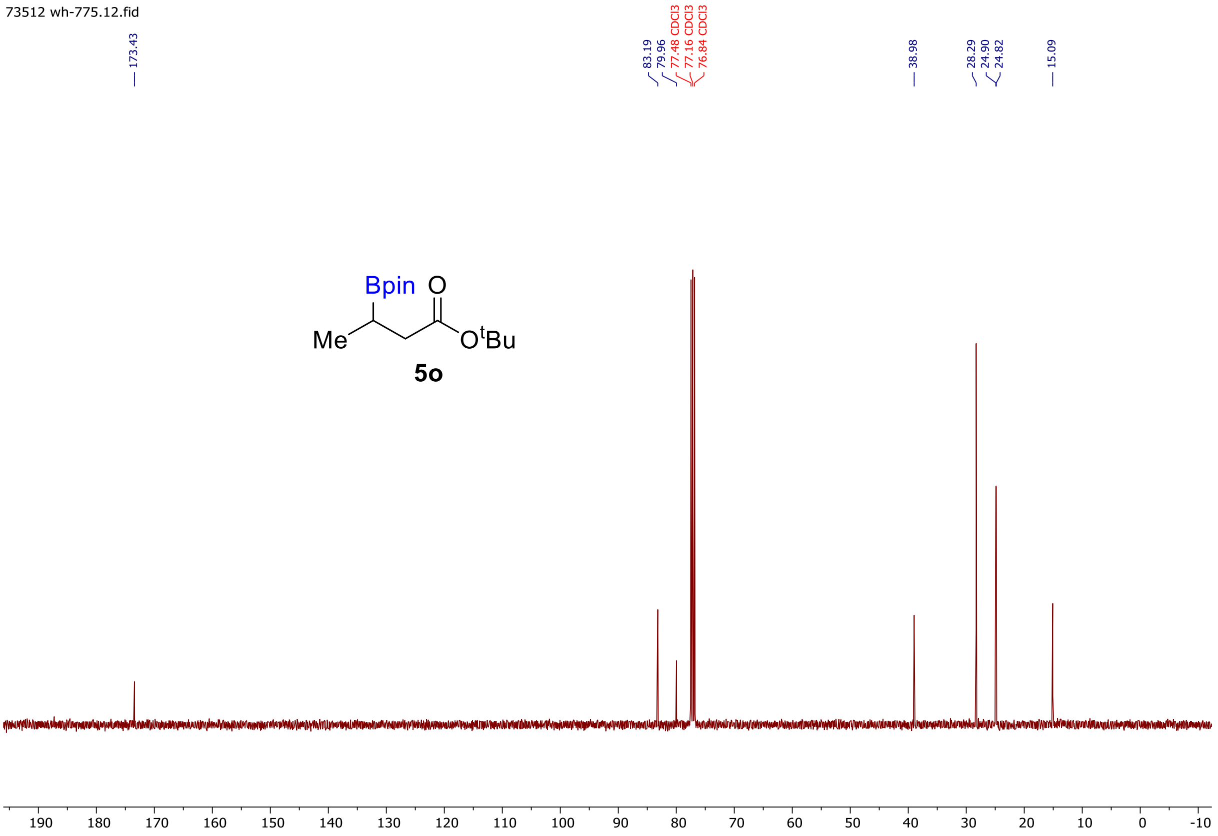

^{11}B NMR (128 MHz, CDCl$_3$) of **5o**

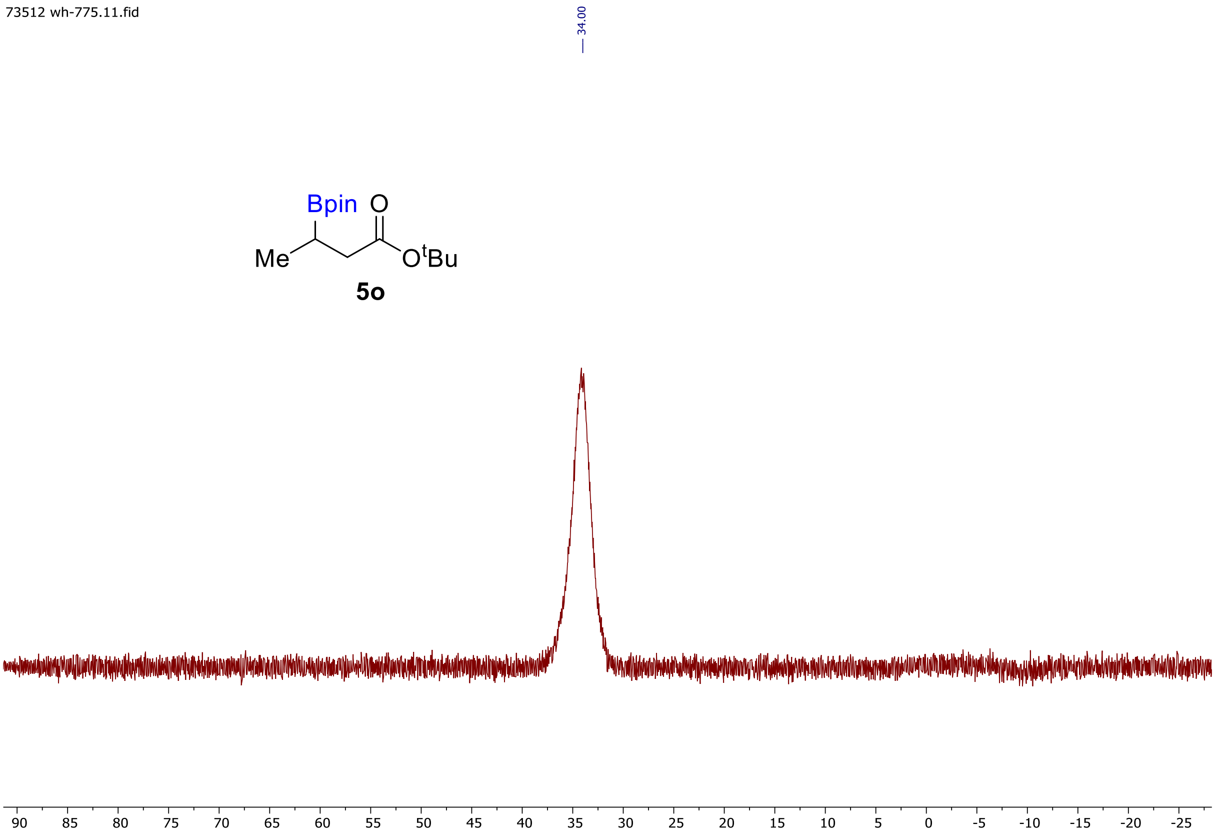

^{1}H NMR (400 MHz, $CDCl_3$) of **5r** (*see procedure*)

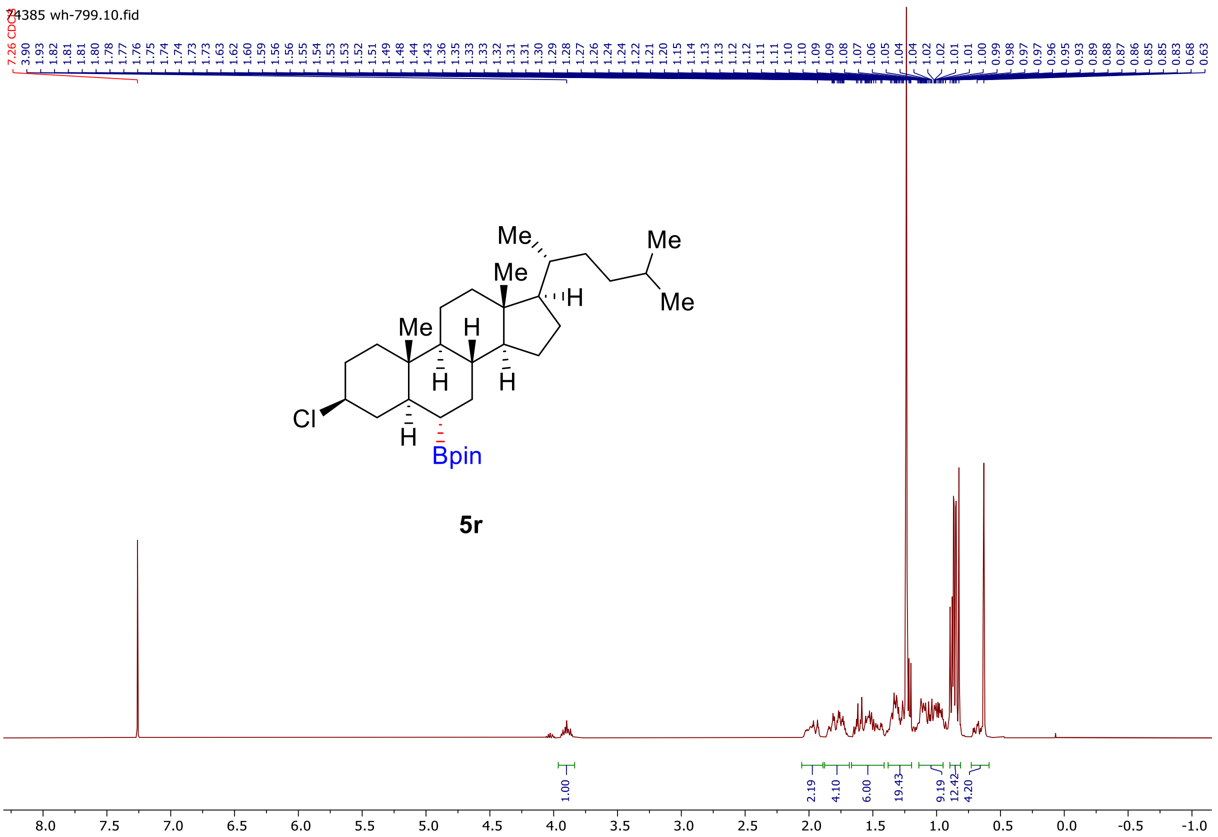

^{13}C NMR (101 MHz, $CDCl_3$) of **5r**

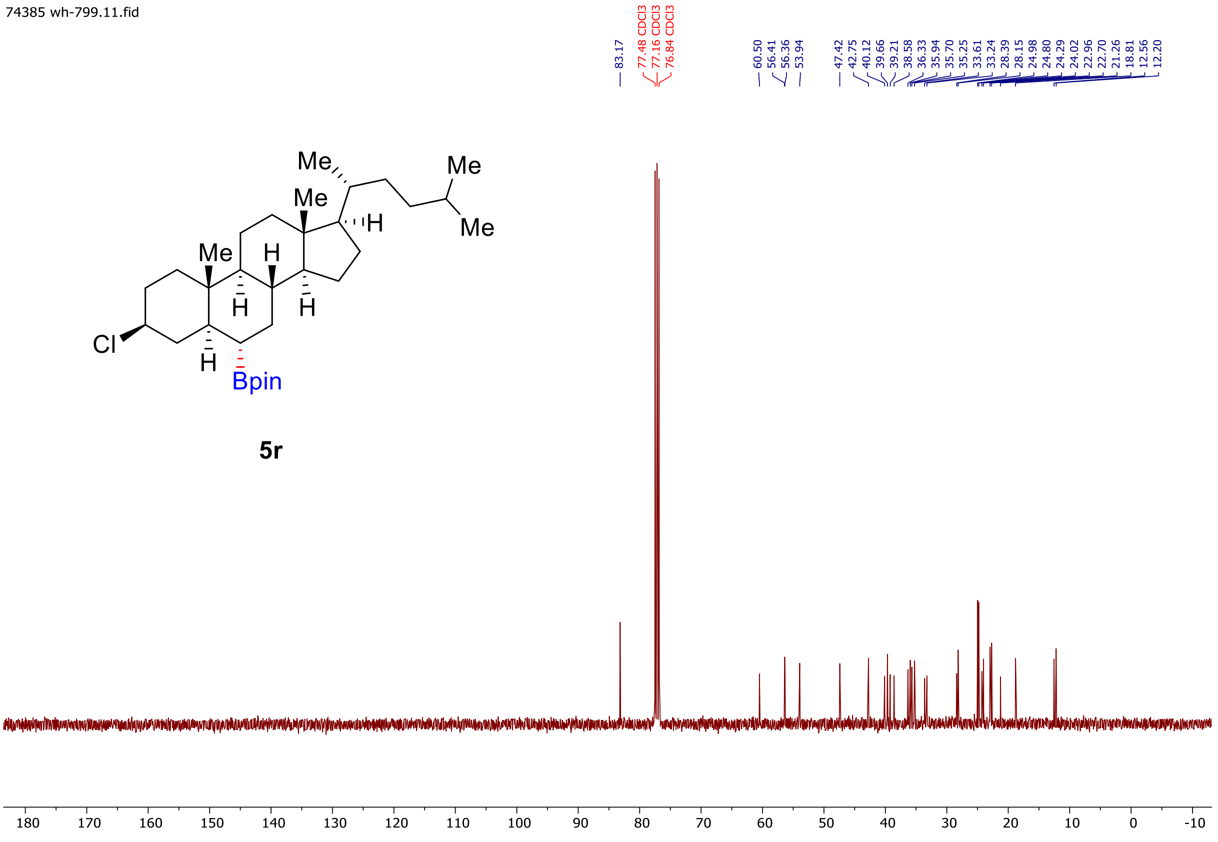

^{1}H NMR (400 MHz, $CDCl_3$) of **3aa** (*see procedure*)

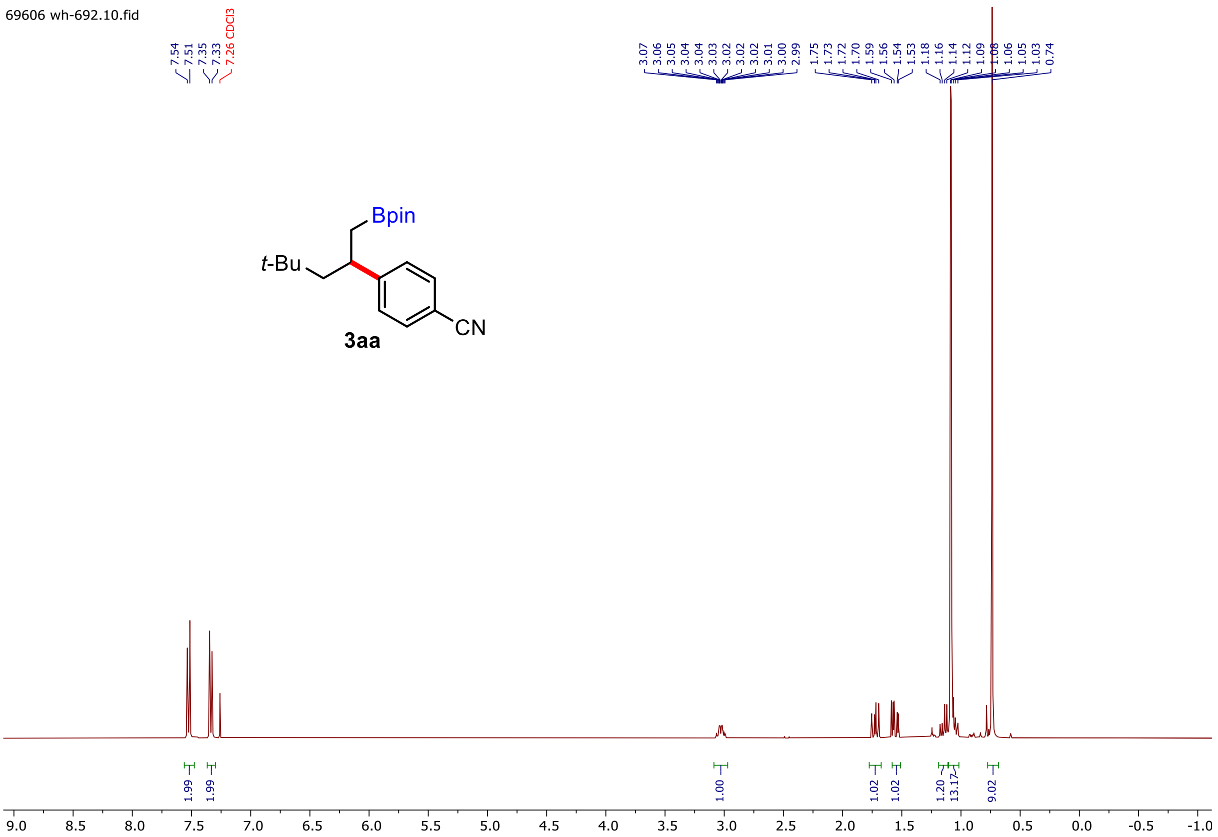

^{13}C NMR (101 MHz, $CDCl_3$) of **3aa**

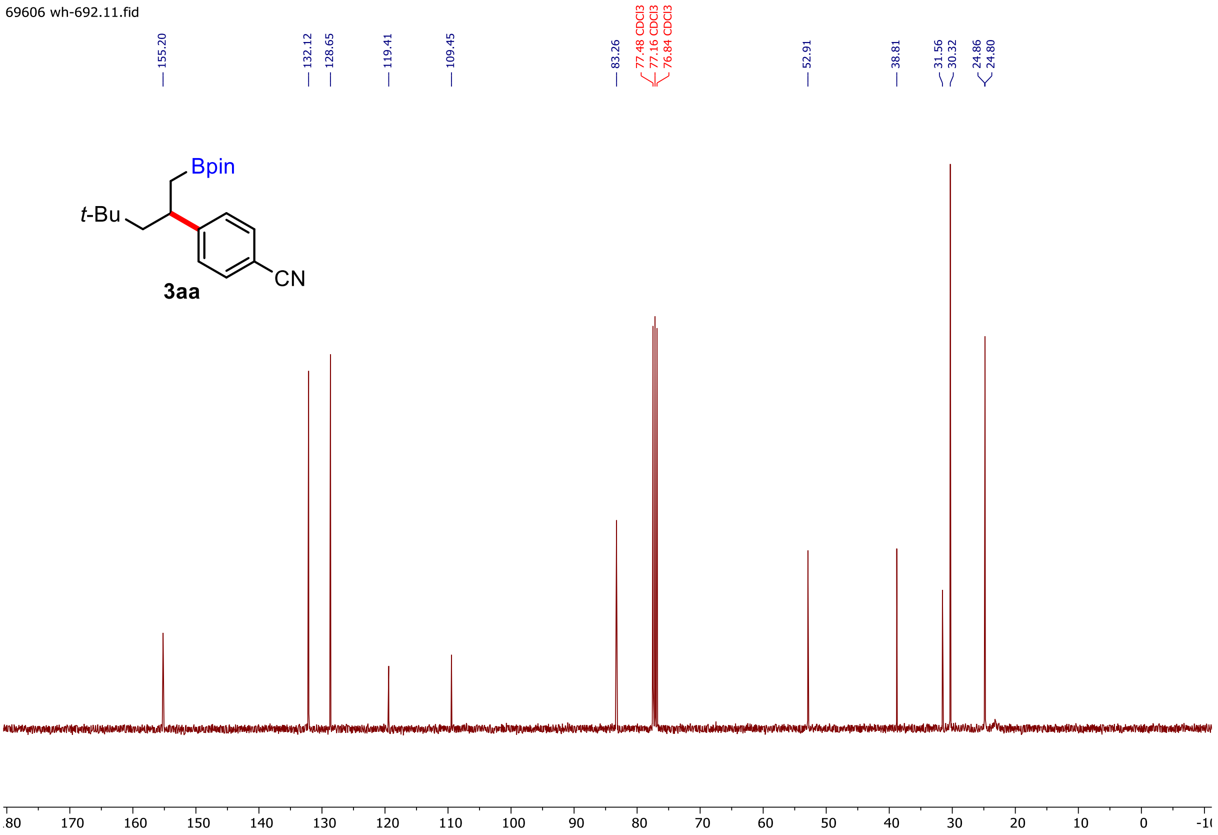

^{11}B NMR (128 MHz, $CDCl_3$) of **3aa**

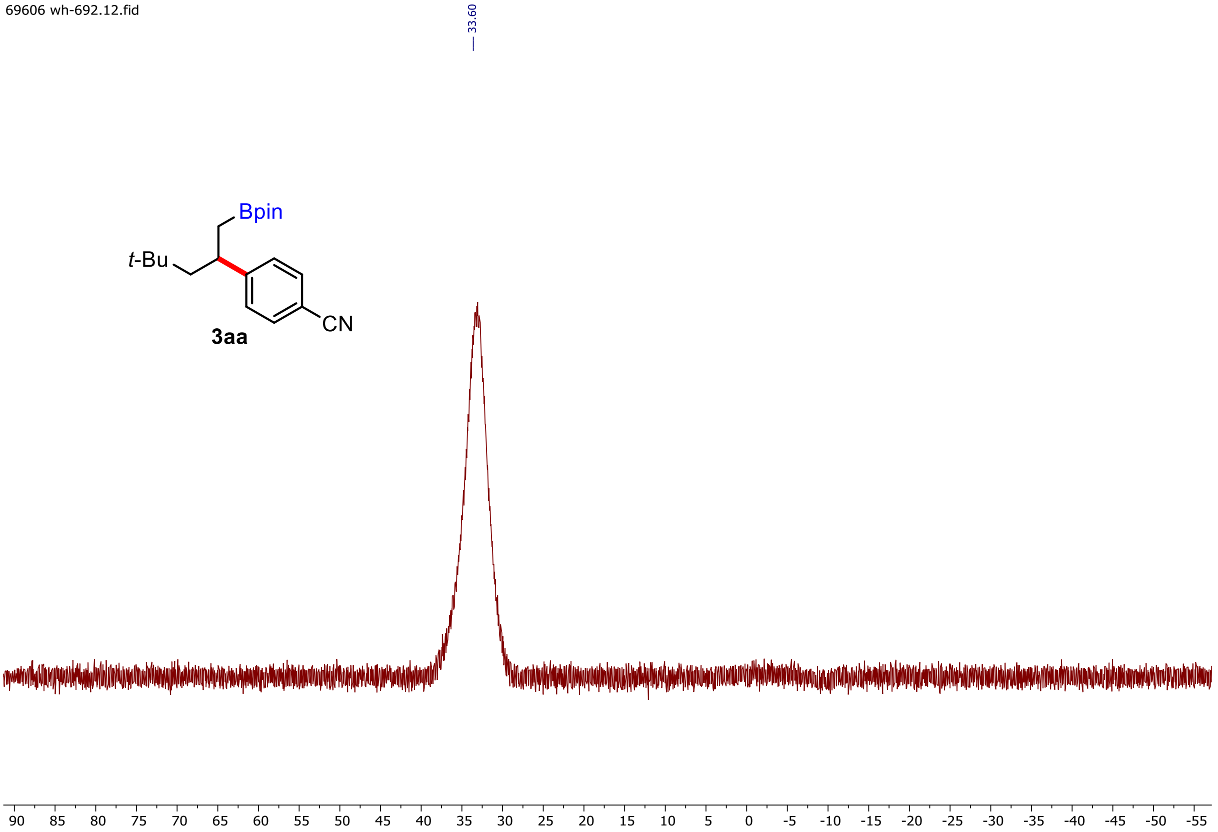

^{1}H NMR (400 MHz, $CDCl_3$) of **3ba** (*see procedure*)

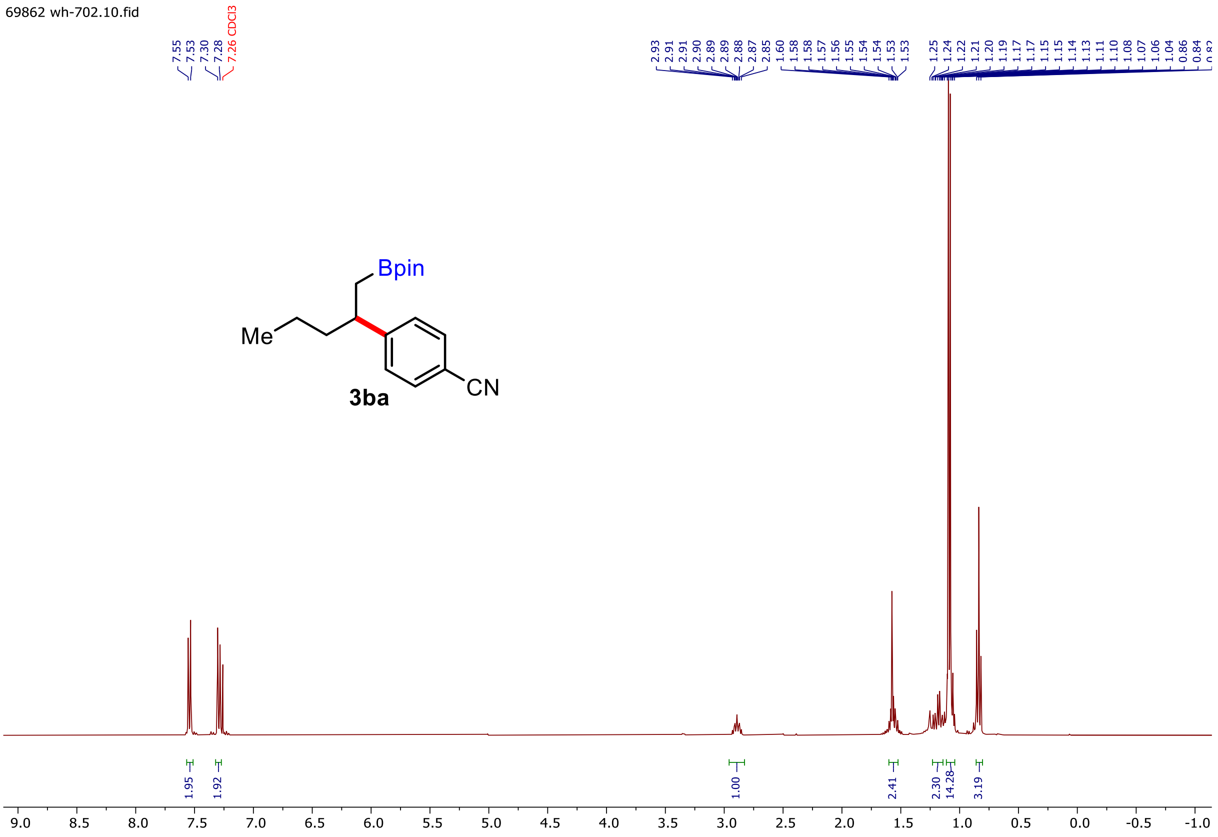

UOB Confidential & Sensitive

^{13}C NMR (101 MHz, $CDCl_3$) of **3ba**

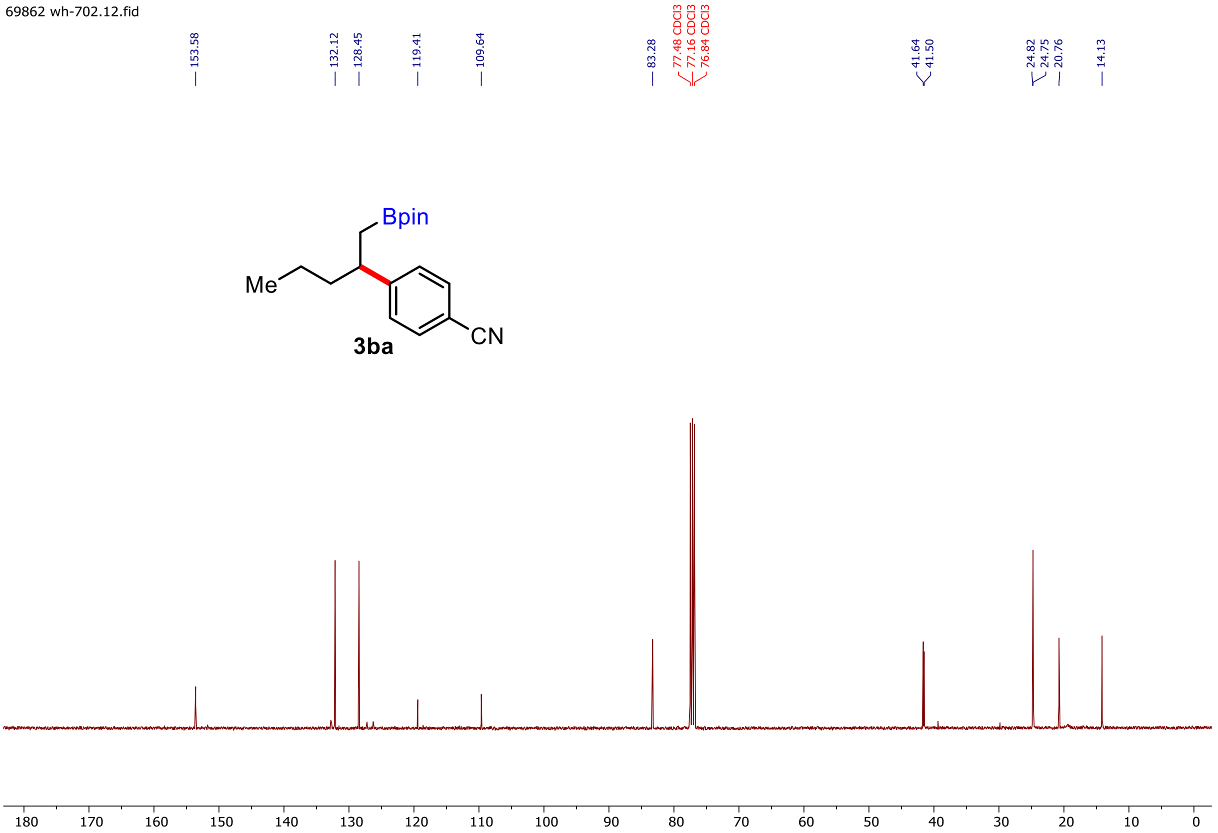

^{11}B NMR (128 MHz, $CDCl_3$) of **3ba**

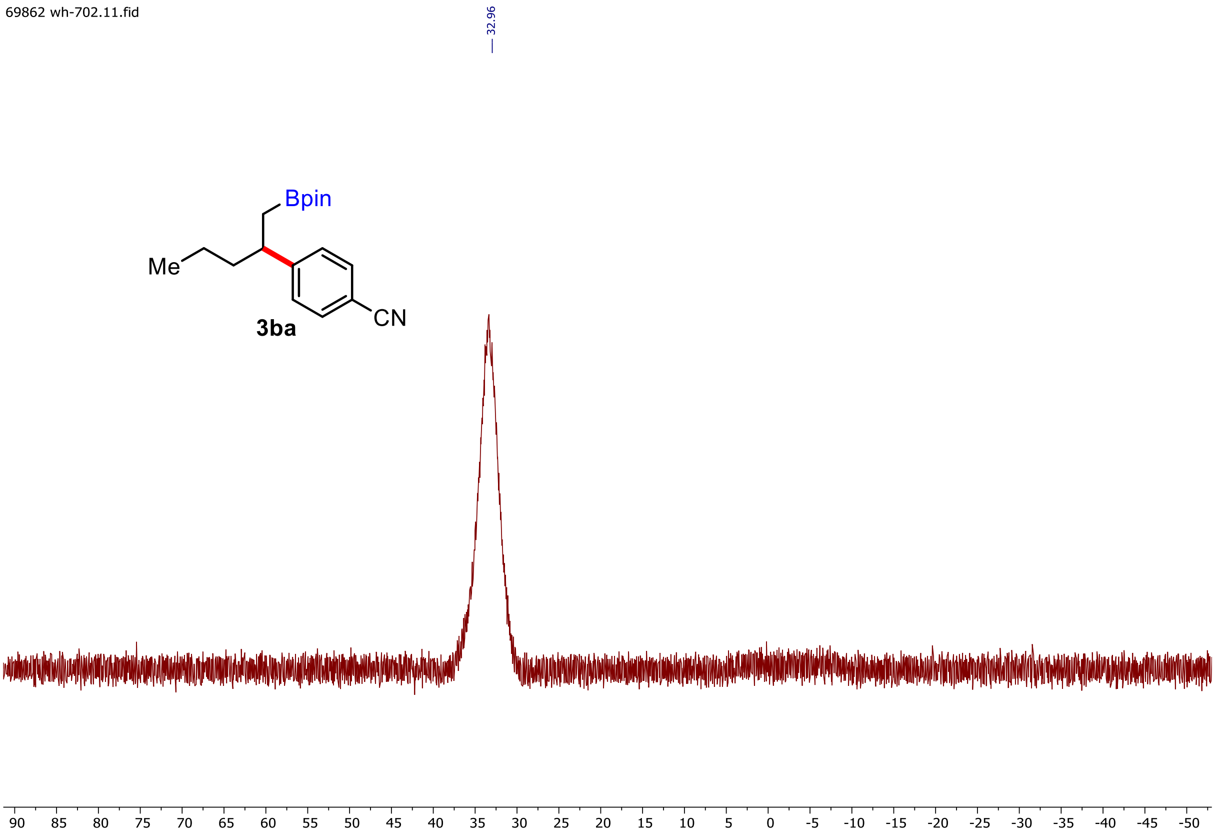

^{1}H NMR (400 MHz, $CDCl_3$) of **3ca** (see procedure)

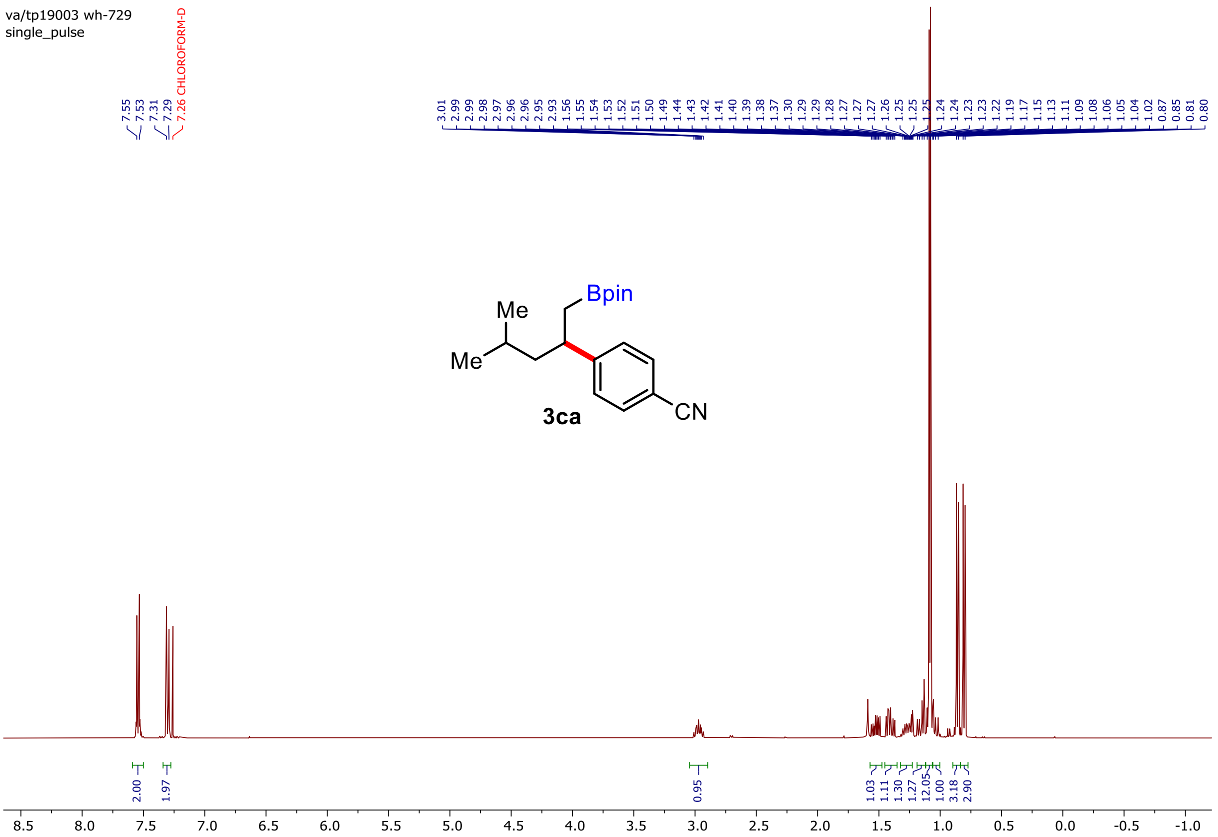

^{13}C NMR (101 MHz, $CDCl_3$) of **3ca**

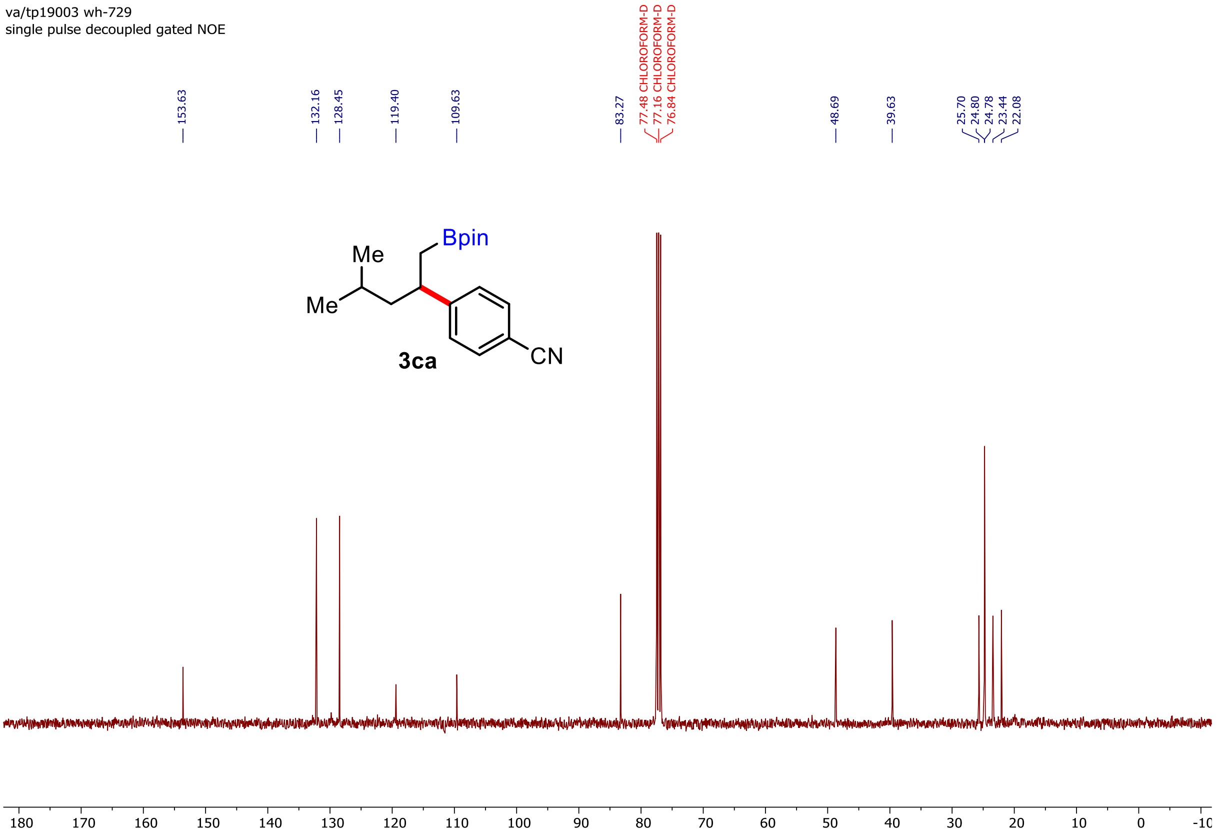

^{11}B NMR (128 MHz, $CDCl_3$) of **3ca**

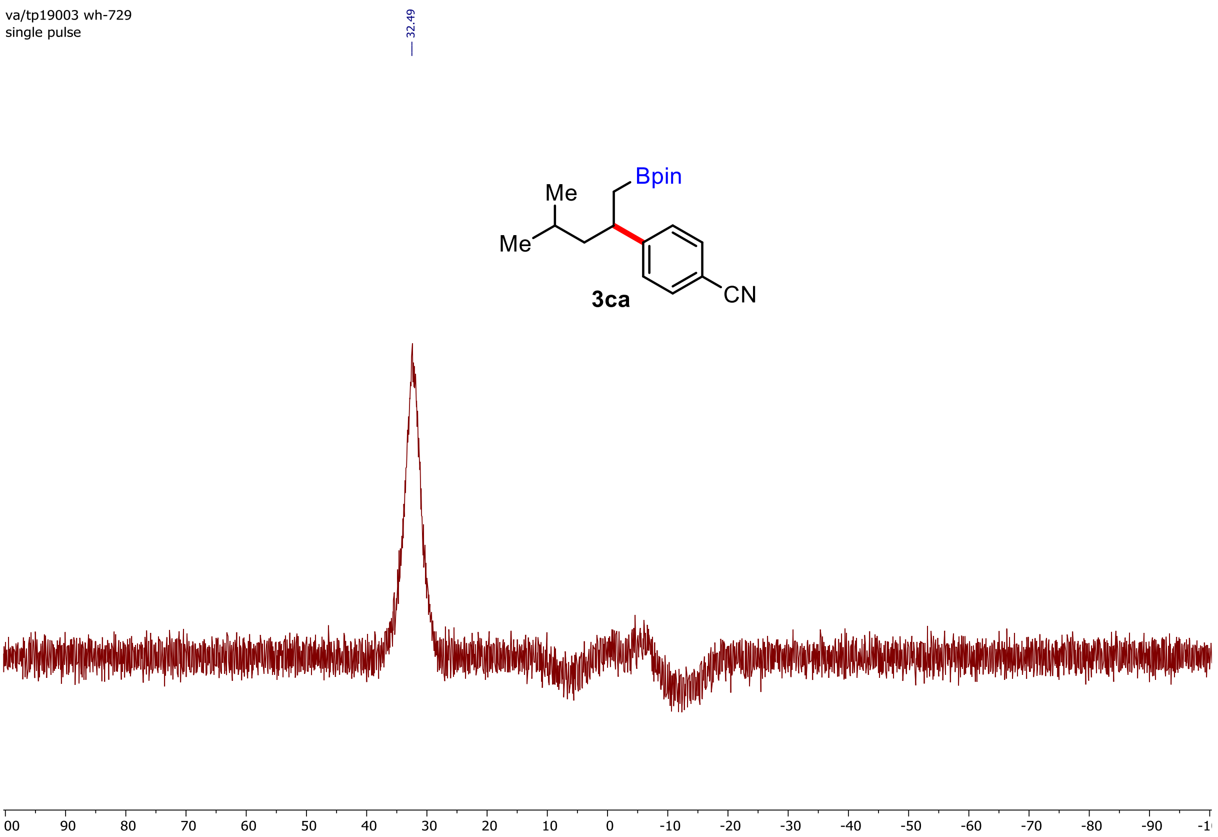

^{1}H NMR (400 MHz, $CDCl_3$) of **3da** (*see procedure*)

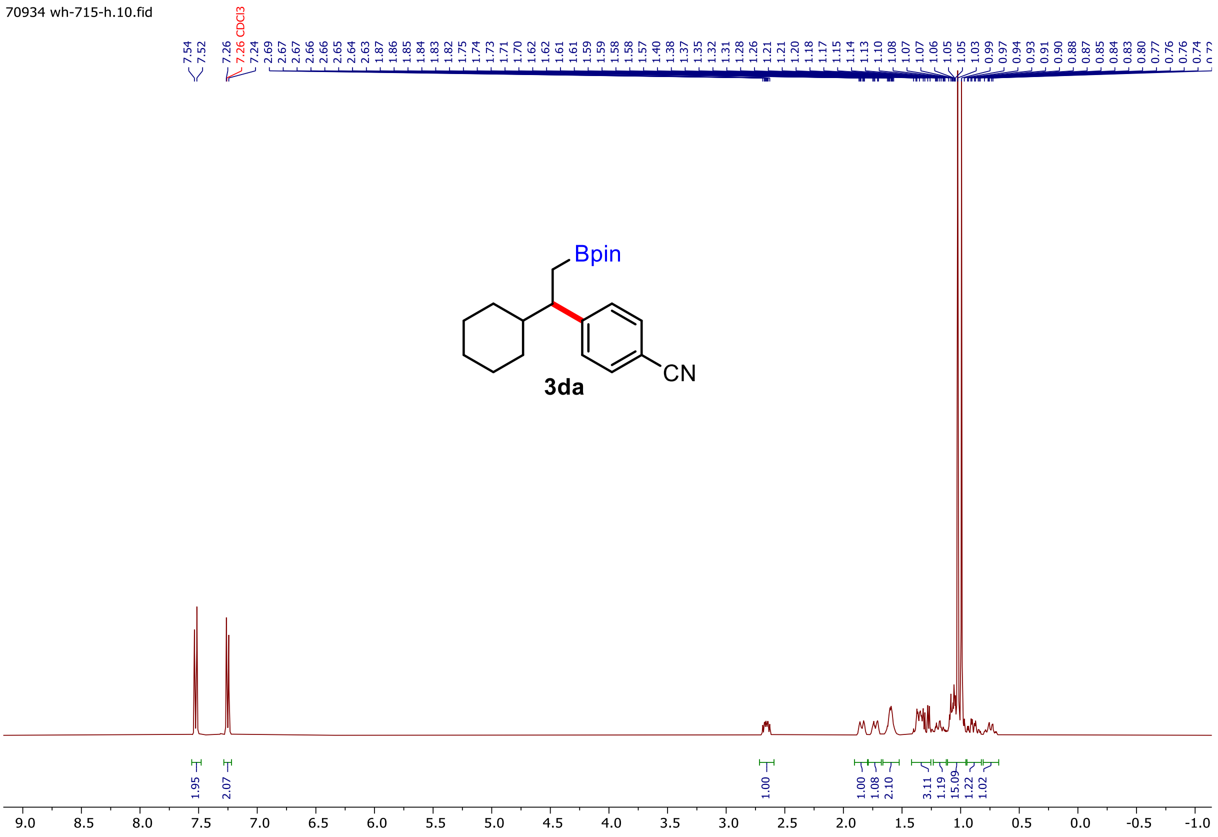

^{13}C NMR (101 MHz, $CDCl_3$) of **3da**

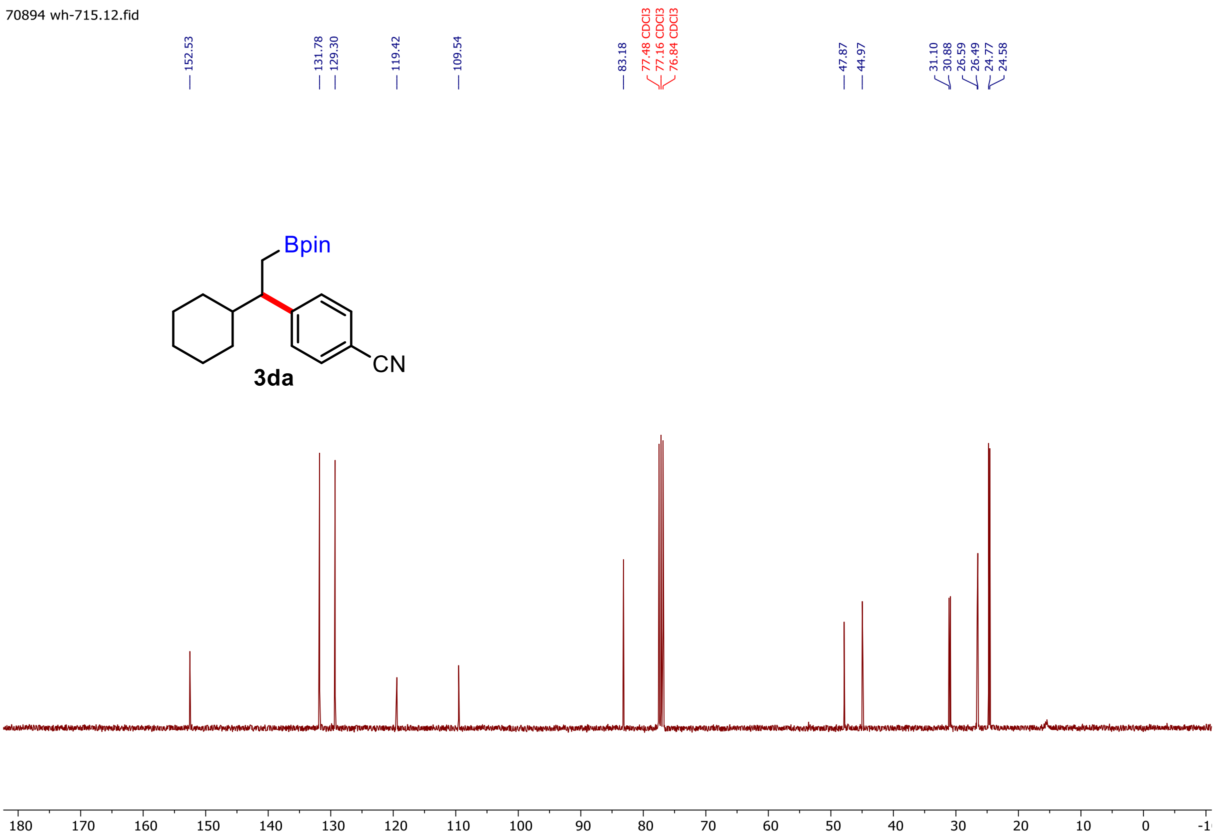

^{11}B NMR (128 MHz, $CDCl_3$) of **3da**

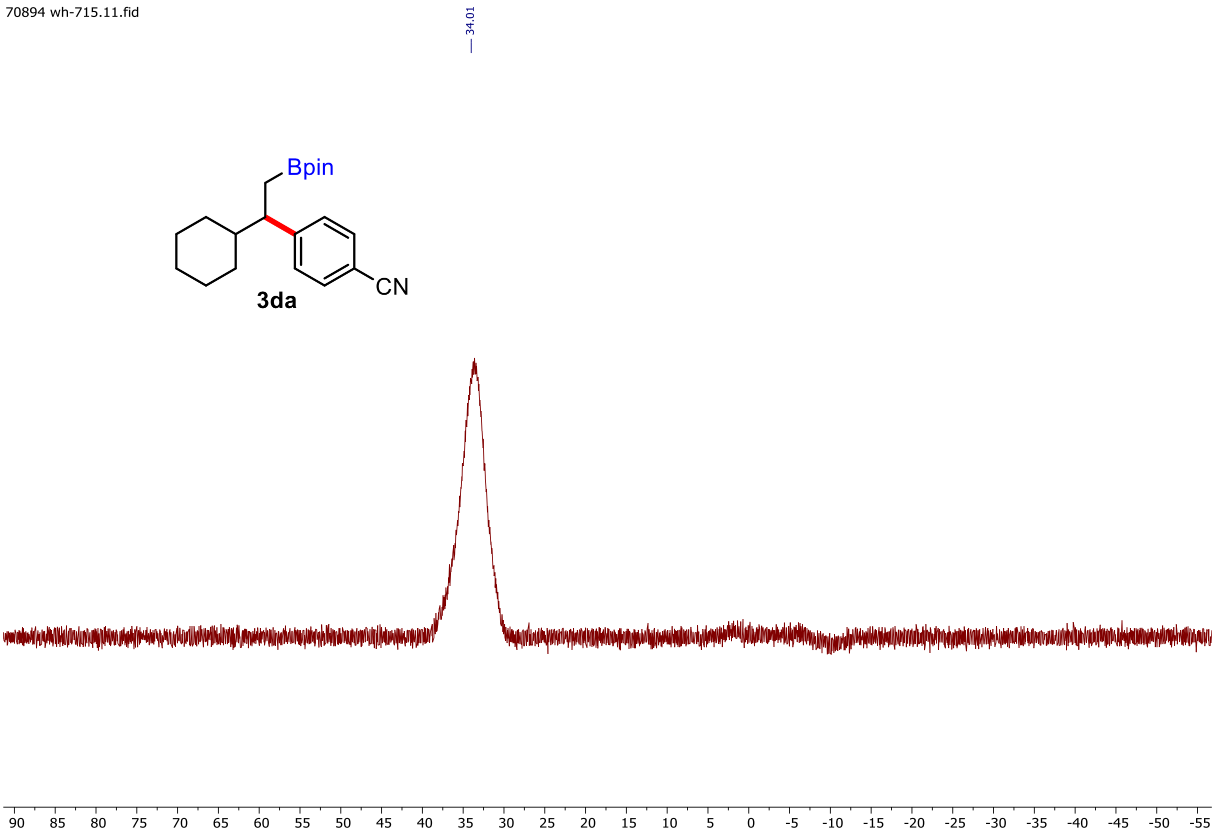

^{1}H NMR (400 MHz, $CDCl_3$) of **3ea** (*see procedure*)

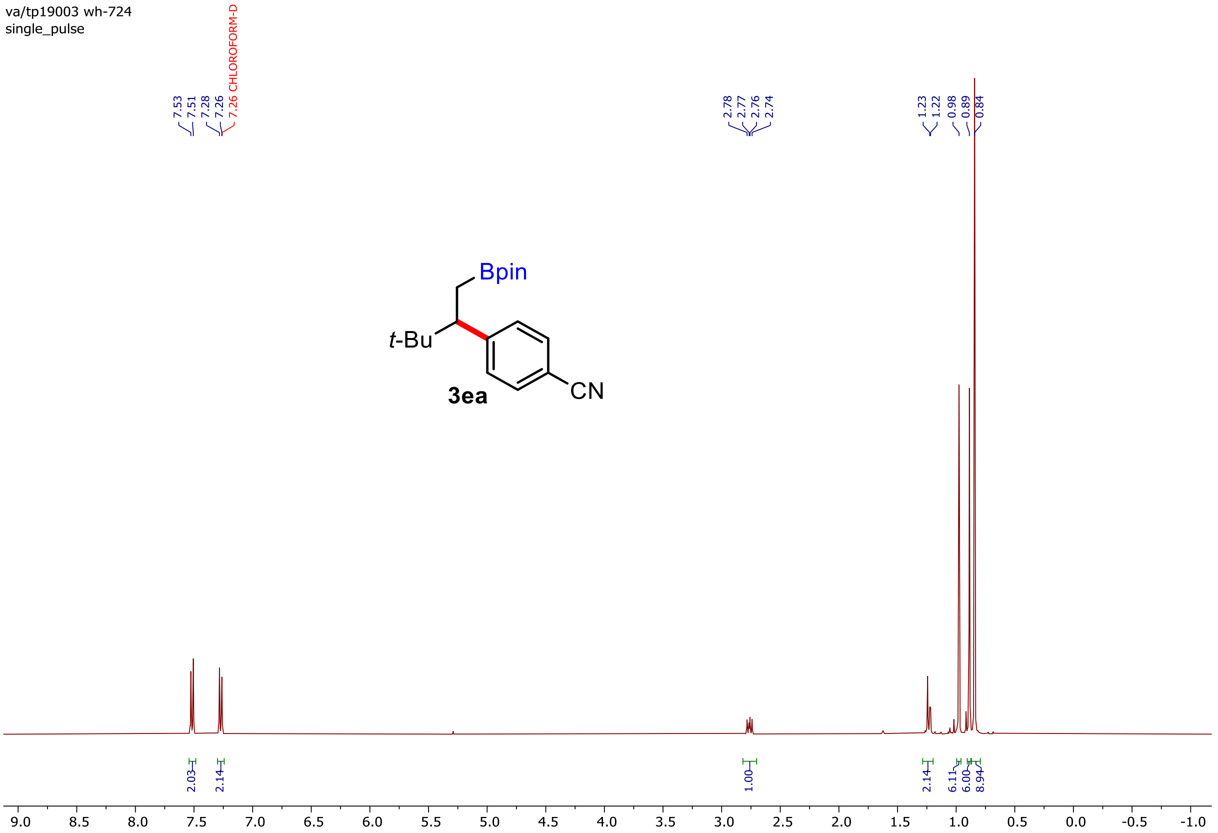

^{13}C NMR (101 MHz, $CDCl_3$) of **3ea**

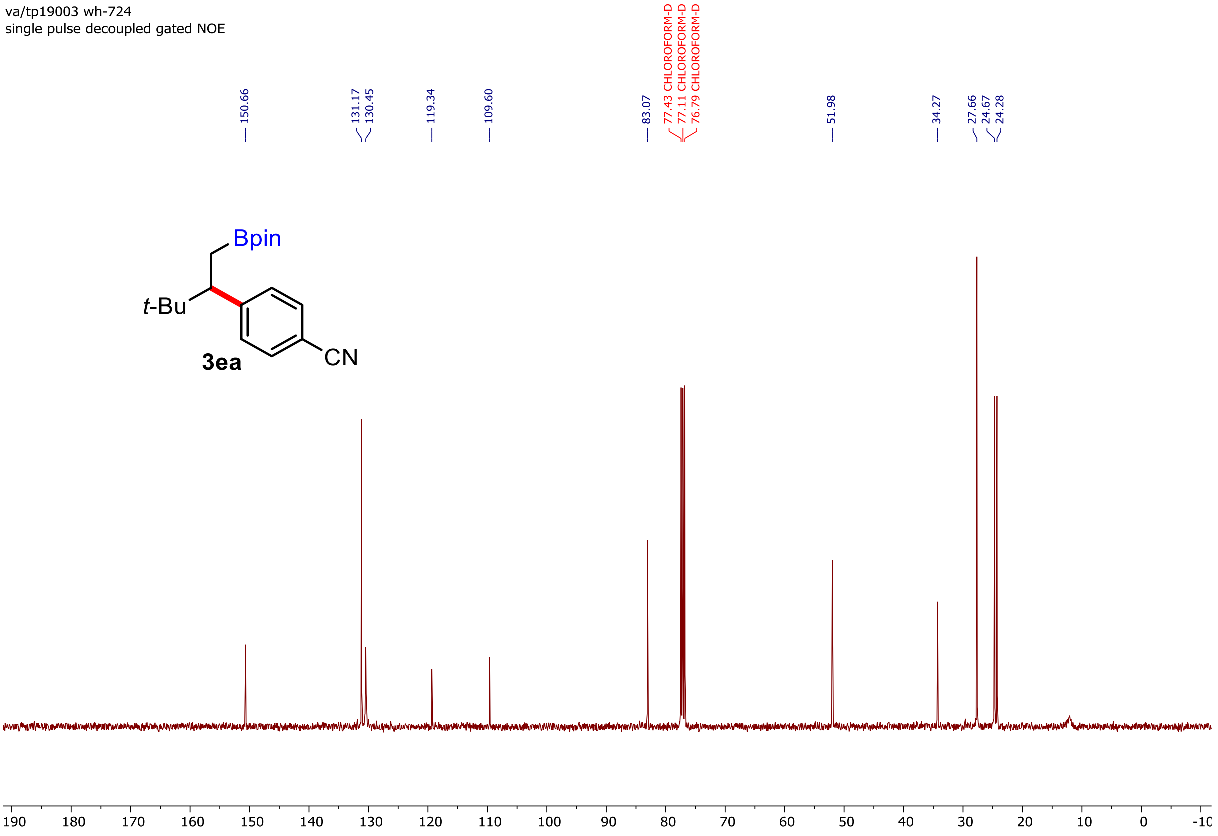

UOB Confidential & Sensitive

^{11}B NMR (128 MHz, CDCl$_3$) of **3ea**

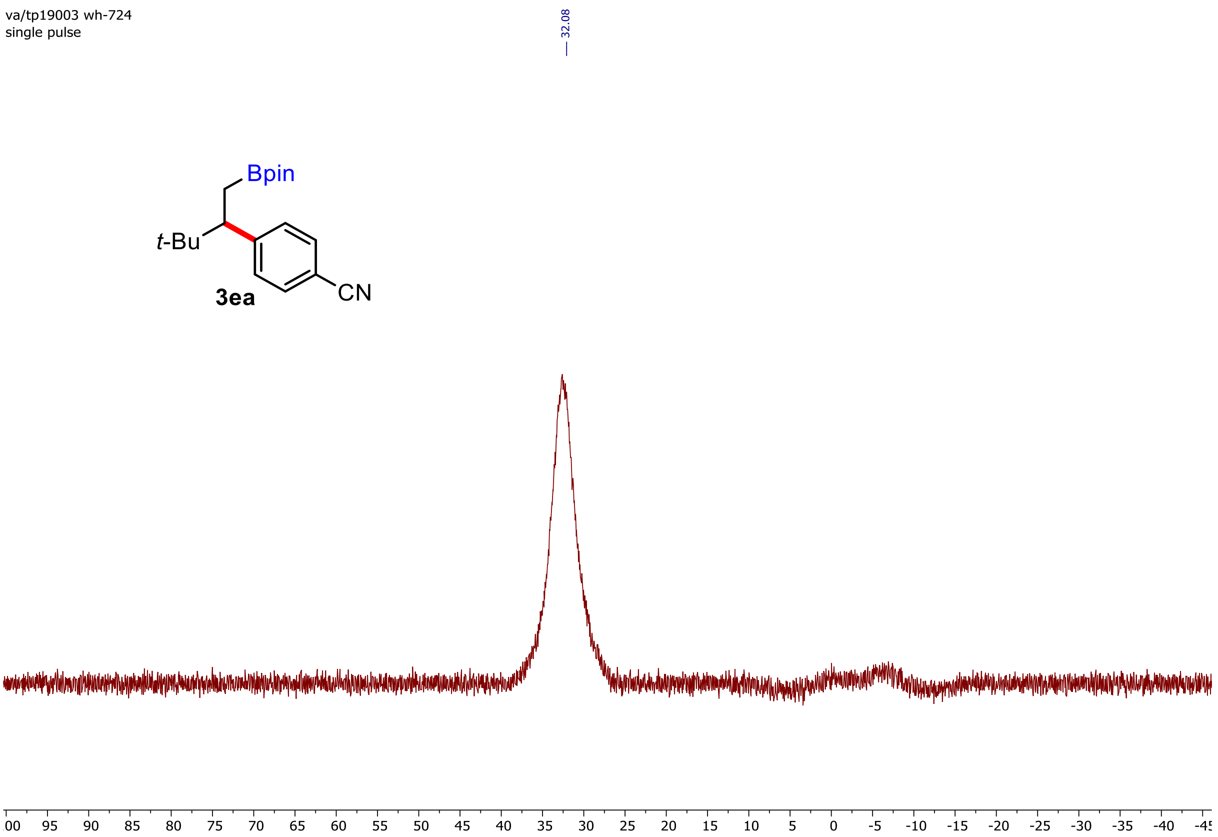

^{1}H NMR (400 MHz, CDCl$_3$) of **3fa** (*see procedure*)

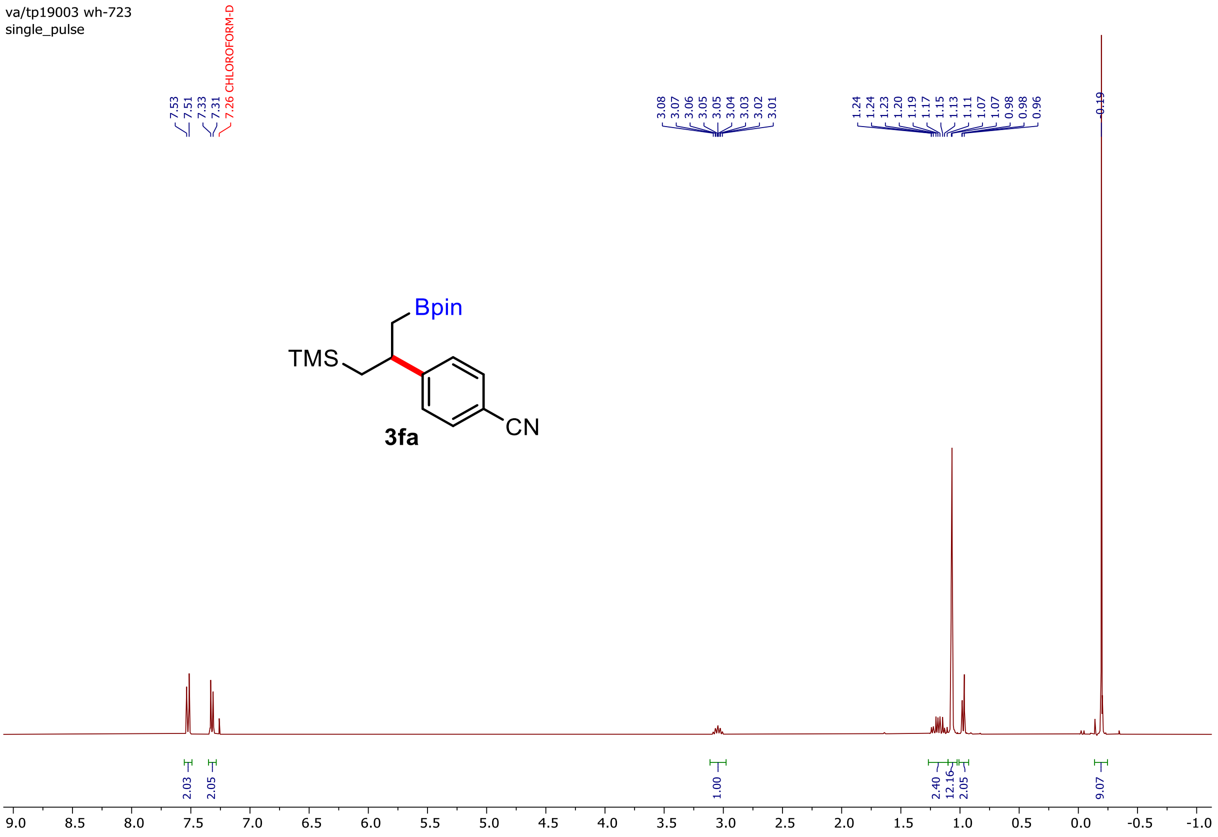

^{13}C NMR (101 MHz, $CDCl_3$) of **3fa**

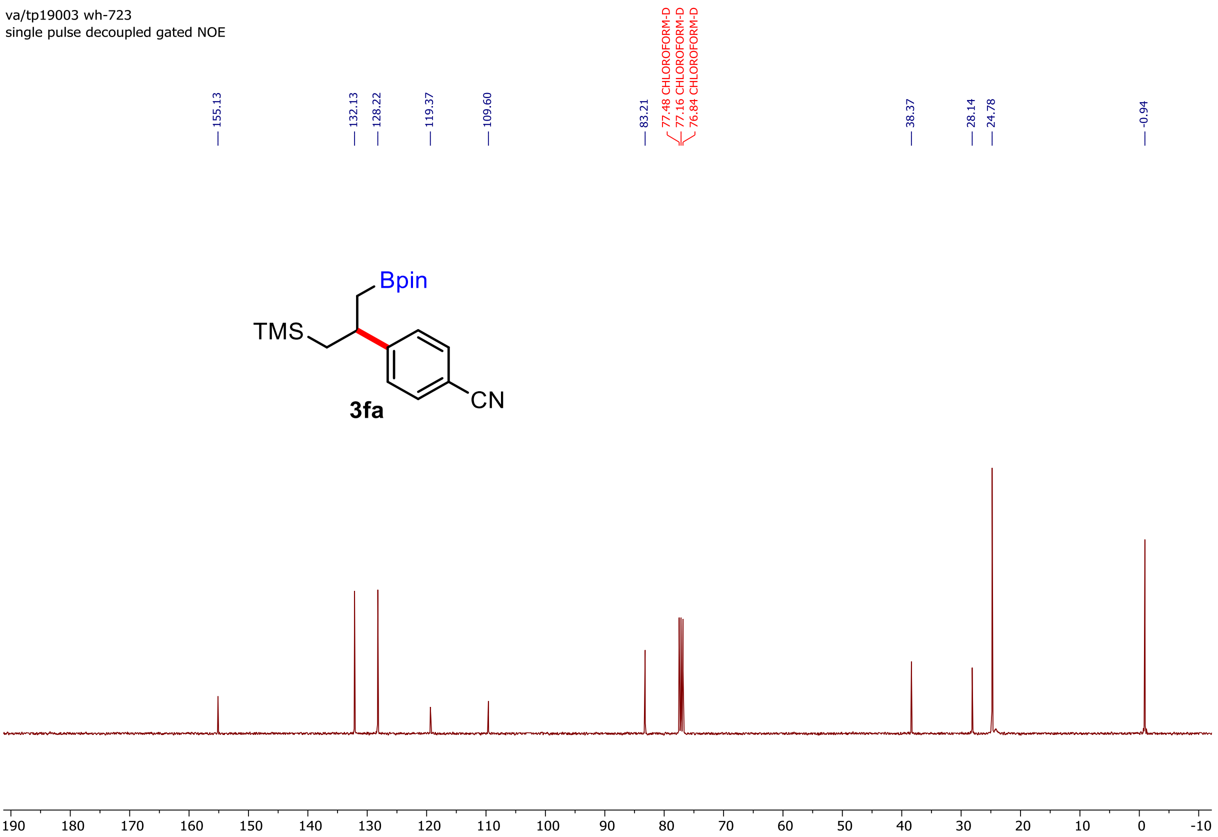

^{11}B NMR (128 MHz, $CDCl_3$) of **3fa**

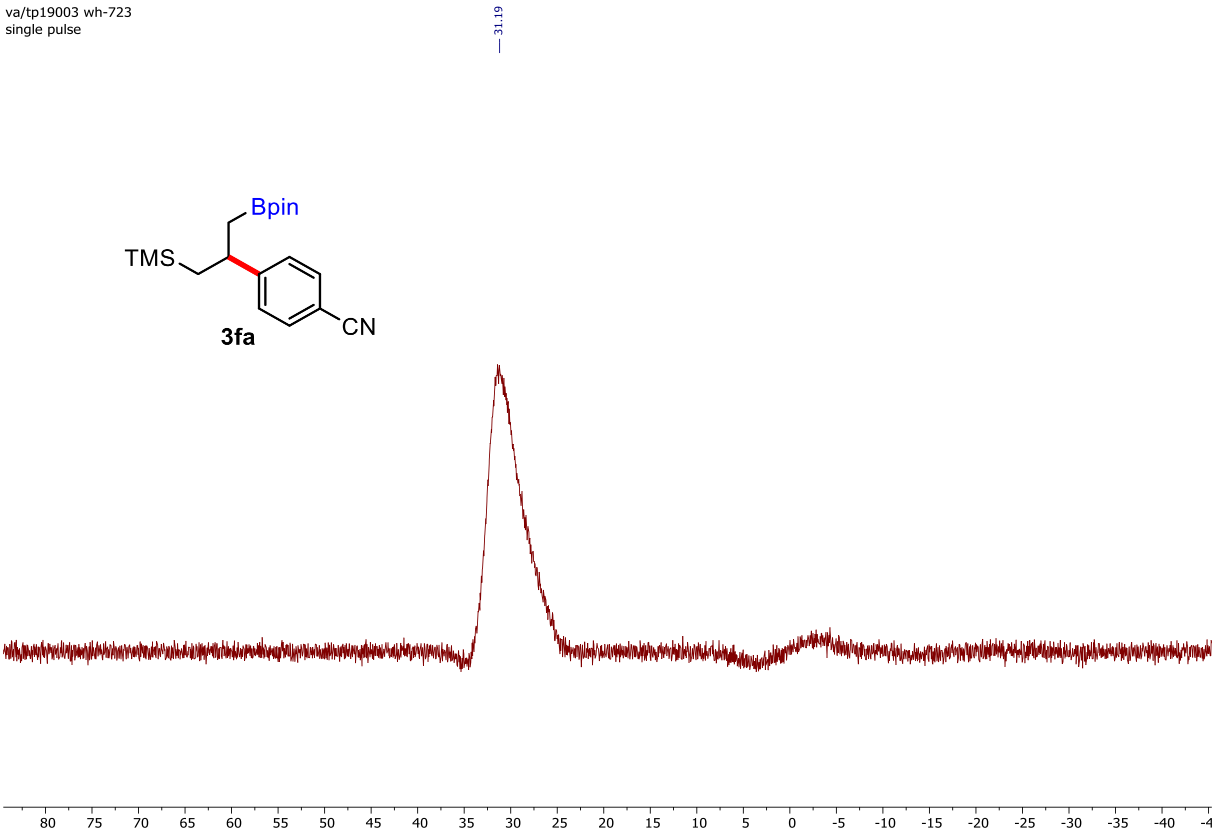

^{1}H NMR (400 MHz, $CDCl_3$) of **3ga** (*see procedure*)

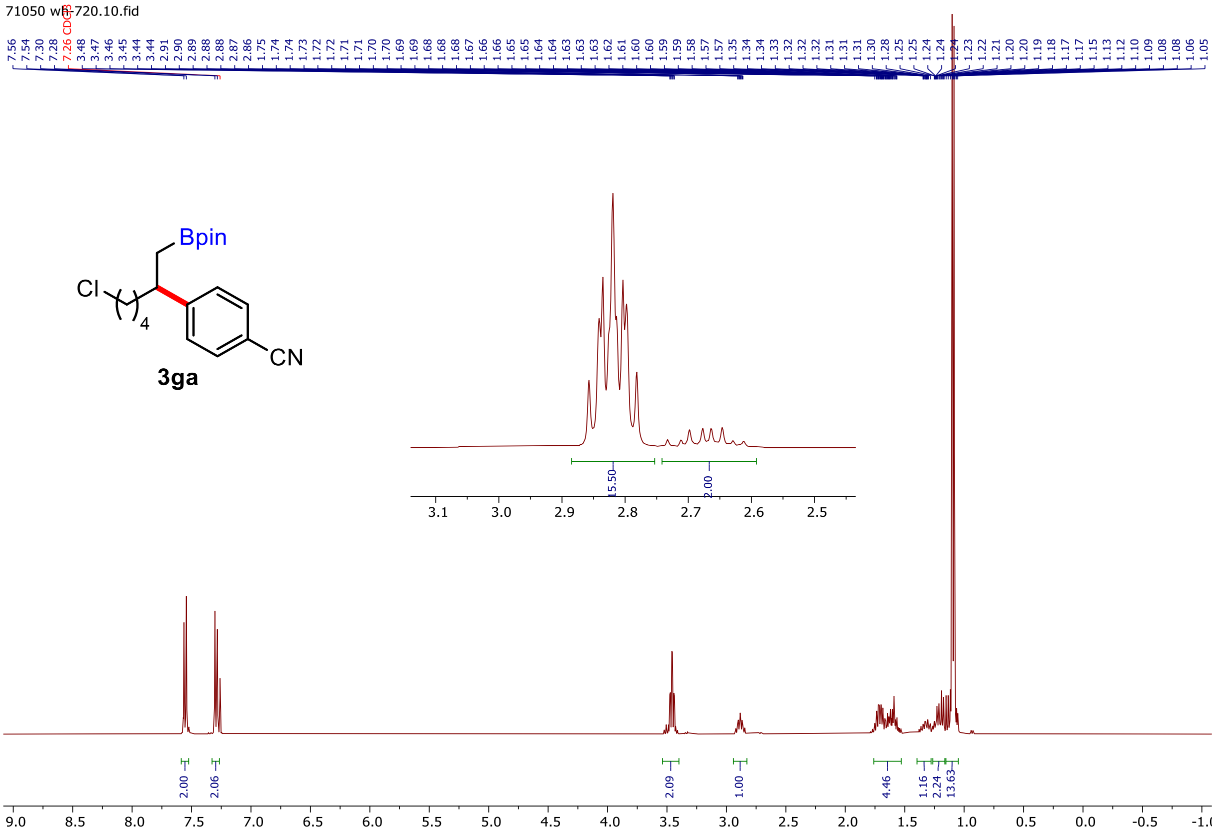

^{13}C NMR (101 MHz, $CDCl_3$) of **3ga**

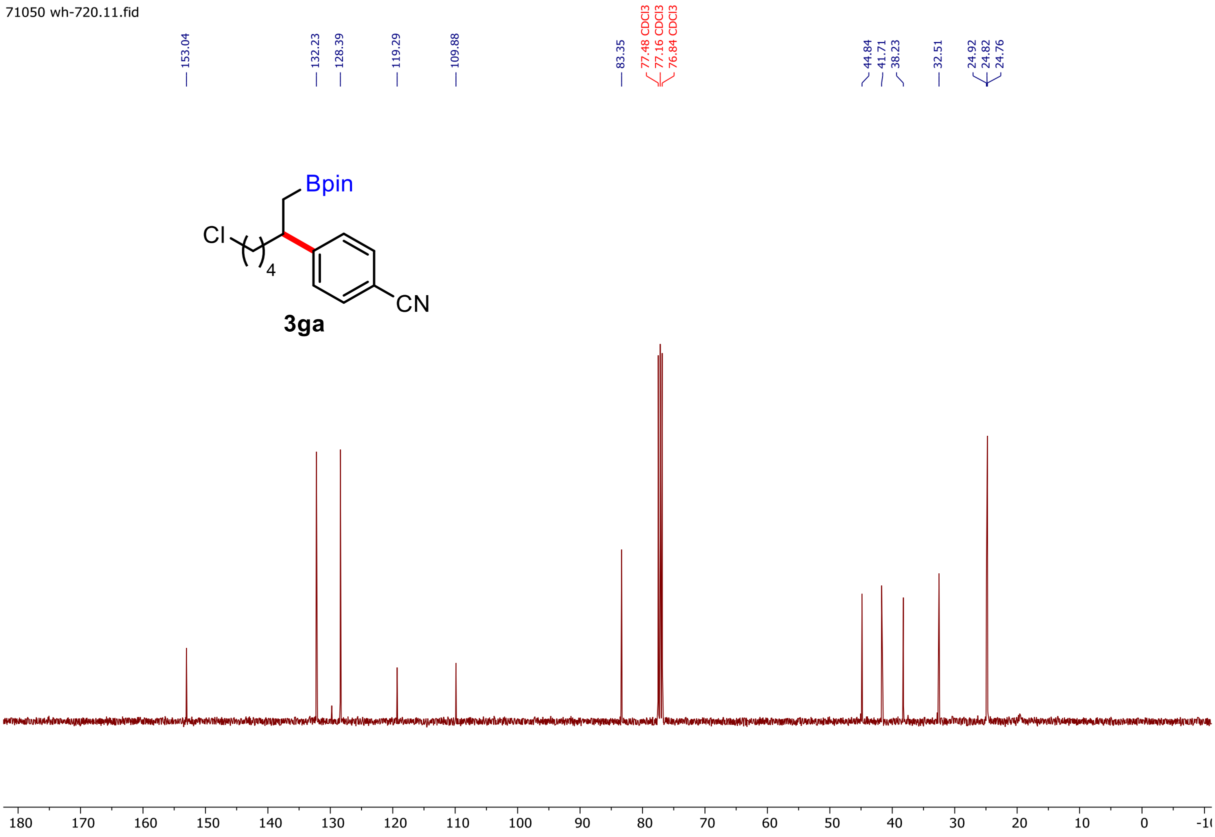

^{11}B NMR (128 MHz, $CDCl_3$) of **3ga**

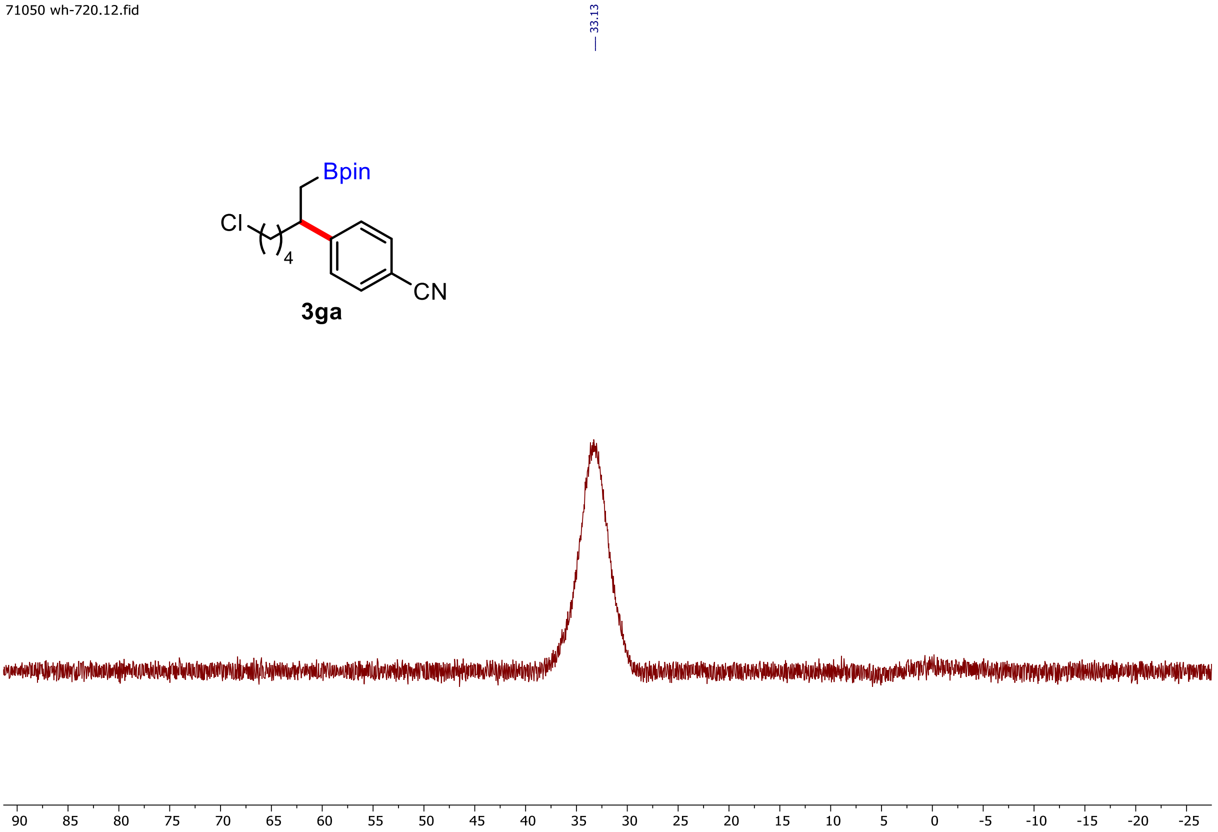

^{1}H NMR (400 MHz, $CDCl_3$) of **3ha** (*see procedure*)

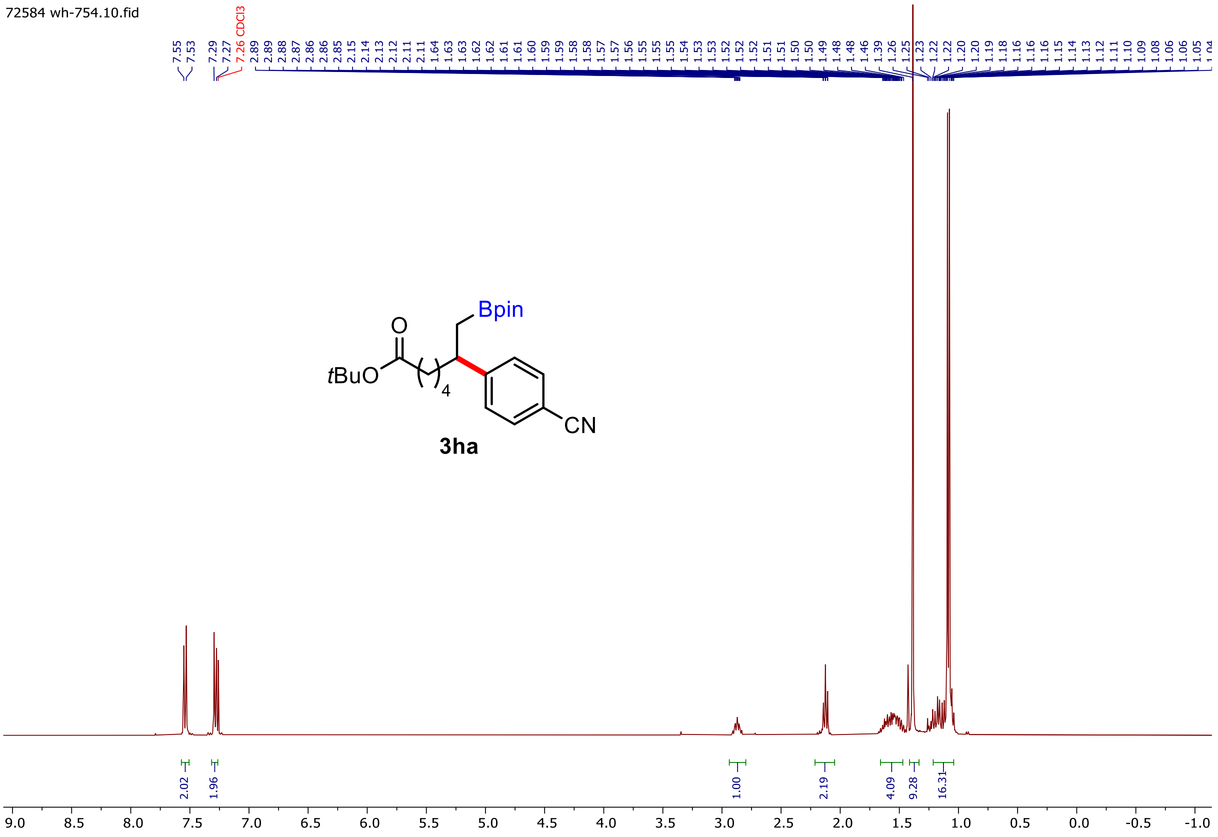

^{13}C NMR (101 MHz, $CDCl_3$) of **3ha**

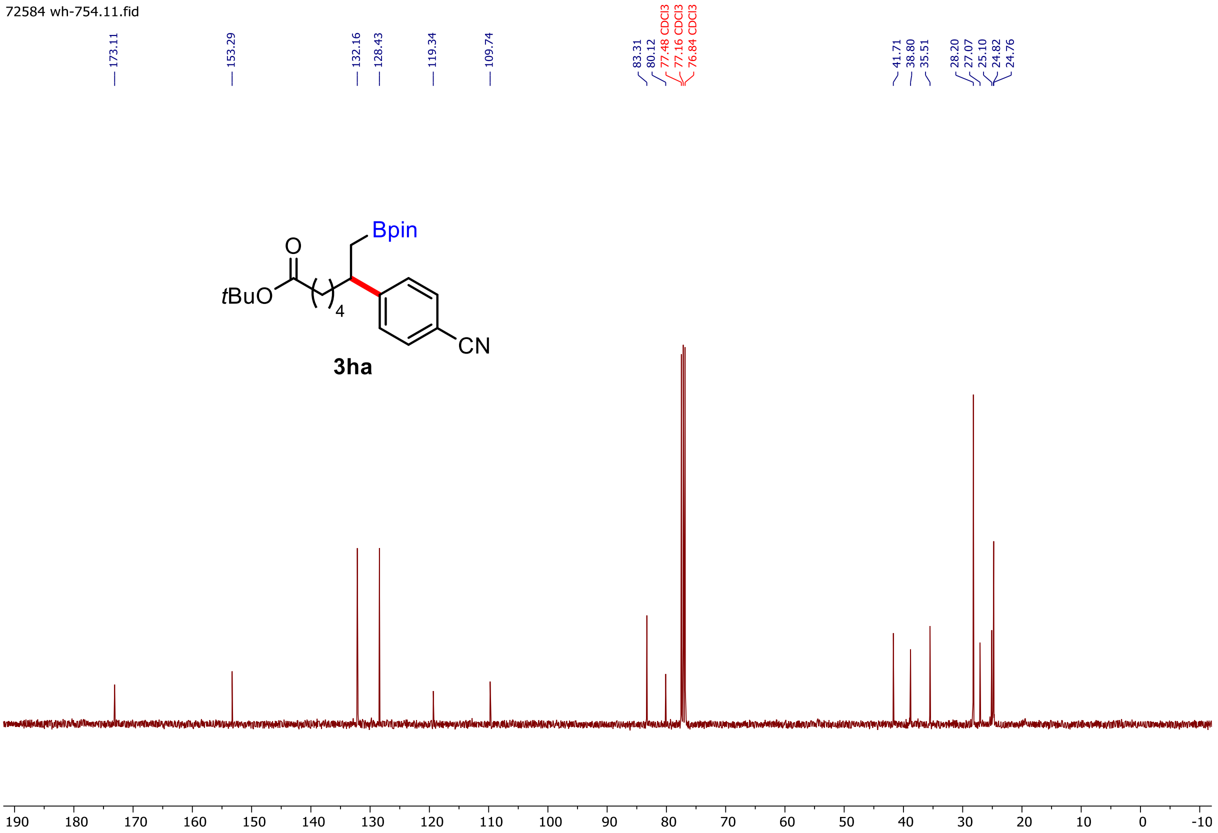

^{11}B NMR (128 MHz, $CDCl_3$) of **3ha**

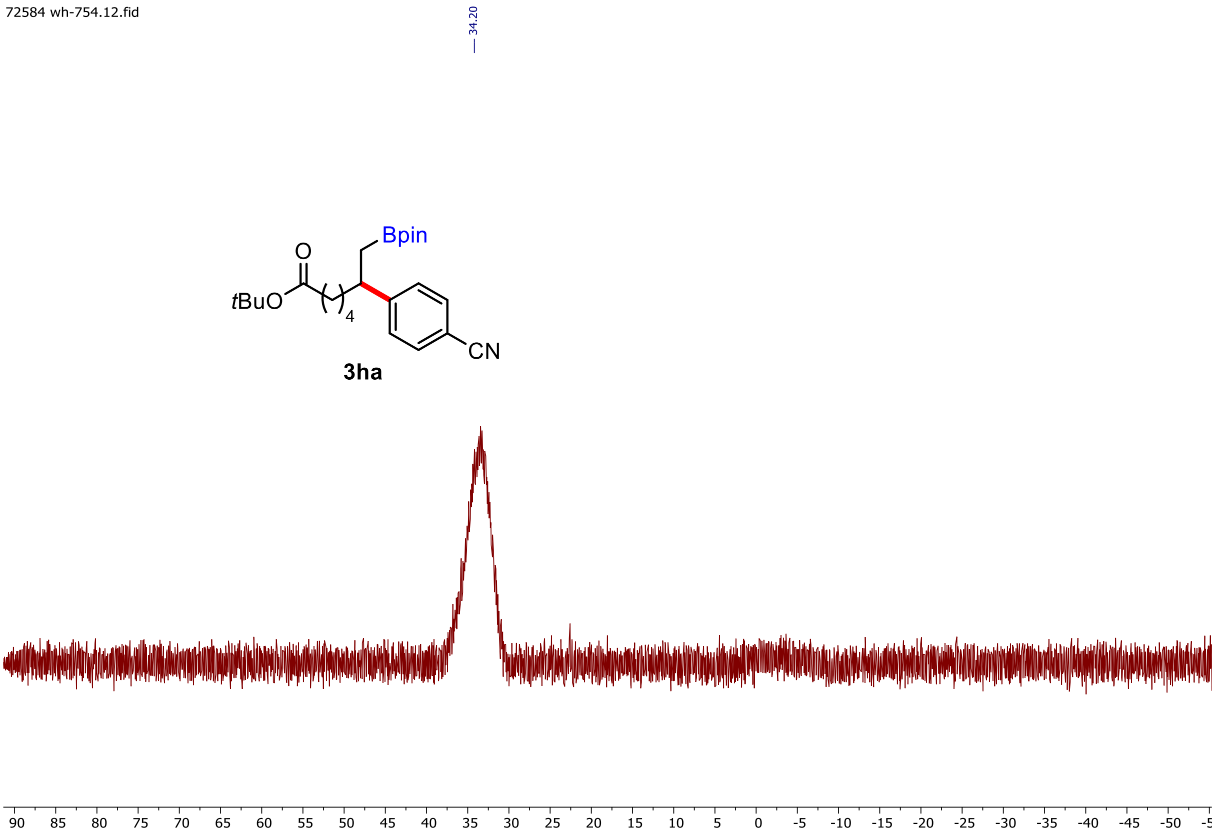

^{1}H NMR (400 MHz, $CDCl_3$) of **3ia** (*see procedure*)

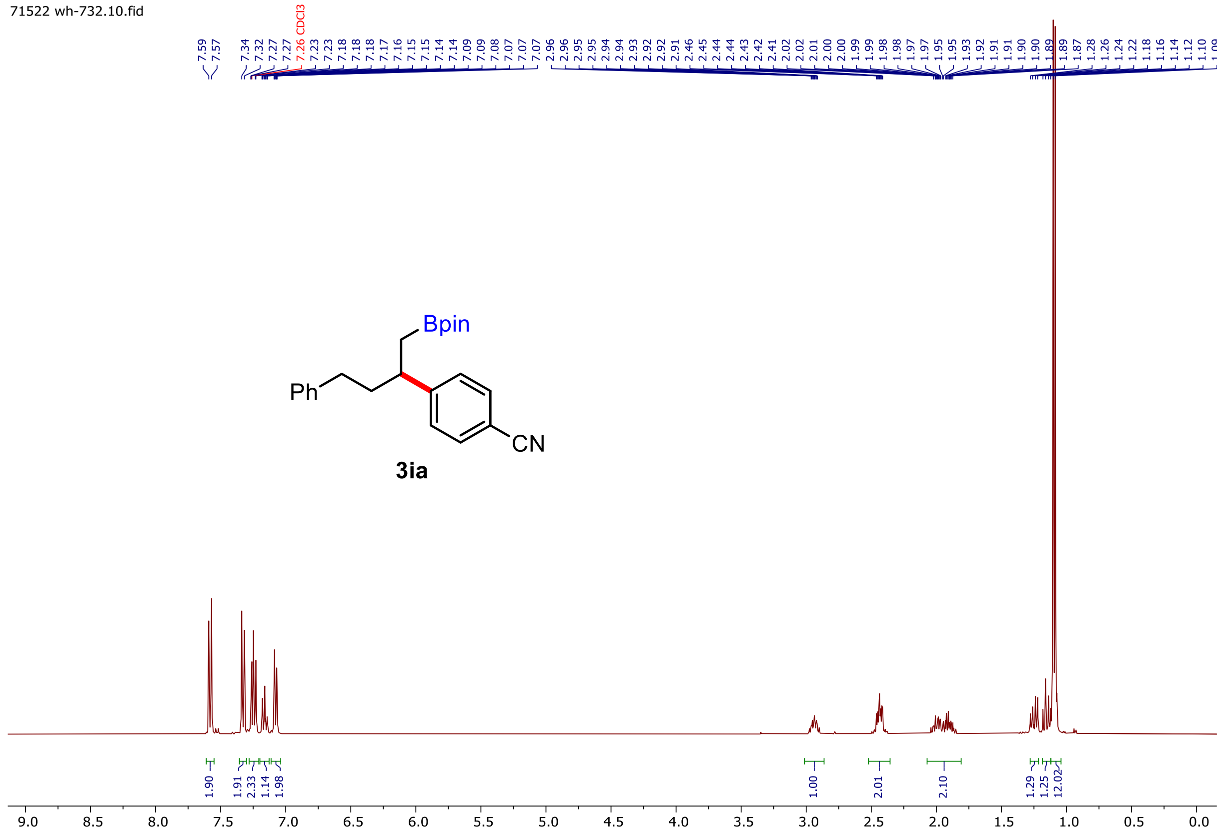

^{13}C NMR (101 MHz, $CDCl_3$) of **3ia**

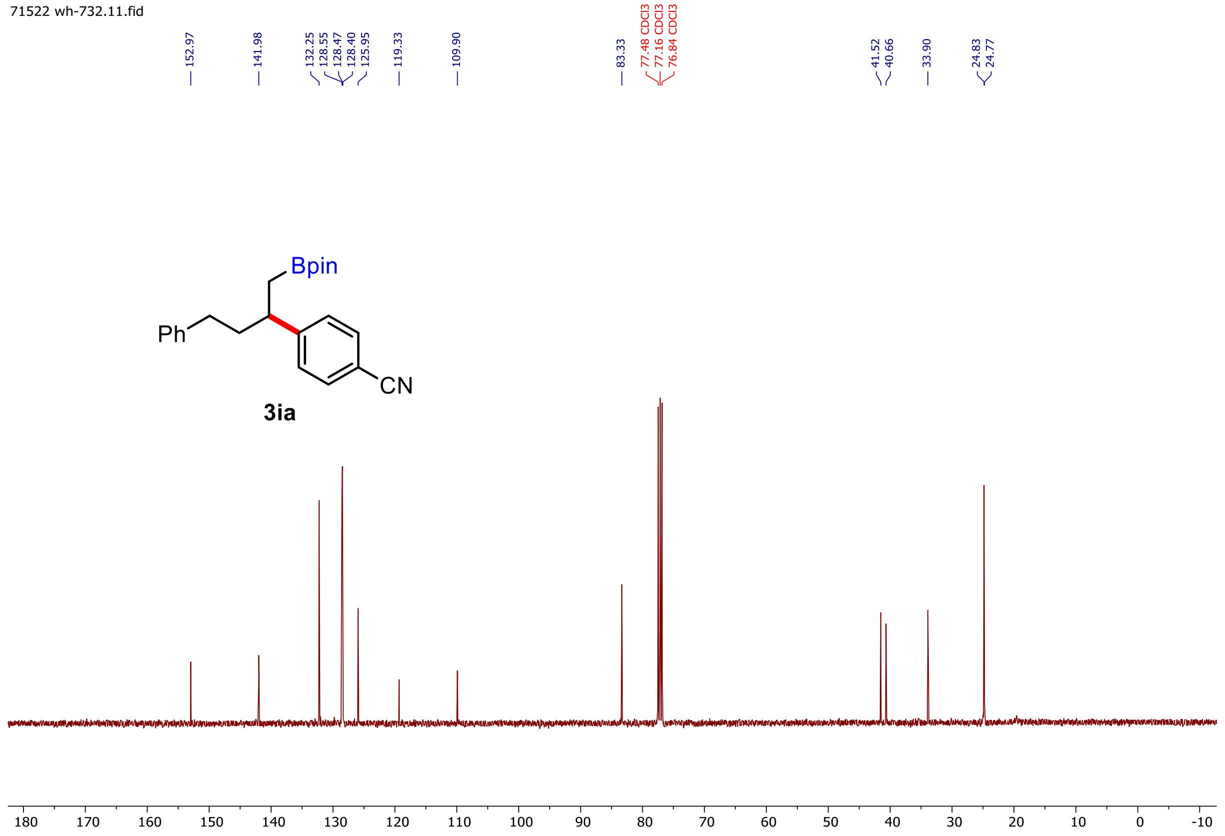

^{11}B NMR (128 MHz, $CDCl_3$) of **3ia**

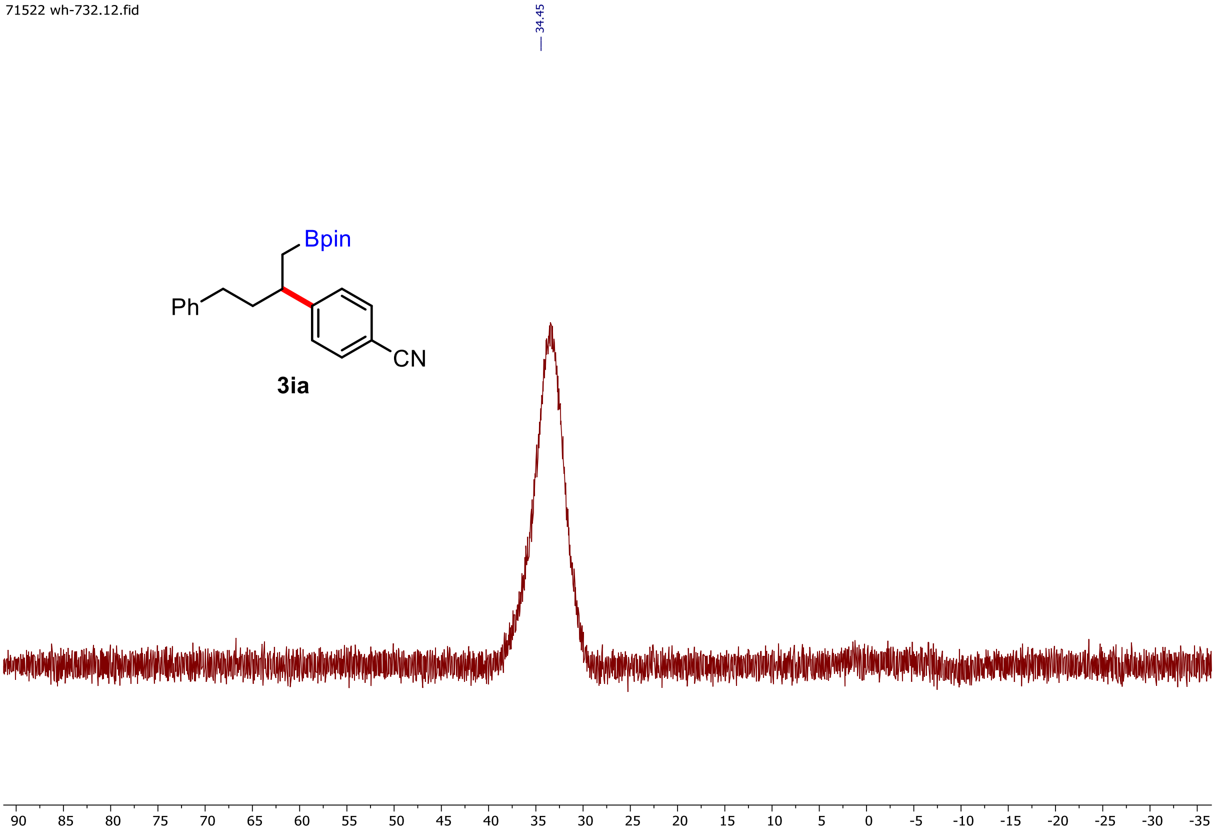

^{1}H NMR (400 MHz, $CDCl_3$) of **3ja** (*see procedure*)

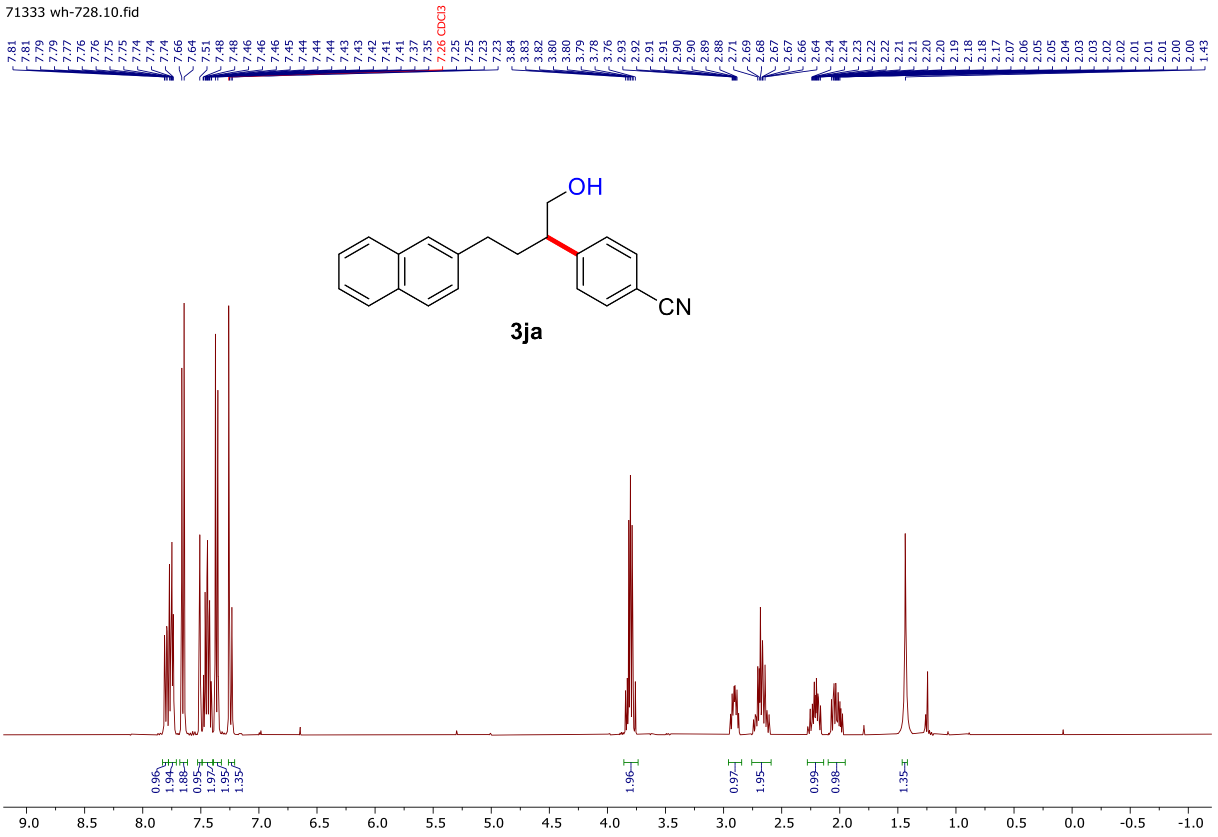

UOB Confidential & Sensitive

^{13}C NMR (101 MHz, $CDCl_3$) of **3ja**

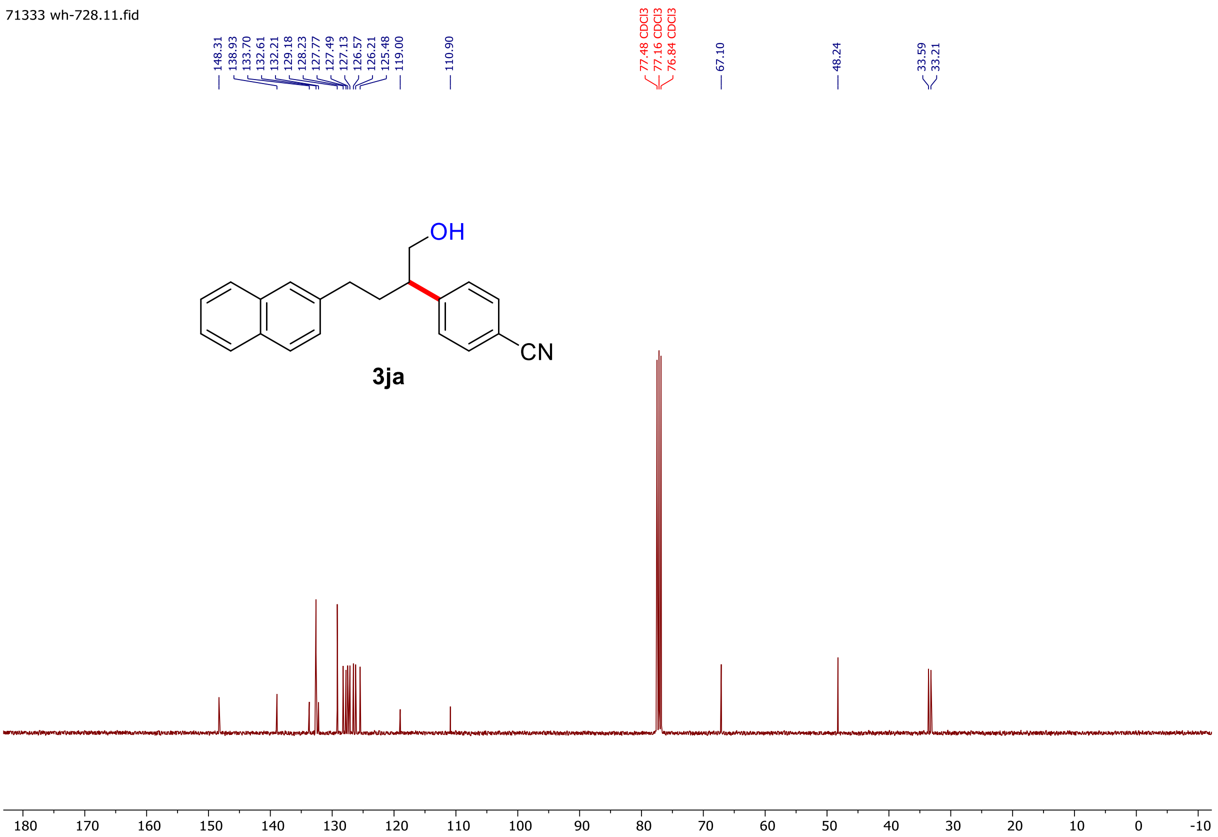

^{1}H NMR (400 MHz, $CDCl_3$) of **3ka** (*see procedure*)

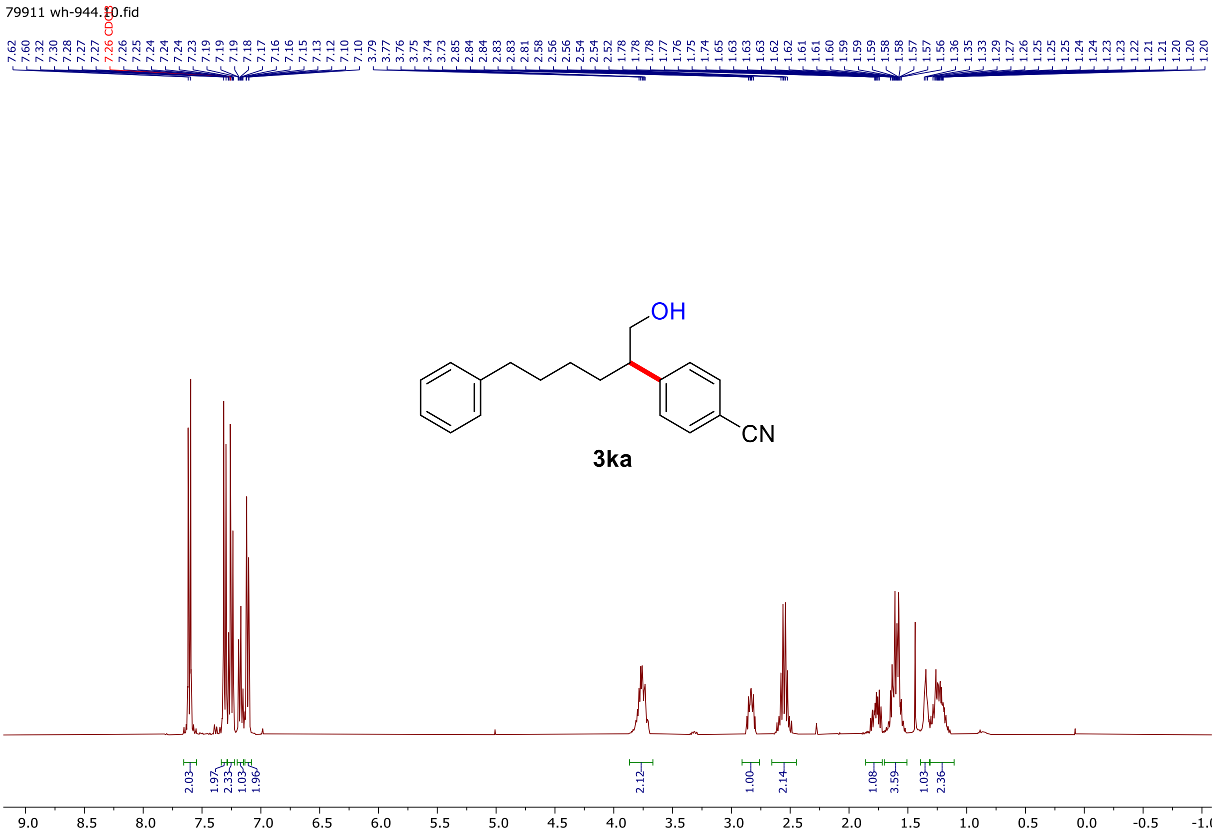

^{13}C NMR (101 MHz, $CDCl_3$) of **3ka**

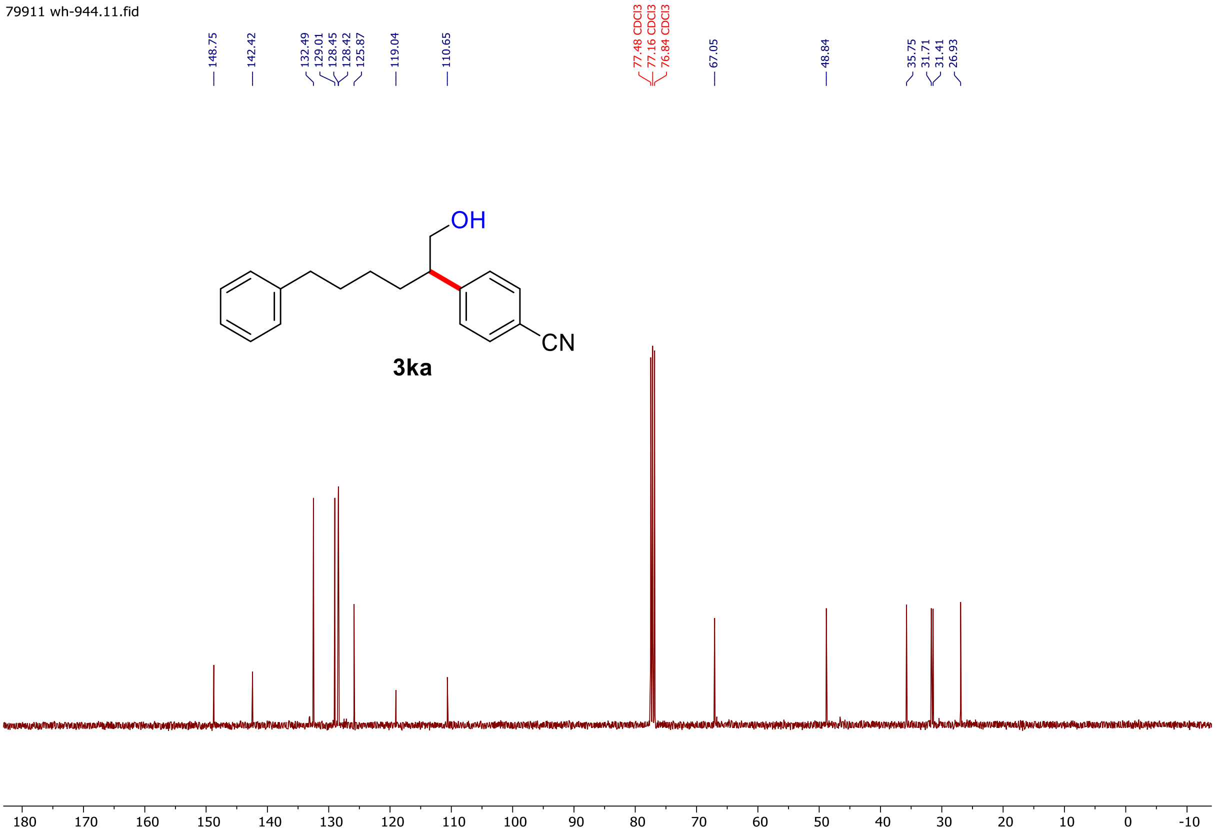

^{1}H NMR (400 MHz, $CDCl_3$) of **3la** (*see procedure*)

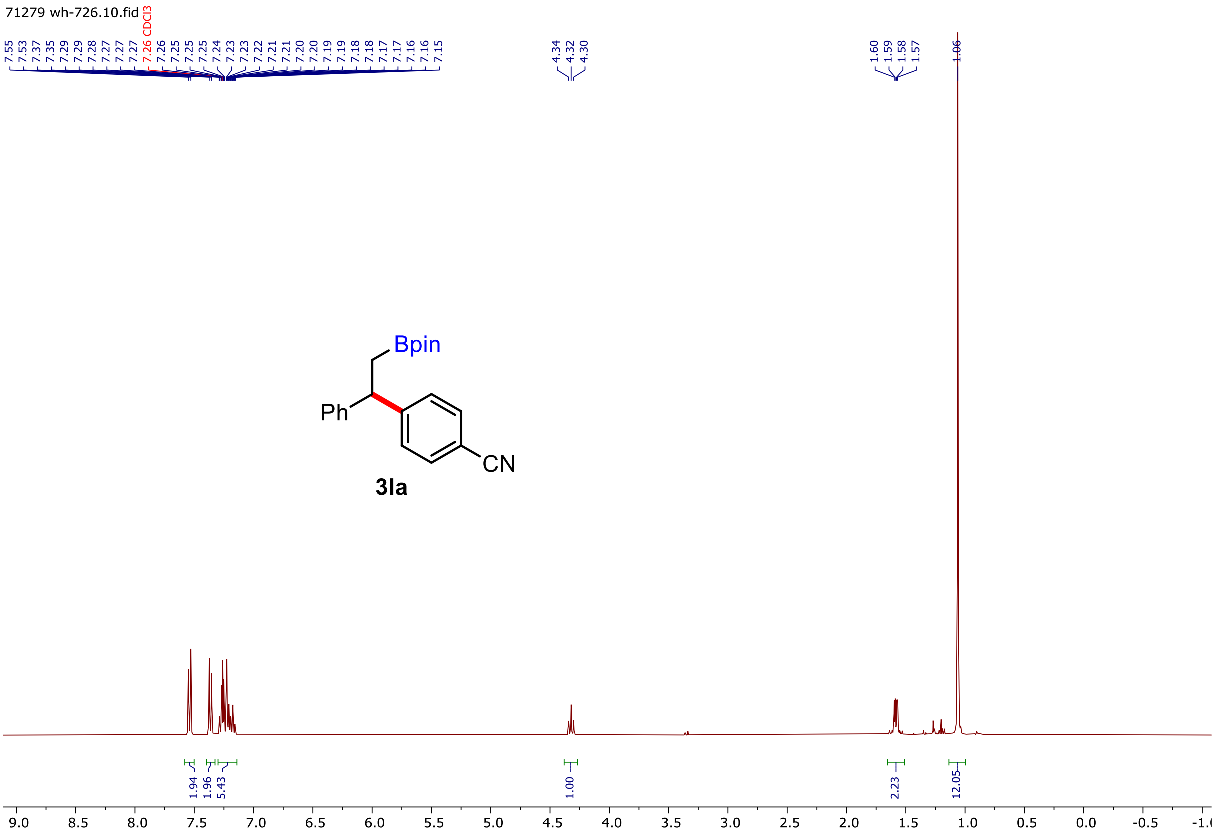

^{13}C NMR (101 MHz, $CDCl_3$) of **3la**

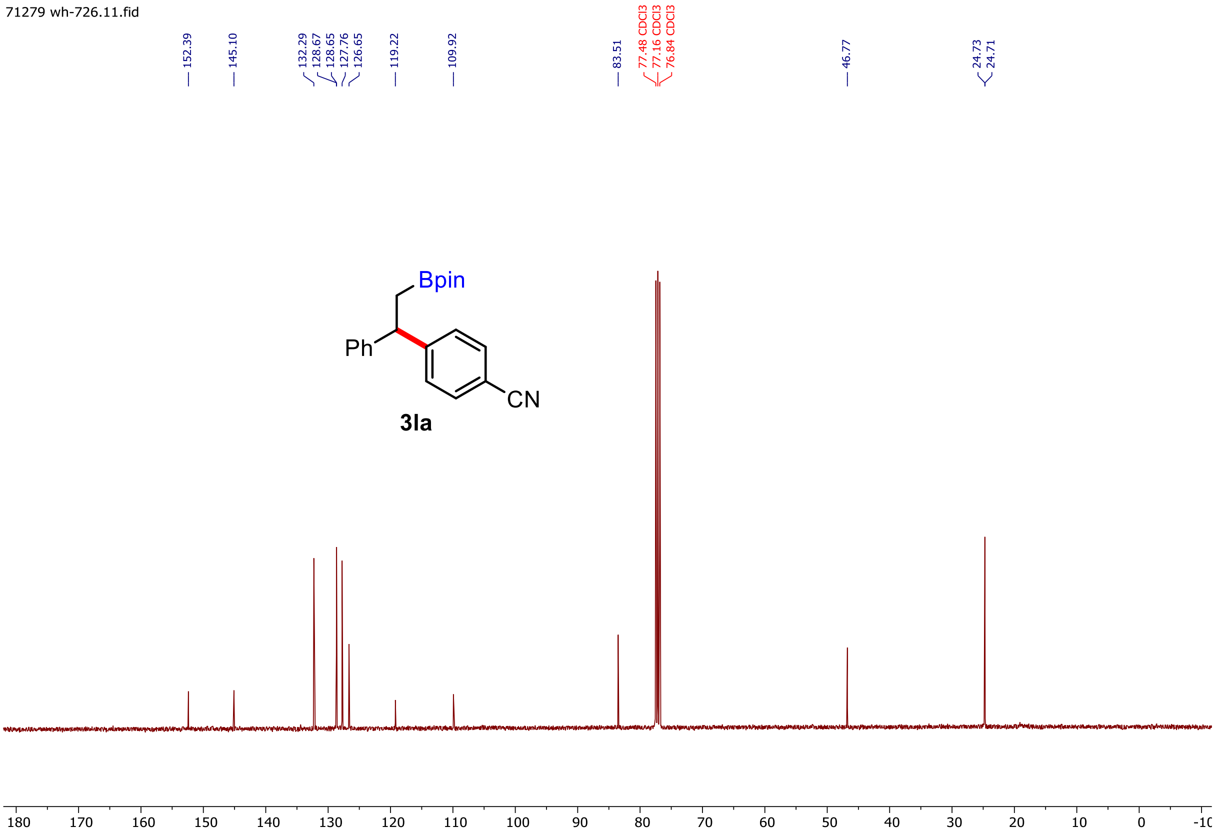

^{11}B NMR (128 MHz, $CDCl_3$) of **3la**

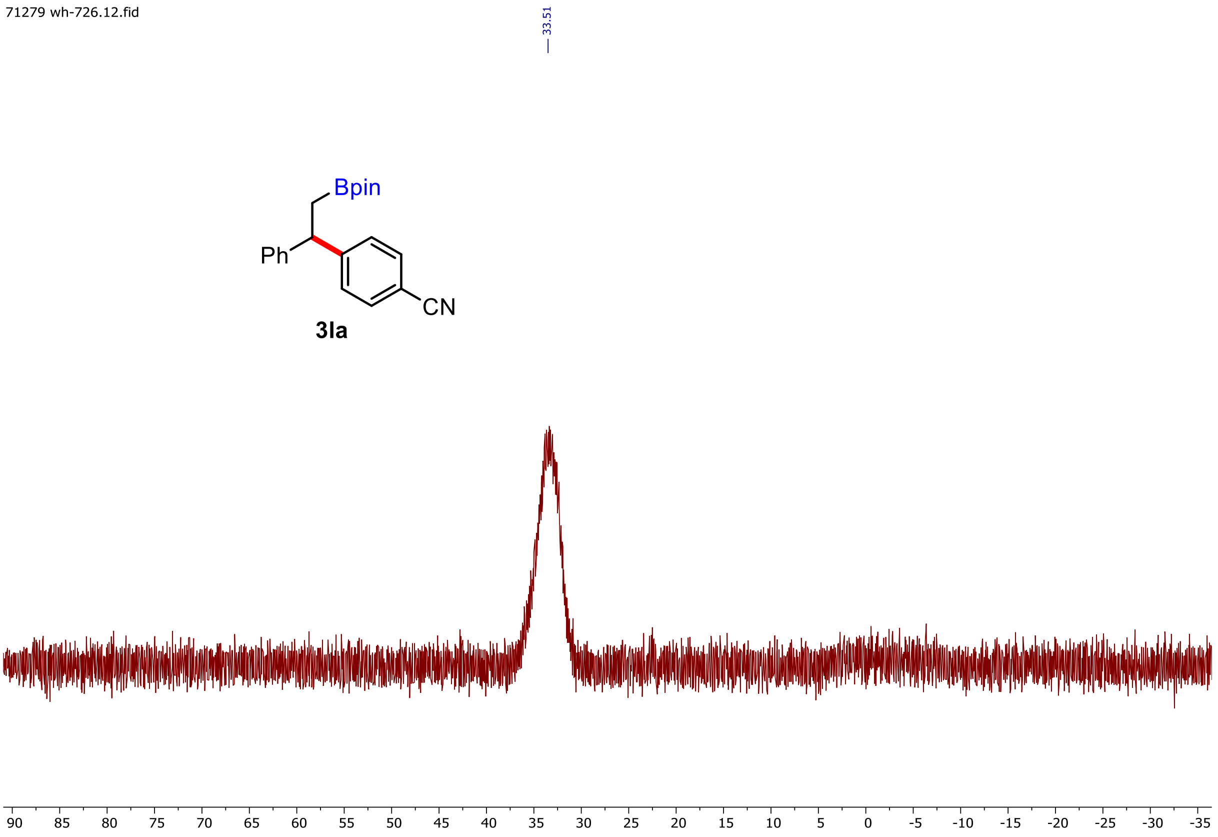

^{1}H NMR (400 MHz, $CDCl_3$) of **3ma** (*see procedure*)

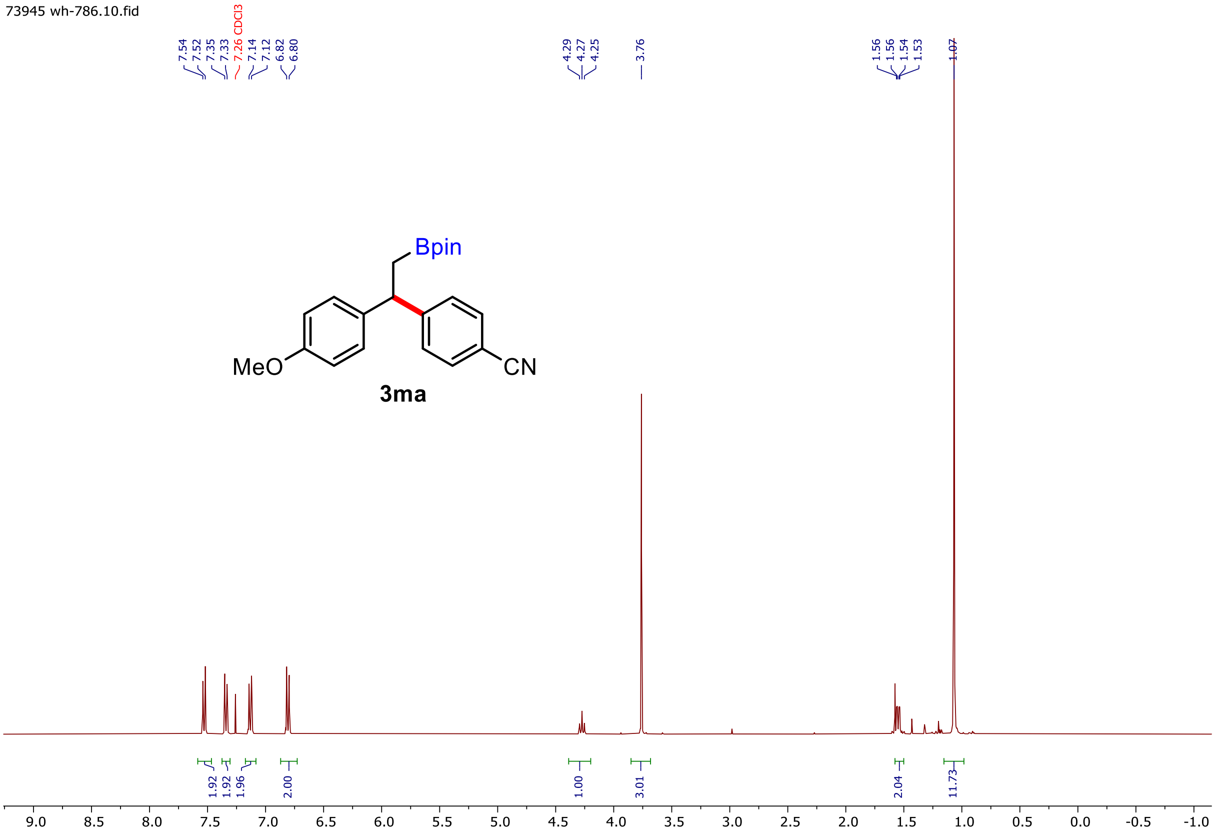

^{13}C NMR (101 MHz, $CDCl_3$) of **3ma**

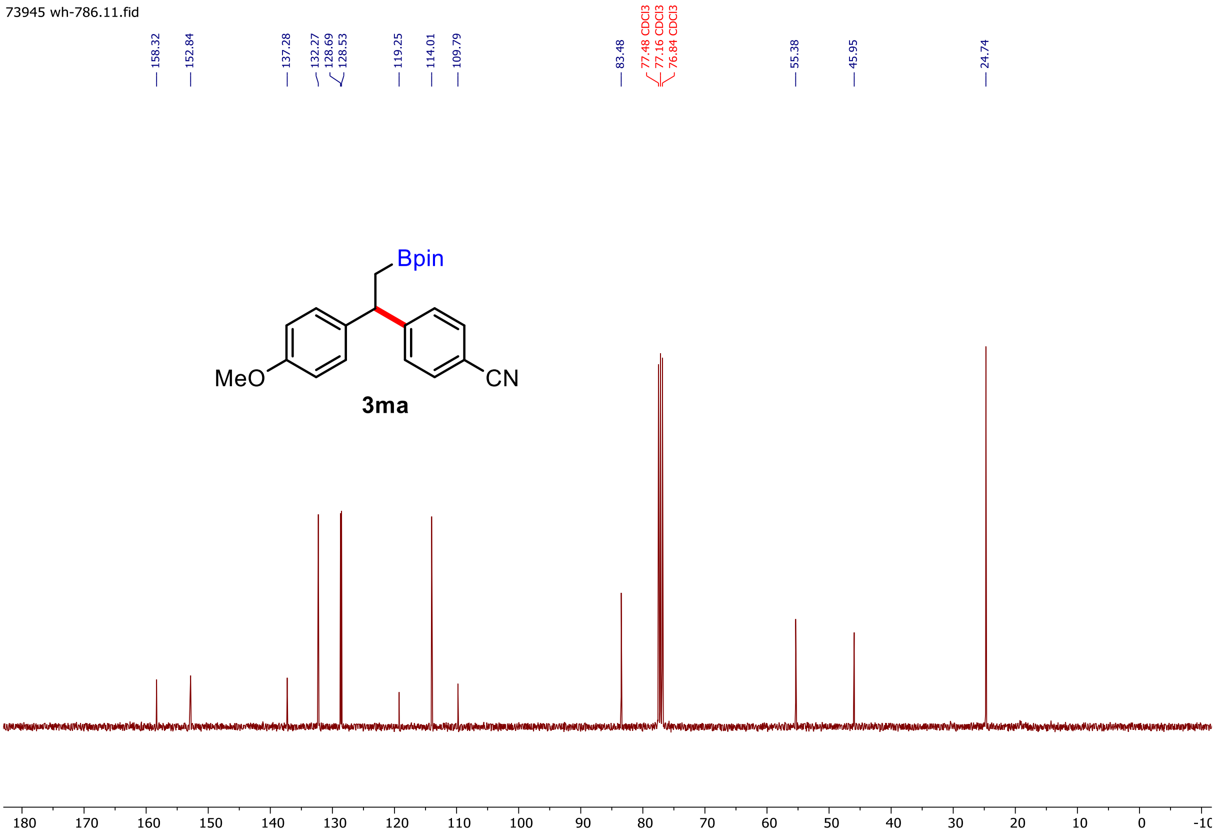

^{11}B NMR (128 MHz, $CDCl_3$) of **3ma**

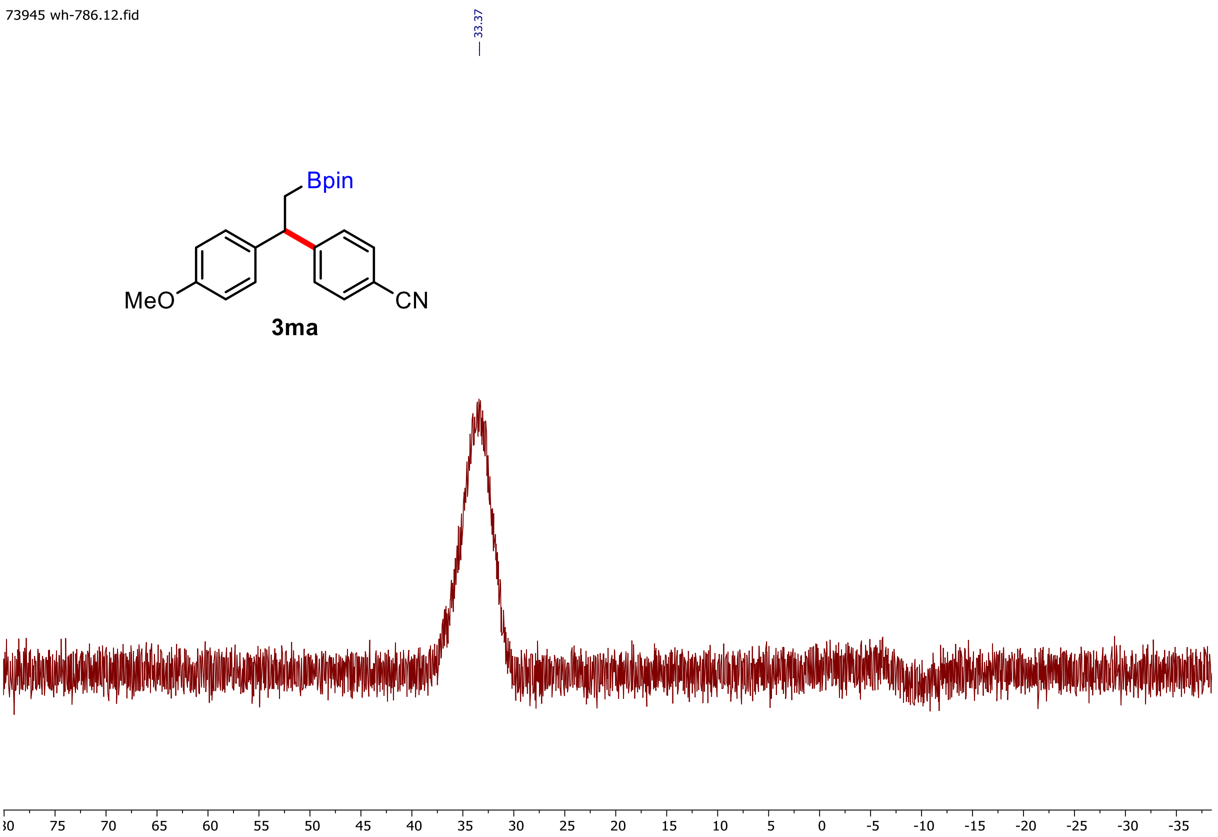

^{1}H NMR (400 MHz, $CDCl_3$) of **3na** (*see procedure*)

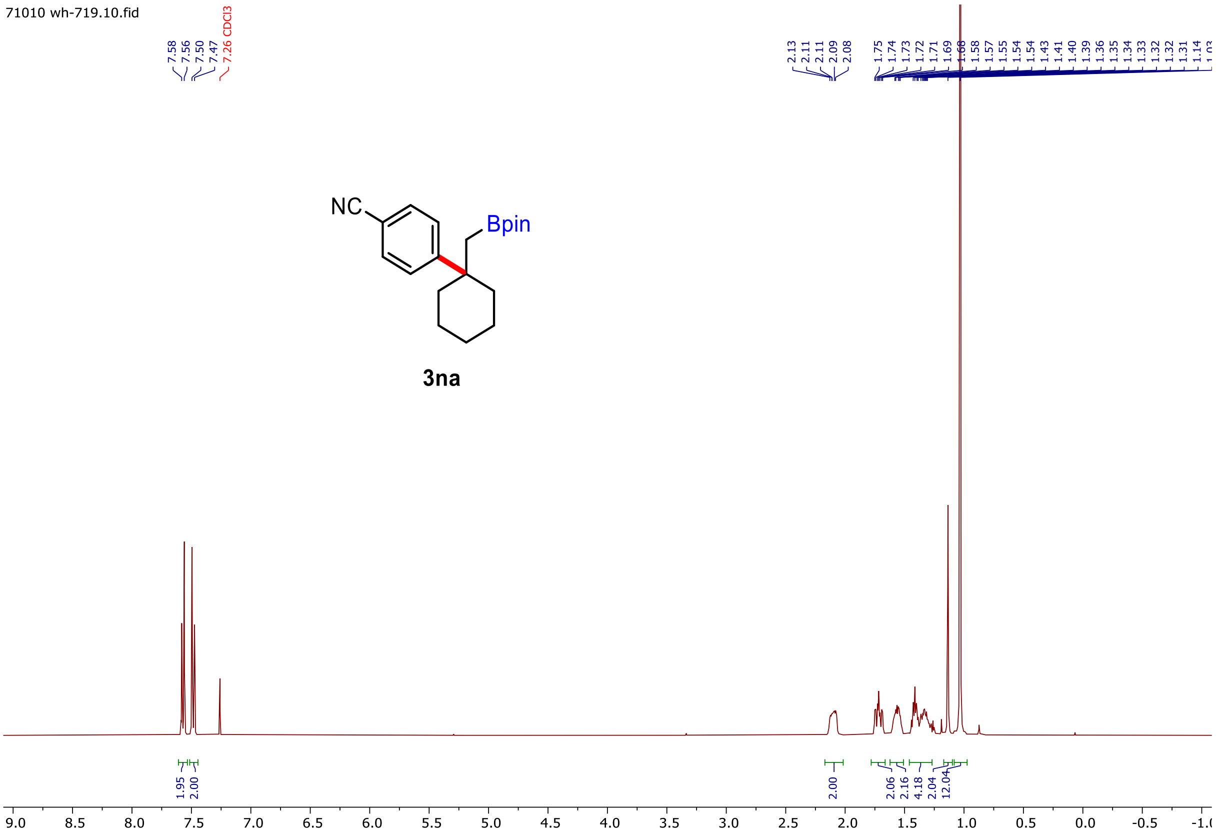

^{13}C NMR (101 MHz, $CDCl_3$) of **3na**

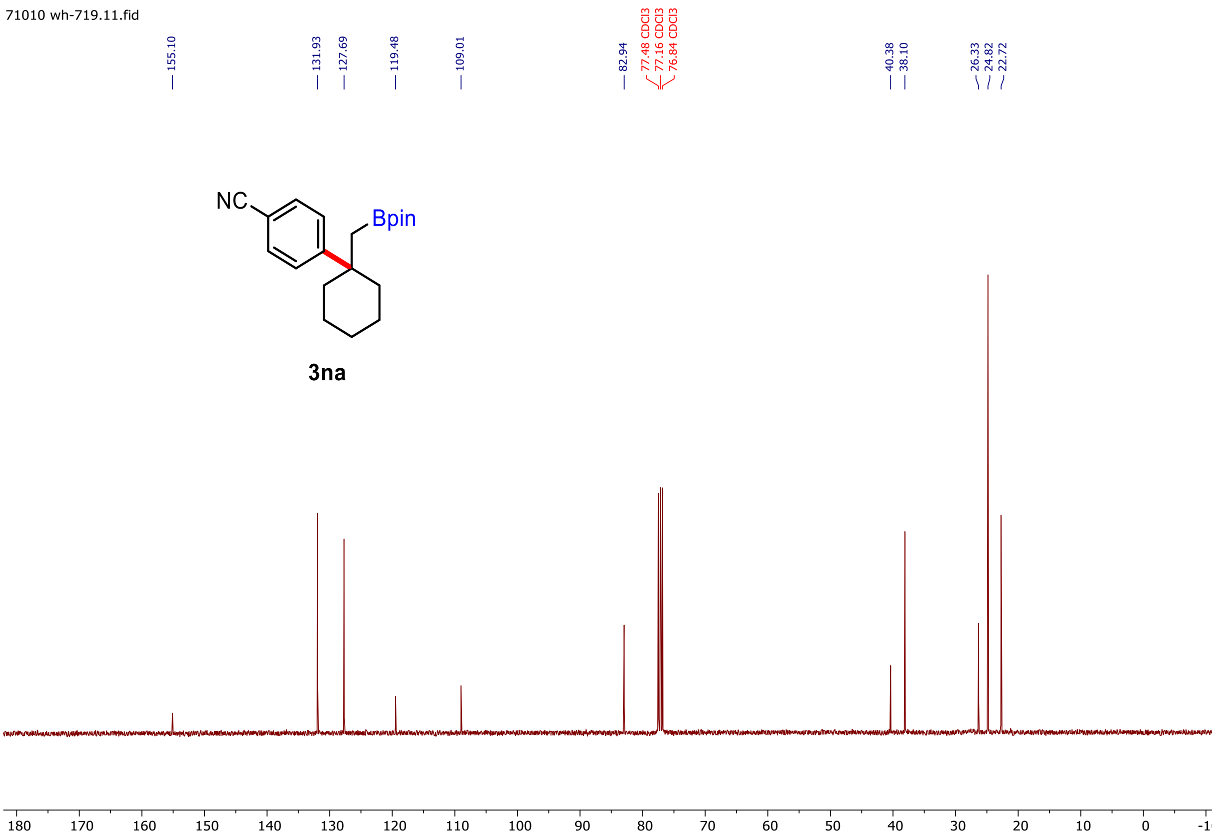

^{11}B NMR (128 MHz, $CDCl_3$) of **3na**

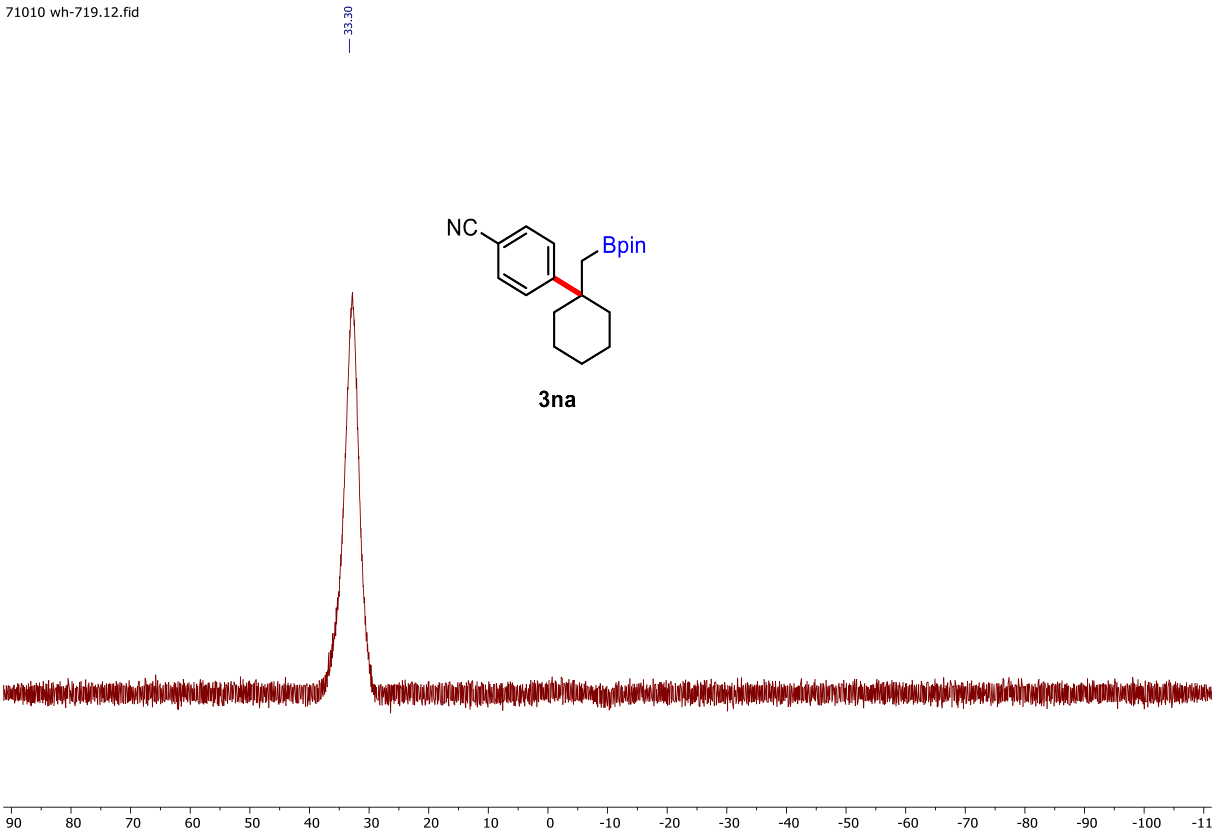

UOB Confidential & Sensitive

^{1}H NMR (400 MHz, $CDCl_3$) of **3oa** (*see procedure*)

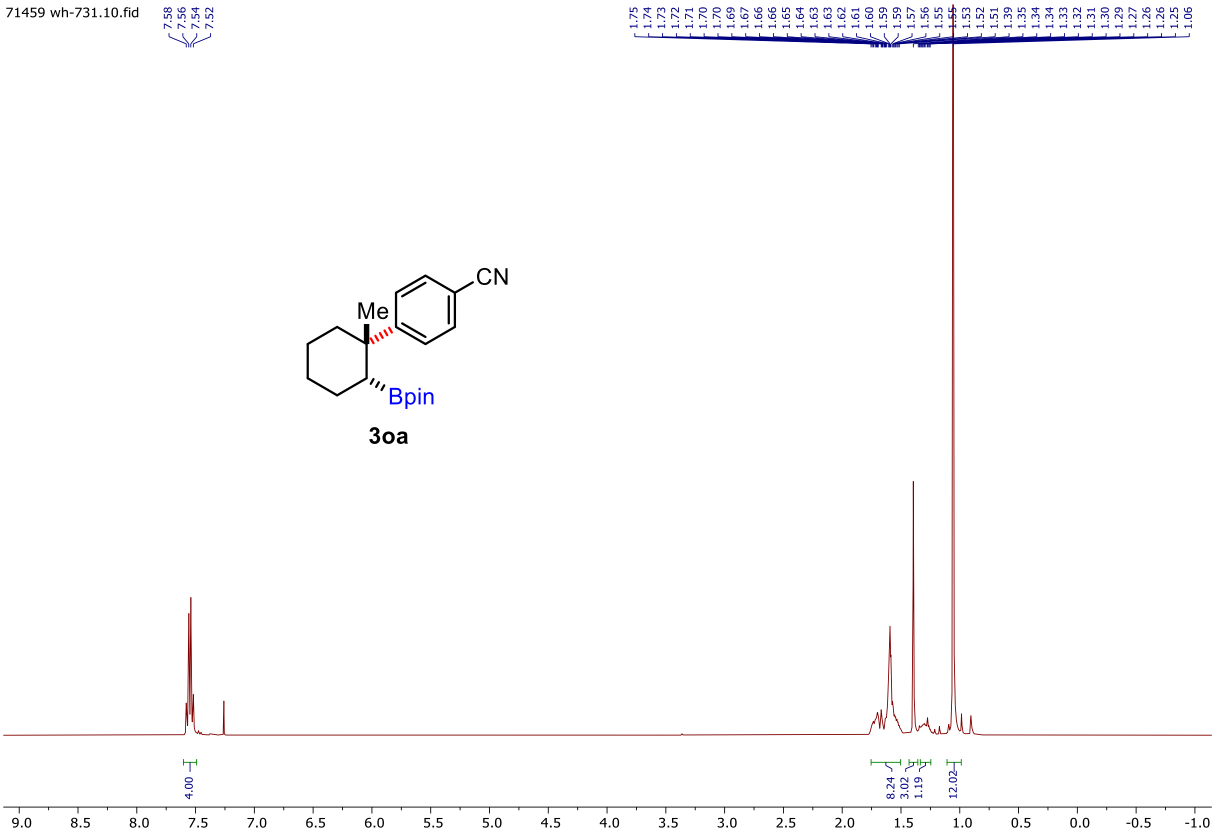

^{13}C NMR (101 MHz, $CDCl_3$) of **3oa**

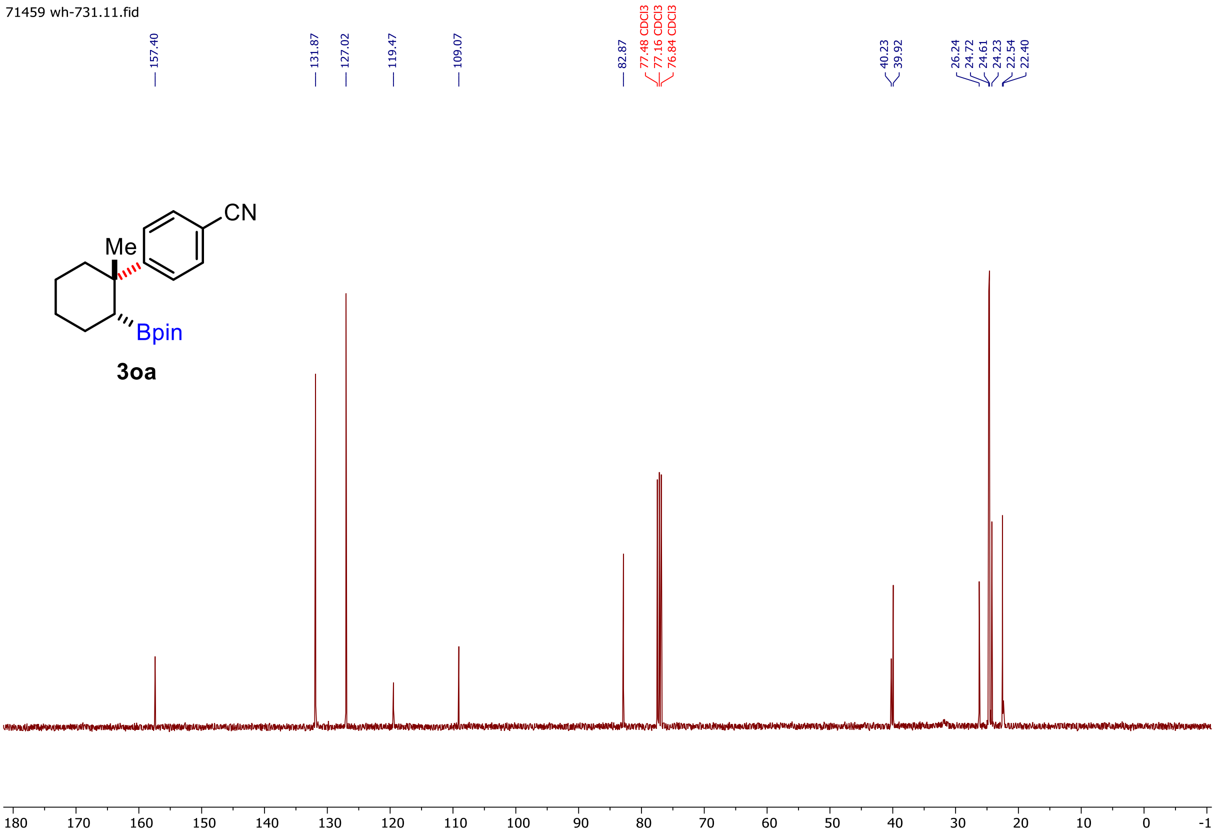

^{11}B NMR (128 MHz, $CDCl_3$) of **3oa**

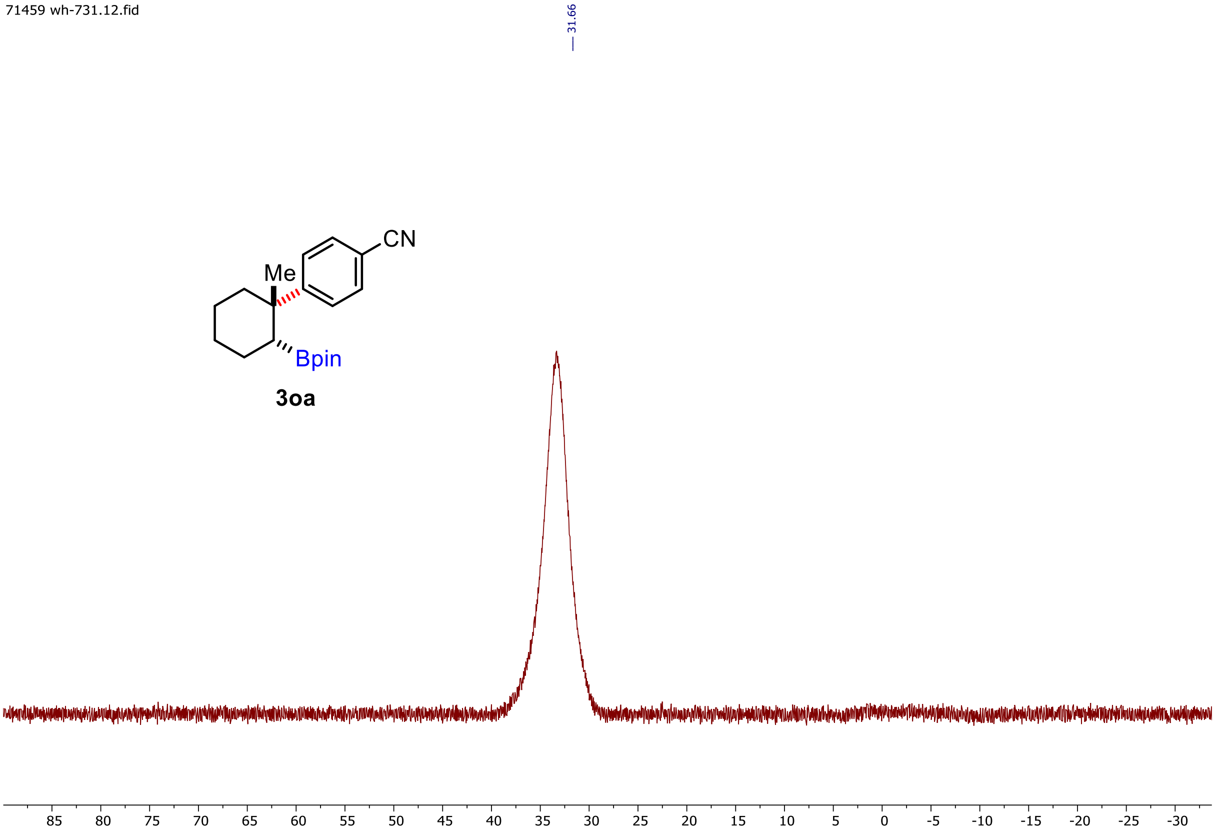

^{1}H NMR (400 MHz, $CDCl_3$) of **3pa** (*see procedure*)

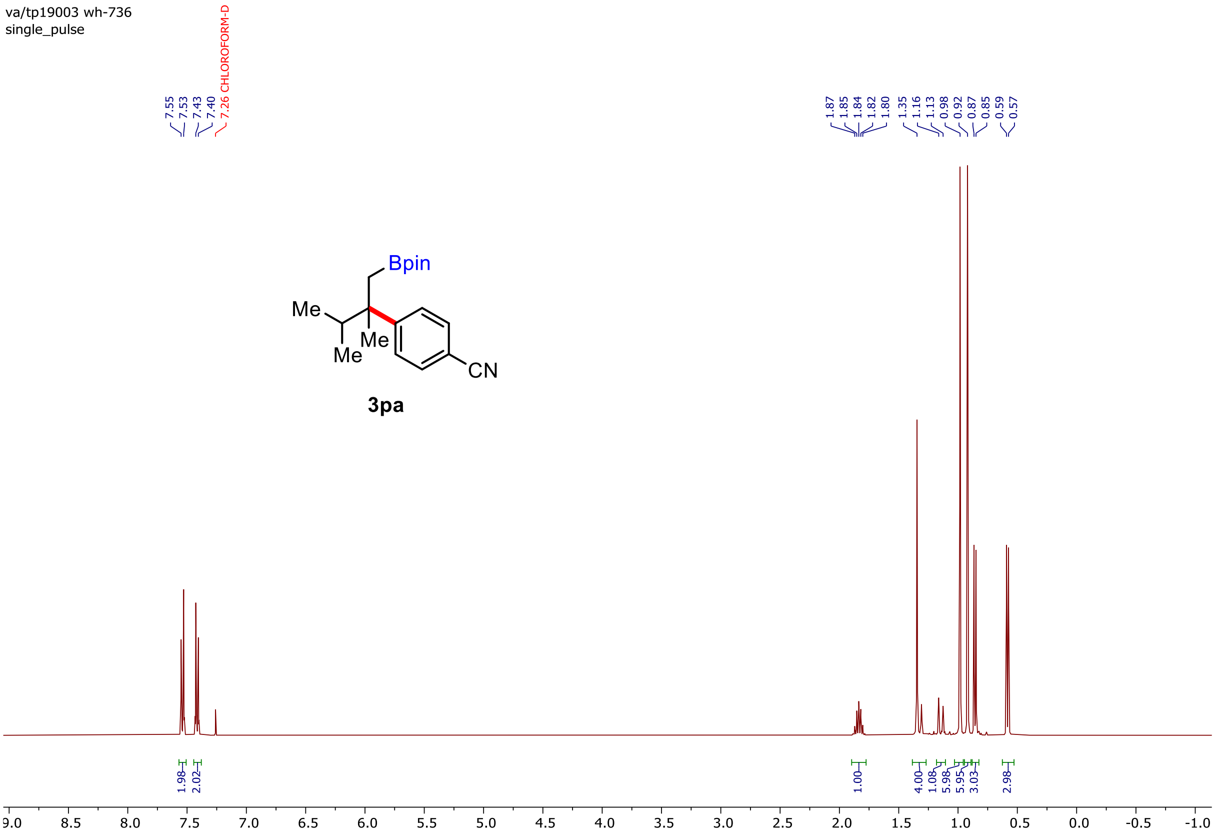

^{13}C NMR (101 MHz, $CDCl_3$) of **3pa**

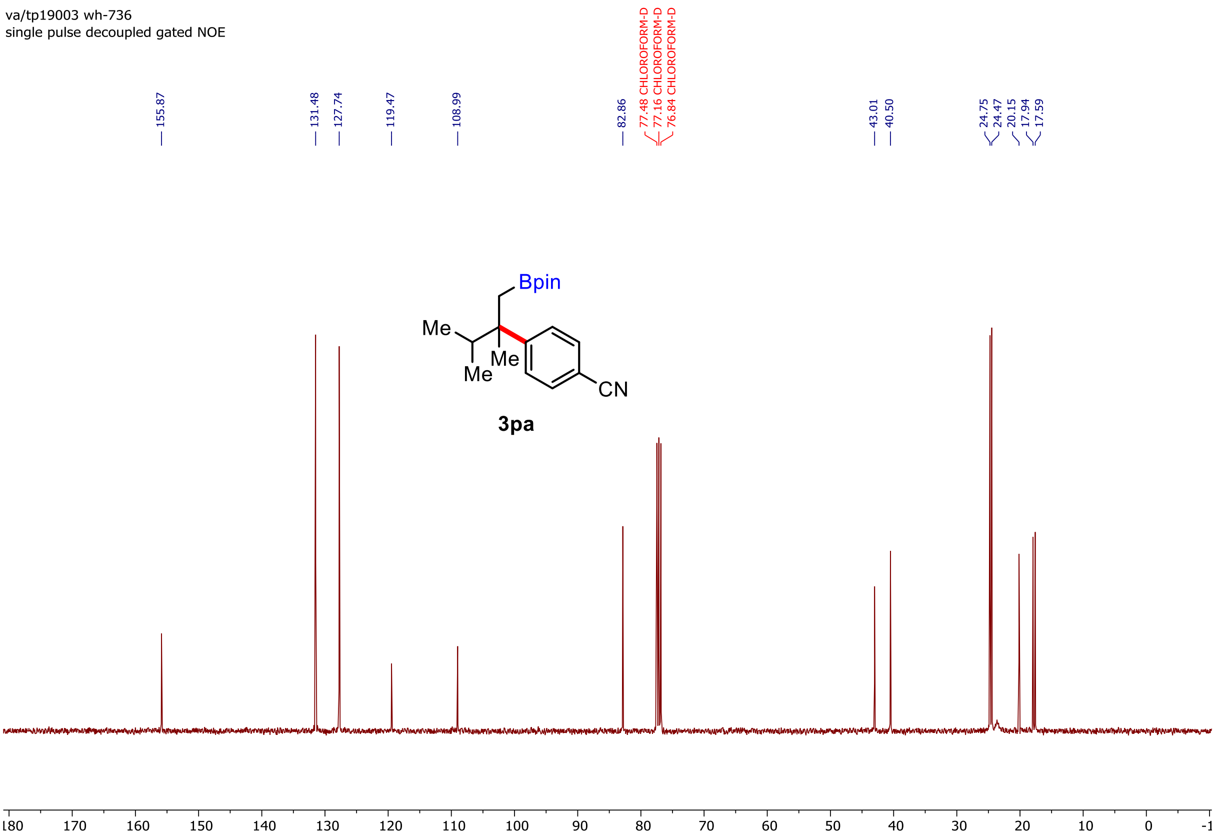

^{11}B NMR (128 MHz, $CDCl_3$) of **3pa**

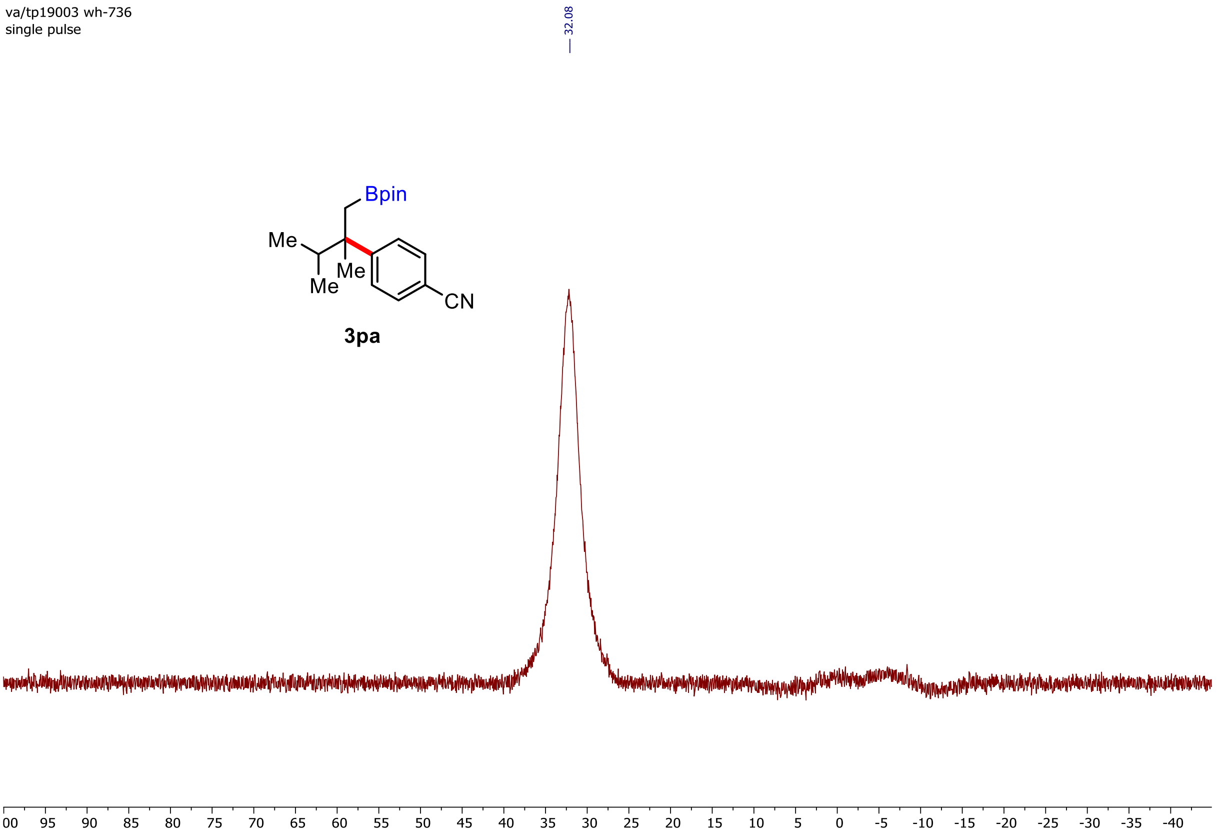

UOB Confidential & Sensitive

^{1}H NMR (400 MHz, $CDCl_3$) of **(*trans*)-3qa** (*see procedure*)

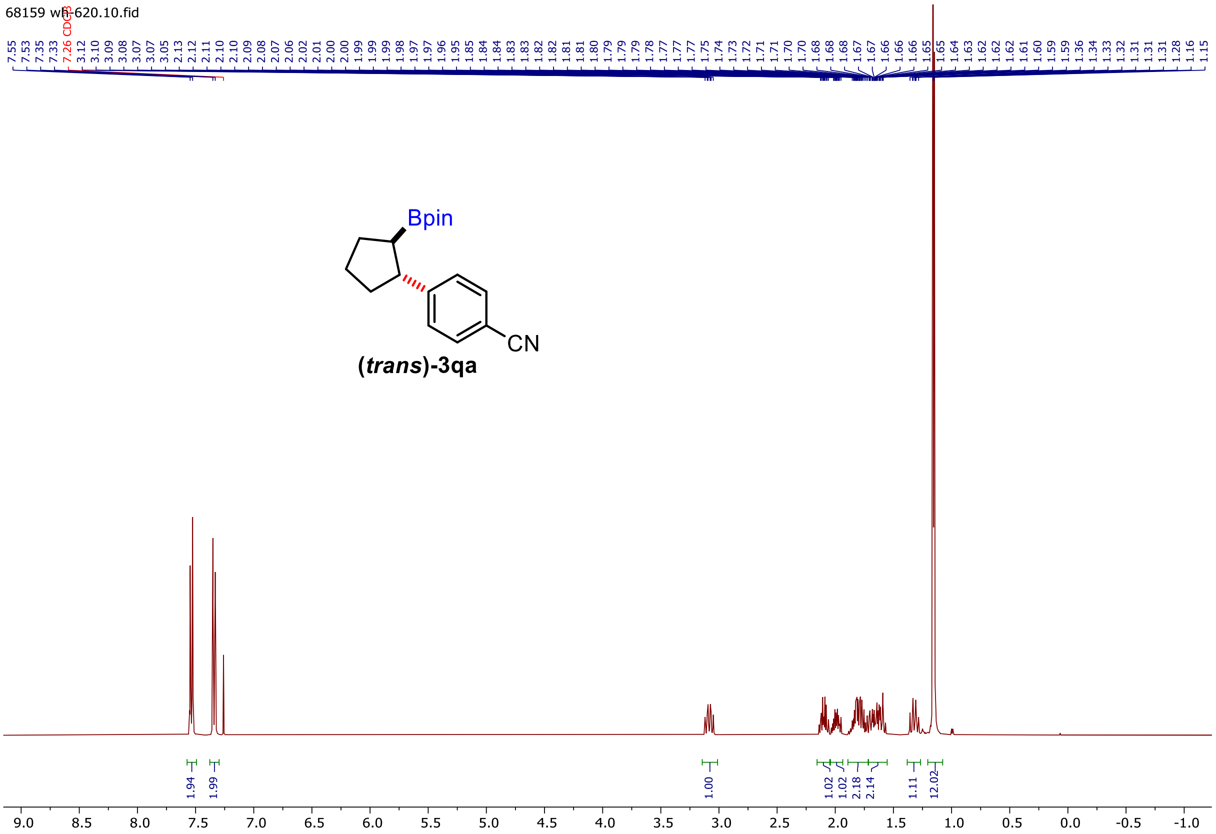

^{13}C NMR (101 MHz, $CDCl_3$) of **(*trans*)-3qa**

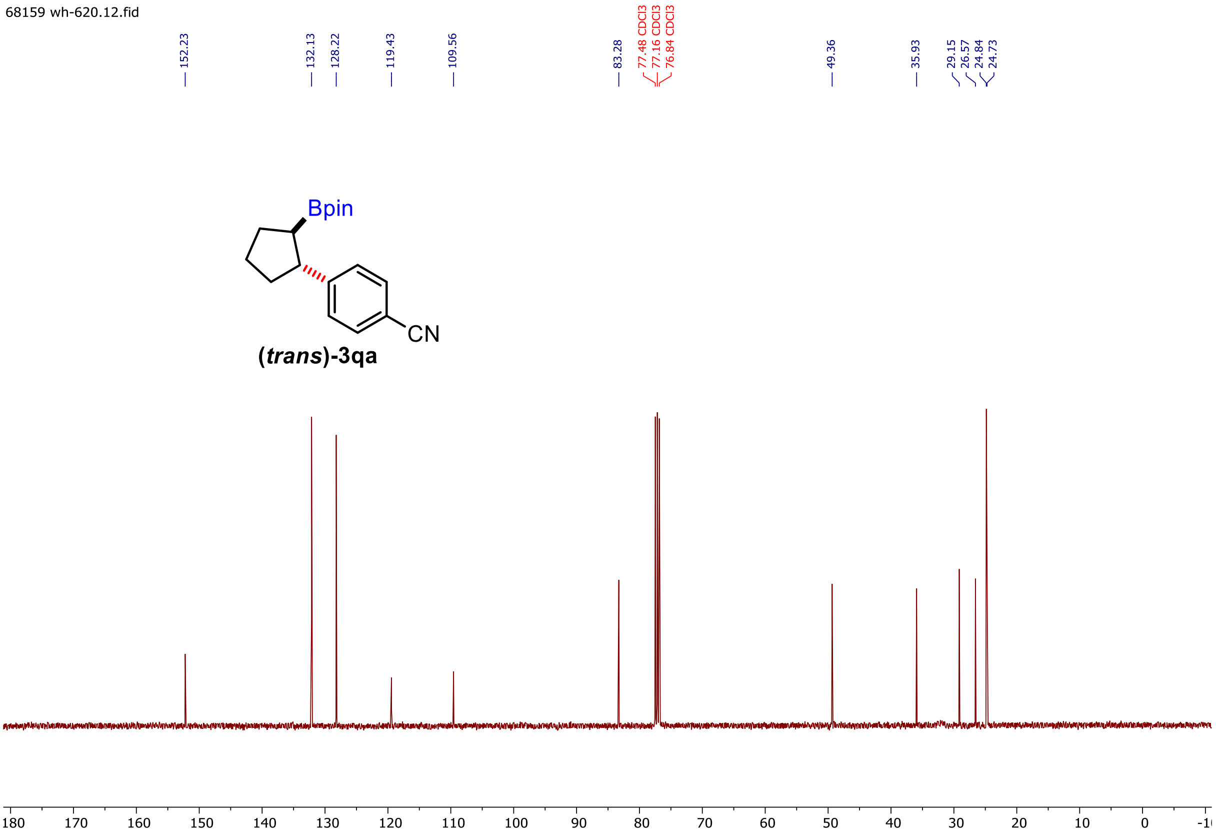

^{11}B NMR (128 MHz, CDCl$_3$) of **(*trans*)-3qa**

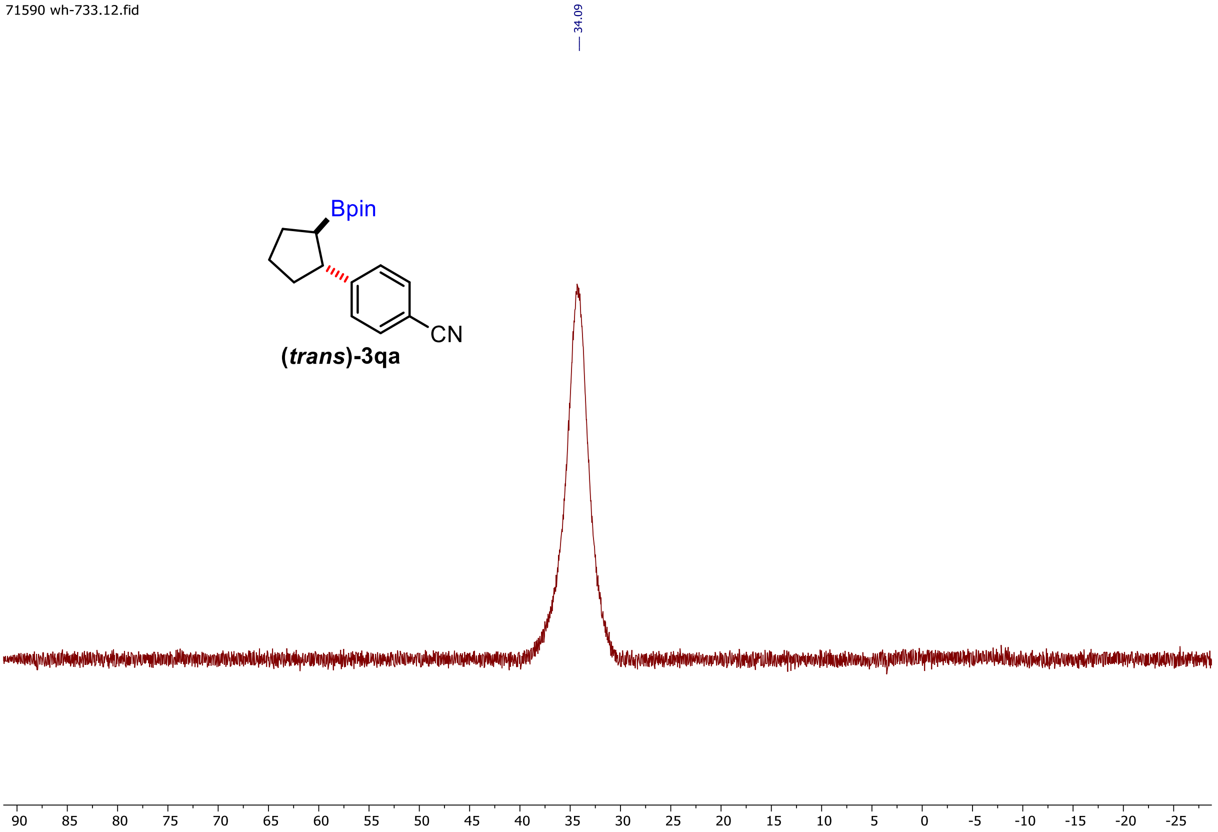

^{1}H NMR (400 MHz, CDCl$_3$) of **(*cis*)-3qa** (*see procedure*)

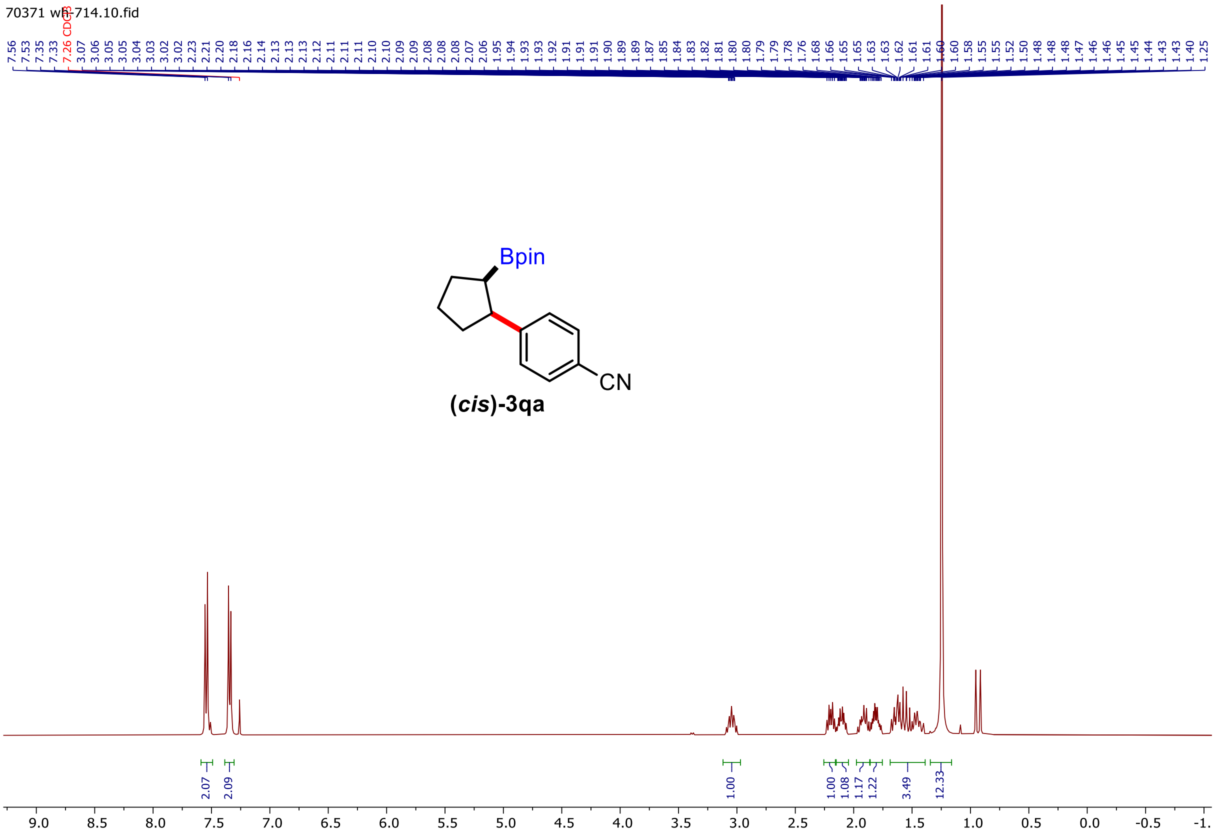

UOB Confidential & Sensitive

^{13}C NMR (101 MHz, $CDCl_3$) of **(*cis*)-3qa**

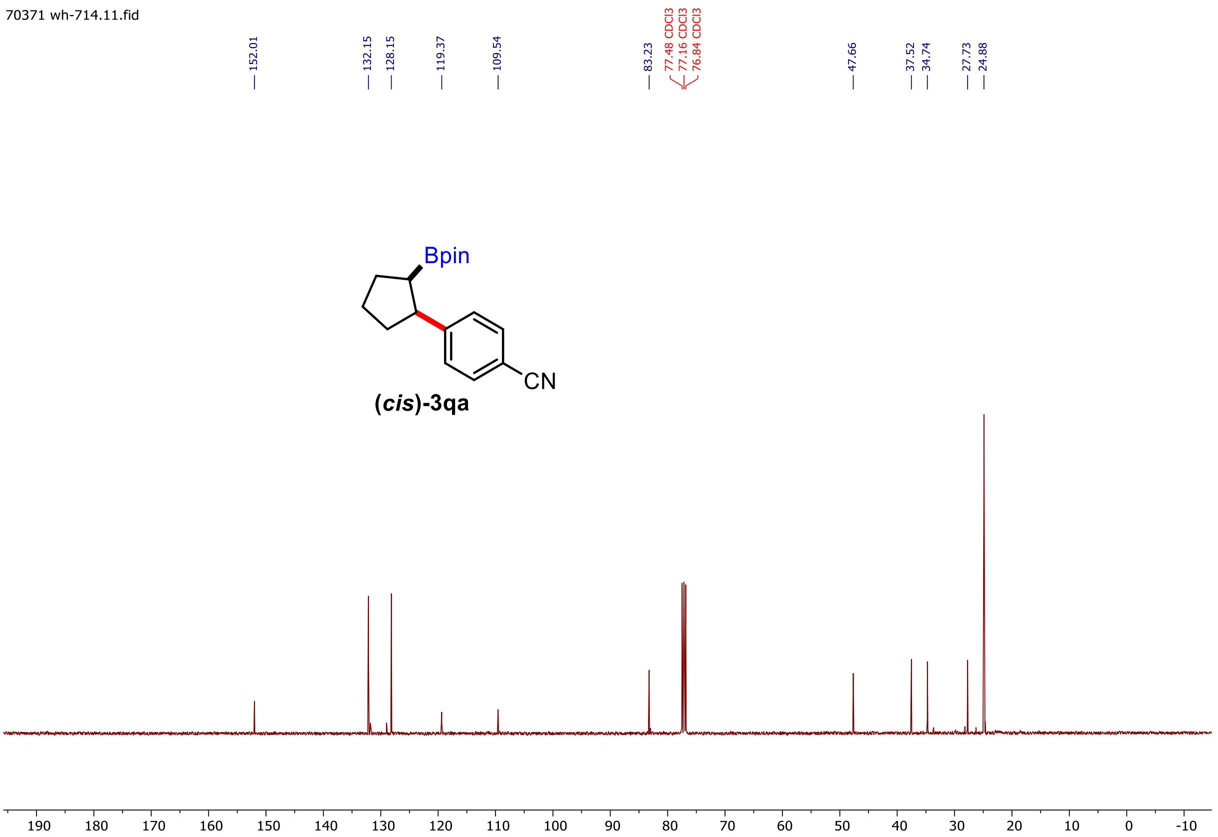

^{11}B NMR (128 MHz, $CDCl_3$) of **(*cis*)-3qa**

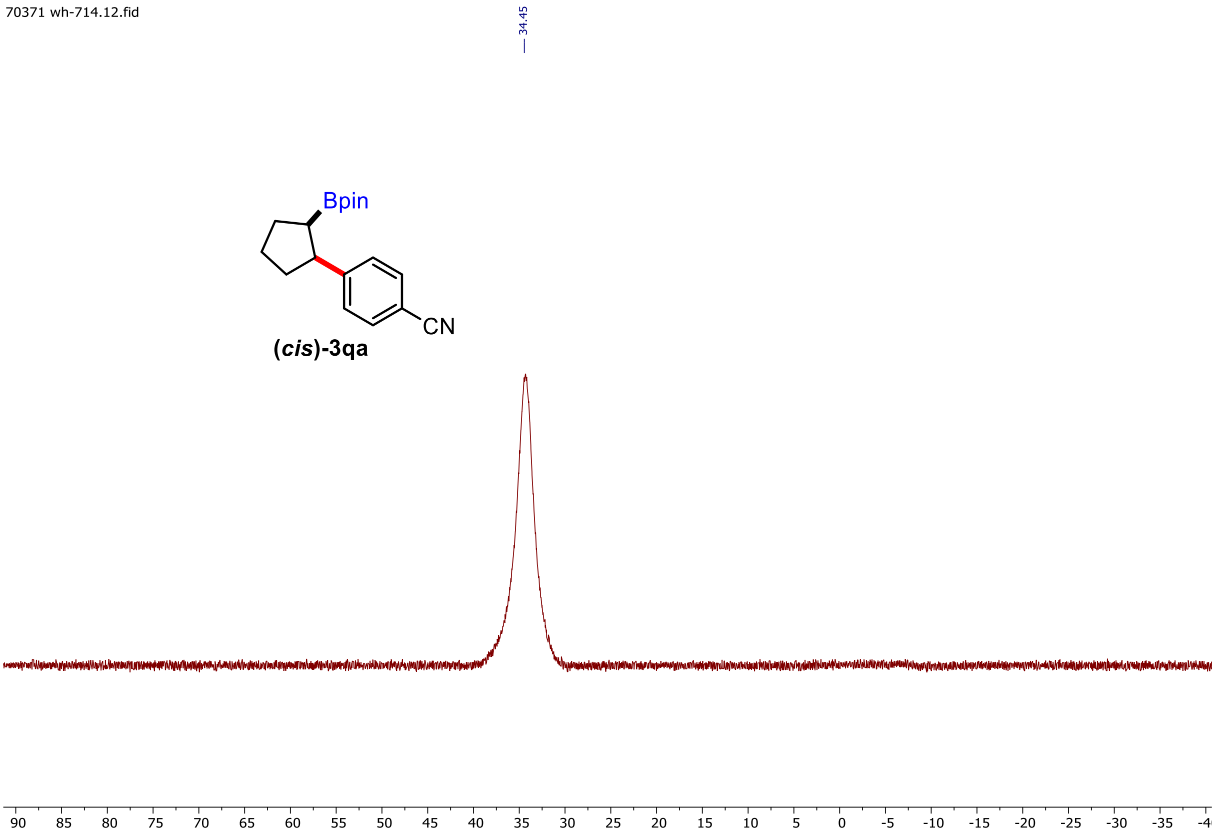

^{1}H NMR (400 MHz, $CDCl_3$) of **(*trans*)-3ra** (*see procedure*)

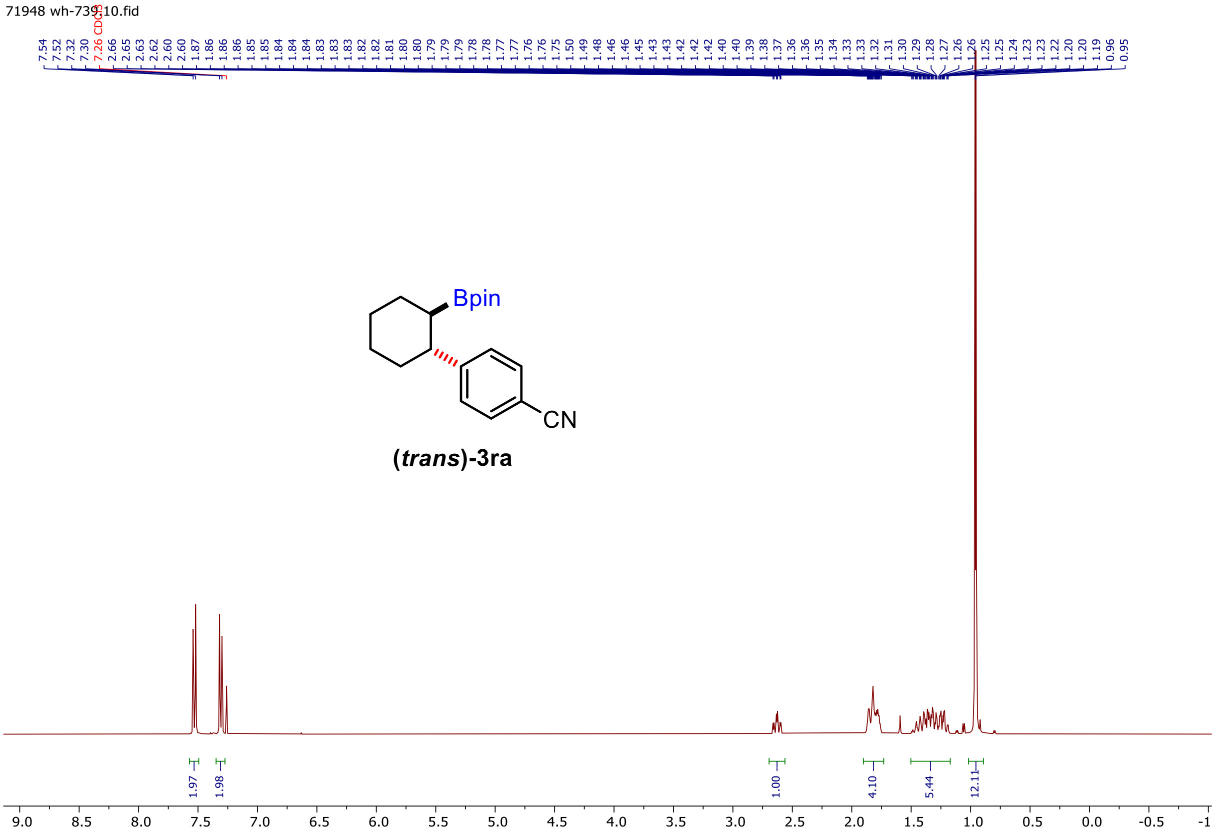

^{13}C NMR (101 MHz, $CDCl_3$) of **(*trans*)-3ra**

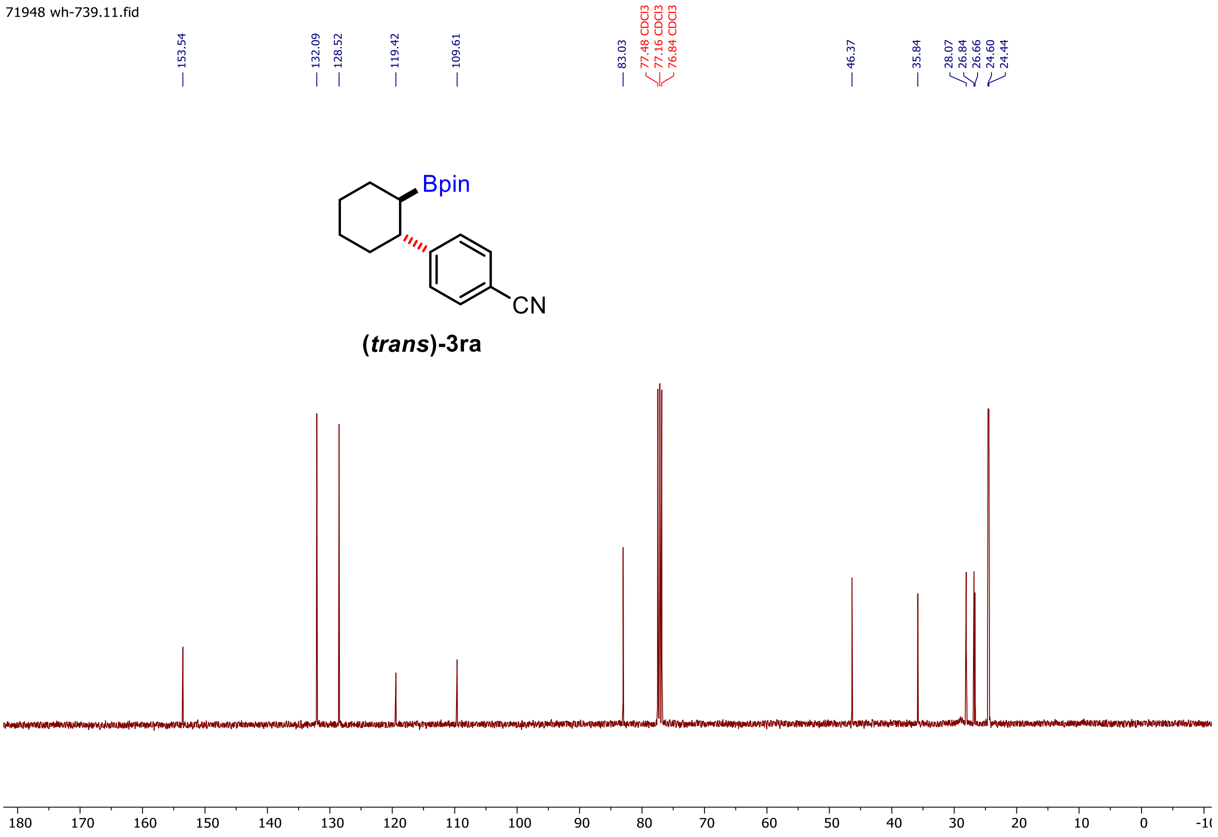

UOB Confidential & Sensitive

^{11}B NMR (128 MHz, $CDCl_3$) of **(*trans*)-3ra**

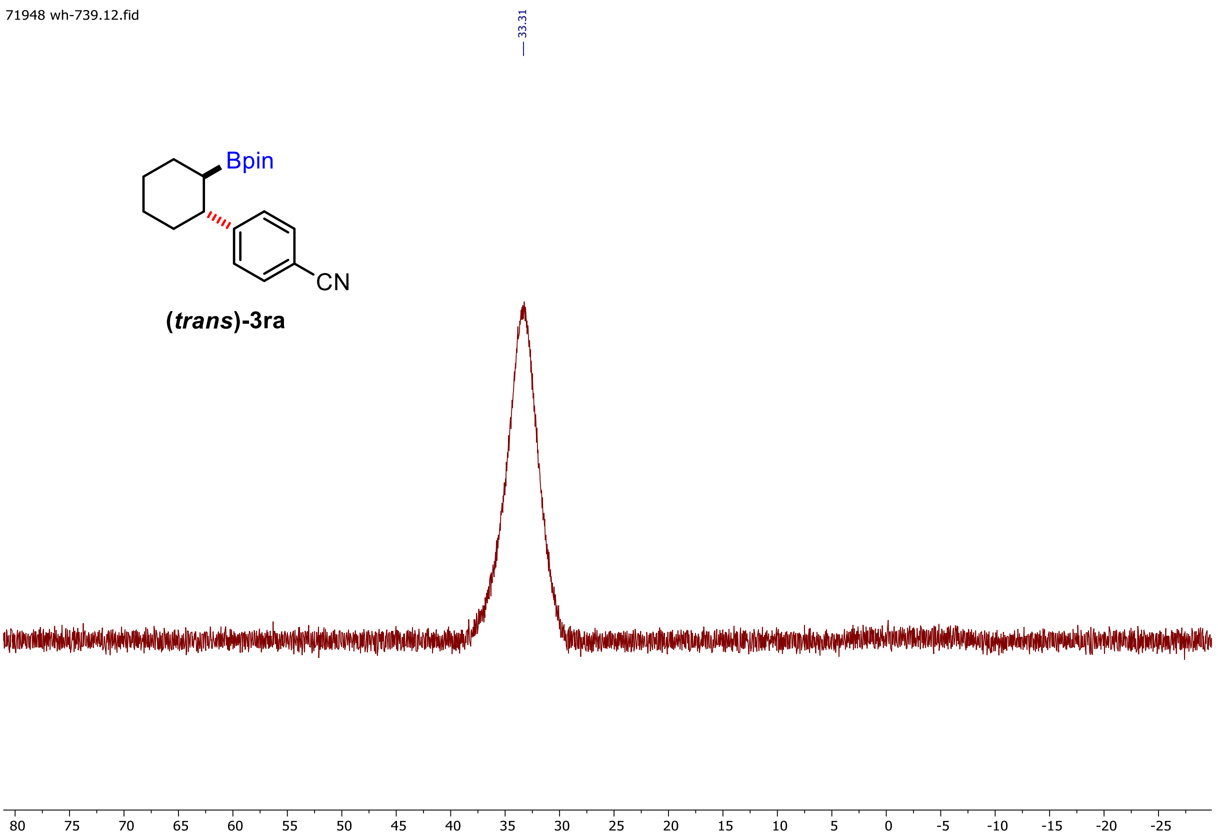

^{1}H NMR (400 MHz, $CDCl_3$) of **(*cis*)-3ra** (*see procedure*)

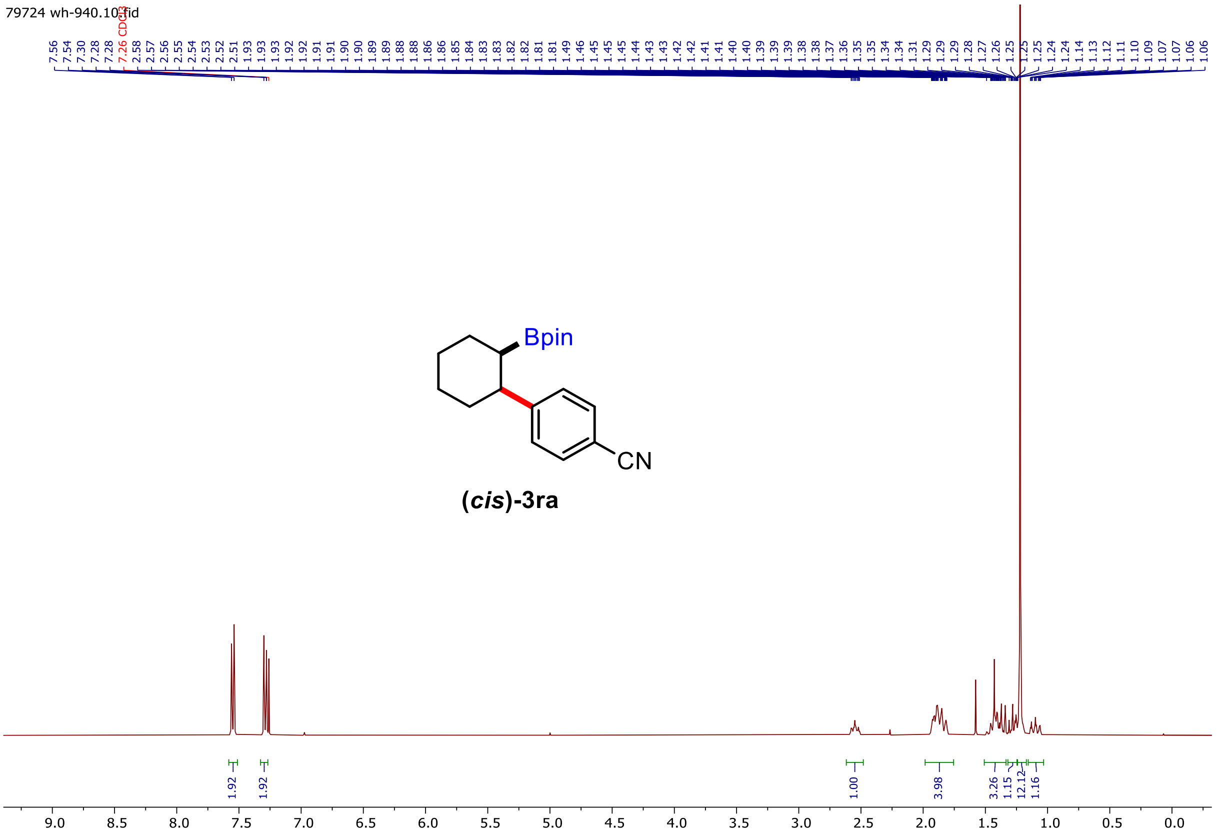

UOB Confidential & Sensitive

^{13}C NMR (101 MHz, CDCl$_3$) of **(*cis*)-3ra**

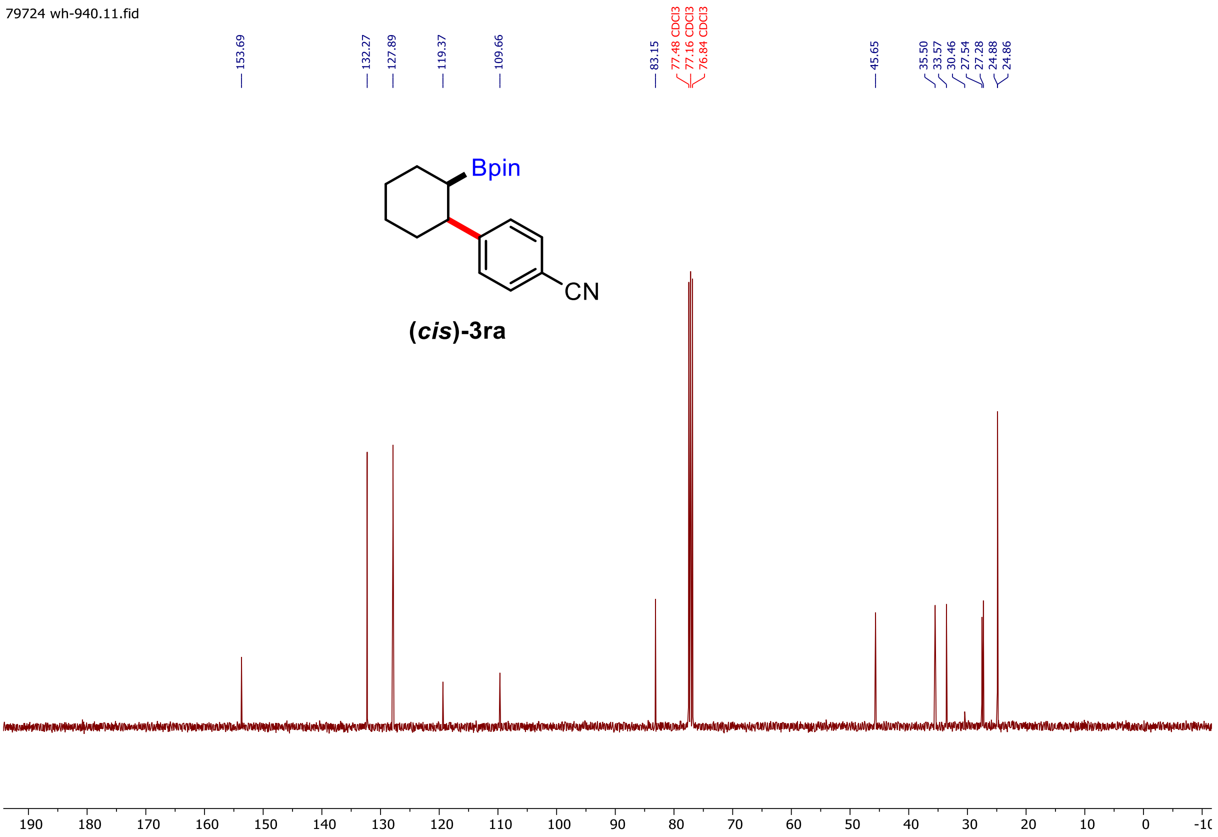

^{11}B NMR (128 MHz, CDCl$_3$) of **(*cis*)-3qa**

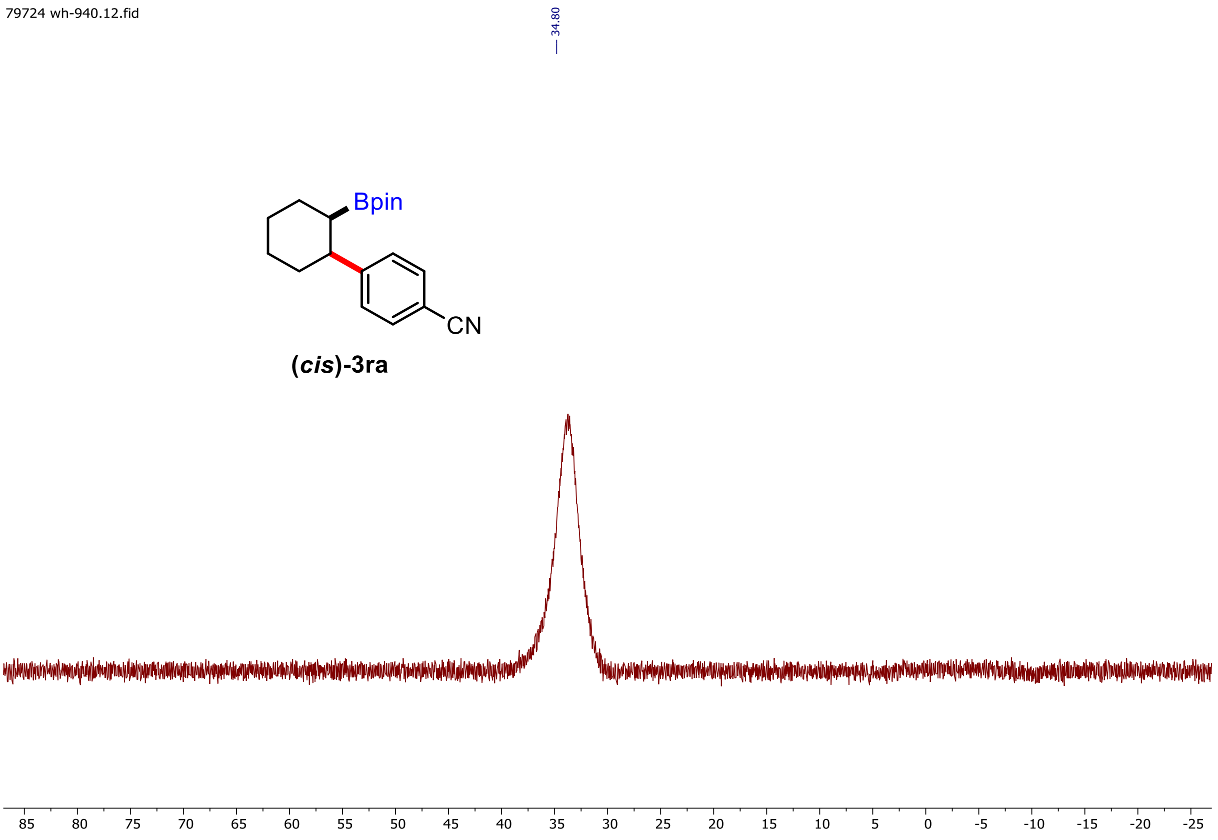

^{1}H NMR (400 MHz, $CDCl_3$) of **3sa** (*see procedure*)

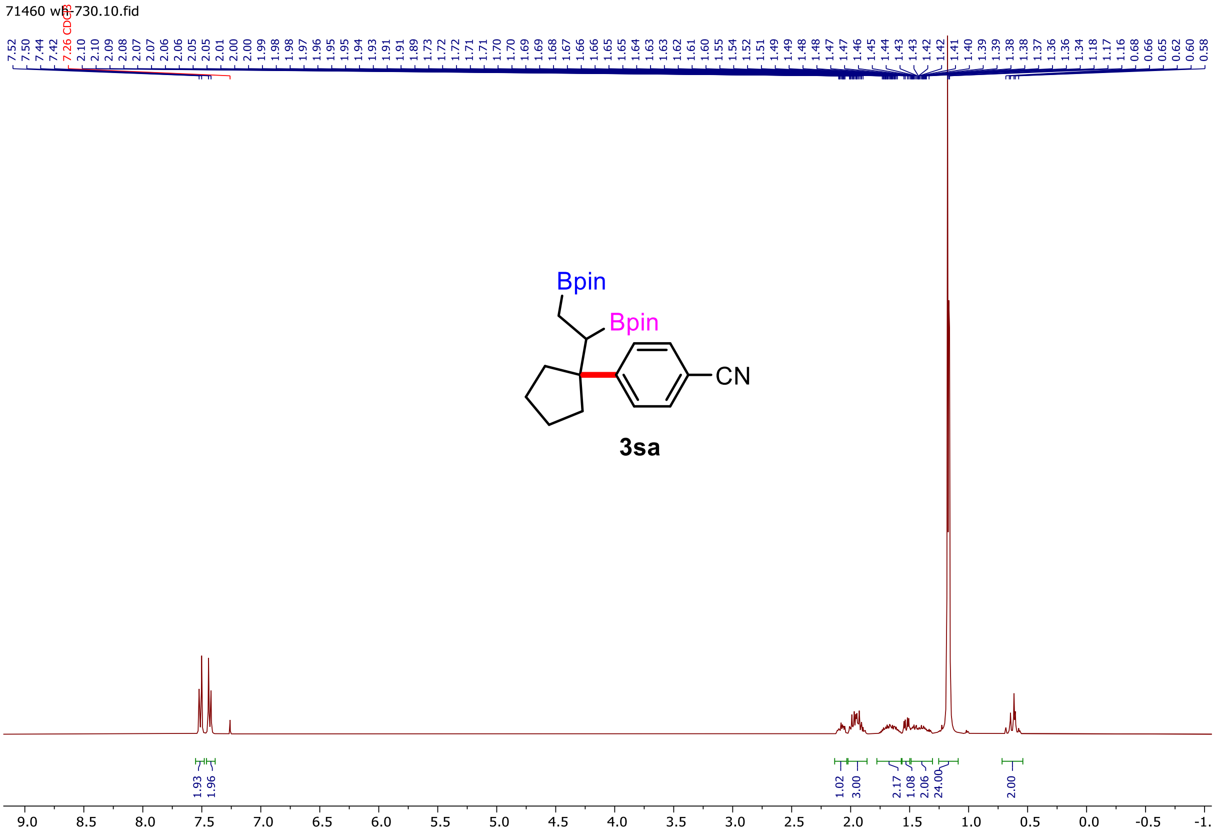

^{13}C NMR (101 MHz, $CDCl_3$) of **3sa**

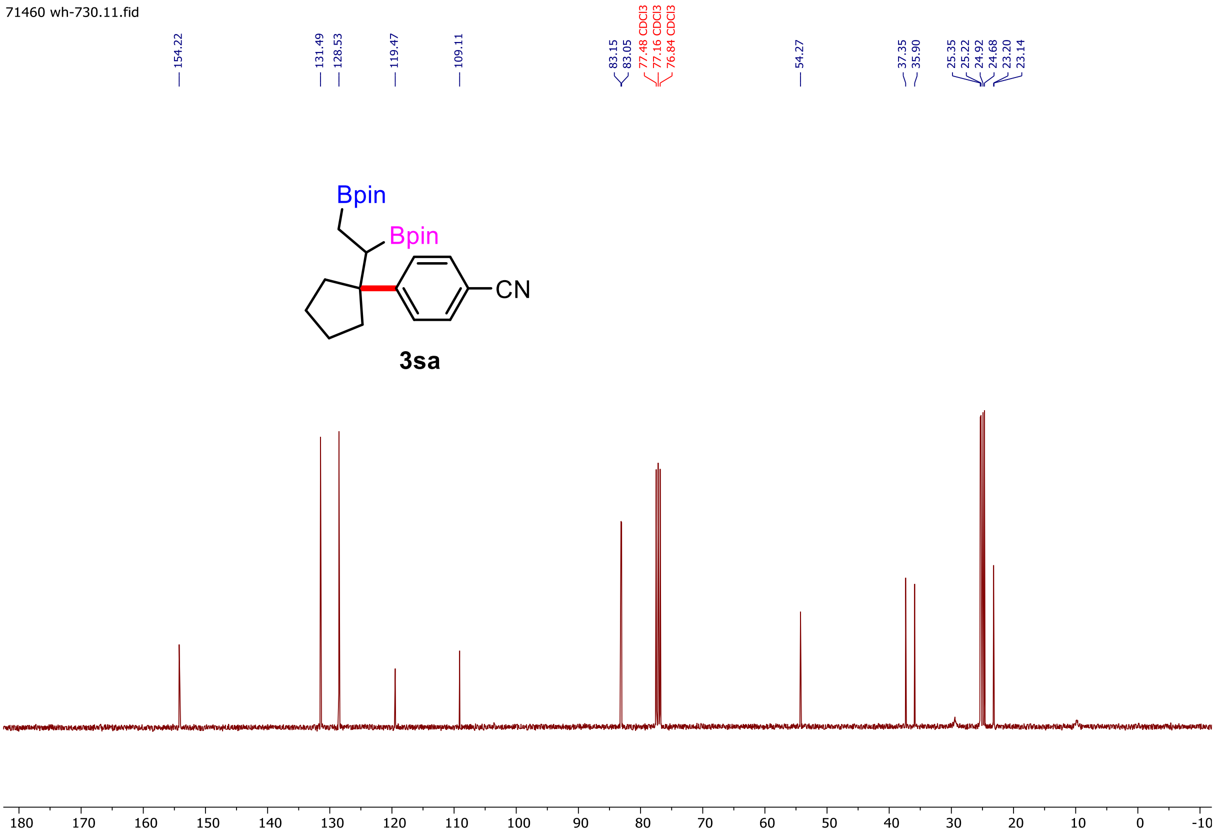

^{11}B NMR (128 MHz, $CDCl_3$) of **3sa**

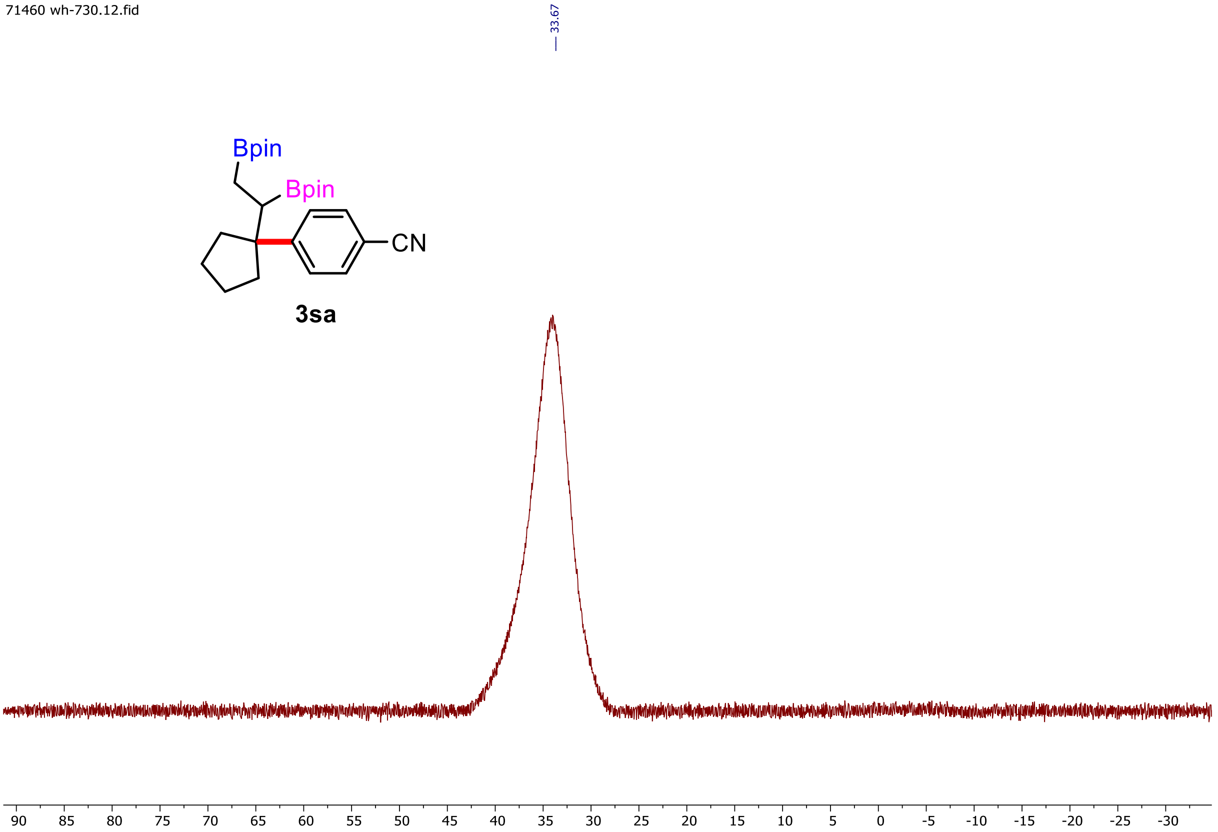

^{1}H NMR (400 MHz, $CDCl_3$) of **3ta** (*see procedure*)

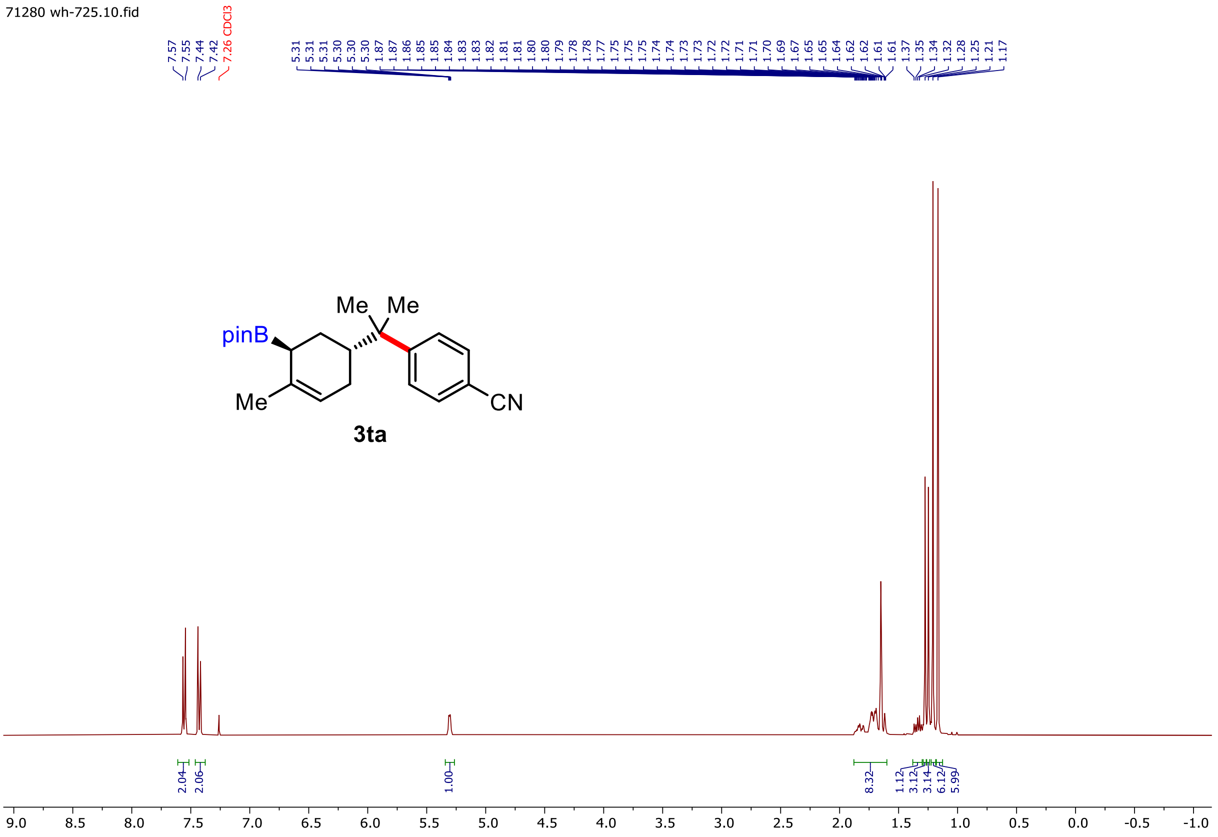

UOB Confidential & Sensitive

^{13}C NMR (101 MHz, $CDCl_3$) of **3ta**

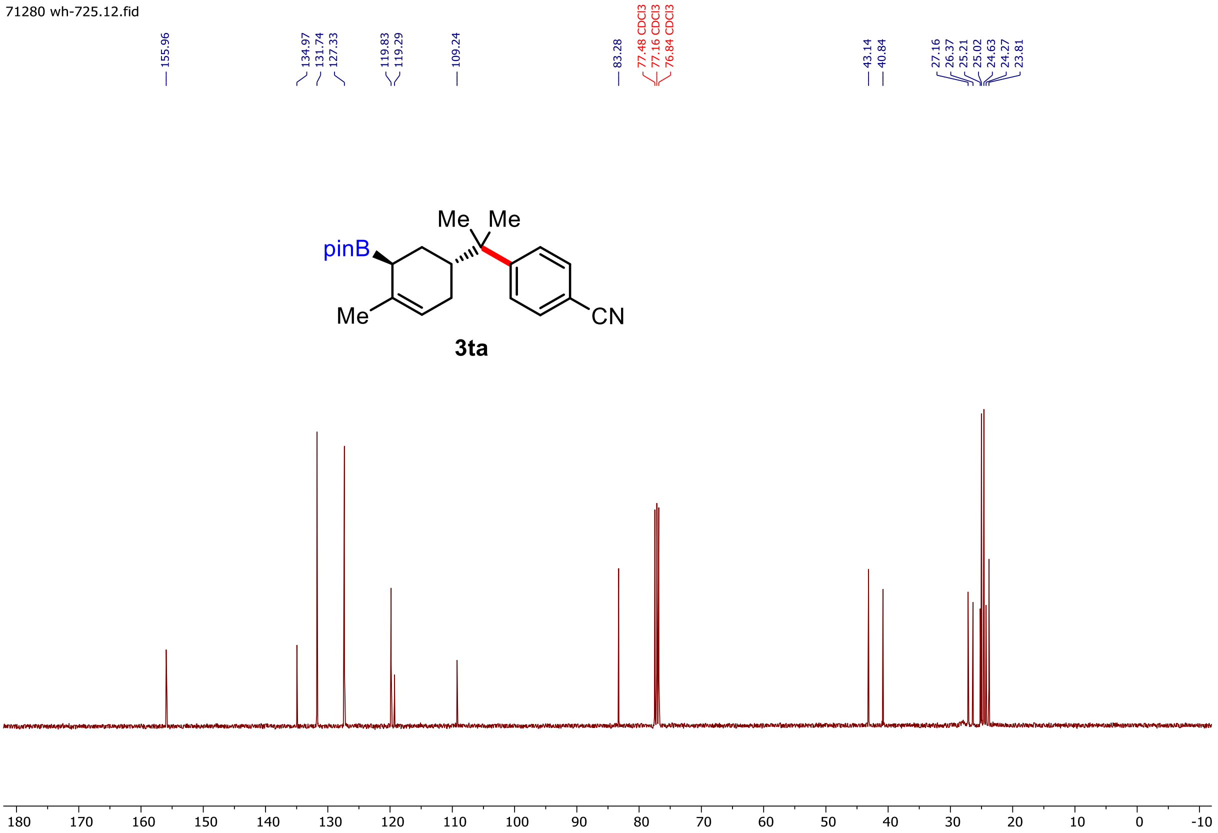

^{11}B NMR (128 MHz, $CDCl_3$) of **3sa**

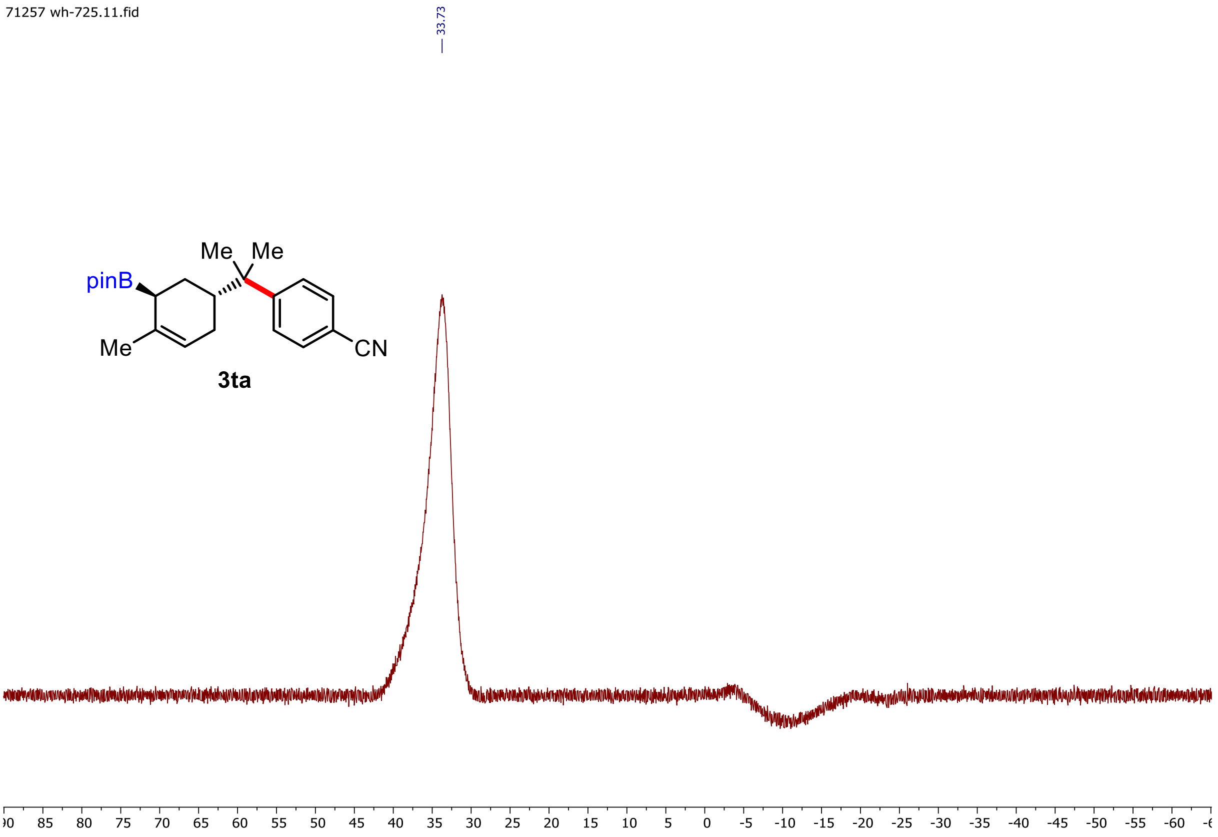

^{1}H NMR (400 MHz, $CDCl_3$) of **3ua** (*see procedure*)

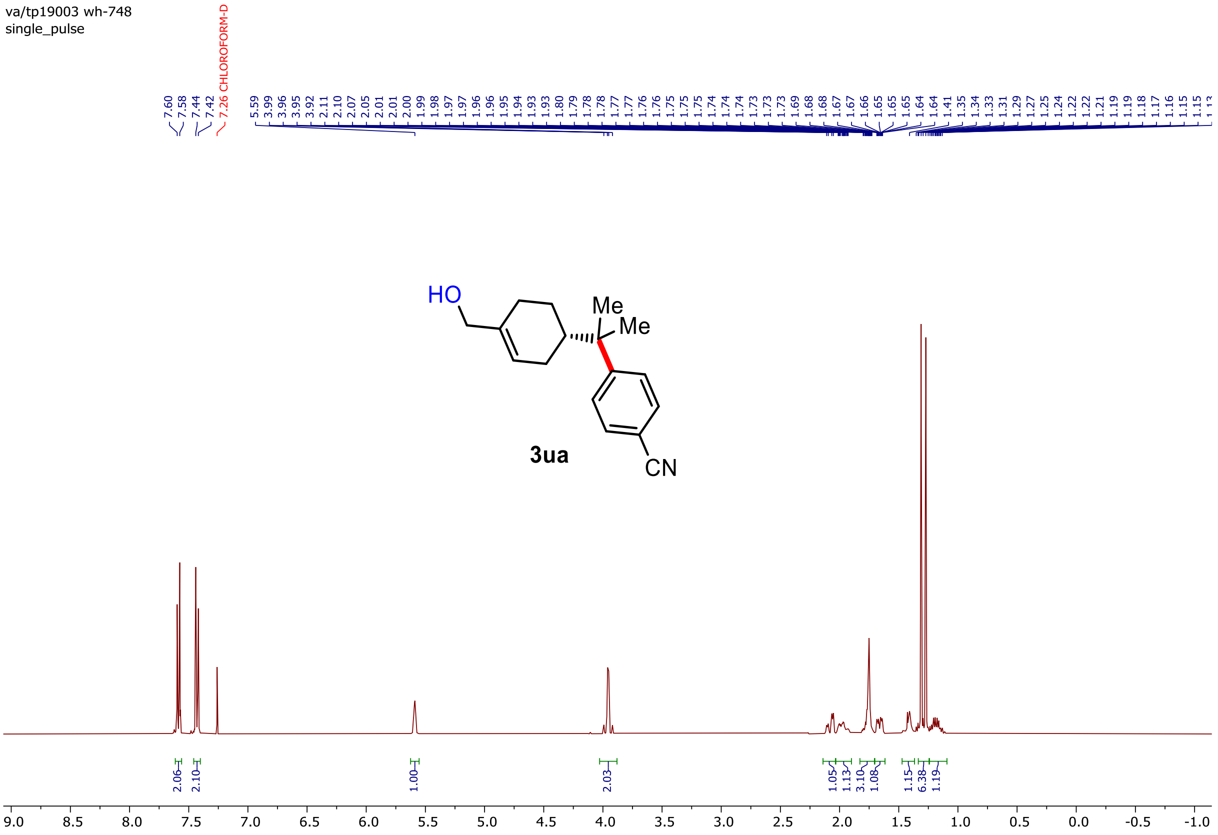

^{13}C NMR (101 MHz, $CDCl_3$) of **3ua**

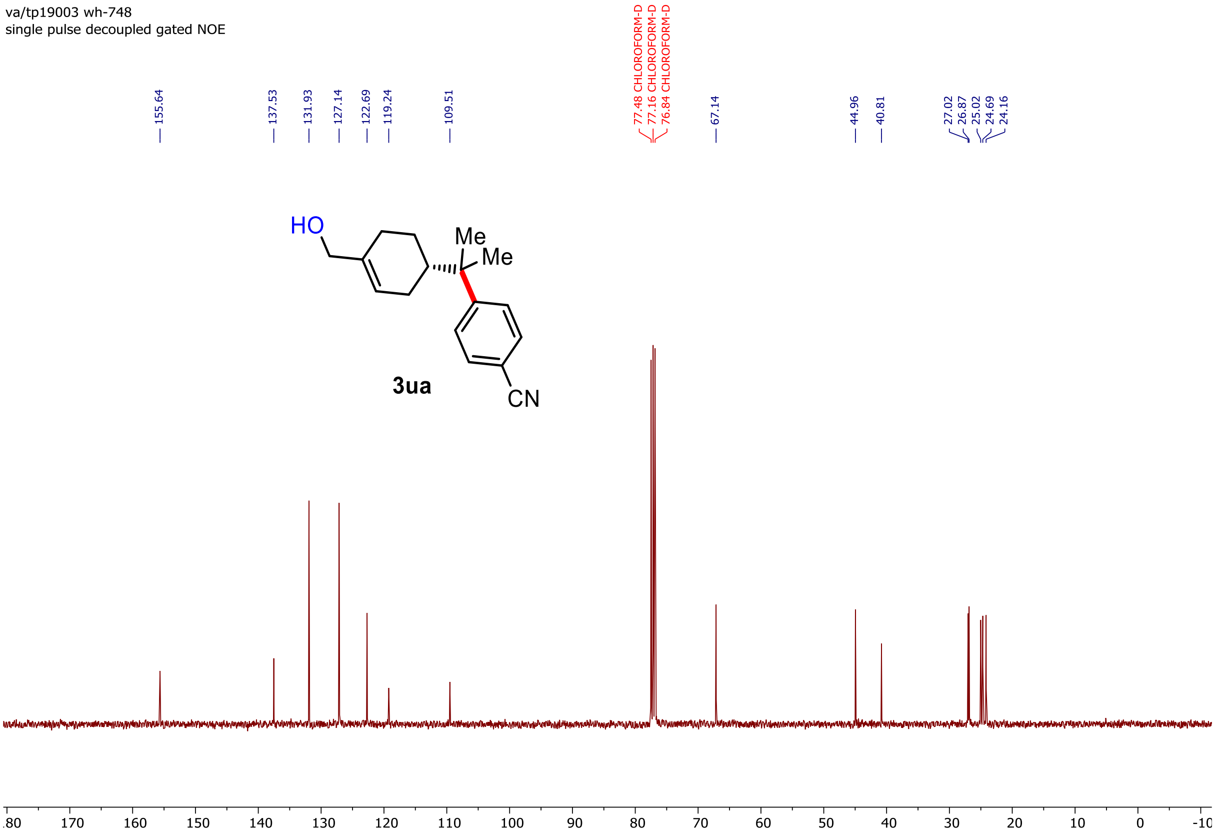

^{1}H NMR (400 MHz, $CDCl_3$) of **3va** (see procedure)

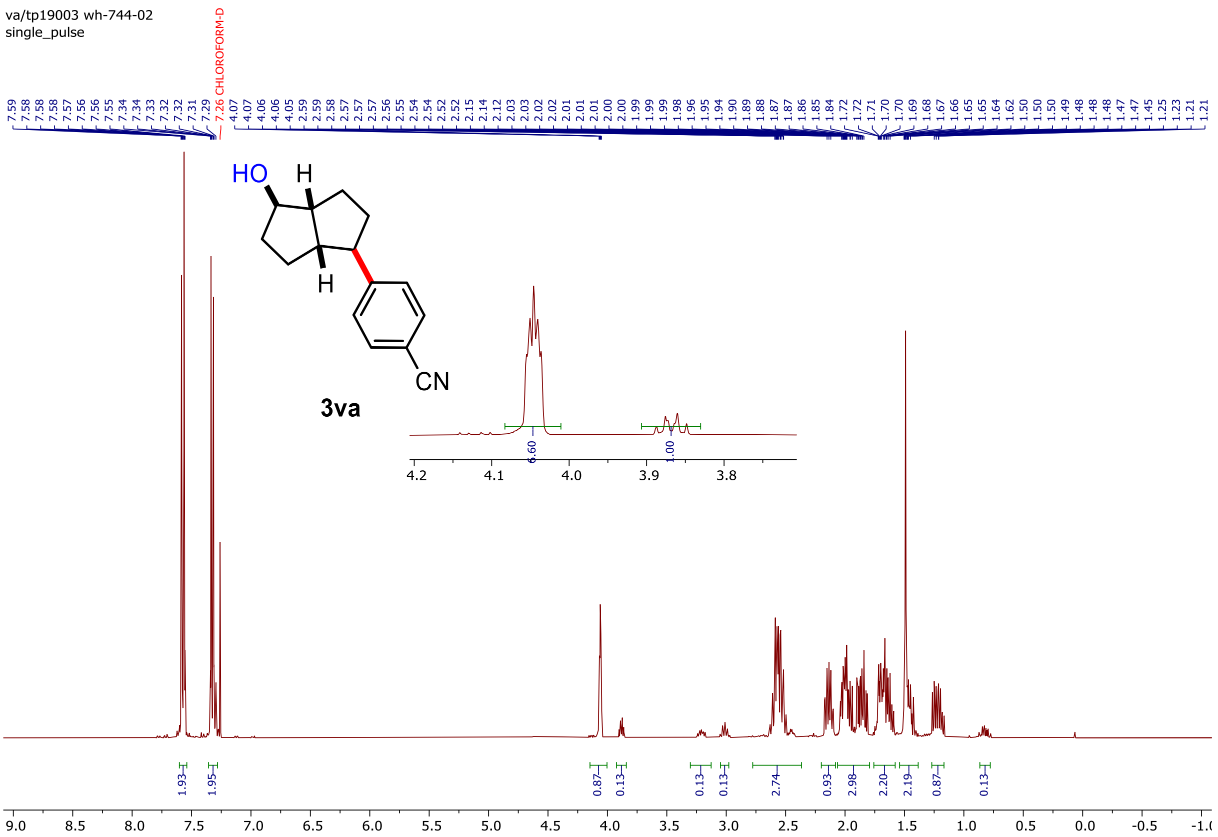

NOESY (400 MHz, $CDCl_3$) of **3ua**

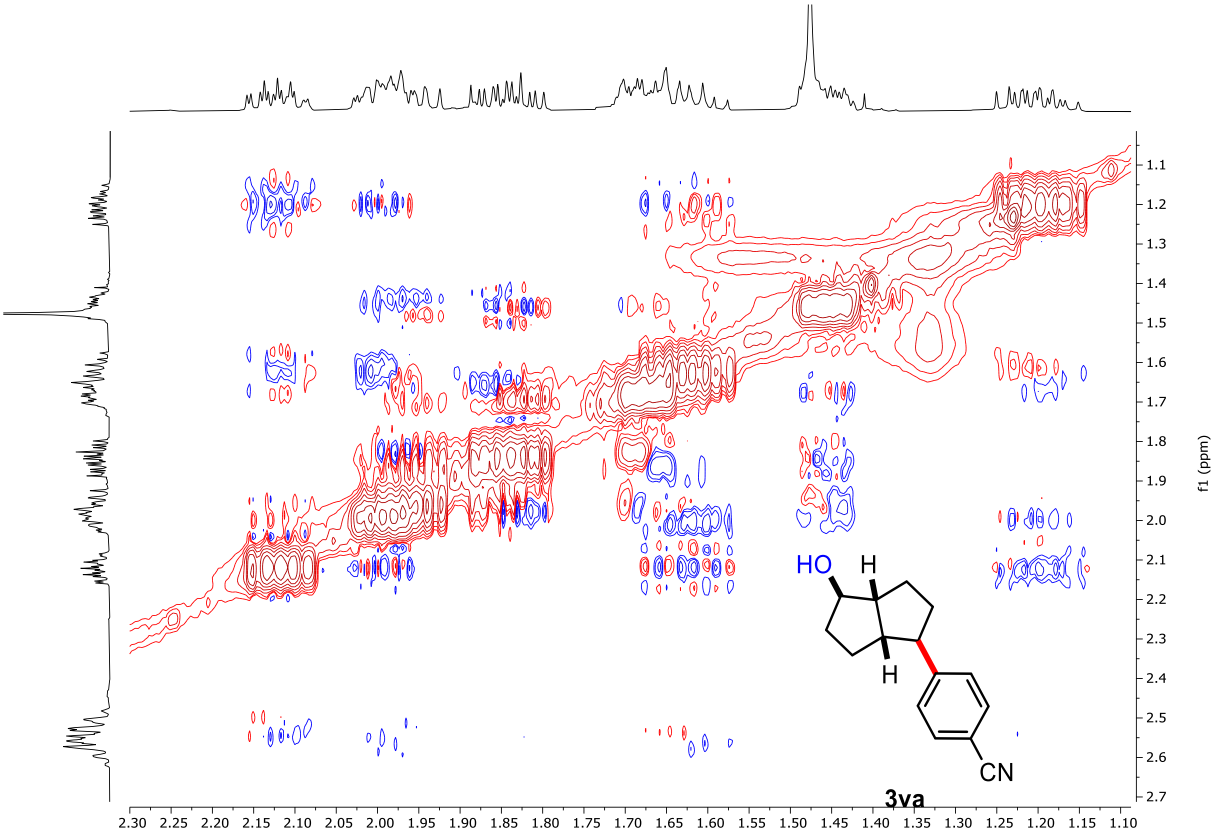

^{13}C NMR (101 MHz, $CDCl_3$) of **3va**

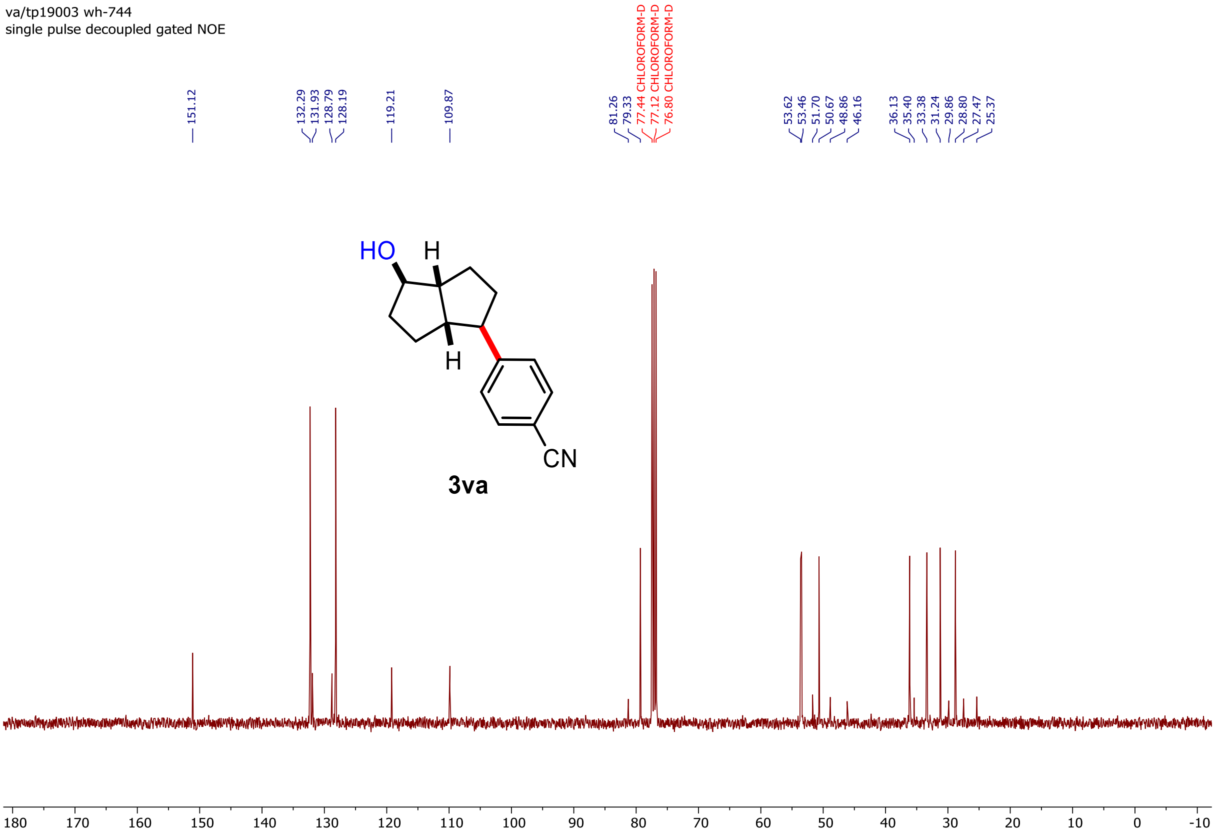

^{1}H NMR (400 MHz, $CDCl_3$) of **3wa** (*see procedure*)

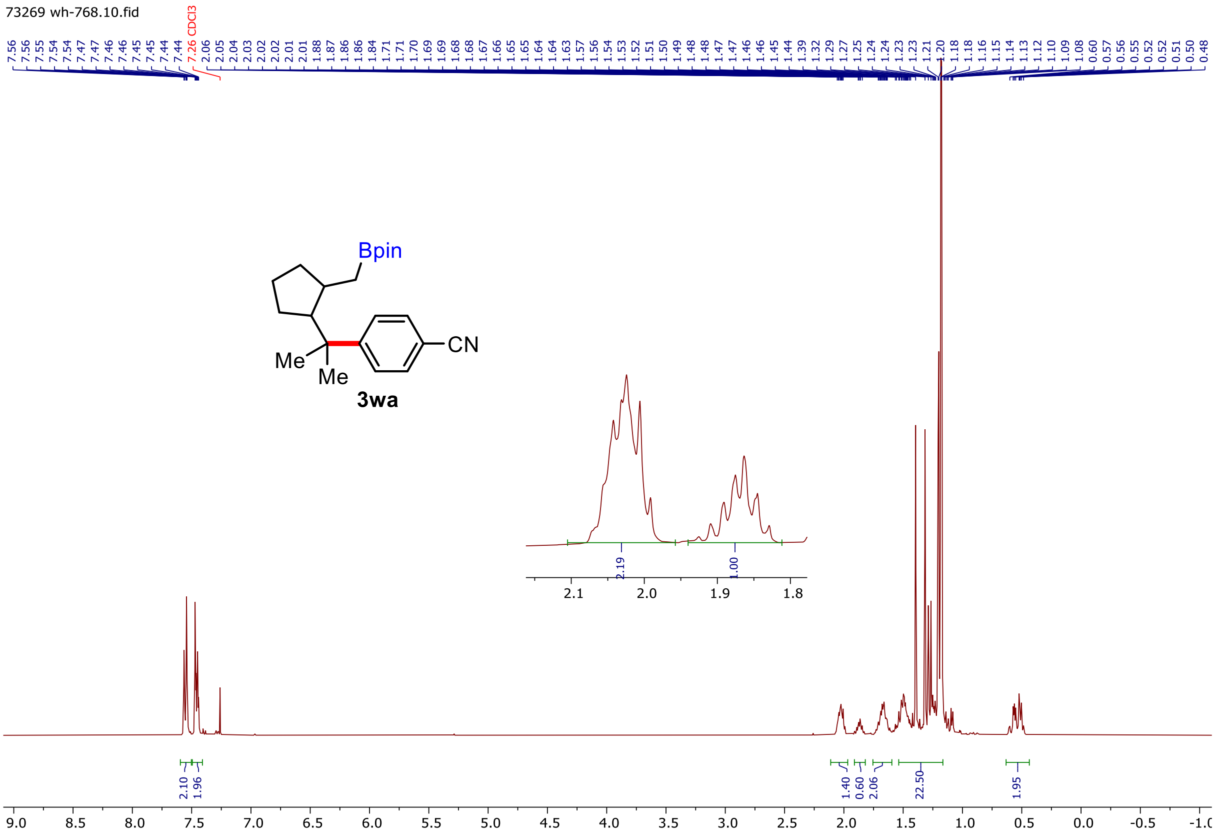

^{13}C NMR (101 MHz, $CDCl_3$) of **3wa**

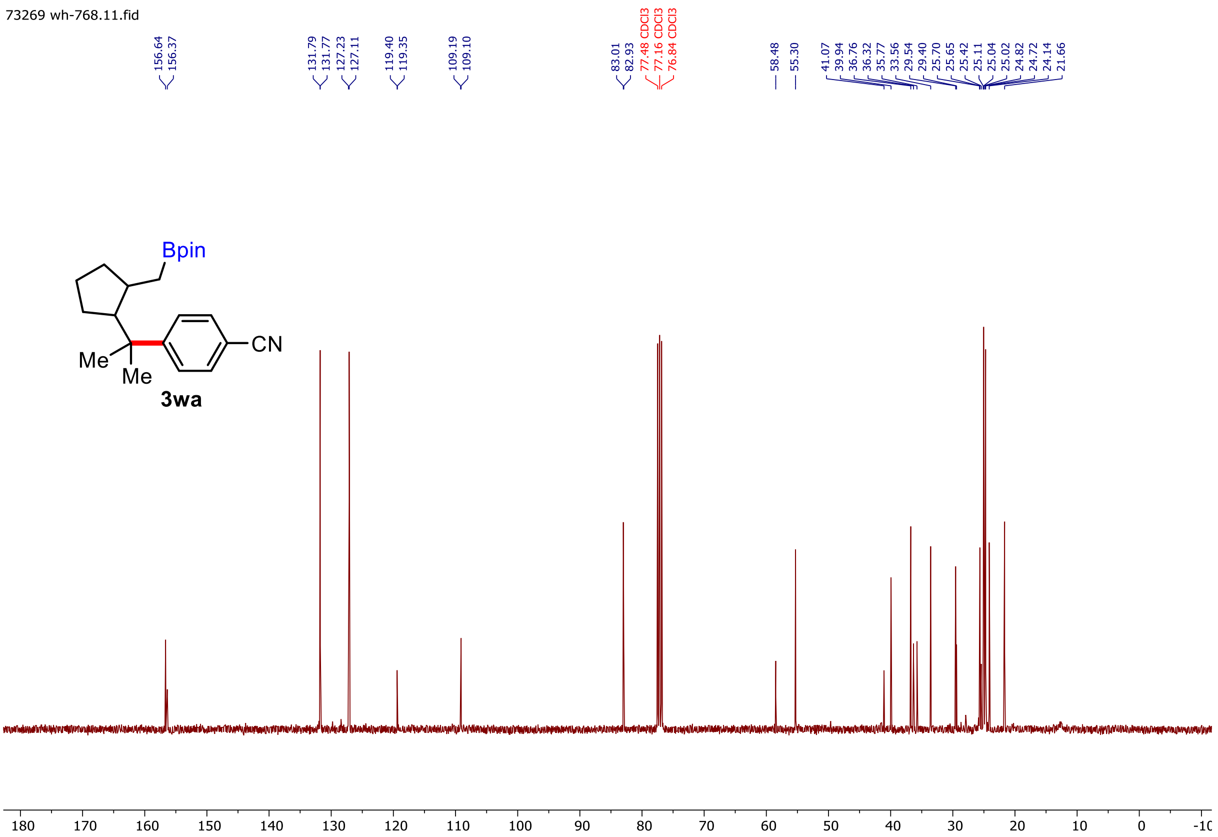

^{11}B NMR (128 MHz, $CDCl_3$) of **3va**

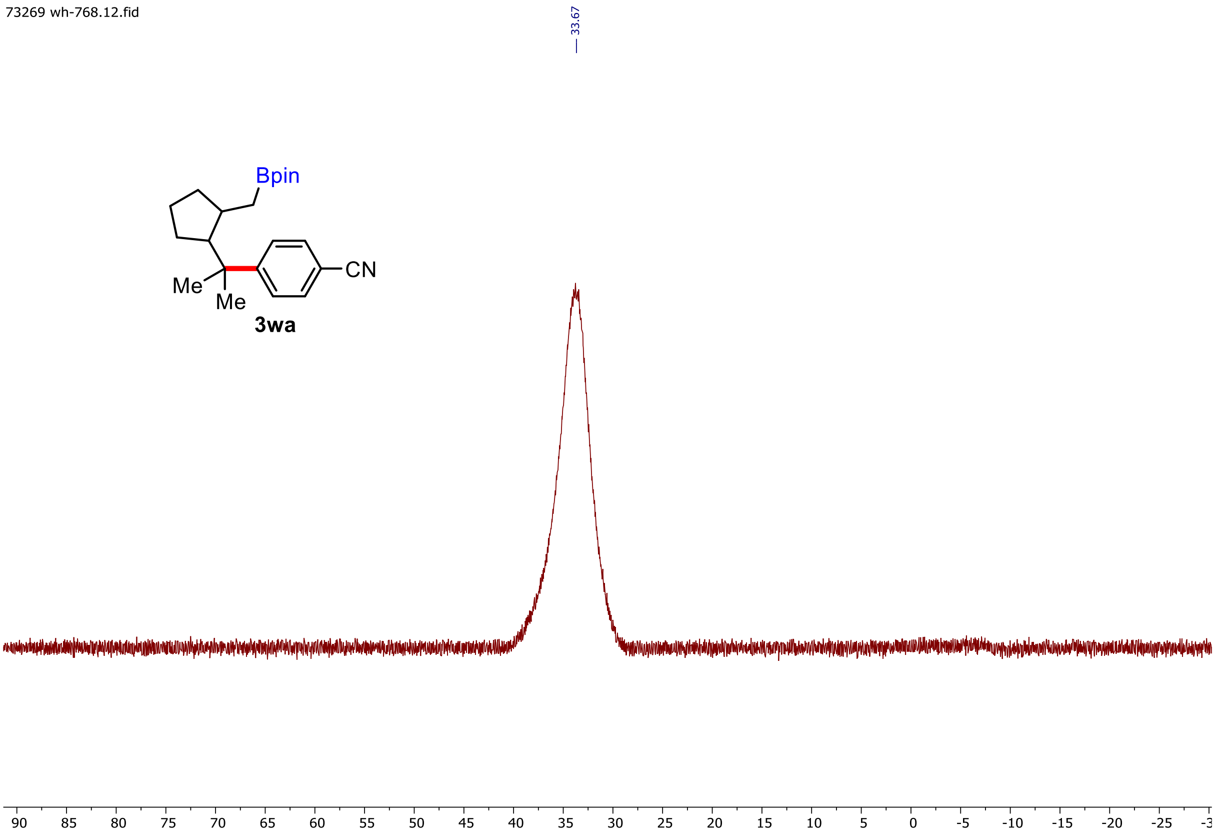

^{1}H NMR (400 MHz, $CDCl_3$) of **3ab** (*see procedure*)

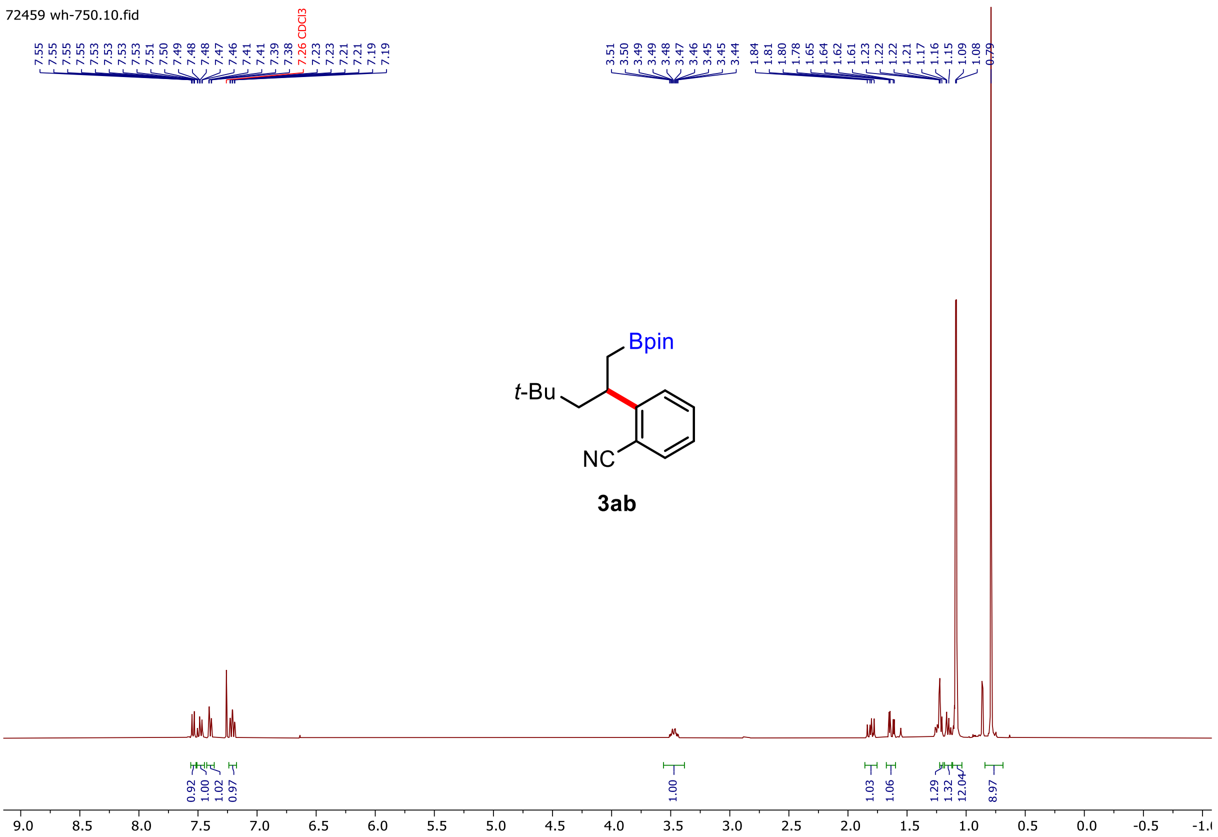

^{13}C NMR (101 MHz, $CDCl_3$) of **3ab**

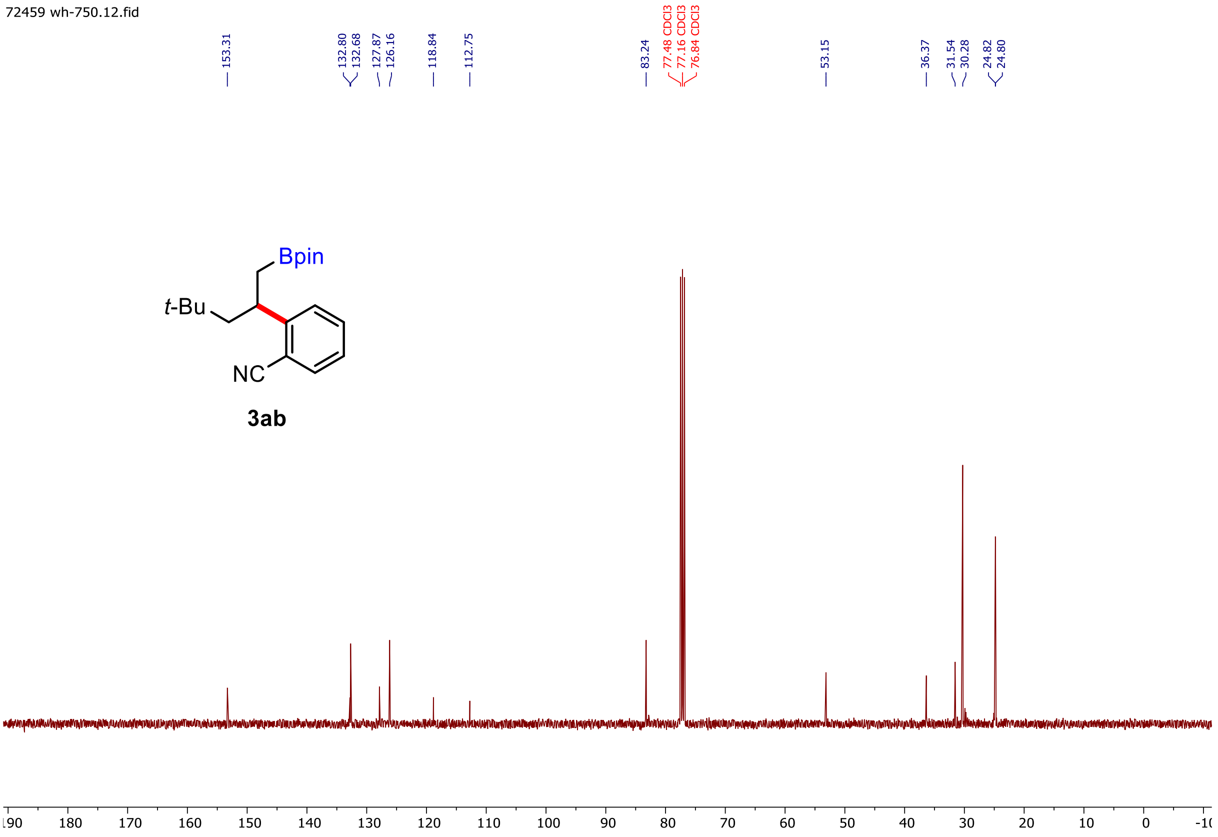

^{11}B NMR (128 MHz, $CDCl_3$) of **3ab**

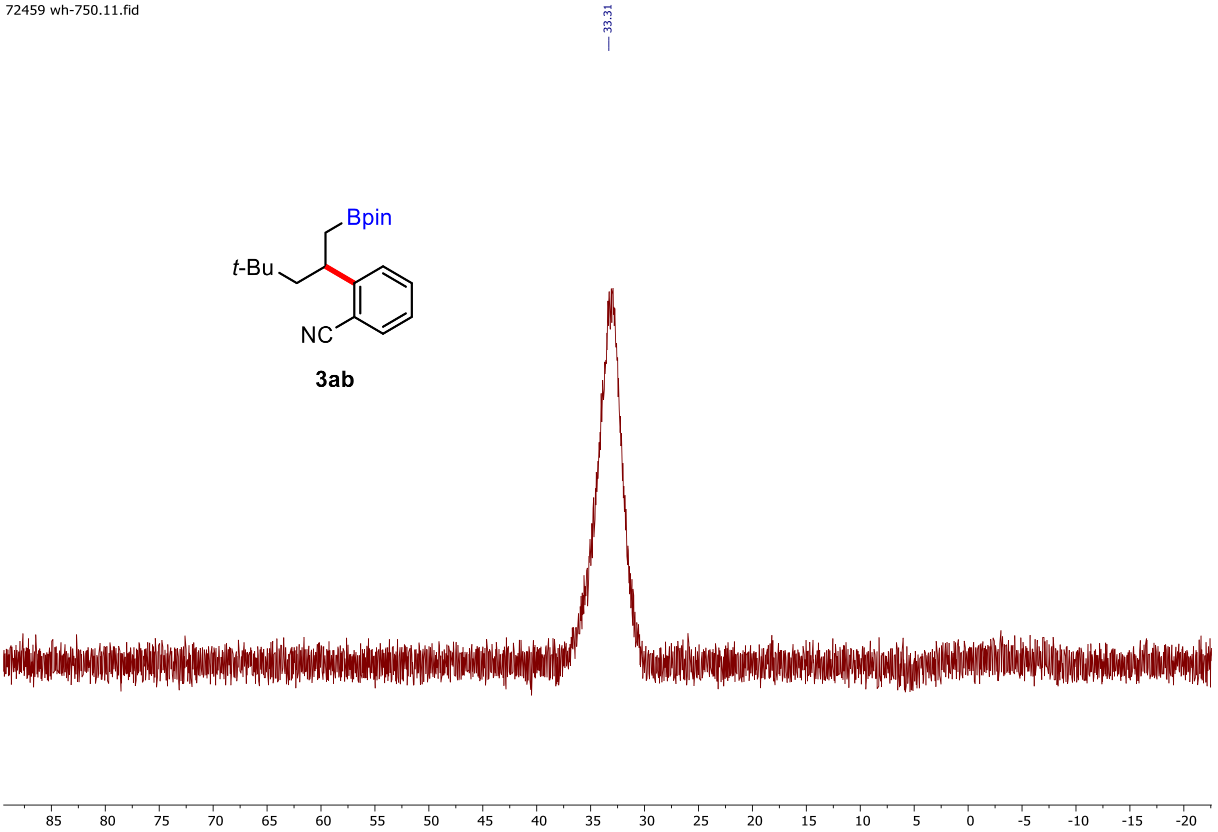

^{1}H NMR (400 MHz, $CDCl_3$) of **3ac** (*see procedure*)

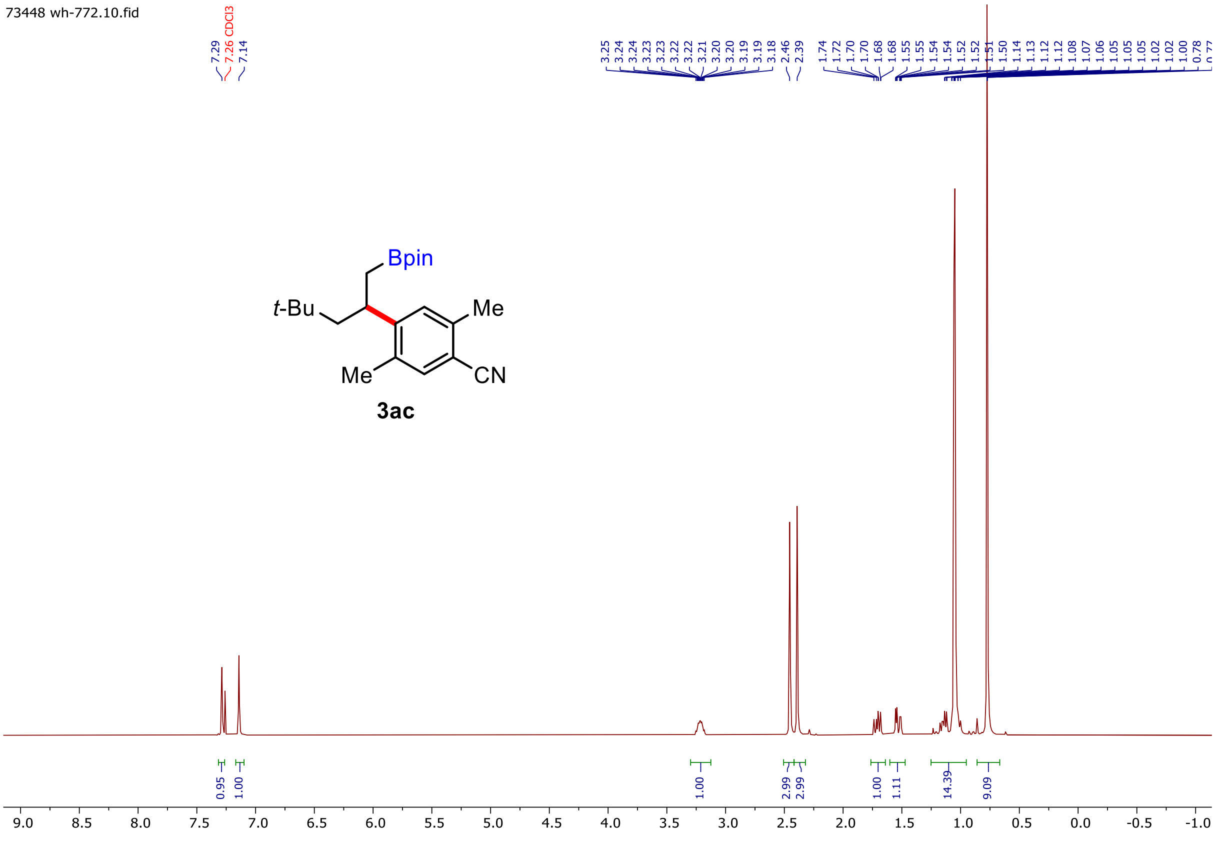

^{13}C NMR (101 MHz, CDCl$_3$) of **3ac**

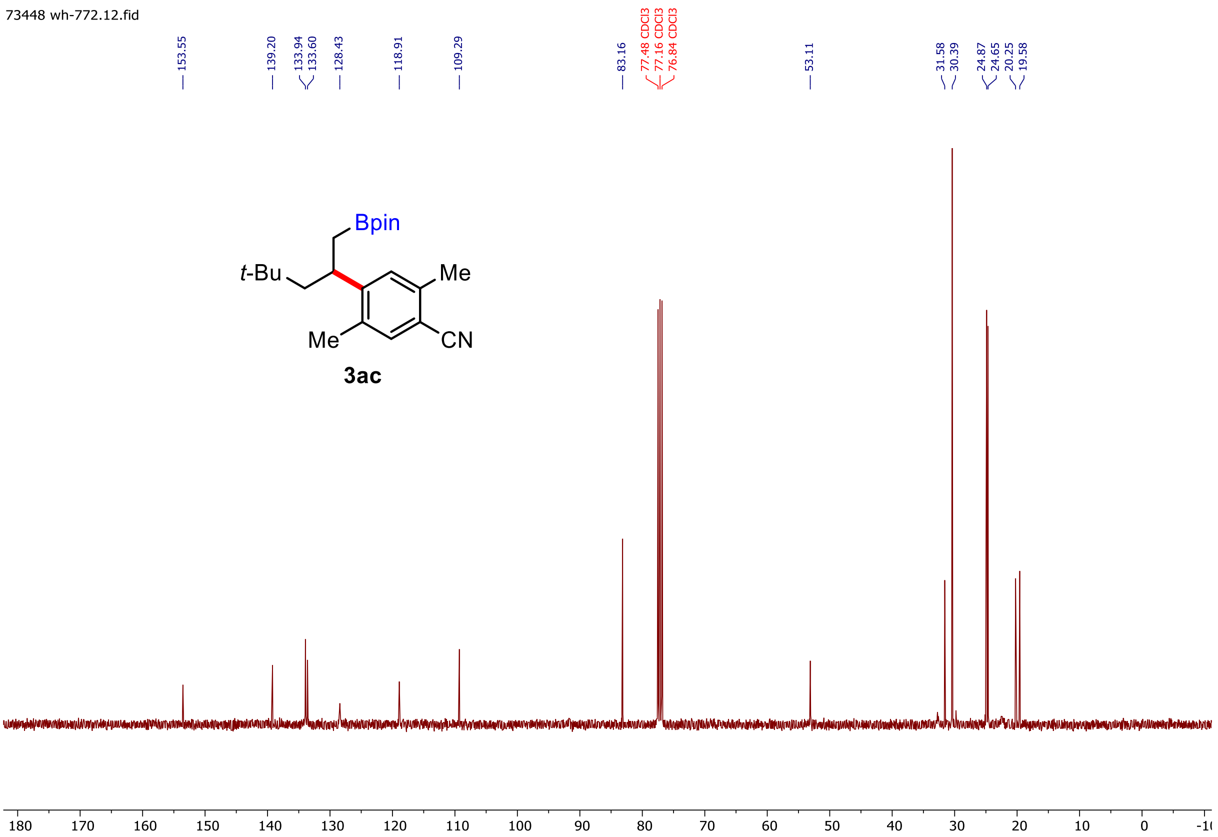

^{11}B NMR (128 MHz, CDCl$_3$) of **3ac**

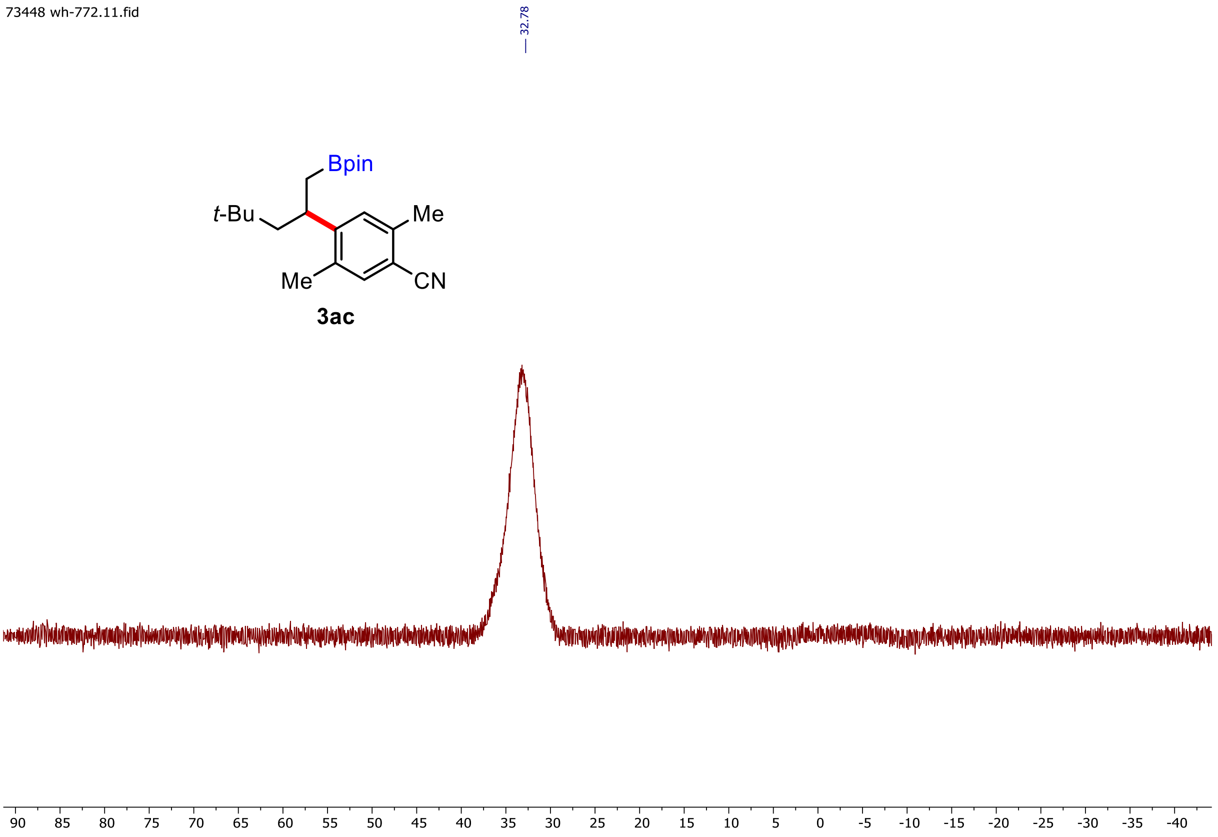

^{1}H NMR (400 MHz, $CDCl_3$) of **3ad** (*see procedure*)

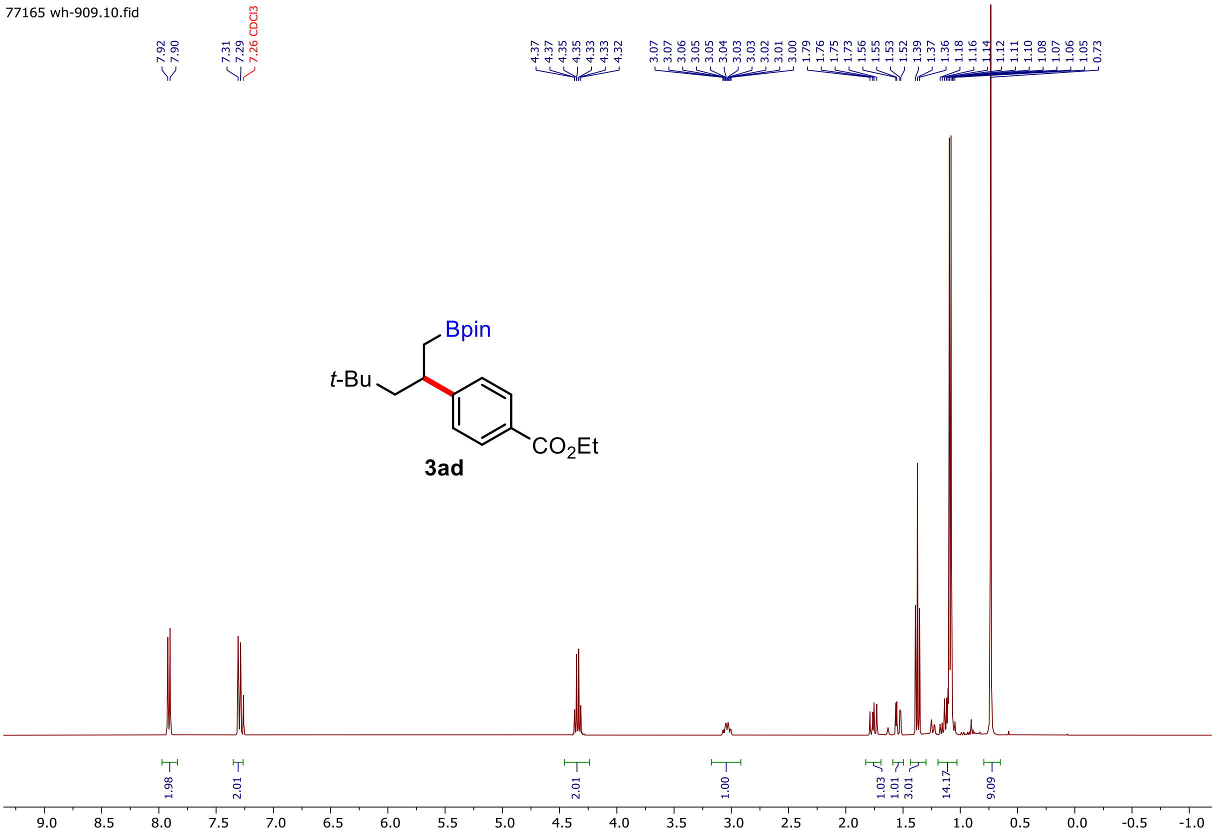

^{13}C NMR (101 MHz, $CDCl_3$) of **3ad**

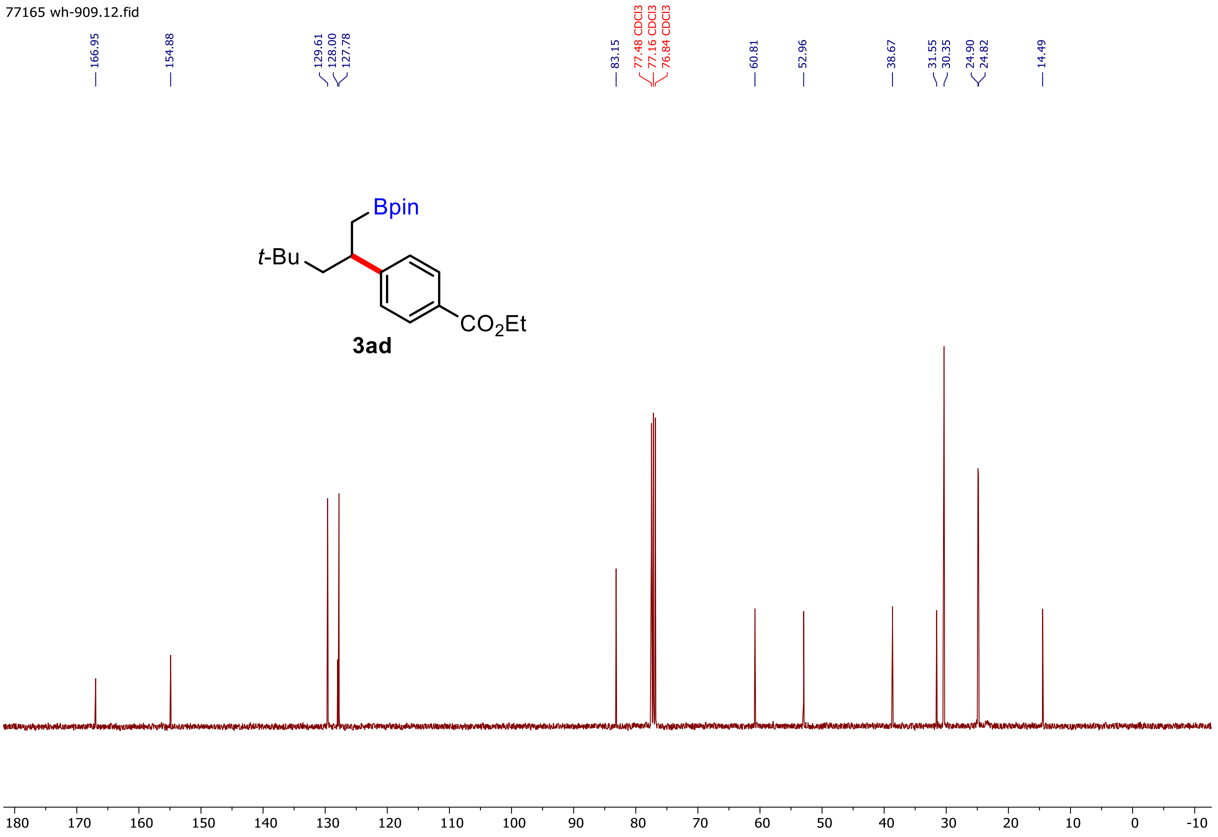

^{11}B NMR (128 MHz, $CDCl_3$) of **3ad**

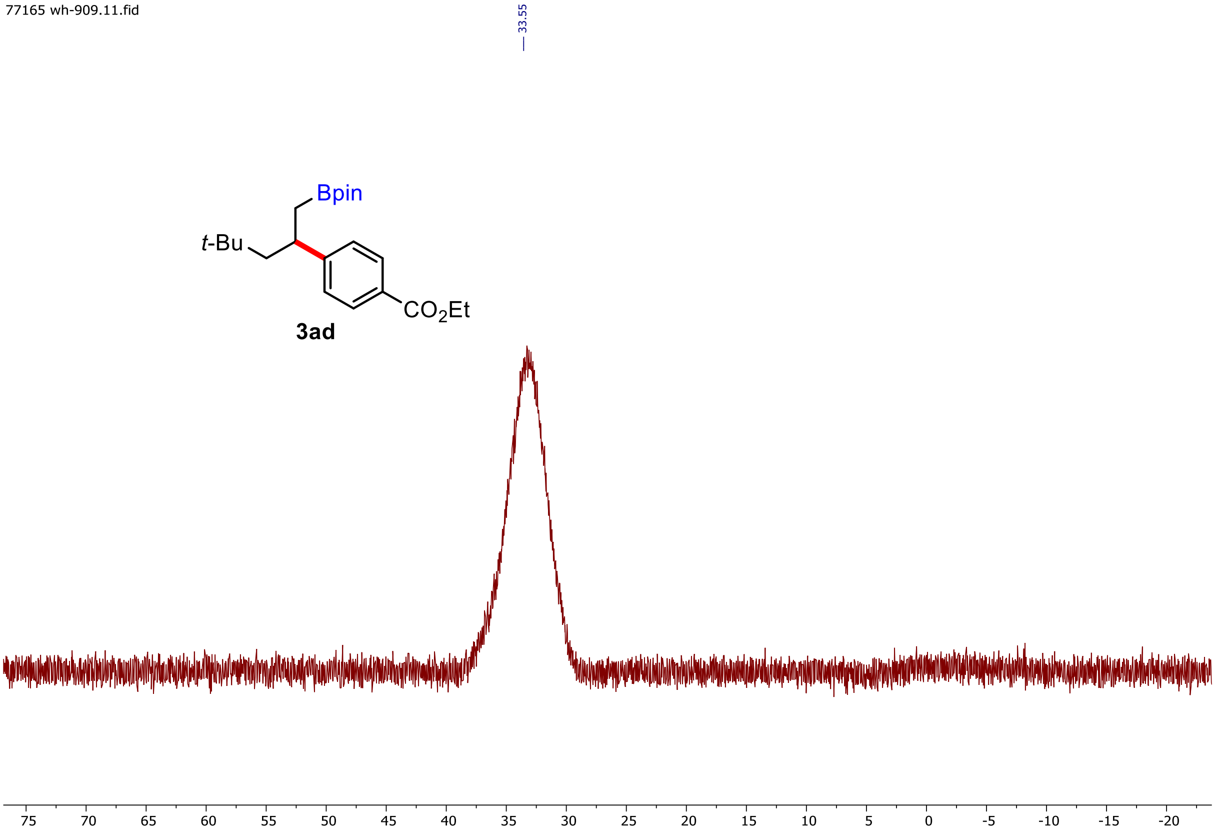

^{1}H NMR (400 MHz, $CDCl_3$) of **3ae** (*see procedure*)

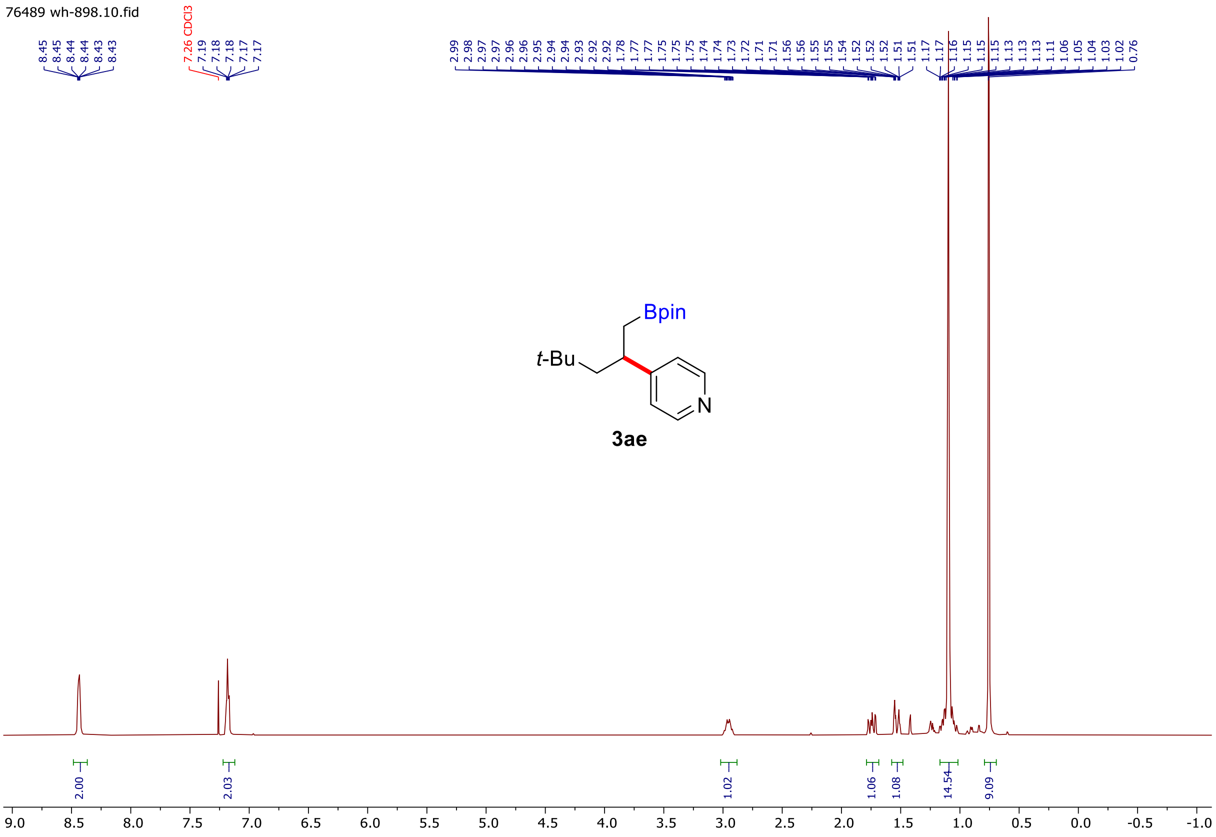

UOB Confidential & Sensitive

^{13}C NMR (101 MHz, $CDCl_3$) of **3ae**

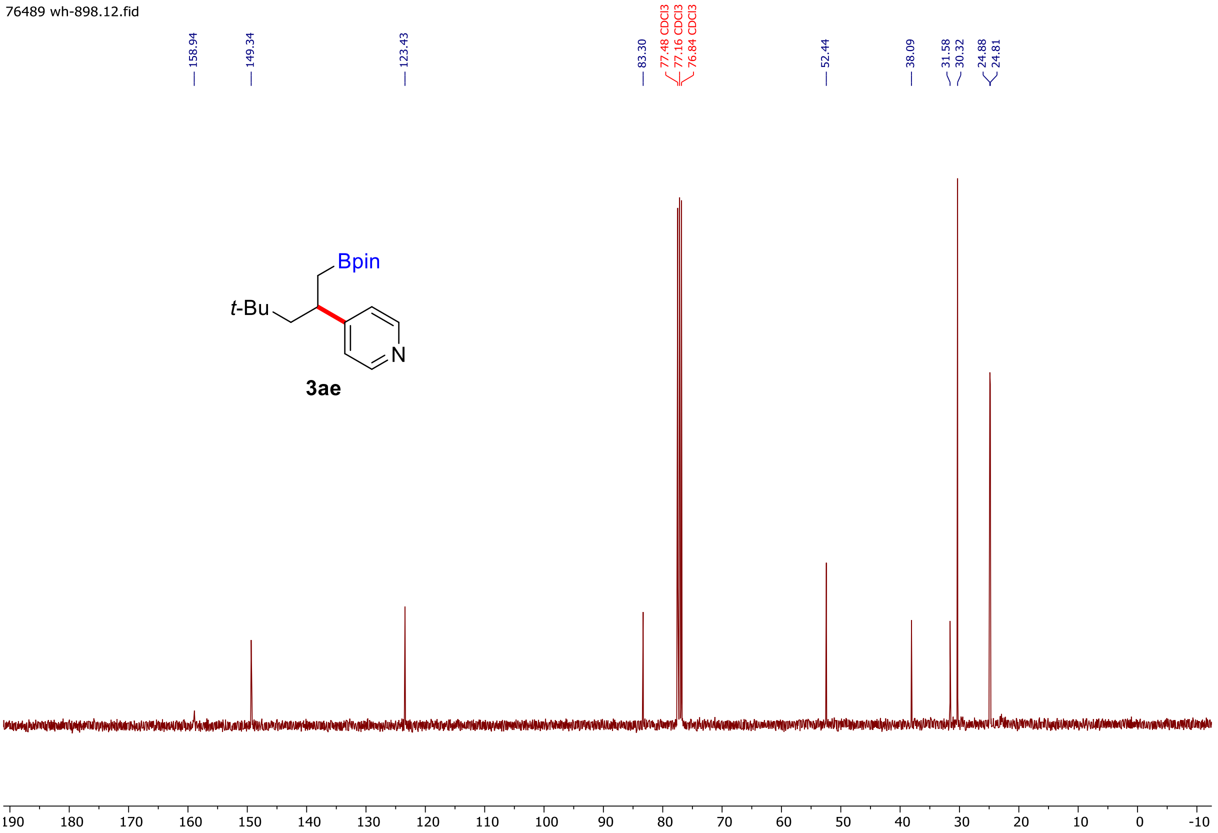

^{11}B NMR (128 MHz, $CDCl_3$) of **3ae**

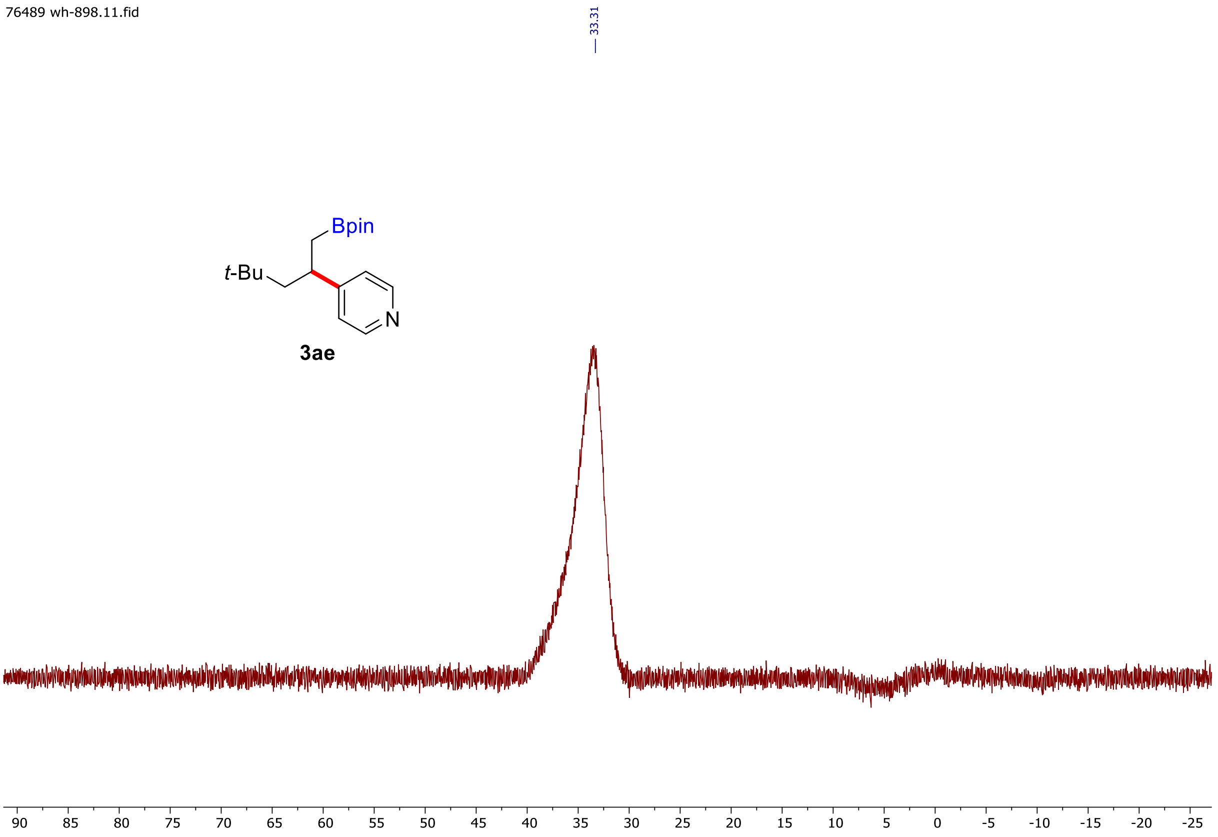

^{1}H NMR (400 MHz, $CDCl_3$) of **3af** (*see procedure*)

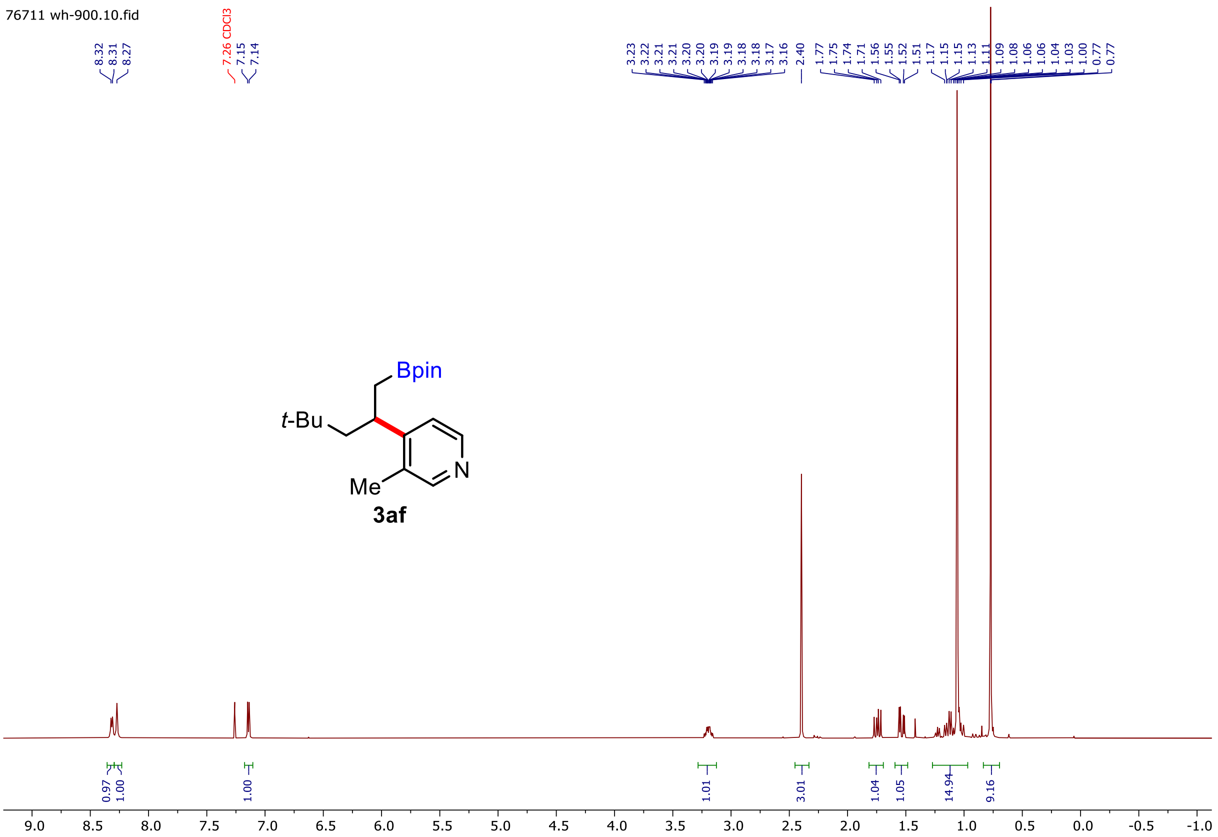

^{13}C NMR (101 MHz, $CDCl_3$) of **3af**

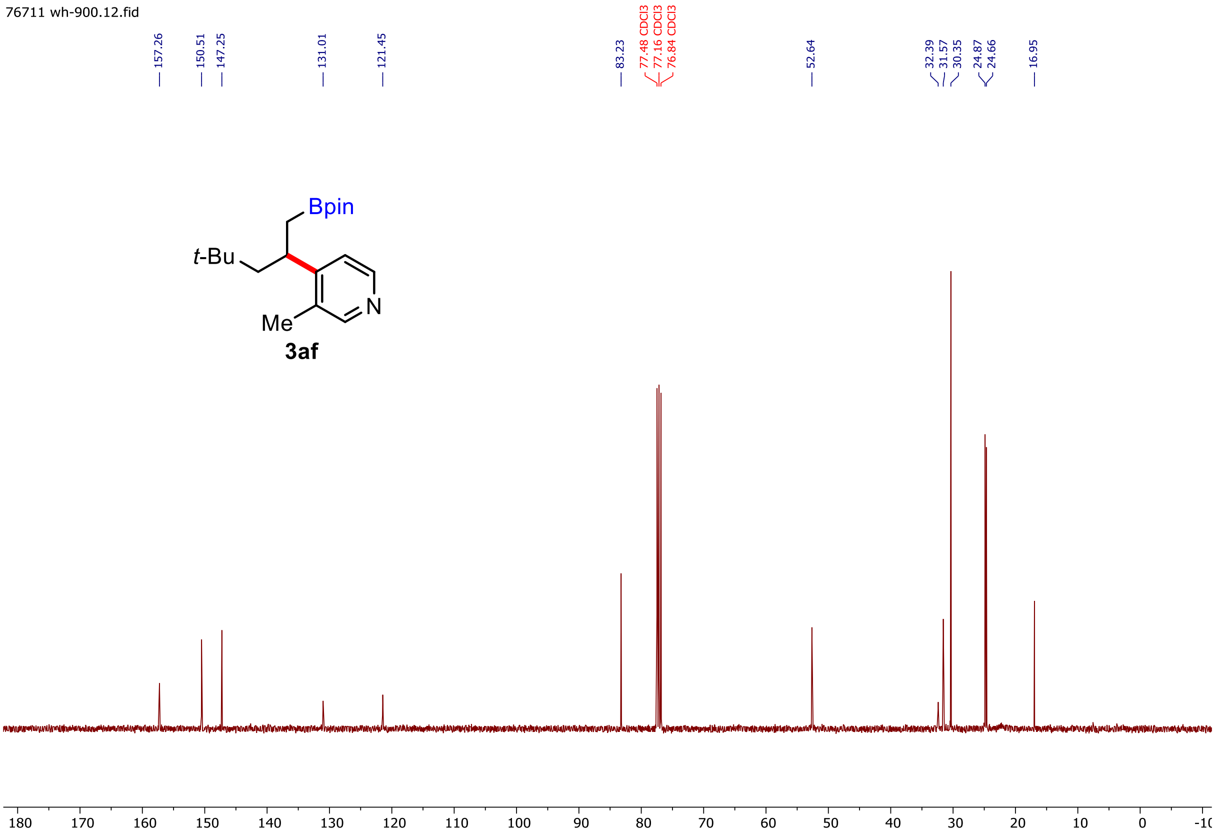

^{11}B NMR (128 MHz, $CDCl_3$) of **3af**

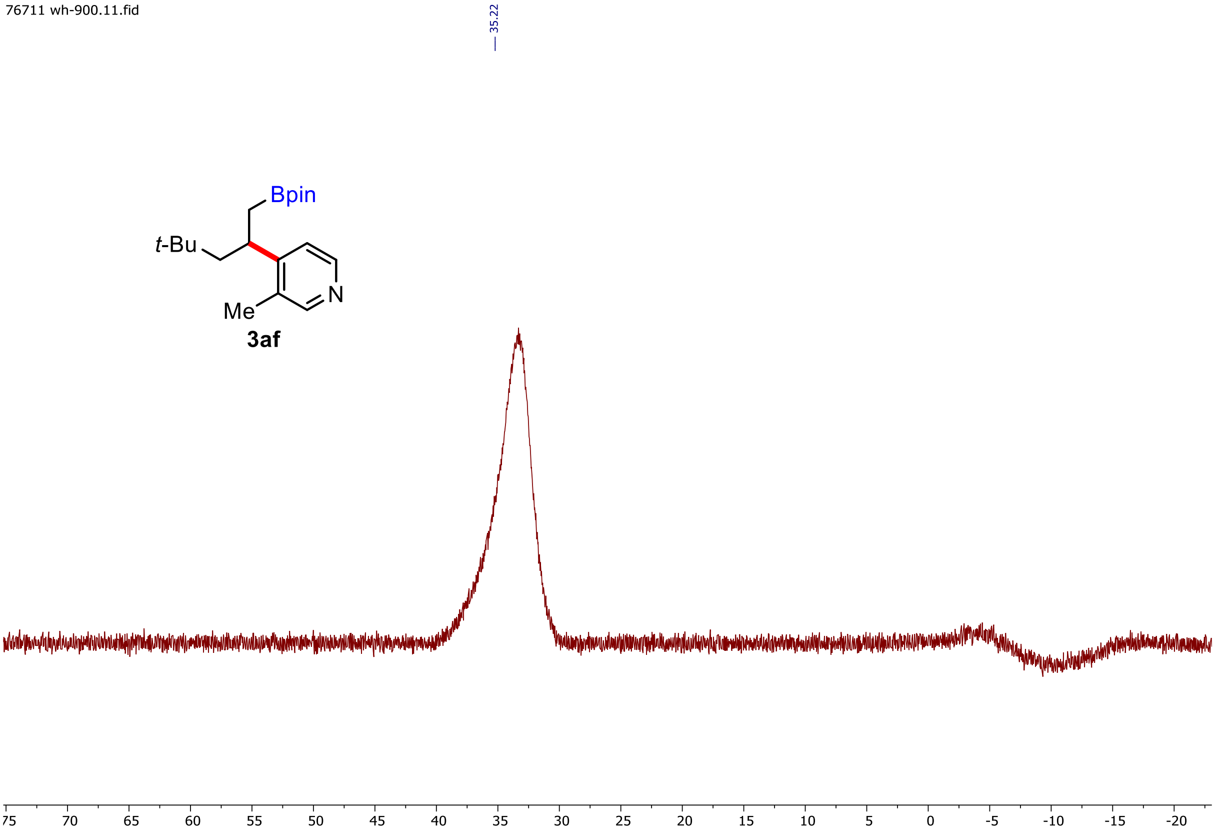

^{1}H NMR (400 MHz, $CDCl_3$) of **3ag** (*see procedure*)

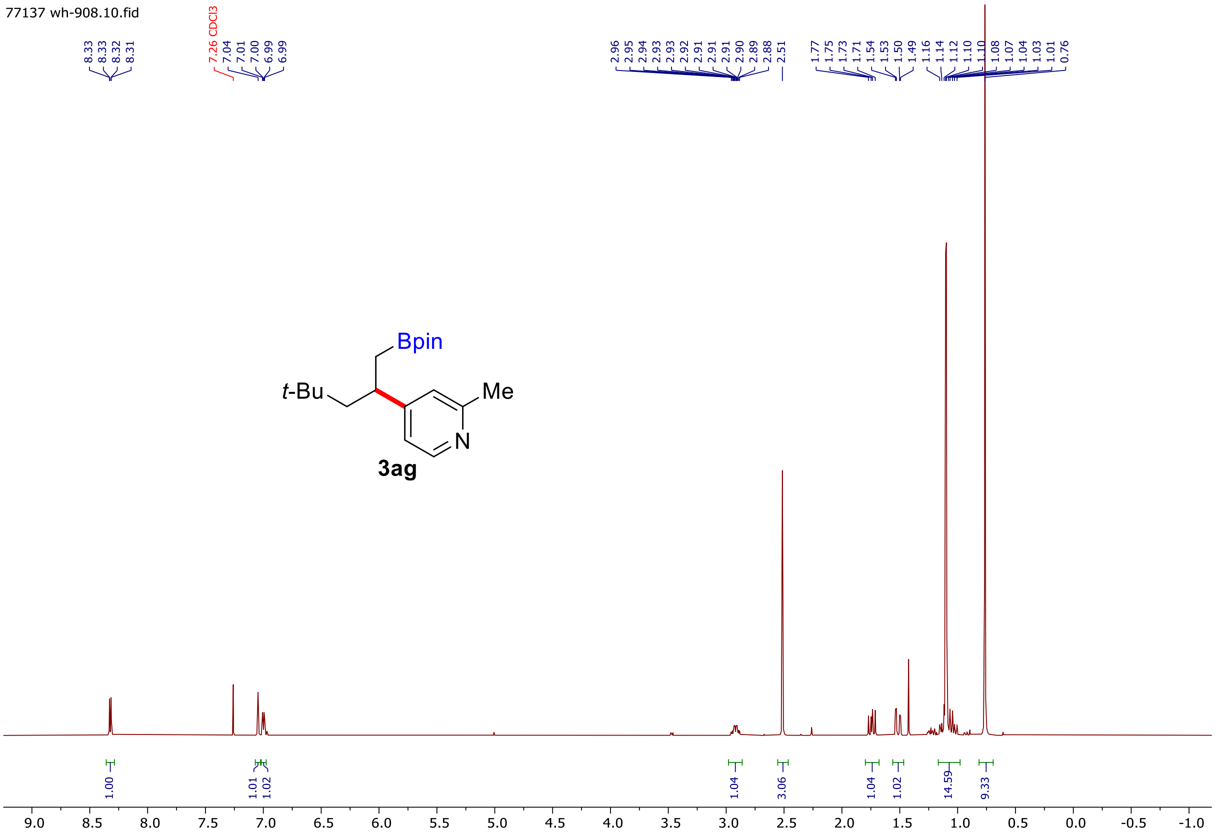

UOB Confidential & Sensitive

^{13}C NMR (101 MHz, $CDCl_3$) of **3ag**

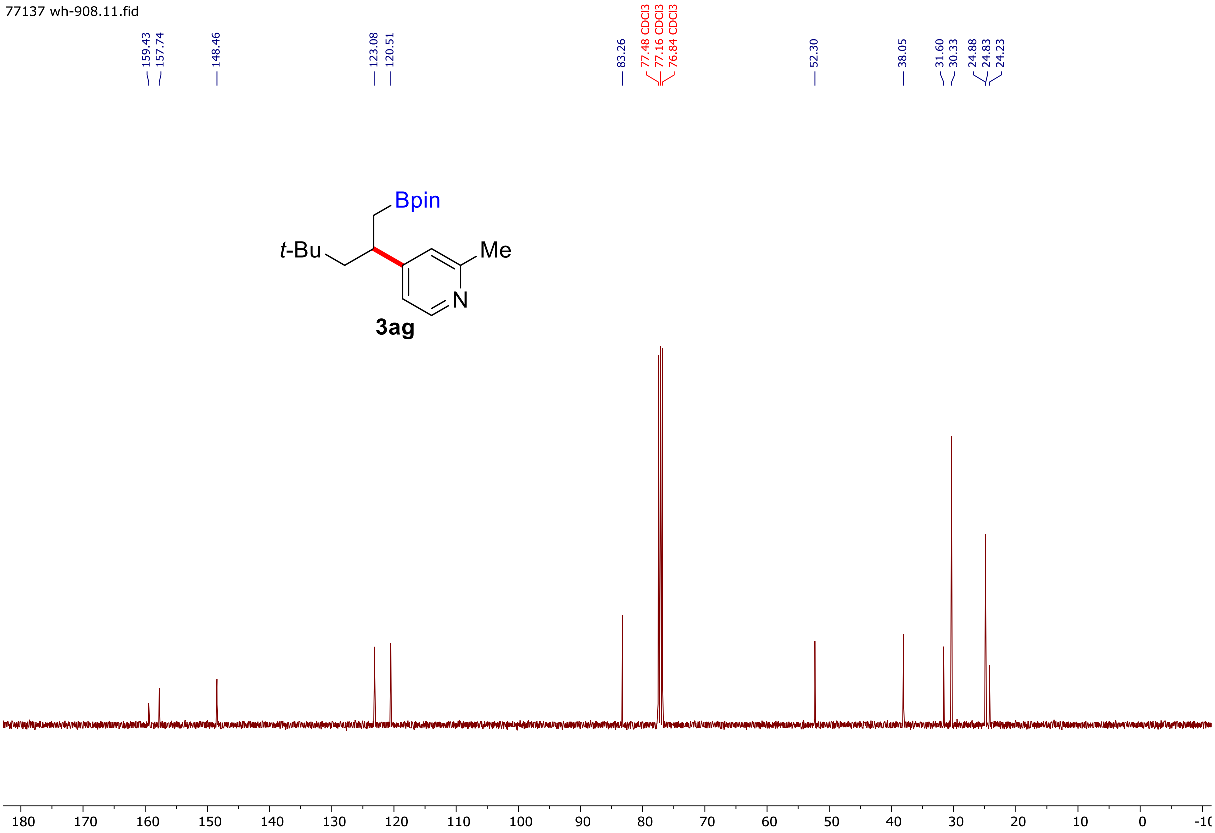

^{11}B NMR (128 MHz, $CDCl_3$) of **3ag**

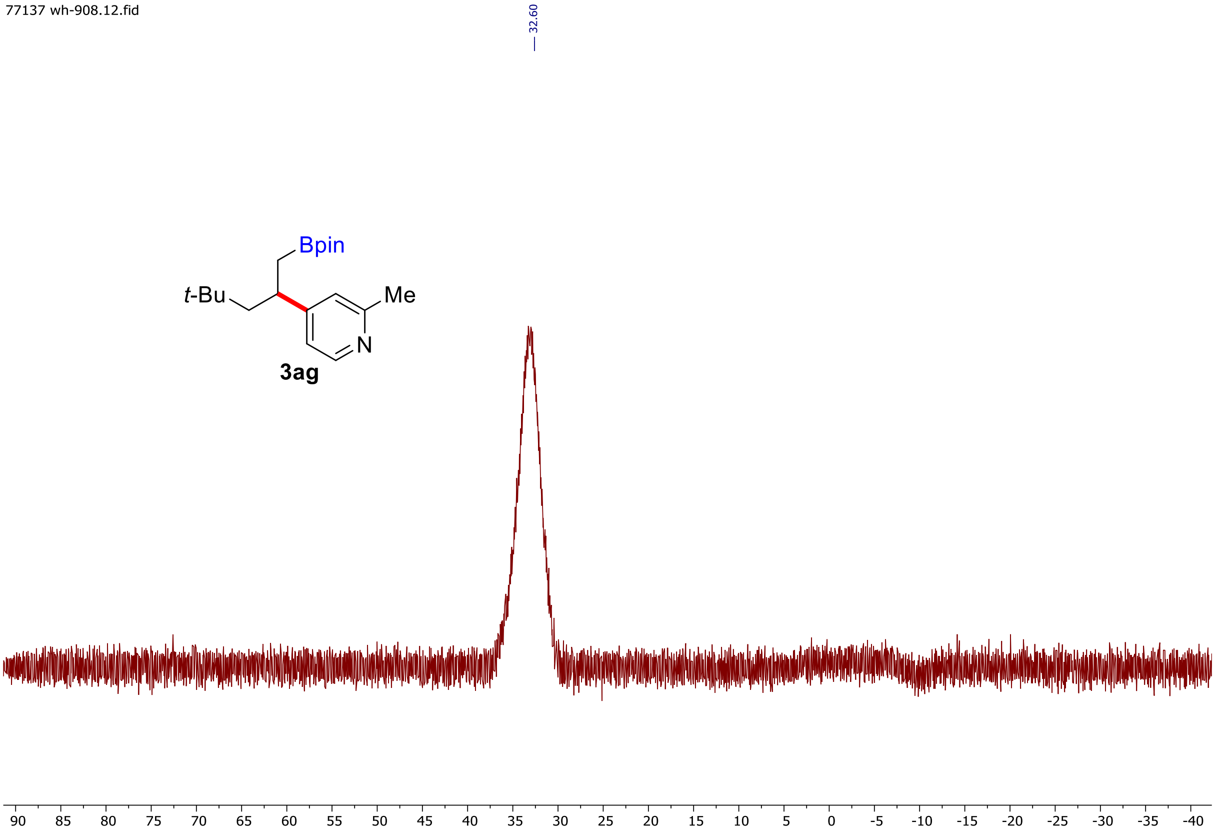

^{1}H NMR (400 MHz, $CDCl_3$) of **3ah** (*see procedure*)

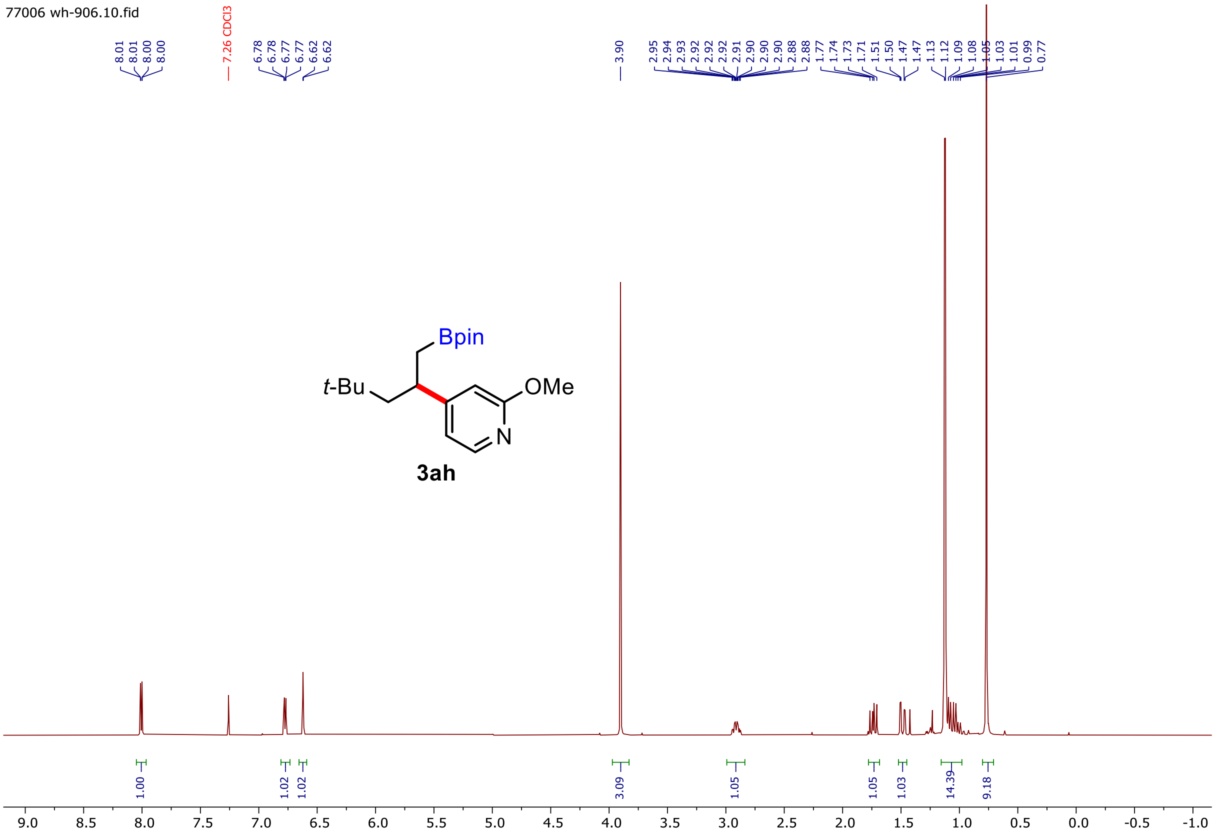

^{13}C NMR (101 MHz, $CDCl_3$) of **3ah**

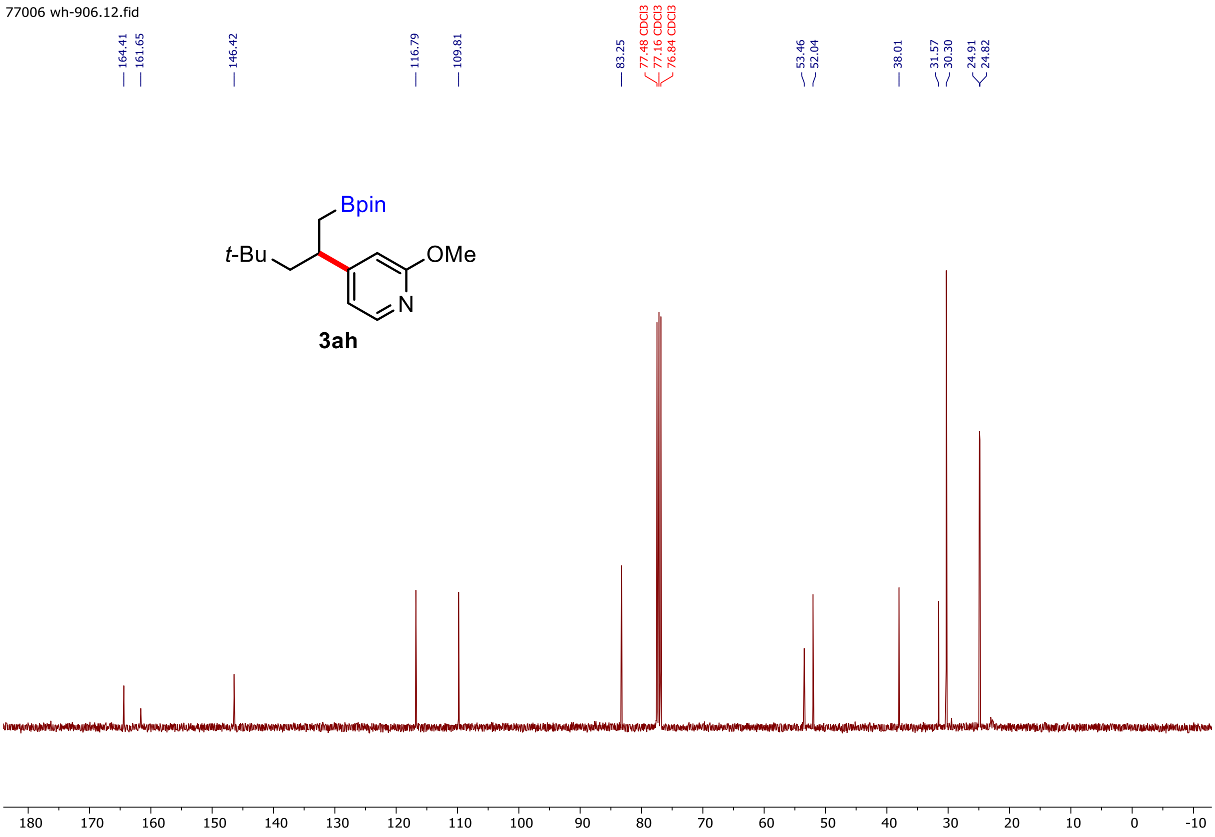

UOB Confidential & Sensitive

^{11}B NMR (128 MHz, $CDCl_3$) of **3ah**

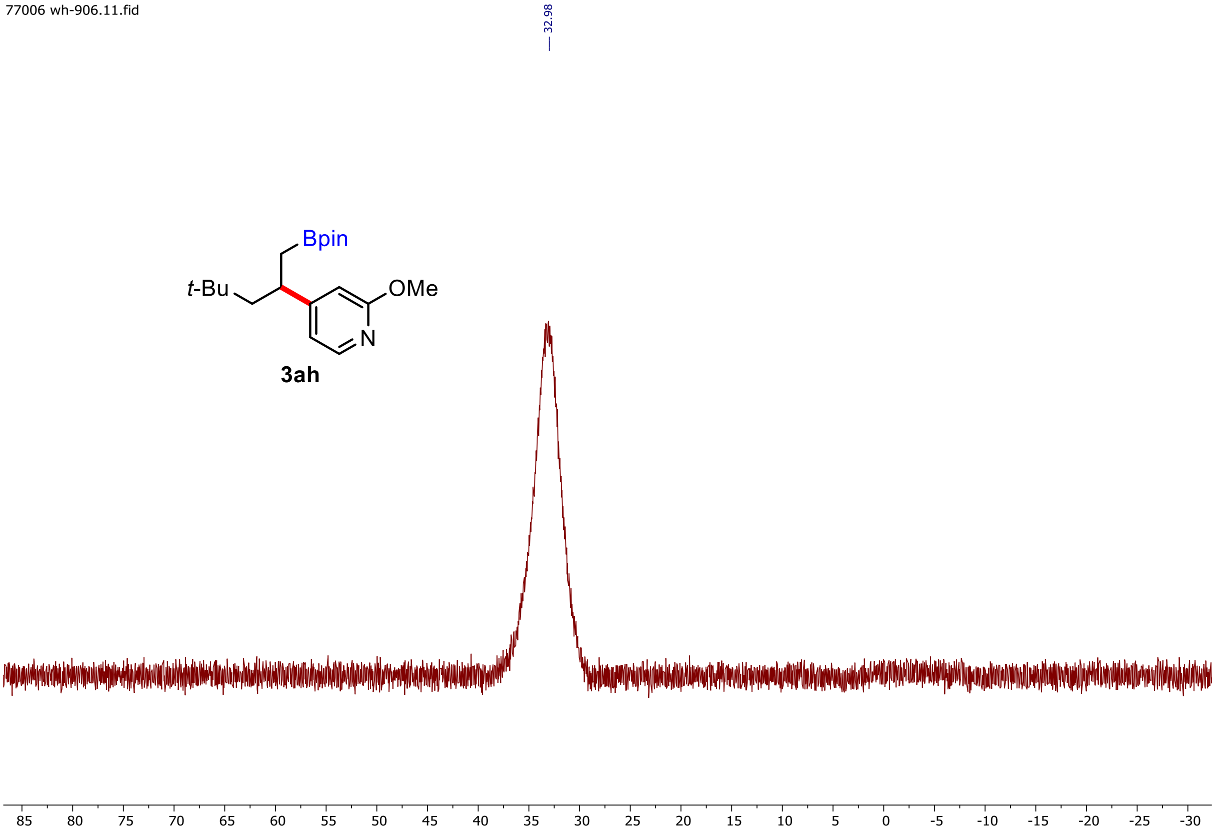

^{1}H NMR (400 MHz, $CDCl_3$) of **3ai** (*see procedure*)

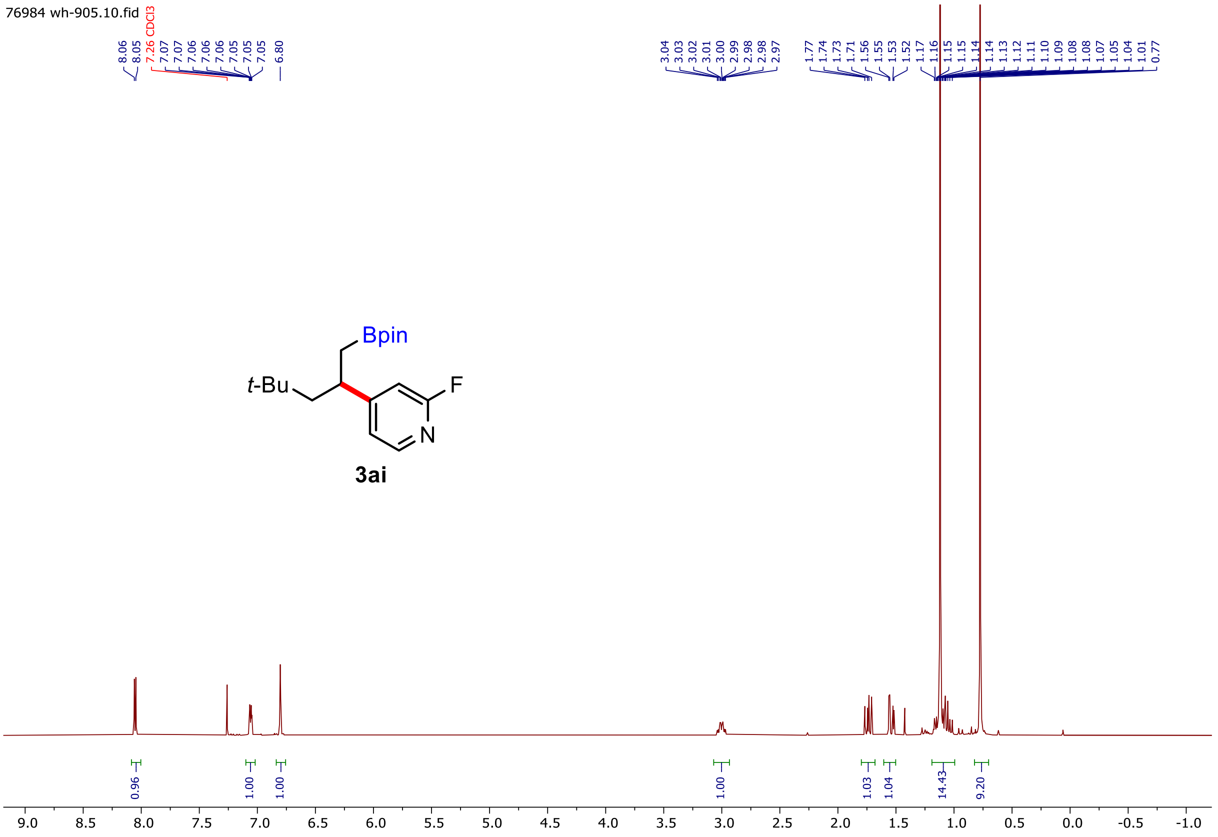

^{13}C NMR (101 MHz, $CDCl_3$) of **3ai**

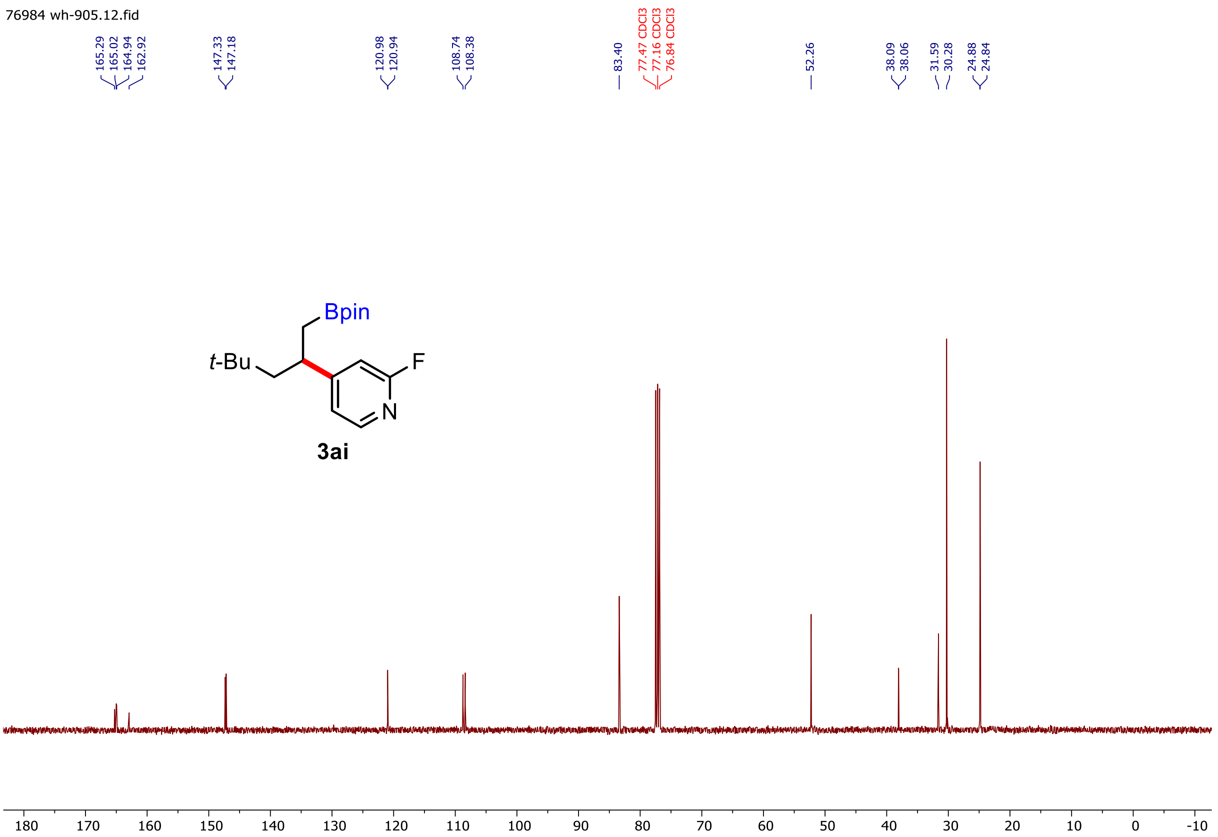

^{19}F NMR (377 MHz, $CDCl_3$) of **3ai**

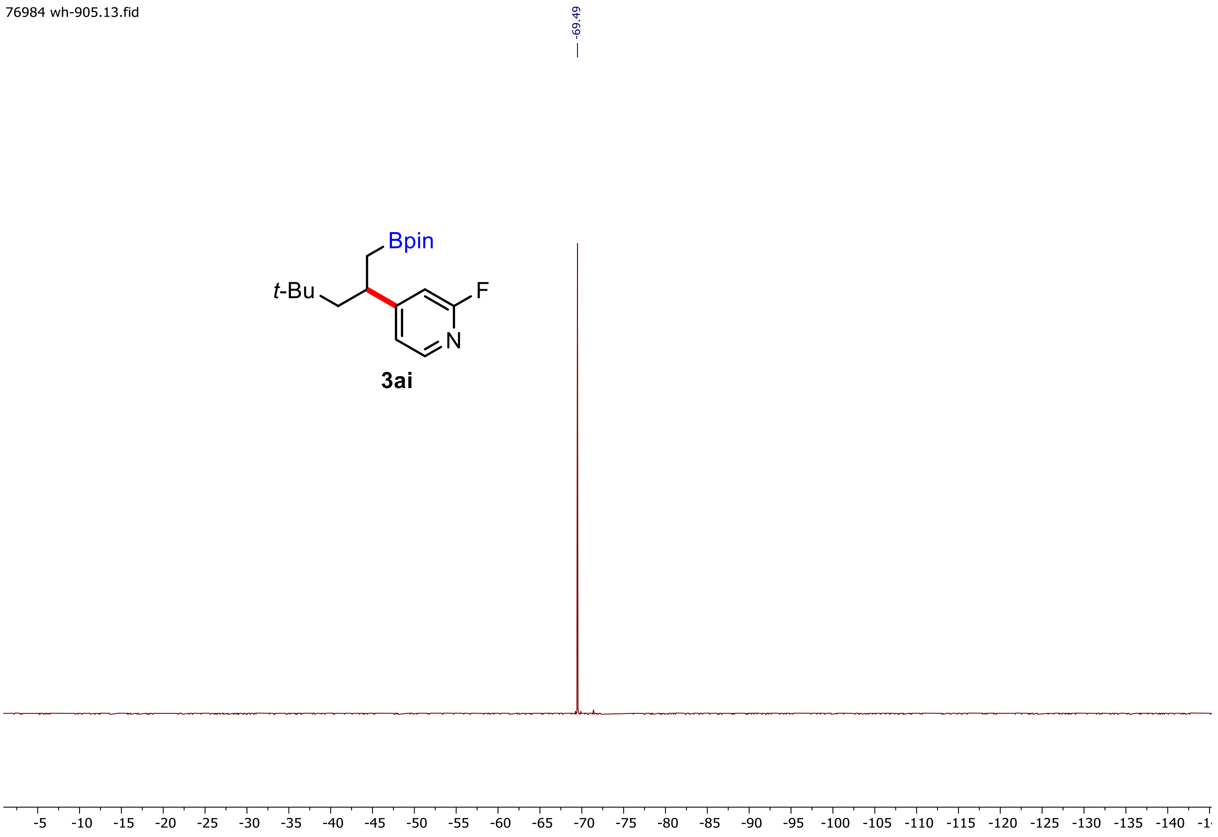

^{11}B NMR (128 MHz, $CDCl_3$) of **3ai**

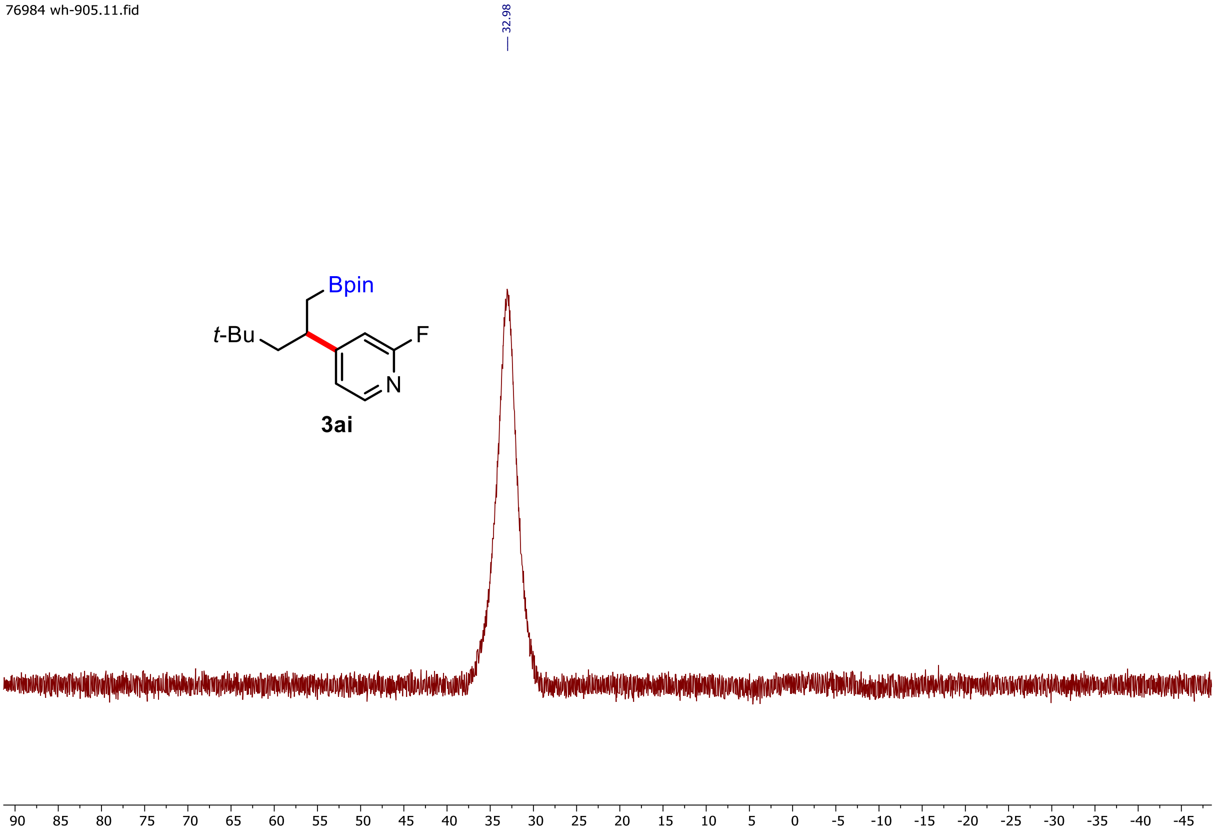

^{1}H NMR (400 MHz, $CDCl_3$) of **3aj** (*see procedure*)

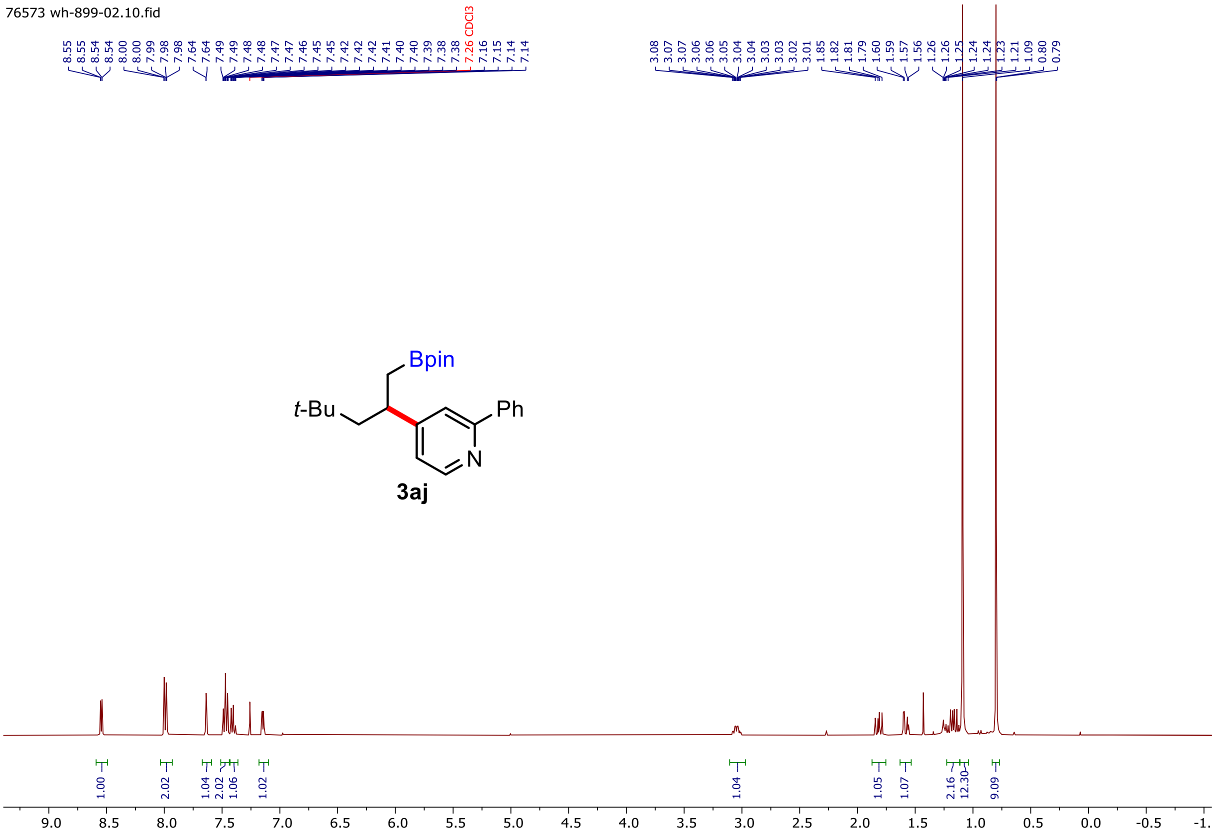

^{13}C NMR (101 MHz, $CDCl_3$) of **3aj**

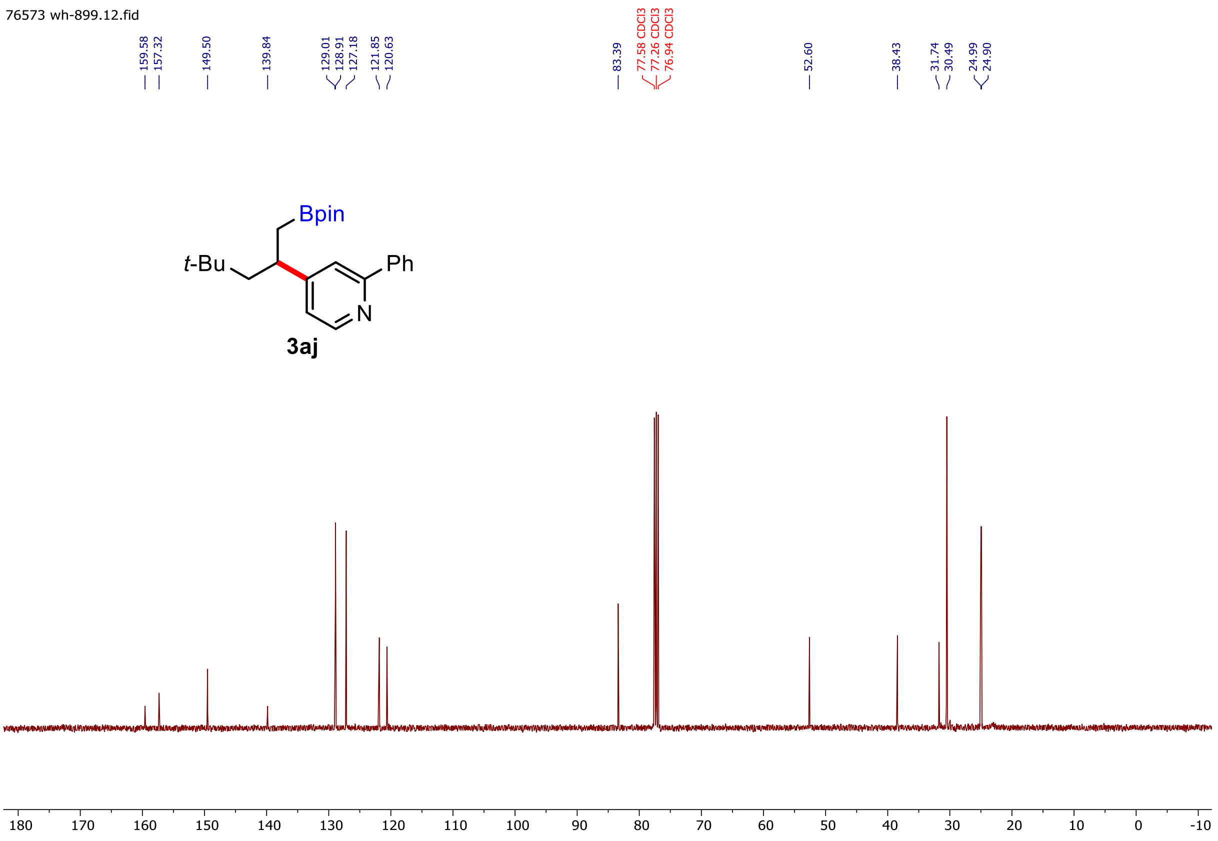

^{11}B NMR (128 MHz, $CDCl_3$) of **3aj**

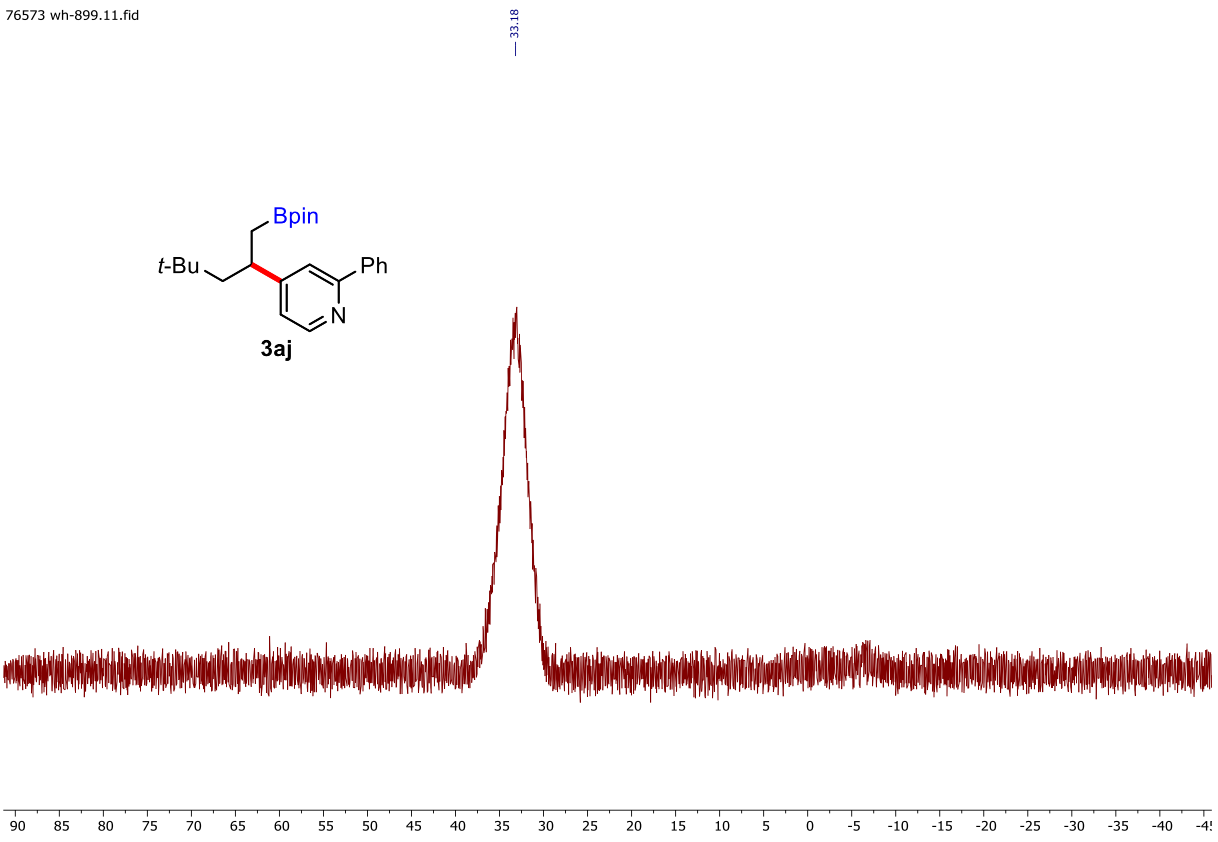

^{1}H NMR (400 MHz, $CDCl_3$) of **3ak** (*see procedure*)

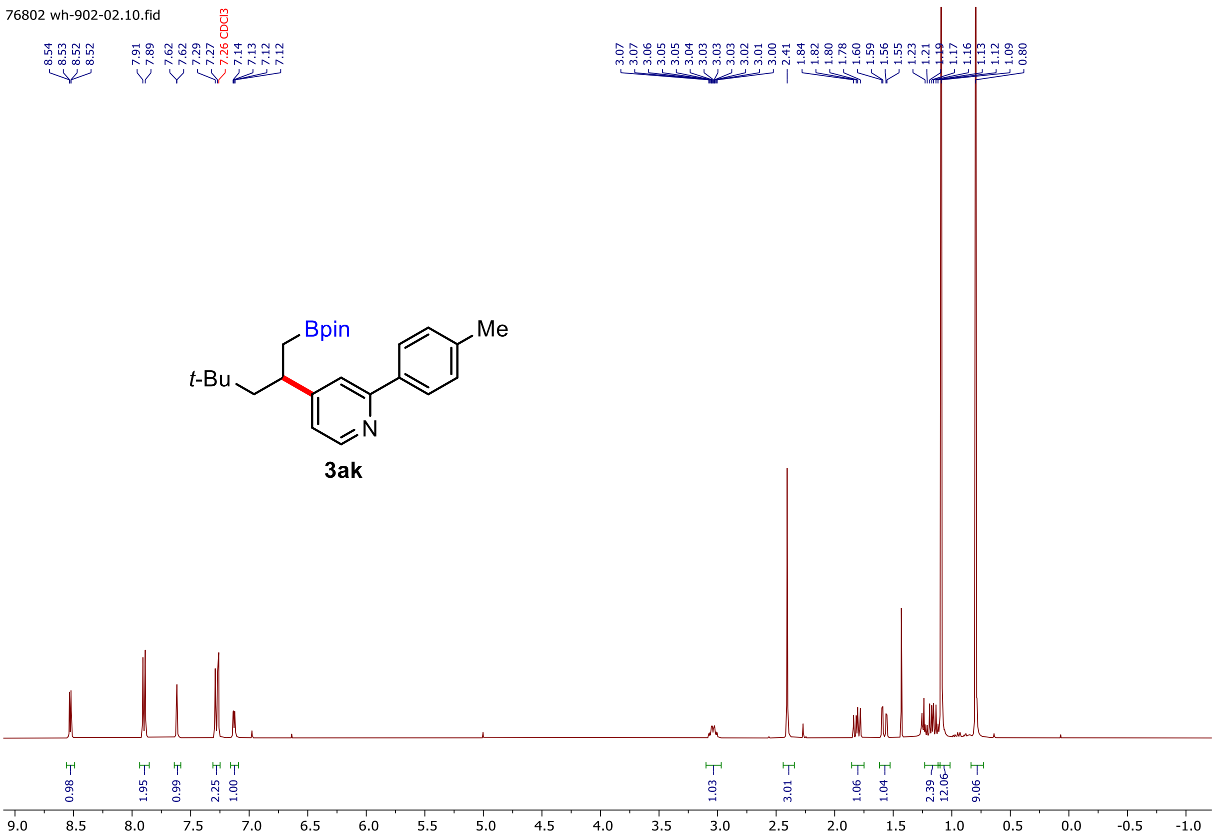

^{13}C NMR (101 MHz, $CDCl_3$) of **3ak**

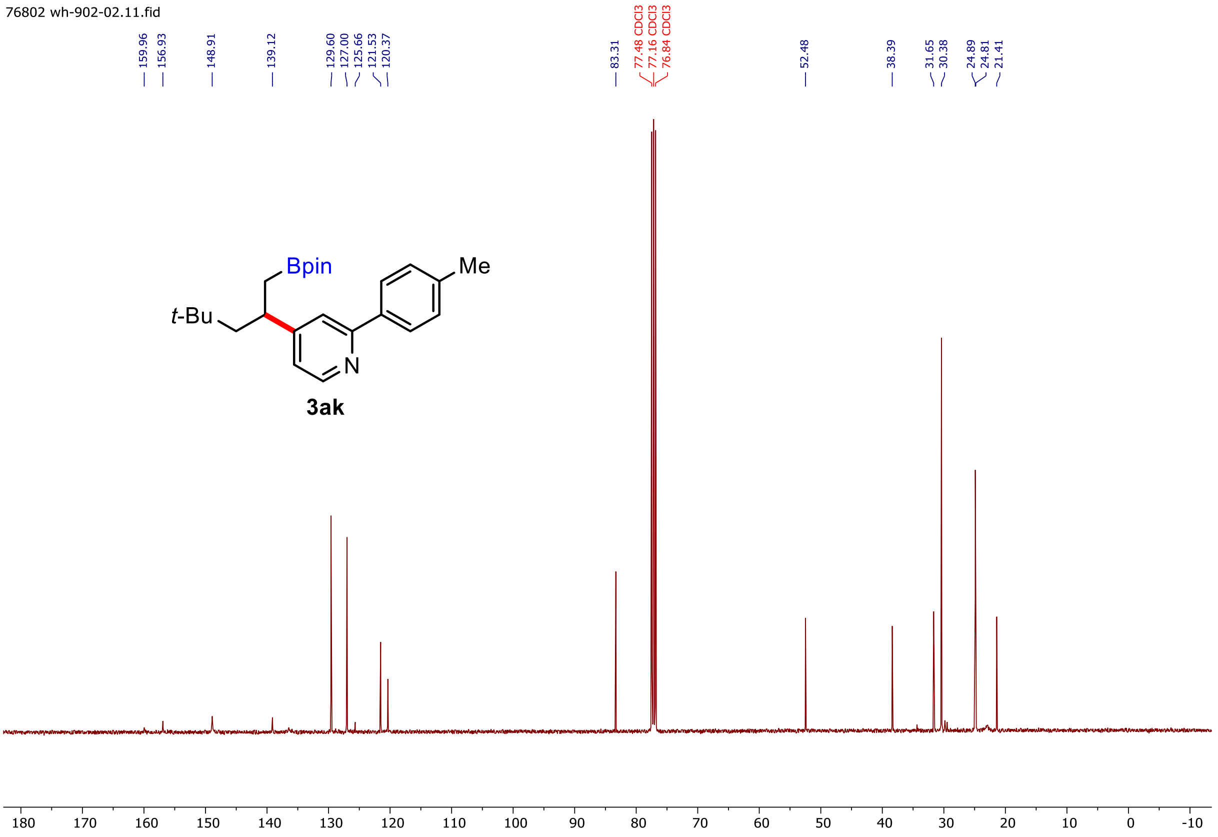

UOB Confidential & Sensitive

^{11}B NMR (128 MHz, $CDCl_3$) of **3ak**

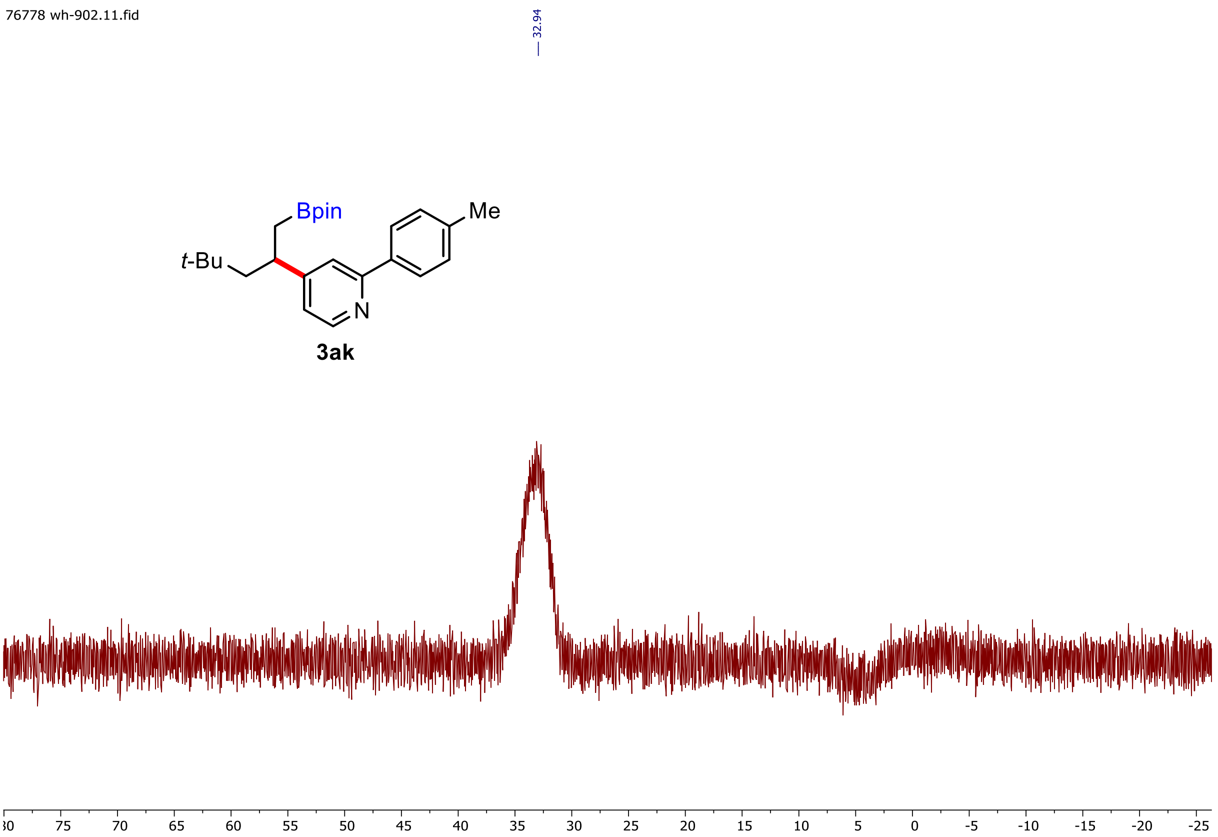

^{1}H NMR (400 MHz, $CDCl_3$) of **3al** (*see procedure*)

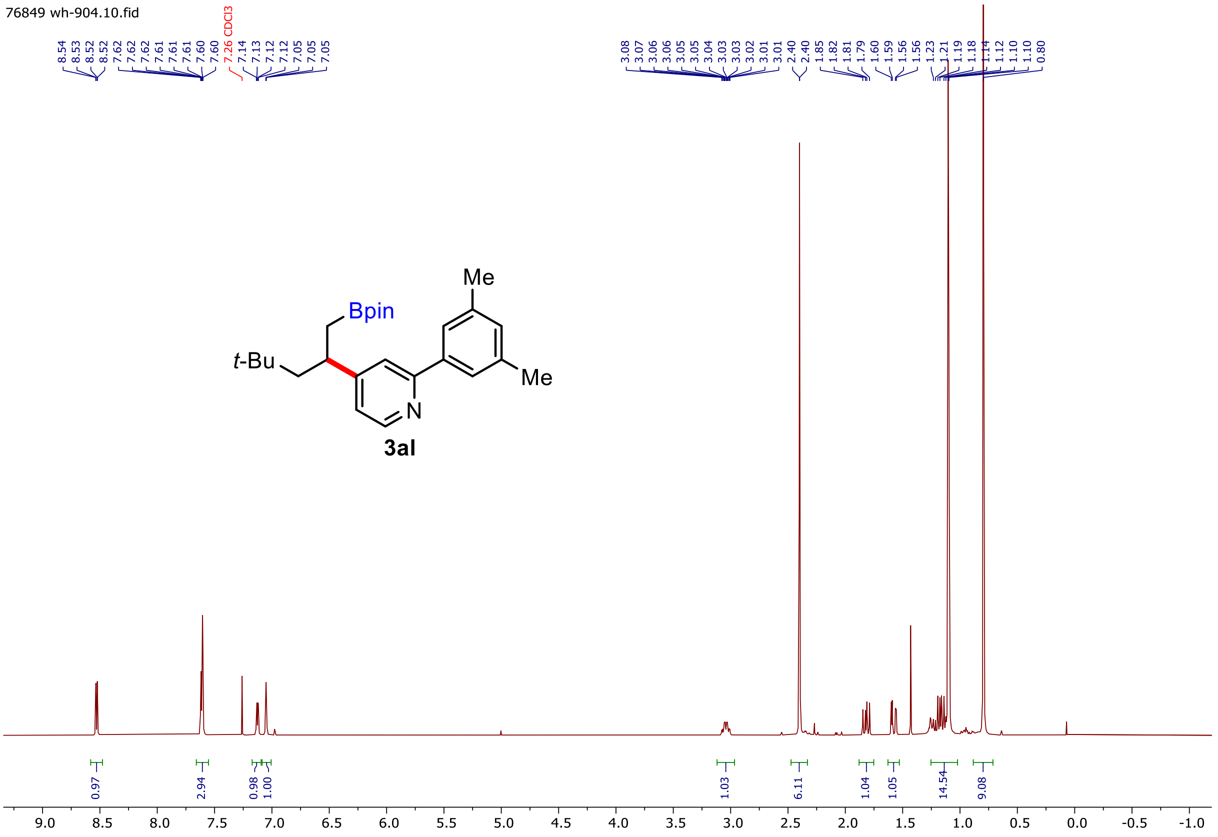

^{13}C NMR (101 MHz, $CDCl_3$) of **3al**

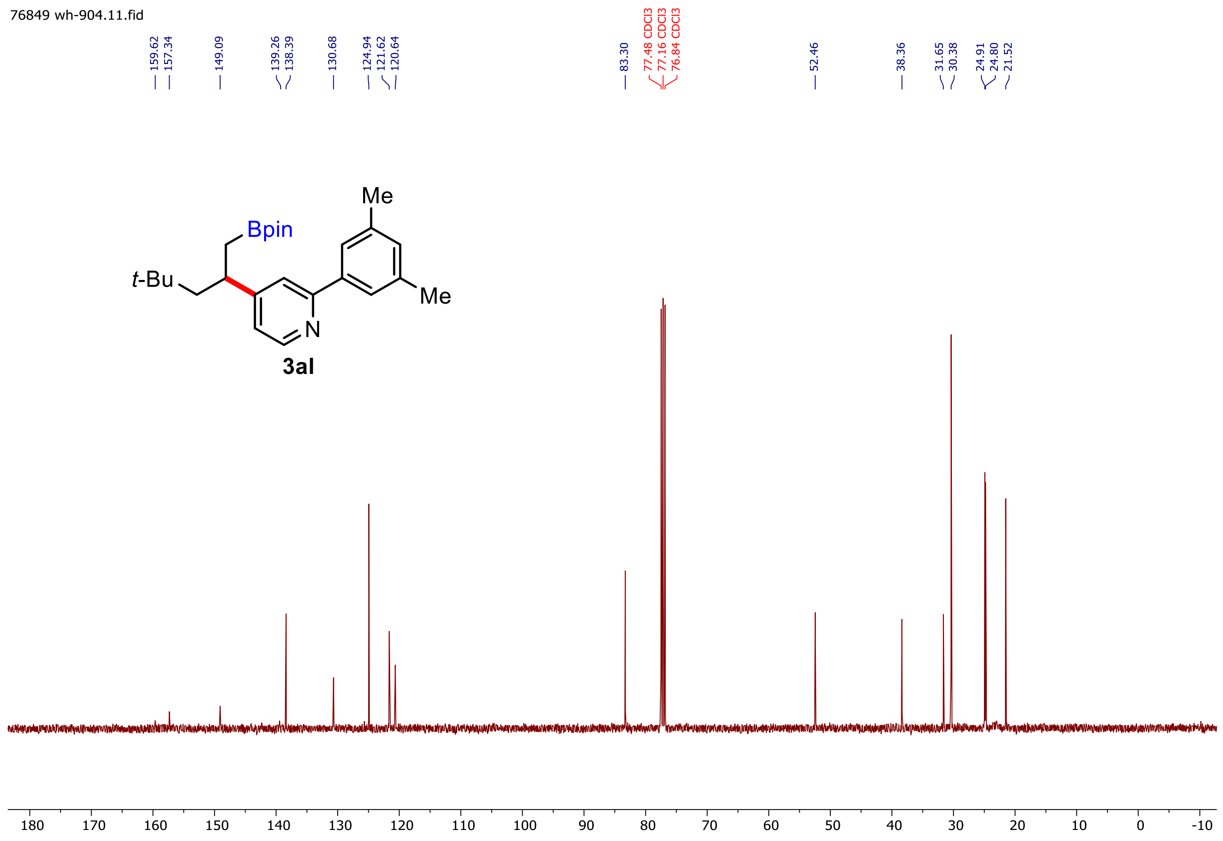

^{11}B NMR (128 MHz, $CDCl_3$) of **3al**

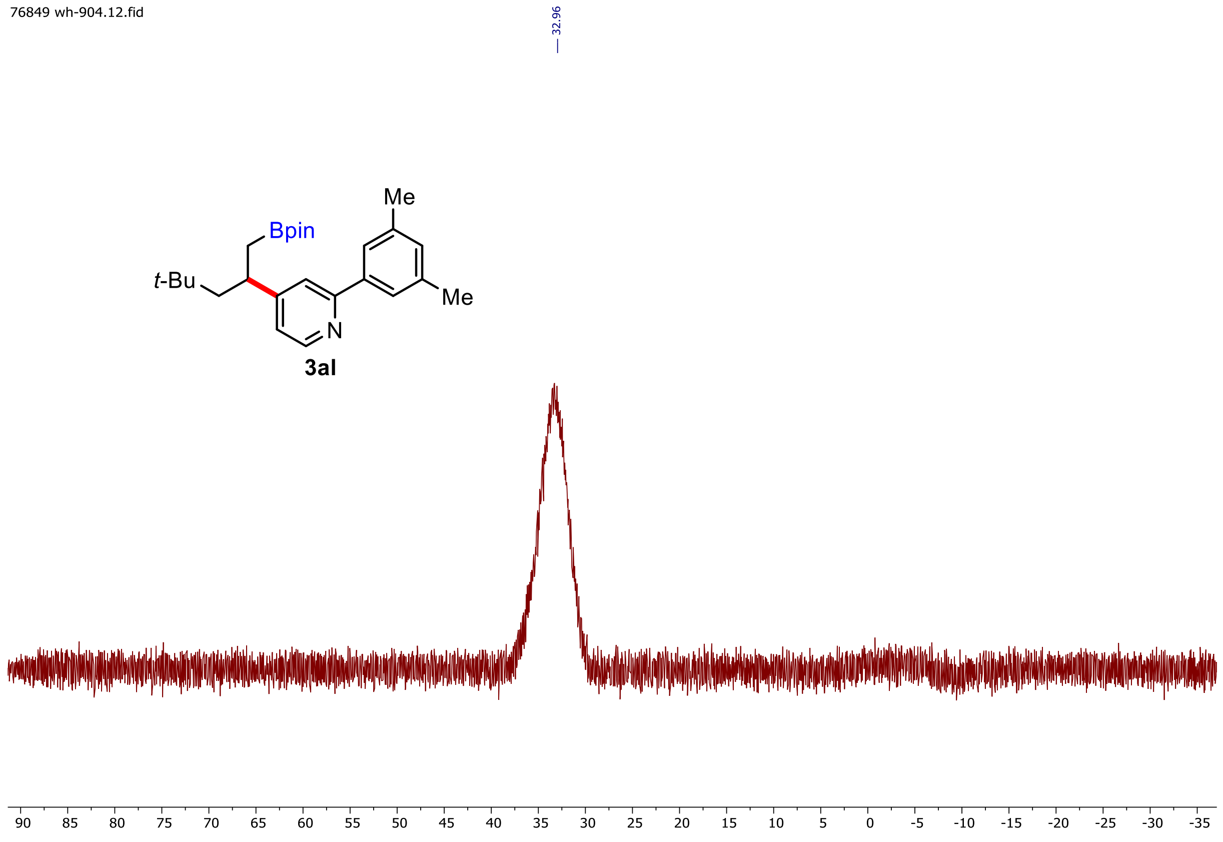

UOB Confidential & Sensitive

^{1}H NMR (400 MHz, $CDCl_3$) of **3am** (*see procedure*)

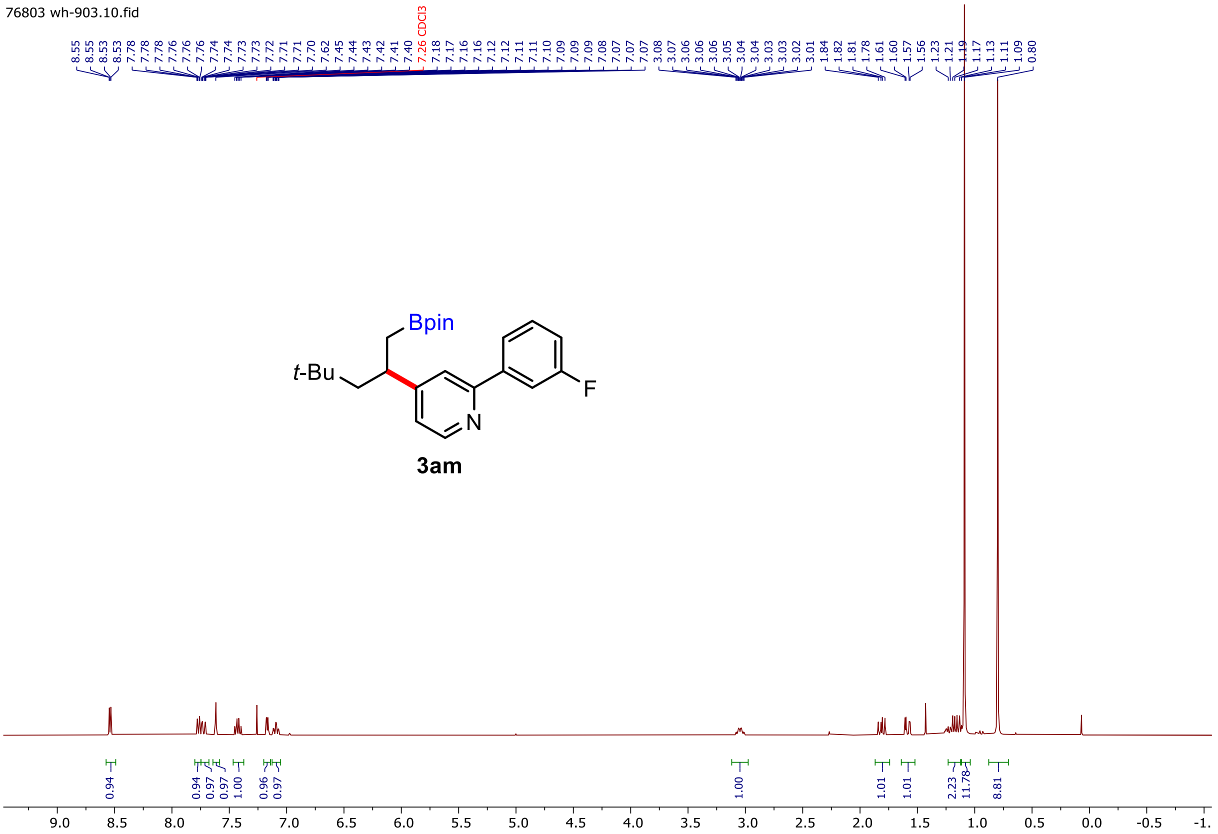

^{13}C NMR (101 MHz, $CDCl_3$) of **3am**

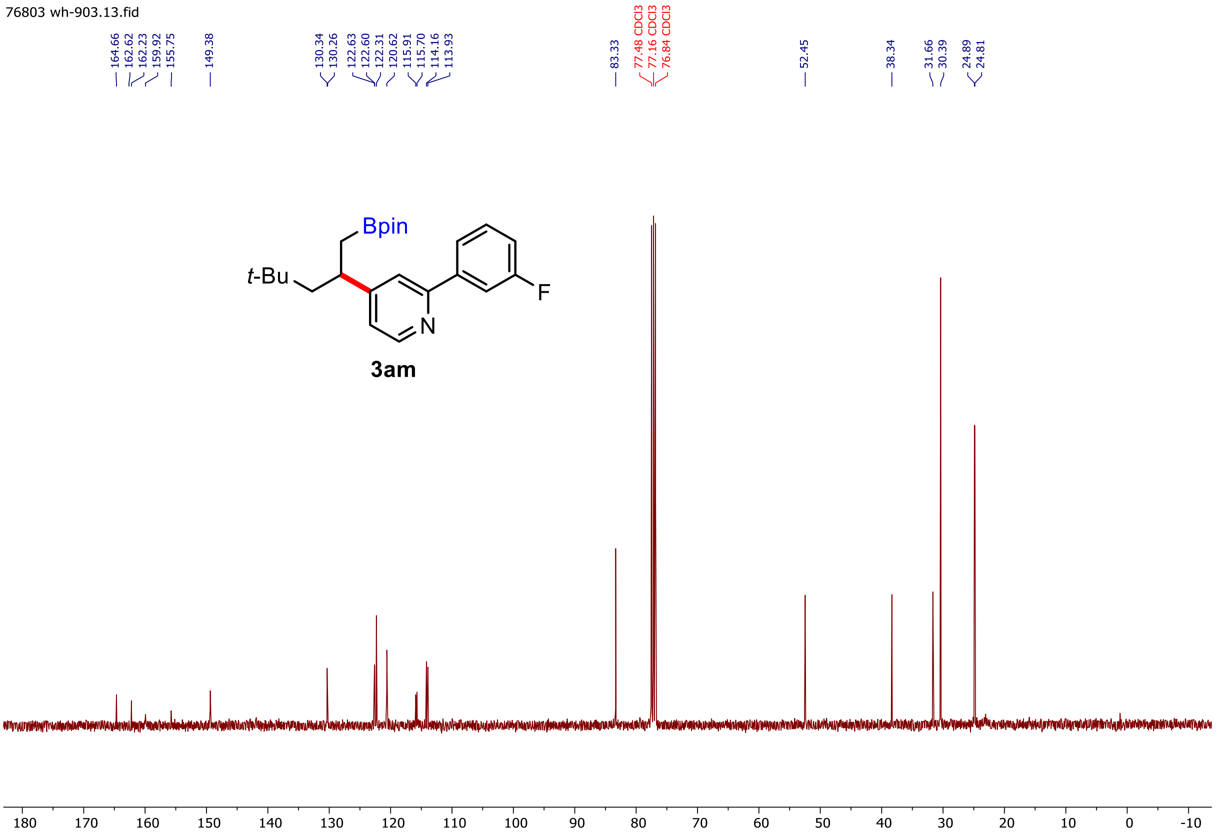

[19]C NMR (377 MHz, $CDCl_3$) of **3am**

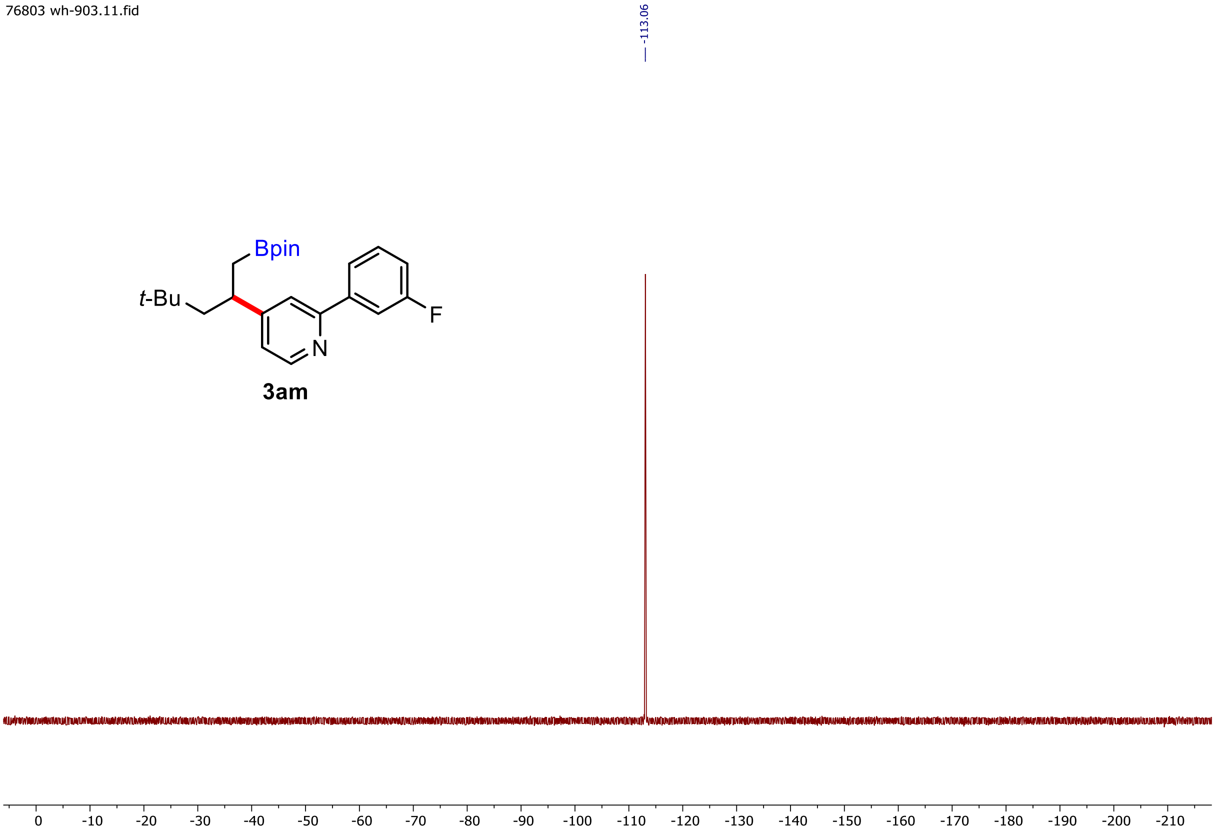

^{11}B NMR (128 MHz, $CDCl_3$) of **3am**

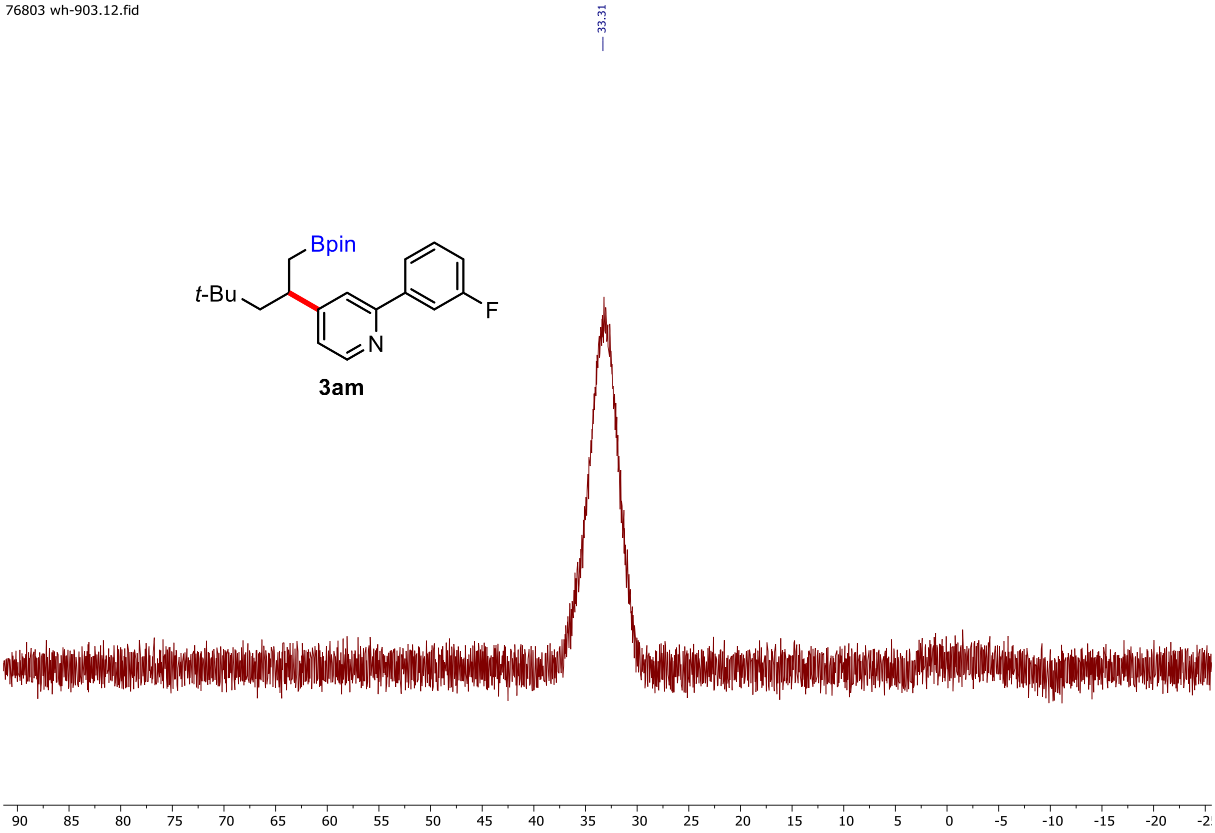

^{1}H NMR (400 MHz, $CDCl_3$) of **6aa** (see procedure)

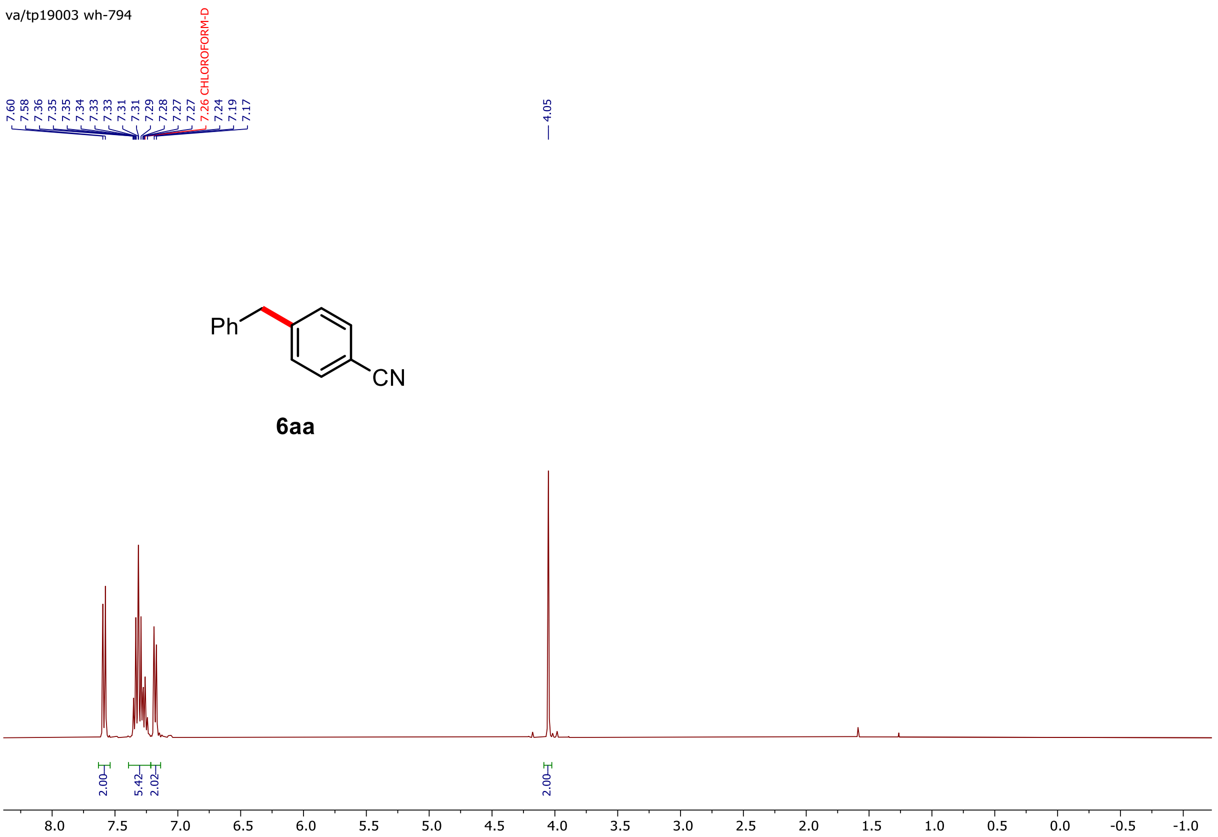

^{13}C NMR (101 MHz, $CDCl_3$) of **6aa**

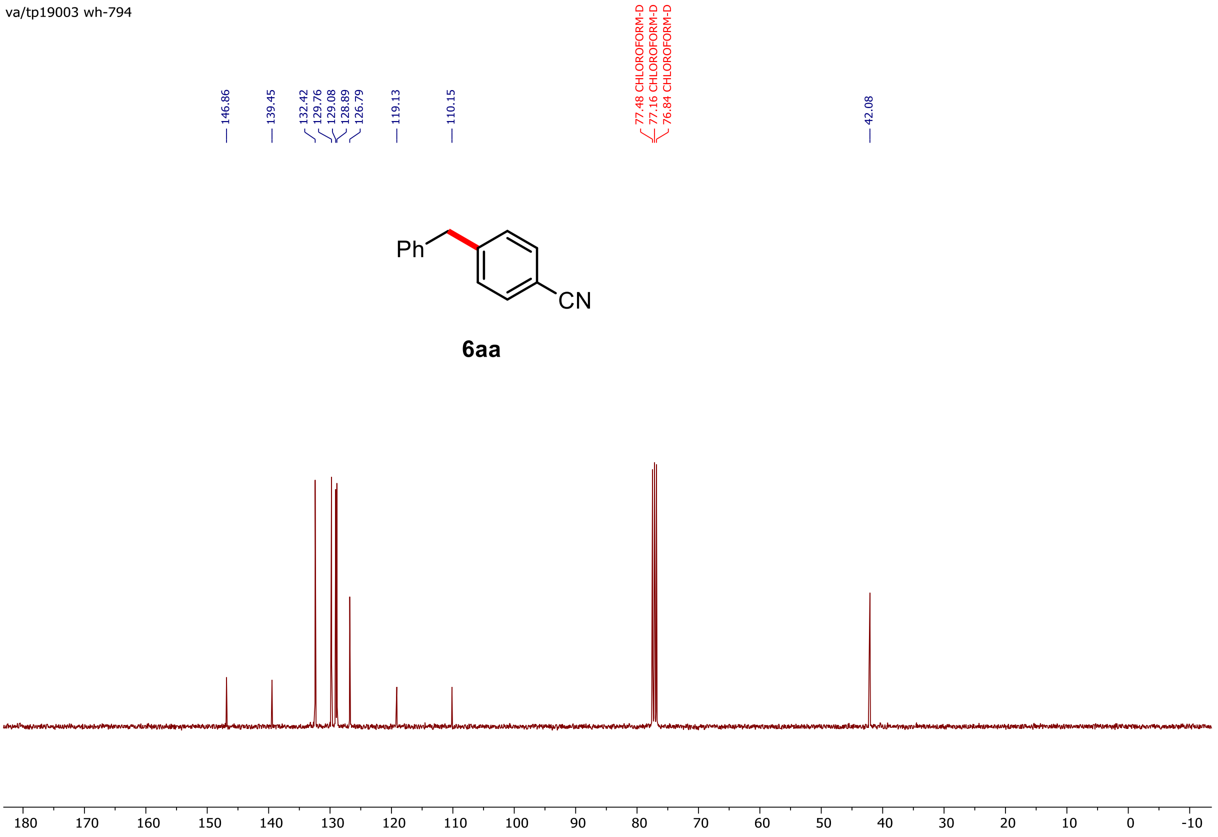

UOB Confidential & Sensitive

^{1}H NMR (400 MHz, $CDCl_3$) of **6ba** (see procedure)

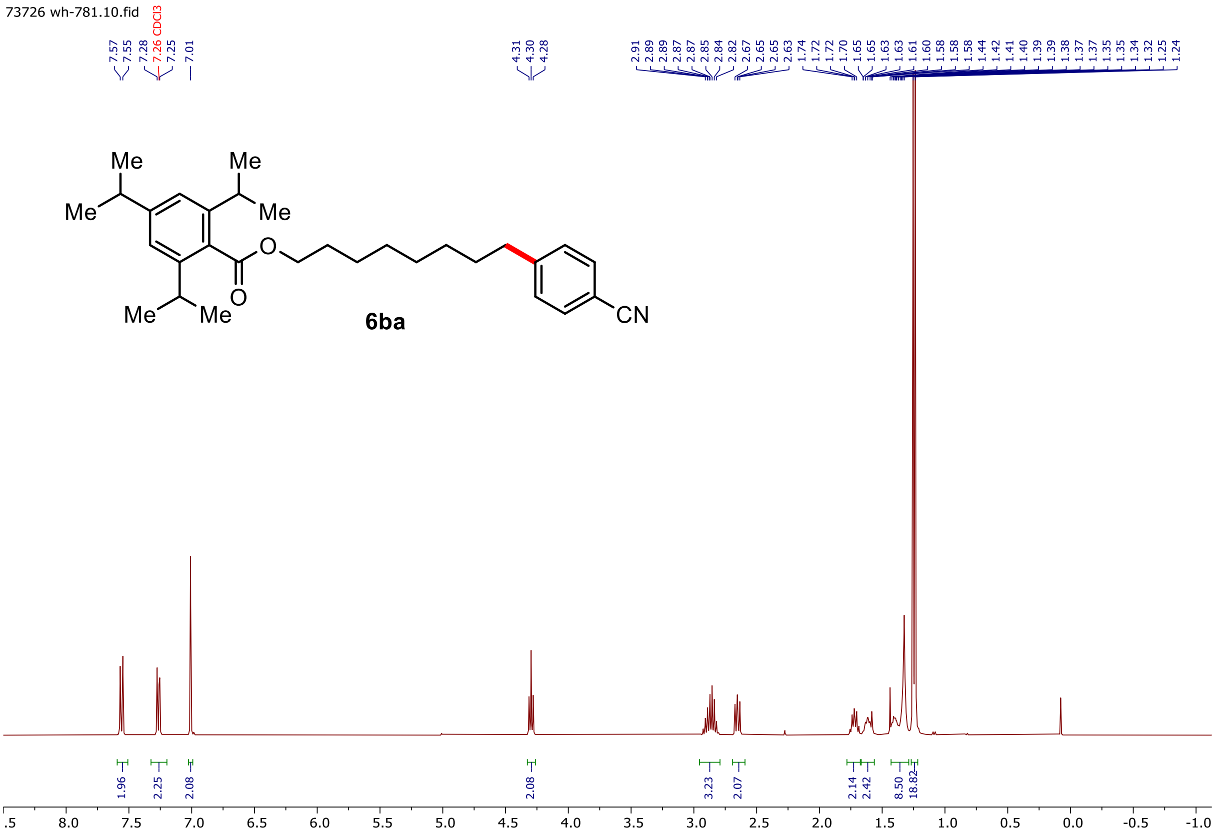

^{13}C NMR (101 MHz, $CDCl_3$) of **6ba**

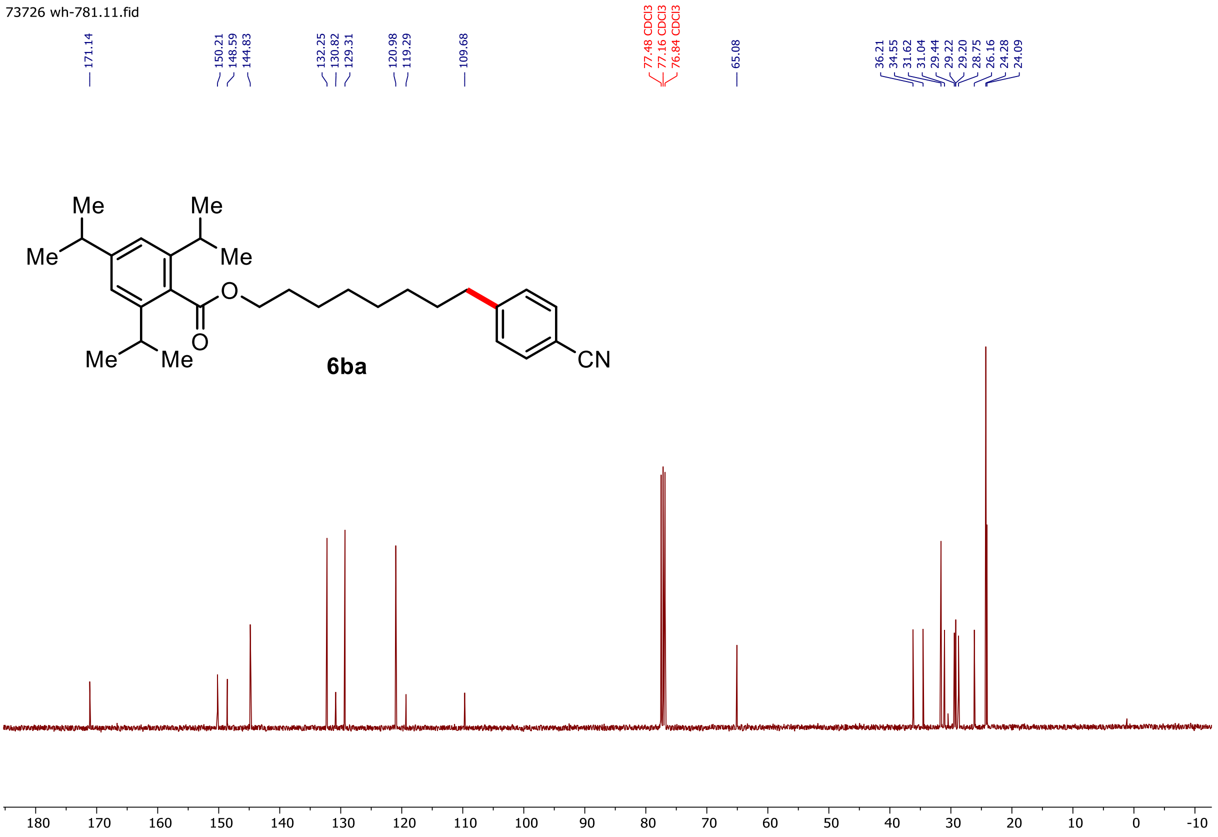

^{1}H NMR (400 MHz, $CDCl_3$) of **6ca** (*see procedure*)

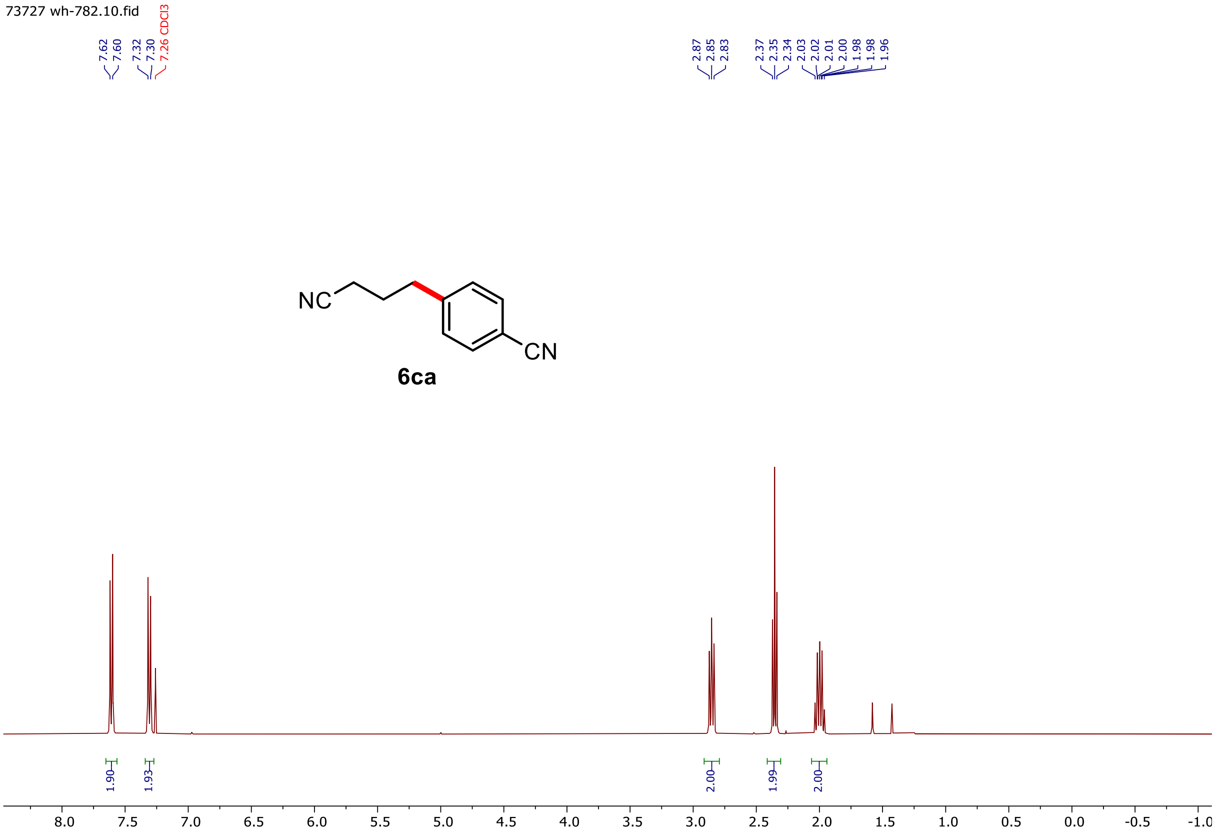

^{13}C NMR (101 MHz, $CDCl_3$) of **6ca**

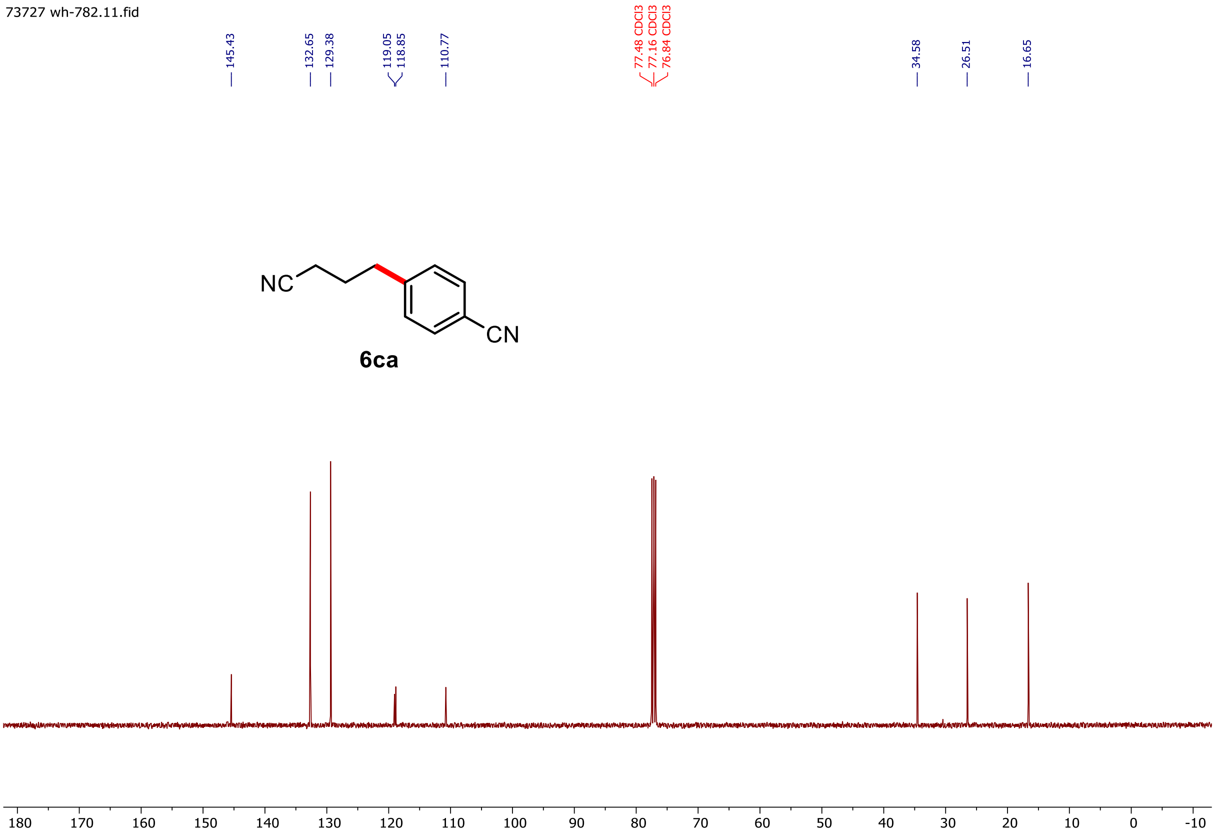

UOB Confidential & Sensitive

^{1}H NMR (400 MHz, $CDCl_3$) of **6da** (*see procedure*)

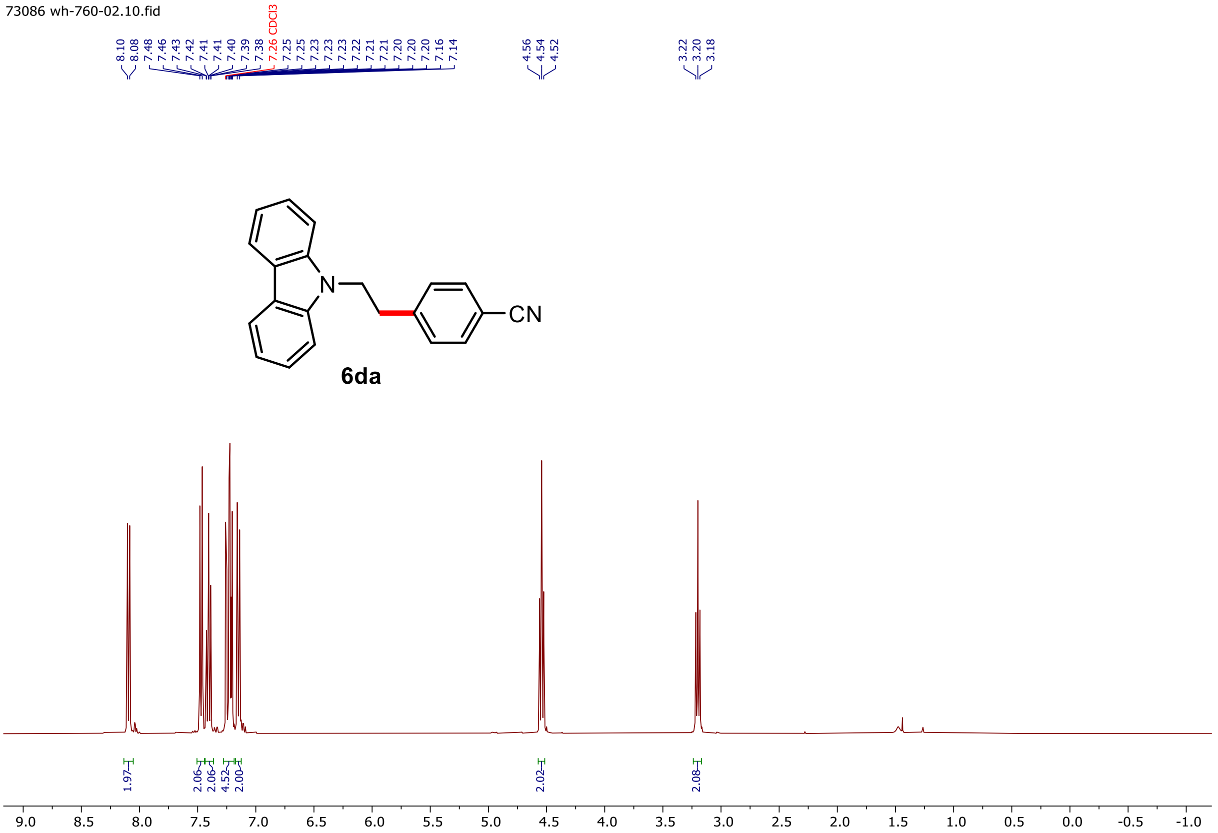

^{13}C NMR (101 MHz, $CDCl_3$) of **6da**

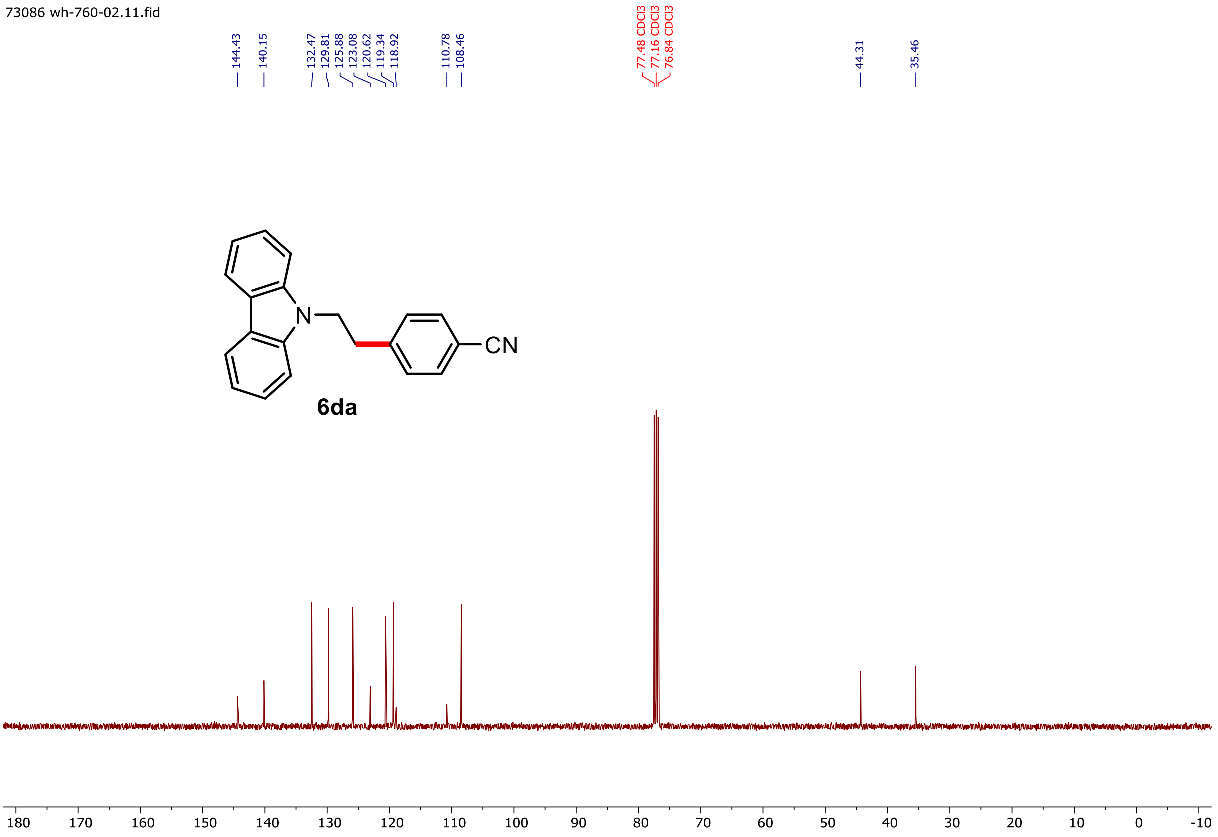

^{1}H NMR (400 MHz, $CDCl_3$) of **6ea** (*see procedure*)

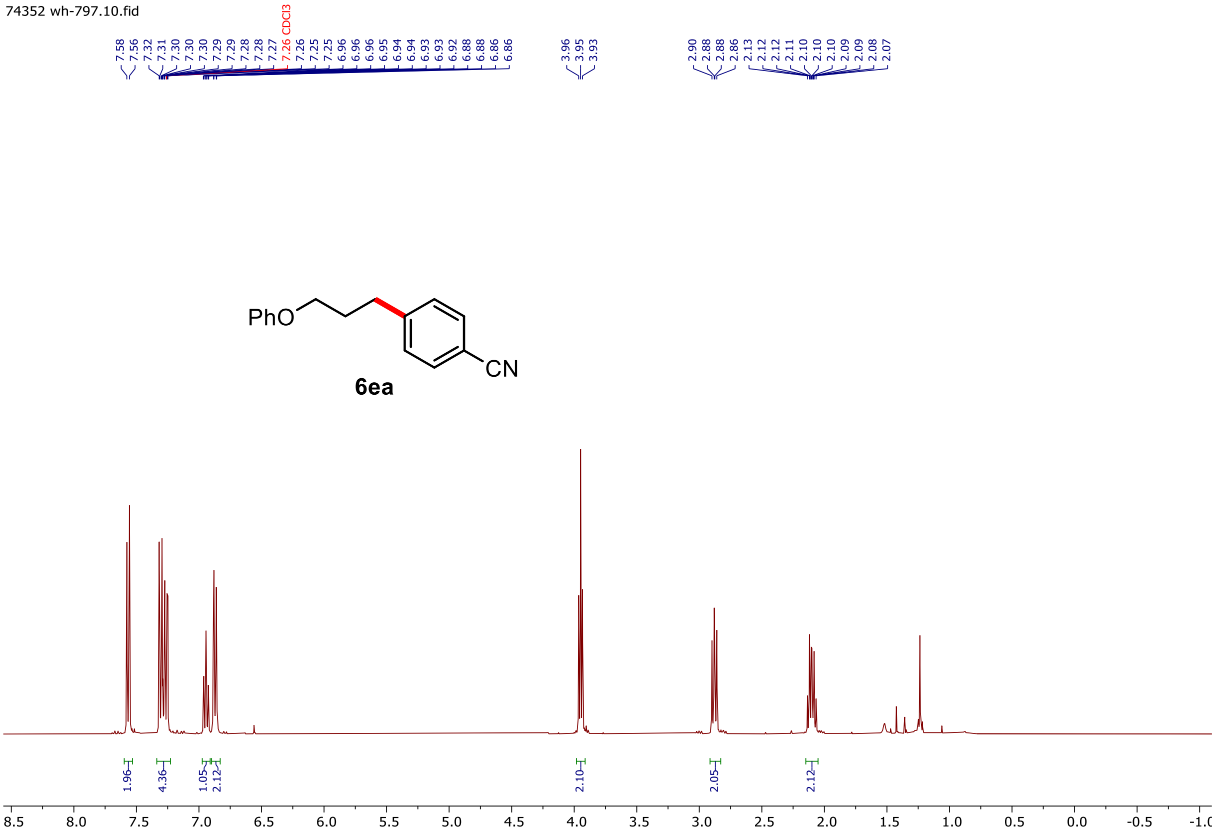

^{13}C NMR (101 MHz, $CDCl_3$) of **6ea**

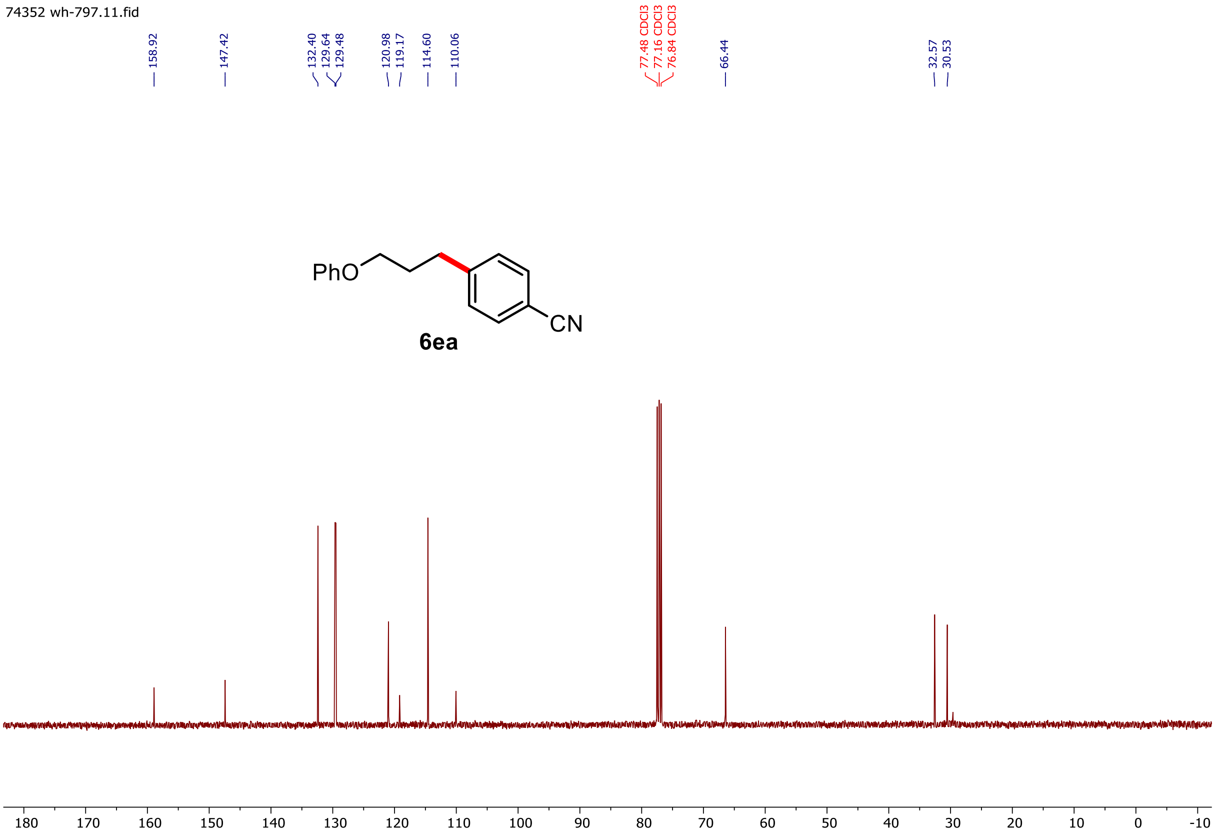

^{1}H NMR (400 MHz, $CDCl_3$) of **6fa** (*see procedure*)

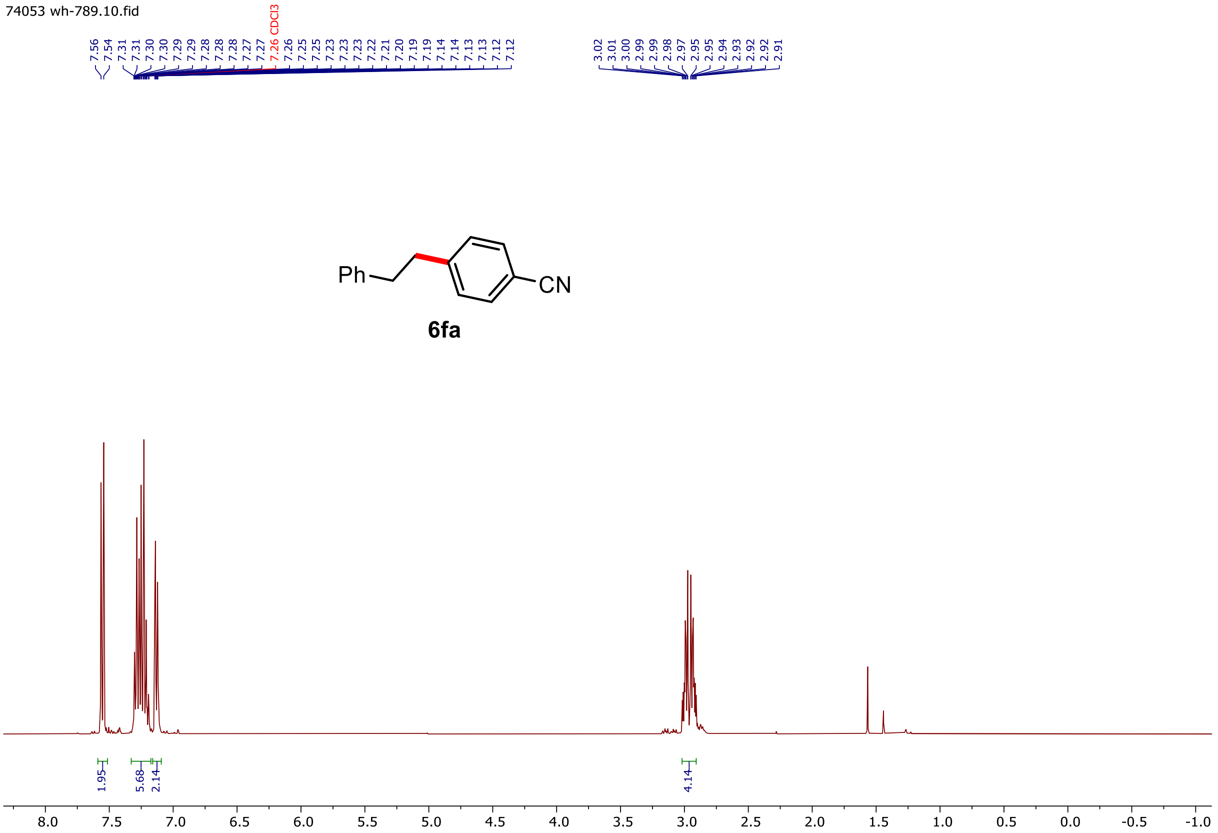

^{13}C NMR (101 MHz, $CDCl_3$) of **6fa**

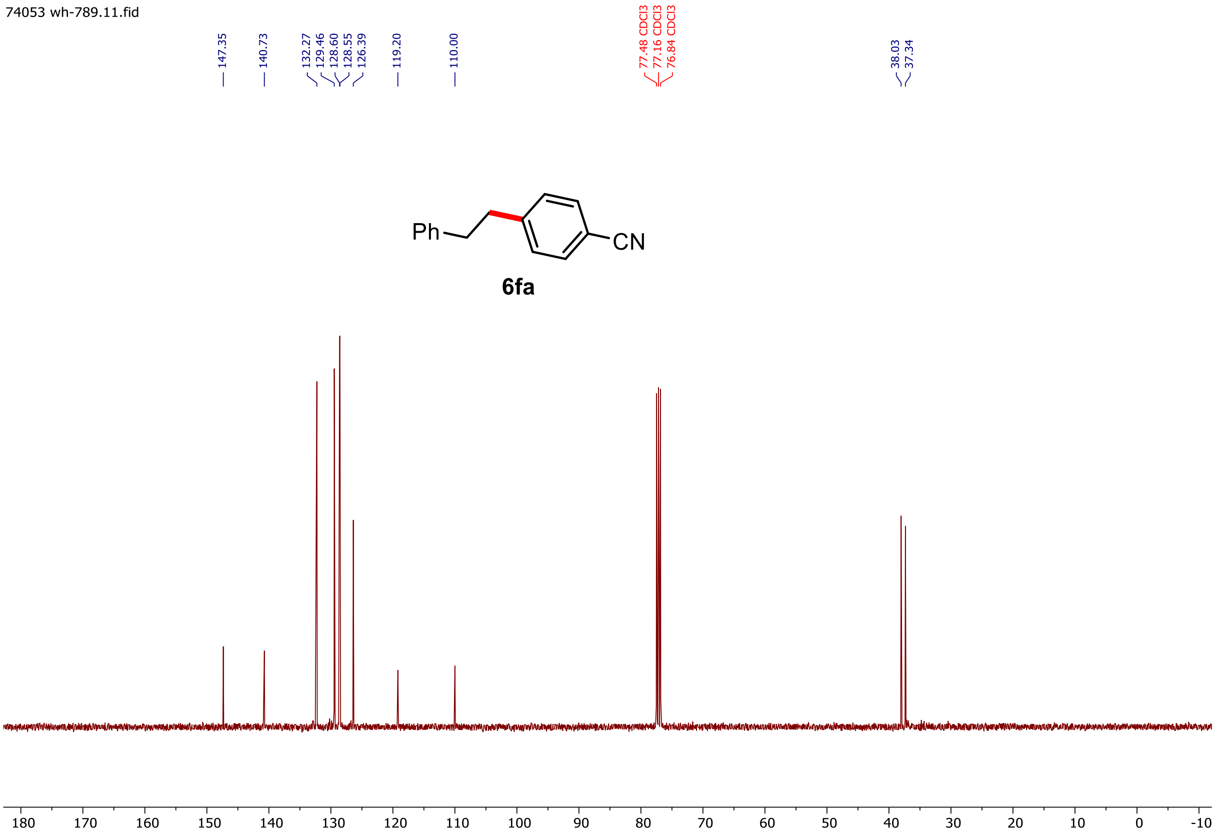

^{1}H NMR (400 MHz, $CDCl_3$) of **6ga** (*see procedure*)

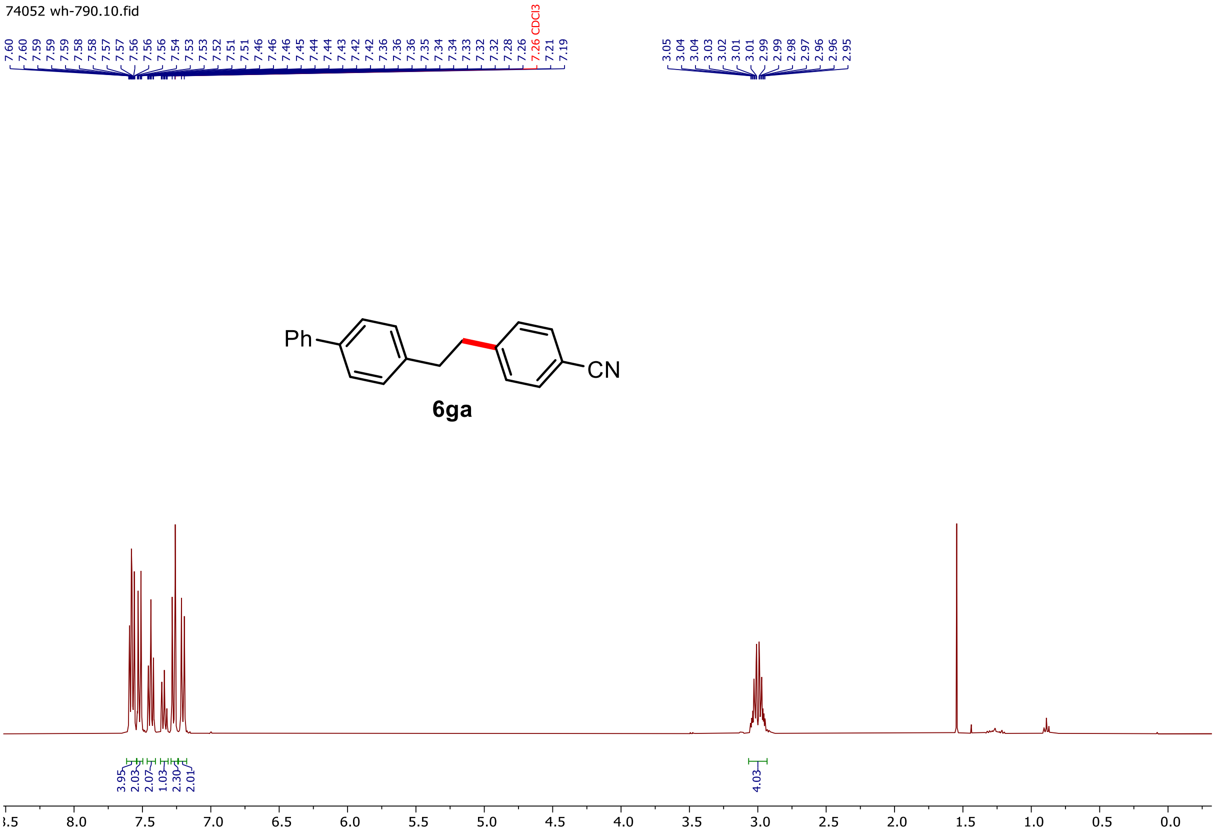

^{13}C NMR (101 MHz, $CDCl_3$) of **6ga**

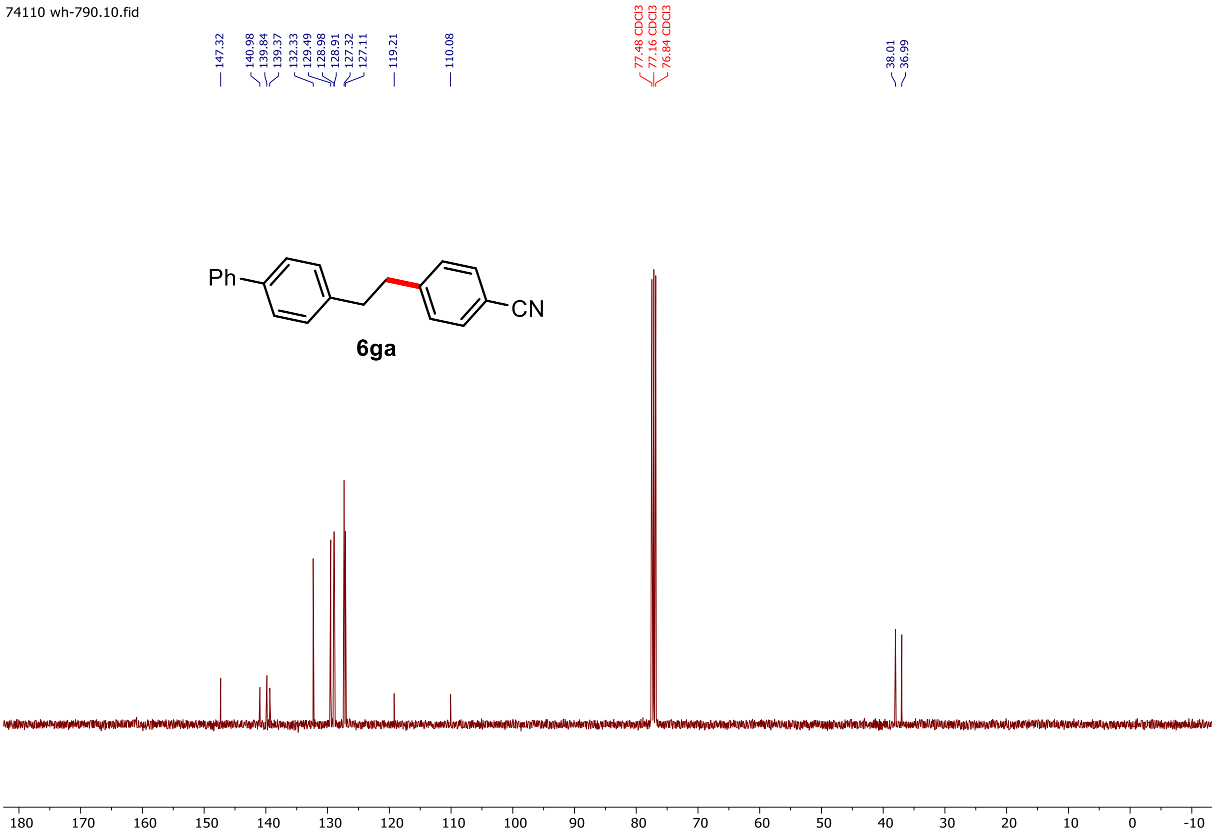

^{1}H NMR (400 MHz, $CDCl_3$) of **6ha** (*see procedure*)

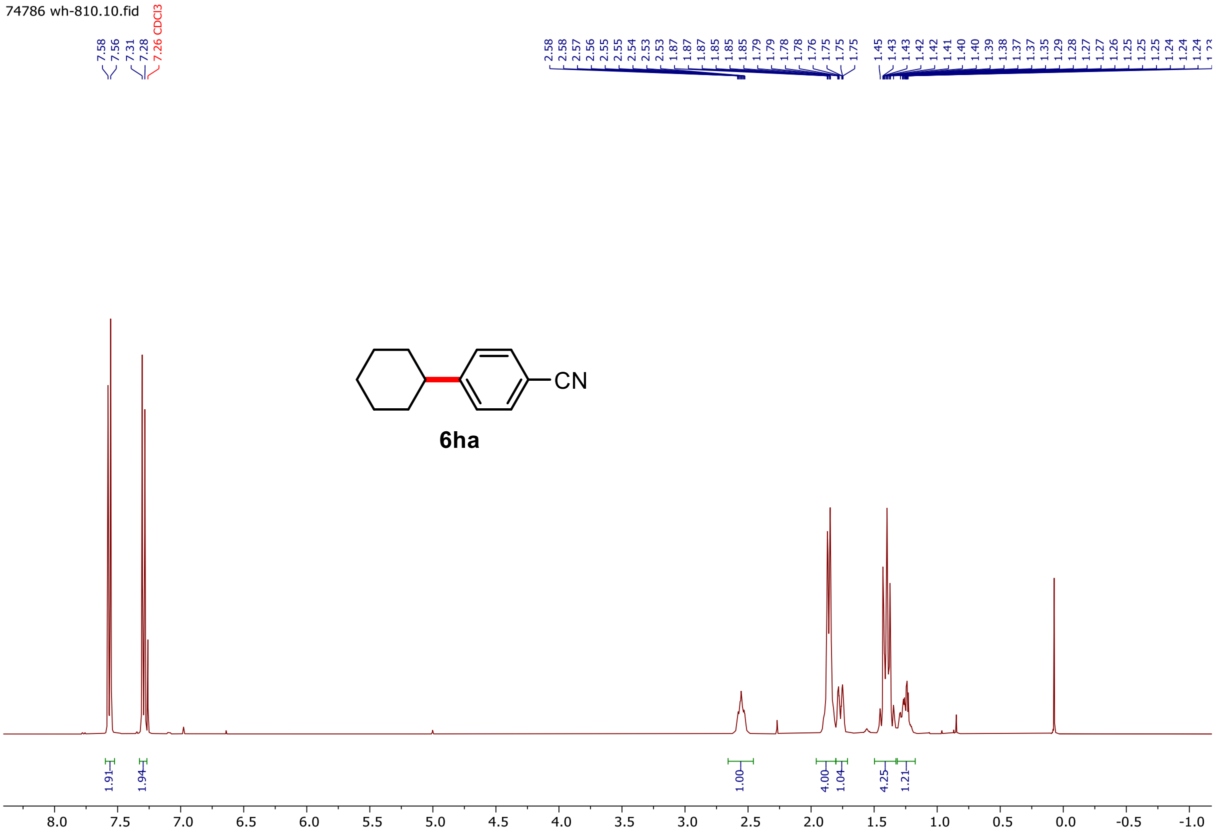

^{13}C NMR (101 MHz, $CDCl_3$) of **6ha**

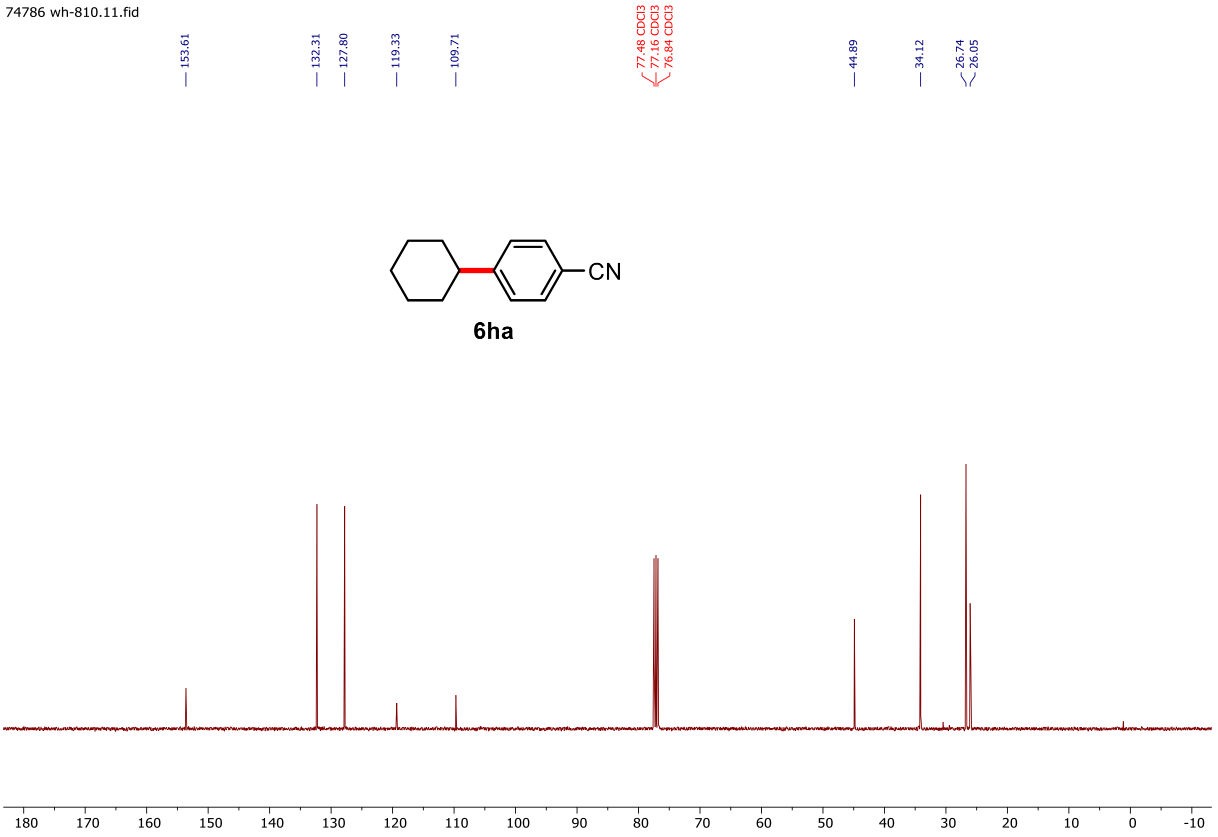

^{1}H NMR (400 MHz, $CDCl_3$) of **6ia** (*see procedure*)

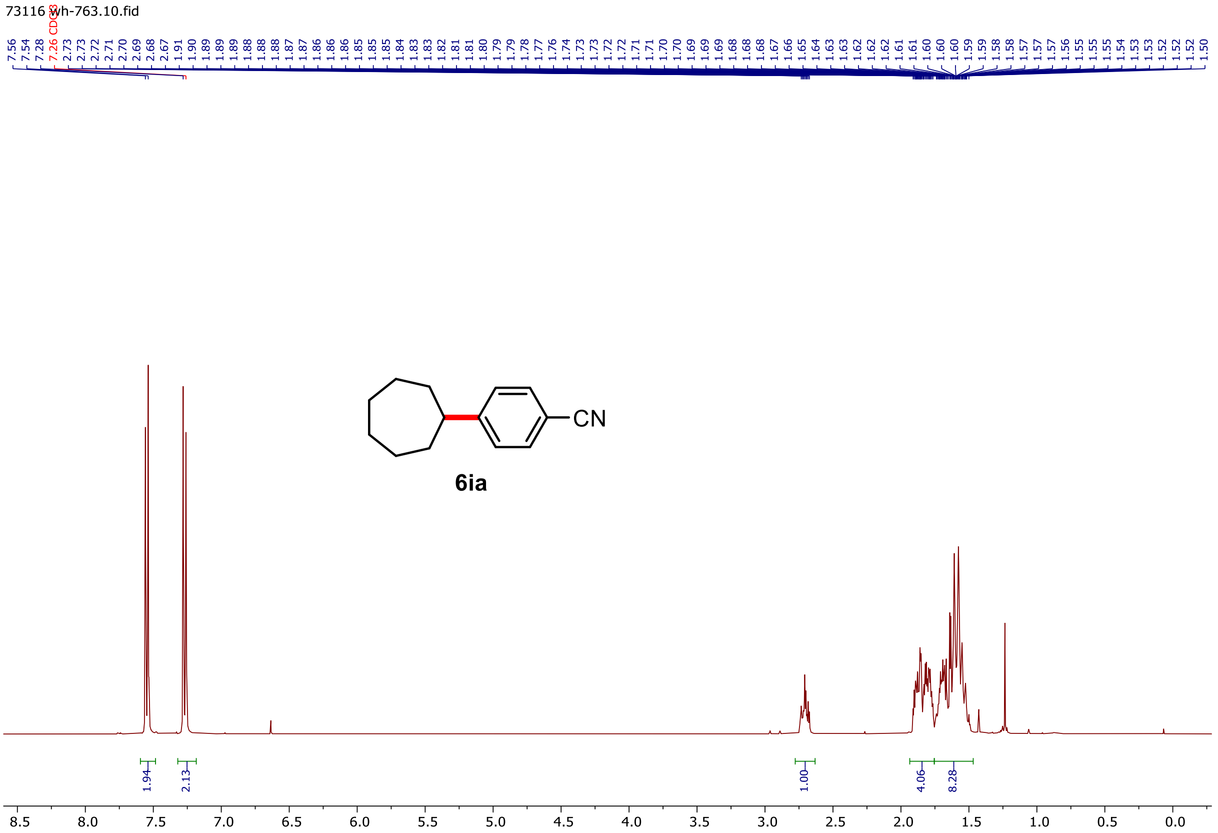

^{13}C NMR (101 MHz, $CDCl_3$) of **6ia**

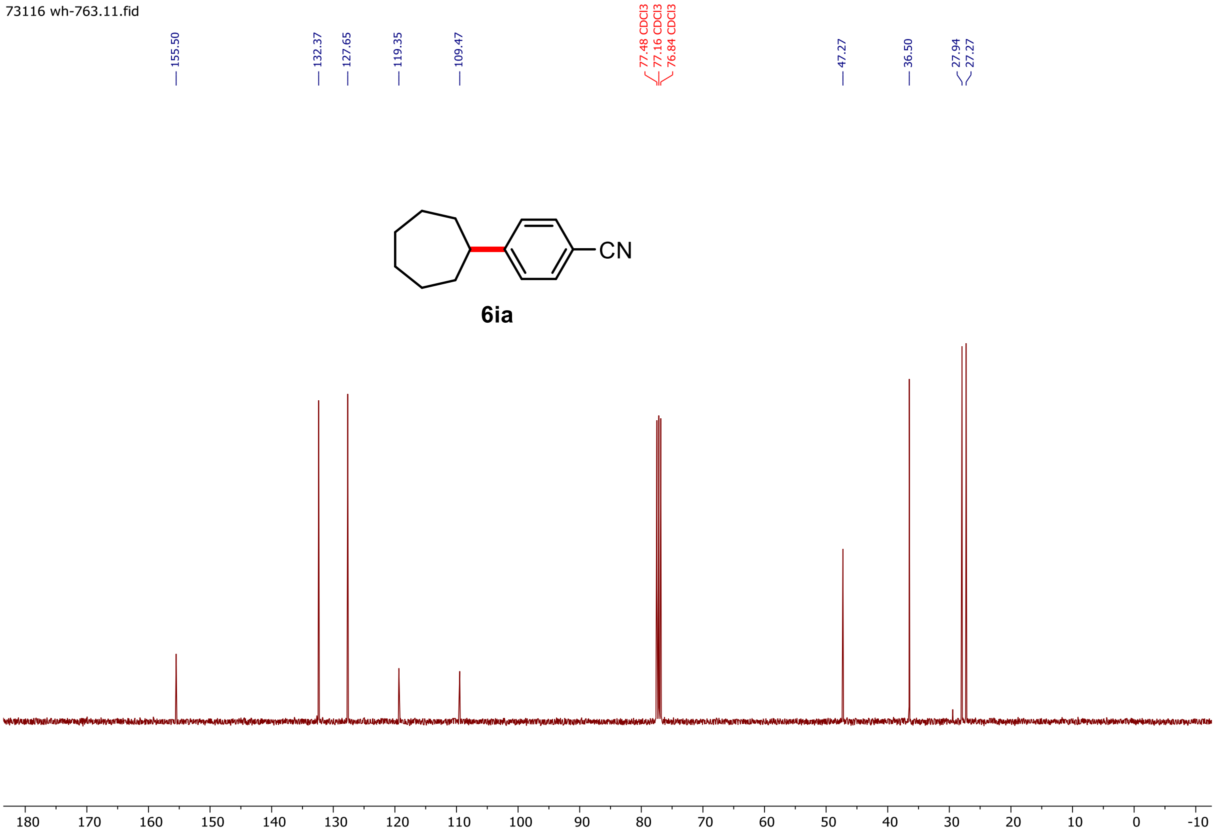

^{1}H NMR (400 MHz, $CDCl_3$) of **6ja** (*see procedure*)

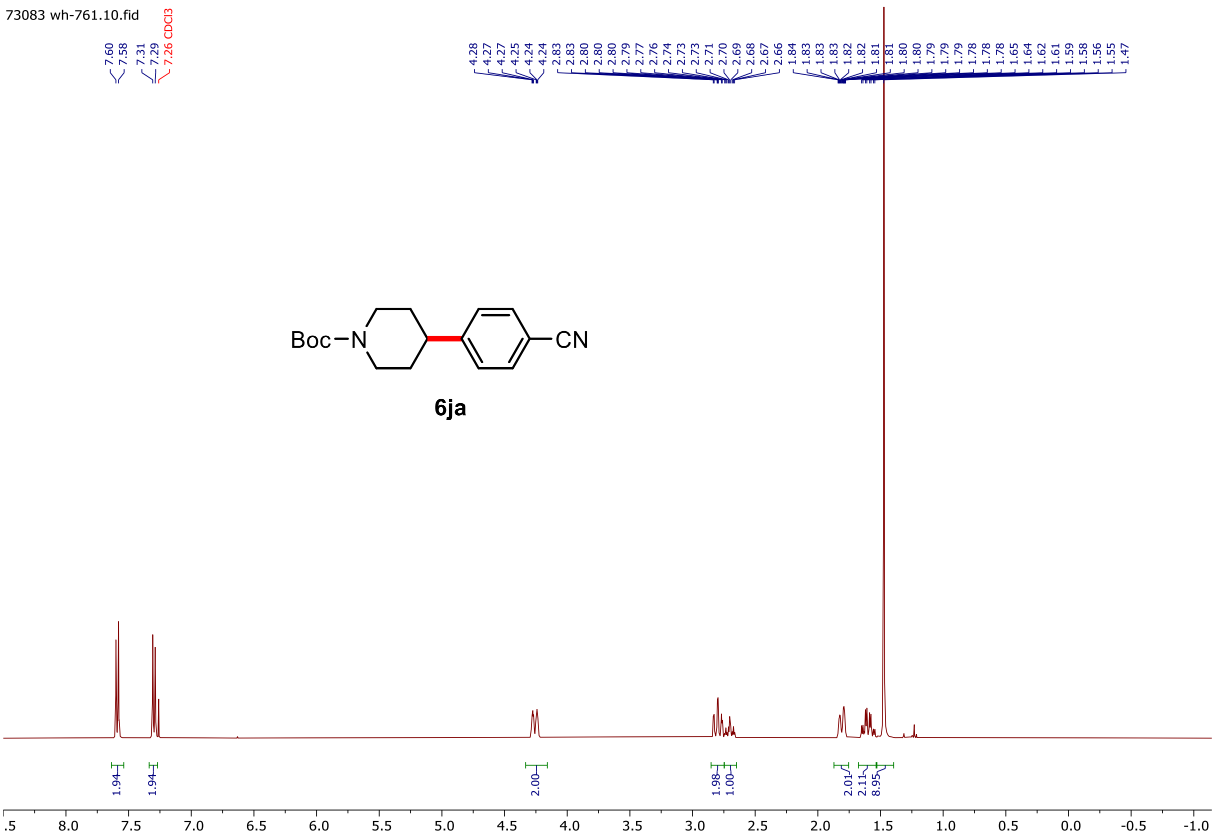

^{13}C NMR (101 MHz, $CDCl_3$) of **6ja**

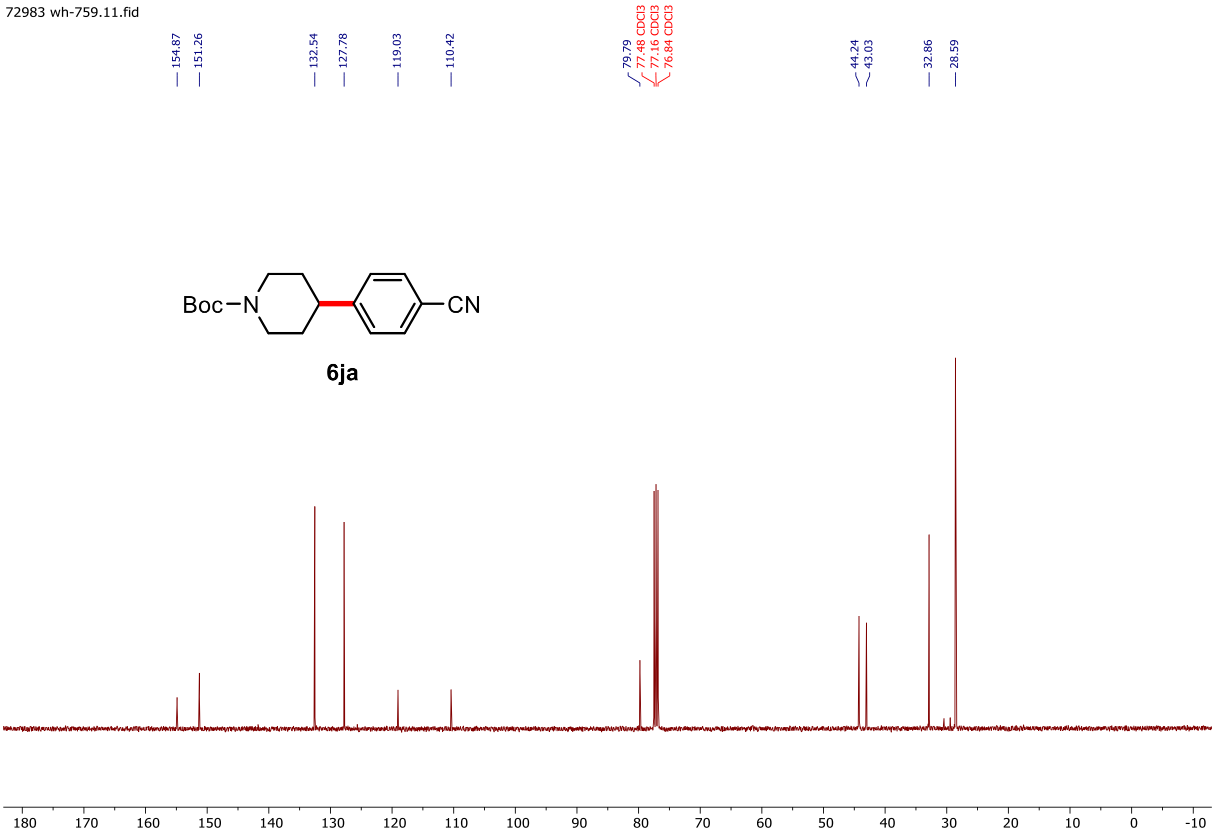

^{1}H NMR (400 MHz, $CDCl_3$) of **6ka** (*see procedure*)

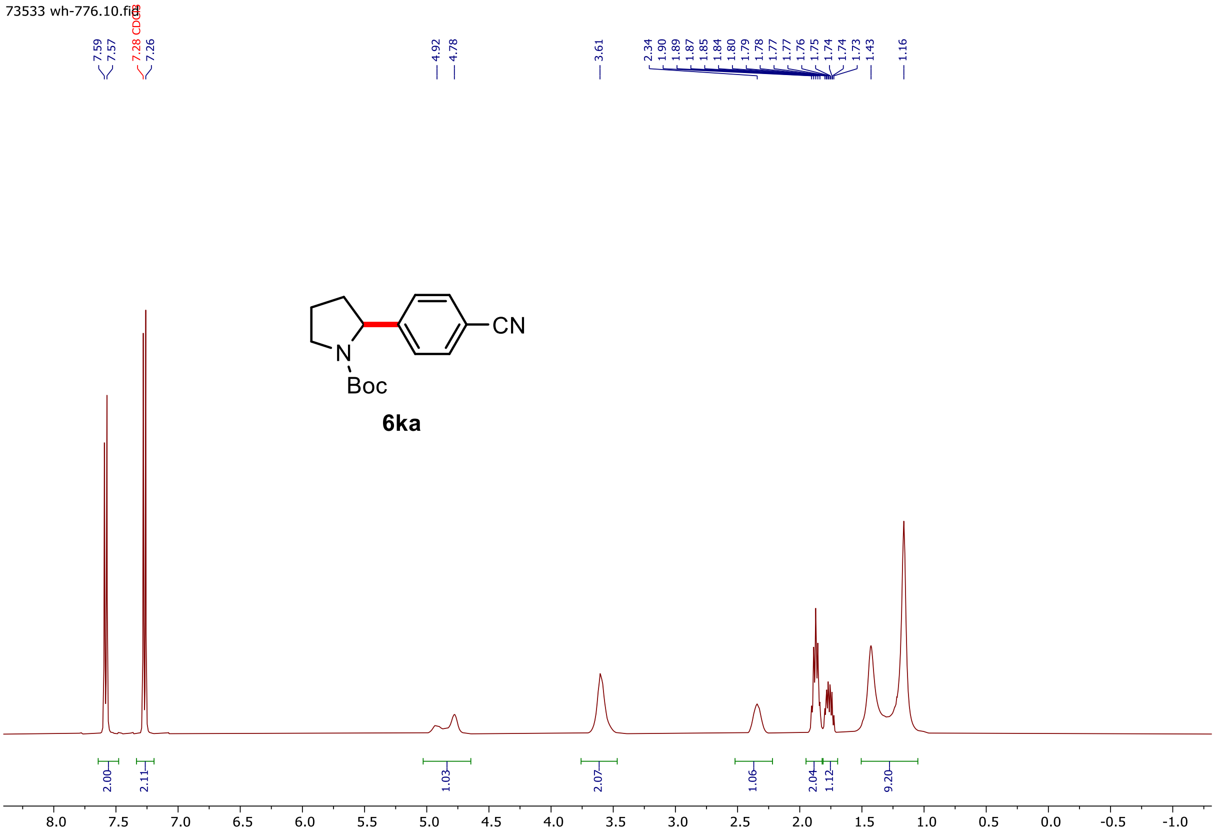

^{13}C NMR (101 MHz, $CDCl_3$) of **6ka**

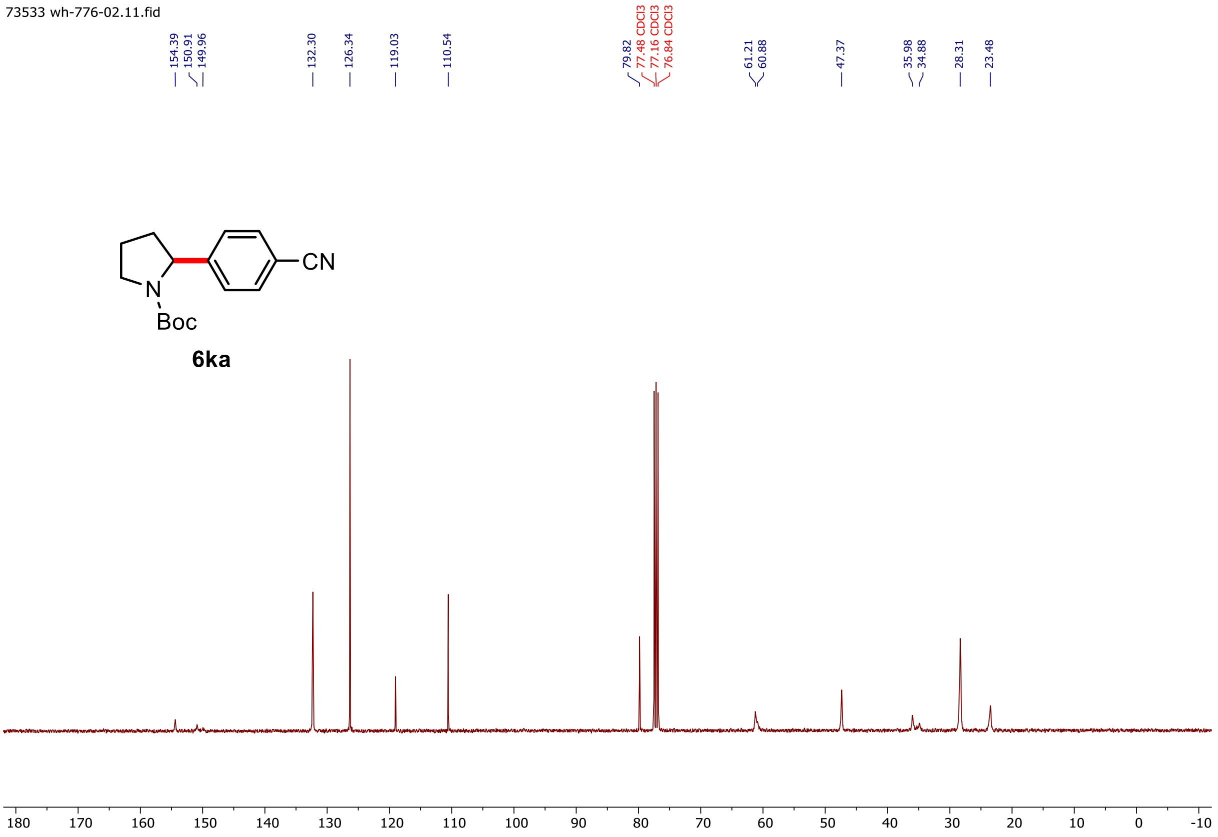

^{1}H NMR (400 MHz, $CDCl_3$) of **6la** (*see procedure*)

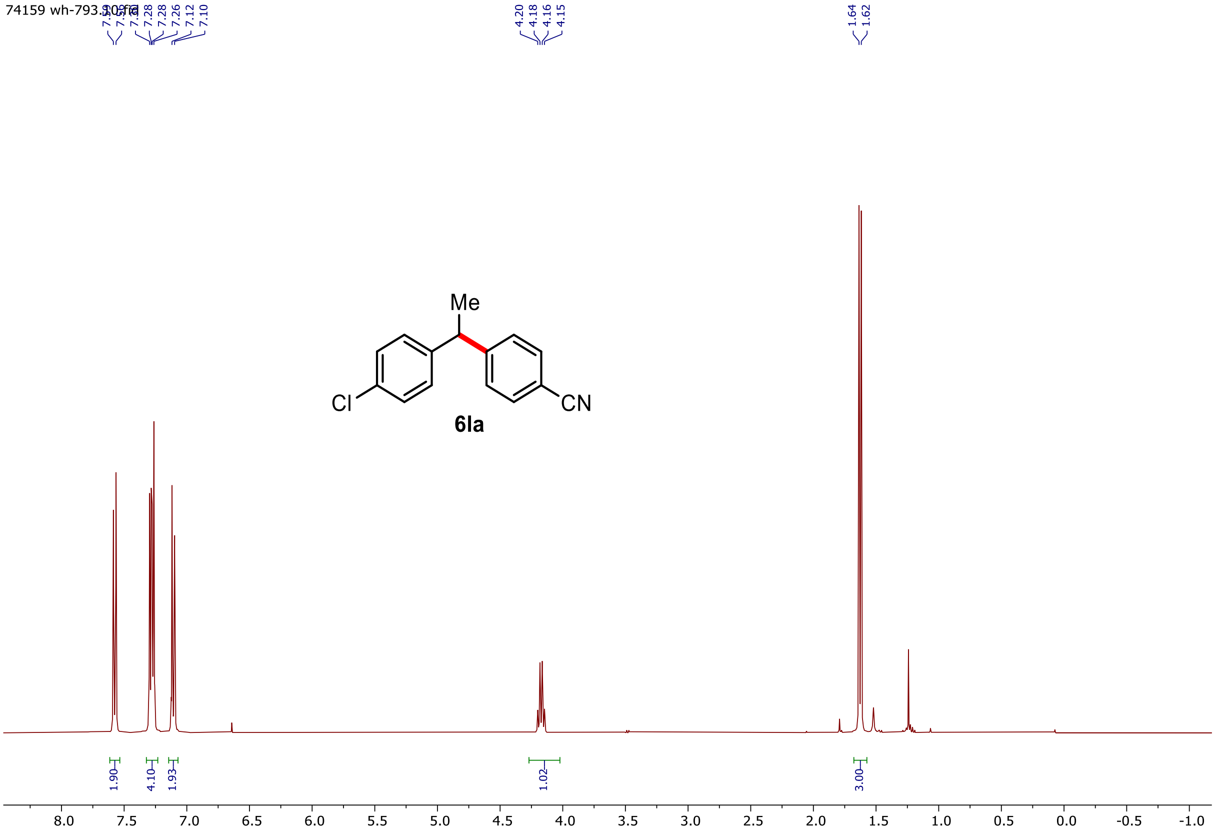

^{13}C NMR (101 MHz, $CDCl_3$) of **6la**

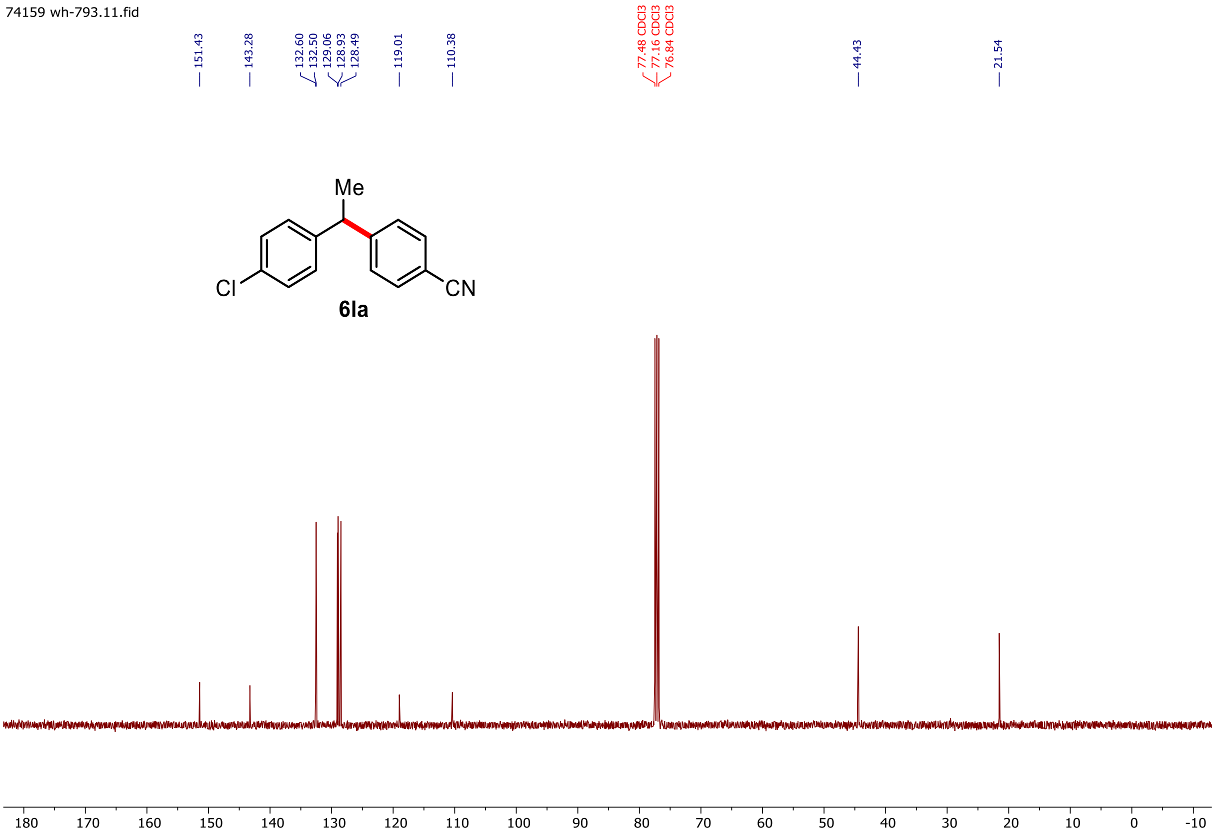

^{1}H NMR (400 MHz, $CDCl_3$) of **6ma** (*see procedure*)

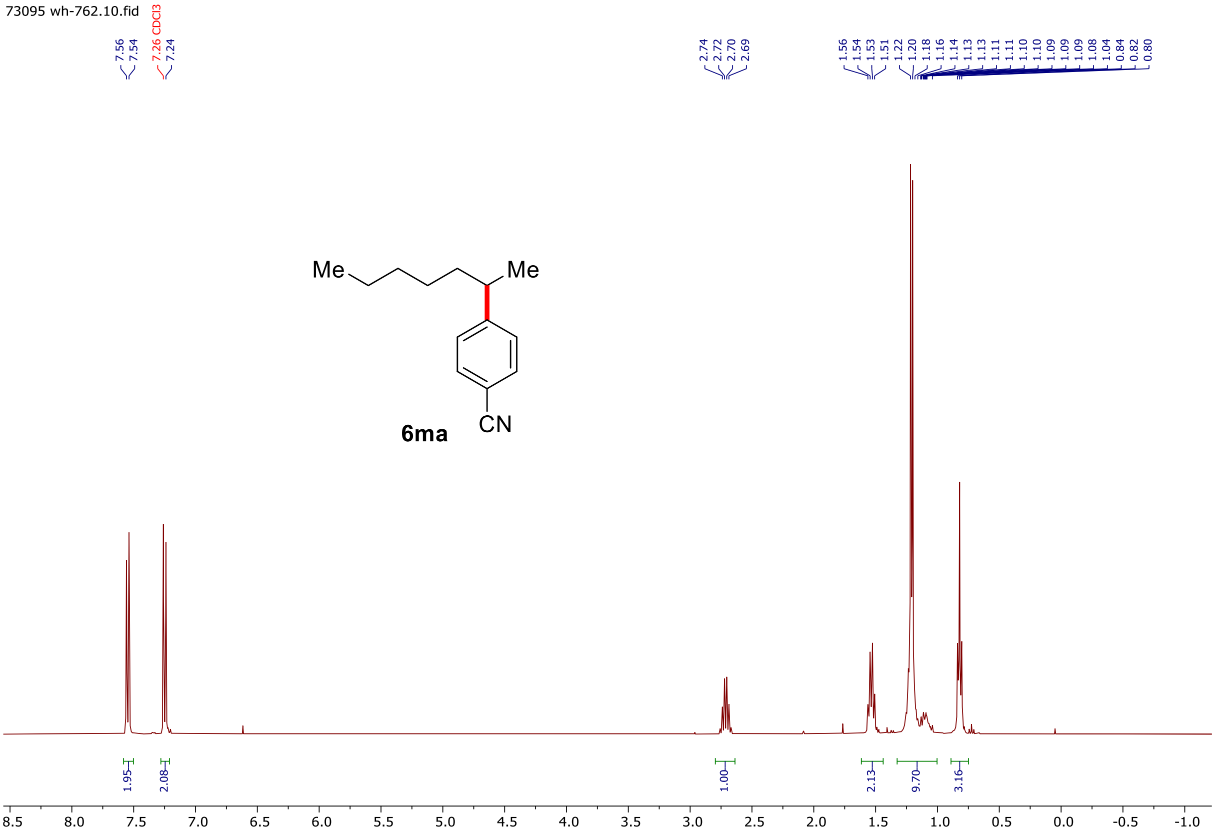

^{13}C NMR (101 MHz, $CDCl_3$) of **6ma**

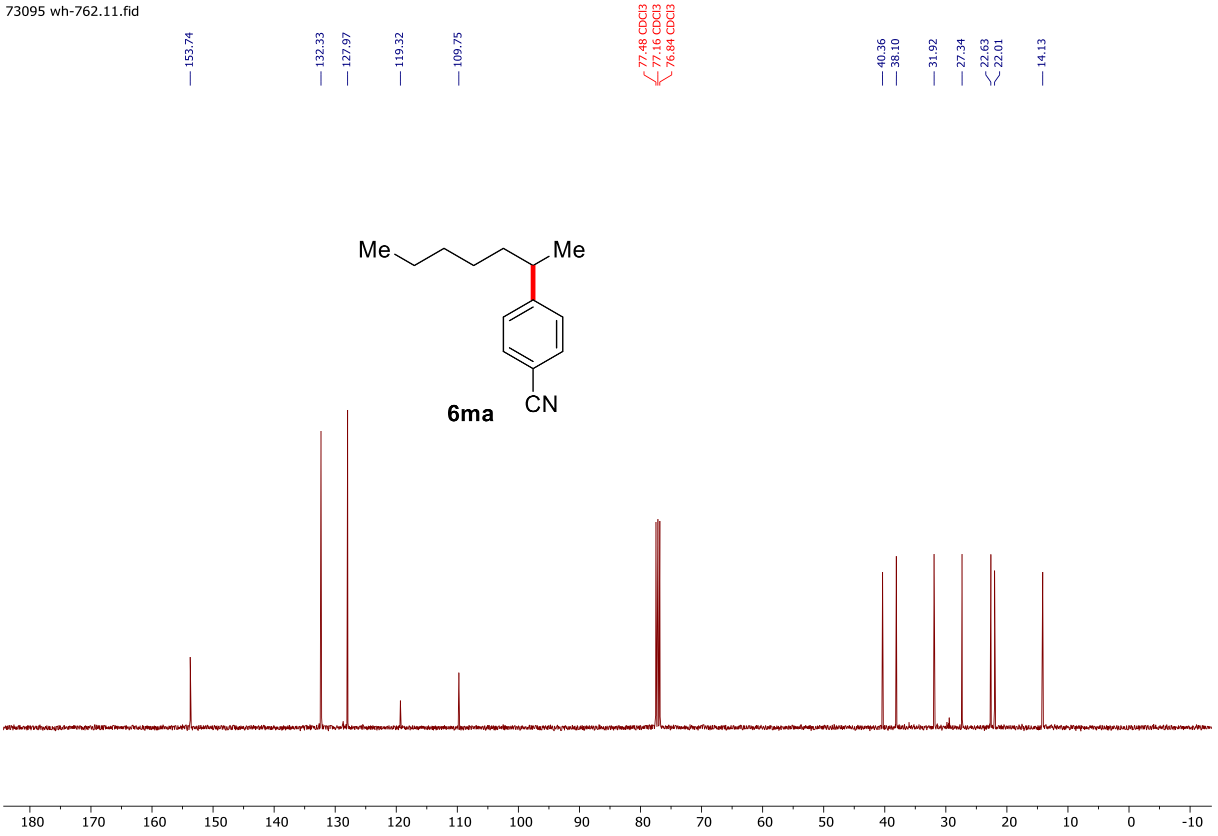

^{1}H NMR (400 MHz, $CDCl_3$) of **6na** (see procedure)

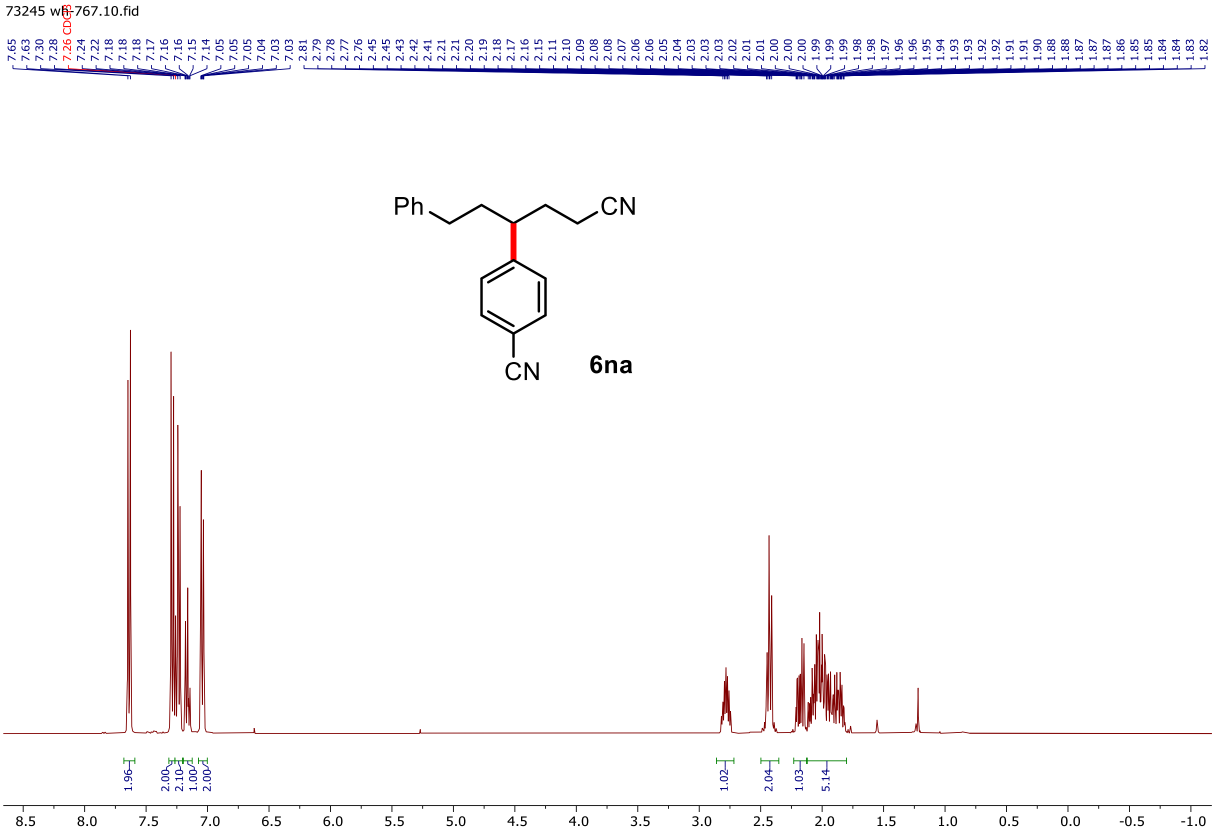

^{13}C NMR (101 MHz, $CDCl_3$) of **6na**

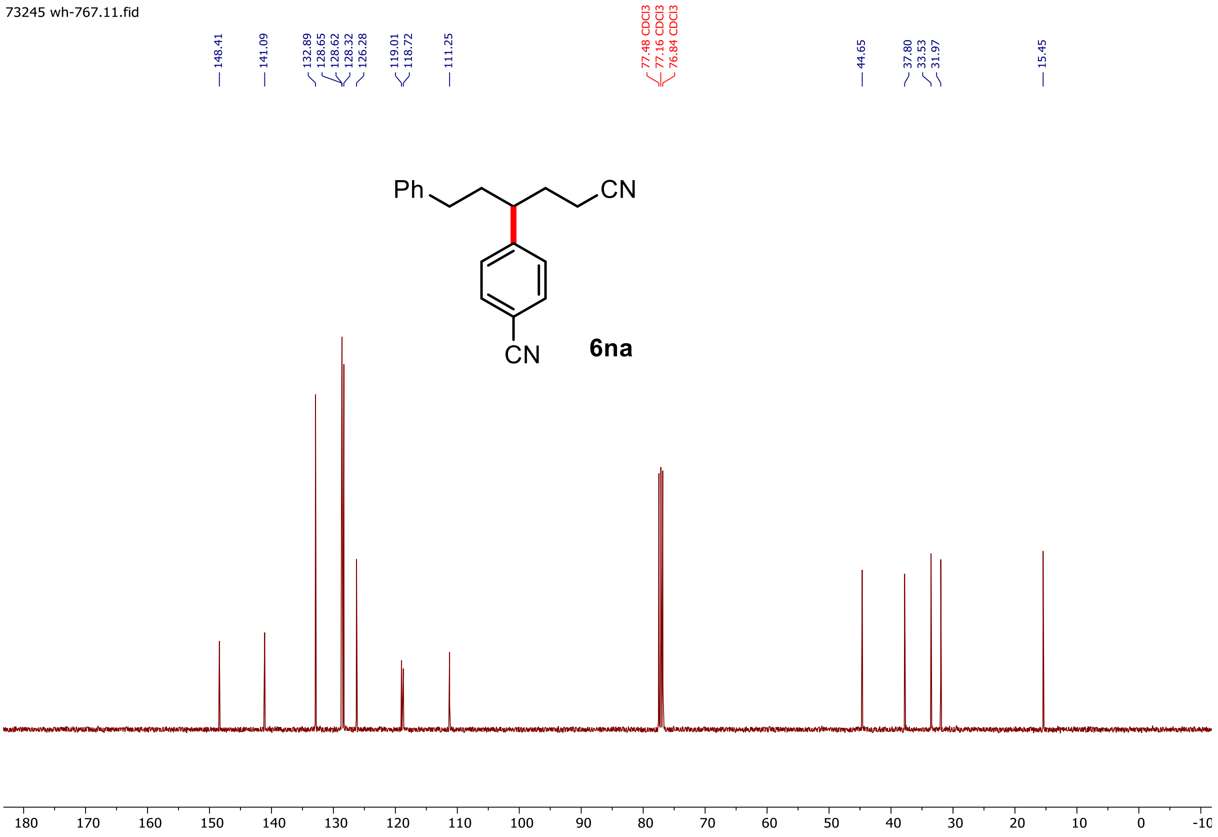

^{1}H NMR (400 MHz, $CDCl_3$) of **6oa** (see procedure)

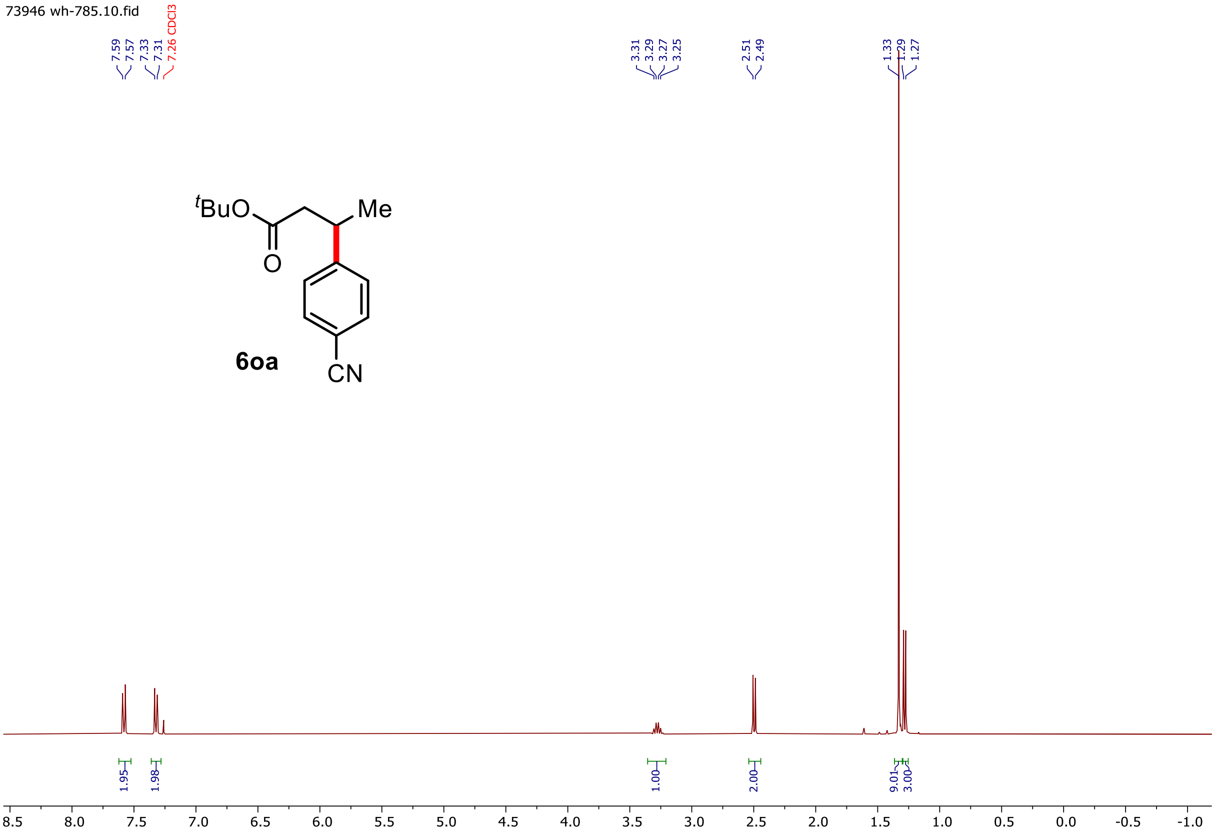

^{13}C NMR (101 MHz, $CDCl_3$) of **6oa**

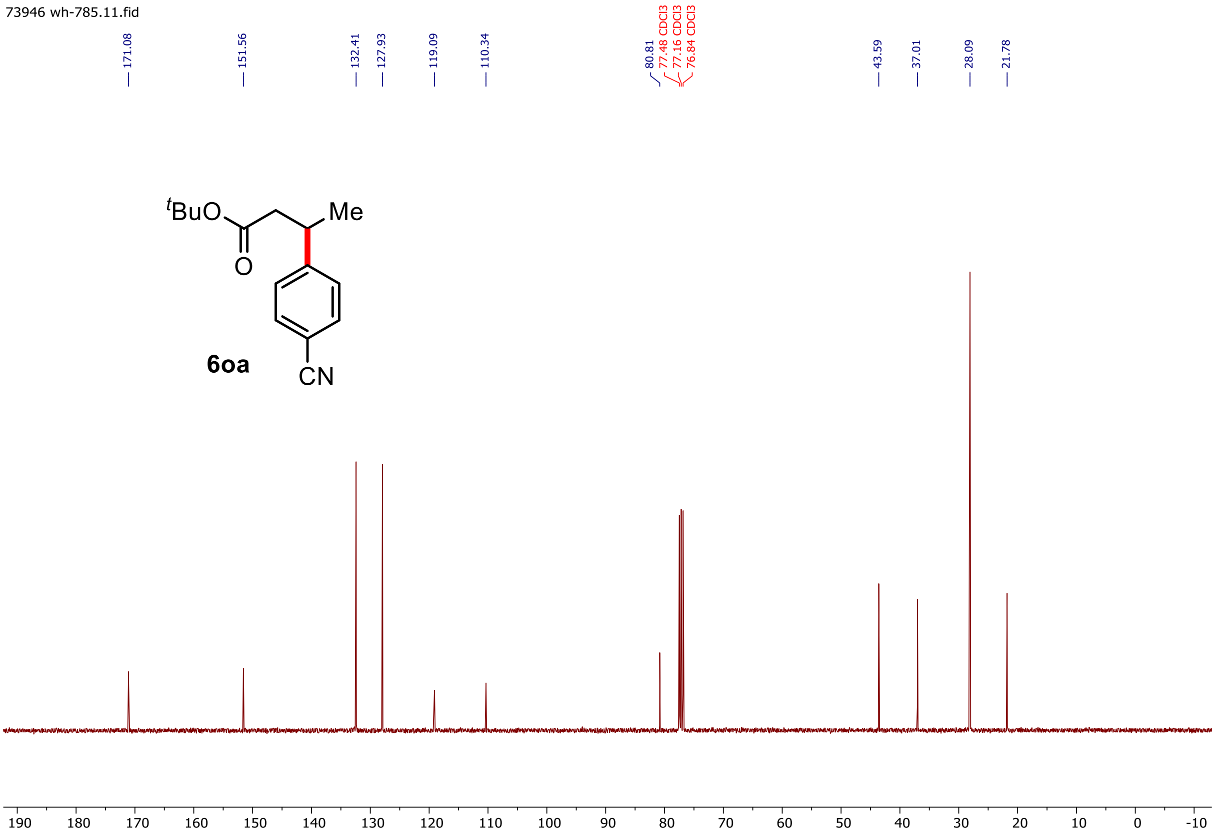

^{1}H NMR (400 MHz, $CDCl_3$) of **6pa** (see procedure)

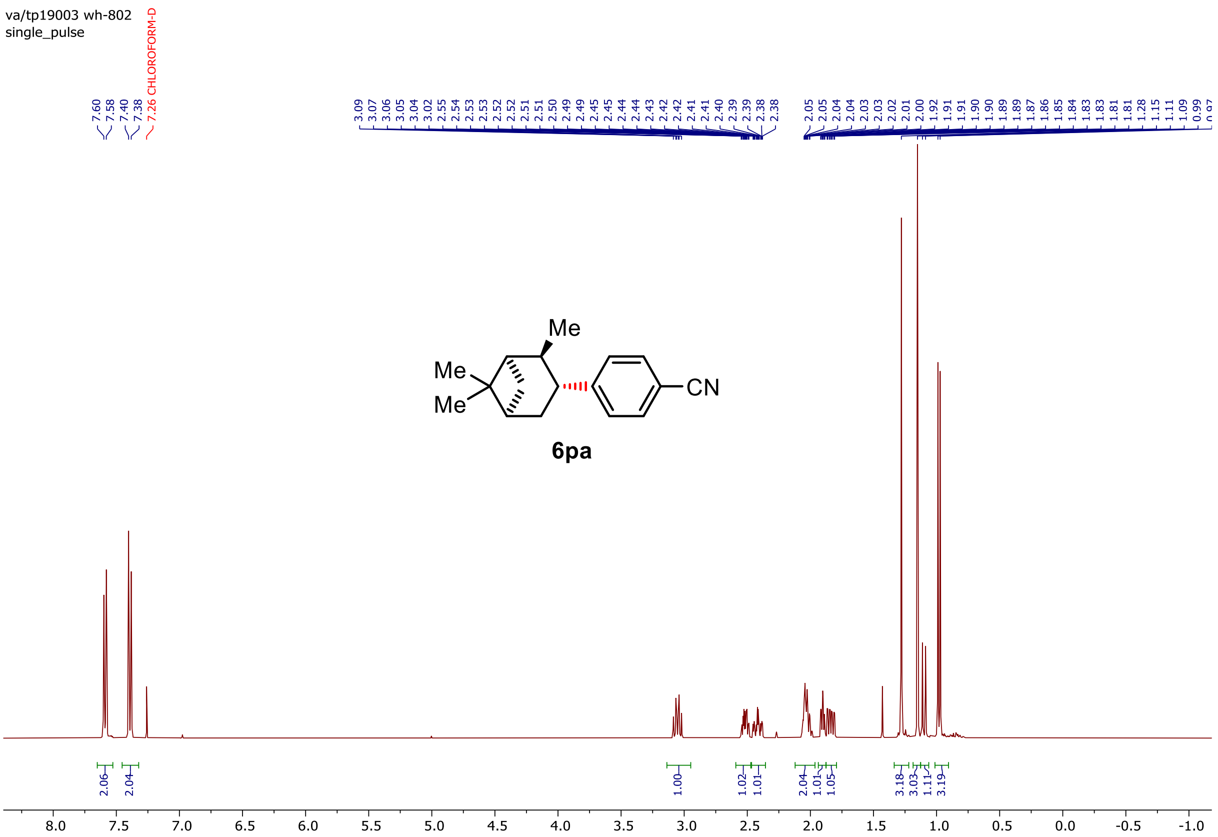

13C NMR (101 MHz, $CDCl_3$) of **6pa**

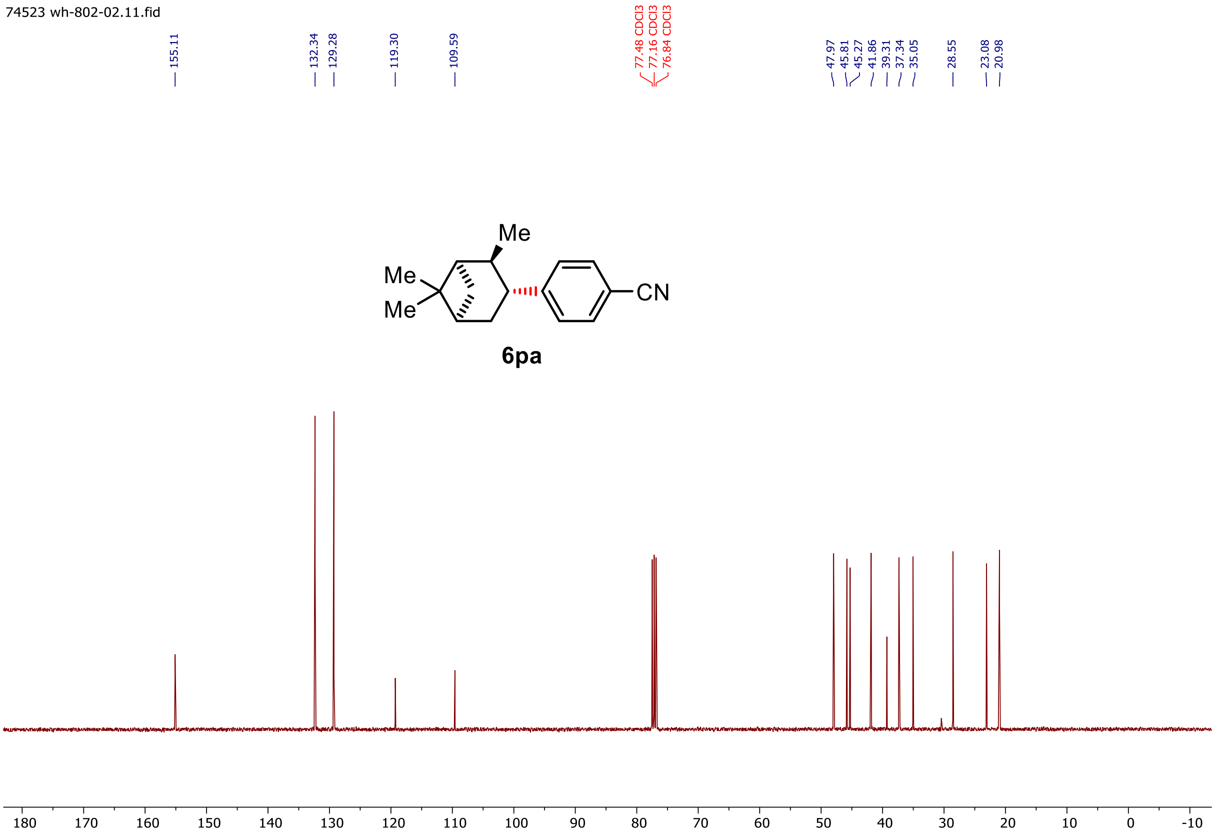

^{1}H NMR (400 MHz, $CDCl_3$) of **6qa** (<u>see procedure</u>)

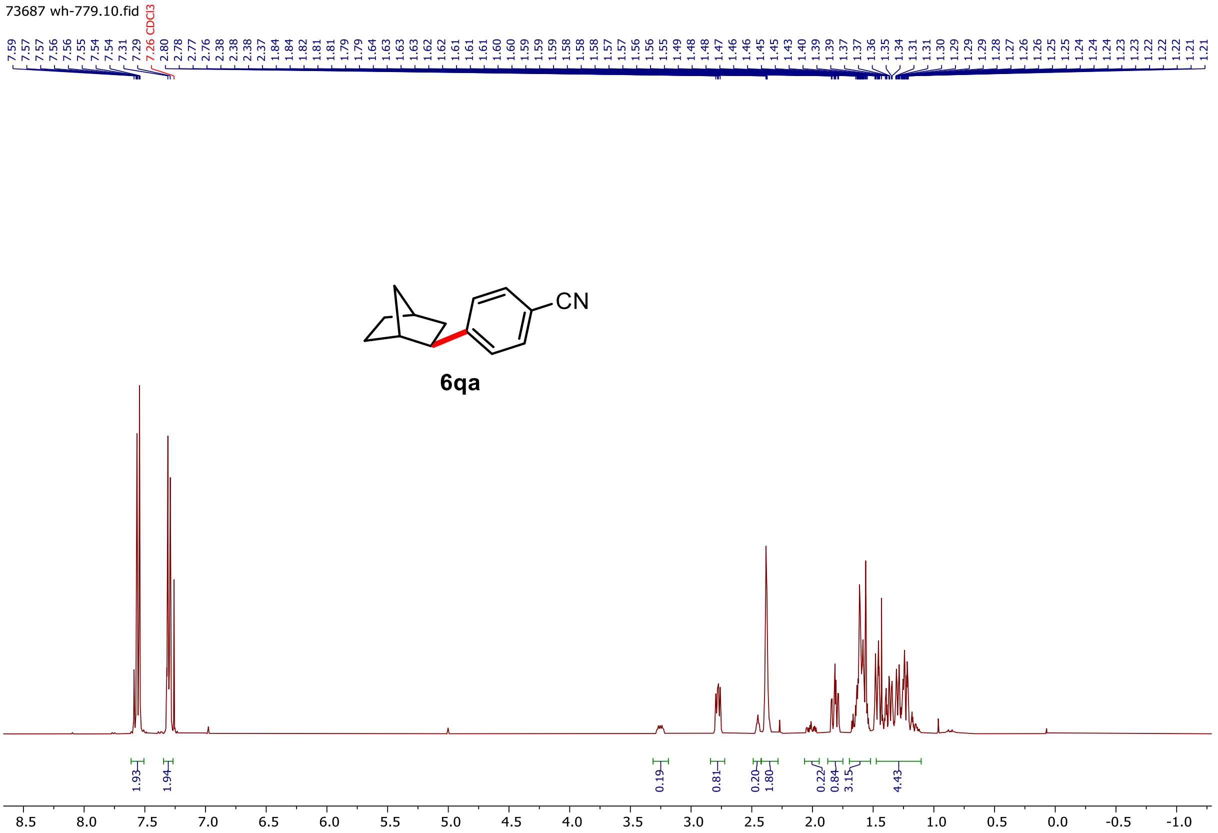

^{13}C NMR (101 MHz, $CDCl_3$) of **6qa**

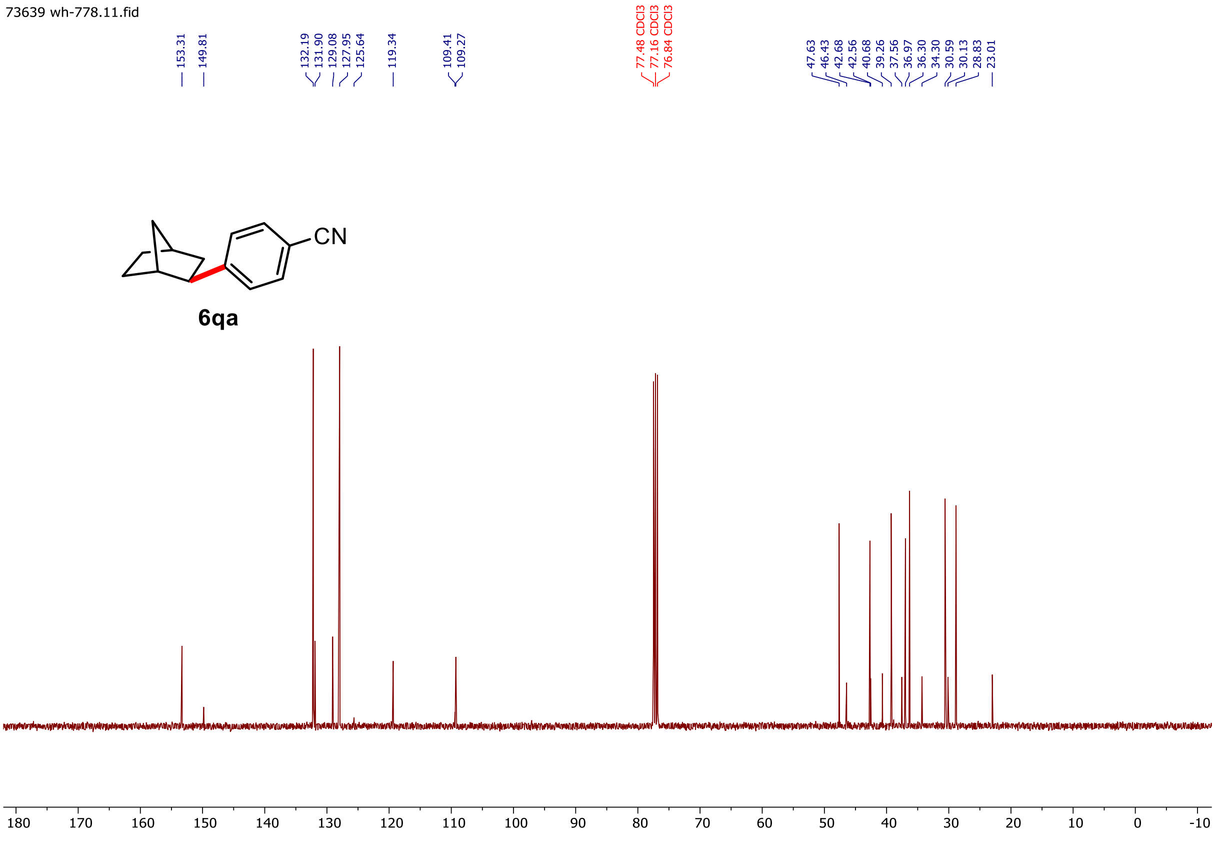

UOB Confidential & Sensitive

^{1}H NMR (400 MHz, $CDCl_3$) of **6ra** (*see procedure*)

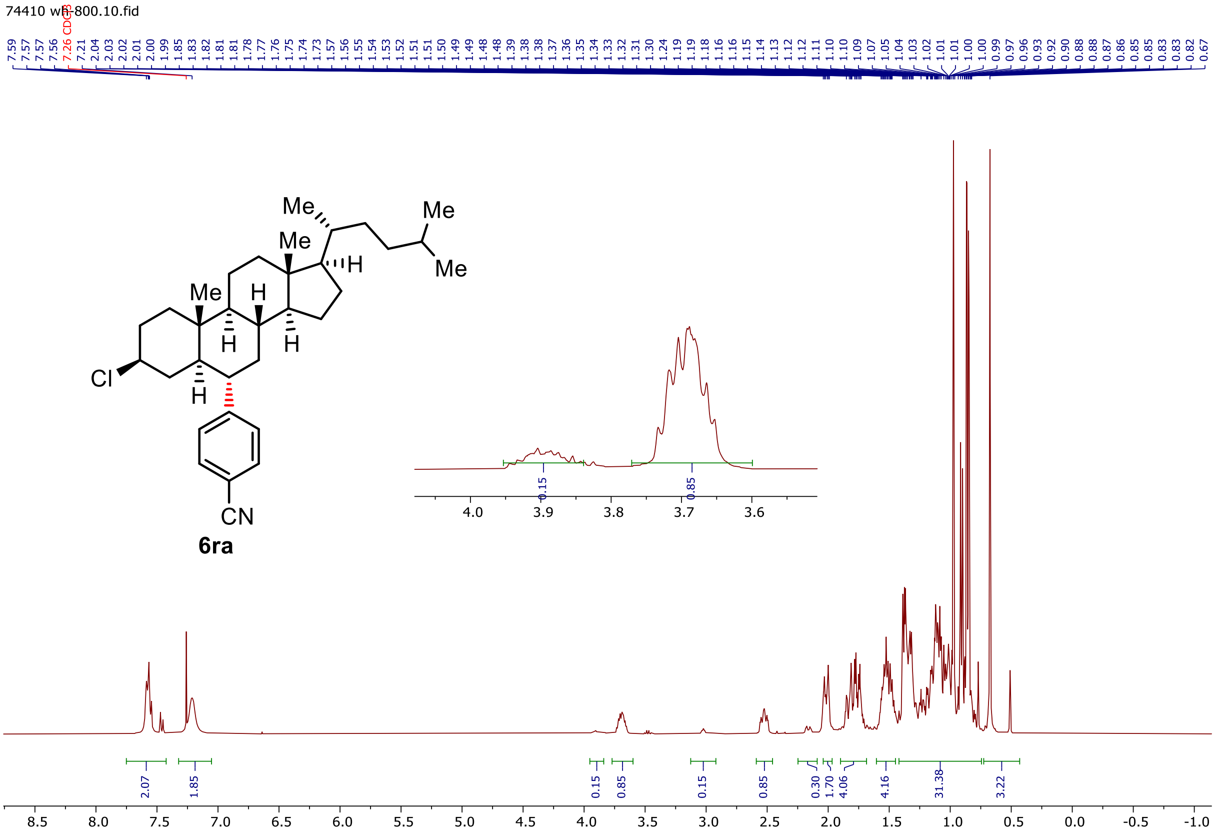

^{13}C NMR (101 MHz, $CDCl_3$) of **6ra**

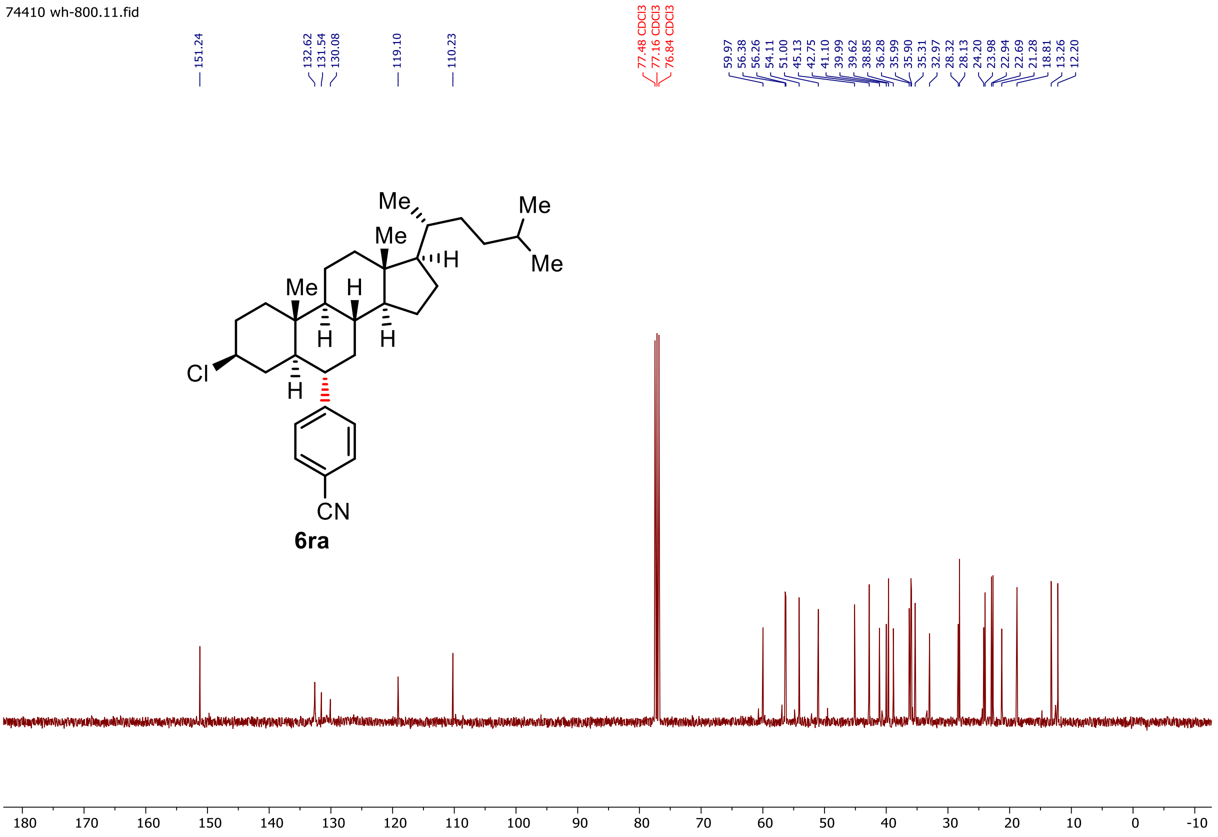

^{1}H NMR (400 MHz, $CDCl_3$) of **6sa** (see procedure)

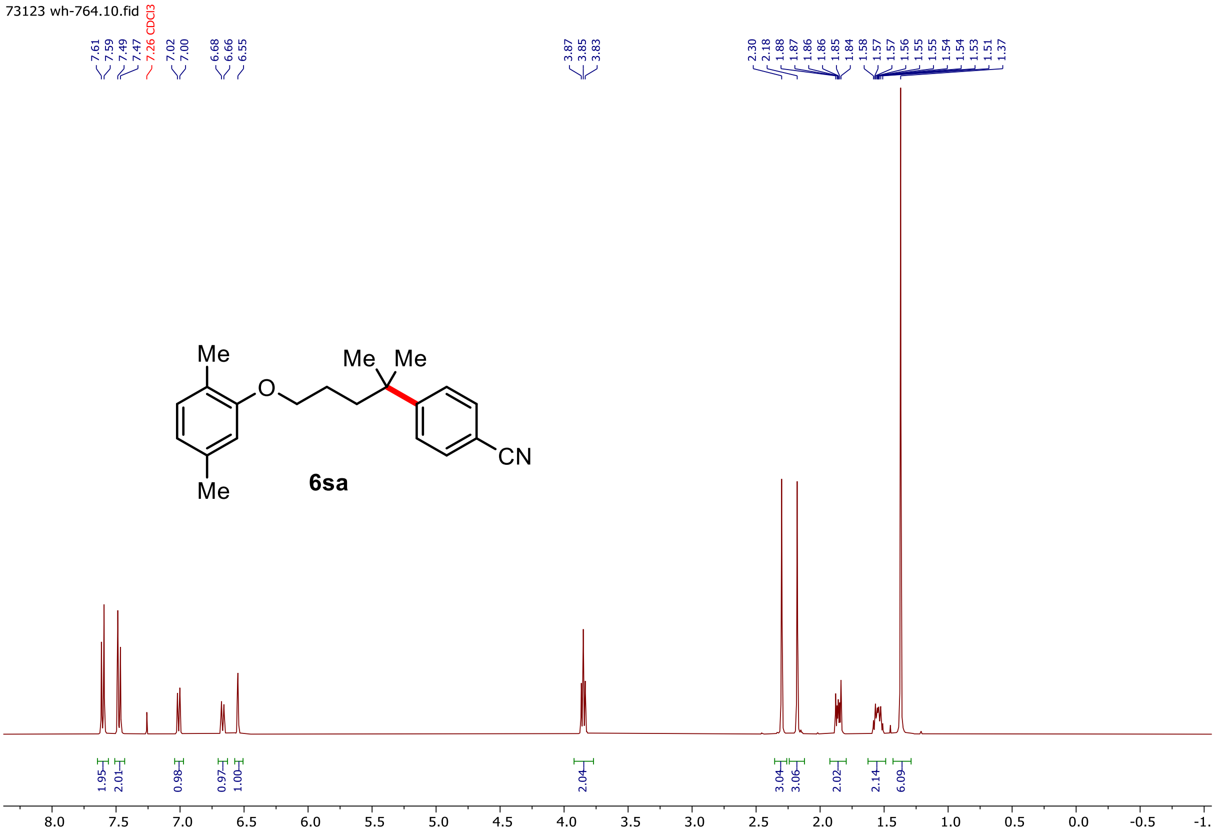

^{13}C NMR (101 MHz, $CDCl_3$) of **6sa**

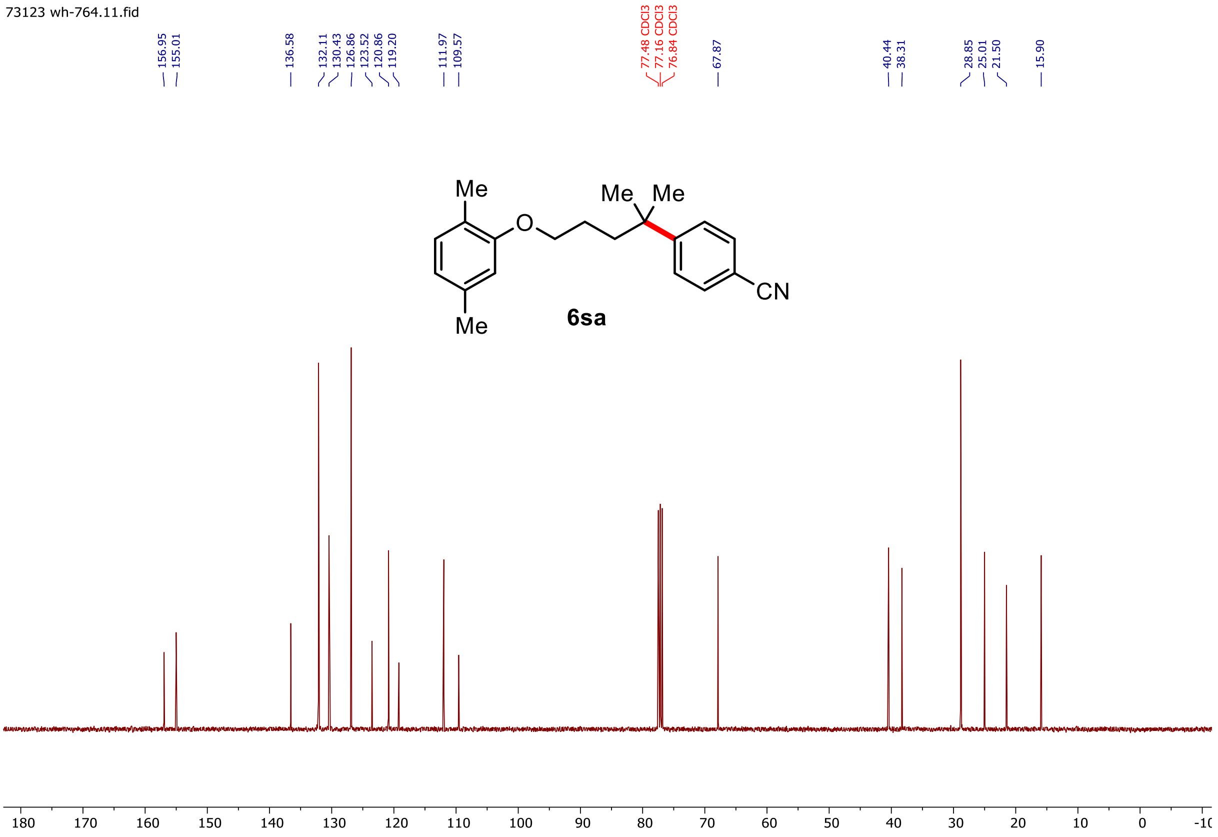

^{1}H NMR (400 MHz, $CDCl_3$) of **6ta** (see procedure)

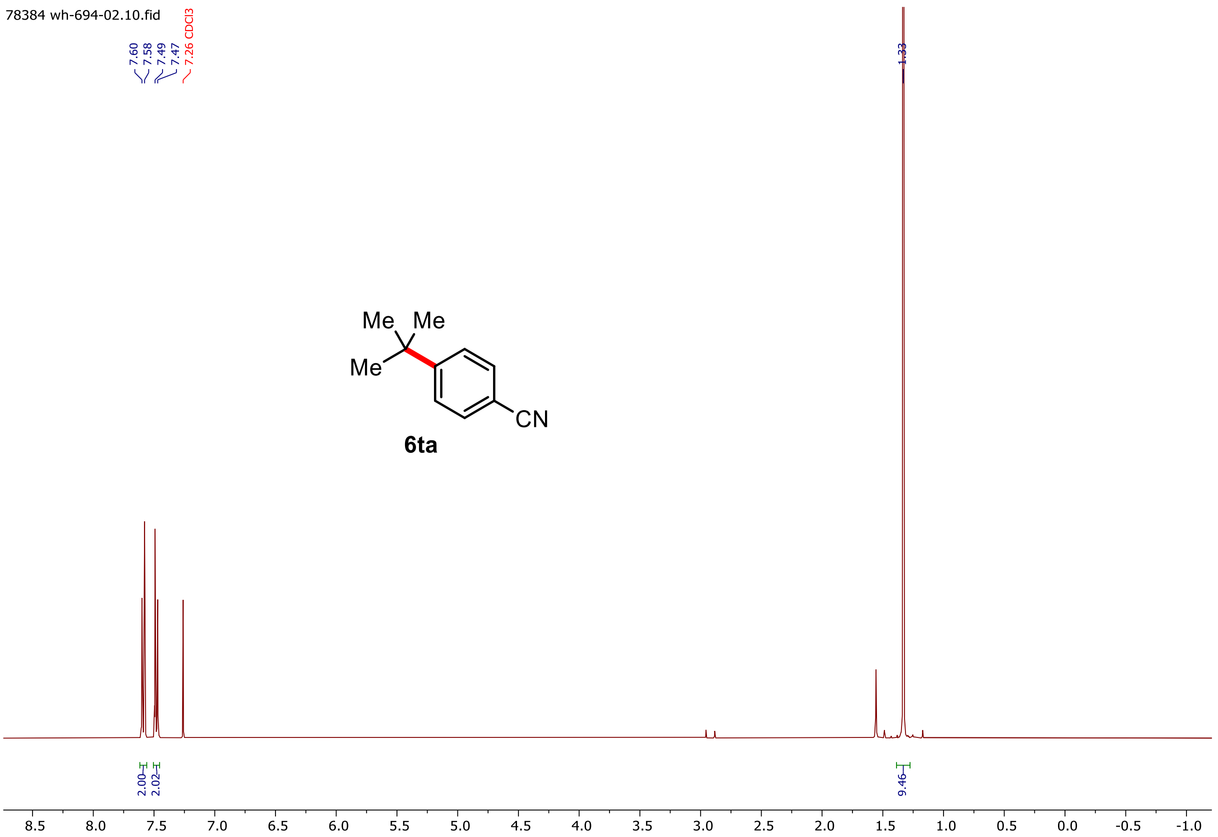

^{13}C NMR (101 MHz, $CDCl_3$) of **6ta**

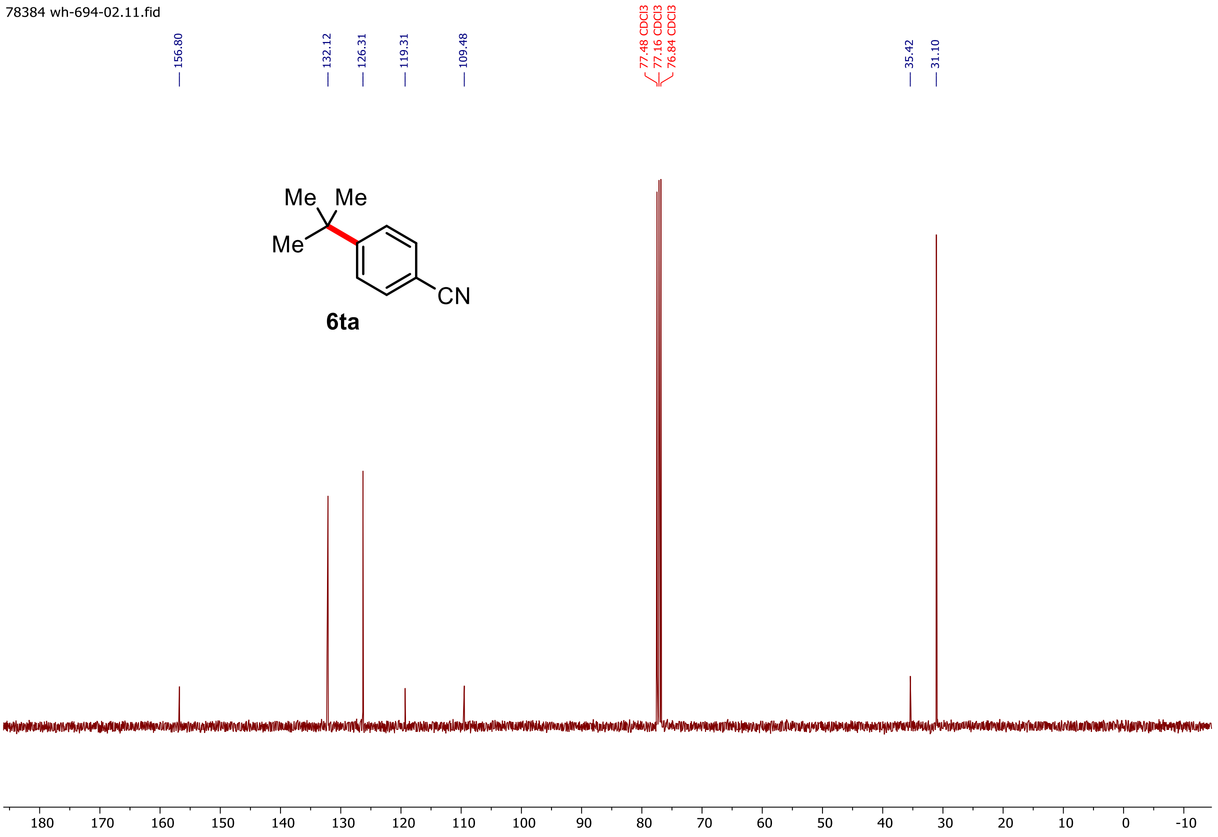

^{1}H NMR (400 MHz, $CDCl_3$) of **6ua** (see procedure)

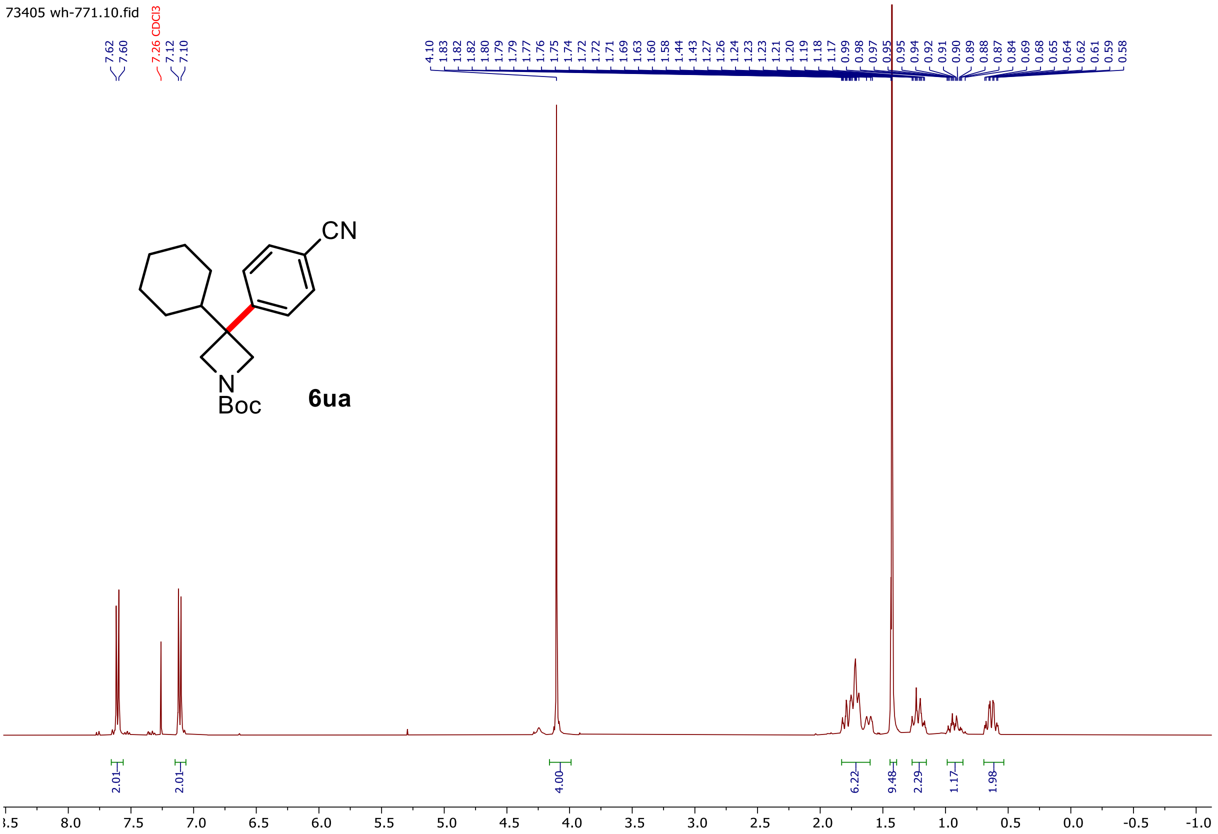

^{13}C NMR (101 MHz, $CDCl_3$) of **6ua**

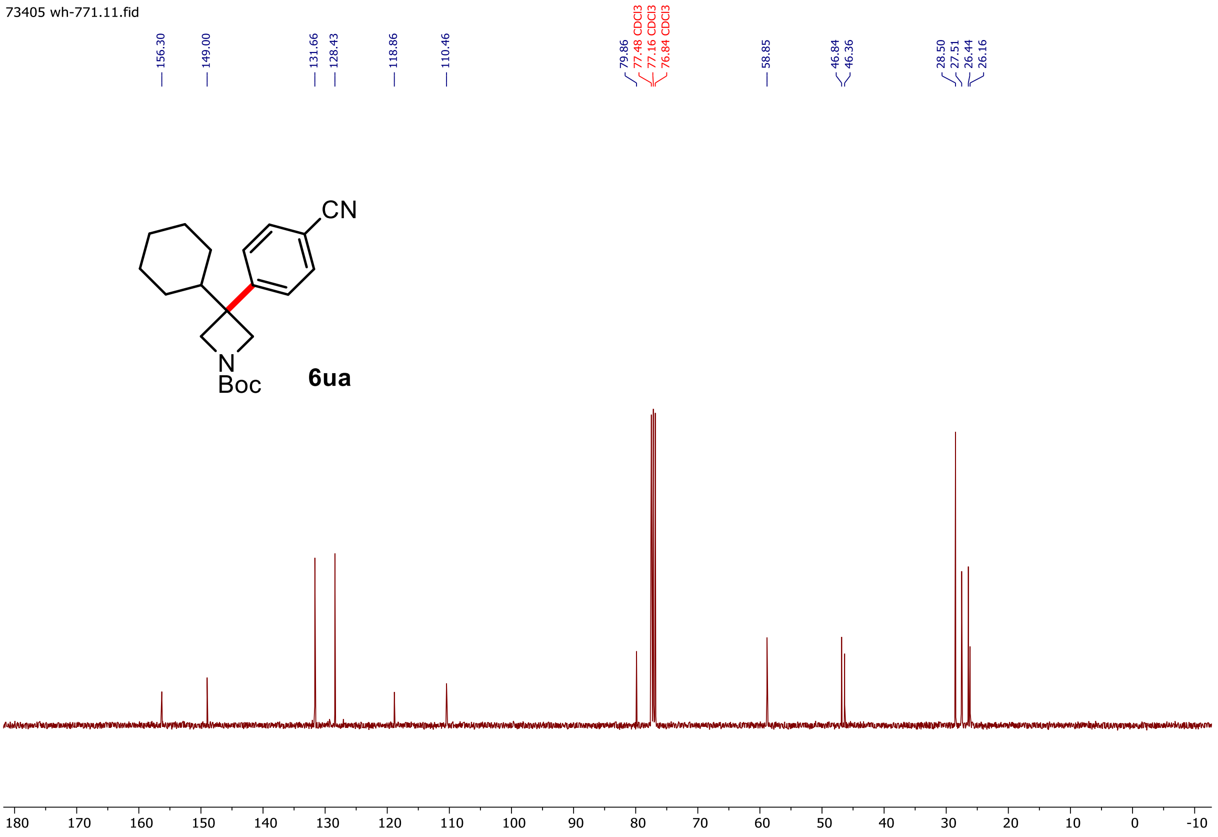

^{1}H NMR (400 MHz, $CDCl_3$) of **6va** (*see procedure*)

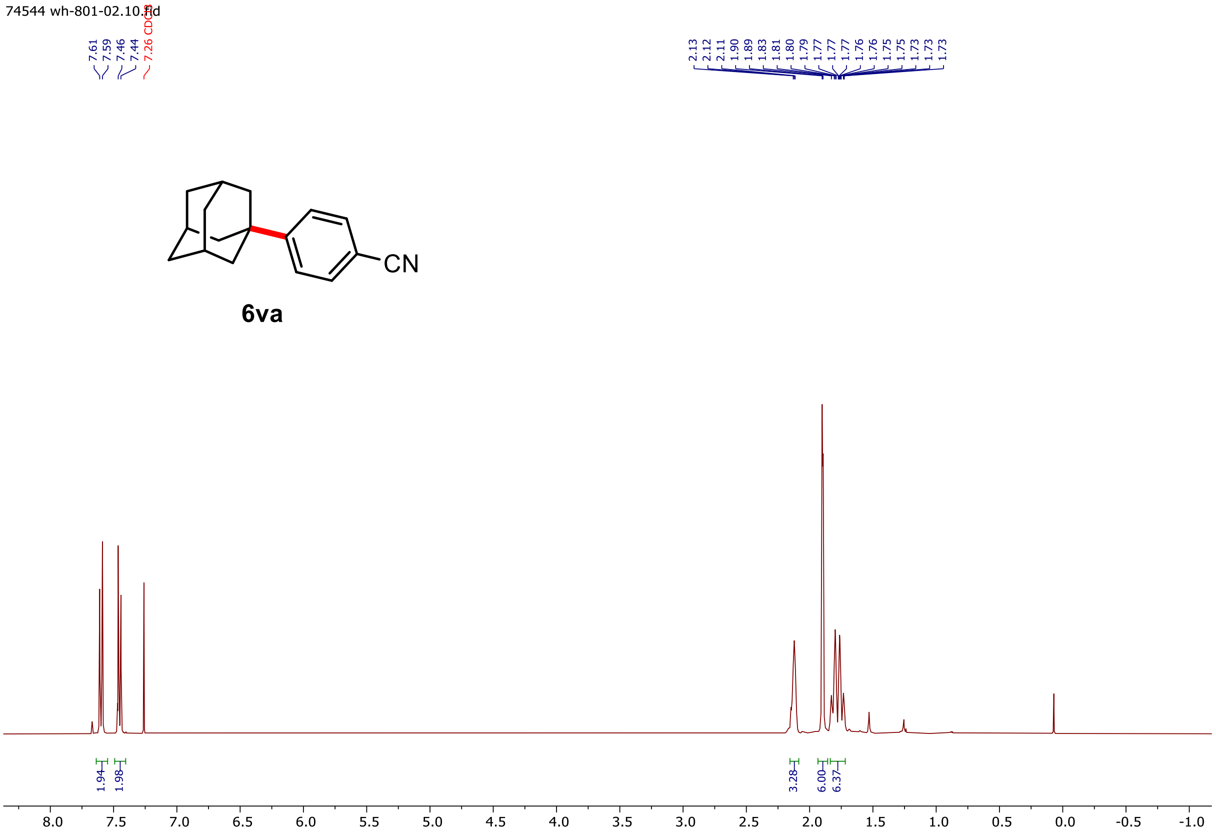

^{13}C NMR (101 MHz, $CDCl_3$) of **6va**

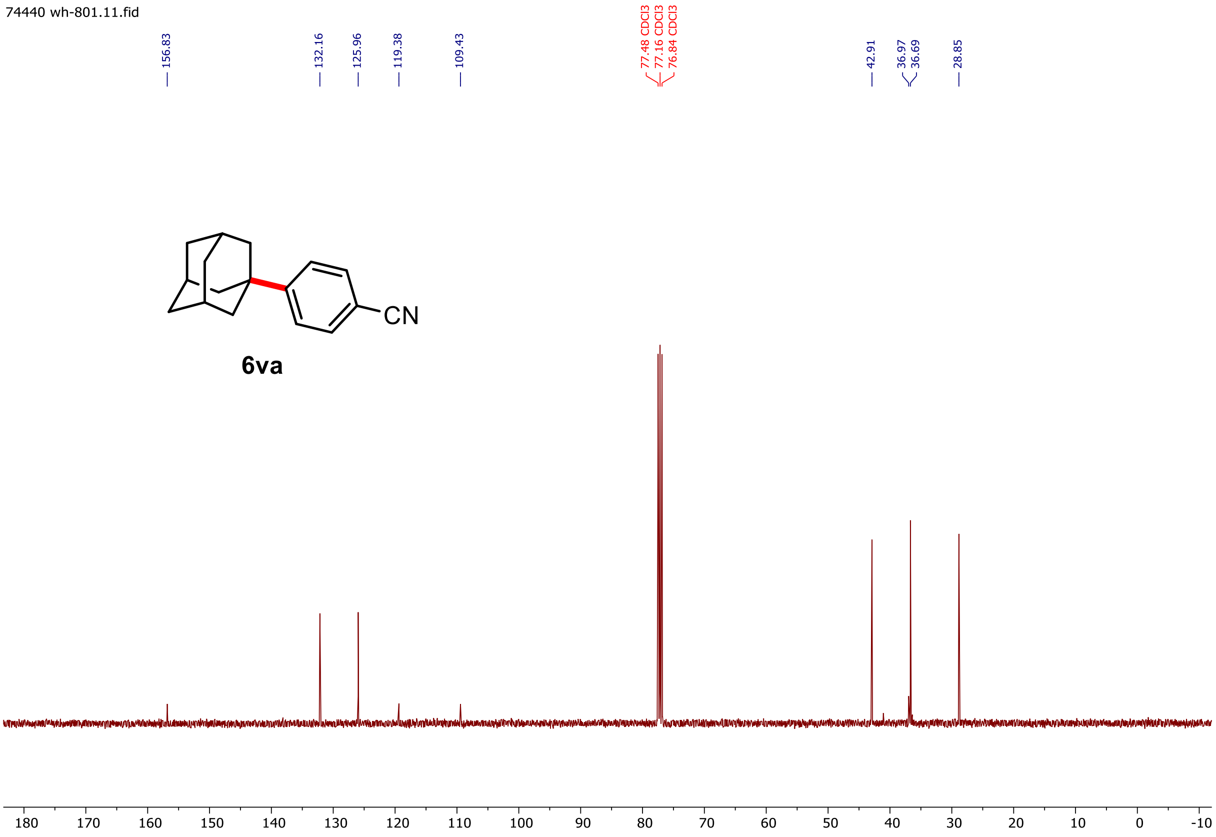

^{1}H NMR (400 MHz, $CDCl_3$) of **6hb** (*see procedure*)

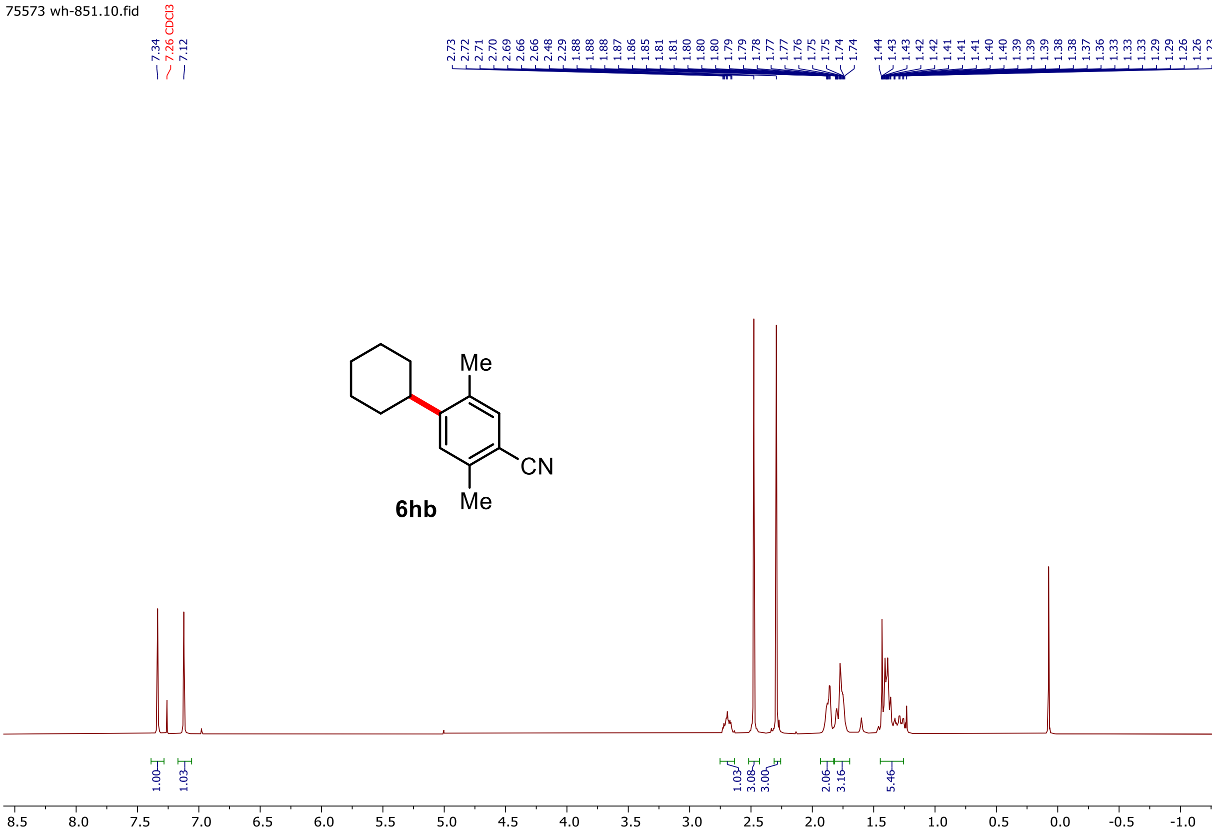

^{13}C NMR (101 MHz, $CDCl_3$) of **6hb**

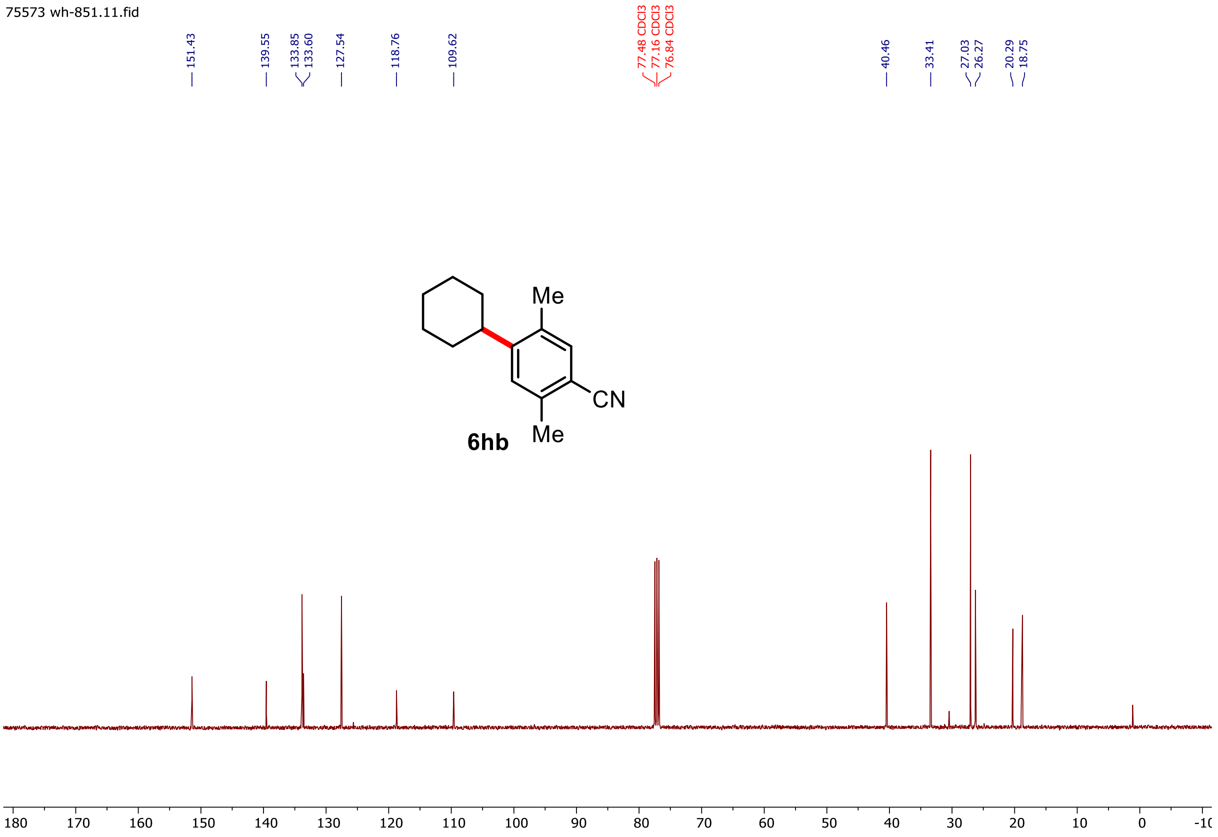

^{1}H NMR (400 MHz, $CDCl_3$) of **6hc** (*see procedure*)

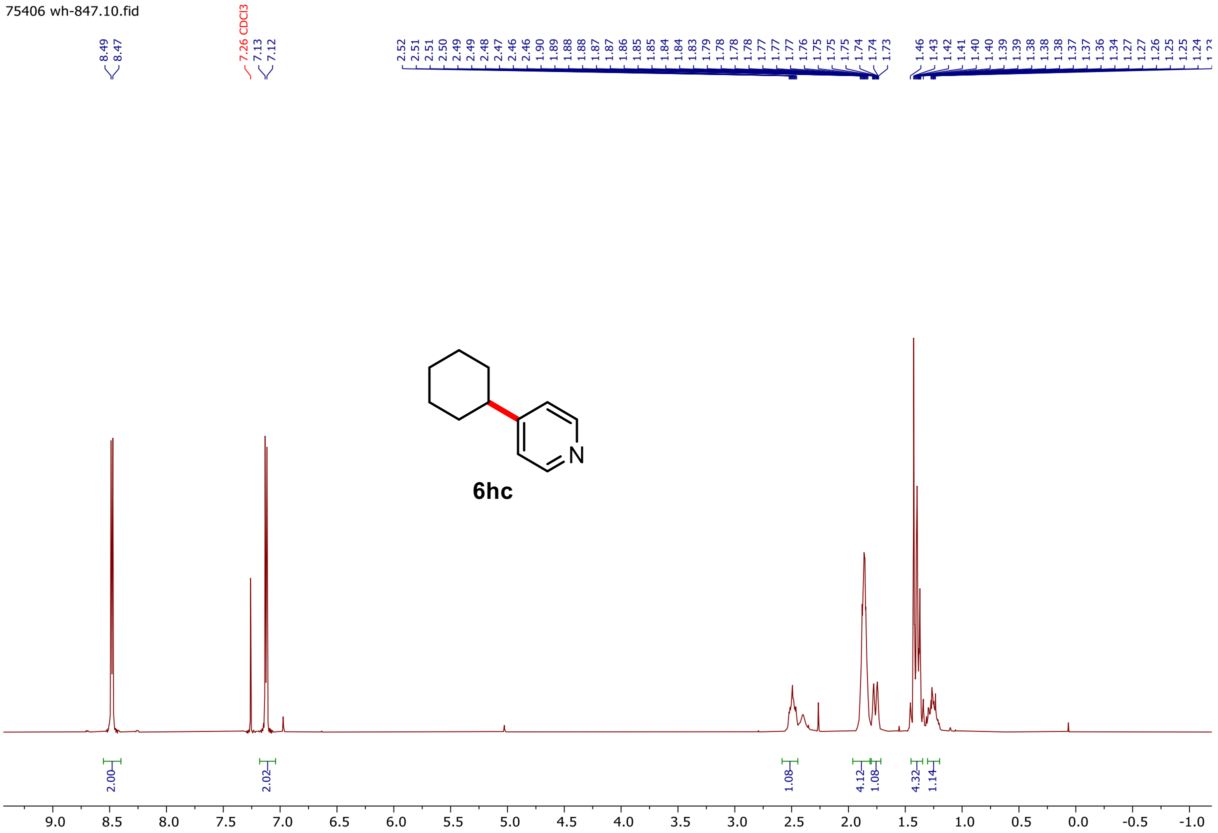

^{13}C NMR (101 MHz, $CDCl_3$) of **6hc**

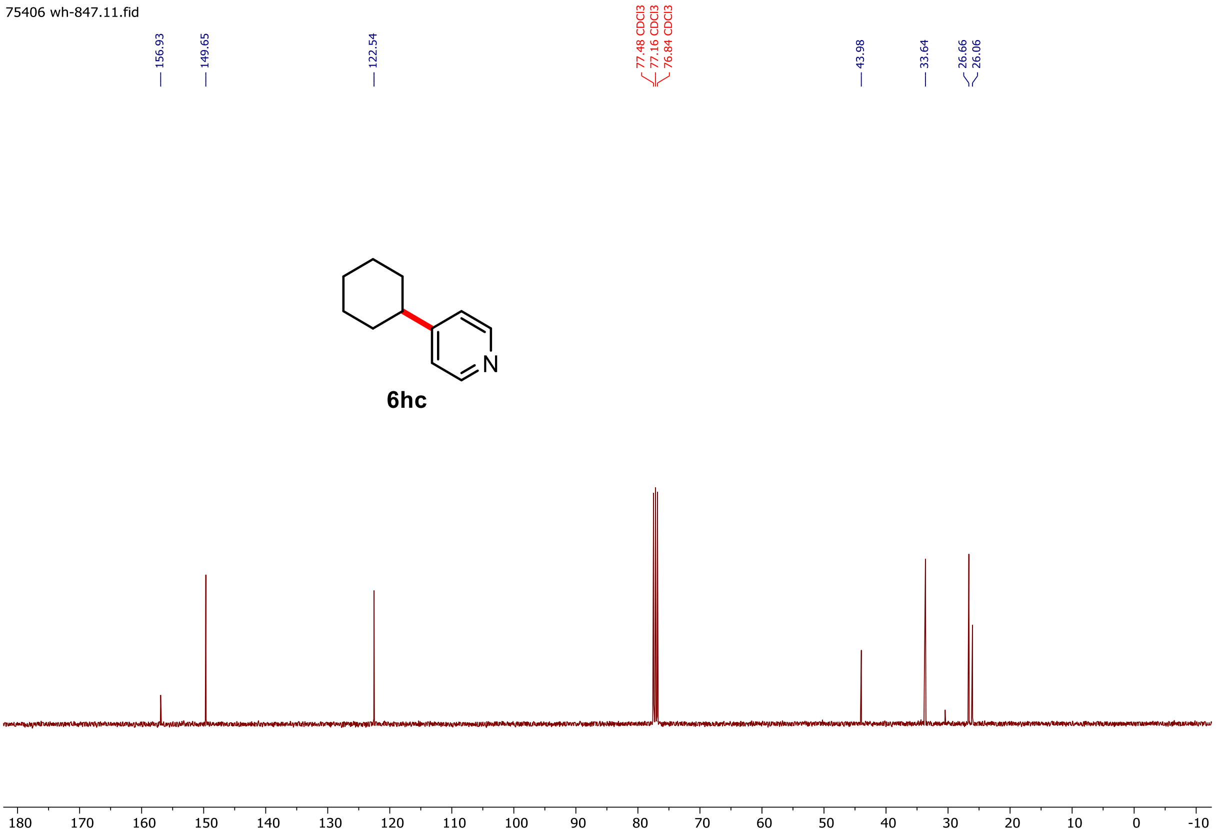

^{1}H NMR (400 MHz, $CDCl_3$) of **6sb** (see procedure)

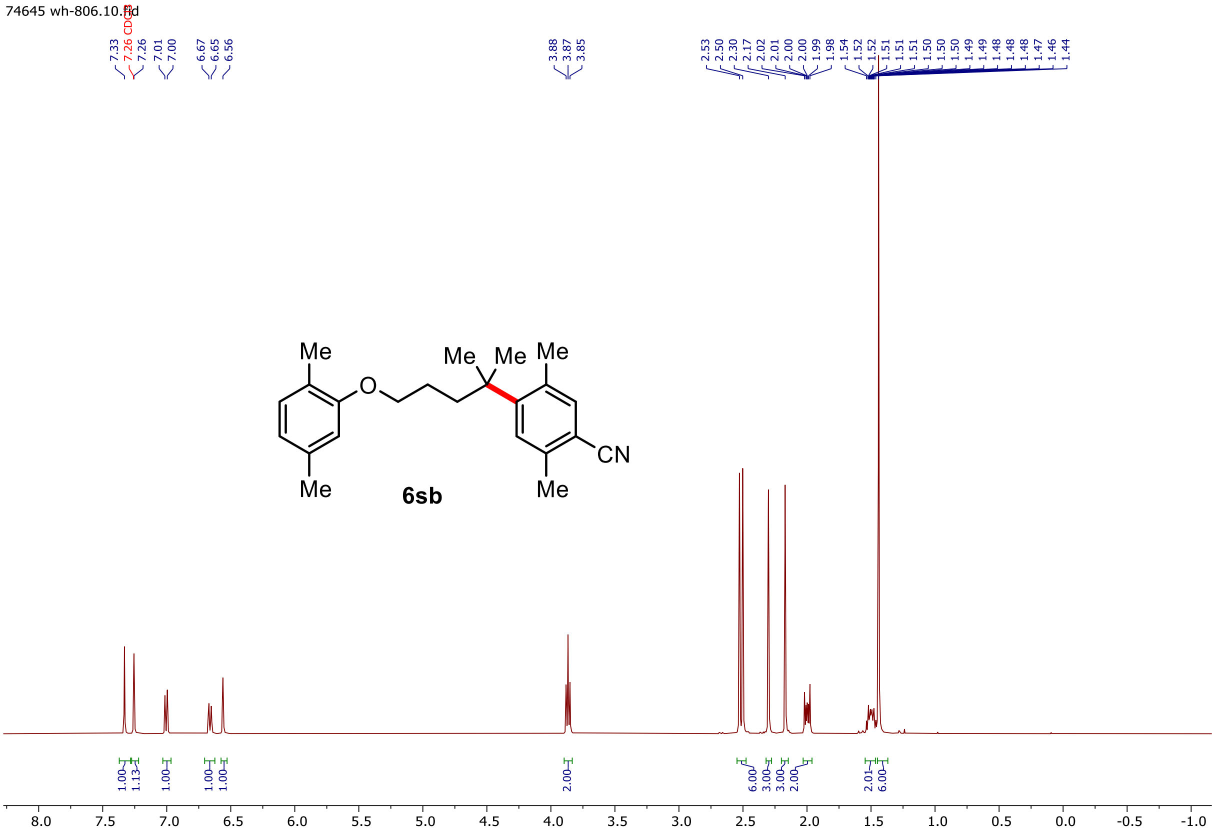

^{13}C NMR (101 MHz, $CDCl_3$) of **6sb**

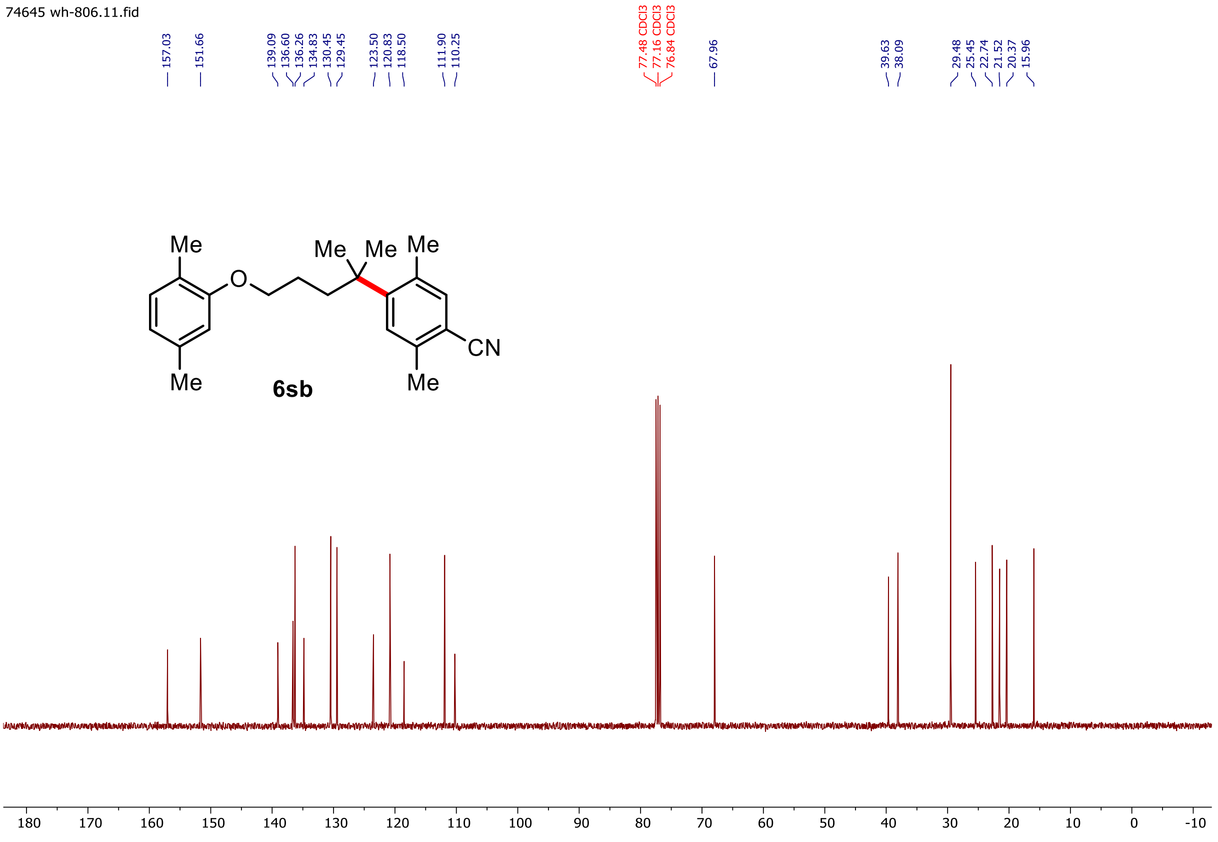

^{1}H NMR (400 MHz, $CDCl_3$) of **6sc** (*see procedure*)

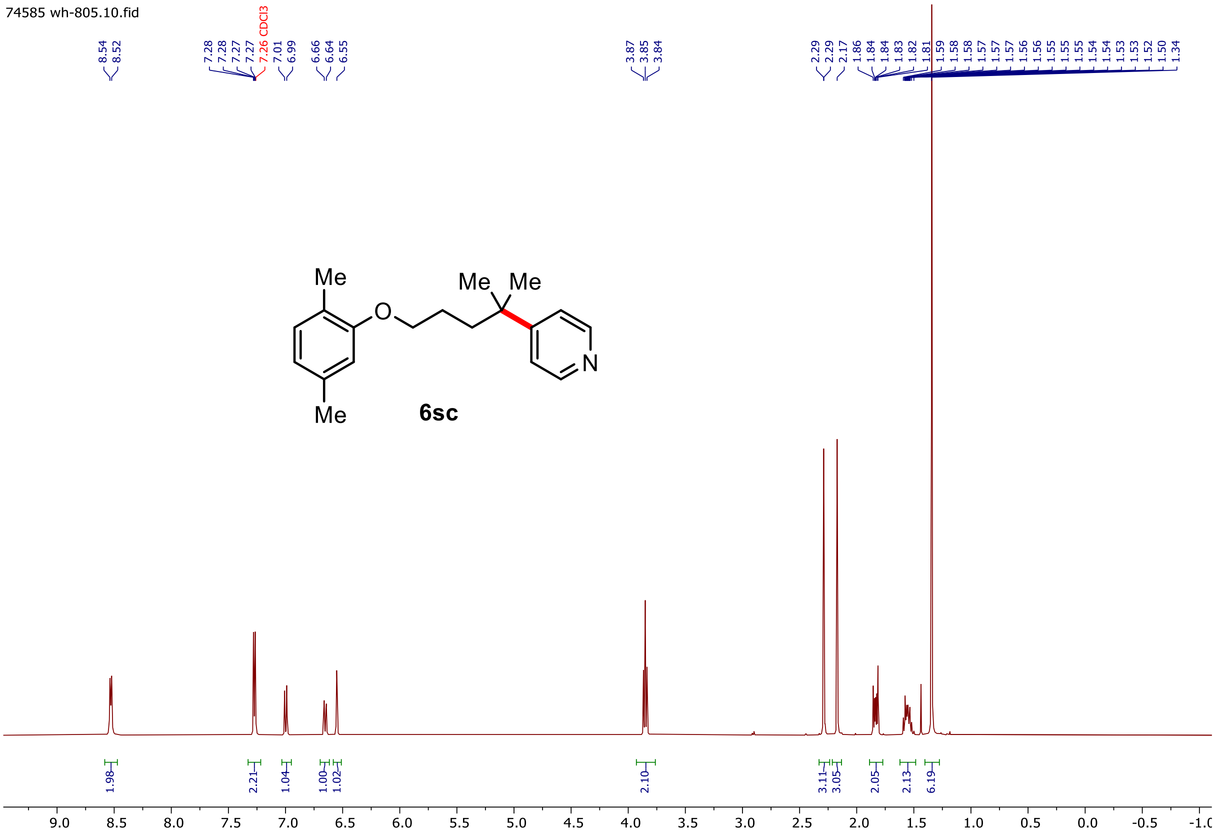

^{13}C NMR (101 MHz, $CDCl_3$) of **6sc**

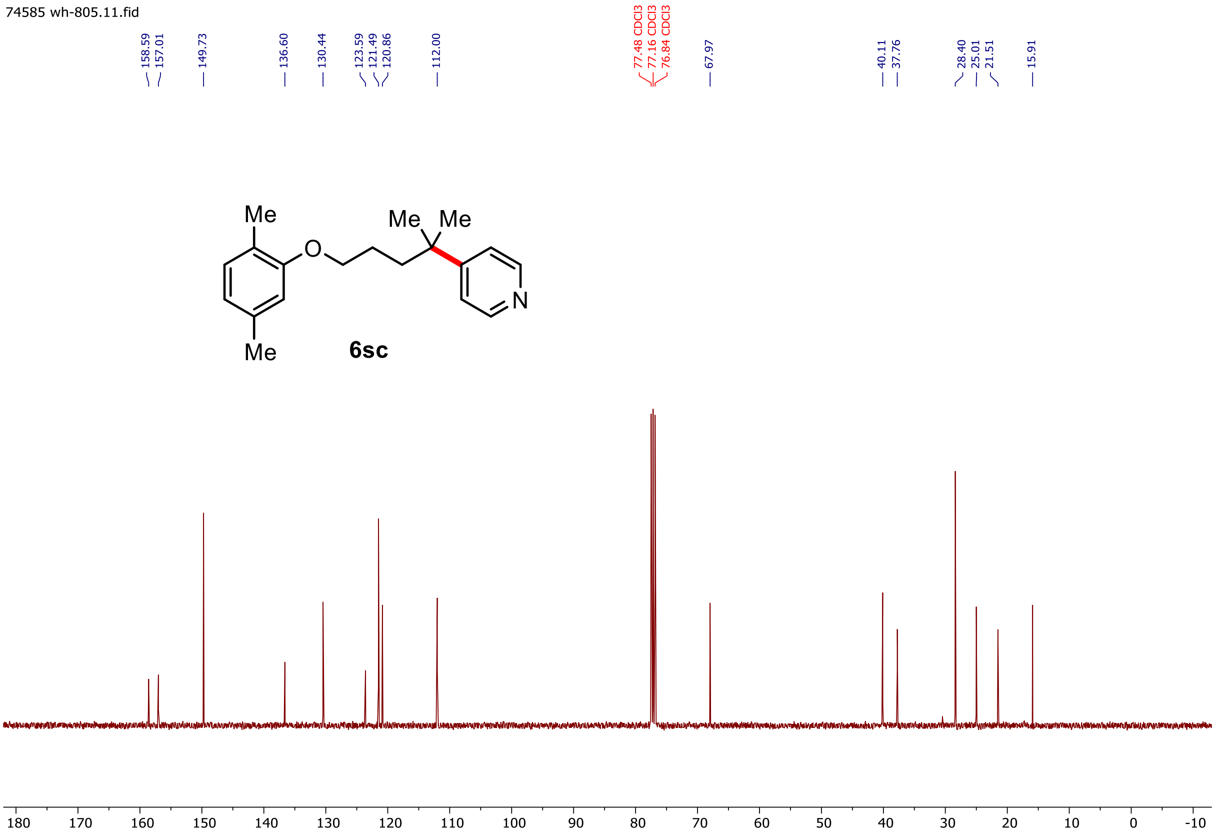

H NMR (400 MHz, $CDCl_3$) of **9** (see procedure)

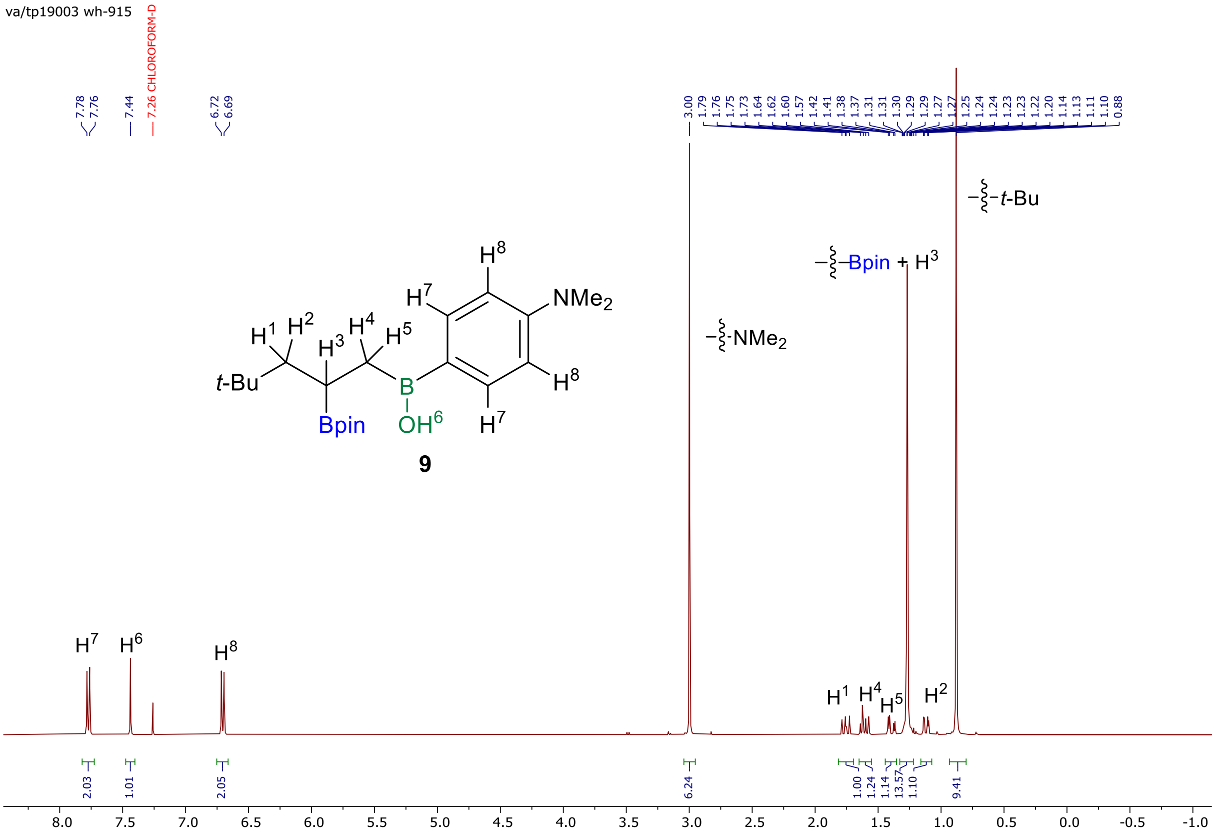

NOESY (400 MHz, $CDCl_3$) of **8**

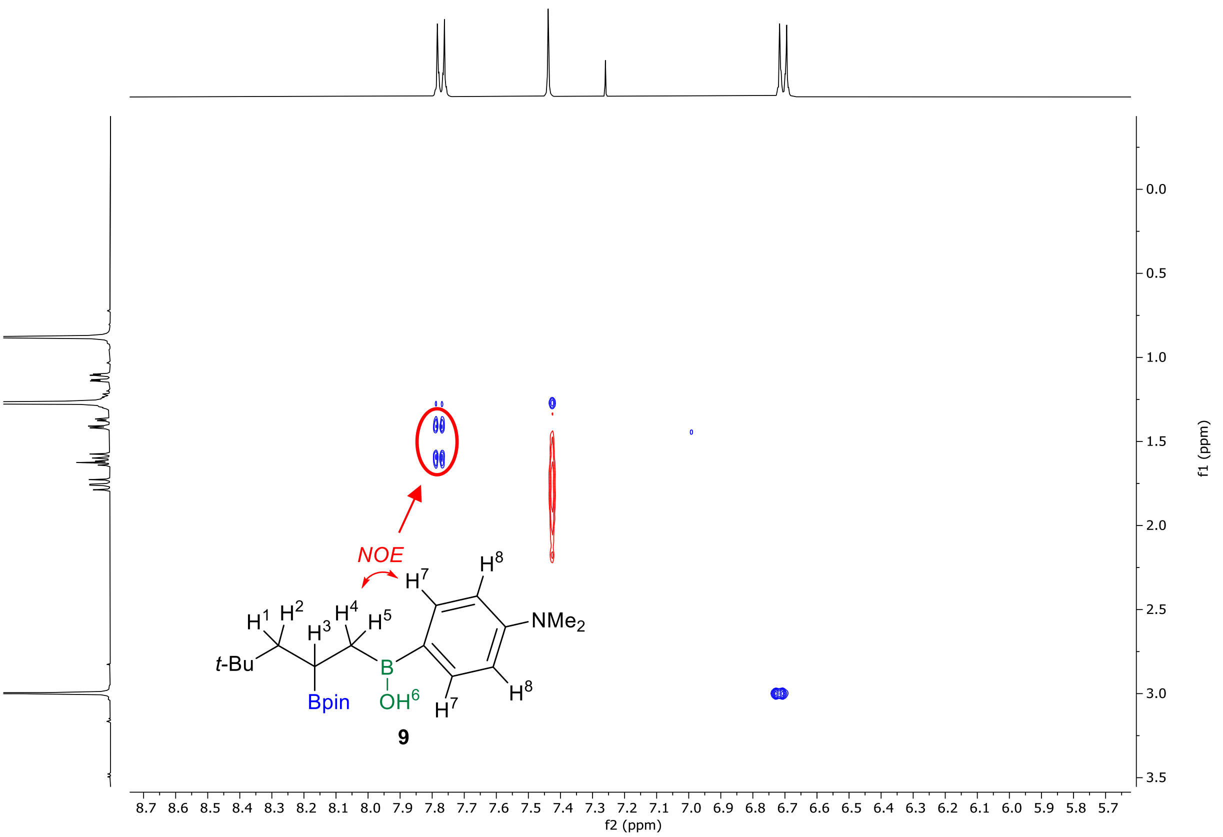

^{13}C NMR (101 MHz, $CDCl_3$) of **9**

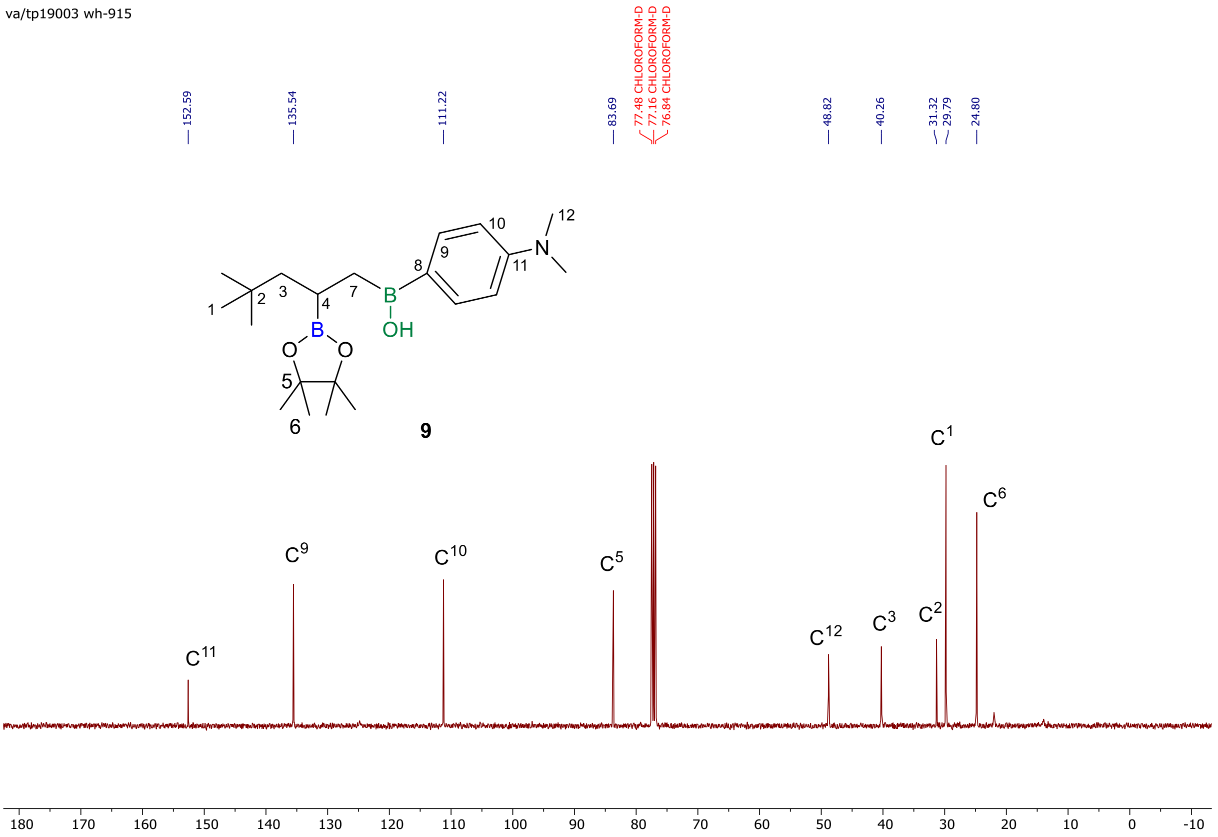

^{11}B NMR (128 MHz, $CDCl_3$) of **9**

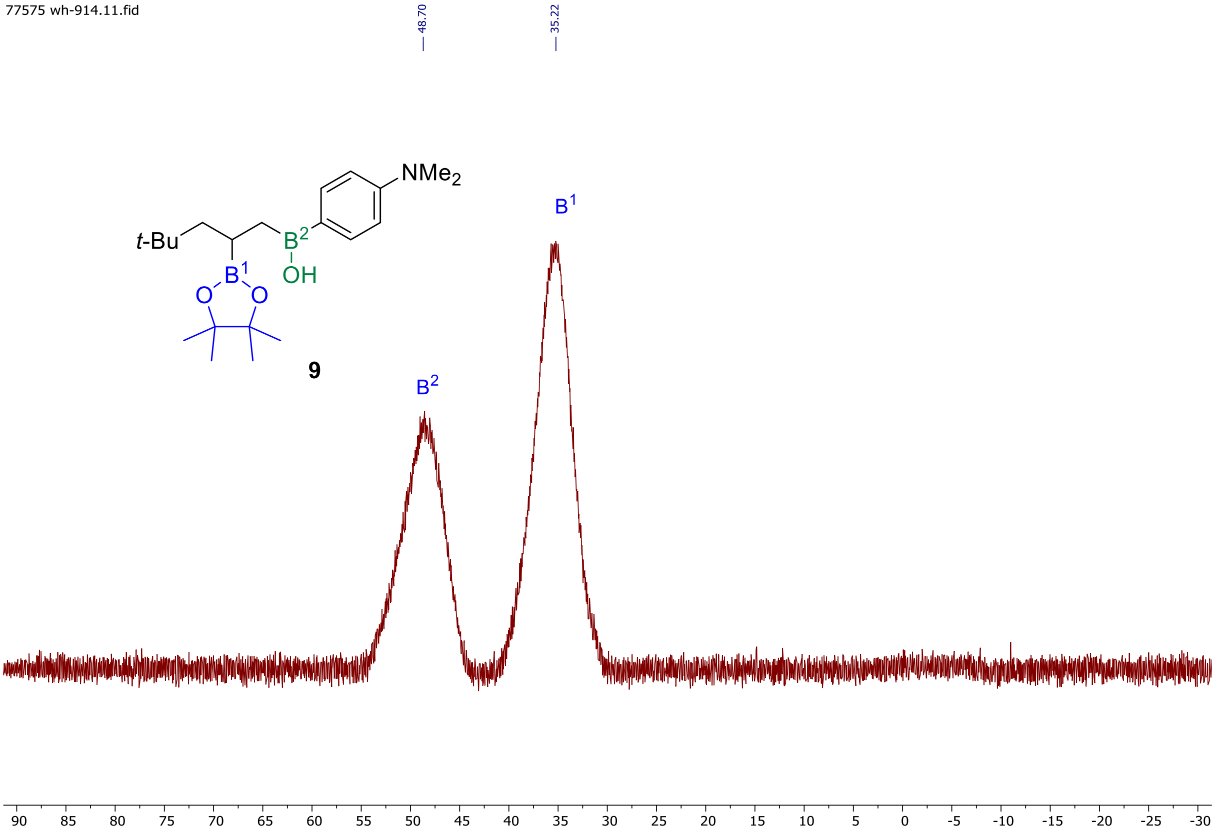

^{1}H NMR (400 MHz, $CDCl_3$) of **7** (see procedure)

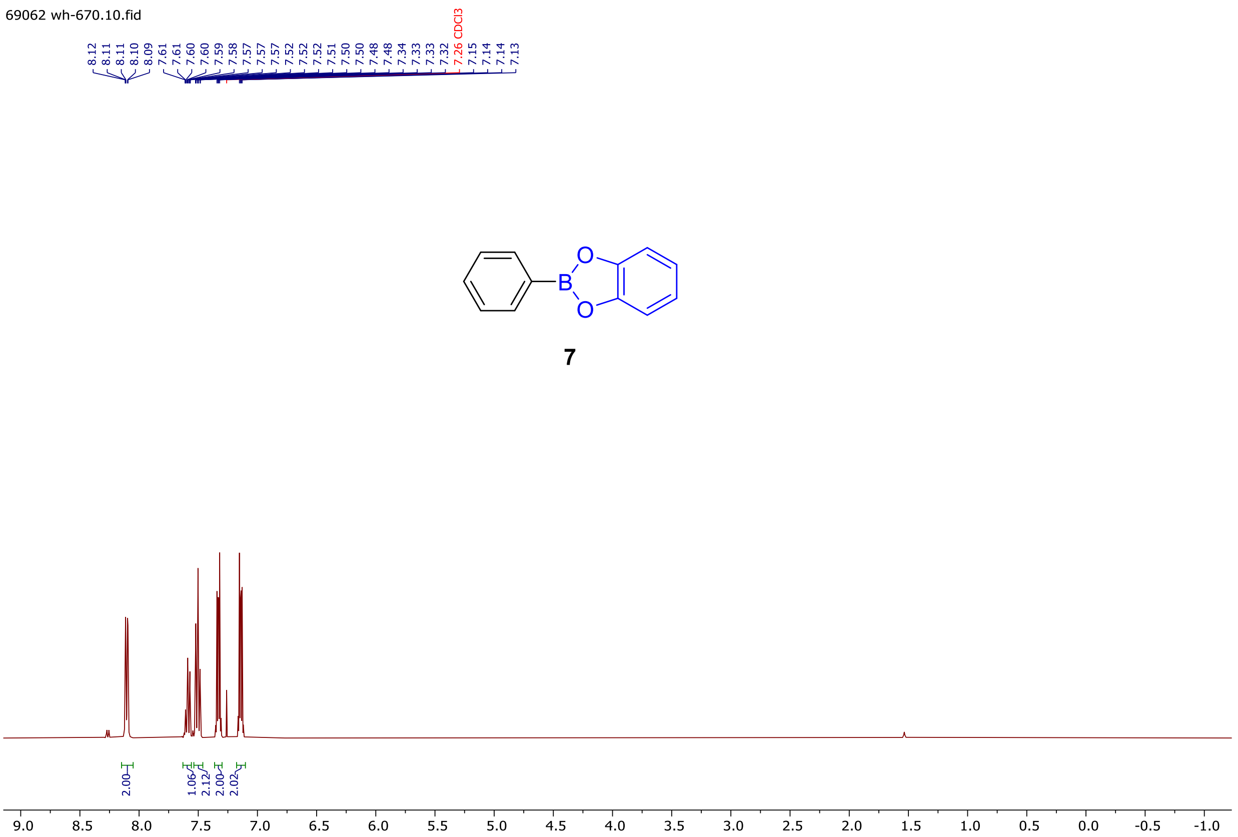

^{13}C NMR (101 MHz, $CDCl_3$) of **7**

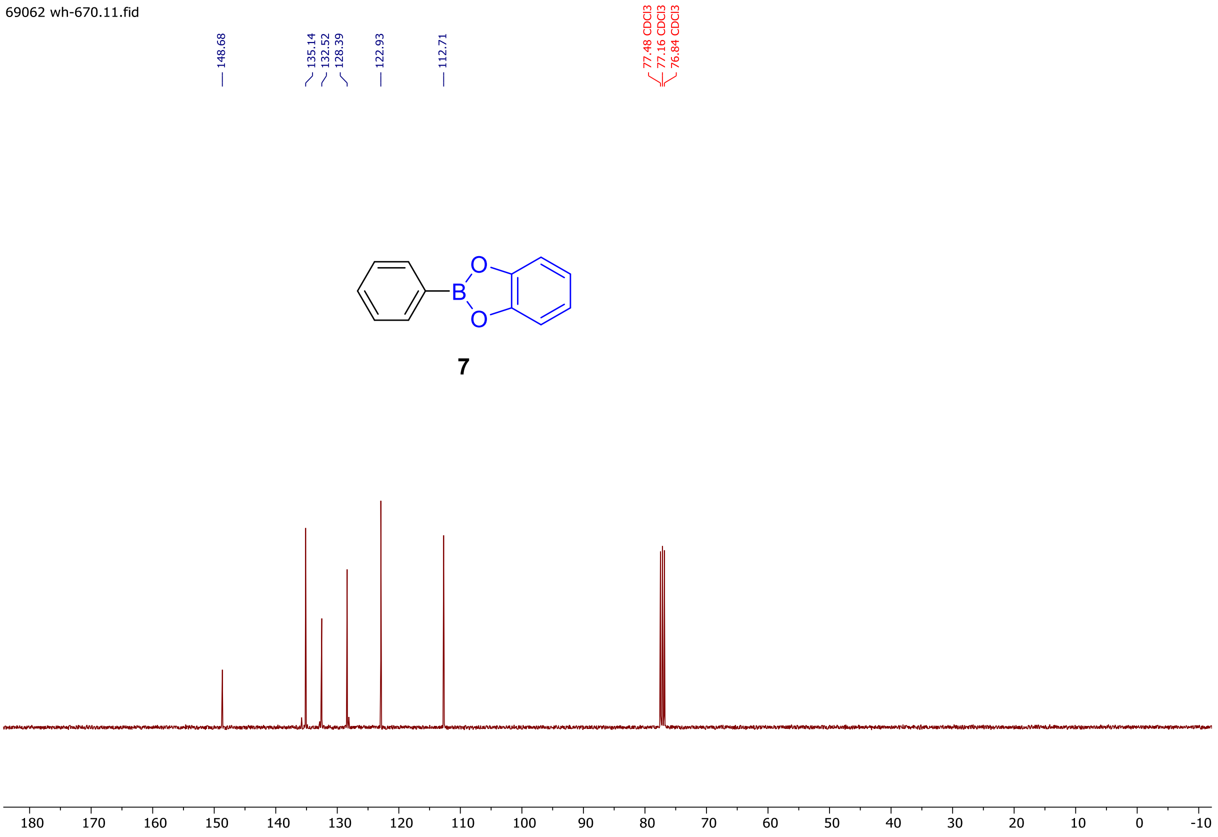

^{1}H NMR (400 MHz, $CDCl_3$) of **6tc** (*see procedure*)

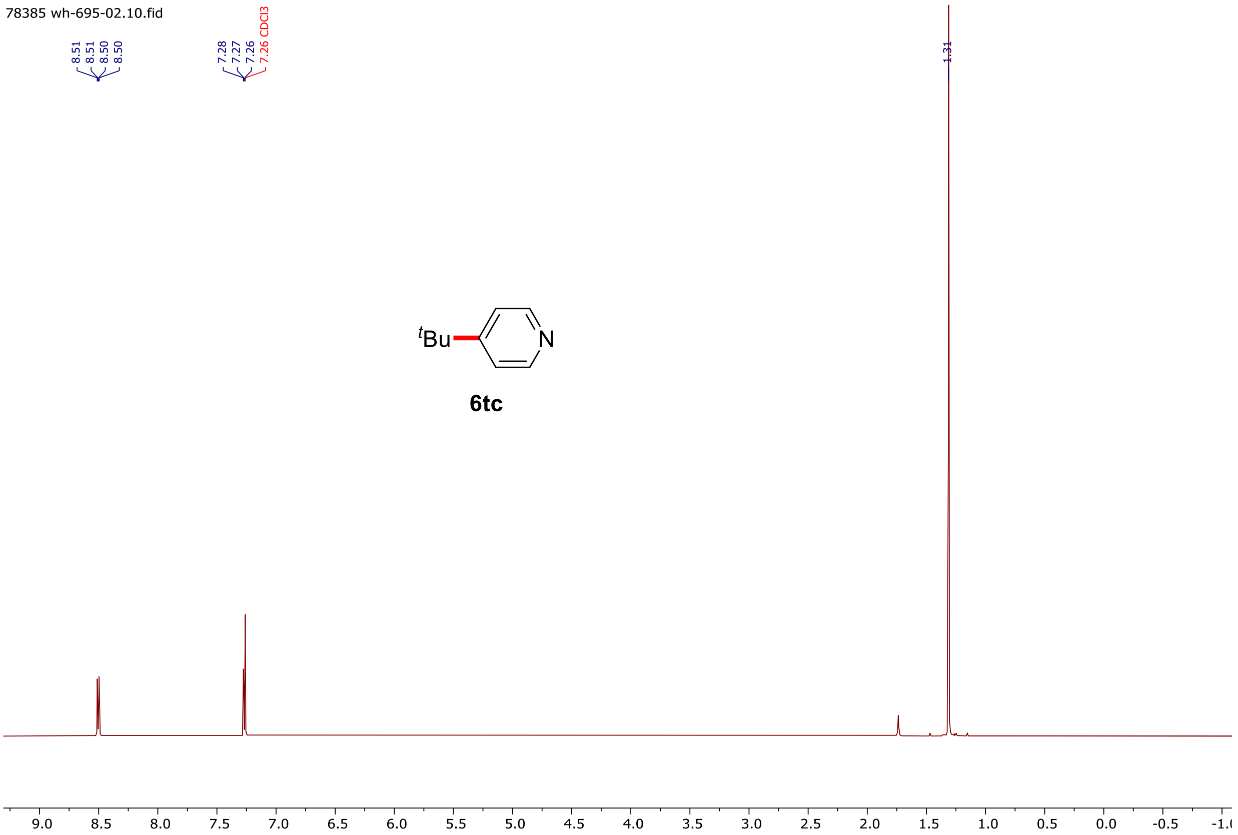

^{13}C NMR (101 MHz, $CDCl_3$) of **6tc**

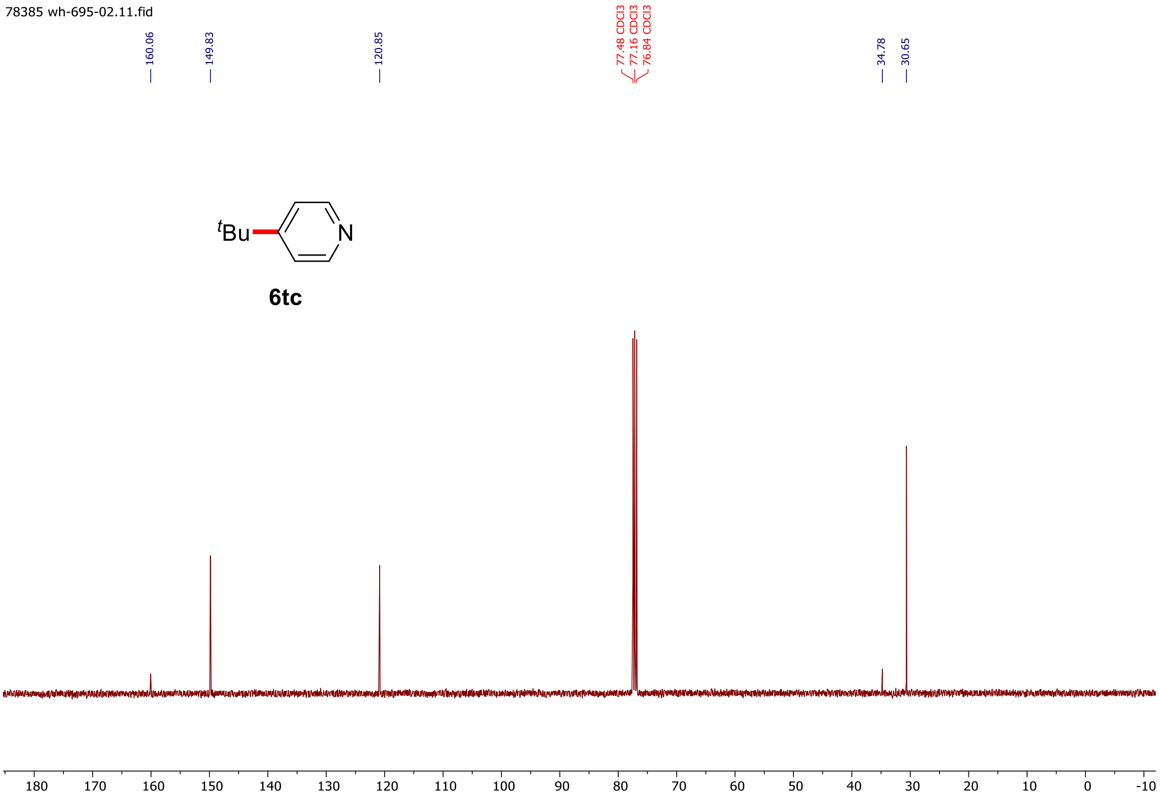

4. REFERENCES

[1] A. F. Burchat, J. M. Chong, N. Nielsen, *J. Organomet. Chem.* **1997**, *542*, 281-283.

[2] A. Bonet, C. Pubill-Ulldemolins, C. Bo, H. Gulyás, *Angew. Chem. Int. Ed.* **2011**, *50*, 7158-7161; *Angew. Chem.* **2011**, *123*, 7296-7299.

[3] A. Farre, K. Soares, R. A. Briggs, A. Balanta, D. M. Benoit, A. Bonet, *Chem. Eur. J.* **2016**, *22*, 17552-17556.

[4] C. Yin, K. Zhong, W. Li, X. Yang, R. Sun, C. Zhang, X. Zheng, M. Yuan, R. Li, Y. Lan, H. Fu, H. Chen, *Adv. Synth. Catal.* **2018**, *360*, 3990-3998.

[5] A. Bonet, M. Odachowski, D. Leonori, S. Essafi, V. K. Aggarwal, *Nat. Chem.* **2014**, *6*, 584-589.

[6] S. Roesner, C. A. Brown, M. Mohiti, A. P. Pulis, R. Rasappan, D. J. Blair, S. Essafi, D. Leonori, V. K. Aggarwal, *Chem. Commun.* **2014**, *50*, 4053-4055.

[7] A. Fawcett, J. Pradeilles, Y. Wang, T. Mutsuga, E. L. Myers, V. K. Aggarwal, *Science* **2017**, *357*, 283-286.

[8] V. Ganesh, M. Odachowski, V. K. Aggarwal, *Angew. Chem. Int. Ed.* **2017**, *56*, 9752-9756; *Angew. Chem.* **2017**, *129*, 9884-9888.

[9] A. Varela, L. K. B. Garve, D. Leonori, V. K. Aggarwal, *Angew. Chem. Int. Ed.* **2017**, *56*, 2127-2131; *Angew. Chem.* **2017**, *129*, 2159-2163.

[10] R. C. Mykura, S. Veth, A. Varela, L. Dewis, J. J. Farndon, E. L. Myers, V. K. Aggarwal, *J. Am. Chem. Soc.* **2018**, *140*, 14677-14686.

[11] A. Fawcett, A. Murtaza, C. H. U. Gregson, V. K. Aggarwal, *J. Am. Chem. Soc.* **2019**, *141*, 4573-4578.

[12] D. Kaiser, A. Noble, V. Fasano, V. K. Aggarwal, *J. Am. Chem. Soc.* **2019**, *141*, 14104-14109.

[13] C. Shu, A. Noble, V. K. Aggarwal, *Angew. Chem. Int. Ed.* **2019**, *58*, 3870-3874; *Angew. Chem.* **2019**, *131*, 3910-3914.

[14] V. Fasano, N. Winter, A. Noble, V. K. Aggarwal, *Angew. Chem. Int. Ed.* **2020**, *59*, 8502-8506; *Angew. Chem.* **2020**, *132*, 8580-8584.

[15] S. Willems, G. Toupalas, J. C. Reisenbauer, B. Morandi, *Chem. Commun.* **2021**, *57*, 3909-3912.

[16] X.-X. Wang, L. Li, T.-J. Gong, B. Xiao, X. Lu, Y. Fu, *Org. Lett.* **2019**, *21*, 4298-4302.

[17] A. Fawcett, D. Nitsch, M. Ali, J. M. Bateman, E. L. Myers, V. K. Aggarwal, *Angew. Chem. Int. Ed.* **2016**, *55*, 14663-14667; *Angew. Chem.* **2016**, *128*, 14883-14887.

[18] S. Zhou, Y. Pu, Z. Liu, X. Zhang, J. Zhu, Z. Feng, *Org. Lett.* **2021**, *23*, 5565-5570.

[19] C. Pubill-Ulldemolins, M. Poyatos, C. Bo, E. Fernández, *Dalton Trans.* **2013**, *42*, 746-752.

[20] M. Auffray, D. H. Kim, J. U. Kim, F. Bencheikh, D. Kreher, Q. Zhang, D A. 'Aléo, J.-C. Ribierre, F. Mathevet, C. Adachi, *Chem. Asian J.* **2019**, *14*, 1921-1925.

[21] M. Van Gool, S. A. Alonso De Diego, O. Delgado, A. A. Trabanco, F. Jourdan, G. J. Macdonald, M. Somers, L. Ver Donck, *ChemMedChem* **2017**, *12*, 905-912.

[22] C. A. Sehon, G. Z. Wang, A. Q. Viet, K. B. Goodman, S. E. Dowdell, P. A. Elkins, S. F. Semus, C. Evans, L. J. Jolivette, R. B. Kirkpatrick, E. Dul, S. S. Khandekar, T. Yi, L. L. Wright, G. K. Smith, D. J. Behm, R. Bentley, C. P. Doe, E. Hu, D. Lee, *J. Med. Chem.* **2008**, *51*, 6631-6634.

[23] R. Wang, J. R. Falck, *Org. Chem. Front.* **2014**, *1*, 1029-1034.

[24] C.-T. Yang, Z.-Q. Zhang, H. Tajuddin, C.-C. Wu, J. Liang, J.-H. Liu, Y. Fu, M. Czyzewska, P. G. Steel, T. B. Marder, L. Liu, *Angew. Chem. Int. Ed.* **2012**, *51*, 528-532; *Angew. Chem.* **2012**, *124*, 543-547.

UOB Confidential & Sensitive

[25] Y. Wang, R. Guan, P. Sivaguru, X. Cong, X. Bi, *Org. Lett.* **2019**, *21*, 4035-4038.

[26] C. Pubill-Ulldemolins, A. Bonet, H. Gulyás, C. Bo, E. Fernández, *Org. Biomol. Chem.* **2012**, *10*, 9677-9682.

[27] M. A. Reichle, B. Breit, *Angew. Chem. Int. Ed.* **2012**, *51*, 5730-5734; *Angew. Chem.* **2012**, *124*, 5828-5832.

[28] J. Schmidt, J. Choi, A. T. Liu, M. Slusarczyk, G. C. Fu, *Science* **2016**, *354*, 1265-1269.

[29] D. Wang, C. Mück-Lichtenfeld, A. Studer, *J. Am. Chem. Soc.* **2019**, *141*, 14126-14130.

[30] K. Semba, Y. Nakao, *J. Am. Chem. Soc.* **2014**, *136*, 7567-7570.

[31] W. Wang, C. Ding, H. Pang, G. Yin, *Org. Lett.* **2019**, *21*, 3968-3971.

[32] P. Zhang, J. Dong, B. Zhong, D. Zhang, H. Yuan, C. Jin, X. Xu, H. Li, Y. Zhou, Z. Liang, M. Ji, T. Xu, G. Song, L. Zhang, G. Chen, X. Meng, D. Sun, J. Shih, R. Zhang, G. Hou, C. Wang, Y. Jin, Q. Yang, *Bioorg. Med. Chem. Lett.* **2016**, *26*, 1910-1918.

[33] J. Luo, J. Zhang, *ACS Catal.* **2016**, *6*, 873-877.

[34] M. D. Rathnayake, J. D. Weaver, *Org. Lett.* **2021**, *23*, 2036-2041.

[35] G. A. Molander, F. Vargas, *Org. Lett.* **2007**, *9*, 203-206.

[36] P. Basnet, S. Thapa, D. A. Dickie, R. Giri, *Chem. Commun.* **2016**, *52*, 11072-11075.

[37] J. D. St. Denis, C. C. G. Scully, C. F. Lee, A. K. Yudin, *Org. Lett.* **2014**, *16*, 1338-1341.

[38] S. A. Green, S. Vásquez-Céspedes, R. A. Shenvi, *J. Am. Chem. Soc.* **2018**, *140*, 11317-11324.

[39] Z. Zuo, D. W. C. MacMillan, *J. Am. Chem. Soc.* **2014**, *136*, 5257-5260.

[40] J. C. Tellis, J. Amani, G. A. Molander, *Org. Lett.* **2016**, *18*, 2994-2997.

[41] O. Grossman, D. Gelman, *Org. Lett.* **2006**, *8*, 1189-1191.

[42] G. A. Molander, O. A. Argintaru, I. Aron, S. D. Dreher, *Org. Lett.* **2010**, *12*, 5783-5785.

[43] M. Montalti, A. Credi, L. Prodi, M. T. Gandolfi, Handbook of Photochemistry (CRC/Taylor & Francis, Boca Raton, FL, ed. 3, 2006).

[44] A. Tlahuext-Aca, R. A. Garza-Sanchez, M. Schäfer, F. Glorius, *Org. Lett.* **2018**, *20*, 1546-1549.

[45] V. Desrosiers, C. Z. Garcia, F.-G. Fontaine, *ACS Catal.* **2020**, *10*, 11046-11056.

[46] J. Choi, G. Laudadio, E. Godineau, P. S. Baran, *J. Am. Chem. Soc.* **2021**, *143*, 11927-11933.

[47] S. Macur, B. T. Farmer, L. R. Brown, *J. Magn. Reson.* **1986**, *70*, 493-499.

[48] H. Hu, K. Krishnamurthy, *J. Magn. Reson.* **2006**, *182*, 173-177.

[49] C. P. Butts, C. R. Jones, Z. Song, T. J. Simpson, *Chem. Commun.* **2012**, *48*, 9023-9025.

[50] J. Wu, P. Lorenzo, S. Zhong, M. Ali, C. P. Butts, E. L. Myers, V. K. Aggarwal, *Nature* **2017**, *547*, 436-440.

[51] L. Guo, O. J. Dutton, M. Kucukdisli, M. Davy, O. Wagnières, C. P. Butts, E. L. Myers, V. K. Aggarwal, *J. Am. Chem. Soc.* **2021**, *143*, 16682-16692.

UOB Confidential & Sensitive
